# Dearomative *syn*-Dihydroxylation of Naphthalenes with a Biomimetic Iron Catalyst

**DOI:** 10.1021/jacs.3c08565

**Published:** 2023-12-20

**Authors:** Najoua Choukairi Afailal, Margarida Borrell, Marco Cianfanelli, Miquel Costas

**Affiliations:** Institut de Química Computacional i Catàlisi (IQCC) and Departament de Química, Universitat de Girona, Campus Montilivi, Girona E-17071, Catalonia, Spain

## Abstract

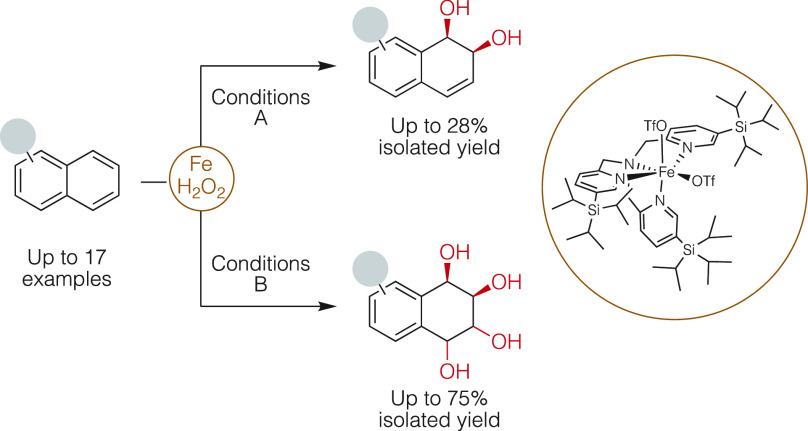

Arenes are interesting feedstocks for organic synthesis because of their natural abundance. However, the stability conferred by aromaticity severely limits their reactivity, mostly to reactions where aromaticity is retained. Methods for oxidative dearomatization of unactivated arenes are exceedingly rare but particularly valuable because the introduction of Csp^3^–O bonds transforms the flat aromatic ring in 3D skeletons and confers the oxygenated molecules with a very rich chemistry suitable for diversification. Mimicking the activity of naphthalene dioxygenase (NDO), a non-heme iron-dependent bacterial enzyme, herein we describe the catalytic *syn*-dihydroxylation of naphthalenes with hydrogen peroxide, employing a sterically encumbered and exceedingly reactive yet chemoselective iron catalyst. The high electrophilicity of hypervalent iron oxo species is devised as a key to enabling overcoming the aromatically promoted kinetic stability. Interestingly, the first dihydroxylation of the arene renders a reactive olefinic site ready for further dihydroxylation. Sequential bis-dihydroxylation of a broad range of naphthalenes provides valuable tetrahydroxylated products in preparative yields, amenable for rapid diversification.

## Introduction

The abundance of arenes makes them valuable feedstocks for organic synthesis. Functionalization of arenes usually relies on methodologies in which aromaticity is maintained. Alternatively, dearomatization reactions are particularly valuable because they install multiple sp^3^ carbon centers, transforming the flat aromatic structure in three-dimensional polyfunctionalized platforms, structurally rich and chemically versatile.^1−6^

**Figure 1:**
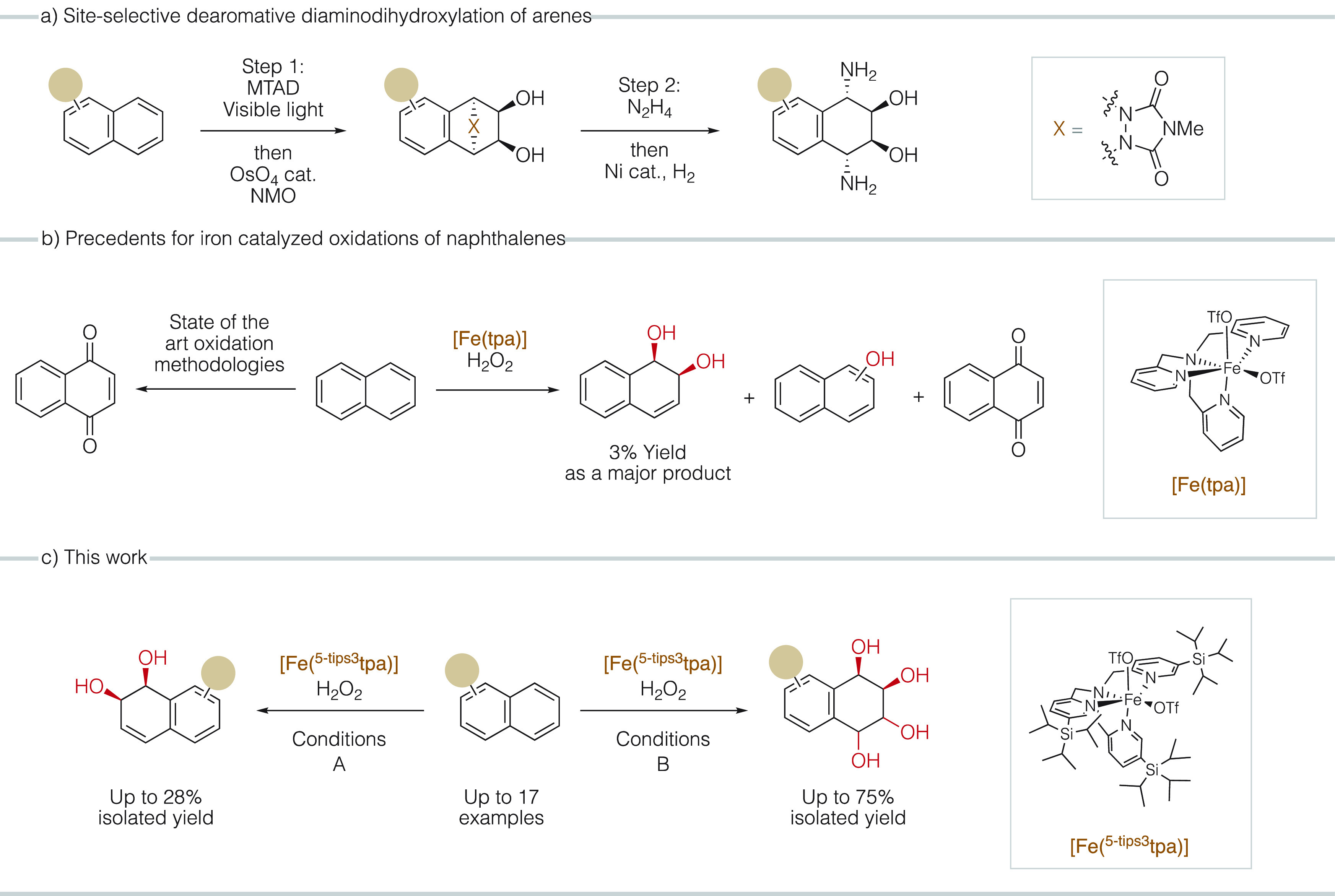
(a) Site-selective dearomative diaminodihydroxylation of napthalenes with arenophiles (MTAD; *N*-methyl-1,2,4-triazoline-3,5-dione). (b) Precedents for iron catalyzed oxidation of naphthalene and reported *syn*-dihydroxylation of naphthalene catalyzed by the [Fe(tpa)] complex. (c) This work, detailing chemoselective *syn*-dihydroxylation of naphthalenes.

Oxidative dearomatization transformations constitute some of the most appealing reactions since they provide oxygenated scaffolds that serve as starting point for the elaboration of numerous products of biological relevance.^6−8^ However, this class of reactions constitutes a paradigmatic example of the difficulties posed by dearomative functionalization of unactivated arenes (Figure 1).^3^ Traditional alkene epoxidizing and dihydroxylation agents are poorly reactive against unactivated arenes.^9−13^ On the other hand, catalytic oxidations with first-row transition metal catalysts can generate powerful oxidants that overcome the kinetic stability of arenes, but their reactions result in the formation of phenols and quinones (Figure 1b).^14−23^

A recent breakthrough to solve this problem disclosed a photochemically promoted [4 + 2] addition of N–N arenophiles to unactivated arenes, removing aromaticity. This way, the diene motif becomes reactive against conventional dihydroxylating or epoxidizing agents, resulting in diaminodihydroxylated derivatives. Removal of the arenophile provides the diols and oxepins that can be further transformed into a diversity of natural products (Figure 1a).^24−26^

On the other hand, contrasting with the scarcity of traditional synthetic methods, several classes of enzymes oxidatively dearomatize arenes. Such reactivity has converted them into the single alternative to address the high interest in these reactions.^7,8,27^ This approach presents several limitations because it requires the use of whole cells, which may be methodologically challenging, and in addition, access to the bacterial strains may be limited.

One of the best studied enzymes is naphthalene 1,2-dioxygenase (NDO), a bacterial non heme iron dependent enzyme from the family of Rieske dioxygenases, which performs the *syn*-dihydroxylation of naphthalene as the first step in the biological degradation of this molecule.^27−29^ Taking inspiration from nature, small molecule iron complexes that serve as functional models of NDO have been developed in the past years. Some of those complexes may provide excellent yields for the *syn*-dihydroxylation of alkenes in preparative yields, making them an attractive alternative to well-established osmium-based reactions.^30−48^

However, the use of arenes as substrates remains a more challenging and standing problem. A single example has been reported by Que and co-workers using the iron complex [Fe(OTf)_2_(tpa)], [**(Fe(tpa)**], where tpa stands for the tetradentate tris-2-pyridylmethylamine ligand. Employing a large excess of the substrate and hydrogen peroxide as oxidant and limiting reagent, *cis*-1,2-dihydro-1,2-naphthalenediol was obtained in a modest 3%, along with modest chemoselectivity; 1-naphthol, 2-naphthol and 1,4-naphthoquinone were also obtained in comparable amounts (Figure 1b).^49^

More recently, we have described a modified tpa-based catalyst, [Fe(OTf)_2_(^5-tips3^tpa)], [**(Fe(**^**5-tips3**^**tpa)**] (Figure 1), which incorporates sterically demanding triisopropyl-silyl groups, (tips) in the structure of the ligands and catalyzes the *syn*-dihydroxylation of a broad range of olefins with excellent yields (up to 97% of isolated yield) and chemoselectivity.^50−52^ The steric isolation of the iron center in this catalyst was key to slowing down the formation of catalytically inactive oxo-bridged diiron complexes. In addition, steric isolation promotes the release of diol product from the metal center. This is important because it is the rate-determining step of the catalytic reaction. The use of Mg(ClO_4_)_2_·6H_2_O synergistically assists catalytic turnover since this Lewis acid captures the diol produced, so it does not bind back to the metal center preventing catalyst arrest. Interestingly, subsequent mechanistic studies in gas-phase suggested that arenes are also suitable substrates for this catalyst, despite only naphthols and not dihydrodiols were observed in the gas phase.^53^

Building on these precedents, herein we develop a synthetic methodology for the *syn*-dihydroxylation of naphthalene derivatives using **[Fe(**^**5-tips3**^**tpa)]** under substrate-limiting conditions using hydrogen peroxide as an oxidant (Figure 1c). Substrate scope and reaction mechanism are investigated, pointing toward the implication of a highly reactive yet chemoselective Fe^V^(O)(OH) intermediate. Interestingly, dihydroxylation of naphthalene unleashes reactivity of the adjacent olefinic site, which is further hydroxylated. The reaction displays a broad substrate scope and delivers tetrahydroxylated products at the nonfunctionalized ring of substituted naphthalenes, while retaining arene functionalities suitable for further elaboration.

## Results and Discussion

### Reaction Design and Optimization

Our initial hypothesis was that the excellent activity of **[Fe(**^**5-tips3**^**tpa)]** in the *syn*-dihydroxylation of alkenes makes it a potential candidate for performing the dihydroxylation of the comparatively less reactive arene substrates. To evaluate this hypothesis, our study was initiated by performing the oxidation of naphthalene under oxidant-limiting conditions. In a typical experiment, a solution of hydrogen peroxide (0.2 equiv with respect to the substrate in acetonitrile solution) was delivered via a syringe pump during 30 min at room temperature to a solution containing naphthalene (1 equiv), catalyst (**[Fe(**^**5-tips3**^**tpa)]**, 13.5 μM, 3 mol % with respect to the substrate), and Mg(ClO_4_)_2_·6H_2_O (4.4 equiv) in acetonitrile in a vial open to air. Following peroxide addition, the mixture was subjected to an acetylation workup and analyzed by gas chromatography. The reaction produced the acetylated diol in 35% yield, which could be improved up to 39% yield by using 6 mol % of the catalyst.

Performing the reaction under stoichiometric (substrate:oxidant ratio) conditions was then explored. Results are listed in Table 1. By employing 3 mol % catalyst and 1 equiv of H_2_O_2_ (conditions A) the reaction delivered a 1:1 mixture of *syn*-diol (11% yield) and overoxidized tetraol (11% yield, *syn*/*anti* = 6.2) (entry 1, Table 1). As previously seen in the *syn*-dihydroxylation of simple alkenes, the addition of Mg(ClO_4_)_2_·6H_2_O as additive had a strong positive impact in yields, chemoselectivities, and mass balance of the reactions, presumably because the binding and sequestering of the diol products by the Mg^2+^ cations protect them against overoxidative degradation while leaving the iron center available for initiating the following catalytic cycle. Using 4.4 equiv of Mg(ClO_4_)_2_·6H_2_O, the yield and chemoselectivity of the reaction improved, obtaining the *syn*-diol **2a** in 29% yield, along with minor amounts of tetraol **3a** (5% combined yield, *syn*/*anti* = 3, entry 2, Table 1). Naphthol and naphthoquinone, products commonly obtained in the oxidation of naphthalene with metal catalysts,^14−20,23,49^ were only detected in trace amounts (<2%). The recovered starting material (**1a**) was 50% and a blank experiment where the same reaction was conducted in the absence of catalyst showed losses of 5% during workup. This leads to an estimated mass balance of 82%. Therefore, we conclude that under 1:1 oxidant:substrate ratio conditions, **[Fe(**^**5-tips3**^**tpa)]** catalyzes the *syn*-dihydroxylation of naphthalene in a chemoselective manner.

**Table 1:**
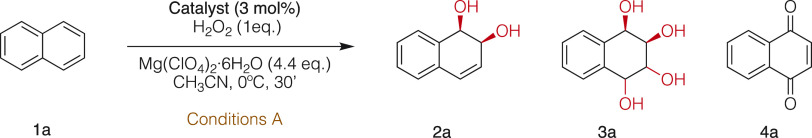
Comparison of Diol Formation Using Different Iron Catalysts^a^

entry	catalyst	yield of **2a** (%)	yield of **3a** (%) (*syn*/*anti*)	yield of **4a** (%)
1^b^	**[Fe(**^**5-tips3**^**tpa)]**	11	11 (6.2)	1
2	**[Fe(**^**5-tips3**^**tpa)]**	29	5 (3.2)	2
3	**[Fe(tpa)]**	9	1 (1.3)	3
4	**[Fe(**^**5-tips2**^**tpa)]**	17	1 (1.8)	3
5	**[Fe(**^**5-tips2,6-Me**^**tpa)]**	14	4 (3.5)	2
6	**[Fe(**^**5-tips3,4-NMe2**^**tpa)]**	13	1 (1.8)	<1
7	**[Fe(**^**COOEt**^**pytacn)]**	7	3 (3.8)	4
8	**[Fe(**^**6-Me**^**pytacn)]**	1	<1 (2.5)	4

a 3 mol % catalyst, 1 equiv of H_2_O_2_, 4.4 equiv of Mg(ClO_4_)_2_·6H_2_O, CH_3_CN, 30′, 0 °C. Yields determined by GC with the response factor of the products. Replicates are included in Table S19. Differences between duplicates are <3%.

b Reaction without Mg(ClO_4_)_2_·6H_2_O.

Recognizing the interest of polyhydroxylated compounds containing adjacent hydroxyl groups and the general difficulty of their preparation,^54^ we sought conditions that could maximize the formation of the tetraol products. Pleasantly, we found that a second addition of oxidant and catalyst (conditions B) yields the tetraol in 47% yield (GC yield, entry 5, Table S3). Various organic additives were explored as potentially trapping diol products different from Mg(ClO_4_)_2_·6H_2_O (acetone, hexafluoroacetone, boronic acids, 1,1′-carbonyl-diimidazole) (Table S10). However, they did not exert the positive effect of Mg(ClO_4_)_2_·6H_2_O. A solvent screening was also conducted (Table S12) showing that acetonitrile and butyronitrile are optimal solvents, ethyl acetate and propylene carbonate are tolerated, but THF and DMF completely suppress reactivity. The reaction also proved to be incompatible with the strong hydrogen donor solvent HFIP.

### Catalyst Dependent Activity

**Figure 2:**
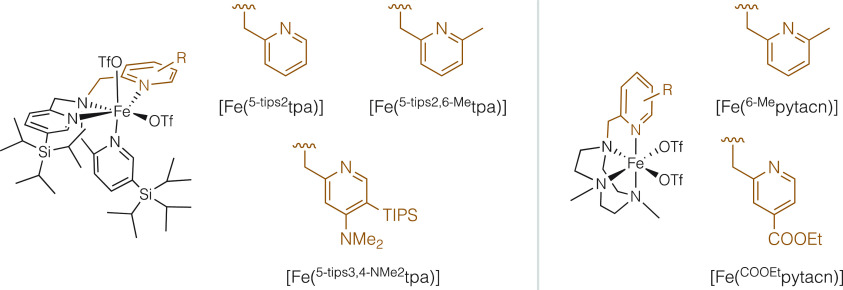
Catalysts tested in this work.

The catalytic competence of **[Fe(**^**5-tips3**^**tpa)]** in *syn*-dihydroxylation is best placed in context when compared with other iron tetradentate complexes under analogous reaction conditions (Table 1, Figure 2). Use of iron C–H hydroxylation and epoxidation catalysts based on linear tetradentate ligands (Fe(pdp) (pdp; *N*,*N*′-bis(2-pyridylmethyl)-2,2′-bipyrrolidine) and Fe(mcp) (mcp; *N*,*N*′-dimethyl-*N*,*N*′-bis(pyridine-2-ylmethyl)-cyclohexane-1,2-diamine) provided complex mixtures, where dearomatized products could not be identified.^55,56^ Naphthols and naphthoquinones were detected in minor amounts among multiple nonidentified products. Instead, the unsubstituted **[Fe(tpa)]** catalysts (entry 3, Table 1) provided lower yields (9% diol), further showing the crucial role of the ligand structure in the outcome of the reaction. This led us to explore different modifications of the **[Fe(**^**5-tips3**^**tpa)]** catalyst (Figure 2). The removal of a single tips group in one of the pyridine arms resulted in a substantial decrease in yields (17%, entry 4, Table 1) highlighting the important role of steric demand. Replacement of pyridine by 6-Me picoline groups generally increase the selectivity toward *syn*-dihydroxylation versus epoxidation of olefins in previously described iron oxidation catalysts.^40,41,57−59^ However, the replacement of one of the tips substituted pyridines by a 6-methyl picoline resulted in a catalyst **[Fe(**^**5-tips3,6-Me**^**tpa)]** that gives a modest 14% yield of diol and a decreased chemoselectivity (entry 5, Table 1) when compared with **[Fe(**^**5-tips3**^**tpa)]**. Introduction of an electron-donating group in the pyridine to decrease the electrophilicity of the metal center was also explored, aiming at minimizing overoxidation processes, but the resulting catalyst **[Fe(**^**5-tips3,4-NMe2**^**tpa)]** showed modest activity (13% yield, entry 6, Table 1) suggesting that the arene dihydroxylating reactivity is limited when the electrophilicity of the catalysts is reduced. Finally, two triazacyclononane-based complexes were tested (**[Fe(**^**COOEt**^**pytacn)]** and **[Fe(**^**6-Me**^**pytacn)]**,entries 7 and 8, respectively, Table 1) since these have shown good activity in the *syn*-dihydroxylation of olefins.^59,60^ The two catalysts displayed catalytic activity in the *syn*-dihydroxylation of naphthalene; however low yields (7% and 1% of diol, respectively) and poor chemoselectivity were observed since in both cases naphthoquinone was formed in a comparable amount with respect to the diol. We concluded that **[Fe(**^**5-tips3**^**tpa)]** is the best catalyst of the series and was used in the exploration of the substrate scope.

### Substrate Scope under Conditions To Favor the Mono-*syn*-dihydroxylation

**Figure 3:**
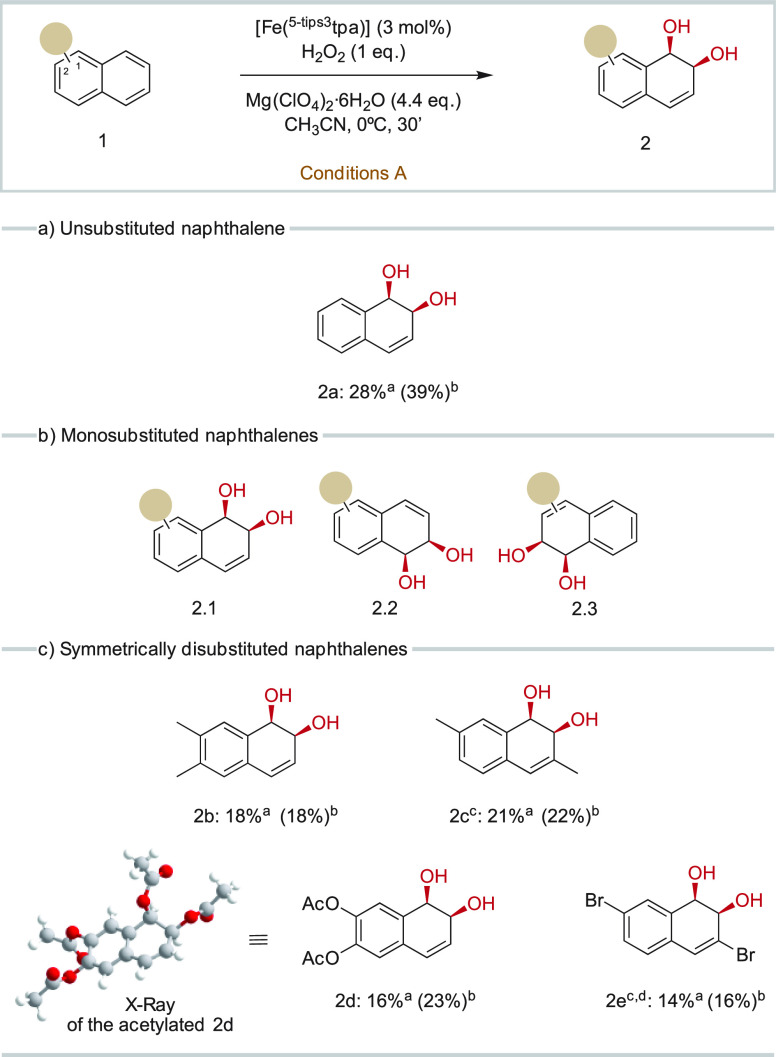
Substrate scope for the dihydroxylation reaction. (a) Naphthalene. (b) Isomeric diols resulting from the dihydroxylation of monosubstituted naphtalenes. (c) Different disubstituted naphthalene derivatives where a single diol isomer is obtained in their oxidation. ^a^Isolated yields. Reaction conditions: 1 equiv of substrate, 3 mol % catalyst, 1 equiv of H_2_O_2_, 4.4 equiv of Mg(ClO_4_)_2_·6H_2_O, CH_3_CN, 30′, 0 °C. ^b^NMR yields. Reaction performed with 5 equiv of substrate under the same conditions. ^c^Reaction performed at rt. ^d^Double addition of catalyst (3 mol % each addition) and H_2_O_2_ (1.5 equiv of each addition) at 30′ of reaction, total reaction time 1 h, reaction mixture AcOEt:CH_3_CN (1:1).

Under the best conditions found for the mono *syn*-dihydroxylation reaction (conditions A), different naphthalene derivatives were tested (Figure 3). Under these conditions, naphthalene delivered the best isolated yield of diol (28%, **2a**, Figure 3a). Monosubstituted naphthalenes constitute a particularly difficult type of substrate because multiple *syn*-diol regioisomers are possible (Figure 3b). Indeed, their oxidation delivered a mixture of three diols, indicating a modest control on the site selectivity of the first dihydroxylation reaction (Figure 3b, see Supporting Information, Table S26 for details). In addition, we find that the oxidation of 2-substituted (**1b**–**1e**) naphthalenes generally provide better yields than 1-substituted naphthalenes (see Supporting Information, Table S26).

Instead, when symmetrically disubstituted naphthalenes were tested, a single *syn*-diol isomer was observed, where dihydroxylation has taken place at nonsubstituted sites. Disubstituted substrates with the two substituents in the same ring (**2b** and **2d**, Figure 3c) and with the substituents symmetrically placed on different rings (**2c** and **2e**, Figure 3c) were also tested, in all cases providing low to moderate yields (21–14%). Use of excess substrate (5 equiv) conditions provided comparable product yields.

Summarizing, yields for dihydroxylation are modest, but it should be considered that the reaction constitutes a single step and easily scalable procedure to access these valuable molecules from readily available naphthalenes. Besides, some of the products are so far only accessible by whole cell enzymatic methods.

### Substrate Scope under Conditions To Favor Tetraol Formation

**Figure 4:**
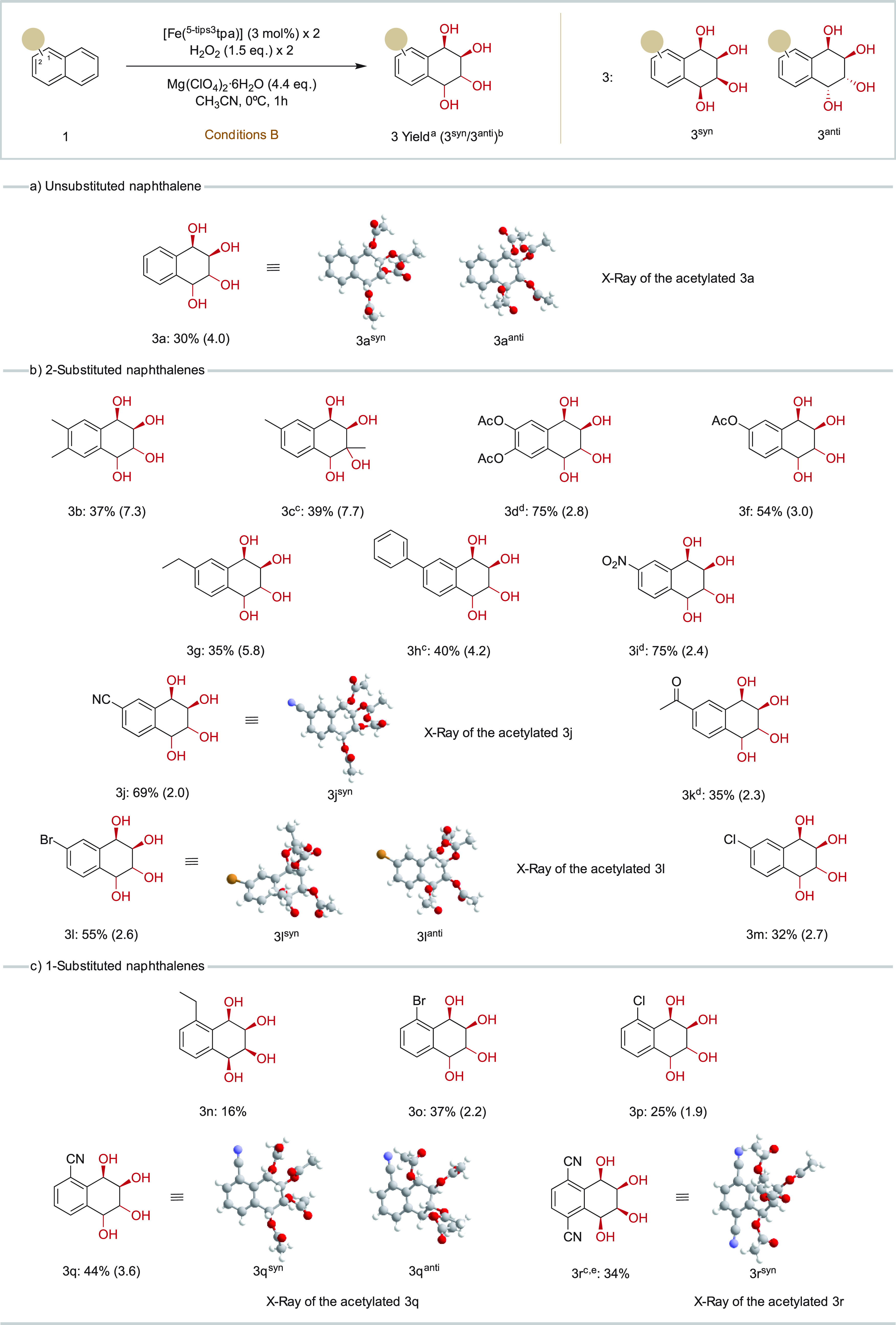
Substrate scope of different naphthalene derivatives for tetraol formation. (a) Nonsubstituted naphthalene. (b) Naphthalenes with the substituent in position 2. (c) Naphthalenes with the substituent in position 1. ^a^Isolated yields. Reaction conditions: 1 equiv of substrate, 3 mol % catalyst (with a second addition of 3 mol % in 30′), 1.5 equiv of H_2_O_2_ (with a second addition and 1.5 equiv of H_2_O_2_ in 30′), 4.4 equiv of Mg(ClO_4_)_2_·6H_2_O, CH_3_CN, 30′, 0 °C. ^b^[3^*syn*^/3^*anti*^] ratio (in parentheses) was determined by ^1^H NMR. ^c^Reaction performed at rt. ^d^Additional 3 mol % catalyst and 1.5 equiv of H_2_O_2_ at 1 h, final reaction time 1.5 h. ^e^Reaction mixture 1:1 (CH_3_CN:AcOEt).

The performance of the reaction was then explored under the reaction conditions to favor the formation of the corresponding tetraols (conditions B). In this case, the substrate scope was not limited to naphthalenes with symmetric substitution patterns since, irrespective of the isomer first formed, the two sequential dihydroxylation events on a ring converged in a single structural isomer (Figure 4), obtaining the products in satisfactory yields.

The oxidation of each of the substrates gave a mixture of two diastereomers resulting from *syn* or *anti* bis-dihydroxylation (**3**^***syn***^ and **3**^***anti***^), *syn* being the major product in all cases. Oxidation of **1d** was explored using different Lewis acids, aiming at controlling the diastereoselectivity of the reaction (see Supporting Information, Table S18). Effectively, the *syn/anti* ratio appears to be dependent on the nature of the Lewis acid. The largest *syn*/anti ratio was obtained in the absence of Lewis acid (see Supporting Information, entry 1, Table S18), while Zn(OTf)_2_ delivered the smaller (1.5) *syn*/anti ratio (see Supporting Information, entry 2, Table S18). Mg(ClO_4_)_2_·6H_2_O provided the best yield while providing an intermediate ratio (2.4, see Supporting Information, entry 3, Table S18) and was chosen for substrate scope exploration.

Unsubstituted naphthalene was oxidized in 30% isolated yield (47% by GC analysis of the crude) of the tetraol with a *syn*:/*anti* ratio of 4 (**3a**, Figure 4a). A series of naphthalenes bearing substituents in position 2 were oxidized in modest to good (32–75%) yields (Figure 4b). Of note is that isolated tetrahydroxylated products contain the substituted ring intact. Naphthalene bearing alkyl groups (**3b**, **3c**, and **3g**) presented moderate yields (35–39%), and in these cases, the ratio of *syn*/*anti* was substantially higher than in the case of naphthalene (7.3, 7.7, and 5.8, respectively, Figure 4). The 2-phenyl-substituted naphthalene substrate **1h** was oxidized in a slightly improved 40% yield and a lower *syn*/*anti* diastereoselectivity (4.2, **3h**, Figure 4). No products resulting from oxidation of the phenyl substituent were detected, attesting to the expected higher reactivity of naphthalene over benzene rings. Furthermore, a competitive oxidation of a 1:1 mixture of benzene and naphthalene yielded only diols resulting from the oxidation of naphthalene (**2a** and **3a**) and trace amounts of benzoquinone (Table S20). Oxidation of the naphthalene over the benzene ring in **3h** reflects the higher energy of the HOMO in the former, more susceptible to electrophilic attack.^25^ Naphthalenes bearing electron-withdrawing groups in position 2 are oxidized in remarkably high yields and selectivities. Mono- and diacetate naphthalenes deliver the corresponding tetraols in 54% and 75% yields, respectively, and moderate (2.8–3.0) diatereoselectivities (**3f** and **3d**). Nitro (**1i**) and cyano (**1j**) substituted naphthalenes are oxidized to the corresponding tetraols in 75% and 69% yields with a diastereomeric ratio of 2.4 and 2.0, respectively (**3i** and **3j**). Finally, the oxidation of 2-acetonaphthone **1k** yielded the corresponding tetraol in a modest 35% yield (dr 2.3, **3k**). The oxidation of halogenated substrates also provided satisfactory yields. Oxidation of 2-bromonaphthalene (**1l**) and 2-chloronaphthalene (**1m**) delivered the corresponding tetraol products in 55% and 32% yield, respectively, with a *syn*/*anti* ratio of around 2.5 (**3l** and **3m**). When two halogen groups were placed on different rings, the tetraol is not formed, and only the *syn*-diol (**2e**, Figure 3) could be isolated, suggesting that the halide substituted olefin site is unreactive. The comparatively high yields obtained for electron-poor substrates suggest that oxidative degradation may be the reason for the modest yields in the case of substrates with electron-rich rings. Unfortunately, no dominant overoxidation products could be isolated and identified. Nevertheless, the reaction is notable because it provides a single step access to products that contain the tetrahydroxylated ring adjacent to a derivatized arene containing C_arene_–C, C_arene_–N, C_arene_–O and C_arene_–halide functionality. The chemical versatility of this functionality facilitates further elaboration by conventional methods. For example, the halide groups are versatile handles for further elaboration via organometallic cross-coupling transformations.

Naphthalenes substituted at position 1 were also suitable substrates (Figure 4c), although yields are reduced when compared with 2-substituted substrates. 1-Ethyl substituted naphthalene provides the tetraol **3n** in a modest 16% isolated yield. Synthetically more valuable yields (37% and 25%) were obtained in the oxidation of chemically versatile halide substituted naphthalenes **1o** and **1p**, respectively. And as observed in the case of 2-substituted substrates, 1-substituted naphthalenes with the strong electron-withdrawing cyanide group led to the best yields (44% for **3q** and 34% for **3r**). An aspect that deserves consideration is that significant amounts (1–13%) of isomeric diol products where oxidation took place at the substituted ring could be detected in the ^1^H NMR spectra of the crude reaction mixtures. However, they decompose during the purification process, and only a small amount of product was recovered (see Supporting Information).

In summary, the reaction proceeds with synthetically valuable modest to good yields for substrates substituted with a wide variety of functional groups that can be further manipulated by traditional organic chemistry methodologies or organometallic cross-coupling transformations. Of interest, the preferential functionalization at the nonsubstituted arene ring expands substantially chemical space, providing products that could be orthogonally manipulated in each of the two rings. While yields may be considered still far from satisfactory, the reaction represents a single step path toward these valuable complex molecules from simple naphthalenes.

### Mechanistic Studies

**Figure 5:**
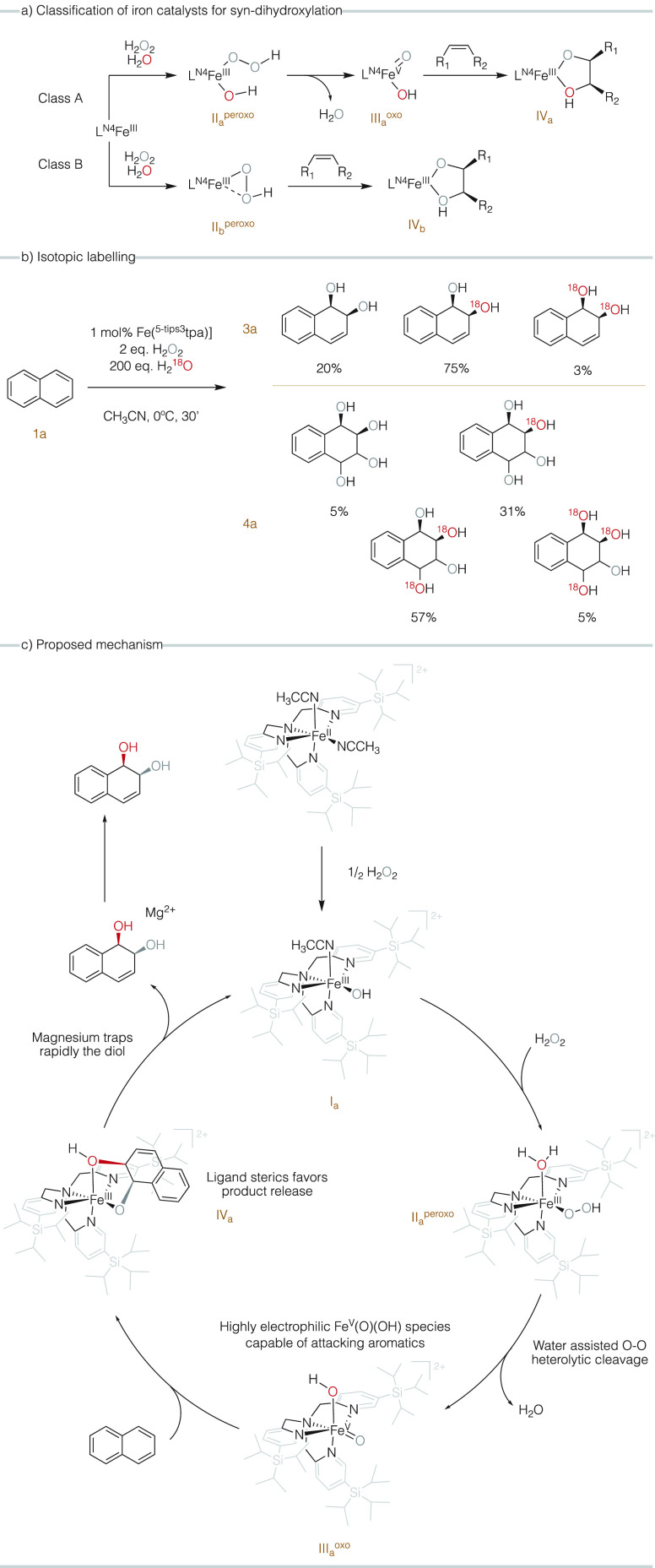
(a) Classification based on the mechanism for iron *syn*-dihydroxylation catalysts. (b) Isotopic labeling results for diol **3a** and tetraol **3b** formation. (c) Proposed mechanism for iron-catalyzed *syn*-dihydroxylation of naphthalene. Note that the specific position of the ^18^O is not determined.

Insight into the reaction mechanism was obtained using isotopic labeling experiments. It is known that iron catalysts can *syn*-dihydroxylate olefins through two different mechanisms (Figure 5a).^31,32,61,62^ Class A catalysts contain strong field N-rich tetradentate ligands (L^N4^) and form [L^N4^Fe^V^(O)(OH)]^2+^ species **III**_**a**_^**oxo**^ (where subindex a refers to class A catalyst) that *syn*-dihydroxylate olefins via a 3 + 2 mechanism (formal cycloaddition between the Fe^V^(O)(OH) and the olefin). **III**_**a**_^**oxo**^ is formed via heterolytic cleavage of the O–O bond of the [L^N4^Fe^III^(OOH)(H_2_O)]^2+^ precursor (**II**_**a**_^**peroxo**^). This is called the water assisted mechanism.^31^ As a result of this O–O cleavage mechanism, species **III**_**a**_^**oxo**^ contains an oxygen atom originating from the peroxide and a second oxygen atom from the water molecule, which are transferred to the olefin. On the other hand, class B catalysts form diols with both oxygens inserted coming from a single hydrogen peroxide molecule (Figure 5a), presumably via a side-on bound hydroperoxide [L^N4^Fe^III^(η^2^-OOH)]^2+^, **II**_**b**_^**peroxo**^ (where subindex b refers to class B catalyst).^40,62^ With this consideration in mind, we performed the oxidation of naphthalene using H_2_^16^O_2_ (2 equiv) in the presence of H_2_^18^O (162 equiv). GC–MS analysis shows that in the case of diol **2a** (Figure 5b), the major isotopomer (75%) contains a ^16^O^18^O composition (the exact position of the ^18^O and ^16^O atoms could not be determined), which indicates that the reaction proceeds via **III**_**a**_^**oxo**^ species, in a class A mechanism. The isotopic pattern derived from this analysis is the same as in the case of the dihydroxylation of olefins with the **[Fe(**^**5-tips3**^**tpa)]** catalyst.^50^ Therefore, we conclude that the *cis*-dihydroxylation of alkenes and arenes is performed by the same **III**_**a**_^**oxo**^ species. These have been previously characterized in the gas phase,^53^ but characterization in solution has not been possible so far because conditions that enable their accumulation have not been identified. An experiment was performed under nitrogen and in an anhydrous solvent to investigate if the nonlabeled oxygen introduced in the molecule could originate from the oxygen present in the air, but no significant changes in product yields and product ratios were observed (see Supporting Information, Table S13), strongly suggesting that external O_2_ does not participate in the reaction. Taking into consideration the isotopic pattern determined for the diol (Figure 5b), we calculated the expected labeling pattern that should be obtained for the tetraol formation assuming that the second dihydroxylation is performed by the same **III**_**a**_^**oxo**^ species (see Supporting Information, entry 2, Table S25). Notably, the calculated isotopic pattern reasonably matches the experimental data (see Supporting Information, entries 3 and 4, Table S25). Moreover, the two isomers of the tetraol have the same isotopic patterns, which strongly suggest that both of them are formed via the same mechanism.

Consequently, we infer that the catalysts behave as previously proposed for the *syn*-dihydroxylation of olefins^50^ and the high electrophilicity of **III**_**a**_^**oxo**^ accounts for the chemoselective arene dihydroxylating ability.^53^ In addition, as it has been previously argued, the bulkiness of this complex may help the release of the diol from the metal center through steric effects, and the diol is rapidly trapped by the magnesium cation so that the catalytic center is regenerated and its catalytic lifetime is extended since the diol does not remain chelated in the iron center. Subsequent dihydroxylation of the olefinic site in the first formed diol is facilitated by the lack of aromaticity but it may be also slowed down because of polar deactivation caused by the binding of the diol moiety to the Mg^2+^ Lewis acid. An intriguing aspect is the origin of the preferential *syn*-diastereoselectivity of the second dihydroxylation reaction. Direct binding of diol to the iron center is unlikely since **III**_**a**_^**oxo**^ is coordinatively saturated. Besides, the ratio is dependent in a moderate manner on the nature of the substituents in the substrate, the catalyst, and the trapping Lewis acid. We note that analogous modest changes in diastereoselectivity are observed when the oxidation of 2-cyclohexenol is performed in the presence and absence of Mg(ClO_4_)_2_·6H_2_O (Table S21). Clarification of this aspect will require further studies, presumably by characterizing the nature of the Lewis acid–diol complexes.

## Conclusions

Herein we describe the *syn*-dihydroxylation of a broad range of naphthalene derivatives with an iron catalyst that mimics the reaction of NDO’s. The high reactivity of this catalyst is presumably rooted in the high electrophilicity of a *cis*-Fe^V^O(OH) intermediate, which permits us to overcome the reactivity inertia posed by aromaticity and may account for the unusual *syn*-dihydroxylation chemoselectivity among known naphthalene oxidizing reagents. Once this first oxidation takes place, a second olefinic site is rendered reactive for a second dihydroxylation step that proceeds smoothly to deliver tetrahydroxylated products in moderate to good yields. The substrate scope of the reaction is relatively broad, providing access to a variety of densely functionalized products. On notice, in the case of functionalized naphthalenes, oxidation takes place at the nonfunctionalized arene moiety, and thus, the resulting products contain reactive handles that can be chemically manipulated in an orthogonal manner. While product yields and diastereoselectivities of the reactions have obvious room for improvement, the reaction is valuable because it is a single step conversion of readily available arene substrates into valuable sp^3^-rich products that represent an expansion of chemical space or are currently only accessible via multistep sequences or whole cell enzymatic methods. The mild reaction conditions, the use of an iron catalyst, and hydrogen peroxide as an oxidant make the reaction also interesting from a sustainability perspective. But arguably the most interesting aspect is the finding that well-defined iron coordination complexes can perform chemoselective oxidative dearomatization reactions akin to those obtained in enzymatic systems and that are not possible for other transition metal catalysts or oxidizing agents. We envision that catalyst design may enable improvement of the current limitations in terms of selectivity and yields, introduce stereoselectivity, and expand the reaction toward benzenes, where aromaticity represents an even greater challenge.

## Supporting Information Available

Materials and methods describing preparation of complexes and substrates, characterization and experimental procedures for the catalytic reactions, NMR spectra, and GC traces (PDF)

## References

[ref1] Lovering F.; Bikker J.; Humblet C. Escape from Flatland: Increasing Saturation as an Approach to Improving Clinical Success. J. Med. Chem. 2009, 52, 6752–6756. 10.1021/jm901241e. 19827778

[ref2] Shu-Li Y. Asymmetric Dearomatization Reactions; Wiley-VCH, 2016.

[ref3] Wertjes W. C.; Southgate E. H.; Sarlah D. Recent advances in chemical dearomatization of nonactivated arenes. Chem. Soc. Rev. 2018, 47, 7996–8017. 10.1039/C8CS00389K. 30073226

[ref4] Mykhailiuk P. K. Saturated bioisosteres of benzene: where to go next?. Org. Biomol. Chem. 2019, 17, 2839–2849. 10.1039/C8OB02812E. 30672560

[ref5] Subbaiah M. A. M.; Meanwell N. A. Bioisosteres of the Phenyl Ring: Recent Strategic Applications in Lead Optimization and Drug Design. J. Med. Chem. 2021, 64, 14046–14128. 10.1021/acs.jmedchem.1c01215. 34591488

[ref6] Huck C. J.; Boyko Y. D.; Sarlah D. Dearomative logic in natural product total synthesis. Nat. Prod. Rep. 2022, 39, 2231–2291. 10.1039/D2NP00042C. 36173020 PMC9772301

[ref7] Hudlicky T.; Reed J. W. Applications of biotransformations and biocatalysis to complexity generation in organic synthesis. Chem. Soc. Rev. 2009, 38, 3117–3132. 10.1039/b901172m. 19847346

[ref8] Hudlicky T. Benefits of Unconventional Methods in the Total Synthesis of Natural Products. ACS Omega 2018, 3, 17326–17340. 10.1021/acsomega.8b02994. 30613812 PMC6312638

[ref9] Ishikawa K.; Charles H. C.; Griffin G. W. Direct peracid oxidation of polynuclear hydrocarbons to arene oxides. Tetrahedron Lett. 1977, 18, 427–430. 10.1016/S0040-4039(01)92656-5.

[ref10] Mello R.; Ciminale F.; Fiorentino M.; Fusco C.; Prencipe T.; Curci R. Oxidations by methyl(trifluoromethyl)dioxirane. 4.1 oxyfunctionalization of aromatic hydrocarbons. Tetrahedron Lett. 1990, 31, 6097–6100. 10.1016/S0040-4039(00)98039-0.

[ref11] Motherwell W. B.; Williams A. S. Catalytic Photoinduced Charge-Transfer Osmylation: A Novel Pathway from Arenes to Cyclitol Derivatives. Angew. Chem., Int. Ed. Engl. 1995, 34, 2031–2033. 10.1002/anie.199520311.

[ref12] Jung P. M. J.; Motherwell W. B.; Williams A. S. Stereochemical observations on the bromate induced monobromopentahydroxylation of benzene by catalytic photoinduced charge transfer osmylation. A concise synthesis of (±)-pinitol. Chem. Commun. 1997, 1283–1284. 10.1039/a703071a.

[ref13] Koppaka A.; Kirkland J. K.; Periana R. A.; Ess D. H. Experimental Demonstration and Density Functional Theory Mechanistic Analysis of Arene C–H Bond Oxidation and Product Protection by Osmium Tetroxide in a Strongly Basic/Nucleophilic Solvent. J. Org. Chem. 2022, 87, 13573–13582. 10.1021/acs.joc.2c01159. 36191170

[ref14] Song R.; Sorokin A.; Bernadou J.; Meunier B. Metalloporphyrin-Catalyzed Oxidation of 2-Methylnaphthalene to Vitamin K3 and 6-Methyl-1,4-naphthoquinone by Potassium Monopersulfate in Aqueous Solution. J. Org. Chem. 1997, 62, 673–678. 10.1021/jo961421v. 11671463

[ref15] Khavasi H. R.; Hosseiny Davarani S. S.; Safari N. Remarkable solvent effect on the yield and specificity of oxidation of naphthalene catalyzed by iron(III)porphyrins. J. Mol. Catal. A: Chem. 2002, 188, 115–122. 10.1016/S1381-1169(02)00354-0.

[ref16] Möller K.; Wienhöfer G.; Schröder K.; Join B.; Junge K.; Beller M. Selective Iron-Catalyzed Oxidation of Phenols and Arenes with Hydrogen Peroxide: Synthesis of Vitamin E Intermediates and Vitamin K3. Chem.—Eur. J. 2010, 16, 10300–10303. 10.1002/chem.201001429. 20661966

[ref17] Liu P.; Liu Y.; Wong E. L.-M.; Xiang S.; Che C.-M. Iron oligopyridine complexes as efficient catalysts for practical oxidation of arenes, alkanes, tertiary amines and N-acyl cyclic amines with Oxone. Chem. Sci. 2011, 2, 2187–2195. 10.1039/c1sc00234a.

[ref18] Rebelo S. L. H.; Silva A. M. N.; Medforth C. J.; Freire C. Iron(III) Fluorinated Porphyrins: Greener Chemistry from Synthesis to Oxidative Catalysis Reactions. Molecules 2016, 21, 481 10.3390/molecules21040481. 27077840 PMC6274165

[ref19] Jeong D.; Yan J. J.; Noh H.; Hedman B.; Hodgson K. O.; Solomon E. I.; Cho J. Oxidation of Naphthalene with a Manganese(IV) Bis(hydroxo) Complex in the Presence of Acid. Angew. Chem., Int. Ed. 2018, 57, 7764–7768. 10.1002/anie.201802641. PMC6013404 29701293

[ref20] Calvete M. J. F.; Piñeiro M.; Dias L. D.; Pereira M. M. Hydrogen Peroxide and Metalloporphyrins in Oxidation Catalysis: Old Dogs with Some New Tricks. ChemCatChem. 2018, 10, 3615–3635. 10.1002/cctc.201800587.

[ref21] Masferrer-Rius E.; Borrell M.; Lutz M.; Costas M.; Klein Gebbink R. J. M. Aromatic C–H Hydroxylation Reactions with Hydrogen Peroxide Catalyzed by Bulky Manganese Complexes. Adv. Synth. Catal. 2021, 363, 3783–3795. 10.1002/adsc.202001590.

[ref22] Rajeev A.; Balamurugan M.; Sankaralingam M. Rational Design of First-Row Transition Metal Complexes as the Catalysts for Oxidation of Arenes: A Homogeneous Approach. ACS Catal. 2022, 12, 9953–9982. 10.1021/acscatal.2c01928.

[ref23] Szymczak J.; Kryjewski M. Porphyrins and Phthalocyanines on Solid-State Mesoporous Matrices as Catalysts in Oxidation Reactions. Materials 2022, 15, 2532 10.3390/ma15072532. 35407864 PMC8999812

[ref24] Hernandez L. W.; Pospech J.; Klöckner U.; Bingham T. W.; Sarlah D. Synthesis of (+)-Pancratistatins via Catalytic Desymmetrization of Benzene. J. Am. Chem. Soc. 2017, 139, 15656–15659. 10.1021/jacs.7b10351. 29059521 PMC5960067

[ref25] Southgate E. H.; Pospech J.; Fu J.; Holycross D. R.; Sarlah D. Dearomative dihydroxylation with arenophiles. Nat. Chem. 2016, 8, 922–928. 10.1038/nchem.2594. 27657867 PMC5971114

[ref26] Siddiqi Z.; Wertjes W. C.; Sarlah D. Chemical Equivalent of Arene Monooxygenases: Dearomative Synthesis of Arene Oxides and Oxepines. J. Am. Chem. Soc. 2020, 142, 10125–10131. 10.1021/jacs.0c02724. 32383862 PMC7327703

[ref27] Dong J.; Fernández-Fueyo E.; Hollmann F.; Paul C. E.; Pesic M.; Schmidt S.; Wang Y.; Younes S.; Zhang W. Biocatalytic Oxidation Reactions: A Chemist’s Perspective. Angew. Chem., Int. Ed. 2018, 57, 9238–9261. 10.1002/anie.201800343. PMC6099261 29573076

[ref28] Karlsson A.; Parales J. V.; Parales R. E.; Gibson D. T.; Eklund H.; Ramaswamy S. Crystal Structure of Naphthalene Dioxygenase: Side-on Binding of Dioxygen to Iron. Science 2003, 299, 1039–1042. 10.1126/science.1078020. 12586937

[ref29] Barry S. M.; Challis G. L. Mechanism and Catalytic Diversity of Rieske Non-Heme Iron-Dependent Oxygenases. ACS Catal. 2013, 3, 2362–2370. 10.1021/cs400087p. PMC3827946 24244885

[ref30] Chen K.; Que L. Jr. cis-Dihydroxylation of Olefins by a Non-Heme Iron Catalyst: A Functional Model for Rieske Dioxygenases. Angew. Chem., Int. Ed. 1999, 38, 2227–2229. 10.1002/(SICI)1521-3773(19990802)38:15<2227::AID-ANIE2227>3.0.CO;2-B. 10425491

[ref31] Chen K.; Costas M.; Kim J.; Tipton A. K.; Que L. Olefin Cis-Dihydroxylation versus Epoxidation by Non-Heme Iron Catalysts: Two Faces of an FeIII–OOH Coin. J. Am. Chem. Soc. 2002, 124, 3026–3035. 10.1021/ja0120025. 11902894

[ref32] Fujita M.; Costas M.; Que L. Iron-Catalyzed Olefin cis-Dihydroxylation by H2O2: Electrophilic versus Nucleophilic Mechanisms. J. Am. Chem. Soc. 2003, 125, 9912–9913. 10.1021/ja029863d. 12914440

[ref33] Bukowski M. R.; Comba P.; Lienke A.; Limberg C.; Lopez de Laorden C.; Mas-Ballesté R.; Merz M.; Que L. Jr. Catalytic Epoxidation and 1,2-Dihydroxylation of Olefins with Bispidine–Iron(II)/H2O2 Systems. Angew. Chem., Int. Ed. 2006, 45, 3446–3449. 10.1002/anie.200504357. 16637091

[ref34] Bautz J.; Comba P.; Lopez de Laorden C.; Menzel M.; Rajaraman G. Biomimetic High-Valent Non-Heme Iron Oxidants for the cis-Dihydroxylation and Epoxidation of Olefins. Angew. Chem., Int. Ed. 2007, 46, 8067–8070. 10.1002/anie.200701681. 17868164

[ref35] Bruijnincx P. C. A.; Buurmans I. L. C.; Gosiewska S.; Moelands M. A. H.; Lutz M.; Spek A. L.; van Koten G.; Klein Gebbink R. J. M. Iron(II) Complexes with Bio-Inspired N,N,O Ligands as Oxidation Catalysts: Olefin Epoxidation and cis-Dihydroxylation. Chem.—Eur. J. 2008, 14, 1228–1237. 10.1002/chem.200700573. 18022966

[ref36] Oldenburg P. D.; Feng Y.; Pryjomska-Ray I.; Ness D.; Que L. Jr. Olefin Cis-Dihydroxylation with Bio-Inspired Iron Catalysts. Evidence for an FeII/FeIV Catalytic Cycle. J. Am. Chem. Soc. 2010, 132, 17713–17723. 10.1021/ja1021014. 21105649

[ref37] Chow T. W.-S.; Wong E. L.-M.; Guo Z.; Liu Y.; Huang J.-S.; Che C.-M. cis-Dihydroxylation of Alkenes with Oxone Catalyzed by Iron Complexes of a Macrocyclic Tetraaza Ligand and Reaction Mechanism by ESI-MS Spectrometry and DFT Calculations. J. Am. Chem. Soc. 2010, 132, 13229–13239. 10.1021/ja100967g. 20812697

[ref38] Feng Y.; England J.; Que L. Jr. Iron-Catalyzed Olefin Epoxidation and cis-Dihydroxylation by Tetraalkylcyclam Complexes: the Importance of cis-Labile Sites. ACS Catal. 2011, 1, 1035–1042. 10.1021/cs200292h.

[ref39] Chatterjee S.; Paine T. K. Olefin cis-Dihydroxylation and Aliphatic C-H Bond Oxygenation by a Dioxygen-Derived Electrophilic Iron–Oxygen Oxidant. Angew. Chem., Int. Ed. 2015, 54, 9338–9342. 10.1002/anie.201502229. 26088714

[ref40] Zang C.; Liu Y.; Xu Z.-J.; Tse C.-W.; Guan X.; Wei J.; Huang J.-S.; Che C.-M. Highly Enantioselective Iron-Catalyzed cis-Dihydroxylation of Alkenes with Hydrogen Peroxide Oxidant via an FeIII-OOH Reactive Intermediate. Angew. Chem., Int. Ed. 2016, 55, 10253–10257. 10.1002/anie.201603410. 27457506

[ref41] Wei J.; Wu L.; Wang H.-X.; Zhang X.; Tse C.-W.; Zhou C.-Y.; Huang J.-S.; Che C.-M. Iron-Catalyzed Highly Enantioselective cis-Dihydroxylation of Trisubstituted Alkenes with Aqueous H2O2. Angew. Chem., Int. Ed. 2020, 59, 16561–16571. 10.1002/anie.202002866. 32500643

[ref42] Chen J.; Luo X.; Sun Y.; Si S.; Xu Y.; Lee Y.-M.; Nam W.; Wang B. Nonheme Iron-Catalyzed Enantioselective cis-Dihydroxylation of Aliphatic Acrylates as Mimics of Rieske Dioxygenases. CCS Chem. 2022, 4, 2369–2381. 10.31635/ccschem.022.202201780.

[ref43] Brinksma J.; Schmieder L.; van Vliet G.; Boaron R.; Hage R.; De Vos D. E.; Alsters P. L.; Feringa B. L. Homogeneous cis-dihydroxylation and epoxidation of olefins with high H2O2 efficiency by mixed manganese/activated carbonyl catalyst system. Tetrahedron Lett. 2002, 43, 2619–2622. 10.1016/S0040-4039(02)00292-7.

[ref44] de Boer J. W.; Brinksma J.; Browne W. R.; Meetsma A.; Alsters P. L.; Hage R.; Feringa B. L. cis-Dihydroxylation and Epoxidation of Alkenes by [Mn2O(RCO2)2(tmtacn)2]: Tailoring the Selectivity of a Highly H2O2-Efficient Catalyst. J. Am. Chem. Soc. 2005, 127, 7990–7991. 10.1021/ja050990u. 15926804

[ref45] de Boer J. W.; Browne W. R.; Brinksma J.; Alsters P. L.; Hage R.; Feringa B. L. Mechanism of Cis-Dihydroxylation and Epoxidation of Alkenes by Highly H2O2 Efficient Dinuclear Manganese Catalysts. Inorg. Chem. 2007, 46, 6353–6372. 10.1021/ic7003613. 17608415

[ref46] de Boer J. W.; Browne W. R.; Harutyunyan S. R.; Bini L.; Tiemersma-Wegman T. D.; Alsters P. L.; Hage R.; Feringa B. L. Manganese catalysed asymmetric cis-dihydroxylation with H2O2. Chem. Commun. 2008, 3747–3749. 10.1039/b808355j. 18685764

[ref47] Saisaha P.; Pijper D.; van Summeren R. P.; Hoen R.; Smit C.; de Boer J. W.; Hage R.; Alsters P. L.; Feringa B. L.; Browne W. R. Manganese catalyzed cis-dihydroxylation of electron deficient alkenes with H2O2. Org. Biomol. Chem. 2010, 8, 4444–4450. 10.1039/c0ob00102c. 20714666

[ref48] Chow T. W.-S.; Liu Y.; Che C.-M. Practical manganese-catalysed highly enantioselective cis-dihydroxylation of electron-deficient alkenes and detection of a cis-dioxomanganese(v) intermediate by high resolution ESI-MS analysis. Chem. Commun. 2011, 47, 11204–11206. 10.1039/c1cc11999k. 21614373

[ref49] Feng Y.; Ke C.-y.; Xue G.; Que L. Bio-inspired arenecis-dihydroxylation by a non-haem iron catalyst modeling the action of naphthalene dioxygenase. Chem. Commun. 2008, 52, 50 10.1039/B817222F. 19081995

[ref50] Borrell M.; Costas M. Mechanistically Driven Development of an Iron Catalyst for Selective Syn-Dihydroxylation of Alkenes with Aqueous Hydrogen Peroxide. J. Am. Chem. Soc. 2017, 139, 12821–12829. 10.1021/jacs.7b07909. 28767230

[ref51] Roy L. Theoretical Insights into the Nature of Oxidant and Mechanism in the Regioselective Syn-dihydroxylation of an Alkene with a Rieske oxygenase inspired Iron Catalyst. ChemCatChem. 2018, 10, 3683–3688. 10.1002/cctc.201800799.

[ref52] Borrell M.; Costas M. Greening Oxidation Catalysis: Iron Catalyzed Alkene syn-Dihydroxylation with Aqueous Hydrogen Peroxide in Green Solvents. ACS Sustain. Chem. Eng. 2018, 6, 8410–8416. 10.1021/acssuschemeng.8b00542.

[ref53] Borrell M.; Andris E.; Navrátil R.; Roithová J.; Costas M. Characterized cis-FeV(O)(OH) intermediate mimics enzymatic oxidations in the gas phase. Nat. Commun. 2019, 10, 901 10.1038/s41467-019-08668-2. 30796210 PMC6385299

[ref54] Shen T.; Li Y.-L.; Ye K.-Y.; Lambert T. H. Electrophotocatalytic oxygenation of multiple adjacent C–H bonds. Nature 2023, 614, 275–280. 10.1038/s41586-022-05608-x. 36473497 PMC10436356

[ref55] Chen M. S.; White M. C. Combined Effects on Selectivity in Fe-Catalyzed Methylene Oxidation. Science 2010, 327, 566–571. 10.1126/science.1183602. 20110502

[ref56] Cussó O.; Garcia-Bosch I.; Ribas X.; Lloret-Fillol J.; Costas M. Asymmetric Epoxidation with H2O2 by Manipulating the Electronic Properties of Non-heme Iron Catalysts. J. Am. Chem. Soc. 2013, 135, 14871–14878. 10.1021/ja4078446. 24060452

[ref57] Costas M.; Tipton A. K.; Chen K.; Jo D.-H.; Que L. Modeling Rieske Dioxygenases: The First Example of Iron-Catalyzed Asymmetric cis-Dihydroxylation of Olefins. J. Am. Chem. Soc. 2001, 123, 6722–6723. 10.1021/ja015601k. 11439071

[ref58] Suzuki K.; Oldenburg P. D.; Que L. Jr Iron-Catalyzed Asymmetric Olefin cis-Dihydroxylation with 97% Enantiomeric Excess. Angew. Chem., Int. Ed. 2008, 47, 1887–1889. 10.1002/anie.200705061. 18236485

[ref59] Company A.; Gómez L.; Fontrodona X.; Ribas X.; Costas M. A Novel Platform for Modeling Oxidative Catalysis in Non-Heme Iron Oxygenases with Unprecedented Efficiency. Chem.—Eur. J. 2008, 14, 5727–5731. 10.1002/chem.200800724. 18481345

[ref60] Prat I.; Font D.; Company A.; Junge K.; Ribas X.; Beller M.; Costas M. Fe(PyTACN)-Catalyzed cis-Dihydroxylation of Olefins with Hydrogen Peroxide. Adv. Synth. Catal. 2013, 355, 947–956. 10.1002/adsc.201200938.

[ref61] Chen K.; Costas M.; Que L. Jr. Spin state tuning of non-heme iron-catalyzed hydrocarbon oxidations: participation of FeIII–OOH and FeV-O intermediates. J. Chem. Soc., Dalton Trans. 2002, 672–679. 10.1039/b108629d.

[ref62] Zhu W.; Kumar A.; Xiong J.; Abernathy M. J.; Li X.-X.; Seo M. S.; Lee Y.-M.; Sarangi R.; Guo Y.; Nam W. Seeing the cis-Dihydroxylating Intermediate: A Mononuclear Nonheme Iron-Peroxo Complex in cis-Dihydroxylation Reactions Modeling Rieske Dioxygenases. J. Am. Chem. Soc. 2023, 145, 4389–4393. 10.1021/jacs.2c13551. 36795537 PMC10544271

