# Supplementary material for: Dearomative syn-Dihydroxylation of Naphthalenes with a Biomimetic Iron Catalyst
Source: J Am Chem Soc. 2023 Dec 20;146(1):240–9. doi: 10.1021/jacs.3c08565 (PMC10785824; doi:10.1021/jacs.3c08565)
Supplement: Supplementary file 1 — ja3c08565_si_001.pdf [file ja3c08565_si_001.pdf]

Supporting information for

Dearomative *syn*-dihydroxylation of naphthalenes with a biomimetic iron catalyst

Najoua Choukairi Afailal, Margarida Borrell, Marco Cianfanelli, Miquel Costas*

Institut de Química Computacional i Catàlisi (IQCC) and Departament de Química, Universitat de Girona, Campus Montilivi, Girona E-17071, Catalonia, Spain.

e-mail: miquel.costas@udg.edu

1. Experimental section S3
   1.1. Materials S3
   1.2. Instrumentation S3

2. Synthesis of the substrates S4
   2.1. Characterization of the substrates S4

3. Synthesis and characterization of ligands S7
   3.1. Synthesis and characterization of synthons S7
   3.2. Ligand assembly and characterization S13

4. Synthesis and characterization of complexes S15

5. Reaction conditions for *syn*-dihydroxylation of arenes S18
   5.1. Experimental conditions for maximizing diol (2x) formation S18
   5.2. Experimental conditions for maximizing tetraol (3x) formation S18
   5.3. Workup of the catalysis S18

6. Optimization S19

7. Reaction monitoring S30
   7.1. Product formation S30
   7.1. HRMS monitoring of a catalytic reaction. S33
   7.2. Experimental and predicted HRMS of the detected compounds S36

8. General procedure for isolation of products S39
   8.1. Diol formation S39
   8.2. Tetraol formation S39
   8.3. Workup for the isolation of products S39

9. Characterization of isolated products S40

10. O^{18} labelling experiments S70

11. GC chromatograms S74

12. NMR spectra of the isolated compounds S81

13. References S242

1. Experimental section

1.1. Materials

Reagents, substrates, and solvents used were of commercially available reagent quality unless stated otherwise. Solvents were purchased from SDS, Aldrich, Fluorochem and Scharlab. Solvents were purified and dried by passing through an activated alumina purification system (M-Braun SPS - 800) or by conventional distillation techniques. All liquid substrates were passed through a silica, a basic alumina and a celite plug before being used.

1.2. Instrumentation

Oxidation products were identified GC/MS and by ^{1}H and $^{13}C\{^{1}H\}$-NMR analyses.
NMR experiments were recorded on a Bruker 400 MHz Avance III HD spectrometer equipped with a 5mm BBOF probe. NMR spectra were performed with standard experiments provided in the Bruker release. Pulse sequences used were zg30 and zgpr in ^{1}H spectra, cosygpqf and cosygpprqf in COSY spectra, hsqcedetgpsisp2.3_bbhd in ^{1}H-^{13}C HSQC spectra implemented with carbon multiplicity editing and proton broadband homodecoupling during acquisition, zgpg30 in $^{13}C\{^{1}H\}$ and deptsp135 in $^{13}C\{^{1}H\}$ DEPT135 spectra. Spectra were referenced to the residual proto solvents peaks or TMS (tetramethylsilane) for ^{1}H. GS-MS spectral analyses were performed on an Agilent 7890A gas chromatograph interfaced with an Agilent 5975c mass spectrometer with a triple-axis detector. GC analyses were carried out on an AgilentGC-7820-A gas chromatograph (HP5 column, 30m) with a flame ionization detector. High resolution mass spectrometry (HRMS) were performed on Bruker MicrOTOF-Q II (Q-TOF) instrument with a quadrupole analyzer with positive and negative ionization mode using methanol as mobile phase and at Serveis Tècnics of the University of Girona. IR spectra were taken in a Mattson- Galaxy Satellite FT-IR spectrophotometer using a MKII Golden Gate single reflection ATR system. Elemental analyses were performed using a CHNS-O EA-1108 elemental analyzer from Fisons. X-Ray diffraction analysis were carried out on a BRUKER SMART APEX CCD diffractometer using graphite-monochromated MoKα radiation (λ = 0.71073 Å) from an X-ray Tube.

2. Synthesis of the substrates

Substrates **1h** and **1i** were prepared as in the reported procedures, characteristic data agreed with the reported ones.[1, 2]

Substrates **1f** and **1d** were synthesized according to the following procedure. In a 20 mL vial, 0.4 g of 2-naphtol or 2,3-dihydroxynaphtalene was added followed by 3 mL of methylimidazole and 5 mL of acetic anhydride. The mixture was stirred at room temperature for 2 h. After this, crushed ice was added, and the product was extracted with dichloromethane. The organic phase was washed with H_2SO_4 1M, $NaHCO_3$ sat., and subsequently cleaned with water, finally it was dried with $MgSO_4$ and filtered through a plug of basic alumina. The solvent was removed under vacuum and the substrates were obtained with 60-70% yield as white solids and were used without further purification.

2.1. Characterization of the substrates

Naphthalen-2-yl acetate (1f)

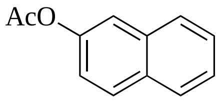

^{1}H NMR (400 MHz, Chloroform-*d*) δ 7.91-7.81 (m, 3H), 7.58 (d, *J* = 2.3 Hz, 1H), 7.50 (dtd, *J* = 7.9, 6.9, 5.3 Hz, 2H), 7.26 (dd, *J* = 8.9, 2.3 Hz, 1H), 2.38 (s, 3H).

^{13}C NMR (101 MHz, Chloroform-*d*) δ 169.80, 148.46, 133.90, 131.61, 129.56, 127.91, 127.79, 126.70, 125.85, 121.27, 118.67, 21.35.

HRMS(ESI+) m/z calculated $[M+Na]^+$ 209.0573, found 209.0574.

Naphthalene-2,3-diyl diacetate (1d)

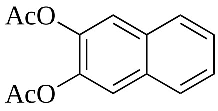

^{1}H NMR (400 MHz, Chloroform-*d*) δ 7.82 (dd, *J* = 6.2, 3.3 Hz, 2H), 7.68 (s, 2H), 7.50 (dd, *J* = 6.3, 3.2 Hz, 2H), 2.37 (s, 6H).

^{13}C NMR (101 MHz, Chloroform-*d*) δ 168.69, 141.06, 131.71, 127.62, 126.51, 121.06, 20.84.

HRMS(ESI+) m/z calculated $[M+Na]^+$ 267.0628, found 267.0627.

2-nitronaphthalene (1i)

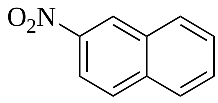

^{1}H NMR (400 MHz, Chloroform-*d*) δ 8.82 (d, *J* = 2.3 Hz, 1H), 8.25 (dd, *J* = 9.0, 2.3 Hz, 1H), 8.07 – 8.03 (m, 1H), 7.99-7.93 (m, 2H), 7.68 (dddd, *J* = 21.2, 8.2, 6.9, 1.3 Hz, 2H).

^{13}C NMR (101 MHz, Chloroform-*d*) δ 129.95, 129.75, 129.49, 127.98, 127.92, 124.56, 119.17.

GC-MS(CI-NH_4^+) m/z [M] 173.0, [M+NH_4^+] 191.0.

2-phenylnaphthalene (1h)

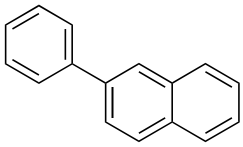

Obtained as a white crystalline solid in 73%yield.

^{1}H NMR (400 MHz, Chloroform-*d*) δ 8.07 (dd, *J* = 1.8, 0.8 Hz, 1H), 7.96-7.87 (m, 3H), 7.80-7.73 (m, 3H), 7.56-7.48 (m, 4H), 7.43-7.38 (m, 1H).

^{13}C NMR (101 MHz, Chloroform-*d*) δ 141.30, 138.74, 133.91, 129.06, 128.64, 127.86, 127.63, 127.55, 126.48, 126.13, 126.00, 125.79.

GC-MS(CI-NH_4^+) m/z [M+NH_4^+] 222.0.

3. Synthesis and characterization of ligands

3.1. Synthesis and characterization of synthons

- **(4-(dimethylamino)pyridin-2-yl)methanol (I)**

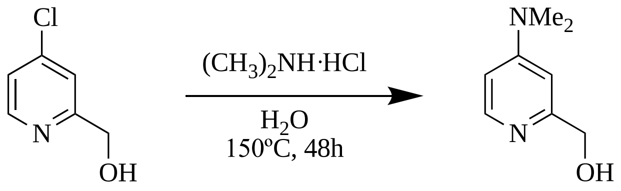

Obtained by small modifications of a reported procedure.[3] In a schlenck tube 2.3g of (4-chloropyridin-2-yl)methanol (16.1 mmol), dimethylamine hydrochloride (6.5 g, 79.7 mmol, 5 eq.) and sodium hydroxide (3g, 75 mmol, 4.7 eq.) were dissolved in 10 mL of water. The vial was closed and the reaction was left stirring under reduced pressure at 150ºC for 48h. When the reaction was finished it was left to cool down and the cap of the vial was slowly removed. An aqueous solution of sodium hydroxide (2M) and dichloromethane were added and the aqueous phase was extracted 2 times with dichloromethane. The organic phase was dried with $MgSO_4$ and the solvent was removed under vacuum to yield 3.28g of (4-(dimethylamino)pyridin-2-yl)methanol (I, 67% yield) as a pale brown solid which was used without further purification.

^{1}H NMR (400 MHz, Chloroform-*d*) δ 8.20-8.13 (m, 1H), 6.42 (d, *J* = 5.1 Hz, 2H), 4.63 (s, 2H), 3.00 (s, 6H).

^{13}C NMR (101 MHz, Chloroform-*d*) δ 159.35, 155.02, 148.63, 105.88, 102.69, 64.62, 39.32.

ESI-MS m/z calculated $[M+H]^+$ 153.1, found 153.0.

- **2-(((tert-butyldimethylsilyl)oxy)methyl)-N,N-dimethylpyridin-4-amine (II)**

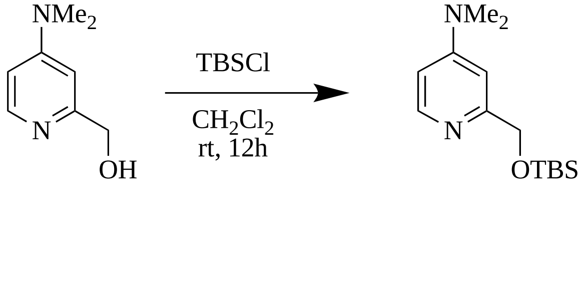

In a round bottom flask, (4-(dimethylamino)pyridin-2-yl)methanol (I) (7.42 g, 48.8 mmol) was dissolved in dichloromethane (150 mL). To the solution, it was added TBSCl (8.83 g, 1.2 eq.), imidazole (5 g, 1.5 eq.) and DMAP (0.6 g, 0.1 eq.). The mixture was left stirring 12h at room temperature, later water was added, and the organic phase was extracted and dried with $MgSO_4$. The solvent was removed under reduced pressure and the crude was purified by flash chromatography silica gel (Hexane : Ethyl acetate, 9:1, and when the first spot of the TLC came out, the eluent changed to ethyl acetate) to obtain 9.6g of 2-(((*tert*-butyldimethylsilyl)oxy)methyl)-*N*,*N*-dimethylpyridin-4-amine (II) as a pale yellowish solid (74% yield).

^{1}H NMR (400 MHz, Chloroform-*d*) δ 8.13 (d, J = 5.9 Hz, 1H), 6.78 (d, J = 2.7 Hz, 1H), 6.35 (dd, J = 5.9, 2.7 Hz, 1H), 4.74 (d, J = 0.8 Hz, 2H), 3.01 (s, 6H), 0.97 (s, 9H), 0.12 (s, 6H).

^{13}C NMR (101 MHz, Chloroform-*d*) δ 161.43, 155.24, 149.08, 104.95, 102.56, 66.24, 39.24, 26.08, 18.51, -5.17.

ESI-MS m/z calculated $[M+H]^+$ 267.2, found 267.1.

- **5-bromo-2-(((*tert*-butyldimethylsilyl)oxy)methyl)-*N*,*N*-dimethylpyridin-4-amine (III)**

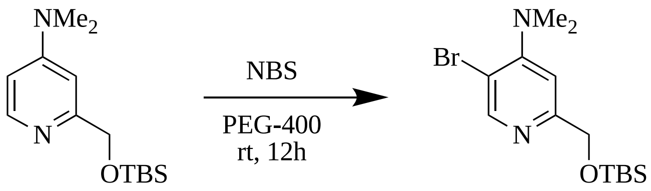

Prepared by small modification of the reported procedure.[4] In a 30 mL vial 266 mg of 2-(((*tert*-butyldimethylsilyl)oxy)methyl)-*N*,*N*-dimethylpyridin-4-amine (II, 1 mmol), 2g of PEG-400 and 187 mg of NBS (1 eq. 1 mmol) were added and left stirring overnight at room temperature. (Important to put maximum 2 grams of PEG-400 because it's very dense and a major amount could affect the yield. In case more amount is needed, put in parallel vials the same amount of product and at the end of the reaction join them to purify.) After this the solution was directly packed and purified by flash chromatography silica gel (Hexane:Ethyl acetate, 9:1) to yield 279 mg (81% yield) of 5-bromo-2-(((*tert*-

butyldimethylsilyl)oxy)methyl)-*N*,*N*-dimethylpyridin-4-amine (III) as a yellow oil that in the freezer becomes a solid.

^{1}H NMR (400 MHz, Chloroform-*d*) δ 8.38 (s, 1H), 7.07 (d, *J* = 1.0 Hz, 1H), 4.71 (d, *J* = 0.9 Hz, 2H), 2.96 (s, 6H), 0.96 (s, 9H), 0.12 (s, 6H).

^{13}C NMR (101 MHz, Chloroform-*d*) δ 161.08, 157.76, 152.43, 110.36, 110.22, 65.63, 42.67, 26.03, 18.45, -5.20.

HRMS(ESI+) m/z calculated $[M+H]^+$ 345.0992, found 345.0984.

- **2-(((*tert*-butyldimethylsilyl)oxy)methyl)-*N*,*N*-dimethyl-5-(triisopropylsilyl)pyridin-4-amine (IV)**

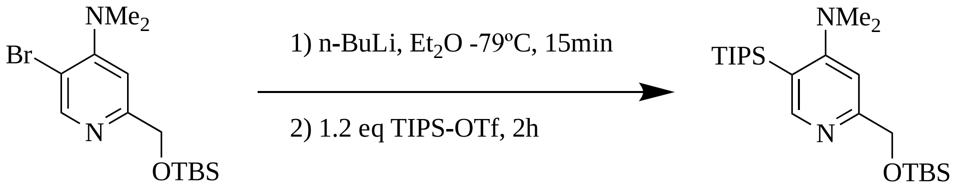

Carefully dried 5-bromo-2-(((*tert*-butyldimethylsilyl)oxy)methyl)-*N*,*N*-dimethylpyridin-4-amine (III, 5 g, 14.5 mmol) was dissolved in 50 mL of anhydrous diethyl ether. The solution was left at -78ºC and n-BuLi was added dropwise (6.38 mL, 2.5M, 1.1 eq.), the solution was left stirring 10-15 minutes and then triisopropylsilyl trifluoromethanesulfonate (5.85 mL, 21.7 mmol, 1.5 eq.) was slowly added and the solution was left stirring for two hours. After this, the temperature was increased and water was added to quench the reaction. The organic phase was separated and the aqueous phase was extracted with diethyl ether. Finally the organic phases were joined, washed with brine, dried with $MgSO_4$ and the solvent was removed under reduced pressure to obtain an orange oil of 2-(((*tert*-butyldimethylsilyl)oxy)methyl)-*N*,*N*-dimethyl-5-(triisopropylsilyl)pyridin-4-amine (IV) which was used without further purification.

^{1}H NMR (400 MHz, Chloroform-*d*) δ 8.46 (s, 1H), 7.32 (s, 1H), 4.80 (d, *J* = 0.8 Hz, 2H), 2.67 (s, 6H), 1.57-1.46 (m, 3H), 1.10 (d, *J* = 7.5 Hz, 18H), 0.97 (s, 9H), 0.13 (d, *J* = 1.2 Hz, 6H).

^{13}C NMR (101 MHz, Chloroform-*d*) δ 159.35, 155.02, 148.63, 105.88, 102.69, 64.62, 39.32.

HRMS(ESI+) m/z calculated $[M+H]^+$ 423.3221, found 423.3217.

- **(4-(dimethylamino)-5-(triisopropylsilyl)pyridin-2-yl)methanol (V)**

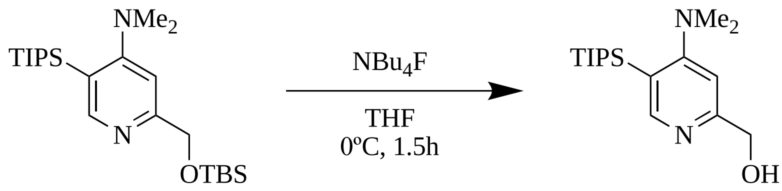

In a vial, the crude of the previous reaction (IV) was dissolved in 13 mL of THF and the mixture was cooled to 0ºC in an ice bath. After this, a solution of NBu_4F in THF (14.5 mL, was added dropwise and the reaction was left stirring for 1.5h at 0ºC. When the reaction was completed, ethyl acetate and water were added, the organic phase was separated and washed with brine, dried with $MgSO_4$ and the solvent was removed under vacuum and purified by flash chromatography silica gel (Hexane : Ethyl acetate, 10:3, and when the first spot of the TLC came out, the eluent was just ethyl acetate) to yield 2.6g (59% yield, two steps) of (4-(dimethylamino)-5-(triisopropylsilyl)pyridin-2-yl)methanol (V) as a yellow oil.

^{1}H NMR (400 MHz, Chloroform-*d*) δ 8.52 (s, 1H), 6.99 (d, *J* = 0.8 Hz, 1H), 4.71 (s, 2H), 2.66 (s, 6H), 1.56-1.44 (m, 4H), 1.11 (d, *J* = 7.5 Hz, 18H).

^{13}C NMR (101 MHz, Chloroform-d) δ 169.70, 160.68, 157.01, 124.95, 112.40, 64.34, 46.00, 19.39, 12.55.

HRMS(ESI+) m/z calculated $[M+H]^+$ 309.2357, found 309.2349.

- **2-(chloromethyl)-*N*,*N*-dimethyl-5-(triisopropylsilyl)pyridin-4-amine (VI)**

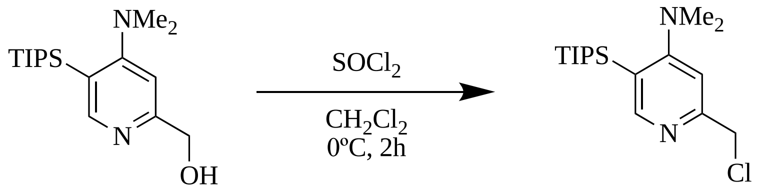

In a round bottom flask, (4-(dimethylamino)-5-(triisopropylsilyl)pyridin-2-yl)methanol (V, 4.2 g, 13.6 mmol) were dissolved in 60 mL of dichloromethane and cooled to 0ºC in an ice bath. After this, $SOCl_2$ (5.1 mL) was added dropwise to the solution and was left stirring for 2.5h at 0ºC. After this saturated $NaHCO_3$ aq. was added dropwise till no

bubbles were formed. The organic phase was extracted, dried with $MgSO_4$ and the solvent was removed under reduced pressure to yield 3.3g (75% yield) of 2-(chloromethyl)-*N*,*N*-dimethyl-5-(triisopropylsilyl)pyridin-4-amine (VI) as a brown oil which was used without further purification.

^{1}H NMR (400 MHz, Chloroform-*d*) δ 8.11 (s, 1H), 7.00 (s, 1H), 5.01 (s, 2H), 3.17 (s, 6H), 1.93 (s, 1H), 1.44 (hept, J = 7.5 Hz, 3H), 1.12 (d, J = 7.5 Hz, 18H).

^{13}C NMR (101 MHz, Chloroform-*d*) δ 167.70, 149.22, 146.63, 116.43, 108.59, 43.14, 40.25, 19.26, 13.50.

HRMS(ESI+) m/z calculated $[M+H]^+$ 327.2018, found 327.2020.

- **($^{tips}PyCH_2)_2NH$ (VII)**

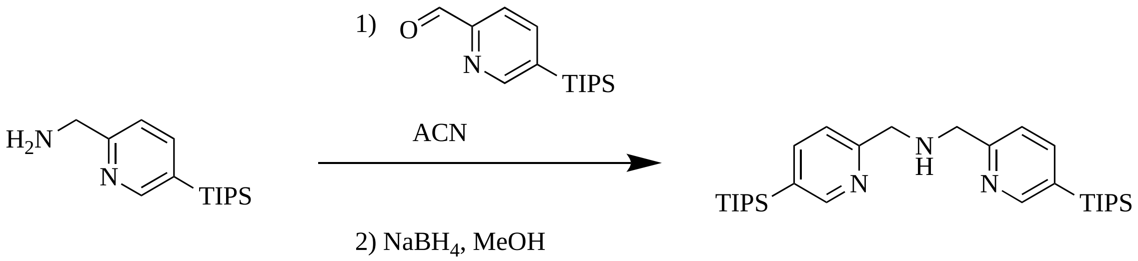

Pyridine synthons ^{tips}PyCHO and $^{tips}PyCH_2NH_2$ were synthesized as reported. [5]

To 0.2 g of $^{tips}PyCH_2NH_2$ (0.76 mmol) dissolved in 18 mL of acetonitrile in a round bottom flask it was added 0.2 g of ^{tips}PyCHO (0.76 mmol, 1 eq.) dissolved in 5 mL of acetonitrile and this last solution was added to the $^{tips}PyCH_2NH_2$ solution dropwise. A white precipitate was formed and after 30 minutes stirring the white solid was filtered and dried.

The white solid was dissolved in 5 mL of methanol and 89 mg of powdered $NaBH_4$ (2.28 mmols, 3 eq.) were slowly added. The solution became pink and was left stirring overnight at room temperature. The next morning NaOH aq. 1M was added and the product was extracted with dichloromethane. The organic phase was dried with $MgSO_4$ and the solvent was removed under vacuum and 0.19 g of ($^{tips}PyCH_2)_2NH$ (VII) were obtained as a yellow oil (49% yield). The product was used without further purification.

^{1}H NMR (400 MHz, Chloroform-d) δ 8.63 (dd, J = 1.9, 1.0 Hz, 2H), 7.74 (dd, J = 7.7, 1.8 Hz, 2H), 7.36 (dd, J = 7.7, 1.0 Hz, 2H), 4.01 (d, J = 6.6 Hz, 4H), 1.40 (hept, J = 7.4 Hz, 6H), 1.07 (d, J = 7.5 Hz, 36H).

^{13}C NMR (101 MHz, Chloroform-d) δ 159.78, 155.15, 143.66, 127.83, 121.76, 55.14, 18.58, 10.77.

ESI-MS m/z calculated $[M+H]^{+}$ 512.4, found 512.4.

3.2. Ligand assembly and characterization

- **5-tips2tpa.**

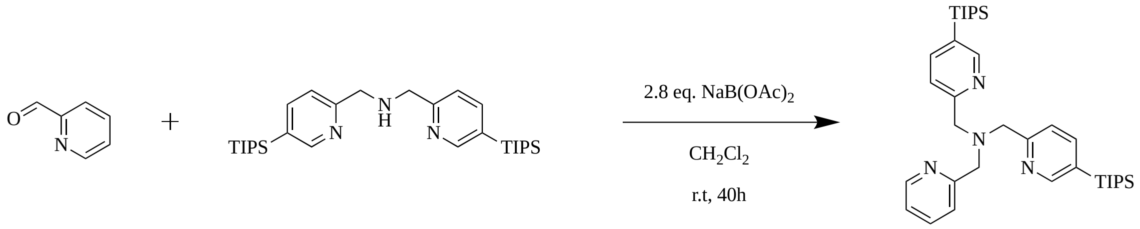

5-tips2tpa was synthetized following the reported procedure.[6] Starting with 59 mg of (^{tips}PyCH$_2$)$_2$NH (0.12 mmols), 22 µmL of picolinaldehyde (0.24 mmols, 2 eq.) and 68 mg of NaB(OAc)$_2$ (0.32 mmols, 2.8 eq.) in 1.6 mL of dichloromethane, the reaction was left stirring for 40h. After this time, a saturated solution of NaHCO3 was added and was left stirring 5 hours, the organic phase was separated, dried with $MgSO_4$ and the solvent was removed under vacuum. The crude was purified by flash chromatography on silica gel (SiO_2; Hexane:AcOEt, 9:1) to yield 52.2 mg of ligand (0.09 mmols, 75% yield) as a yellow oil.

^{1}H NMR (400 MHz, Chloroform-d) δ 8.59 (dd, J = 1.9, 1.0 Hz, 2H), 8.51 (ddd, J = 4.9, 1.8, 1.0 Hz, 1H), 7.75 (dd, J = 7.8, 1.8 Hz, 2H), 7.65 – 7.56 (m, 4H), 7.11 (ddd, J = 6.8, 4.9, 1.7 Hz, 1H), 3.91 (s, 6H), 1.43 – 1.32 (m, 6H), 1.05 (d, J = 7.5 Hz, 36H).

^{13}C NMR (101 MHz, Chloroform-d) δ 159.51, 159.21, 154.71, 148.99, 143.75, 136.66, 128.07, 123.12, 122.28, 122.12, 60.43, 60.39, 18.56, 10.74.

HRMS(ESI+) m/z calculated $[M+Na]^+$ 625.4092, found 625.4088.

- **5-tips2,6Metpa**

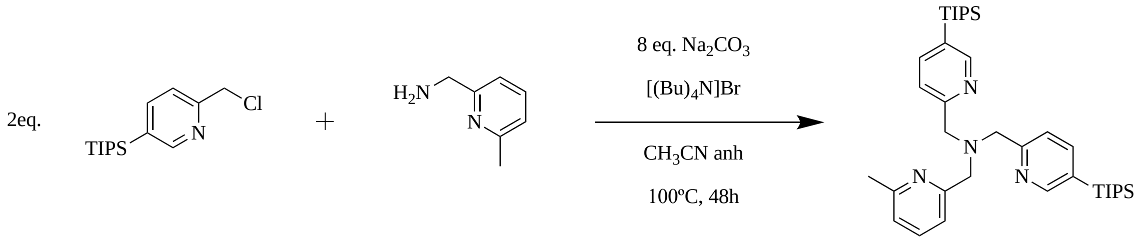

5-tips2,6Metpa was synthetized following the procedure reported[5] starting with 0.164 g of ^{tips}PyCH$_2$Cl (0.58 mmols, 2 eq.) and 0.035 g of (6-methylpyridin-2-yl)methanamine (0.29

mmols), to end up with 0.146 g of the ligand (0.24 mmols, 82% yield) after purification by flash chromatography on silica gel (SiO_2; Hexane:AcOEt, 9:1)

HRMS(ESI+) m/z calculated $[M+H]^+$ 639.4249, found 639.4247.

- **$^{5\text{-tips3,4NMe2}}$tpa**

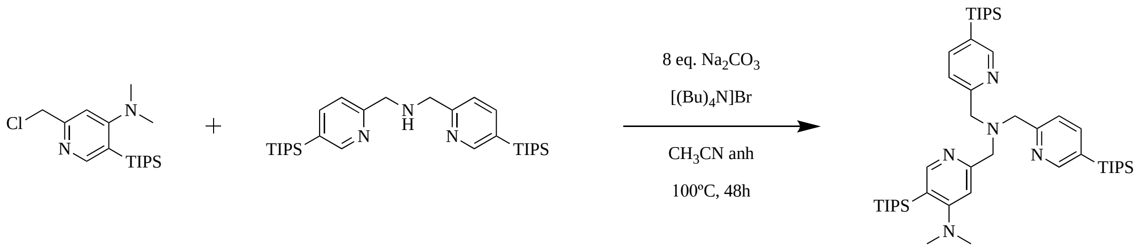

$^{5\text{-tips3,4NMe2}}$tpa was synthetized following the procedure reported[5] starting with 0.146 g of $^{\text{tips,NMe2}}$$PyCH_2Cl$ (0.44 mmols, 1.2 eq.) and 0.19 g of (^{tips}$PyCH_2)_2NH$ (0.37 mmols), with a variation of the temperature and the reaction time (100 ºC, 48 h), to end up with 0.154 g of the ligand (0.19 mmols, 52% yield) after purification by flash chromatography on silica gel (SiO_2; Hexane:AcOEt, 9:2).

^{1}H NMR (400 MHz, Chloroform-d) δ 8.51 (dd, J = 1.9, 1.0 Hz, 2H), 8.41 (s, 1H), 7.68 (dd, J = 7.8, 1.8 Hz, 2H), 7.51 (dd, J = 7.8, 1.0 Hz, 2H), 7.31 (s, 1H), 3.84 (s, 4H), 3.79 (s, 2H), 2.52 (s, 6H), 1.39 (p, J = 7.5 Hz, 3H), 1.30 (dt, J = 14.9, 7.5 Hz, 7H), 0.98 (t, J = 7.5 Hz, 54H).

^{13}C NMR (101 MHz, Chloroform-d) δ 169.7, 161.03, 159.61, 157.70, 155.03, 143.62, 127.88, 124.50, 122.16, 114.80, 60.20, 46.18, 19.42, 18.61, 12.56, 10.80.

HRMS(ESI+) m/z calculated $[M+H]^+$ 802.6029, found 802.6013.

4. Synthesis and characterization of complexes

The following catalysts were prepared following reported procedures, characteristic data agreed with the reported ones. [Fe(OTf)$_2$($^{5\text{-tips3}}$tpa)][5] OTf = trifluoromethanesulfonate anion, [Fe(OTf)$_2$(tpa)],[7] [Fe(^{COOEt}pytacn)],[8] [Fe($^{6\text{-Me}}$pytacn)].[9] The new catalyst ([Fe(OTf)$_2$($^{5\text{-tips2}}$tpa)], [Fe(OTf)$_2$($^{5\text{-tips2,6-Me}}$tpa)], [Fe($^{5\text{-tips3,4-NMe2}}$tpa)]), were prepared as the reported [Fe(OTf)$_2$($^{5\text{-tips3}}$tpa)].[5, 6, 8, 9]

Fe($^{5\text{-tips2}}$tpa)

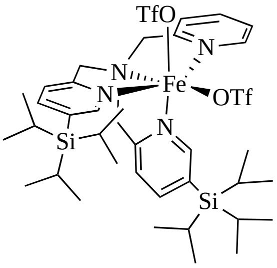

HRMS(ESI+) m/z calculated [M-OTF]$^+$ 807.3060, found 807.3065.

Elemental analysis (%) for $C_{38}H_{58}F_6FeN_4O_6S_2Si_2$ (MW: 956.6 g/mol). Calculated N: 5.85, C: 47.69, H: 6.11; obtained N: 6.02, C: 47.27, H: 5.96.

FT-IR (ATR) v, cm^{-1}: 2945, 2867, 1607, 1592, 1461, 1448, 1324, 1234, 1205, 1168, 1115, 996, 984, 882, 768, 683, 632, 569, 516.

X-Ray, CCDC (2281991)

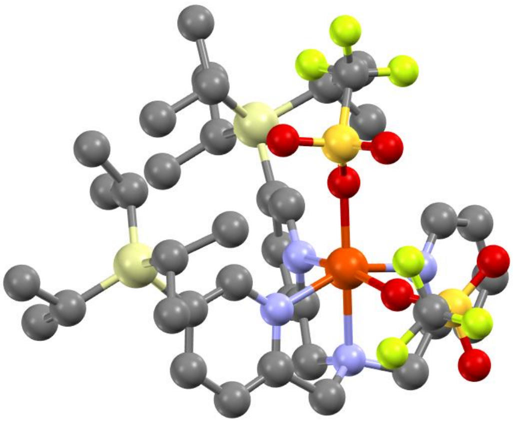

Fe($^{5\text{-tips2,6-Me}}$tpa)

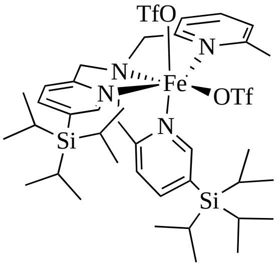

HRMS(ESI+) m/z calculated [M-OTF]$^+$ 821.3222, found 821.3244.

Elemental analysis (%) for $C_{39}H_{60}F_6FeN_4O_6S_2Si_2$ (MW: 970.27g/mol). Calculated N: 5.77, C: 48.24, H: 6.23; obtained N: 5.76, C: 47.08, H: 6.29.

FT-IR (ATR) v, cm^{-1}: 2944, 2867, 1592, 1461, 1289, 1236, 1163, 1020, 882, 681, 634, 572, 511.

X-Ray: CCDC (2281992)

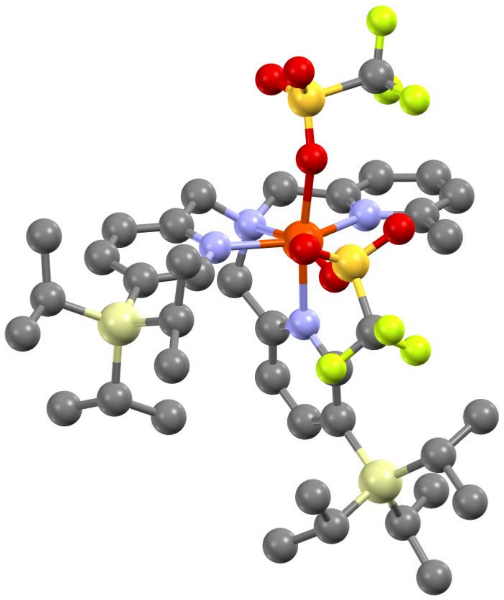

Fe($^{5\text{-tips3,4-NMe2}}$tpa)

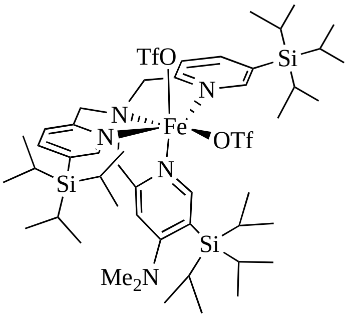

HRMS(ESI+) m/z calculated [M-OTF]$^{+}$ 1006.4803, found 1006.4822.

Elemental analysis (%) for $C_{49}H_{83}F_6FeN_5O_6S_2Si_3$ (MW: 1155.43 g/mol). Calculated N: 6.06, C: 50.89, H: 7.23; obtained N: 6.00, C: 49.24, H: 7.06.

FT-IR (ATR) v, cm^{-1}: 2944, 2867, 1591, 1461, 1318, 1286, 1234, 1165, 1024, 882, 682, 635, 570, 516.

5. Reaction conditions for *syn*-dihydroxylation of arenes

5.1. Experimental conditions for maximizing diol (**2x**) formation

In a 3mL vial equipped with a stir bar a solution of substrate (0.45 μmol) in 400 μL of acetonitrile was added, afterwards $Mg(ClO_4)_2 \cdot 6H_2O$ (65.6 mg, 1.98 μmol, 4.4 eq.) and the catalyst (1.5 mg, 0.0135 μmol, 3 mol%) were added. The mixture was cooled in an ice bath and a solution of H_2O_2 50% aq. (76.3 μL, 0.59 M, 1 eq.) was added via syringe pump for 30 minutes.

5.2. Experimental conditions for maximizing tetraol (**3x**) formation

In a 3mL vial equipped with a stir bar a solution of substrate (0.45 μmol) in 400 μL of acetonitrile was added, afterwards $Mg(ClO_4)_2 \cdot 6H_2O$ (65.6 mg, 1.98 μmol, 4.4 eq.) and the catalyst (1.5 mg, 0.0135 μmol, 3 mol%) were added. The mixture was cooled in an ice bath and a solution of H_2O_2 50% aq. (114.5 μL, 0.59 M, 1.5 eq.) was added via syringe pump for 30 minutes.

After this, a second addition of catalyst (3 mol%) and H_2O_2 (1.5 eq.) was performed under the same conditions. If required, a third addition was performed leading to the total time of the reaction up to 1.5h.

5.3. Workup of the catalysis

After the catalysis was finished, 0.1mL of methyl imidazole and 1mL of acetic anhydride were added and stirred for 45 minutes at room temperature. Then ice was added, and the solution was stirred till the ice dissolved and the internal standard (biphenyl) was added.

A first extraction with dichloromethane was performed, followed by an extraction with H_2SO_4 1M of the organic solution. Afterwards the organic phase was extracted with $NaHCO_3$ sat., subsequently cleaned with water, and finally dried with $MgSO_4$. The crude of the reaction was directly analyzed by GC. GC analysis of the solution provided substrate conversions and product yields relative to the internal standard.

6. Optimization

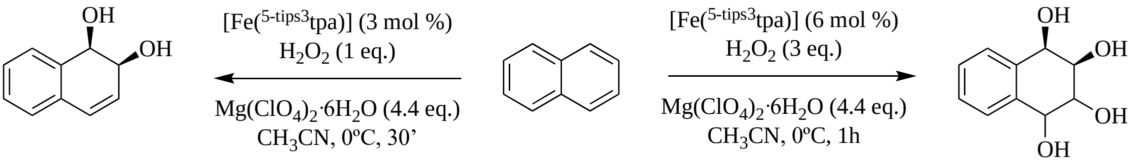

2a 1a 3a

Table S1. Optimization of the catalyst loading without additives.

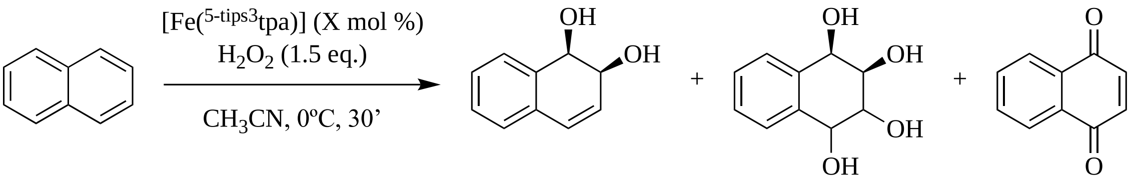

1a 2a 3a 4a

Entry[a]	Cat. Loading (X mol %)	Conv. (%)	**2a** (%)	**3a** (%) (*syn*/anti)	**4a** (%)
1	1	52	13	6 (3.2)	2
2	4	76	21	14 (3.8)	2

[a] Reaction conditions as described in Diol formation (5.1.1 and 5.1.3).

Table S1. Effect of $Mg(ClO_4)_2 \cdot 6H_2O$ for the formation of **2a**.

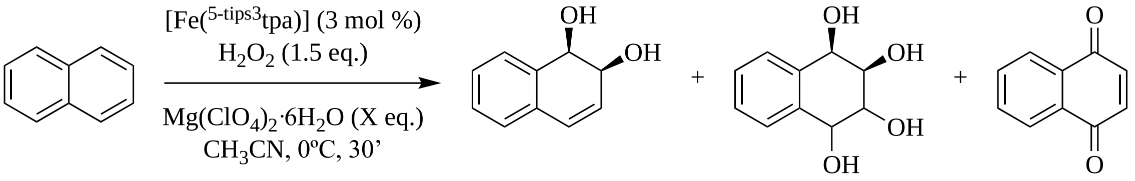

1a 2a 3a 4a

Entry[a]	Equivalents of $Mg(ClO_4)_2 \cdot 6H_2O$	Conv. (%)	**2a** (%)	**3a** (%) (*syn*/anti)	**4a** (%)
1[b]	0	56	5	8 (4.0)	3
2[b]	1.1	75	12	16 (3.4)	1
3[b]	2.2	72	17	12 (3.2)	3
4	0	76	19	14 (3.9)	2
5	1.1	54	18	9 (4.3)	2
6	2.2	72	14	17 (4.6)	2
7[c]	0	48	11	11 (6.2)	1

8[c]	1.1	58	19	10 (4.7)	1
9[c]	2.2	54	23	9 (4.3)	1
10[b]	4.4	50	29	5 (3.1)	1

[a] Reaction conditions as described in Diol formation (5.1.1 and 5.1.3). [b] 2 mol % of catalyst. [c] 1 eq. of H_2O_2.

Table S3. Effect of $Mg(ClO_4)_2 \cdot 6H_2O$ for the formation of 3a.

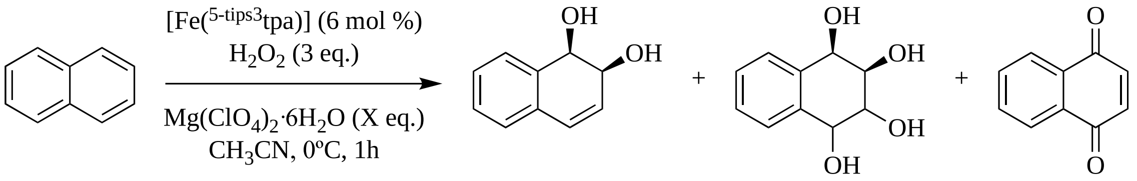

1a 2a 3a 4a

Entry[a]	Equivalents of $Mg(ClO_4)_2 \cdot 6H_2O$	Conv. (%)	**2a** (%)	**3a** (%) (*syn*/anti)	**4a** (%)
1[b]	0	87	10	18 (2.7)	4
2[b]	2.2	59	15	4 (2.2)	2
3	0	91	2	27 (4.5)	3
4	2.2	99	3	46 (4.6)	6
5	4.4	88	0	47 (4.1)	6

[a] Reaction conditions as described in tetraol formation (5.1.2 and 5.1.3). [b]4mol% of catalyst.

Table S4. Optimization with $Mg(ClO_4)_2 \cdot 6H_2O$ as additive for the formation of **2a**.

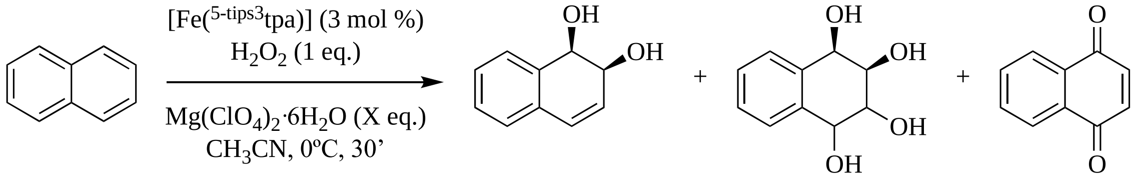

1a 2a 3a 4a

Entry[a]	Equivalents of $Mg(ClO_4)_2 \cdot 6H_2O$	Conv. (%)	**2a** (%)	**3a** (%) (*syn*/anti)	**4a** (%)
1 [b]	2.2	55	15	9 (3.2)	2
2	2.2	54	23	9 (4.3)	1
3	4.4	50	29	5 (3.1)	1
4 [c]	4.4	54	27	5 (3.1)	3
5[d]	4.4	50	19	4 (3.1)	4

6[b,e]	1.1	64	9	9 (3.0)	2
7	1.1	53	18	9 (4.3)	<1

[a] Reaction conditions as described in Diol formation (5.1.1 and 5.1.3). [b] 1 mol% of catalyst, [c] 1h of reaction time. [d] 2 equivalents of H_2O_2. [e]3 equivalents of H_2O_2.

Table S5. Catalyst loading with $Mg(ClO_4)_2{\cdot}6H_2O$ as additive for the formation of **3a**.

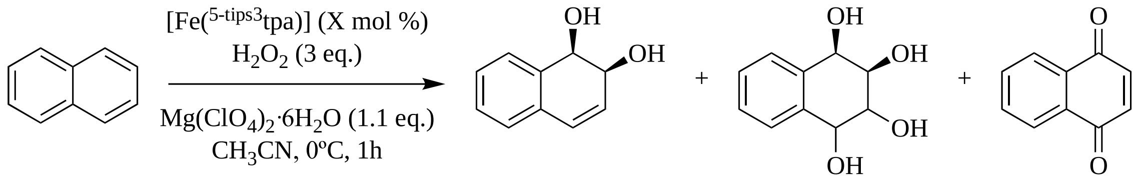

1a 2a 3a 4a

Entry[a]	Cat. Loading (X mol %)	Conv. (%)	**2a** (%)	**3a** (%) (*syn*/anti)	**4a** (%)
1[b]	6	88	0	47 (4.1)	6
2[b,c]	6	100	1	48 (2.7)	5
3[d,e]	6	99	3	46 (4.6)	6
4	2	92	1	20 (3.3)	3
5	6	99	1	42 (4.2)	2
6[f]	6	99	1	43 (4.3)	3
7[f]	3	84	6	23 (3.4)	4
8[f]	5	78	15	25 (4.6)	2
9	8	98	2	41 (4.5)	2

[a] Reaction conditions as described in tetraol formation (5.1.2 and 5.1.3). [b] 4.4 equivalents of $Mg(ClO_4)_2{\cdot}6H_2O$. [c] 5 equivalents of H_2O_2. [d] 2.2 equivalents of $Mg(ClO_4)_2{\cdot}6H_2O$. [e] 6 equivalents of H_2O_2. [f] 2 mol% of catalyst on the second addition.

Table S6. Temperature effect.

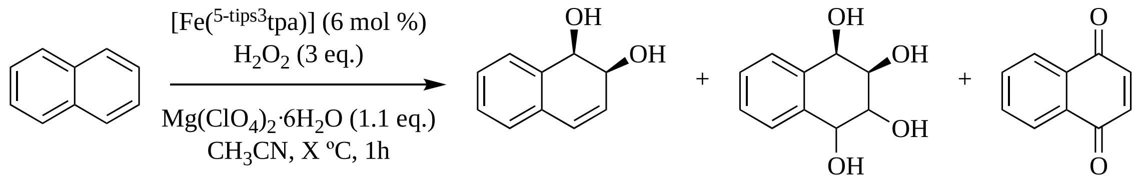

1a 2a 3a 4a

Entry[a]	Temperature	Conv. (%)	**2a** (%)	**3a** (%) (*syn*/anti)	**4a** (%)
1	-40	84	8	35 (4.0)	5

2	0	99	1	42 (4.2)	2
3	25	94	5	33 (4.1)	2

[a] Reaction conditions as described in tetraol formation (5.1.2 and 5.1.3).

Table S7. Reaction time effect.

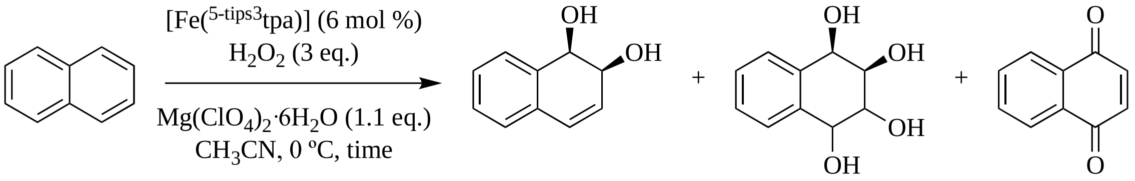

1a 2a 3a 4a

Entry[a]	Reaction time	Conv. (%)	**2a** (%)	**3a** (%) (*syn*/anti)	**4a** (%)
1	30'	95	4	45 (4.4)	5
2	1h	99	1	42 (4.2)	2
3	1h 30'	97	3	43 (4.3)	4

[a] Reaction conditions as described in tetraol formation (5.1.2 and 5.1.3).

Table S8. Concentration of substrate effect.

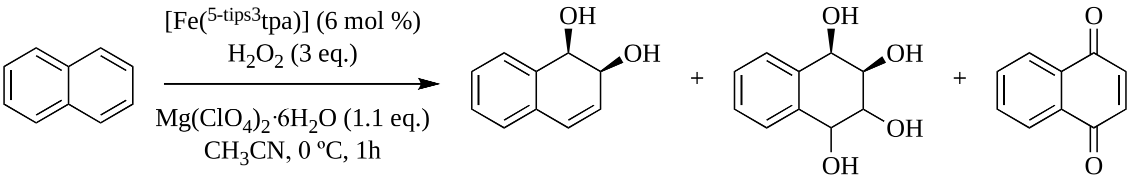

1a 2a 3a 4a

Entry[a]	Concentration [mM]	Conv. (%)	**2a** (%)	**3a** (%) (*syn*/anti)	**4a** (%)
1	22.6	95	3	42 (4.6)	54
2	90.1	98	2	44 (4.0)	3

[a] Reaction conditions as described in tetraol formation (5.1.2 and 5.1.3).

Table S9. Additive screening.

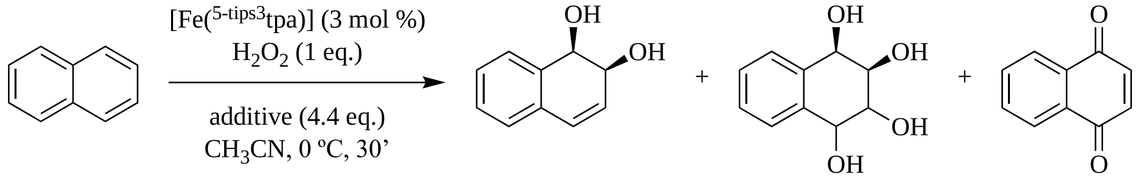

1a 2a 3a 4a

Entry[a]	Additive	Conv. (%)	**2a** (%)	**3a** (%) (*syn*/anti)	**4a** (%)
1	None	48	11	11 (6.2)	1
2	$Mg(ClO_4)_2·6H_2O$	50	29	5 (3.1)	1
3	$Zn(ClO_4)_2·6H_2O$	51	22	1 (0.4)	2
4	$Li(ClO_4)·3H_2O$	48	23	9 (3.9)	1
5	$Mg(OTf)_2$	47	13	1 (1.9)	2
6	$Mg(OTf)_2$	84	17	16 (4.1)	3
7 [b]	$Zn(OTf)_2$	57	22	5 (2.4)	1
8 [c]	$Zn(OTf)_2$	57	25	2 (1.2)	2
9 [d]	$Zn(OTf)_2$	52	24	1 (0.6)	2
10	$Zn(OTf)_2$	54	29	3 (1.9)	1
11 [e]	$Zn(OTf)_2$	50	22	5 (3.6)	3

[a] Reaction conditions as described in diol formation (5.1.1 and 5.1.3). [b] 1.1 equivalents of additive. [c] 2.2 equivalents of additive. [d] 3.3 equivalents of additive. [e] 5.5 equivalents of additive.

Table S10. Additive screening.

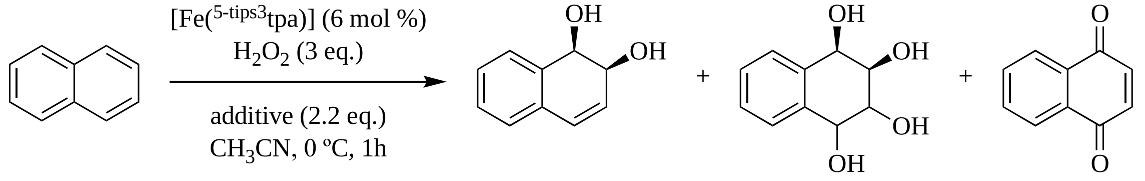

1a 2a 3a 4a

Entry[a]	Additive	Conv. (%)	**2a** (%)	**3a** (%) (*syn*/anti)	**4a** (%)
1	None	91	2	27 (4.5)	3
2	$Mg(ClO_4)_2·6H_2O$	99	3	46 (4.6)	6
3	Butylboronic acid	74	13	0	7

4	4-(Trifluoromethyl) phenylboronic acid	73	11	0	1
5	Acetone	5	0	0	0
6	CDI	4	0	0	0

[a] Reaction conditions as described in tetraol formation (5.1.2 and 5.1.3). CDI: carbonyldiiimidazole.

Table S11. Solvent effect for the formation of **2a**.

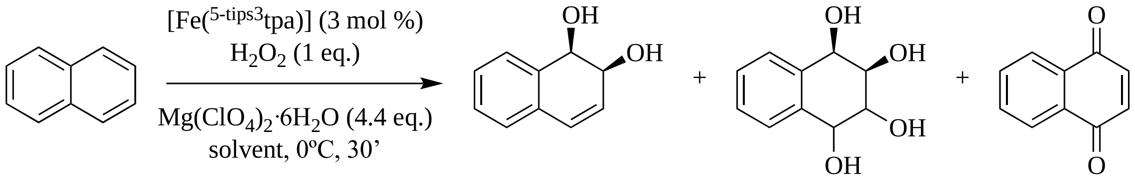

1a 2a 3a 4a

Entry[a]	Solvent	Conv. (%)	**2a** (%)	**3a** (%) (*syn*/anti)	**4a** (%)
1	Acetonitrile	50	29	5 (3.1)	1
4	Butyronitrile	50	21	6 (5.1)	1
5	CH_3CN:HFIP (95:5)	59	25	5 (3.1)	1
6	CH_3CN:HFIP (9:1)	57	20	4 (2.6)	1
7[b]	CH_3CN:HFIP (9:1)	48	7	8 (6.2)	1
8	CH_3CN:TFE (9:1)	58	23	4 (3.0)	1

[a] Reaction conditions as described in diol formation (5.1.1 and 5.1.3). [b] No additive was added.

Table S12. Solvent effect for the formation of **3a**.

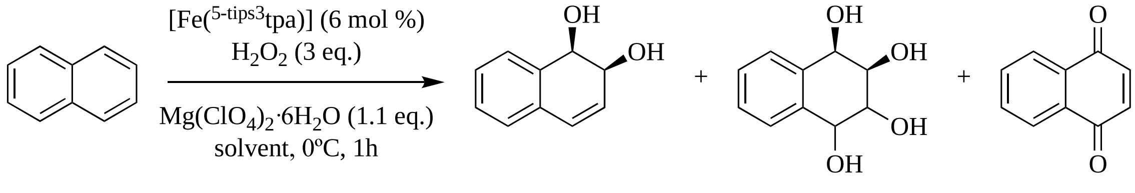

1a 2a 3a 4a

Entry[a]	Solvent	Conv. (%)	**2a** (%)	**3a** (%) (*syn*/anti)	**4a** (%)
1[b]	Acetonitrile	88	0	47 (4.1)	6
2	Acetonitrile	99	1	42 (4.2)	2
3	AcOEt	85	1	35 (3.6)	4
4[b]	AcOEt:PC (1:1)	96	2	38 (7.5)	3
5	AcOEt:Butyronitrile (9:1)	85	1	40 (4.4)	5

6	Acetonitrile:DCM (1:1)	68	5	20 (9.5)	4
7	DMF	2	0	0	0
8	THF	5	0	0	0
9	HFIP	3	0	0	0

[a] Reaction conditions as described in tetraol formation (5.1.2 and 5.1.3). [b] 4.4 eq. $Mg(ClO_4)_2·6(H_2O)$. PC: Propylene carbonate.

Table S13. Reaction under air or under nitrogen for the formation of **2a**.

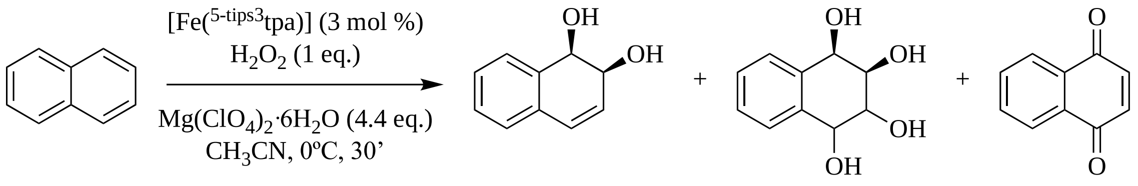

1a 2a 3a 4a

Entry[a]	Solvent	Conv. (%)	**2a** (%)	**3a** (%) (*syn*/anti)	**4a** (%)
1	Air	50	29	5 (3.1)	1
2	N_2	51	23	3 (3.3)	1

[a] Reaction conditions as described in diol formation (5.1.1 and 5.1.3).

Table S14. 4-(Trifluoromethyl) phenylboronic acid as potential **2a** trapping agent.

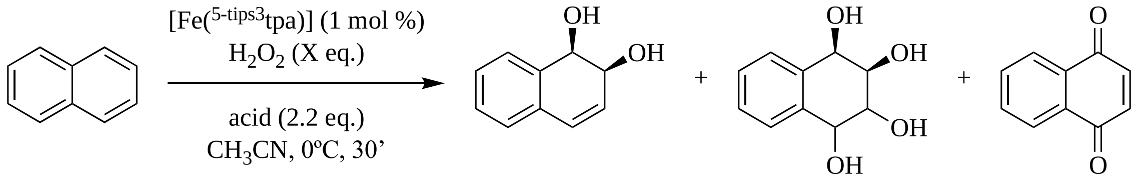

1a 2a 3a 4a

Entry [a]	Eq. of H_2O_2	Conv. (%)	**2a** (%)	**3a** (%) (*syn*/anti)	**4a** (%)
1 [b]	2	47	11	0	4
2	2	35	7	0	2
3 [c]	2	22	9	0	2
4	4	50	9	0	3
5 [c]	4	44	9	0	4
6 [c]	3	47	13	0	3
7 [d]	1	70	17	0	4
8 [d]	4	77	19	0	5
9	3	69	19	0	5

[a] Reaction conditions as described in diol formation (5.1.1 and 5.1.3). [b] 1.1 eq. of 4-(Trifluoromethyl) phenylboronic acid. [c] 15’ reaction time. [d] 3 mol % cat.

Table S15. 4-(Trifluoromethyl) phenylboronic acid as potential **2a** trapping agent with double additions.

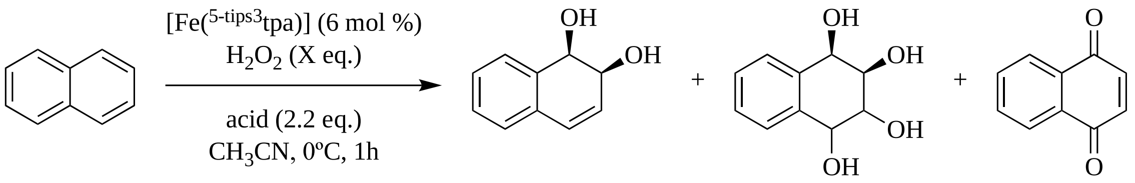

1a 2a 3a 4a

Entry [a]	Total eq. of H_2O_2	Conv. (%)	**2a** (%)	**3a** (%) (*syn*/anti)	**4a** (%)
1 [b]	2	67	25	0	5
2	3	73	11	0	1
3 [c]	3	58	16	0	1
4 [c,d]	4.5	89	23	0	1

[a] *Reaction conditions as described in tetraol formation (5.1.2 and 5.1.3).* [b] 4.4 eq. $Mg(ClO_4)\cdot 6(H_2O)$ were added with the boronic acid. [c] 4.4 eq. of 4-(Trifluoromethyl) phenylboronic acid. [d] Triple addition of catalyst and H_2O_2 at 1h (total amount of catalyst 9 mol %).

Table S16. Effect of hexafluoro acetone trihydrate as potential trapping agent of **3a**.

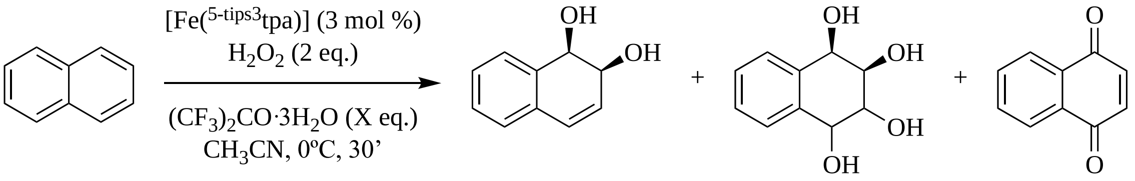

1a 2a 3a 4a

Entry [a]	Eq. of $(CF_3)_2CO\cdot 3H_2O$	Conv. (%)	**2a** (%)	**3a** (%) (*syn*/anti)	**4a** (%)
1 [b,c]	2	34	8	9 (5.6)	1
2	1	75	5	21 (4.1)	2
3	2	70	6	20 (4.0)	3
4	3	66	7	16 (5.6)	3
5	4	68	6	15 (5.5)	2

[a] *Reaction conditions as described in diol formation (5.1.1 and 5.1.3).* [b] 1mol % of catalyst. [c] 1.5 eq. H_2O_2.

Table S17. Effect of hexafluoro acetone trihydrate as additive potential trapping agent of **3a** with double additions.

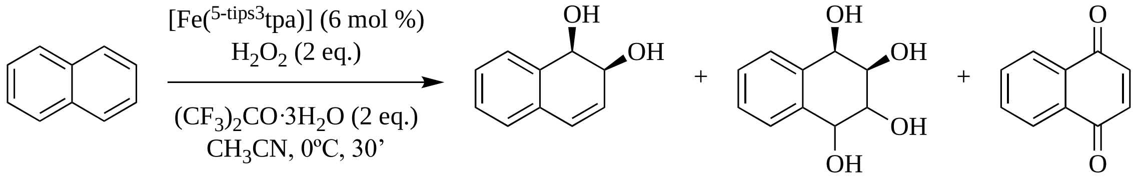

1a 2a 3a 4a

Entry [a]	Conv. (%)	**2a** (%)	**3a** (%) (*syn*/anti)	**4a** (%)
1 [b]	69	6	17 (5.8)	2
2	76	6	26 (4.6)	3
3 [c]	95	1	35 (4.2)	3

[a] Reaction conditions as described in tetraol formation (5.1.2 and 5.1.3). [b] 3mol% of catalyst. [c] 3 eq. of H_2O_2.

Table S18. Bis-dihydroxylation yields and ratios employing different Lewis acids.

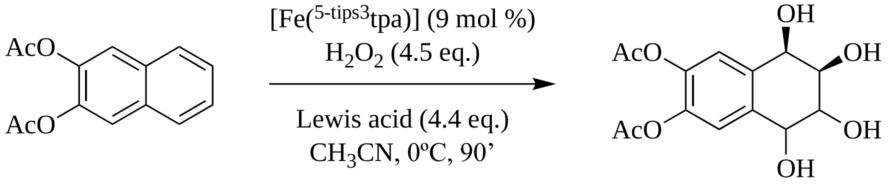

1d 3d

Entry [a]	Lewis acid 4.4 eq.	**3d**	Ratio *syn*/*anti*
1	None	25	3.3
2	$Zn(OTf)_2$	59	1.5
3	$Mg(ClO_4)_2 \cdot 6H_2O$	62	2.4
4	$Li(ClO_4) \cdot 6H_2O$	41	2.5

[a] Reaction conditions: 1 eq. substrate (4.5 μmol), 3 mol % catalyst (with a second and third addition of another 3 mol % at every 30'), 4.5 eq. H_2O_2 (50% w/w solution in H_2O, via syringe pump over 1.5h), 4.4 eq. $Mg(ClO_4)_2 \cdot 6H_2O$ (19.8 μmol), CH_3CN (4mL), 1.5h, 0ºC. Yields are determined by ^{1}H-NMR.

Table S19. Catalyst screening with the respective duplicates.

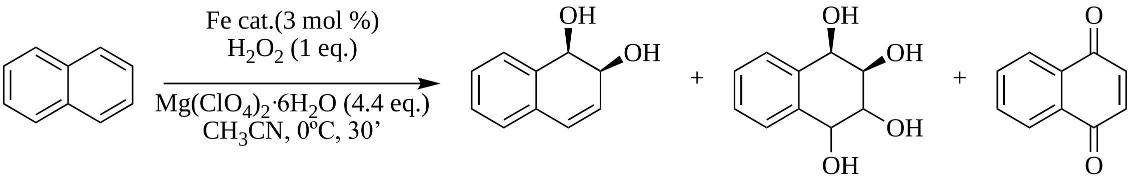

1a 2a 3a 4a

Entry [a]	Catalyst	Conv. (%)	**2a** (%)	**3a** (%) (*syn*/anti)	**4a** (%)
1	[Fe($^{5\text{-tips3}}$tpa)]	50	29	5 (3.1)	1
2		54	28	5 (3.3)	3
3	[Fe(tpa)]	30	9	1 (1.2)	3
4		42	9	1 (1.4)	2
5	[Fe($^{5\text{-tips2}}$tpa)]	53	16	1 (1.6)	2
6		52	18	2 (2.1)	4
7	[Fe($^{5\text{-tips2,6-Me}}$tpa)]	40	14	4 (3.1)	2
8		41	13	4 (3.9)	1
9	[Fe($^{5\text{-tips3,4-NMe2}}$tpa)]	31	12	1 (1.8)	0
10		36	15	1 (1.8)	1
11	[Fe(^{COOEt}pytacn)]	37	7	3 (3.8)	5
12		31	7	3 (3.9)	3
13	[Fe($^{6\text{-Me}}$pytacn)]	37	1	< 1 (2.5)	4
14		34	1	< 1 (2.4)	3
15	$Fe(OTf)_2$	20	< 1	< 1	< 1
16	None	7	-	-	-
17		3	-	-	-

[a] *Reaction conditions as described in diol formation (5.1.1 and 5.1.3).*

Table S20. Competition reaction between naphthalene and benzene.

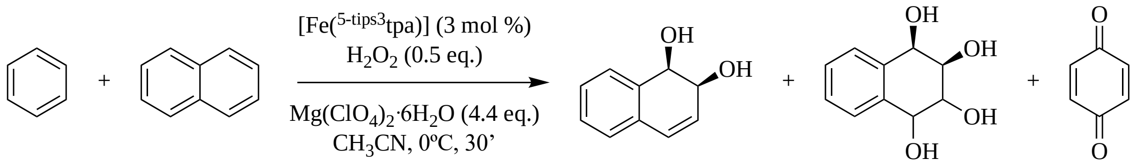

0.5 eq. 0.5 eq. 2a 3a 4a

Entry [a]	Conv. Benzene (%)	Conv. Naphthalene (%)	**2a** (%)	**3a** (%)	**4a** (%)
1	62	41	19	1	1

[a] *Reaction conditions as described in diol formation (5.1.1 and 5.1.3).*

Table S21. Allylic alcohol oxidation.

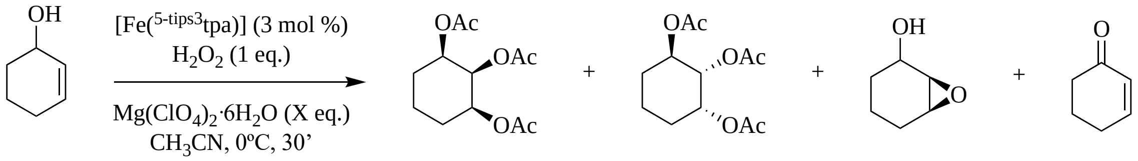

a b c d

Entry [a]	Equivalents of $Mg(ClO_4)_2·6H_2O$	Conv. (%)	**a** (%)	**b** (%)	**Ratio a/b**	**c** (%)	**d** (%)
1	0	48	29±2	16±2	1.8	-	traces
2	4.4	58	32±2	29±2	1.1	-	traces

[a] *Reaction conditions as described in diol formation (5.1.1 and 5.1.3).* Yields determined by GC. Isomers were identified by ^{1}H-NMR and COSY experiments.

The reaction was proved to proceed through a mechanism where hydroxyl radicals are not significantly involved with the following experiments, where two hydroxyl radical trapping agents[10, 11] were used, and the *syn*/*anti* ratio remained consistent with the results in the absence of trapping agents.

Table S22. Radical scavengers.

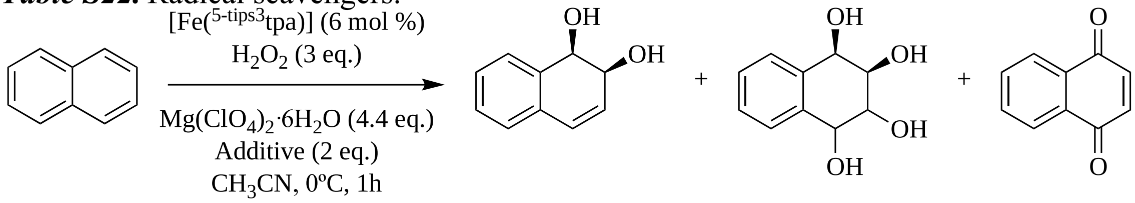

1a 2a 3a 4a

Entry [a]	Additive	Conv. (%)	**2a** (%)	**3a** (%) (*syn*/anti)	**4a** (%)
1[b]	DMSO	92	3	18 (3.0)	4
2	t-BuOH	100	0	41 (4.1)	3

[a] *Reaction conditions as described in diol formation (5.1.2 and 5.1.3).* [b] Not clean reaction.

7. Reaction monitoring

7.1. Product formation

Table S23. Monitoring the formation of diol **2a**.

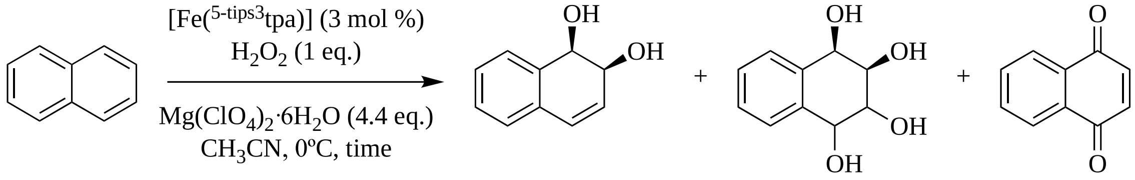

1a 2a 3a 4a

Entry [a]	Minutes	Conv. (%)	**2a** (%)	**3a** (%) (*syn*/anti)	**4a** (%)
1	5	20	5	0	1
2	10	20	11	0	0
3	15	27	17	1	0
4	20	40	23	2 (3)	0
5	25	49	27	4 (3)	0
6	30	43	28	4 (3)	0

[a] *Reaction conditions as described in diol formation (5.1.1 and 5.1.3).*

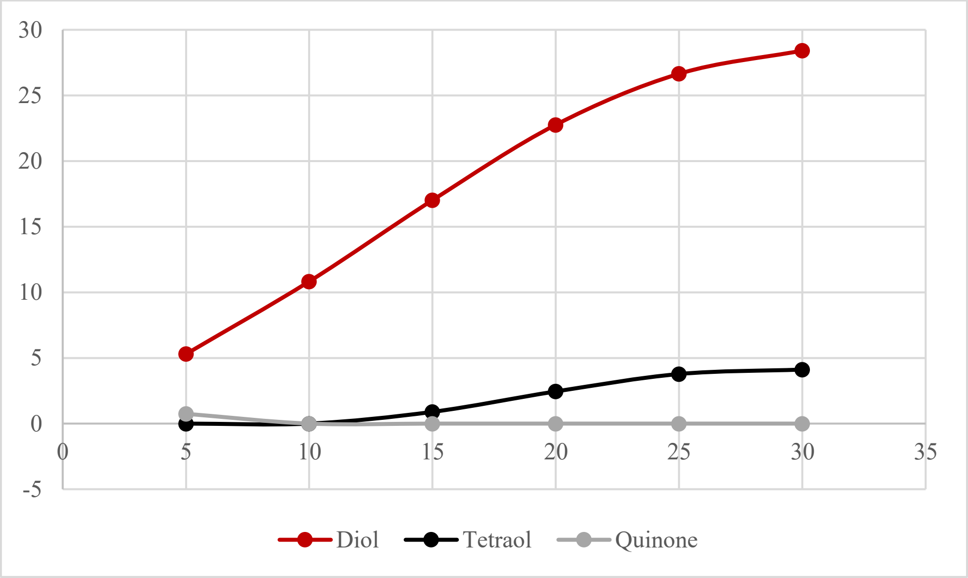

Graphic S1. Representation of the product formation in the reaction of *Table 23*.

Table S24. Monitoring the formation of tetraol **3a**.

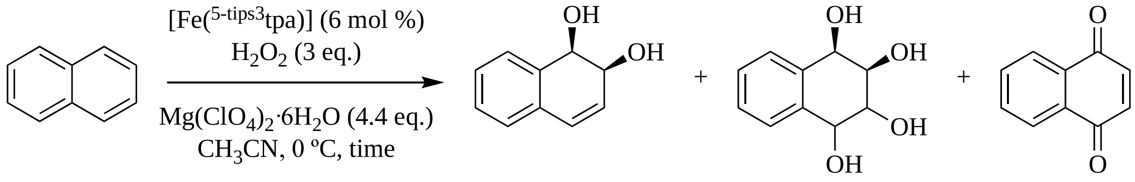

1a 2a 3a 4a

Entry [a]	Minutes	Conv. (%)	**2a** (%)	**3a** (%) (*syn*/anti)	**4a** (%)
1	5	24	10	0	0
2	10	33	17	1 (3.9)	0
3	15	35	27	4 (4.0)	0
4	20	52	30	7 (4.1)	0
5	25	64	30	10 (3.9)	0
6	30	70	32	16 (3.8)	2
7	35	85	23	19 (3.9)	1
8	40	91	19	24 (3.9)	2
9	45	93	16	32 (4.2)	2
10	50	98	10	39 (4.0)	3
11	55	99	4	45 (4.2)	2
12	60	100	1	50 (4.3)	3

[a] *Reaction conditions as described in tetraol formation (5.1.2 and 5.1.3).*

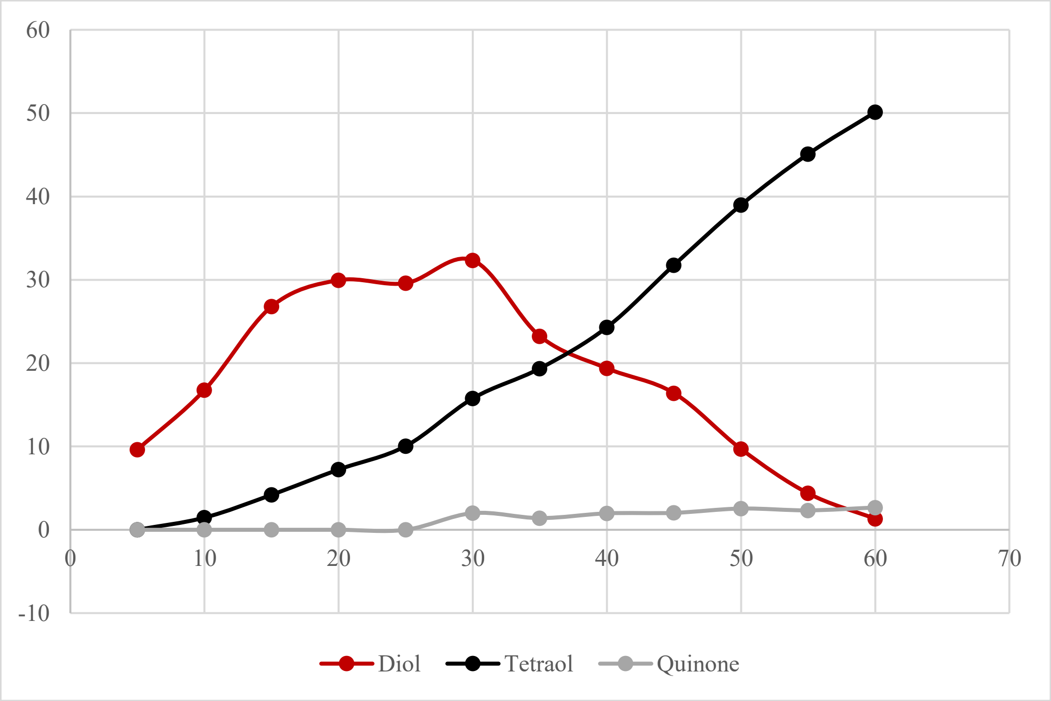

Graphic S2. Representation of the product formation in the reaction of *Table 24*.

7.1. HRMS monitoring of a catalytic reaction.

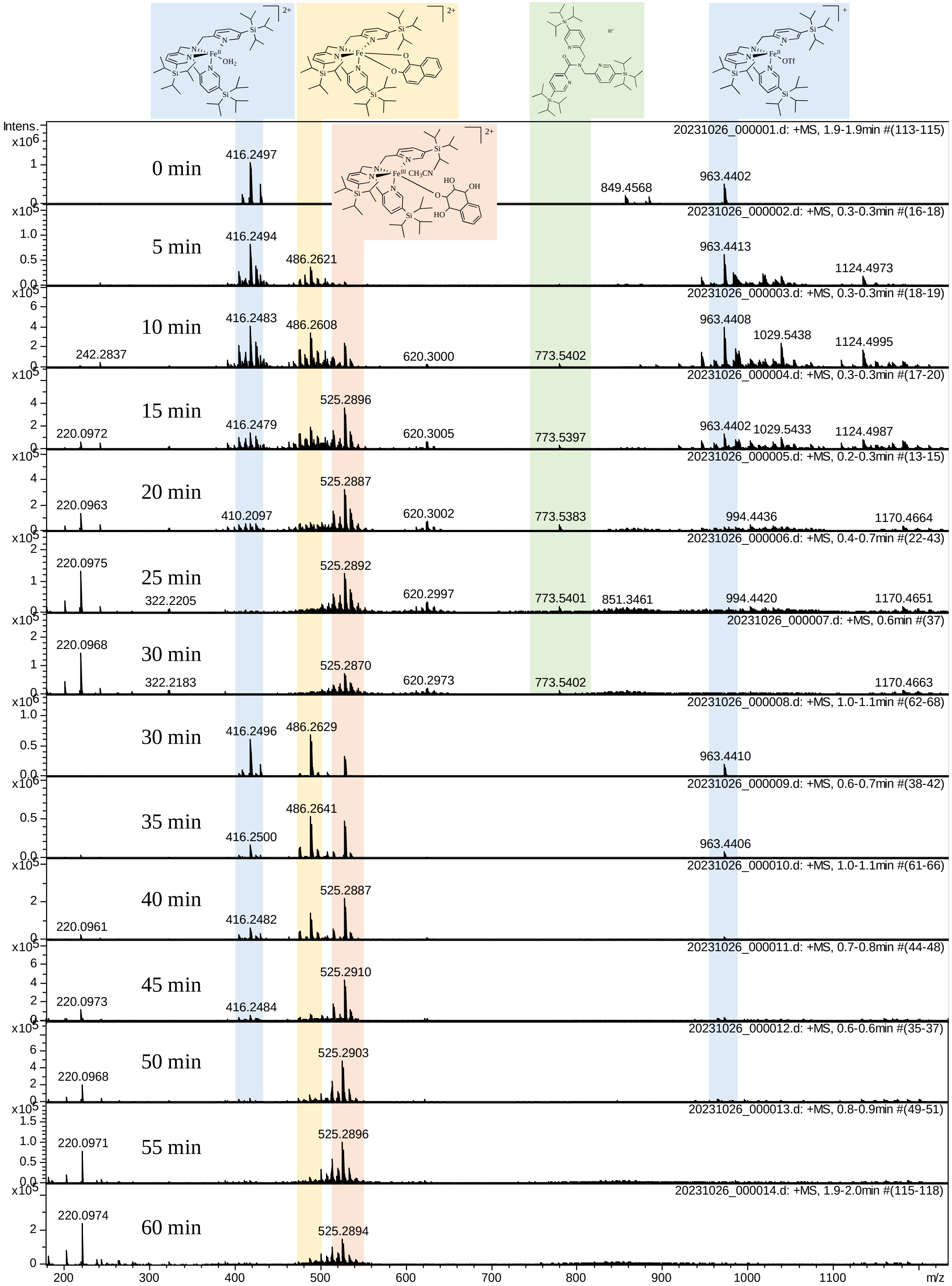

Figure S1. HRMS analysis of the reaction under tetraol formation conditions (5.1.2) without the use of $Mg(ClO_4)_2 \cdot 6H_2O$.

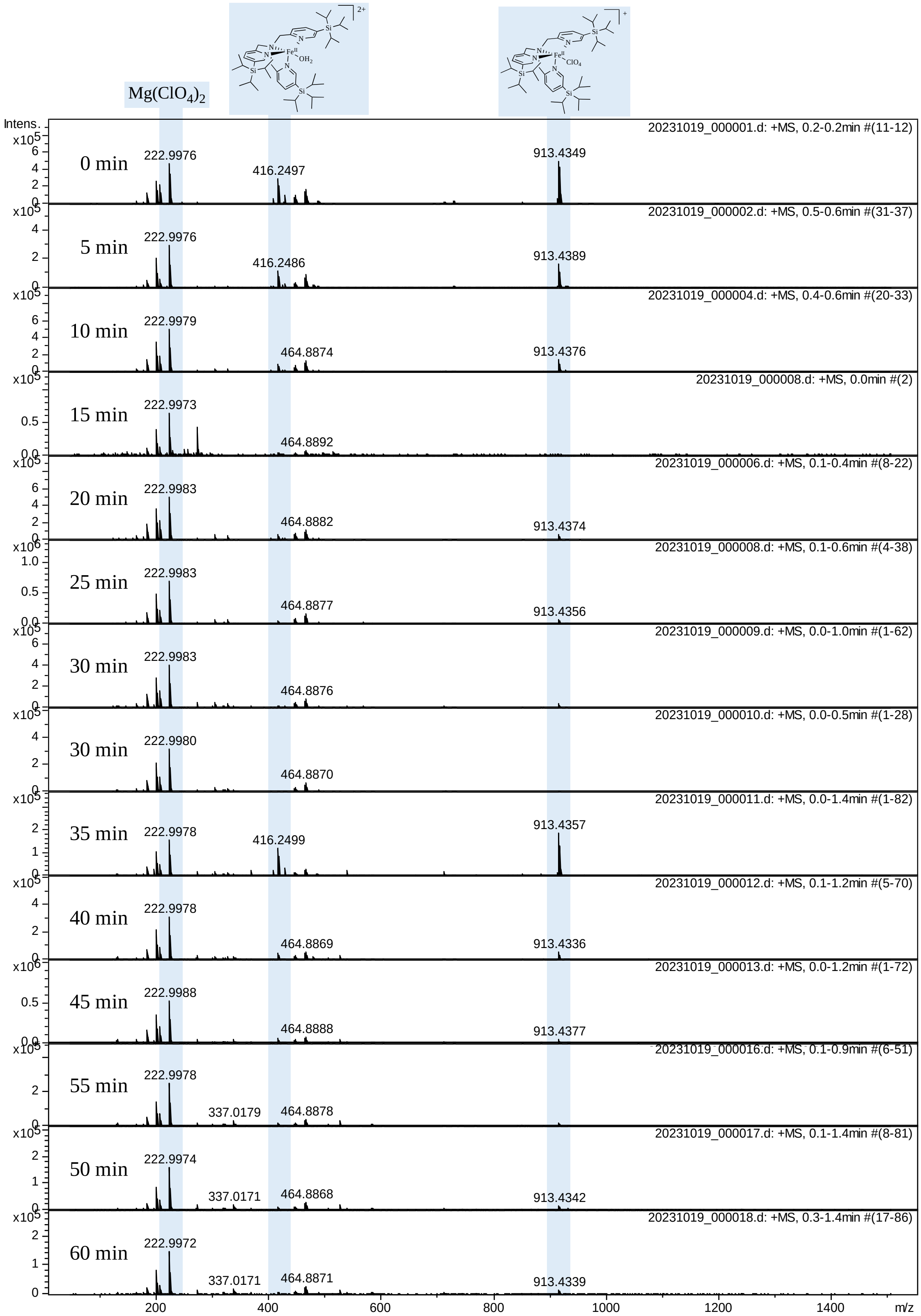

Figure S2. HRMS analysis of the reaction under tetraol formation conditions (5.1.2).

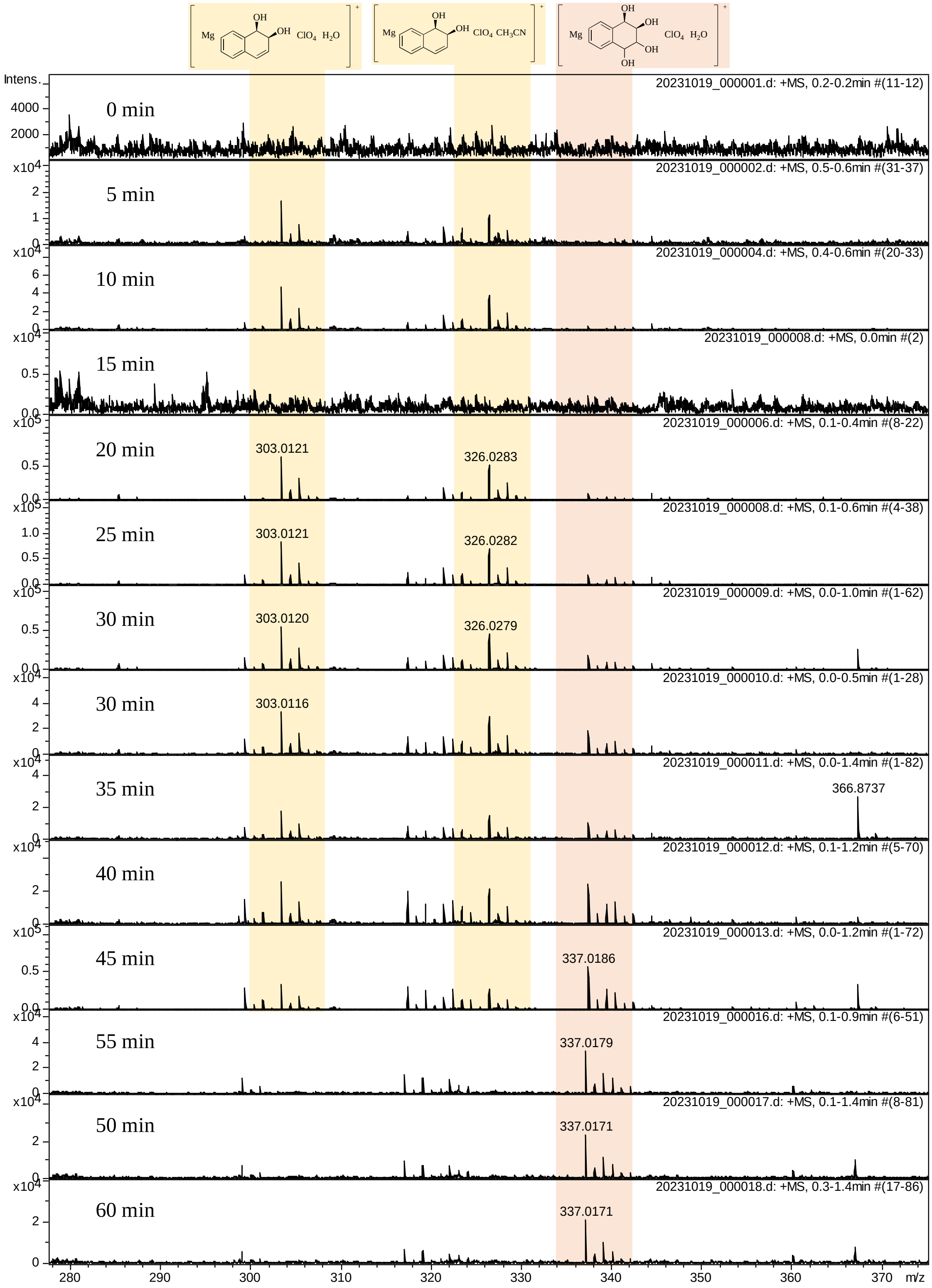

Figure S3. Amplified area of the spectrum *Figure S2* to observe the diol and tetraol formation.

7.2. Experimental and predicted HRMS of the detected compounds

For each pair of spectra, the top one corresponds to experimental data while the bottom one is the simulated spectra.

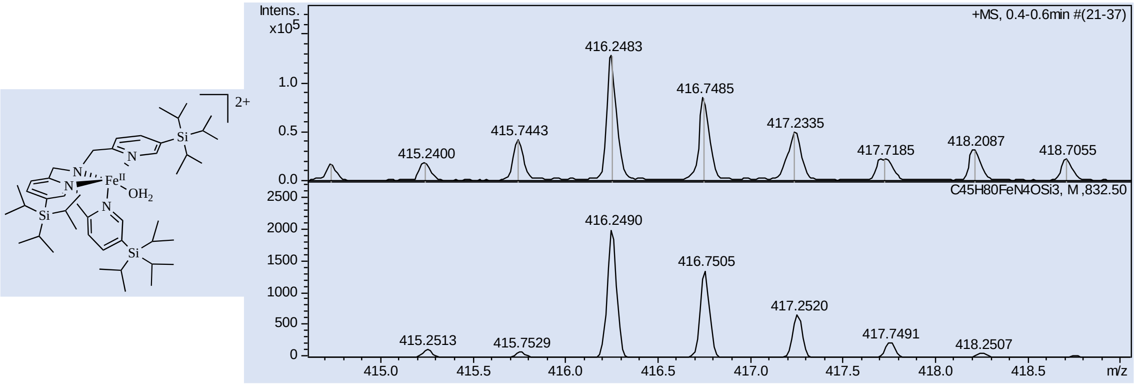

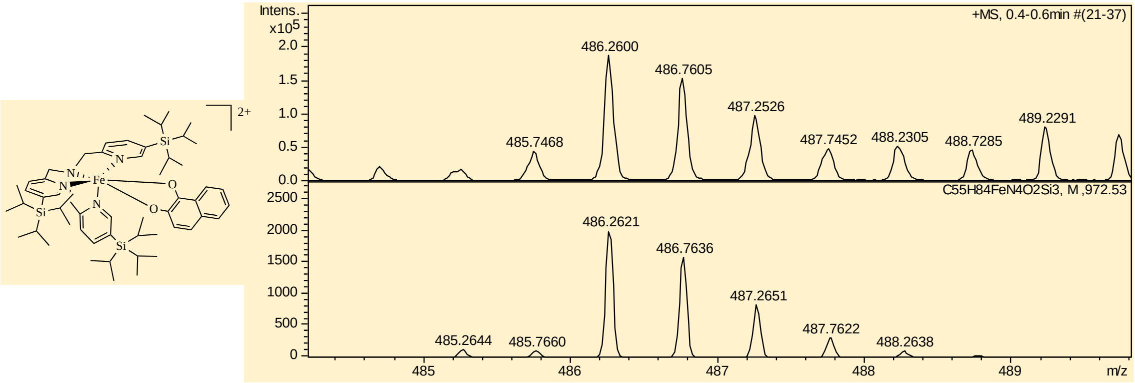

Precise electronic structure is not defined, for example a more likely structure is the ferric semiquinone.

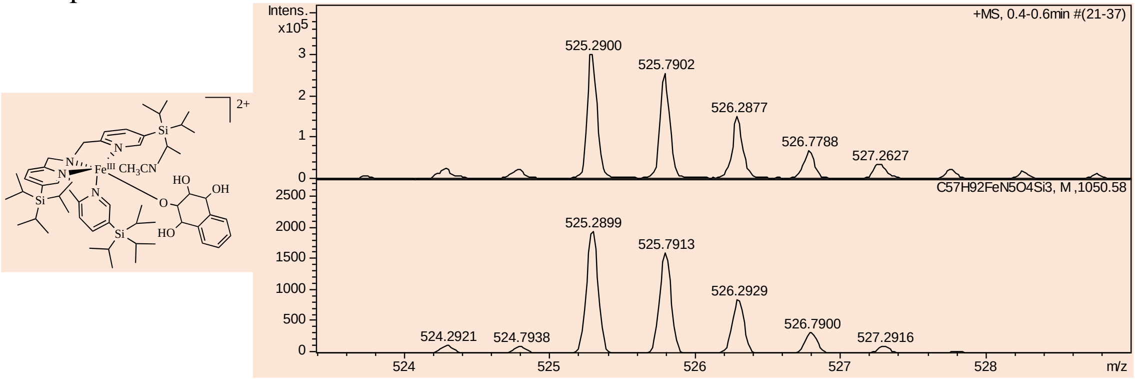

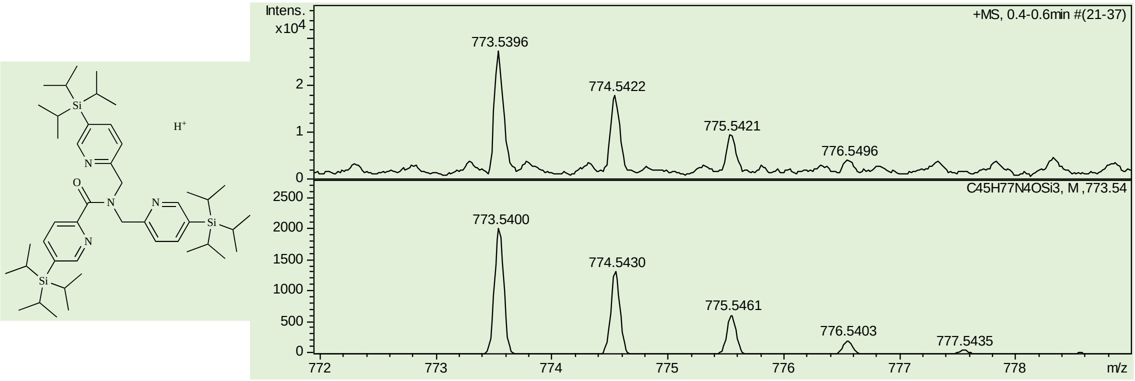

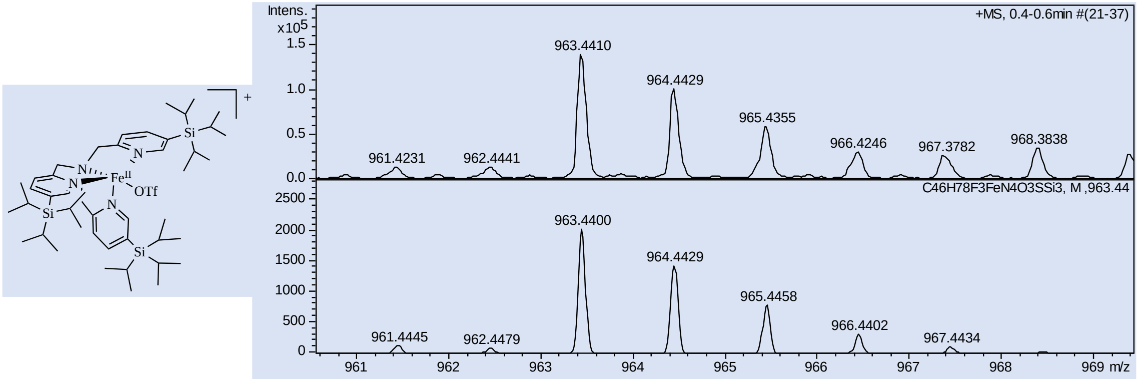

Intens. x10^5
+MS, 0.4-0.6min #(21-37)
961.4231
962.4441
963.4410
964.4429
965.4355
966.4246
967.3782
968.3838
C46H78F3FeN4O3SSi3, M ,963.44
961.4445
962.4479
963.4400
964.4429
965.4458
966.4402
967.4434
m/z
OTf
Fe^II

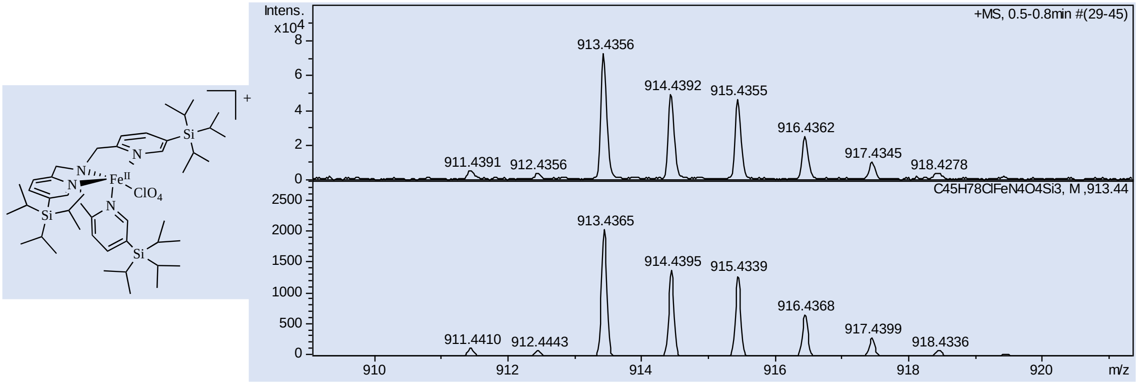

Intens. x10^4
+MS, 0.5-0.8min #(29-45)
911.4391
912.4356
913.4356
914.4392
915.4355
916.4362
917.4345
918.4278
C45H78ClFeN4O4Si3, M ,913.44
911.4410
912.4443
913.4365
914.4395
915.4339
916.4368
917.4399
918.4336
m/z
ClO4
Fe^II

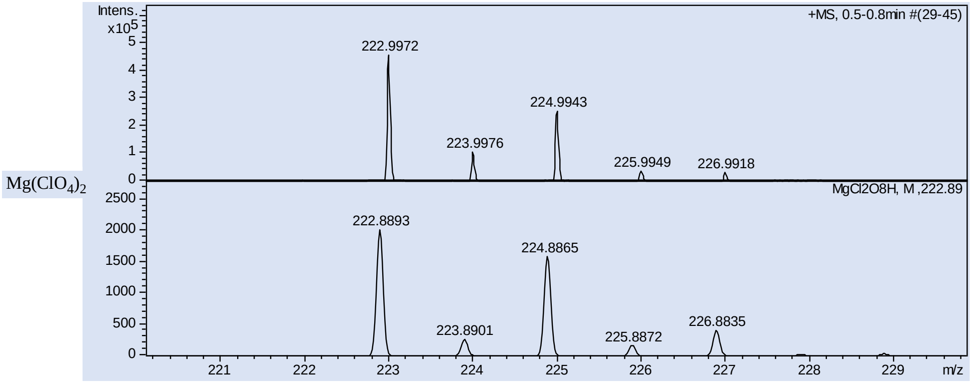

Mg(ClO4)2
Intens. x10^5
+MS, 0.5-0.8min #(29-45)
222.9972
223.9976
224.9943
225.9949
226.9918
MgCl2O8H, M ,222.89
222.8893
223.8901
224.8865
225.8872
226.8835
m/z

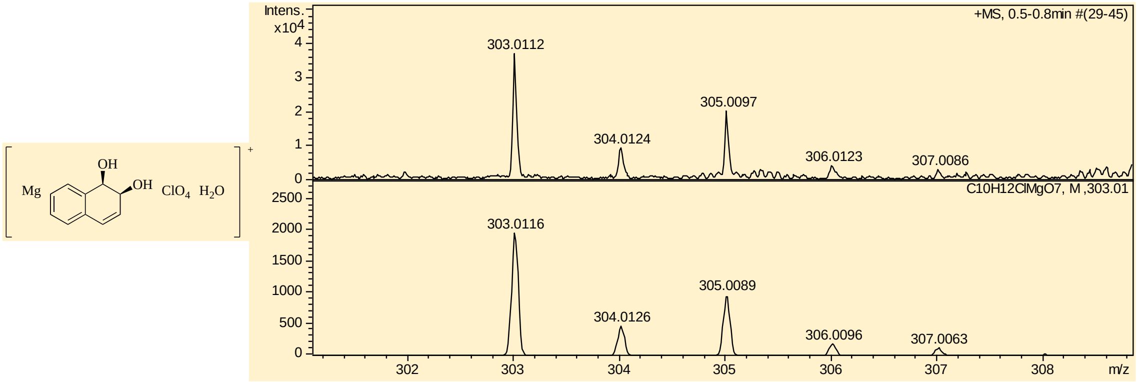

Mg
OH
OH
ClO4 H2O
Intens. x10^4
+MS, 0.5-0.8min #(29-45)
303.0112
304.0124
305.0097
306.0123
307.0086
C10H12ClMgO7, M ,303.01
303.0116
304.0126
305.0089
306.0096
307.0063
m/z

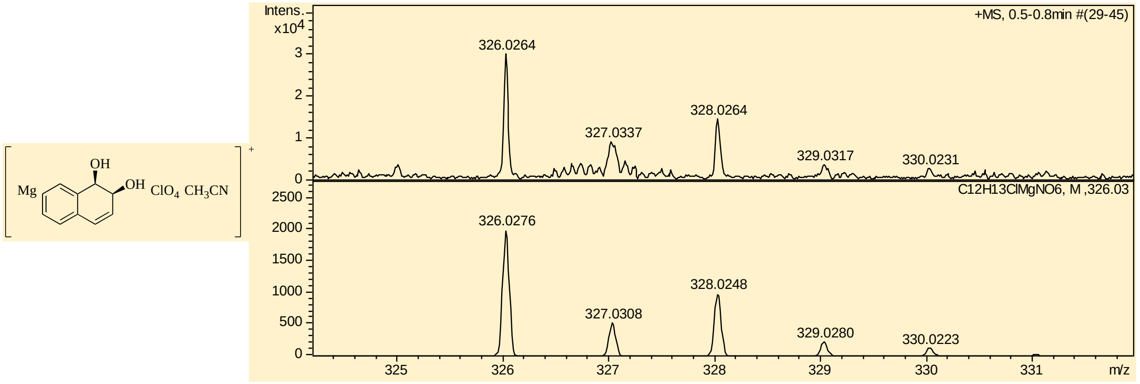

Mg
OH
OH
ClO_4 CH_3CN
Intens.
x10^4
+MS, 0.5-0.8min #(29-45)
326.0264
327.0337
328.0264
329.0317
330.0231
C12H13ClMgNO6, M ,326.03
326.0276
327.0308
328.0248
329.0280
330.0223
m/z

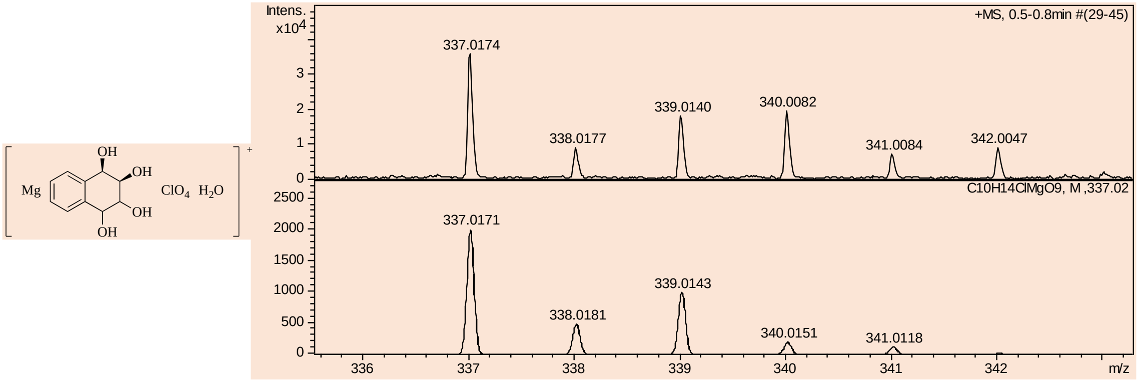

Mg
OH
OH
OH
OH
ClO_4 H_2O
Intens.
x10^4
+MS, 0.5-0.8min #(29-45)
337.0174
338.0177
339.0140
340.0082
341.0084
342.0047
C10H14ClMgO9, M ,337.02
337.0171
338.0181
339.0143
340.0151
341.0118
m/z

8. General procedure for isolation of products

8.1. Diol formation

In a 25mL round bottom flask equipped with a stir bar a solution of substrate (4.5 µmol) in 4 mL of acetonitrile was added, afterwards $Mg(ClO_4)_2 \cdot 6H_2O$ (0.656 g, 19.8 µmol, 2.2 eq.) and the catalyst (15 mg, 0.135 µmol, 3 mol%) were added. The mixture was cooled in an ice bath and a solution of H_2O_2 50% aq. (0.763 mL, 0.59 M, 1 eq.) was added via syringe pump for 30 minutes.

8.2. Tetraol formation

In a 25mL round bottom flask equipped with a stir bar a solution of substrate (4.5 µmol) in 4 mL of acetonitrile was added, afterwards $Mg(ClO_4)_2 \cdot 6H_2O$ (0.656 g, 19.8 µmol, 2.2 eq.) and the catalyst (15 mg, 0.135 µmol, 3 mol%) were added. The mixture was cooled in an ice bath and a solution of H_2O_2 50% aq. (1.145 mL, 0.59 M, 1.5 eq.) was added via syringe pump for 30 minutes.

After this, a second addition of catalyst (3 mol%) and H_2O_2 (1.5 eq.) was performed under the same conditions. If required, a third addition was performed leading to the total time of the reaction up to 1.5h.

8.3. Workup for the isolation of products

After the reaction was finished, 1mL of methyl imidazole and 10mL of acetic anhydride were added and stirred for 45 minutes at room temperature. Then ice was added, and the solution was stirred till the ice dissolved.

A first extraction with dichloromethane was performed, followed by an extraction with H_2SO_4 1M of the organic solution. Afterwards the organic phase was extracted with $NaHCO_3$ sat., subsequently cleaned with water, and finally dried with $MgSO_4$. The crude of the reaction was purified by flash chromatography on silica gel and the purity of the products obtained was checked by ^{1}H-NMR, ^{13}C-NMR and HRMS-TOF.

9. Characterization of isolated products

(1α,2α)-1,2-dihydronaphthalene-1,2-diyl diacetate (2a)

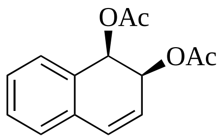

Purification by flash chromatography on silica gel (SiO_2; Hexane:AcOEt, 95:5) gave the product as a colorless oil (28% yield).

^{1}H NMR (400 MHz, Chloroform-*d*) δ 7.37-7.26 (m, 3H), 7.17 (dd, *J* = 7.4, 1.4 Hz, 1H), 6.64 (dd, *J* = 9.7, 1.5 Hz, 1H), 6.10 (d, *J* = 4.7 Hz, 1H), 5.96 (dd, *J* = 9.7, 3.9 Hz, 1H), 5.69 (ddd, *J* = 5.3, 3.9, 1.5 Hz, 1H), 2.12 (s, 3H), 2.05 (s, 3H).

^{13}C NMR (101 MHz, Chloroform-*d*) δ 170.66, 170.64, 132.56, 131.39, 130.31, 129.21, 128.40, 127.49, 127.27, 124.87, 69.68, 68.01, 21.15, 21.12.

HRMS(ESI+) m/z calculated $[M+Na]^+$ 269.0784, found 269.0782.

(1α,2α)-3,7-dimethyl-1,2-dihydronaphthalene-1,2-diyl diacetate (2b)

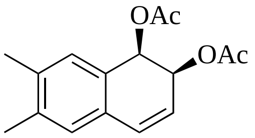

Purification by flash chromatography on silica gel (SiO_2; Hexane:AcOEt, 95:5) gave the product as a yellow oil (18% yield).

^{1}H NMR (400 MHz, Chloroform-*d*) δ 7.11 (s, 1H), 6.95 (s, 1H), 6.57 (dd, *J* = 9.7, 1.7 Hz, 1H), 6.05 (d, *J* = 4.8 Hz, 1H), 5.86 (dd, *J* = 9.7, 3.6 Hz, 1H), 5.67 (ddd, *J* = 5.1, 3.6, 1.7 Hz, 1H), 2.26 (s, 3H), 2.25 (s, 3H), 2.10 (s, 3H), 2.05 (s, 3H).

^{13}C NMR (101 MHz, Chloroform-*d*) δ 170.73, 170.68, 137.67, 136.93, 130.29, 129.87, 129.18, 128.62, 124.08, 69.53, 68.66, 21.22, 21.14, 19.81, 19.66.

HRMS(ESI+) m/z calculated $[M+Na]^+$ 297.1097, found 297.1099.

(1α,2α)-6,7-dimethyl-1,2-dihydronaphthalene-1,2-diyl diacetate (2c)

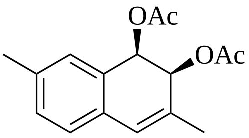

Purification by flash chromatography on silica gel (SiO_2; Hexane:AcOEt, 95:5) gave the product as a pale yellow oil (21% yield).

^{1}H NMR (400 MHz, Chloroform-*d*) δ 7.12-7.07 (m, 2H), 6.99 (d, *J* = 7.5 Hz, 1H), 6.38 (s, 1H), 6.07 (d, *J* = 4.7 Hz, 1H), 5.64 (dt, *J* = 4.8, 0.9 Hz, 1H), 2.34 (s, 3H), 2.13 (s, 3H), 2.05 (s, 3H), 1.92 (s, 3H).

^{13}C NMR (101 MHz, Chloroform-*d*) δ 171.01, 170.74, 137.34, 131.98, 130.46, 130.37, 129.42, 127.17, 126.32, 126.13, 70.48, 70.24, 21.50, 21.17, 21.04, 20.02.

HRMS(ESI+) m/z calculated $[M+Na]^+$ 297.1097, found 297.1107.

(1α,2α)-1,2-dihydronaphthalene-1,2,6,7-tetrayl tetraacetate (2d)

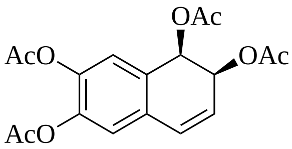

Purification by flash chromatography on silica gel (SiO_2; Hexane:AcOEt, 9:1) gave the product as a white solid (16% yield).

^{1}H NMR (400 MHz, Chloroform-*d*) δ 7.19 (t, *J* = 0.7 Hz, 1H), 7.02 (s, 1H), 6.60-6.55 (m, 1H), 6.05-5.98 (m, 2H), 5.66 (td, *J* = 4.5, 1.3 Hz, 1H), 2.29 (s, 6H), 2.11 (s, 3H), 2.04 (s, 3H).

^{13}C NMR (101 MHz, Chloroform-*d*) δ 170.56, 170.43, 168.28, 168.26, 142.44, 141.58, 131.27, 130.11, 129.22, 125.57, 122.68, 122.14, 69.02, 66.97, 21.08, 21.04, 20.79, 20.72.

HRMS(ESI+) m/z calculated $[M+Na]^+$ 385.0894, found 385.0899.

X-Ray: CCDC (2281990)

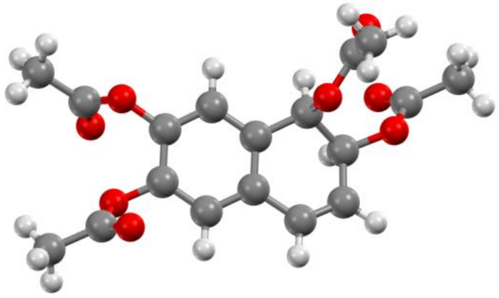

(1α,2α)-7-formyl-1,2-dihydronaphthalene-1,2-diyl diacetate (2s)

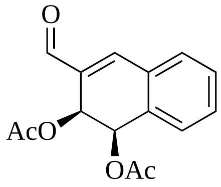

Purification by flash chromatography on silica gel (SiO_2; Hexane:AcOEt, 9:1) gave the product as a pale orange solid (11% yield).

^{1}H NMR (400 MHz, Chloroform-*d*) δ 9.71 (s, 1H), 7.52 (s, 1H), 7.51-7.39 (m, 4H), 6.25 (d, *J* = 4.8 Hz, 1H), 6.12 (d, *J* = 4.7 Hz, 1H), 2.21 (s, 3H), 1.95 (s, 3H).

^{13}C NMR (101 MHz, Chloroform-*d*) δ 190.39, 170.39, 170.16, 146.85, 134.78, 134.06, 132.11, 130.20, 130.09, 128.67, 125.36, 70.32, 61.21, 20.98, 20.90.

HRMS(ESI+) m/z calculated [M+Na]$^+$ 297.0733, found 297.0730.

(1α,2α)-3,7-dibromo-1,2-dihydronaphthalene-1,2-diyl diacetate (2e)

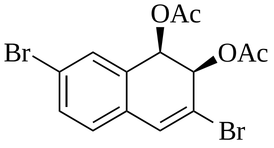

Purification by flash chromatography on silica gel (SiO_2; Hexane:AcOEt, 98:2) gave the product as a pale orange oil (14% yield).

^{1}H NMR (400 MHz, Chloroform-*d*) δ 7.47-7.43 (m, 2H), 7.04-6.99 (m, 2H), 6.17 (dt, *J* = 5.1, 1.0 Hz, 1H), 5.85 (d, *J* = 5.0 Hz, 1H), 2.17 (s, 3H), 2.07 (s, 3H).

^{13}C NMR (101 MHz, Chloroform-*d*) δ 170.30, 170.14, 132.89, 132.29, 131.90, 130.67, 128.88, 128.17, 122.76, 119.24, 70.35, 69.88, 20.92, 20.81.

HRMS(ESI+) m/z calculated $[M+Na]^+$ 426.8979, found 426.8975.

1,2,3,4-tetrahydronaphthalene-1,2,3,4-tetraol (3a)

Purification by flash chromatography on silica gel (SiO_2; Hexane:AcOEt, 9:1) gave the products in a 30% yield, as a white solid for both isomers.

(1α,2α,3α,4α)-1,2,3,4-tetrahydronaphthalene-1,2,3,4-tetraol ($3a^{syn}$)

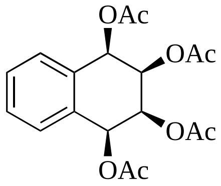

^{1}H NMR (400 MHz, Chloroform-*d*) δ 7.43-7.37 (m, 2H), 7.35-7.28 (m, 2H), 6.27-6.21 (m, 2H), 5.61-5.53 (m, 2H), 2.14 (s, 6H), 2.06 (s, 6H).

^{13}C NMR (101 MHz, Chloroform-*d*) δ 170.53, 170.14, 132.02, 129.32, 128.42, 67.93, 67.60, 20.95, 20.83.

HRMS(ESI+) m/z calculated $[M+Na]^+$ 387.1050, found 387.1065.

X-Ray: CCDC (2281988)

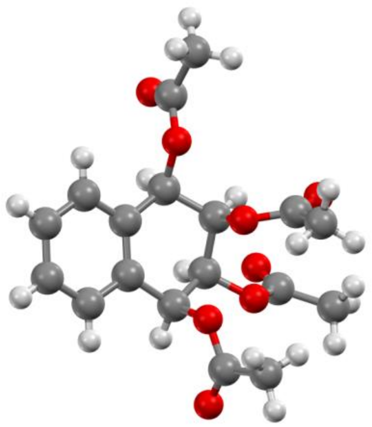

(1α,2α,3β,4β)-1,2,3,4-tetrahydronaphthalene-1,2,3,4-tetraol (3b^{anti})

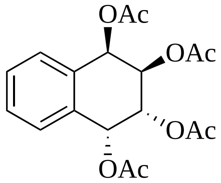

^{1}H NMR (400 MHz, Chloroform-*d*) δ 7.41 (dd, *J* = 5.7, 3.4 Hz, 2H), 7.32 (dd, *J* = 5.8, 3.4 Hz, 2H), 6.43 (dd, *J* = 2.1, 1.3 Hz, 2H), 5.70 (dd, *J* = 2.2, 1.3 Hz, 2H), 2.08 (s, 6H), 2.06 (s, 6H).

^{13}C NMR (101 MHz, Chloroform-*d*) δ 170.47, 170.22, 132.81, 130.29, 130.16, 69.35, 67.23, 21.08, 20.87.

HRMS(ESI+) m/z calculated [M+Na]$^{+}$ 387.1040, found 387.1050.

X-Ray: CCDC (2281984)

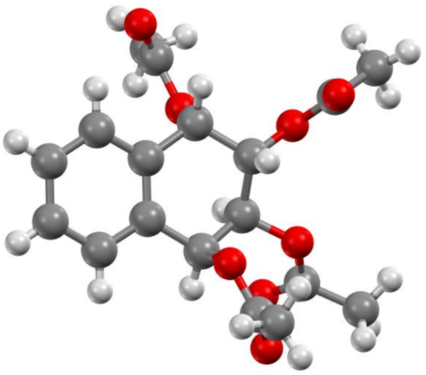

6,7-dimethyl-1,2,3,4-tetrahydronaphthalene-1,2,3,4-tetraol (3b)

Purification by flash chromatography on silica gel (SiO_2; Hexane:AcOEt, 9:1) gave the products in a 37% yield, as a white solid for isomer *syn* and an orange solid for isomer *anti*.

(1α,2α,3α,4α)-6,7-dimethyl-1,2,3,4-tetrahydronaphthalene-1,2,3,4-tetraol (3b^{syn})

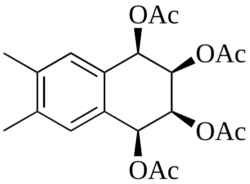

^{1}H NMR (400 MHz, Chloroform-*d*) δ 7.05 (s, 2H), 6.22-6.17 (m, 2H), 5.57-5.50 (m, 2H), 2.26 (s, 6H), 2.14 (s, 6H), 2.06 (s, 6H).

^{13}C NMR (101 MHz, Chloroform-*d*) δ 170.65, 170.20, 138.32, 129.30, 129.25, 67.90, 67.78, 21.08, 20.89, 19.75.

HRMS(ESI+) m/z calculated $[M+Na]^{+}$ 415.1363, found 415.1368.

(1α,2α,3β,4β)-6,7-dimethyl-1,2,3,4-tetrahydronaphthalene-1,2,3,4-tetraol (3b^{anti})

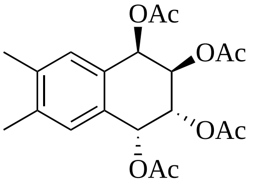

^{1}H NMR (400 MHz, Chloroform-*d*) δ 7.07 (s, 2H), 6.37 (t, *J* = 1.7 Hz, 2H), 5.67 (dd, *J* = 2.2, 1.3 Hz, 2H), 2.25 (s, 6H), 2.07 (s, 6H), 2.05 (s, 6H).

^{13}C NMR (101 MHz, Chloroform-*d*) δ 170.58, 170.23, 139.30, 131.22, 130.11, 69.36, 67.33, 21.18, 20.88, 19.73.

HRMS(ESI+) m/z calculated $[M+Na]^{+}$ 415.1363, found 415.1363.

3-hydroxy-3,7-dimethyl-1,2,3,4-tetrahydronaphthalene-1,2,4-triyl triacetate (3c)

Purification by flash chromatography on silica gel (SiO_2; Hexane:AcOEt, 8.5:1.5) gave the products in a 39% yield, as a beige solid for isomer *syn* and a pale orange solid for isomer *anti*.

(1α,2α,3α,4α)-3-hydroxy-3,7-dimethyl-1,2,3,4-tetrahydronaphthalene-1,2,4-triyl triacetate (3c^{syn})

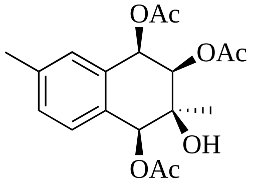

^{1}H NMR (400 MHz, Chloroform-*d*) δ 7.22-7.17 (m, 1H), 7.14 (d, *J* = 8.0 Hz, 1H), 7.09 (d, *J* = 1.7 Hz, 1H), 6.26 (d, *J* = 4.6 Hz, 1H), 5.97 (s, 1H), 5.33 (d, *J* = 4.6 Hz, 1H), 2.85 (s, 1H), 2.34 (s, 3H), 2.26 (s, 3H), 2.15 (s, 3H), 2.12 (s, 3H), 1.32 (s, 3H).

^{13}C NMR (101 MHz, Chloroform-*d*) δ 171.11, 170.38, 170.34, 139.22, 130.99, 130.68, 130.29, 129.35, 128.38, 74.25, 72.33, 72.12, 68.79, 22.34, 21.26, 21.21, 21.16, 20.84.

HRMS(ESI+) m/z calculated $[M+Na]^+$ 373.1258, found 373.1255.

(1α,2α,3β,4β)-3-hydroxy-3,7-dimethyl-1,2,3,4-tetrahydronaphthalene-1,2,4-triyl triacetate (3c^{anti})

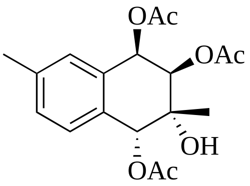

^{1}H NMR (400 MHz, Chloroform-*d*) δ 7.18-7.15 (m, 2H), 7.06 (s, 1H), 6.43 (d, *J* = 4.5 Hz, 1H), 6.08 (s, 1H), 5.61 (d, *J* = 4.5 Hz, 1H), 2.34 (s, 3H), 2.19 (s, 3H), 2.12 (s, 3H), 2.09 (s, 3H), 1.33 (s, 3H).

^{13}C NMR (101 MHz, Chloroform-*d*) δ 170.84, 170.74, 170.63, 139.17, 132.67, 130.47, 129.97, 128.99, 128.84, 72.41, 72.39, 68.69, 22.37, 21.33, 21.30, 21.11, 21.01.

HRMS(ESI+) m/z calculated $[M+Na]^+$ 373.1258, found 373.1257.

6-ethyl-1,2,3,4-tetrahydronaphthalene-1,2,3,4-tetrayl tetraacetate (3g)

Purification by flash chromatography on silica gel (SiO_2; Hexane:AcOEt, 9:1) gave the products in a 35% yield, as a white solid for isomer *syn* and a pale orange solid for isomer *anti*.

(1α,2α,3α,4α)-6-ethyl-1,2,3,4-tetrahydronaphthalene-1,2,3,4-tetrayl tetraacetate ($3g^{syn}$)

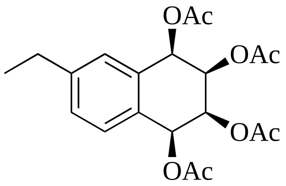

^{1}H NMR (400 MHz, Chloroform-*d*) δ 7.23 (d, *J* = 1.2 Hz, 2H), 7.11 (s, 1H), 6.25-6.19 (m, 2H), 5.56 (td, *J* = 4.3, 3.8, 2.3 Hz, 2H), 2.66 (q, *J* = 7.6 Hz, 2H), 2.15 (s, 3H), 2.13 (s, 3H), 2.07 (s, 3H), 2.06 (s, 3H), 1.23 (t, *J* = 7.6 Hz, 3H).

^{13}C NMR (101 MHz, Chloroform-*d*) δ 170.64, 170.22, 170.20, 145.72, 131.87, 129.28, 129.14, 128.58, 127.64, 68.10, 67.91, 67.76, 67.72, 28.70, 21.06, 21.04, 20.90, 20.89, 15.45.

HRMS(ESI+) m/z calculated $[M+Na]^+$ 415.1377, found 415.1363.

(1α,2α,3β,4β)-6-ethyl-1,2,3,4-tetrahydronaphthalene-1,2,3,4-tetrayl tetraacetate ($3g^{anti}$)

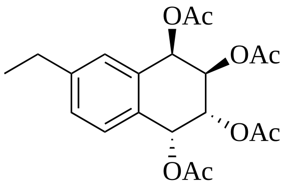

^{1}H NMR (400 MHz, Chloroform-*d*) δ 7.24 (s, 2H), 7.13 (s, 1H), 6.41 (t, *J* = 1.8 Hz, 2H), 5.68 (dd, *J* = 2.1, 1.3 Hz, 2H), 2.65 (q, *J* = 7.6 Hz, 3H), 2.09 (s, 3H), 2.07 (s, 3H), 2.06 (d, *J* = 0.8 Hz, 6H), 1.22 (t, *J* = 7.6 Hz, 3H).

^{13}C NMR (101 MHz, Chloroform-*d*) δ 170.57, 170.54, 170.23, 146.68, 132.67, 130.36, 130.09, 129.96, 129.59, 69.51, 69.31, 67.31, 67.28, 28.66, 21.15, 21.13, 20.88, 15.38.

HRMS(ESI+) m/z calculated $[M+Na]^+$ 415.1364, found 415.1363.

6-cyano-1,2,3,4-tetrahydronaphthalene-1,2,3,4-tetrayl tetraacetate (3j)

Purification by flash chromatography on silica gel (SiO_2; Hexane:AcOEt, 9:1) gave the products in a 69% yield, as a white solid for both isomers.

(1α,2α,3α,4α)-6-cyano-1,2,3,4-tetrahydronaphthalene-1,2,3,4-tetrayl tetraacetate ($3j^{syn}$)

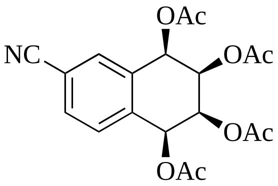

^{1}H NMR (400 MHz, Chloroform-*d*) δ 7.66 (dd, *J* = 8.1, 1.6 Hz, 1H), 7.63 (t, *J* = 1.3 Hz, 1H), 7.44 (d, *J* = 8.1 Hz, 1H), 6.24 (d, *J* = 4.4 Hz, 1H), 6.21 (d, *J* = 4.3 Hz, 1H), 5.63-5.53 (m, 2H), 2.17 (s, 3H), 2.15 (s, 3H), 2.06 (s, 6H).

^{13}C NMR (101 MHz, Chloroform-*d*) δ 170.25, 169.97, 169.95, 137.23, 133.86, 132.40, 132.34, 129.47, 117.97, 113.51, 67.33, 67.21, 67.07, 20.85, 20.83, 20.75.

HRMS(ESI+) m/z calculated $[M+Na]^+$ 412.1003, found 412.0996.

X-Ray: CCDC (2282085)

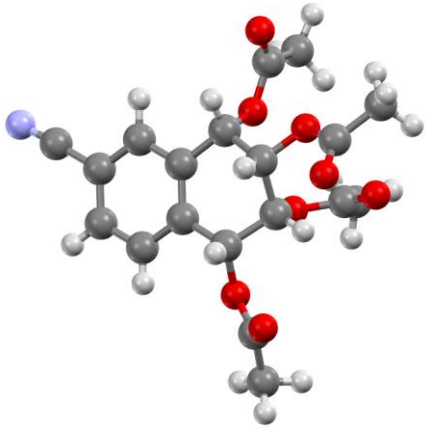

(1α,2α,3β,4β)-6-cyano-1,2,3,4-tetrahydronaphthalene-1,2,3,4-tetrayl tetraacetate ($3j^{anti}$)

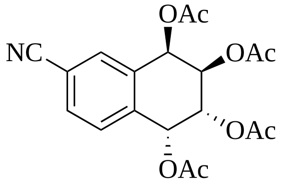

^{1}H NMR (400 MHz, Chloroform-*d*) δ 7.67 (dd, *J* = 8.0, 1.7 Hz, 1H), 7.63 (d, *J* = 1.6 Hz, 1H), 7.44 (d, *J* = 8.0 Hz, 1H), 6.40 (t, *J* = 1.7 Hz, 1H), 6.38 (t, *J* = 1.8 Hz, 1H), 5.66 (dd, *J* = 2.3, 1.2 Hz, 2H), 2.12 (s, 3H), 2.10 (s, 3H), 2.07 (s, 6H).

^{13}C NMR (101 MHz, Chloroform-*d*) δ 170.16, 170.15, 169.94, 169.92, 137.59, 134.31, 133.41, 132.93, 130.52, 117.78, 113.92, 68.16, 67.93, 66.98, 66.95, 20.91, 20.89, 20.77.

HRMS(ESI+) m/z calculated $[M+Na]^+$ 412.1003, found 412.0997.

5-cyano-1,2,3,4-tetrahydronaphthalene-1,2,3,4-tetrayl tetraacetate (3q)

Purification by flash chromatography on silica gel (SiO_2; Hexane:AcOEt, 9:1) gave the products in a 44% yield, as a white solid for isomer *syn* and a pale yellow solid for isomer *anti.*

(1α,2α,3α,4α)-5-cyano-1,2,3,4-tetrahydronaphthalene-1,2,3,4-tetrayl tetraacetate ($3q^{syn}$)

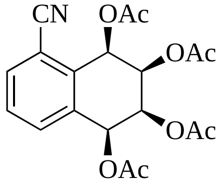

^{1}H NMR (400 MHz, Chloroform-*d*) δ 7.78-7.72 (m, 1H), 7.60-7.49 (m, 2H), 6.43 (d, *J* = 5.1 Hz, 1H), 6.14 (d, *J* = 3.9 Hz, 1H), 5.70-5.64 (m, 1H), 5.53 (dd, *J* = 5.1, 2.0 Hz, 1H), 2.17 (s, 3H), 2.16 (s, 3H), 2.07 (s, 3H), 2.06 (s, 3H).

^{13}C NMR (101 MHz, Chloroform-*d*) δ 170.26, 170.23, 169.85, 169.74, 135.51, 134.46, 134.31, 131.86, 129.82, 116.61, 113.80, 68.22, 67.35, 66.35, 65.46, 20.86, 20.71, 20.33.

HRMS(ESI+) m/z calculated $[M+Na]^+$ 412.1003, found 412.0993.

X-Ray: CCDC (2281985)

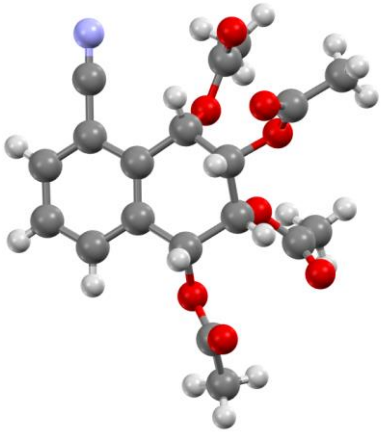

(1α,2α,3β,4β)-5-cyano-1,2,3,4-tetrahydronaphthalene-1,2,3,4-tetrayl tetraacetate ($3q^{anti}$)

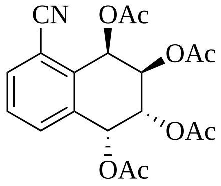

^{1}H NMR (400 MHz, Chloroform-*d*) δ 7.76 (dd, *J* = 7.4, 1.6 Hz, 1H), 7.60-7.50 (m, 2H), 6.61 (d, *J* = 4.2 Hz, 1H), 6.41 (d, *J* = 3.8 Hz, 1H), 5.74 (dd, *J* = 10.9, 4.2 Hz, 1H), 5.61 (dd, *J* = 10.9, 3.8 Hz, 1H), 2.13 (s, 3H), 2.10 (s, 3H), 2.07 (s, 3H), 2.06 (s, 3H).

^{13}C NMR (101 MHz, Chloroform-*d*) δ 170.26, 170.11, 170.05, 169.61, 136.24, 134.78, 134.62, 130.38, 116.13, 114.08, 68.44, 66.82, 66.52, 66.17, 20.97, 20.81, 20.78, 20.47.

HRMS(ESI+) m/z calculated $[M+Na]^{+}$ 412.1003, found 412.1006.

X-Ray: CCDC (2281986)

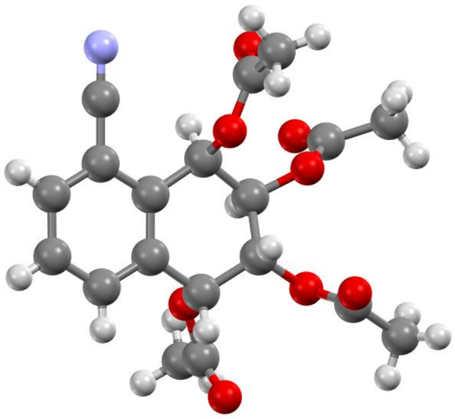

6-phenyl-1,2,3,4-tetrahydronaphthalene-1,2,3,4-tetrayl tetraacetate (3h)

Purification by flash chromatography on silica gel (SiO_2; Hexane:AcOEt, 9:1) gave the products in a 40% yield, as a pale beige solid for isomer *syn* and a beige solid for isomer *anti*.

(1α,2α,3α,4α)-6-phenyl-1,2,3,4-tetrahydronaphthalene-1,2,3,4-tetrayl tetraacetate (3h^{syn})

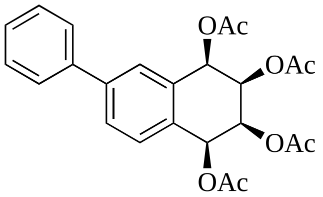

^{1}H NMR (400 MHz, Chloroform-*d*) δ 7.65-7.33 (m, 9H), 6.33 – 6.28 (m, 2H), 5.64-5.58 (m, 2H), 2.16 (s, 3H), 2.15 (s, 3H), 2.08 (s, 6H).

^{13}C NMR (101 MHz, Chloroform-*d*) δ 170.62, 170.59, 170.18, 142.49, 140.06, 132.51, 130.93, 129.05, 128.98, 128.24, 128.04, 127.30, 127.07, 68.05, 67.91, 67.67, 21.04, 21.03, 20.89.

HRMS(ESI+) m/z calculated [M+Na]$^{+}$ 463.1363, found 463.1360.

(1α,2α,3β,4β)-6-phenyl-1,2,3,4-tetrahydronaphthalene-1,2,3,4-tetrayl tetraacetate (3h^{anti})

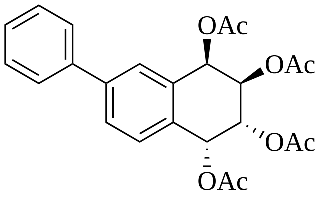

^{1}H NMR (400 MHz, Chloroform-*d*) δ 7.69 -7.33 (m, 9H), 6.49 (d, *J* = 10.2 Hz, 2H), 5.74 (s, 2H), 2.12 -2.05 (m, 12H).

^{13}C NMR (101 MHz, Chloroform-*d*) δ 170.20, 130.78, 129.08, 128.89, 128.82, 128.17, 127.27, 69.42, 69.15, 67.26, 67.24, 21.10, 20.86.

HRMS(ESI+) m/z calculated [M+Na]$^{+}$ 463.1363, found 463.1361.

(1α,2α,3α,4α)-5,8-dicyano-1,2,3,4-tetrahydronaphthalene-1,2,3,4-tetrayl tetraacetate (3r)

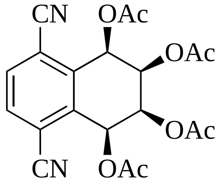

Purification by crystallization with hot ethyl acetate gave the product in a 34% yield, as pale pink crystals.

^{1}H NMR (400 MHz, Chloroform-*d*) δ 7.87 (s, 2H), 6.38-6.35 (m, 2H), 5.61-5.58 (m, 2H), 2.19 (s, 6H), 2.08 (s, 6H).

^{13}C NMR (101 MHz, Chloroform-*d*) δ 169.78, 169.62, 137.87, 134.82, 117.45, 115.50, 66.03, 65.98, 20.71, 20.18.

HRMS(ESI+) m/z calculated [M+Na]$^{+}$ 437.0955, found 437.0952.

X-Ray: CCDC (2281987)

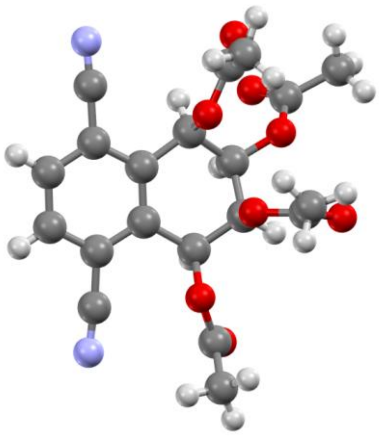

6-bromo-1,2,3,4-tetrahydronaphthalene-1,2,3,4-tetrayl tetraacetate (3l)

Purification by flash chromatography on silica gel (SiO_2; Hexane:AcOEt, 9:1) gave the products in a 51% yield, as a white solid for both isomers.

(1α,2α,3α,4α)-6-bromo-1,2,3,4-tetrahydronaphthalene-1,2,3,4-tetrayl tetraacetate (3l^{syn})

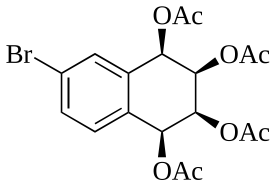

^{1}H NMR (400 MHz, Chloroform-*d*) δ 7.48 (dd, *J* = 8.3, 2.1 Hz, 1H), 7.43 (d, *J* = 2.0 Hz, 1H), 7.17 (d, *J* = 8.4 Hz, 1H), 6.16 (dd, *J* = 6.7, 4.5 Hz, 2H), 5.55 (dd, *J* = 4.5, 2.0 Hz, 1H), 5.51 (dd, *J* = 4.5, 2.0 Hz, 1H), 2.14 (s, 3H), 2.11 (s, 3H), 2.04 (s, 6H).

^{13}C NMR (101 MHz, Chloroform-*d*) δ 170.38, 170.28, 170.02, 169.93, 134.21, 132.44, 131.11, 131.02, 130.29, 123.35, 67.51, 67.33, 67.30, 67.22, 20.86, 20.75, 20.72.

HRMS(ESI+) m/z calculated $[M+Na]^{+}$ 465.0156, found 465.0150.

X-Ray: CCDC (2281980)

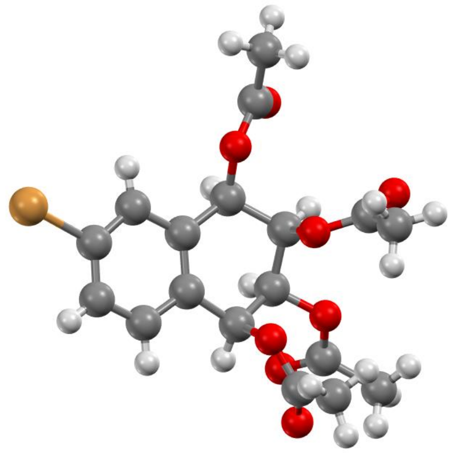

(1α,2α,3β,4β)-6-bromo-1,2,3,4-tetrahydronaphthalene-1,2,3,4-tetrayl tetraacetate ($3l^{anti}$)

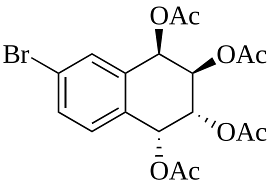

^{1}H NMR (400 MHz, Chloroform-*d*) δ 7.51 (dd, *J* = 8.3, 2.1 Hz, 1H), 7.46 (d, *J* = 2.0 Hz, 1H), 7.19 (d, *J* = 8.3 Hz, 1H), 6.37-6.32 (m, 2H), 5.66-5.62 (m, 2H), 2.09 (s, 3H), 2.07 (s, 3H), 2.05 (s, 6H).

^{13}C NMR (101 MHz, Chloroform-*d*) δ 169.32, 169.25, 169.07, 169.04, 133.78, 132.24, 131.85, 130.75, 130.67, 123.03, 67.61, 67.46, 66.03, 65.93, 19.99, 19.79.

HRMS(ESI+) m/z calculated [M+Na]$^{+}$ 465.0156, found 465.0155.

X-Ray: CCDC (2281989)

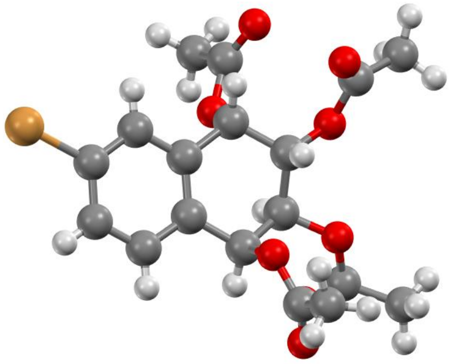

6-chloro-1,2,3,4-tetrahydronaphthalene-1,2,3,4-tetrayl tetraacetate (3m)

Purification by flash chromatography on silica gel (SiO_2; Hexane:AcOEt, 9:1) gave the products in a 32% yield, as a white solid for both isomers.

(1α,2α,3α,4α)-6-chloro-1,2,3,4-tetrahydronaphthalene-1,2,3,4-tetrayl tetraacetate ($3m^{syn}$)

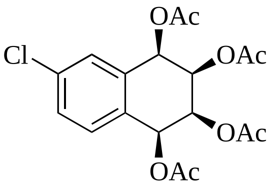

^{1}H NMR (400 MHz, Chloroform-*d*) δ 7.38-7.34 (m, 1H), 7.30 (d, *J* = 1.9 Hz, 1H), 7.27 (s, 1H), 6.21 (d, *J* = 4.5 Hz, 1H), 6.17 (d, *J* = 4.4 Hz, 1H), 5.60-5.56 (m, 1H), 5.53 (dd, *J* = 4.6, 2.0 Hz, 1H), 2.16 (s, 3H), 2.13 (s, 3H), 2.06 (s, 6H).

^{13}C NMR (101 MHz, Chloroform-*d*) δ 170.48, 170.37, 170.13, 170.02, 135.44, 134.03, 130.62, 130.21, 129.65, 128.13, 67.68, 67.41, 67.34, 20.96, 20.94, 20.84, 20.81.

HRMS(ESI+) m/z calculated $[M+Na]^+$ 421.0661, found 421.0669.

(1α,2α,3β,4β)-6-chloro-1,2,3,4-tetrahydronaphthalene-1,2,3,4-tetrayl tetraacetate ($3m^{anti}$)

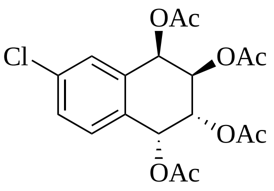

^{1}H NMR (400 MHz, Chloroform-*d*) δ 7.37 (dd, *J* = 8.2, 2.2 Hz, 1H), 7.31 (d, *J* = 2.2 Hz, 1H), 7.28 (s, 1H), 6.37 (dd, *J* = 4.8, 2.8 Hz, 2H), 5.66 (dt, *J* = 2.2, 0.8 Hz, 2H), 2.10 (s, 3H), 2.08 (s, 3H), 2.06 (s, 6H).

^{13}C NMR (101 MHz, Chloroform-*d*) δ 170.37, 170.29, 170.12, 170.09, 136.00, 134.61, 131.54, 131.28, 130.37, 129.94, 68.61, 68.57, 67.10, 66.97, 21.04, 21.02, 20.83.

HRMS(ESI+) m/z calculated $[M+Na]^+$ 421.0661, found 421.0677.

1,2,3,4-tetrahydronaphthalene-1,2,3,4,6-pentayl pentaacetate (3f)

Purification by flash chromatography on silica gel (SiO_2; Hexane:AcOEt, 9:1) gave the products in a 54% yield, as a pale yellow solid for isomer *syn* and pale beige solid for isomer *anti*.

(1α,2α,3α,4α)-1,2,3,4-tetrahydronaphthalene-1,2,3,4,6-pentayl pentaacetate ($3f^{syn}$)

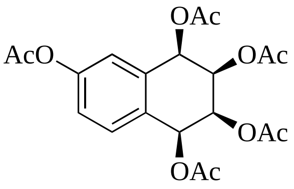

^{1}H NMR (400 MHz, Chloroform-*d*) δ 7.34 (d, *J* = 8.5 Hz, 1H), 7.13 (dd, *J* = 8.5, 2.4 Hz, 1H), 7.04 (d, *J* = 2.4 Hz, 1H), 6.24 (d, *J* = 4.6 Hz, 1H), 6.18 (d, *J* = 4.4 Hz, 1H), 5.59 (dd, *J* = 4.5, 2.0 Hz, 1H), 5.53 (dd, *J* = 4.6, 2.0 Hz, 1H), 2.31 (s, 3H), 2.14 (s, 3H), 2.13 (s, 3H), 2.07 (s, 6H).

^{13}C NMR (101 MHz, Chloroform-*d*) δ 170.54, 170.46, 170.21, 170.07, 169.34, 151.30, 133.86, 130.14, 129.64, 122.95, 121.06, 67.92, 67.48, 67.45, 67.42, 21.24, 20.99, 20.97, 20.87, 20.84.

HRMS(ESI+) m/z calculated $[M+Na]^+$ 445.1105, found 445.1102.

(1α,2α,3β,4β)-1,2,3,4-tetrahydronaphthalene-1,2,3,4,6-pentayl pentaacetate ($3f^{anti}$)

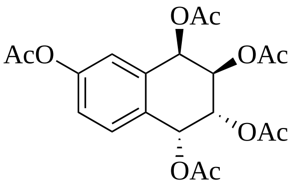

^{1}H NMR (400 MHz, Chloroform-*d*) δ 7.35 (d, *J* = 8.4 Hz, 1H), 7.14 (dd, *J* = 8.4, 2.4 Hz, 1H), 7.06 (d, *J* = 2.4 Hz, 1H), 6.40 (d, *J* = 2.6 Hz, 1H), 6.38 (d, *J* = 2.6 Hz, 1H), 5.68 (dd, *J* = 2.6, 1.1 Hz, 2H), 2.30 (s, 3H), 2.09 (s, 3H), 2.08 (s, 3H), 2.06 (s, 3H), 2.06 (s, 3H).

^{13}C NMR (101 MHz, Chloroform-*d*) δ 170.45, 170.40, 170.14, 170.10, 169.16, 151.67, 134.41, 131.53, 130.35, 123.67, 122.97, 68.85, 68.75, 67.20, 67.02, 21.20, 21.08, 21.06, 20.86, 20.85.

HRMS(ESI+) m/z calculated $[M+Na]^+$ 445.1105, found 445.1101.

1,2,3,4-tetrahydronaphthalene-1,2,3,4,6,7-hexayl hexaacetate (3d)

Purification by flash chromatography on silica gel (SiO_2; Hexane:AcOEt, 8.5:1.5) gave the products in a 75% yield, as a beige solid for isomer *syn* and white solid for isomer *anti.*

(1α,2α,3α,4α)-1,2,3,4-tetrahydronaphthalene-1,2,3,4,6,7-hexayl hexaacetate (3d^{syn})

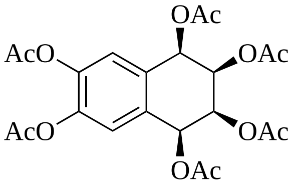

^{1}H NMR (400 MHz, Chloroform-*d*) δ 7.15 (s, 2H), 6.21-6.15 (m, 2H), 5.58-5.53 (m, 2H), 2.30 (s, 6H), 2.13 (s, 6H), 2.07 (s, 6H).

^{13}C NMR (101 MHz, Chloroform-*d*) δ 170.45, 170.11, 168.13, 142.90, 130.97, 123.54, 67.42, 67.27, 20.96, 20.86, 20.75.

HRMS(ESI+) m/z calculated $[M+Na]^+$ 503.1160, found 503.1163.

(1α,2α,3β,4β)-1,2,3,4-tetrahydronaphthalene-1,2,3,4,6,7-hexayl hexaacetate (3d^{anti})

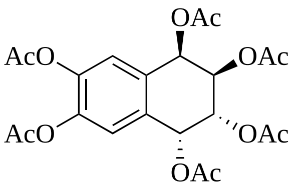

^{1}H NMR (400 MHz, Chloroform-*d*) δ 7.17 (s, 1H), 6.39-6.34 (m, 1H), 5.66 (dd, J = 2.3, 1.3 Hz, 1H), 2.29 (s, 3H), 2.09 (s, 3H), 2.06 (s, 3H).

^{13}C NMR (101 MHz, Chloroform-*d*) δ 170.41, 170.04, 167.99, 143.29, 131.51, 125.00, 68.41, 66.96, 21.05, 20.84, 20.72.

HRMS(ESI+) m/z calculated $[M+Na]^+$ 503.1160, found 503.1155.

6-nitro-1,2,3,4-tetrahydronaphthalene-1,2,3,4-tetrayl tetraacetate (3i)

Purification by flash chromatography on silica gel (SiO_2; Hexane:AcOEt, 9:1) gave the products in a 75% yield, as a beige solid for isomer *syn* and pale beige solid for isomer *anti*.

(1α,2α,3α,4α)-6-nitro-1,2,3,4-tetrahydronaphthalene-1,2,3,4-tetrayl tetraacetate (3i^{syn})

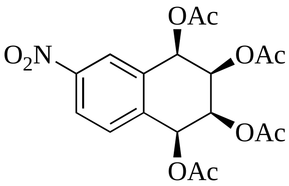

^{1}H NMR (400 MHz, Chloroform-*d*) δ 8.23 (dd, J = 8.6, 2.3 Hz, 1H), 8.20-8.17 (m, 1H), 7.52 (d, J = 8.6 Hz, 1H), 6.27 (dd, J = 6.9, 4.5 Hz, 2H), 5.62 (dd, J = 4.6, 1.9 Hz, 1H), 5.60 (dd, J = 4.6, 2.0 Hz, 1H), 2.19 (s, 3H), 2.17 (s, 3H), 2.07 (d, J = 0.6 Hz, 6H).

^{13}C NMR (101 MHz, Chloroform-*d*) δ 170.35-170.13 (m), 169.95, 169.94, 148.52, 139.02, 134.31, 129.90, 124.01, 123.66, 67.38, 67.28, 67.09, 67.05, 20.90, 20.85, 20.76.

HRMS(ESI+) m/z calculated $[M+Na]^+$ 432.0901, found 432.0898.

(1α,2α,3β,4β)-6-nitro-1,2,3,4-tetrahydronaphthalene-1,2,3,4-tetrayl tetraacetate (3i^{anti})

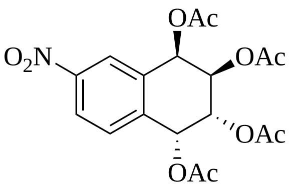

^{1}H NMR (400 MHz, Chloroform-*d*) δ 8.23 (dd, *J* = 8.5, 2.4 Hz, 1H), 8.19 (d, *J* = 2.3 Hz, 1H), 7.52 (d, *J* = 8.5 Hz, 1H), 6.44 (t, *J* = 2.1 Hz, 2H), 5.72 – 5.65 (m, 2H), 2.14 (s, 3H), 2.12 (s, 3H), 2.08 (s, 6H).

^{13}C NMR (101 MHz, Chloroform-*d*) δ 170.16, 169.94, 169.92, 148.67, 139.38, 134.71, 130.83, 124.65, 124.45, 68.01, 67.97, 67.08, 67.04, 20.95, 20.92, 20.80.

HRMS(ESI+) m/z calculated [M+Na]$^+$ 432.0901, found 432.0901.

(1α,2α,3α,4α)-5-ethyl-1,2,3,4-tetrahydronaphthalene-1,2,3,4-tetrayl tetraacetate (3n^{syn})

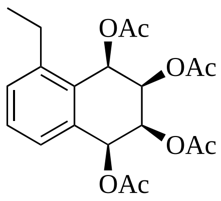

Purification by flash chromatography on silica gel (SiO_2; Hexane:AcOEt, 9:1) gave the products in a 16% yield, as a white solid.

^{1}H NMR (400 MHz, Chloroform-*d*) δ 7.39 (t, *J* = 7.7 Hz, 1H), 7.28 (d, *J* = 7.7 Hz, 1H), 7.15 (d, *J* = 7.8 Hz, 1H), 6.45 (d, *J* = 4.7 Hz, 1H), 6.12 (d, *J* = 4.1 Hz, 1H), 5.74 (ddd, *J* = 4.1, 2.1, 1.0 Hz, 1H), 5.37 (dd, *J* = 4.8, 2.1 Hz, 1H), 2.53 (dhept, *J* = 29.8, 7.5 Hz, 2H), 2.17 (s, 3H), 2.09 (s, 3H), 2.08 (s, 3H), 2.06 (s, 3H), 1.22 (t, *J* = 7.5 Hz, 3H).

^{13}C NMR (101 MHz, Chloroform-*d*) δ 170.65, 170.47, 170.33, 169.76, 144.75, 132.76, 129.85, 129.07, 128.85, 124.36, 69.29, 67.86, 67.27, 64.84, 24.80, 21.07, 20.95, 20.74, 15.15.

HRMS(ESI+) m/z calculated [M+Na]$^+$ 415.1363, found 415.1367.

(3α,4α)-4-ethyl-4-hydroxy-1,2,3,4-tetrahydronaphthalene-1,2,3-triyl triacetate (4n)

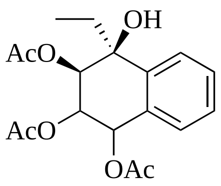

GC-MS(CI-NH_4^+) m/z [M] 350.1, [M+NH_4^+] 368.2.

Syn

^{1}H NMR (400 MHz, Chloroform-*d*) δ 7.65 (dd, *J* = 8.0, 1.4 Hz, 1H), 7.46-7.41 (m, 1H), 7.38-7.32 (m, 2H), 6.27 (d, *J* = 4.6 Hz, 1H), 5.56 (dt, *J* = 5.8, 2.0 Hz, 2H), 2.13 (s, 3H), 2.11 (s, 3H), 2.06 (s, 3H), 1.86 (h, *J* = 7.2 Hz, 2H), 1.21 (t, *J* = 7.0 Hz, 3H).

Anti

^{1}H NMR (400 MHz, Chloroform-*d*) δ 7.31-7.28 (m, 1H), 7.28-7.26 (m, 2H), 7.11 (d, *J* = 7.8 Hz, 1H), 6.10 (d, *J* = 3.9 Hz, 1H), 5.88-5.85 (m, 1H), 5.18 (dd, *J* = 4.8, 1.9 Hz, 1H), 2.88 (dp, *J* = 22.1, 7.4 Hz, 2H), 2.20 (s, 3H), 2.15 (s, 3H), 2.09 (s, 3H), 1.21 (t, *J* = 7.0 Hz, 3H).

GC and GC-MS analysis of the reaction crude after acetylation w-up showing minor amounts of **4n**. The product decomposes during purification and can only be isolated in minor (<5%) amounts.

GC-MS trace of the reaction mixture after acetylation w-up and assignment according to the MS spectrum.

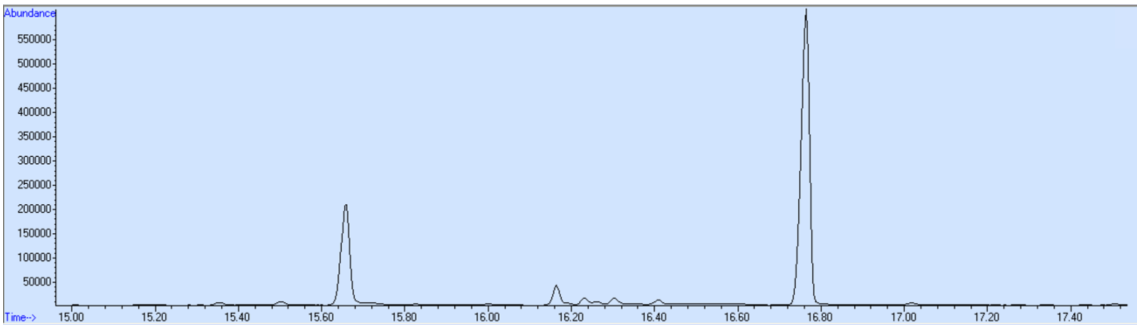

Mass of the signal after 15.6 minutes

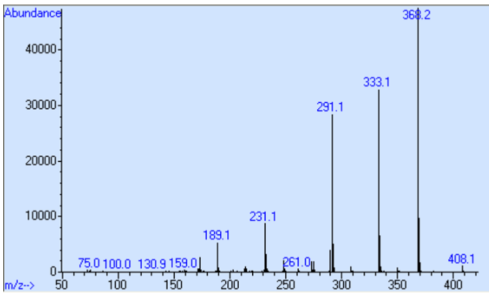

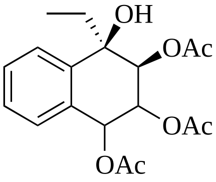

NH_4^+

m.w: 368.17

Both diastereoisomers appear at the same retention time in the GC (but are identifiable by ^{1}H-NMR).

Mass of the signal before 16.2 minutes

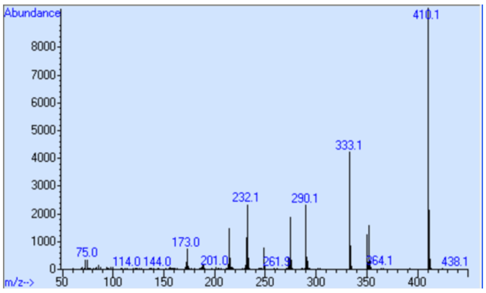

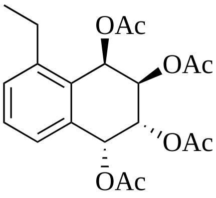

NH_4^+

m.w: 410.18

Mass of the signal before 16.8 minutes

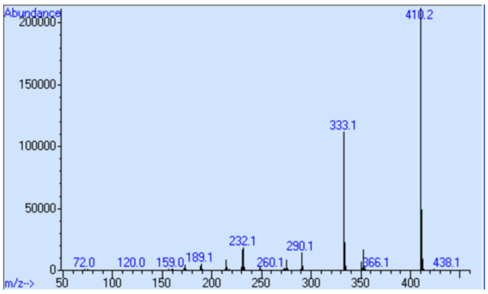

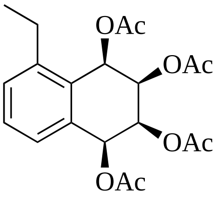

NH_4^+

m.w: 410.18

5-bromo-1,2,3,4-tetrahydronaphthalene-1,2,3,4-tetrayl tetraacetate (3o)

Purification by flash chromatography on silica gel (SiO_2; Hexane:AcOEt, 9:1) gave the products in a 37% yield, as a pale orange solid for isomer *syn* and pale green solid for isomer *anti*.

(1α,2α,3α,4α)-5-bromo-1,2,3,4-tetrahydronaphthalene-1,2,3,4-tetrayl tetraacetate ($3o^{syn}$)

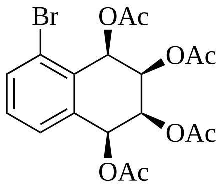

^{1}H NMR (400 MHz, Chloroform-*d*) δ 7.64-7.60 (m, 1H), 7.32-7.27 (m, 2H), 6.36 (d, J = 5.0 Hz, 1H), 6.09 (d, J = 3.8 Hz, 1H), 5.72 (ddd, J = 3.9, 2.1, 1.0 Hz, 1H), 5.39 (dd, J = 4.9, 2.0 Hz, 1H), 2.17 (s, 3H), 2.09 (s, 3H), 2.07 (s, 3H), 2.06 (s, 3H).

^{13}C NMR (101 MHz, Chloroform-*d*) δ 170.53, 170.29, 169.97, 169.71, 135.35, 133.62, 131.02, 130.87, 126.09, 125.83, 69.07, 67.82, 66.87, 66.67, 20.98, 20.86, 20.73, 20.71.

HRMS(ESI+) m/z calculated $[M+Na]^{+}$ 465.0156, found 465.0155.

(1α,2α,3β,4β)-5-bromo-1,2,3,4-tetrahydronaphthalene-1,2,3,4-tetrayl tetraacetate ($3o^{anti}$)

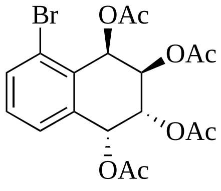

^{1}H NMR (400 MHz, Chloroform-*d*) δ 7.68 (dd, J = 6.6, 2.7 Hz, 1H), 7.31 (q, J = 4.8, 4.2 Hz, 2H), 6.58 (d, J = 3.7 Hz, 1H), 6.43 (d, J = 3.6 Hz, 1H), 5.71 (dd, J = 11.6, 3.7 Hz, 1H), 5.65 (dd, J = 11.6, 3.6 Hz, 1H), 2.11 (s, 3H), 2.10 (s, 3H), 2.08 (s, 3H), 2.08 (s, 3H).

^{13}C NMR (101 MHz, Chloroform-*d*) δ 170.32, 170.30, 170.24, 169.87, 135.51, 134.64, 132.35, 131.45, 130.00, 125.90, 69.21, 69.02, 66.94, 66.65, 21.04, 20.84, 20.83, 20.76.

HRMS(ESI+) m/z calculated $[M+Na]^{+}$ 465.0156, found 465.0155.

(3α,4α)-4-bromo-4-hydroxy-1,2,3,4-tetrahydronaphthalene-1,2,3-triyl triacetate (4o)

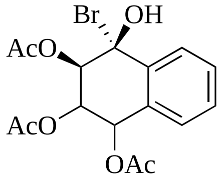

^{1}H NMR (400 MHz, Chloroform-*d*) δ 7.68-7.64 (m, 1H), 7.31-7.27 (m, 2H), 7.24 (d, *J* = 2.6 Hz, 1H), 6.09 (d, *J* = 4.1 Hz, 1H), 5.86 (dt, *J* = 3.8, 1.7 Hz, 1H), 5.20 (d, *J* = 1.8 Hz, 1H), 2.20 (s, 3H), 2.16 (s, 3H), 2.10 (s, 3H).

^{13}C NMR (101 MHz, Chloroform-*d*) δ 170.29, 169.99, 169.67, 134.78, 133.95, 133.22, 130.43, 126.63, 125.89, 70.13, 69.29, 68.00, 66.01, 21.11, 21.01, 20.86.

GC and GC-MS analysis of the reaction crude after acetylation w-up showing minor amounts of **4o**. The product decomposes during purification and can only be isolated in minor (<5%) amounts.

GC-MS trace of the reaction mixture after acetylation w-up and assignment according to the MS spectrum.

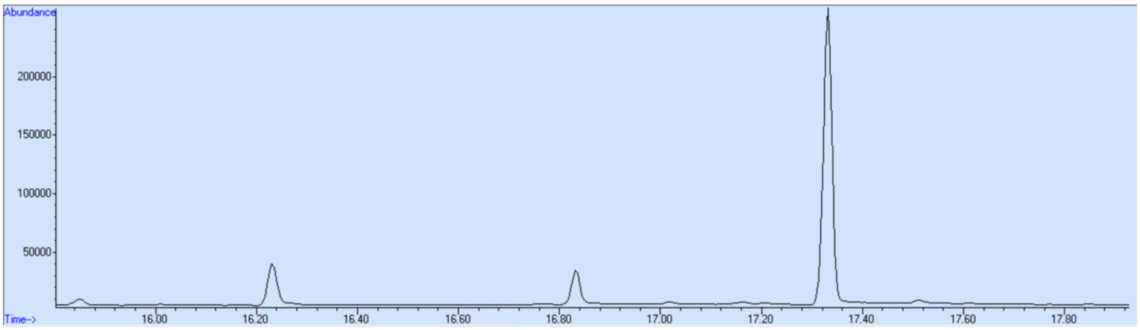

Mass of the signal after 16.2 minutes

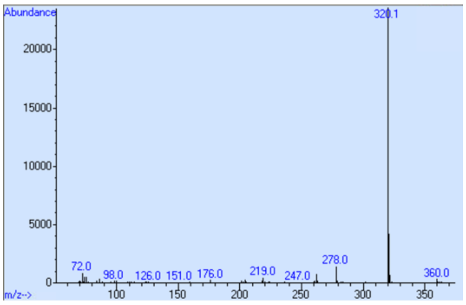

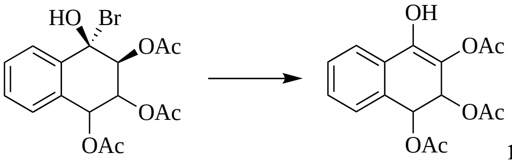

m.w: 320.09

Mass of the signal after 16.8 minutes

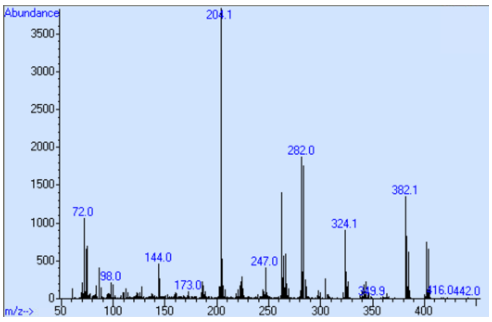

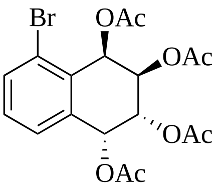

m.w: 442.03

Mass of the signal after 17.3 minutes

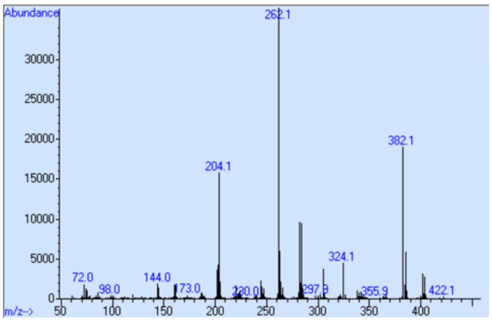

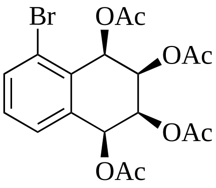

NH_4^+

m.w: 460.06

m/z 382.1:

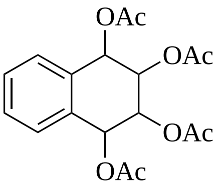

NH_4^+

5-chloro-1,2,3,4-tetrahydronaphthalene-1,2,3,4-tetrayl tetraacetate (3p)

Purification by flash chromatography on silica gel (SiO_2; Hexane:AcOEt, 9:1) gave the products in a 25% yield, as a white solid for isomer *syn* and pale orange solid for isomer *anti*.

(1α,2α,3α,4α)-5-chloro-1,2,3,4-tetrahydronaphthalene-1,2,3,4-tetrayl tetraacetate ($3p^{syn}$)

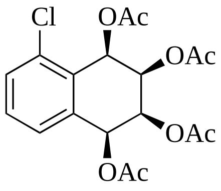

^{1}H NMR (400 MHz, Chloroform-*d*) δ 7.43 (dt, *J* = 7.9, 1.2 Hz, 1H), 7.37 (t, *J* = 7.8 Hz, 1H), 7.23 (dt, *J* = 7.8, 1.3 Hz, 1H), 6.45 (d, *J* = 5.0 Hz, 1H), 6.09 (d, *J* = 3.9 Hz, 1H), 5.73 (ddd, *J* = 3.9, 2.1, 1.0 Hz, 1H), 5.39 (dd, *J* = 5.0, 2.0 Hz, 1H), 2.18 (s, 3H), 2.09 (s, 3H), 2.08 (s, 3H), 2.06 (s, 3H).

^{13}C NMR (101 MHz, Chloroform-*d*) δ 170.55, 170.30, 169.97, 169.71, 135.95, 135.06, 130.61, 130.13, 129.60, 125.15, 69.07, 67.77, 66.64, 64.81, 20.99, 20.89, 20.72, 20.67.

HRMS(ESI+) m/z calculated $[M+Na]^{+}$ 421.0661, found 421.0664.

(1α,2α,3β,4β)-5-chloro-1,2,3,4-tetrahydronaphthalene-1,2,3,4-tetrayl tetraacetate ($3p^{anti}$)

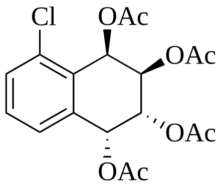

^{1}H NMR (400 MHz, Chloroform-*d*) δ 7.46 (dd, *J* = 8.0, 1.3 Hz, 1H), 7.37 (t, *J* = 7.8 Hz, 1H), 7.27-7.24 (m, 1H), 6.63 (d, *J* = 3.8 Hz, 1H), 6.42 (d, *J* = 3.6 Hz, 1H), 5.69 (dd, *J* = 11.7, 3.8 Hz, 1H), 5.63 (dd, *J* = 11.6, 3.6 Hz, 1H), 2.09 (s, 3H), 2.08 (s, 3H), 2.06 (s, 3H), 2.06 (s, 3H).

^{13}C NMR (101 MHz, Chloroform-*d*) δ 170.32, 170.30, 170.25, 169.90, 135.71, 135.29, 131.27, 131.18, 130.81, 129.28, 69.17, 66.78, 66.76, 66.66, 21.05, 20.84, 20.72.

HRMS(ESI+) m/z calculated $[M+Na]^+$ 421.0661, found 421.0669.

(3α,4α)-4-chloro-4-hydroxy-1,2,3,4-tetrahydronaphthalene-1,2,3-triyl triacetate (4p)

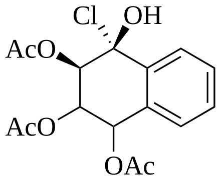

Syn

^1H NMR (400 MHz, Chloroform-*d*) δ 7.49-7.44 (m, 1H), 7.34 (t, *J* = 7.9 Hz, 1H), 7.20 (d, *J* = 7.9 Hz, 1H), 6.08 (d, *J* = 3.9 Hz, 1H), 5.86 (dt, *J* = 3.8, 1.6 Hz, 1H), 5.19 (dd, *J* = 4.9, 1.9 Hz, 1H), 2.20 (s, 3H), 2.16 (s, 3H), 2.10 (s, 3H).

Anti

^1H NMR (400 MHz, Chloroform-*d*) δ 7.46-7.42 (m, 1H), 7.37 (t, *J* = 7.8 Hz, 1H), 7.22 (d, *J* = 10.2 Hz, 2H), 6.41 (d, *J* = 5.0 Hz, 1H), 6.04 (d, *J* = 3.7 Hz, 1H), 5.65 (d, *J* = 2.6 Hz, 1H), 2.20 (d, *J* = 0.7 Hz, 3H), 2.13 (s, 3H), 2.07 (s, 3H).

GC and GC-MS analysis of the reaction crude after acetylation w-up showing minor amounts of **4p**. The product decomposes during purification and can only be isolated in minor (<5%) amounts.

GC-MS trace of the reaction mixture after acetylation w-up and assignment according to the MS spectrum.

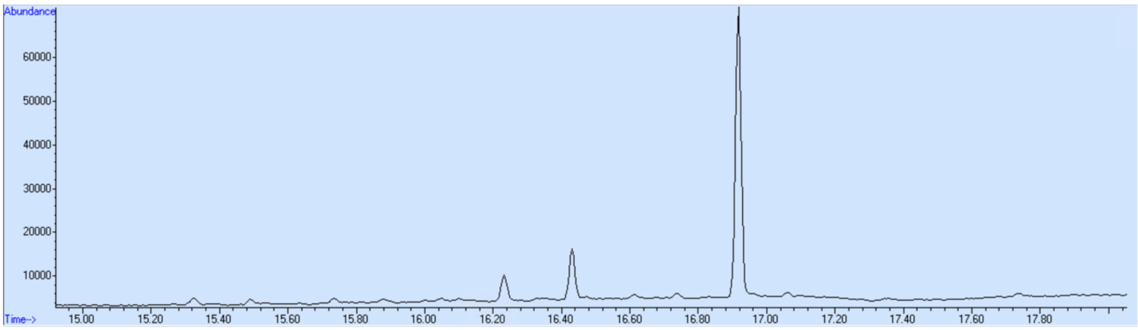

Mass of the signal after 16.2 minutes

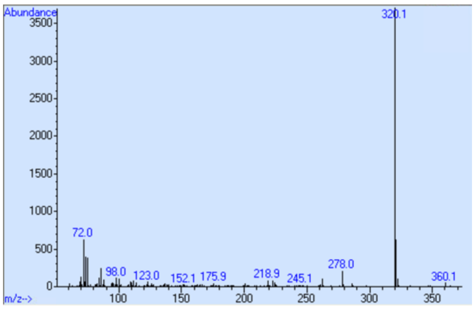

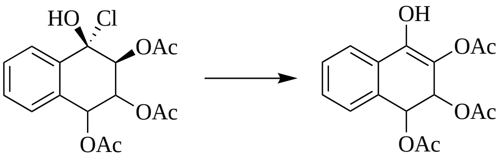

m.w: 320.09

Mass of the signal after 16.4 minutes

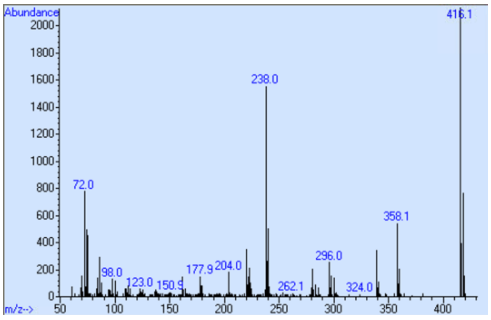

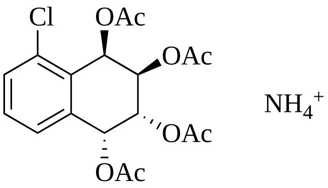

m.w: 416.11

Mass of the signal after 16.9 minutes

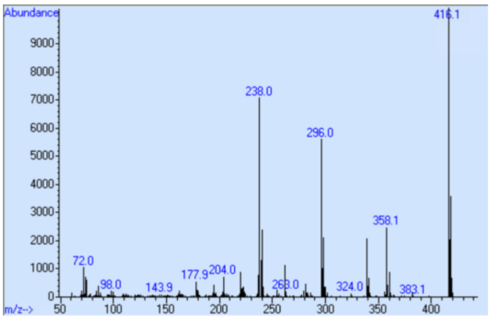

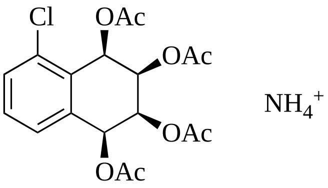

m.w: 416.11

6-acetyl-1,2,3,4-tetrahydronaphthalene-1,2,3,4-tetrayl tetraacetate (3k)

Purification by flash chromatography on silica gel (SiO_2; Hexane:AcOEt, 8.5:1.5) gave the products in a 37% yield, as a pale yellow solid for isomer *syn* and pale orange solid for isomer *anti*.

(1α,2α,3α,4α)-6-acetyl-1,2,3,4-tetrahydronaphthalene-1,2,3,4-tetrayl tetraacetate (3k^{syn})

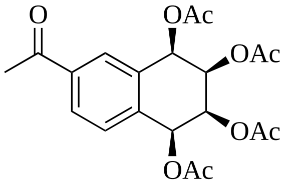

^{1}H NMR (400 MHz, Chloroform-*d*) δ 7.96 (dd, *J* = 8.1, 1.8 Hz, 1H), 7.90 (d, *J* = 1.8 Hz, 1H), 7.43 (d, *J* = 8.2 Hz, 1H), 6.28 (d, *J* = 4.4 Hz, 1H), 6.26 (d, *J* = 4.3 Hz, 1H), 5.65 – 5.56 (m, 2H), 2.61 (s, 3H), 2.17 (s, 3H), 2.16 (s, 3H), 2.07 (s, 6H).

^{13}C NMR (101 MHz, Chloroform-*d*) δ 197.01, 170.33, 170.24, 169.95, 169.88, 137.72, 136.90, 132.62, 128.78, 128.62, 128.49, 67.63, 67.54, 67.29, 67.22, 26.69, 20.86, 20.77, 20.68, 20.66.

HRMS(ESI+) m/z calculated $[M+Na]^+$ 429.1156, found 429.1150.

(1α,2α,3β,4β)-6-acetyl-1,2,3,4-tetrahydronaphthalene-1,2,3,4-tetrayl tetraacetate (3k^{anti})

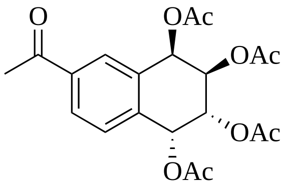

^{1}H NMR (400 MHz, Chloroform-*d*) δ 7.96 (dd, *J* = 8.1, 1.8 Hz, 1H), 7.88 (d, *J* = 1.8 Hz, 1H), 7.42 (d, *J* = 8.1 Hz, 1H), 6.45 (t, *J* = 3.1 Hz, 2H), 5.70 – 5.67 (m, 2H), 2.60 (s, 3H), 2.10 (s, 3H), 2.09 (s, 3H), 2.07 (d, *J* = 0.8 Hz, 6H).

^{13}C NMR (101 MHz, Chloroform-*d*) δ 197.01, 170.35, 170.27, 170.10, 138.39, 137.47, 133.33, 130.43, 130.06, 129.52, 68.87, 68.61, 67.18, 67.05, 26.85, 21.05, 20.98, 20.83.

HRMS(ESI+) m/z calculated $[M+Na]^+$ 429.1156, found 429.1154.

(3α,4α)-3-acetyl-3-hydroxy-1,2,3,4-tetrahydronaphthalene-1,2,4-triyl triacetate (4k)

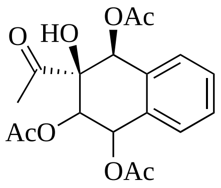

^{1}H NMR (400 MHz, Chloroform-*d*) δ 7.40 (dtd, *J* = 14.8, 6.7, 6.2, 1.2 Hz, 2H), 7.33 (dd, *J* = 7.6, 1.8 Hz, 1H), 7.21 – 7.14 (m, 1H), 6.44 (d, *J* = 4.8 Hz, 1H), 6.35 (s, 1H), 5.69 (d, *J* = 4.9 Hz, 1H), 2.35 (s, 3H), 2.21 (s, 3H), 2.14 (s, 3H), 2.05 (s, 3H).

^{13}C NMR (101 MHz, Chloroform-*d*) δ 207.30, 170.61, 170.57, 169.60, 132.27, 131.07, 130.44, 130.09, 129.21, 127.00, 81.16, 71.92, 69.38, 67.57, 25.39, 21.16, 20.76, 20.56.

HRMS(ESI+) m/z calculated $[M+Na]^+$ 387.1050, found 387.1042.

10. ^{18}O labelling experiments

In a 3mL vial equipped with a stir bar a solution of substrate (0.45 µmol) in 400 µL of acetonitrile was added, afterwards the catalyst (0.5 mg, 0.0045 µmol, 1 mol%) was added. The mixture was cooled in an ice bath and 2 eq. of H_2O_2 were added via syringe pump for 30 minutes. After the catalysis acetylation was performed as described before.

H_2O_2 preparation: in a small vial 9mg of H_2O_2 70% in H_2O were weighted, 300 µL of $H_2{}^{18}O$ 97% (100 eq. respect to the $H_2{}^{16}O$ of the solution of H_2O_2) and 50 µL of acetonitrile were added.

GC-MS analysis of the solution provided m/z peaks of the products.

Non labelled diol **2a** analysis:

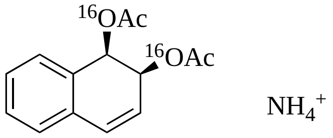

Molecular weight: 264.12

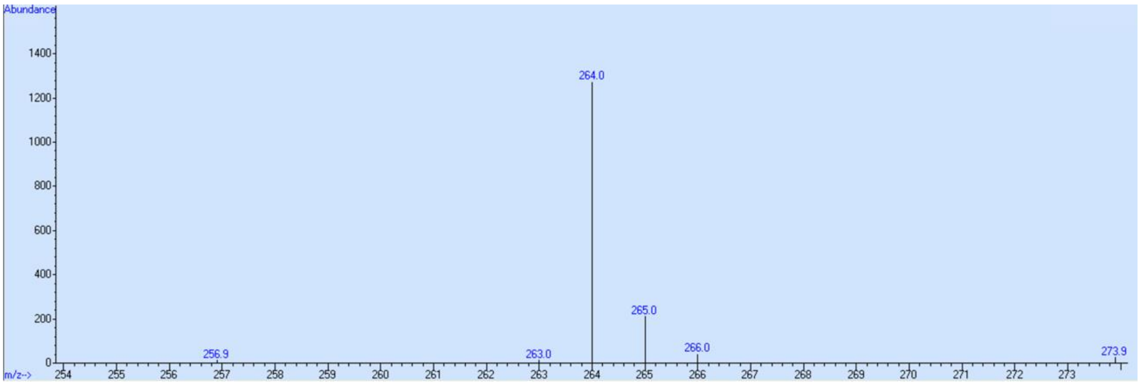

Labelled diol **2a** analysis (entry 1, Table S25):

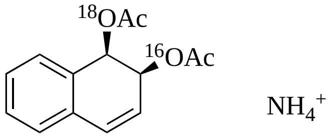

Molecular weight: 266.12

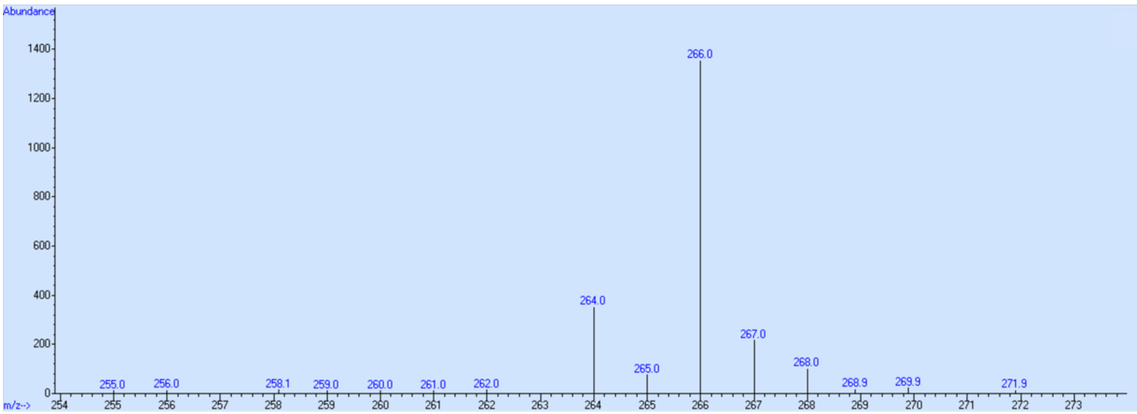

Non labelled *syn* tetraol **3a**$^{\mathbf{syn}}$ analysis:

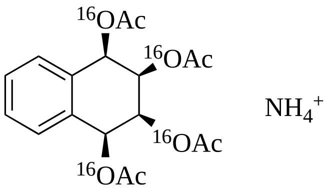

Molecular weight: 382.15

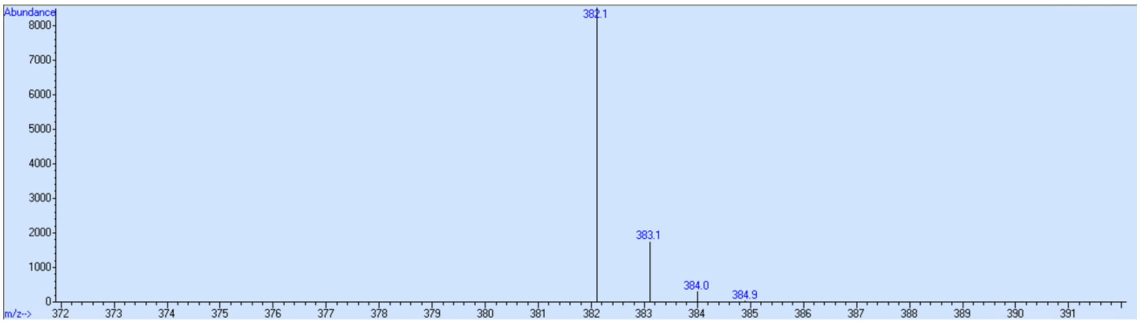

Labelled *syn* tetraol **3a**$^{\mathbf{syn}}$ analysis, (entry 3, Table S25):

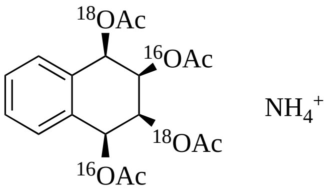

Molecular weight: 386.15

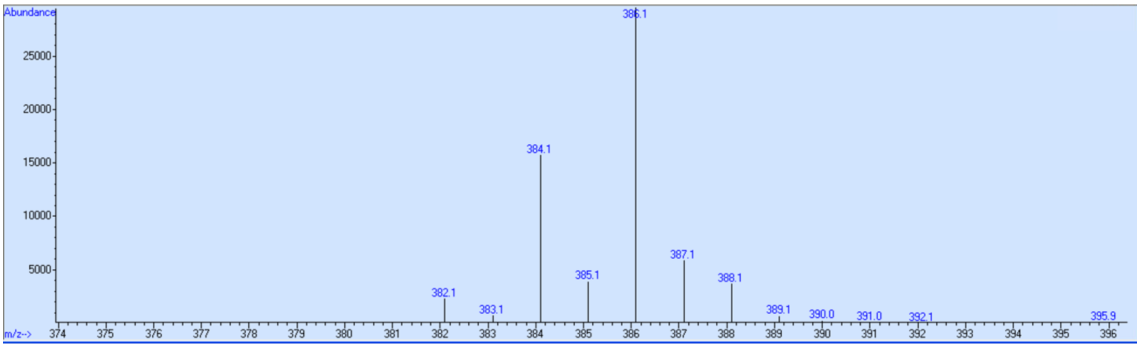

Non labelled *anti* tetraol **3a**$^{\mathbf{anti}}$ analysis:

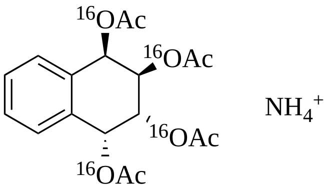

Molecular weight: 382.15

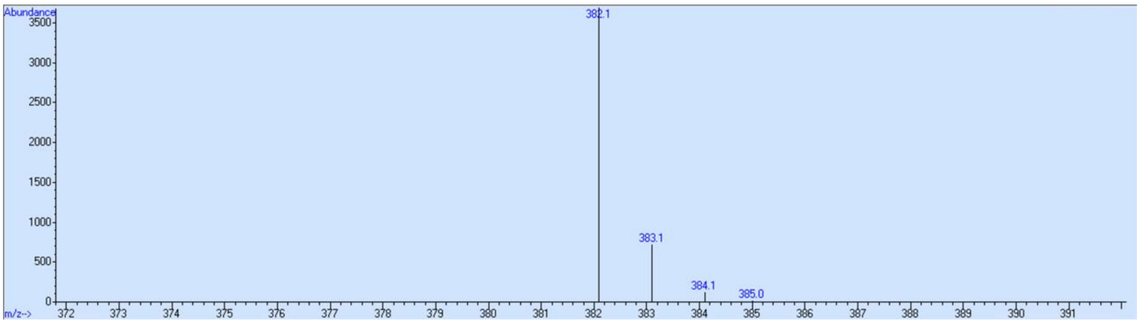

Labelled *anti* tetraol **3a**$^{\mathbf{anti}}$ analysis, (entry 4, Table S25):

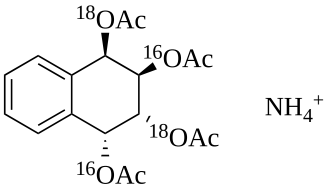

Molecular weight: 386.15

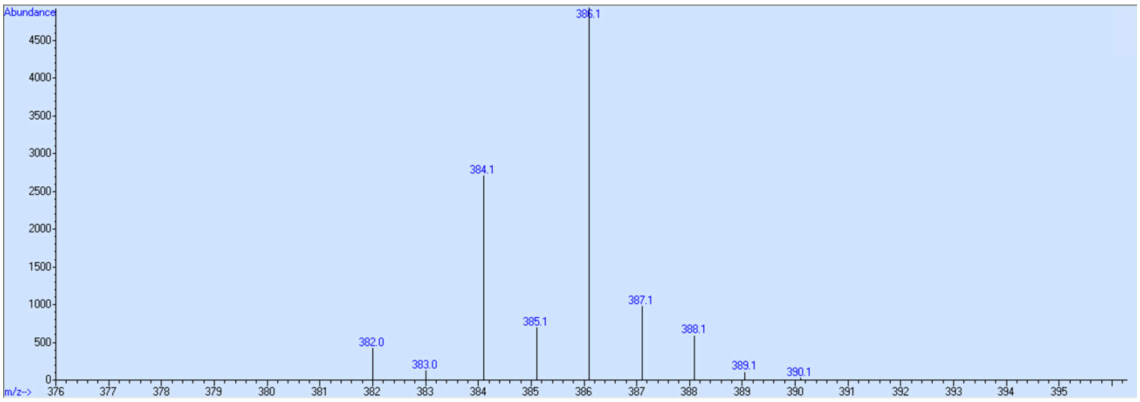

Table S25. Isotopic labelling data for the different products. Empty O corresponds to ^{16}O, black filled O corresponds to ^{18}O. The specific position of ^{16}O and ^{18}O in ^{16}O^{18}O-labelled products can not be determined.

Entry[a]	(%)	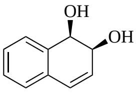	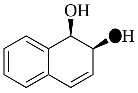	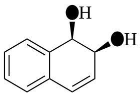	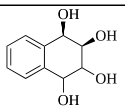	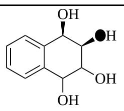	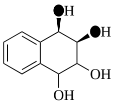	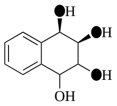
1	Exp. **2a**	19.9	75.2	2.9				
2[b]	Theor. **3a**				4.0	29.9	57.7	4.4
3	Exp. **3a**$^{\mathbf{syn}}$				4.9	31.6	56.6	5.0
4	Exp **3a**$^{\mathbf{anti}}$				4.4	30.4	57.0	5.4

[a] Experimental conditions as in ^{18}O labelling experiment (section 8).

[b] Theoretical values of **3a** were calculated based on the experimentally determined isotopic distribution of **2a** in the following way: 4.0% (19.9*0.199), 29.9% (19.9*0.752+75.2*0.199), 57.7% (19.9*0.029+2.9*0.199+75.2*0.752), 4.4% (75.2*0.029+2.9*0.752).

11. GC chromatograms

- **Diol analysis for non-symmetric substrates:**

Table S26. Diols obtained through the reaction of non-symmetric substrates. Separation and specific identification of the three diols could not be made.

Entry[a]	Substrate	Conversion	Diol 1	Diol 2	Diol 3
1	1-Et (**1n**)	48	7.6	6.9	3.8
2	2-Et (**1g**)	39	11.2	7.4	11.2
3	1-CN (**1q**)	35	2.0	3.7	0.1
4	2-CN (**1j**)	39	5.2	5.4	7.6
5	1-AcO	38	5.2	4.5	0.0
6	2-AcO (**1f**)	49	8.5	9.4	0.0
7	1-Cl (**1p**)	-	2.2	1.4	3.6
8	2-Cl (**1m**)	40	5.8	10.2	9.9

[a] Reaction conditions as described in diol formation 5.1.1. (3 mol% catalyst, 1 eq. H_2O_2, 2.2 eq. $Mg(SO_4)$, 1 eq. substrate), followed by standard acetylation w-up and analysis by GC and GC-MS. Product yields estimated by GC, by integration against an internal standard. Response factors corresponding to **3x** were used for estimating the amounts of diols.

Oxidation of **1o**:

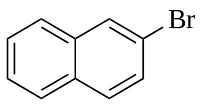

Products detected in the GC-MS (proposed structures according to the ms):

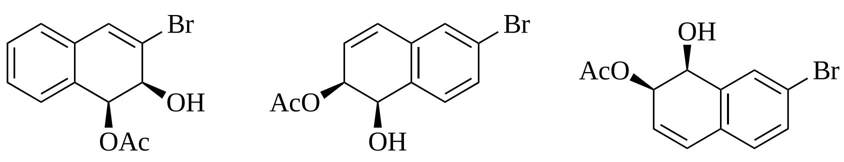

m.w: 281.99

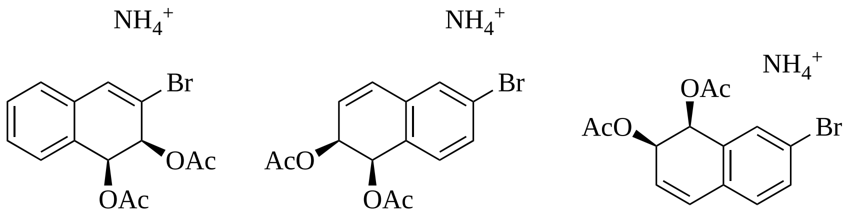

m.w: 342.03

GC-MS trace of the reaction mixture after acetylation w-up.

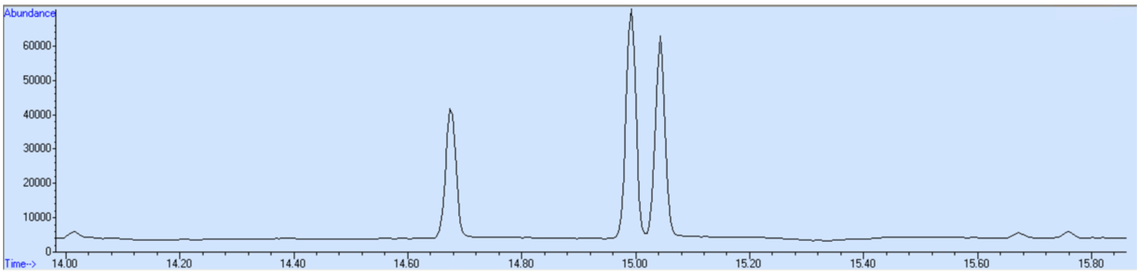

Mass of the signal after 14.6 minutes

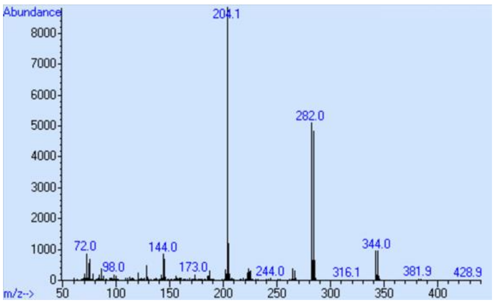

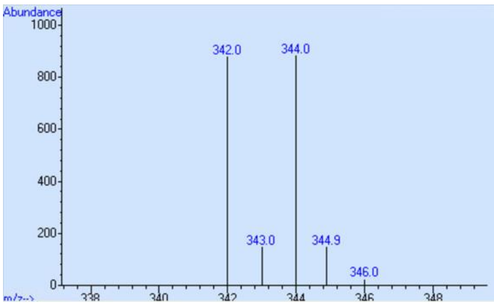

Mass of the signal before 15.0 minutes

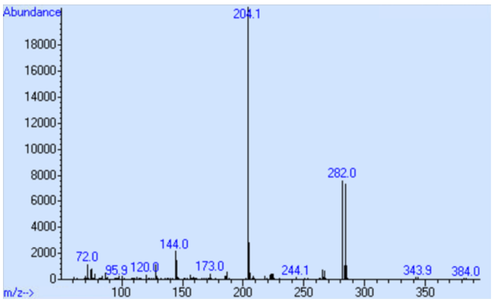

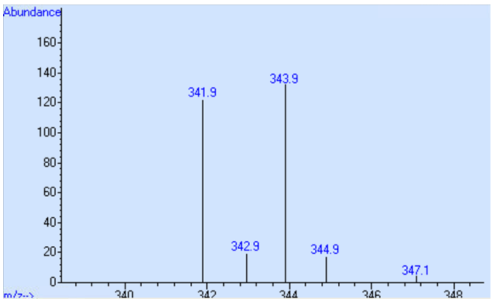

Mass of the signal after 15.0 minutes

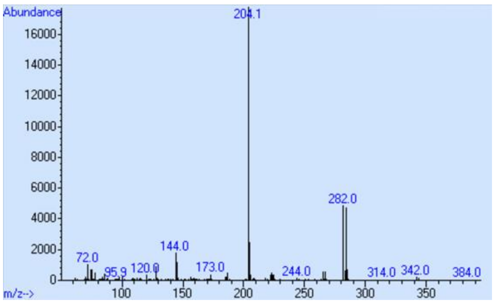

Oxidation of **1m**:

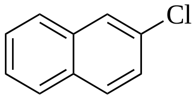

Products detected in the GC-MS (proposed structures according to the ms):

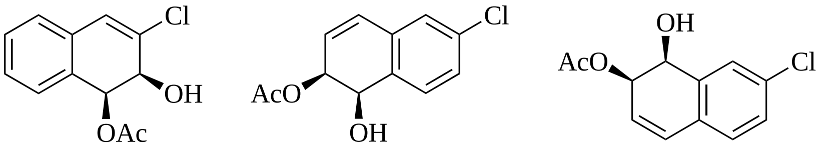

m.w: 238.04

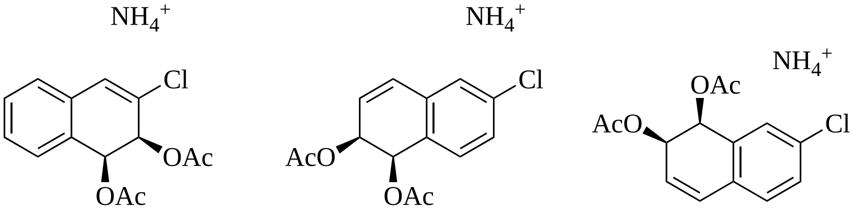

m.w: 298.08

GC-MS trace of the reaction mixture after acetylation w-up.

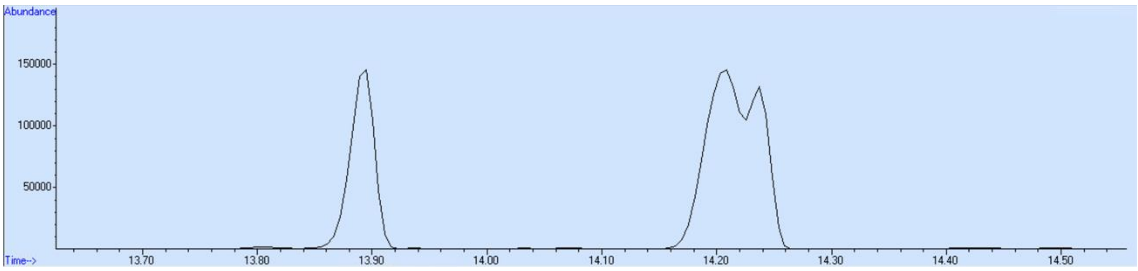

Mass of the signal before 13.9 minutes

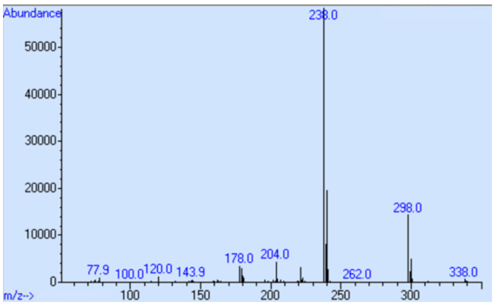

Mass of the signal at 14.2 minutes

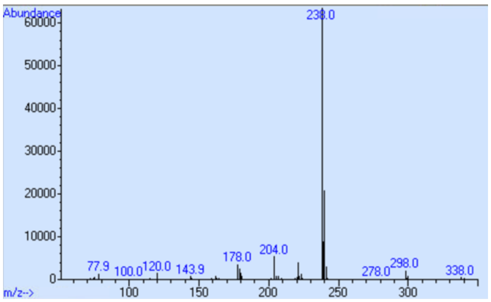

Mass of the signal after 14.2 minutes

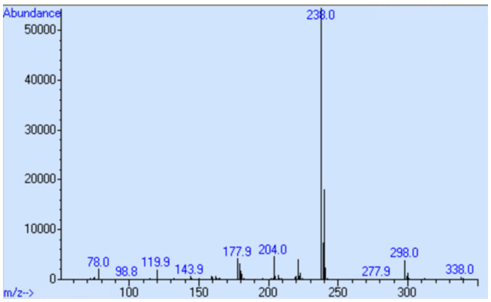

Oxidation of **1p**:

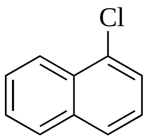

Products detected in the GC-MS (proposed structures according to the ms):

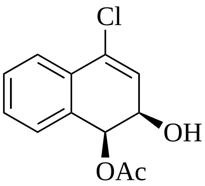

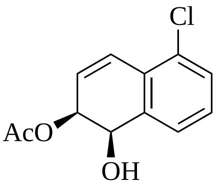

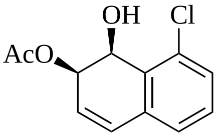

m.w: 238.04

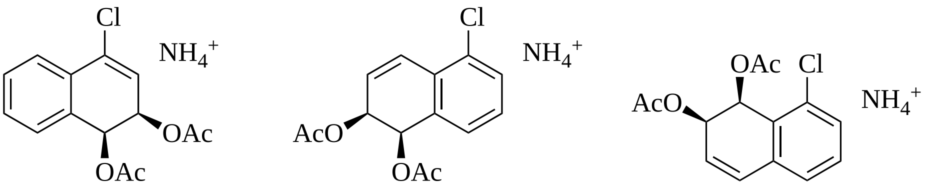

m.w: 298.08

GC-MS trace of the reaction mixture after acetylation w-up.

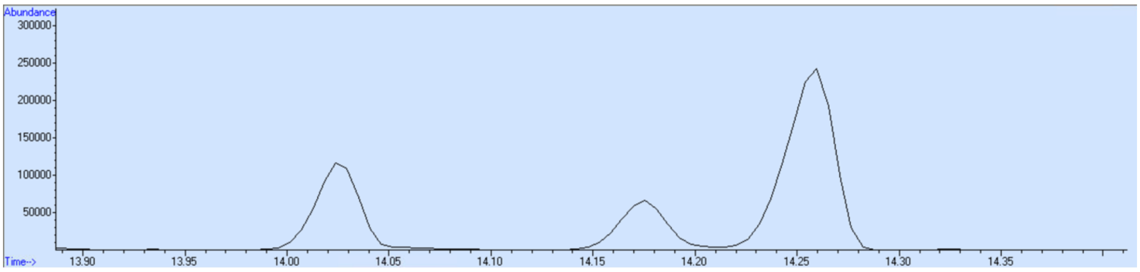

Mass of the signal after 14.0 minutes

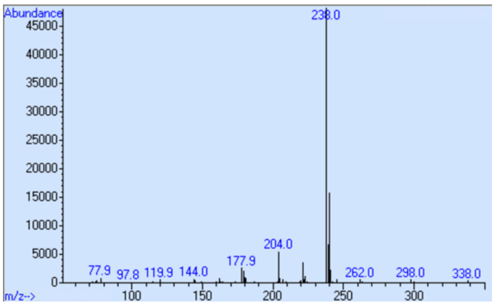

Mass of the signal after 14.15 minutes

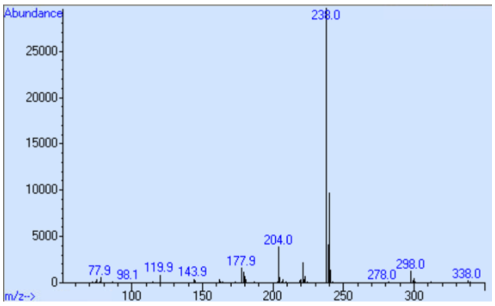

Mass of the signal at 14.25 minutes

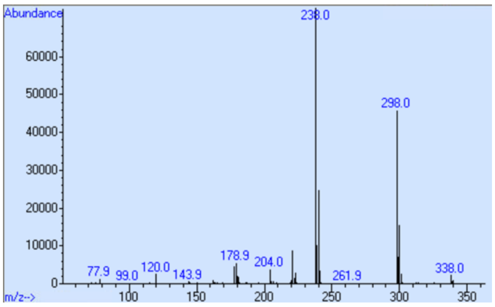

Oxidation of **1g**

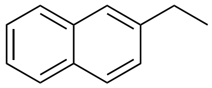

Products detected in the GC-MS (proposed structures according to the ms):

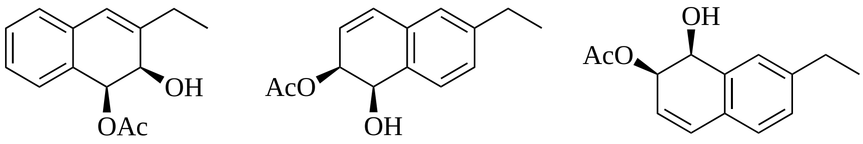

m.w: 232.11

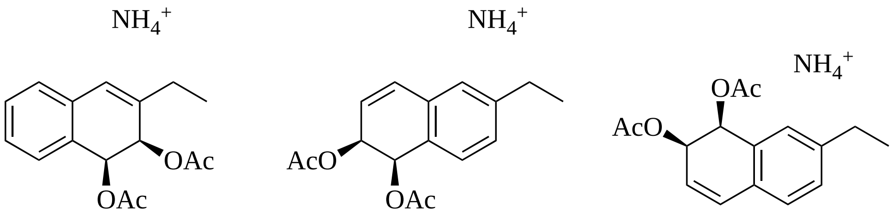

m.w: 292.15

GC-MS trace of the reaction mixture after acetylation w-up.

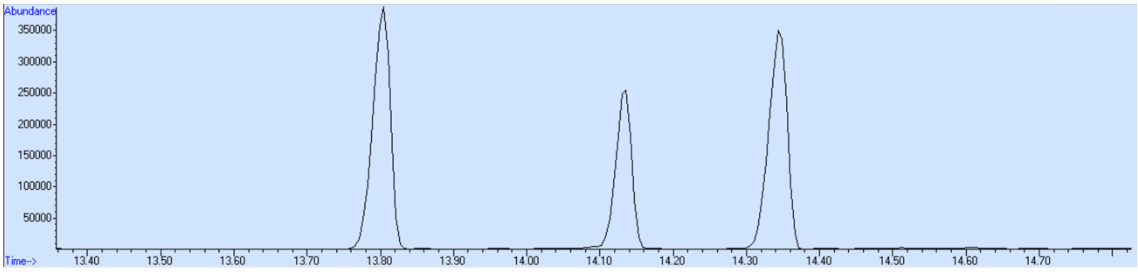

Mass of the signal at 13.8 minutes

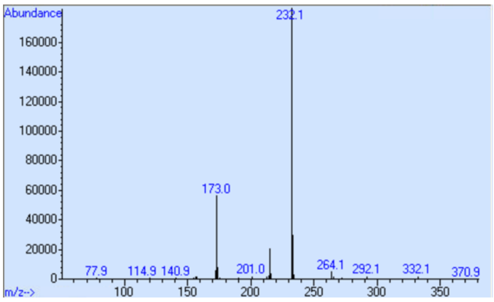

Mass of the signal after 14.1 minutes

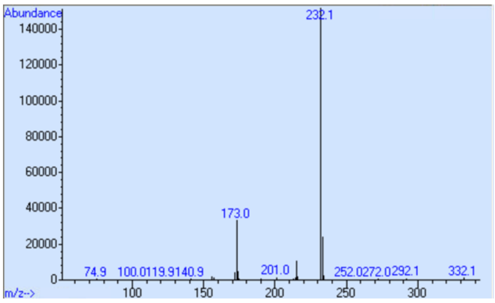

Mass of the signal after 14.3 minutes

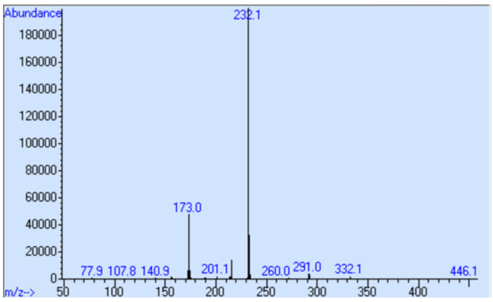

12. NMR spectra of the isolated compounds

(1α,2α)-1,2-dihydronaphthalene-1,2-diyl diacetate (2a)

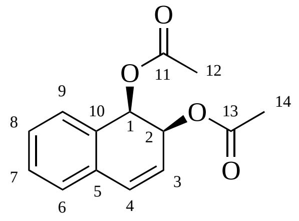

2a - ^{1}H NMR (400 MHz, $CDCl_3$)

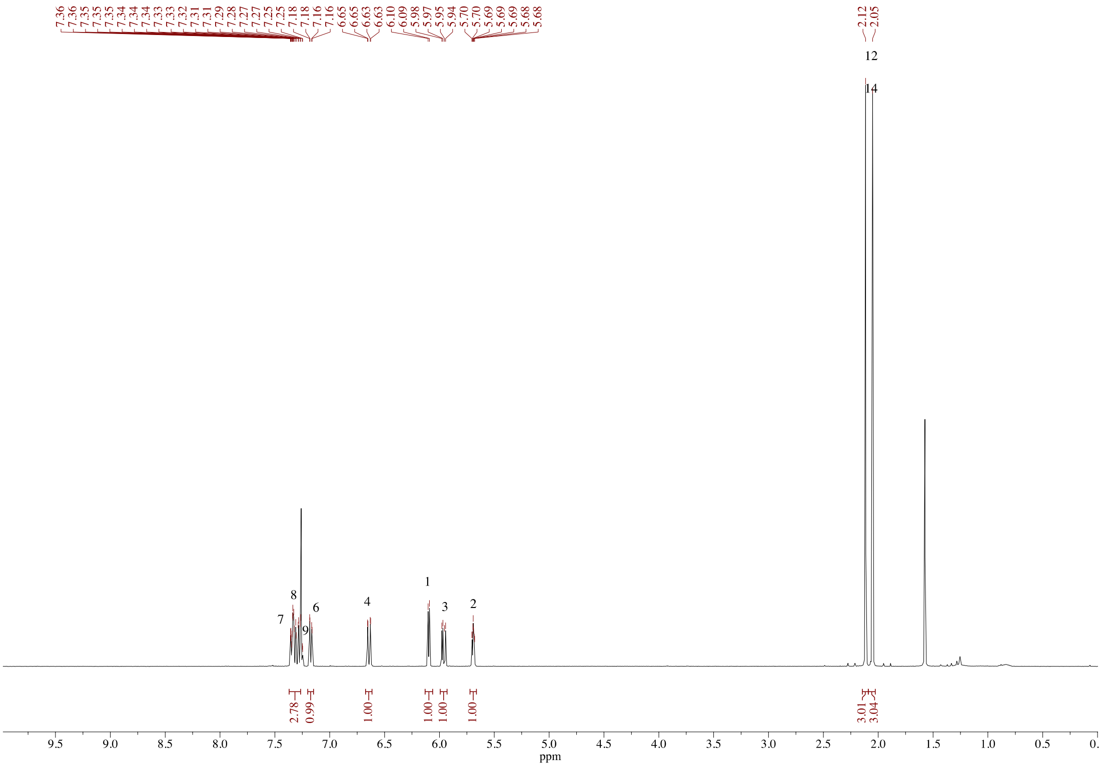

2a - ^{13}C NMR (100 MHz, $CDCl_3$)

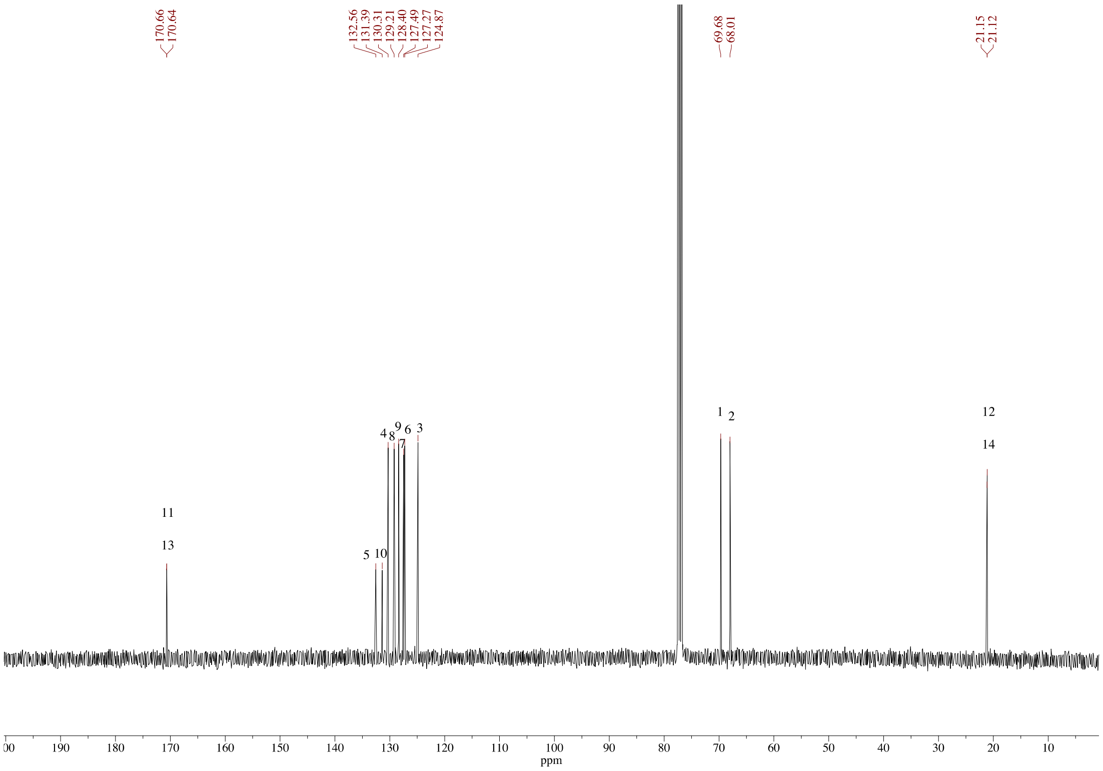

2a - DEPT ($CDCl_3$)

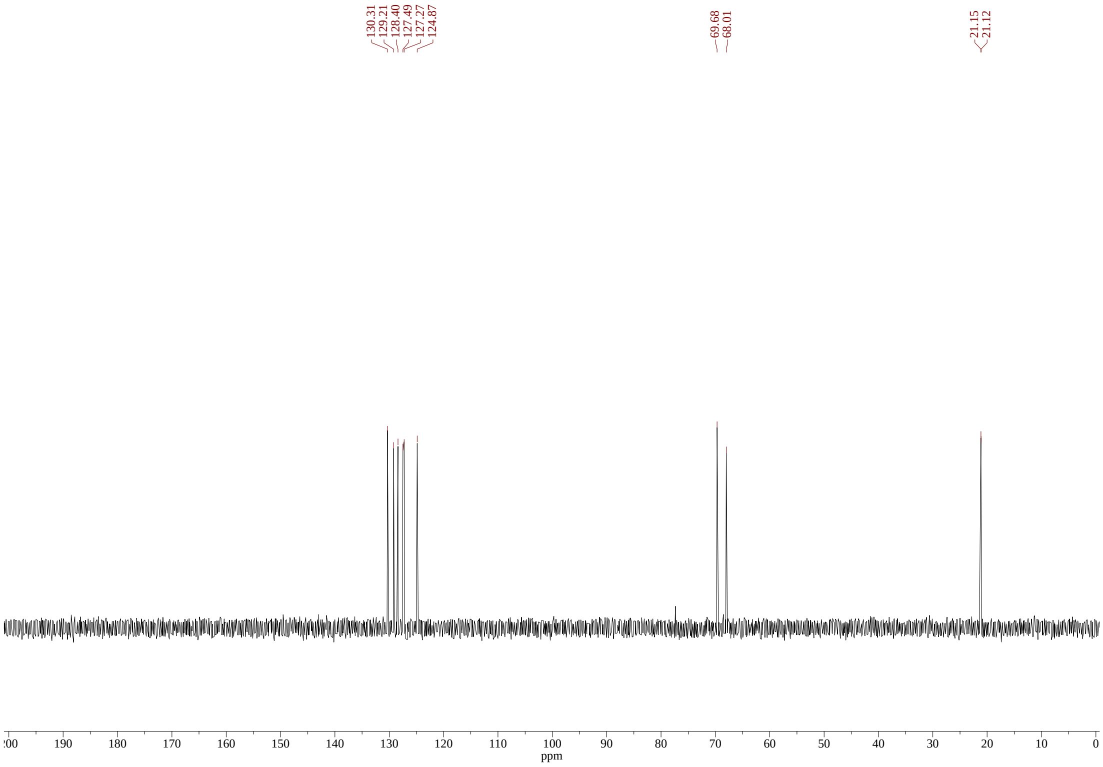

2a - DEPTQ ($CDCl_3$)

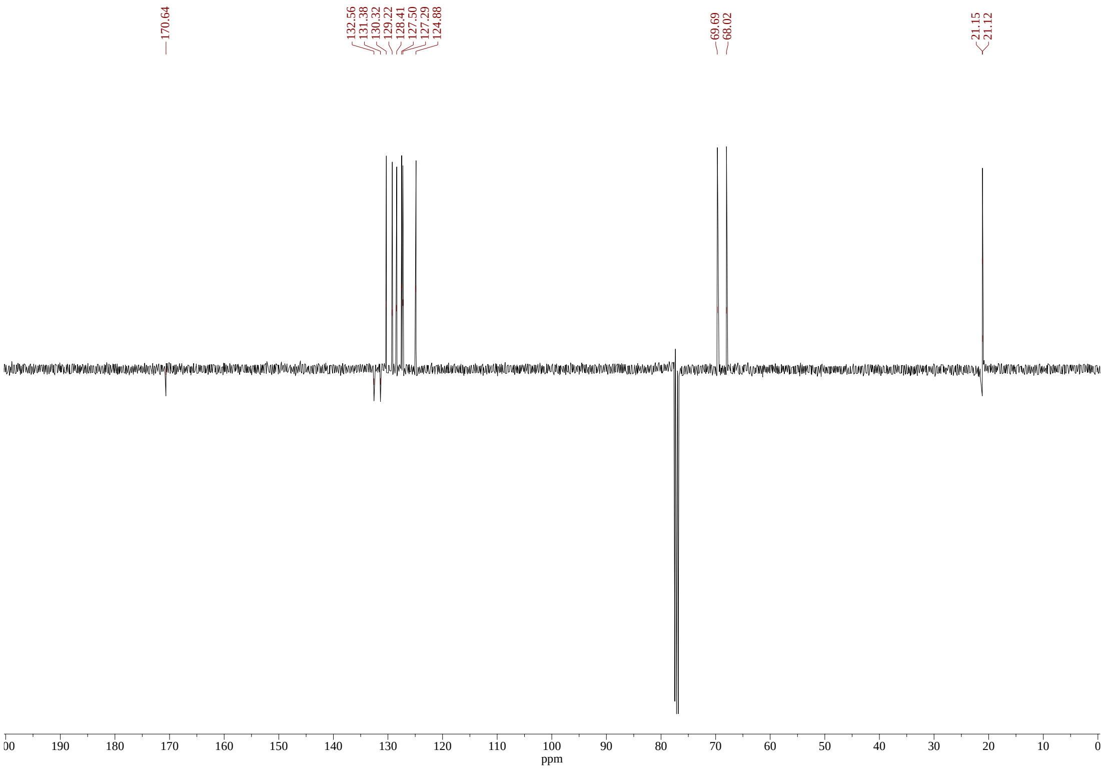

2a - ^1H-^1H COSY ($CDCl_3$)

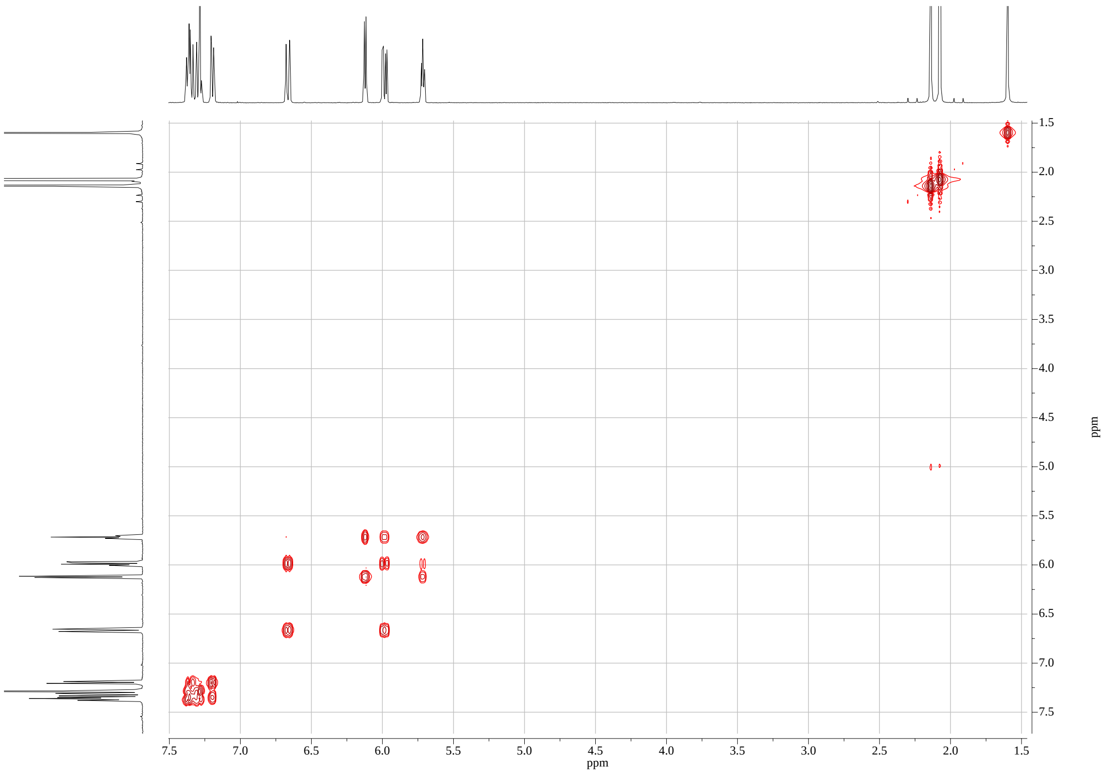

2a - ^{1}H-^{13}C HSQCED ($CDCl_3$)

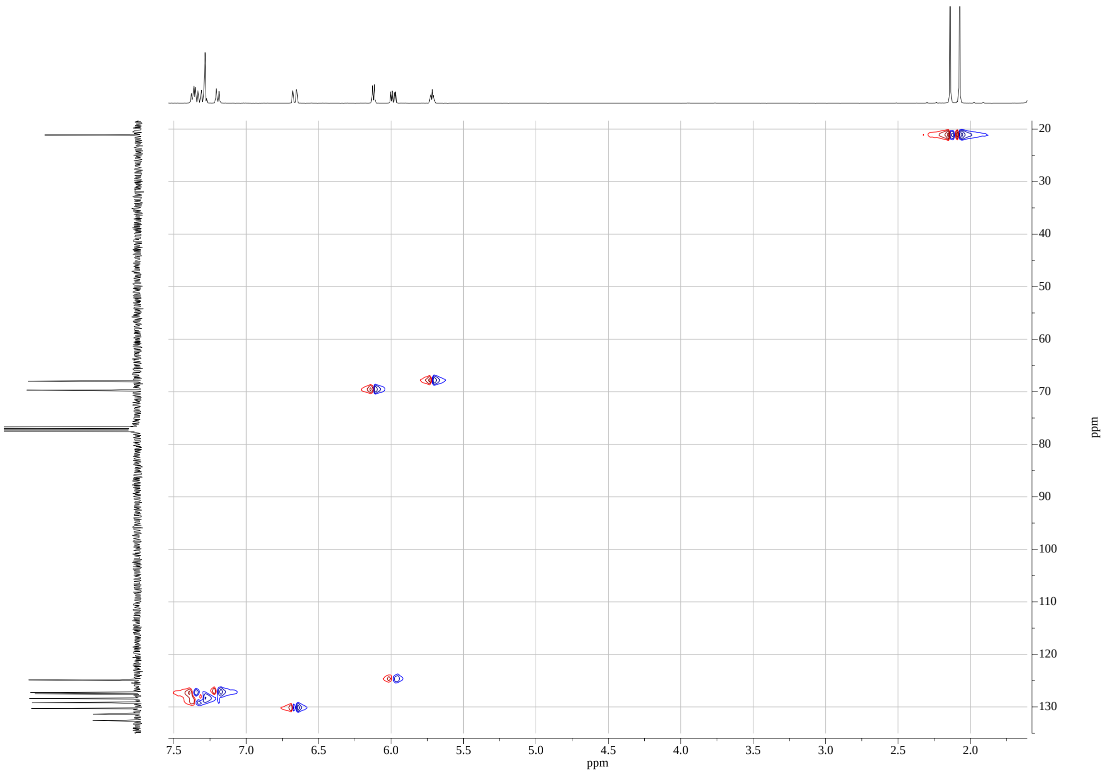

2a - ^{1}H-^{13}C HMBC ($CDCl_3$)

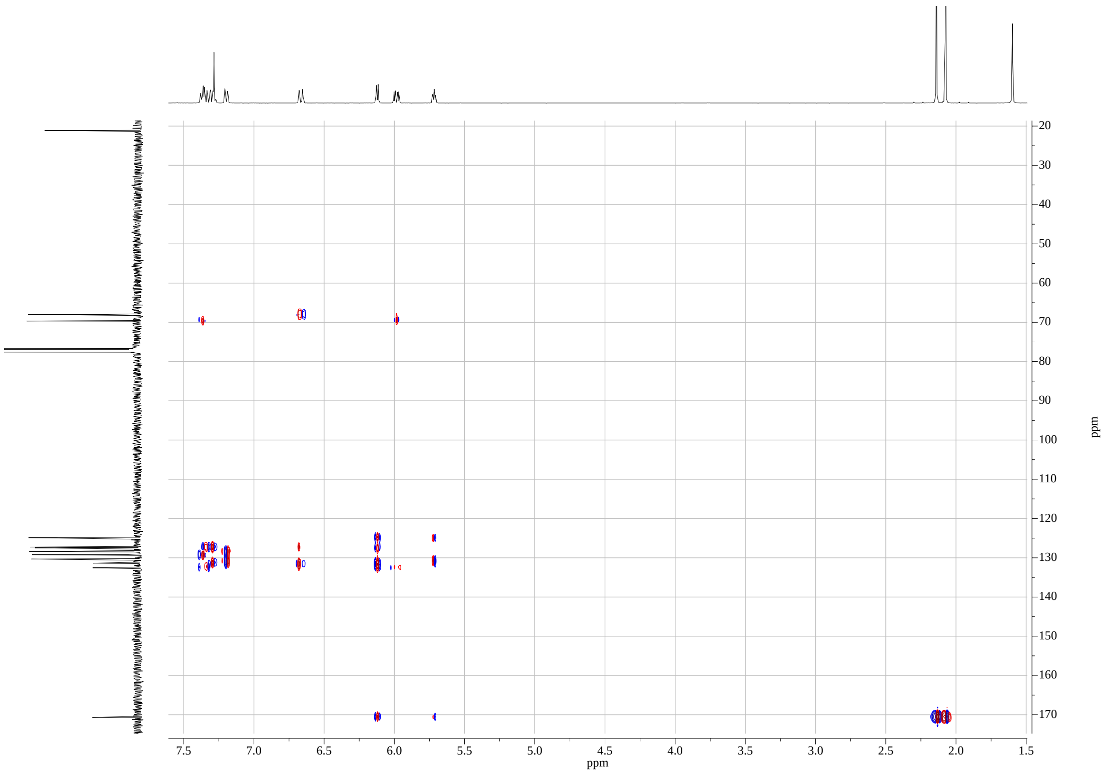

(1α,2α)-3,7-dimethyl-1,2-dihydronaphthalene-1,2-diyl diacetate (2b)

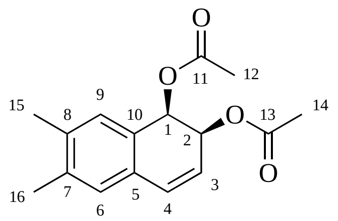

2b - ^{1}H NMR (400 MHz, $CDCl_3$)

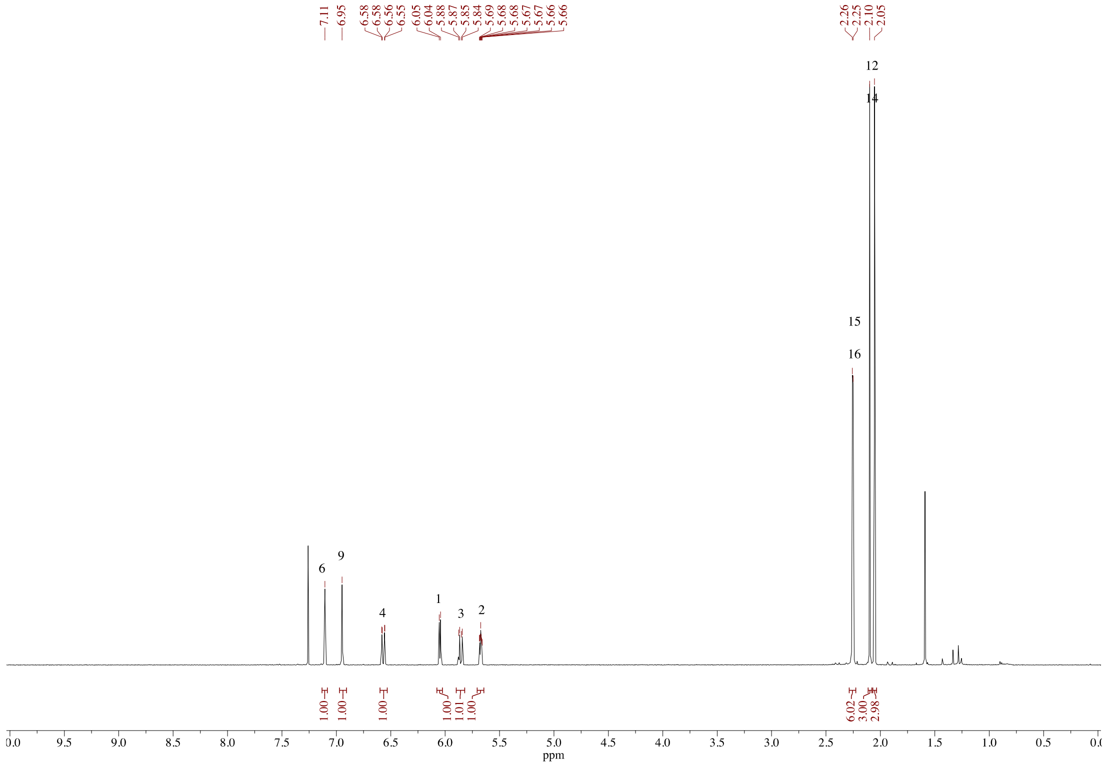

2b - ^{13}C NMR (100 MHz, $CDCl_3$)

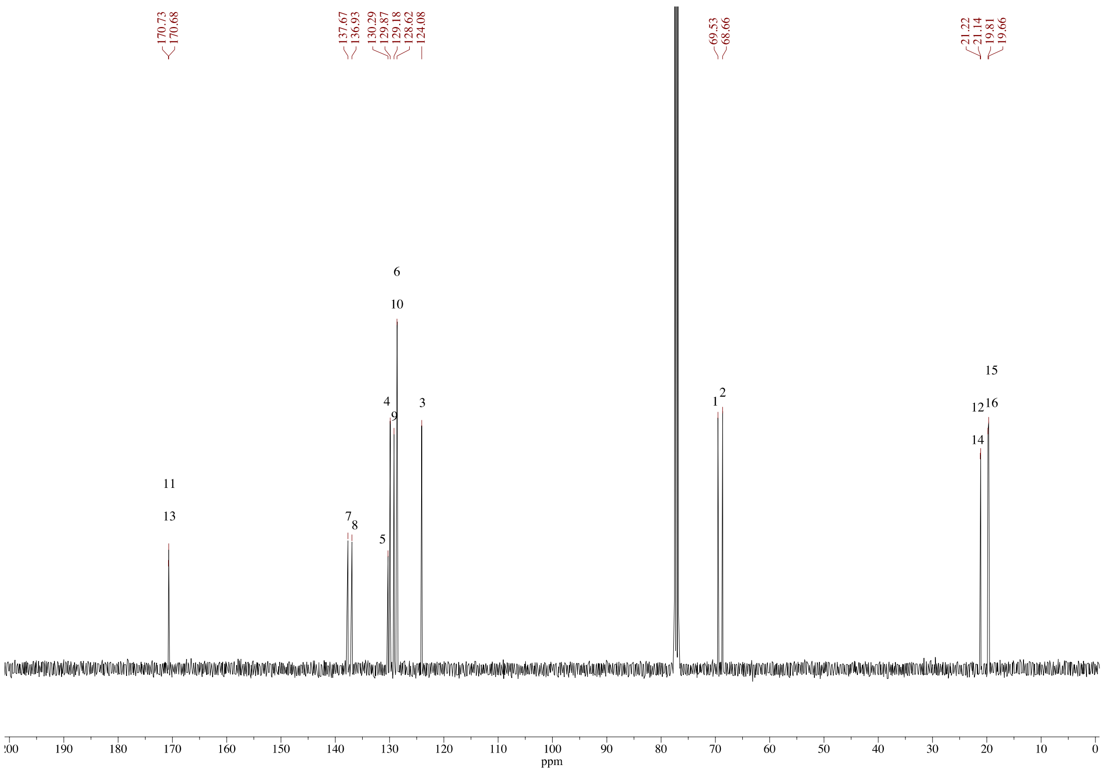

2b - DEPT ($CDCl_3$)

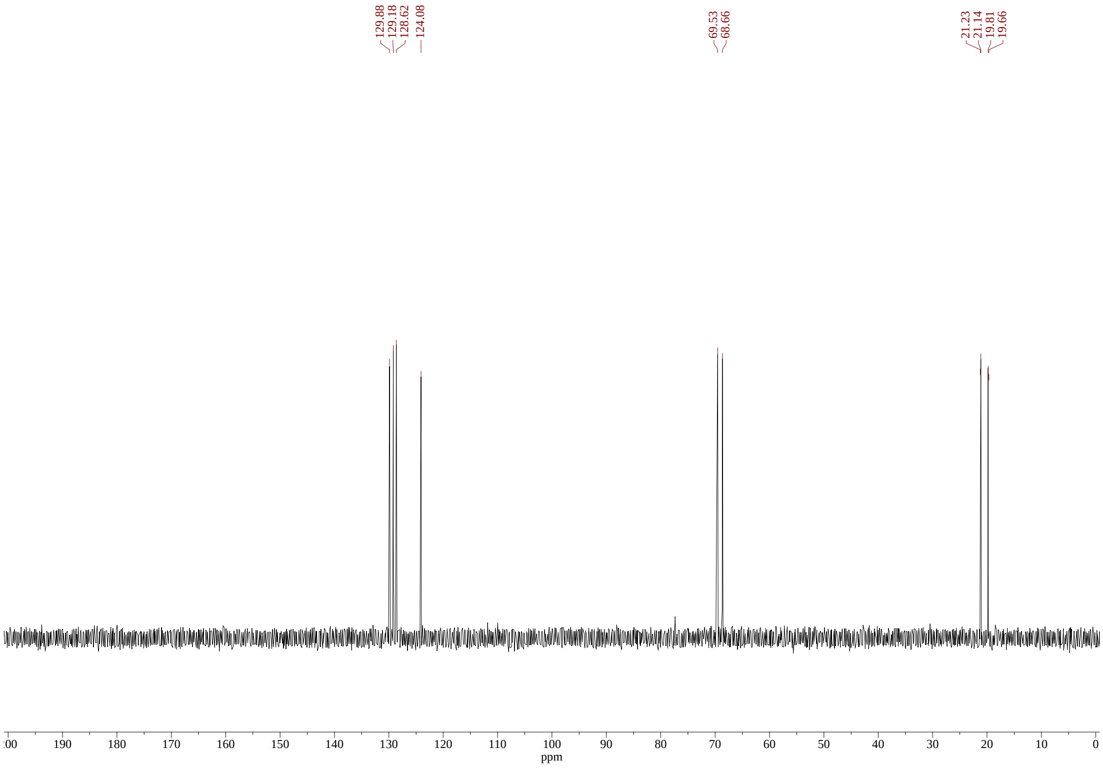

2b - DEPTQ ($CDCl_3$)

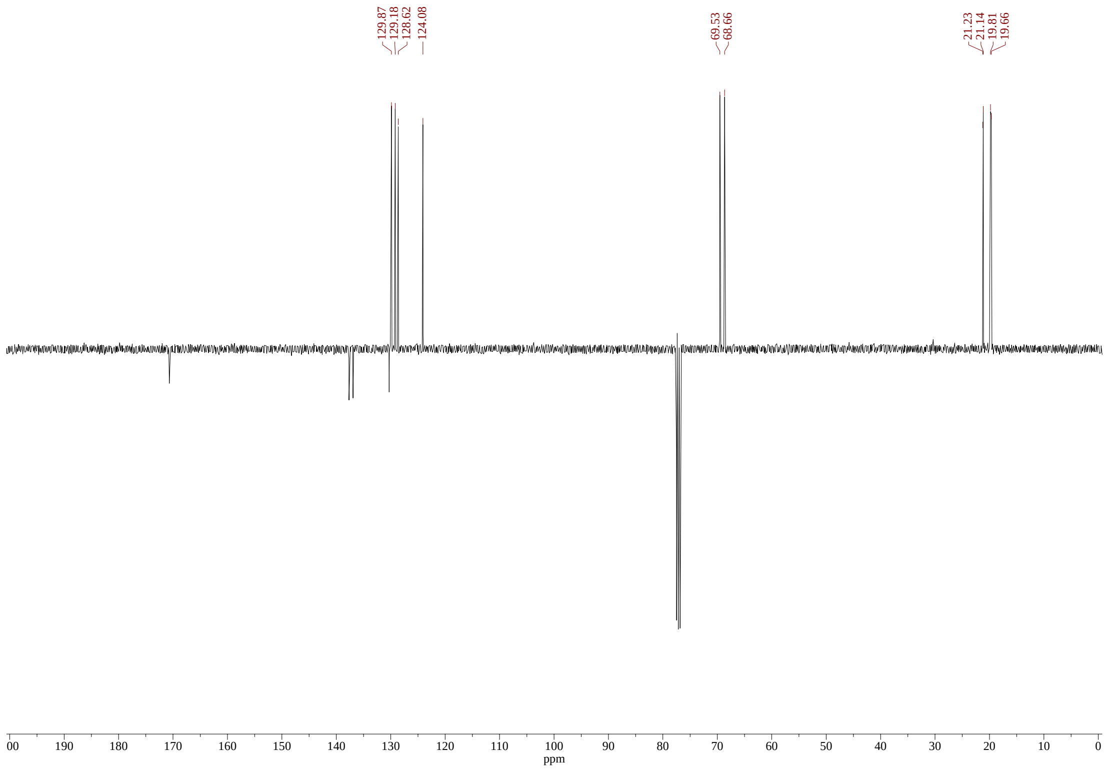

2b - ^1H-^1H COSY ($CDCl_3$)

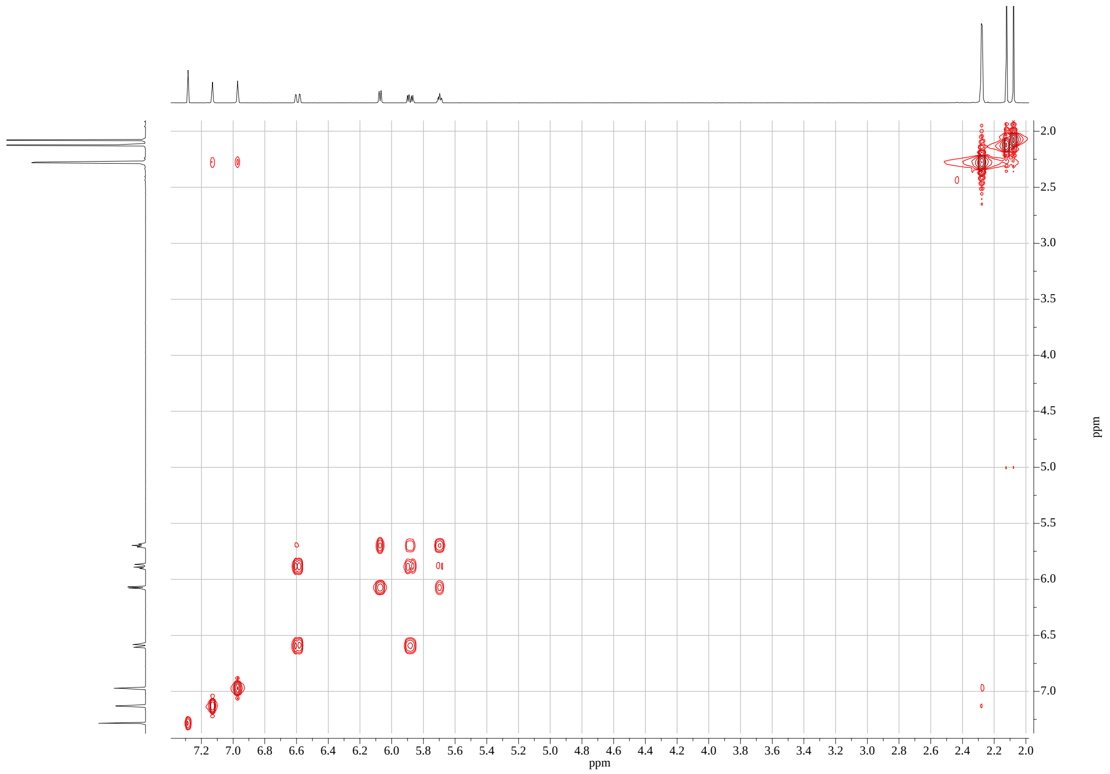

2b - ^{1}H-^{13}C HSQCED (CDCl$_3$)

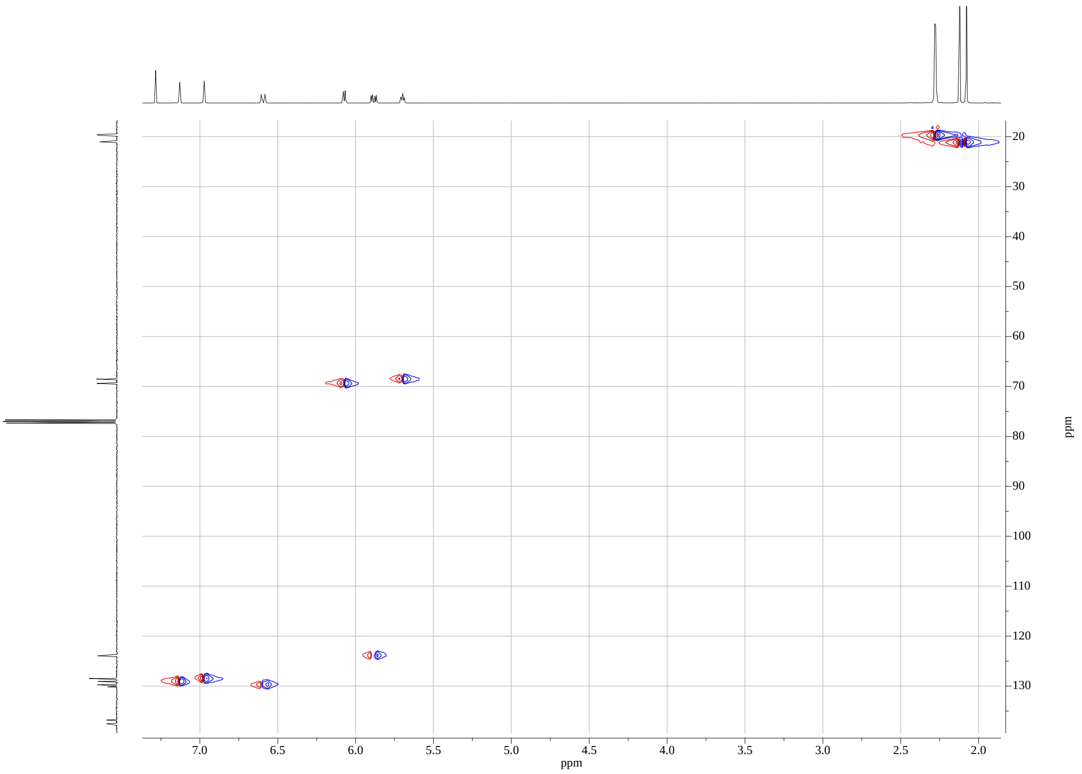

2b - ^{1}H-^{13}C HMBC (CDCl$_3$)

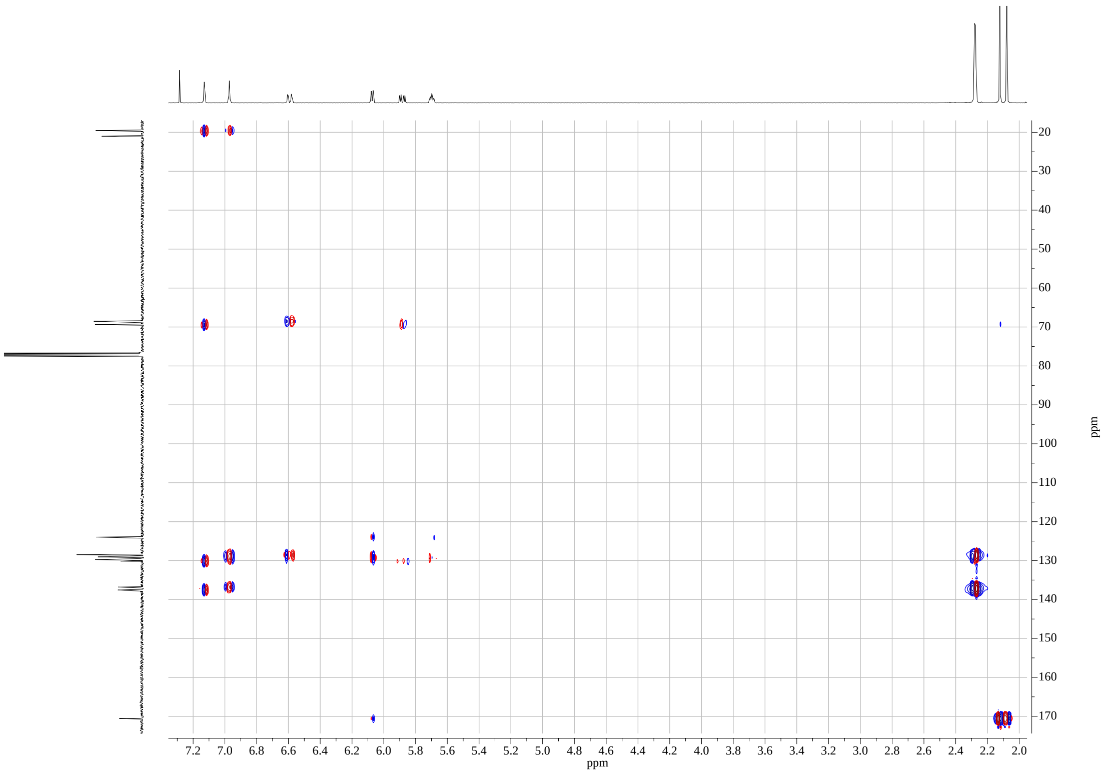

(1α,2α)-6,7-dimethyl-1,2-dihydronaphthalene-1,2-diyl diacetate (2c)

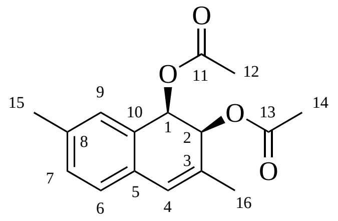

2c - ^{1}H NMR (400 MHz, $CDCl_3$)

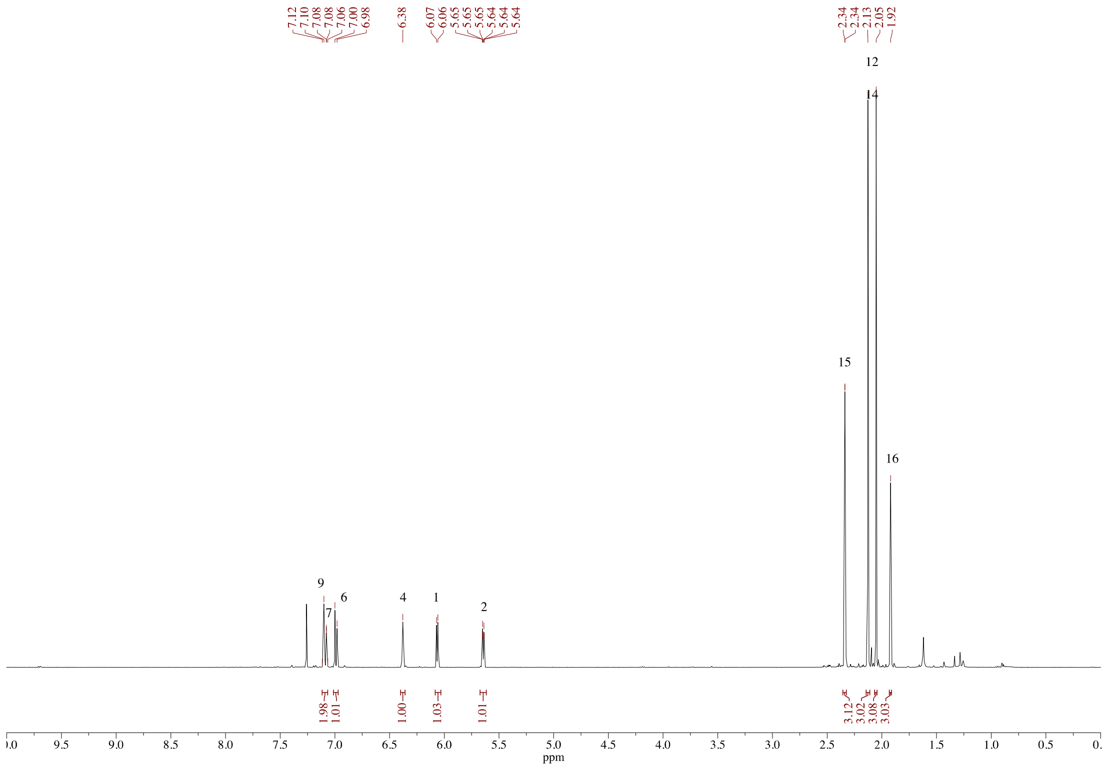

2c - ^{13}C NMR (100 MHz, $CDCl_3$)

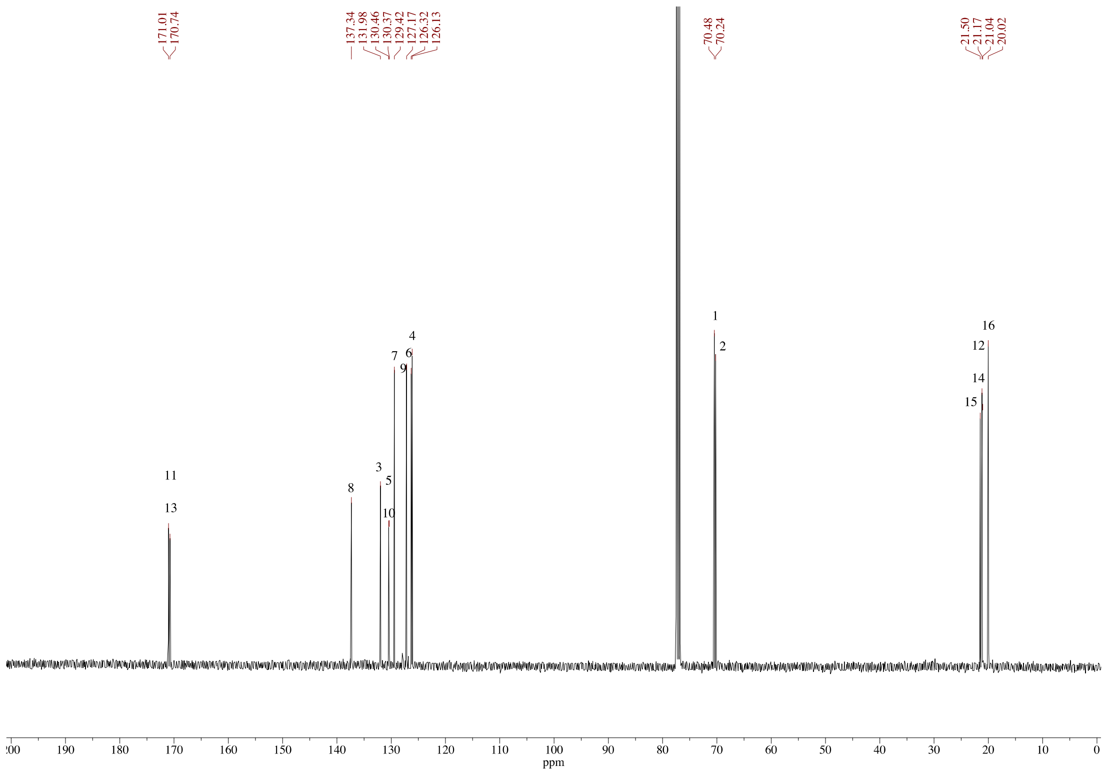

2c - DEPT ($CDCl_3$)

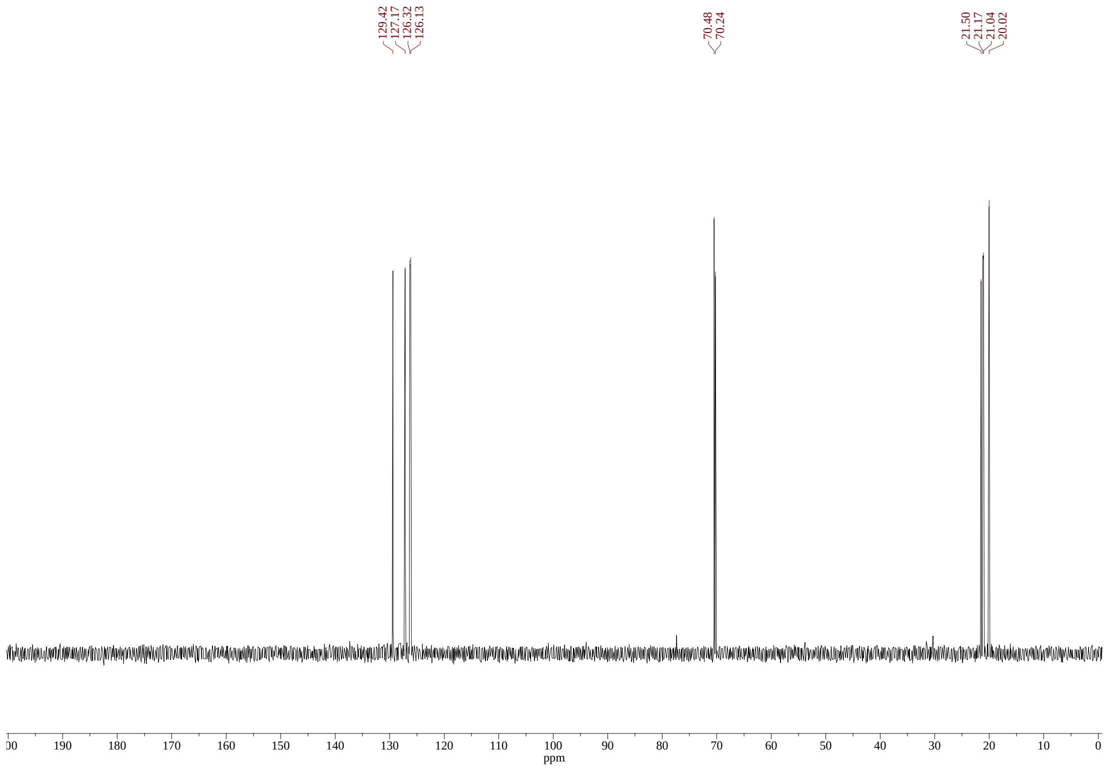

2c - DEPTQ ($CDCl_3$)

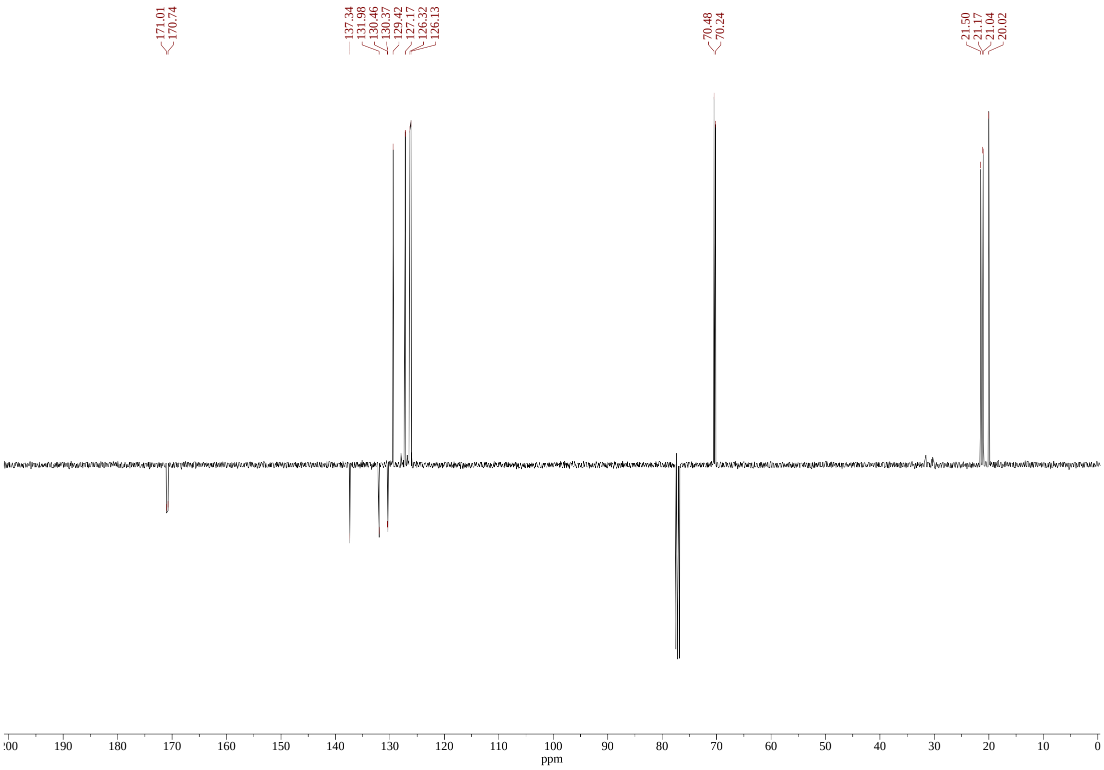

2c - ^1H-^1H COSY ($CDCl_3$)

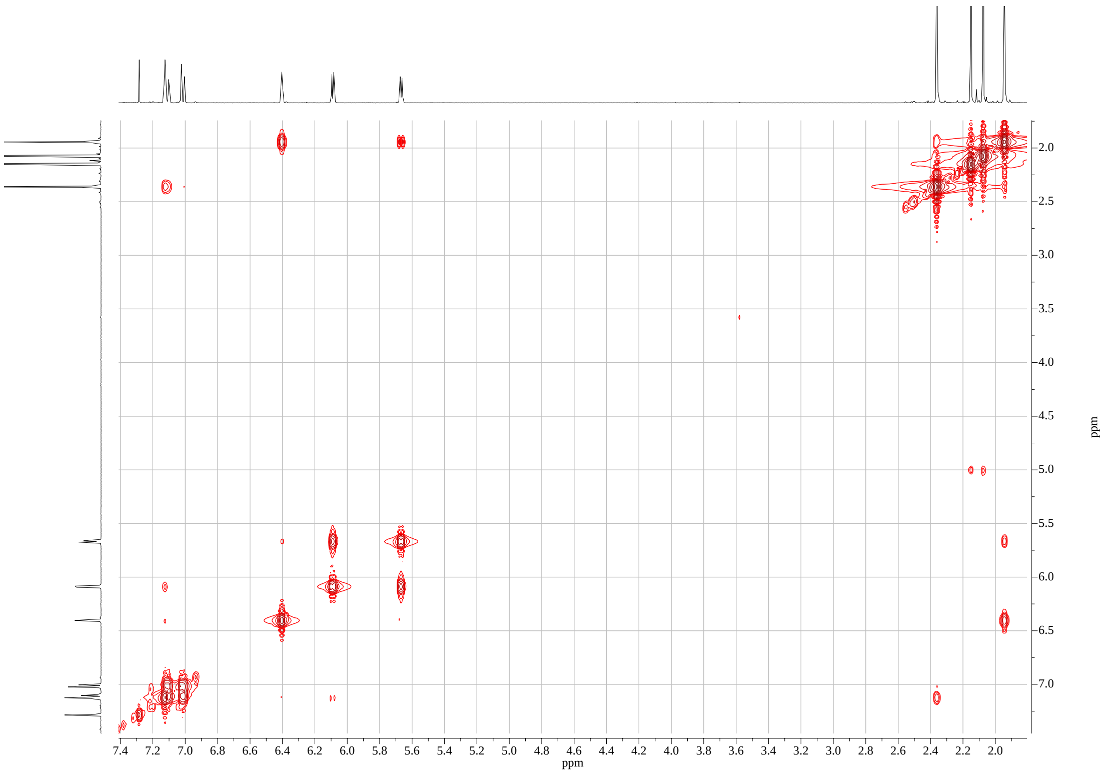

2c - ^{1}H-^{13}C HSQCED ($CDCl_3$)

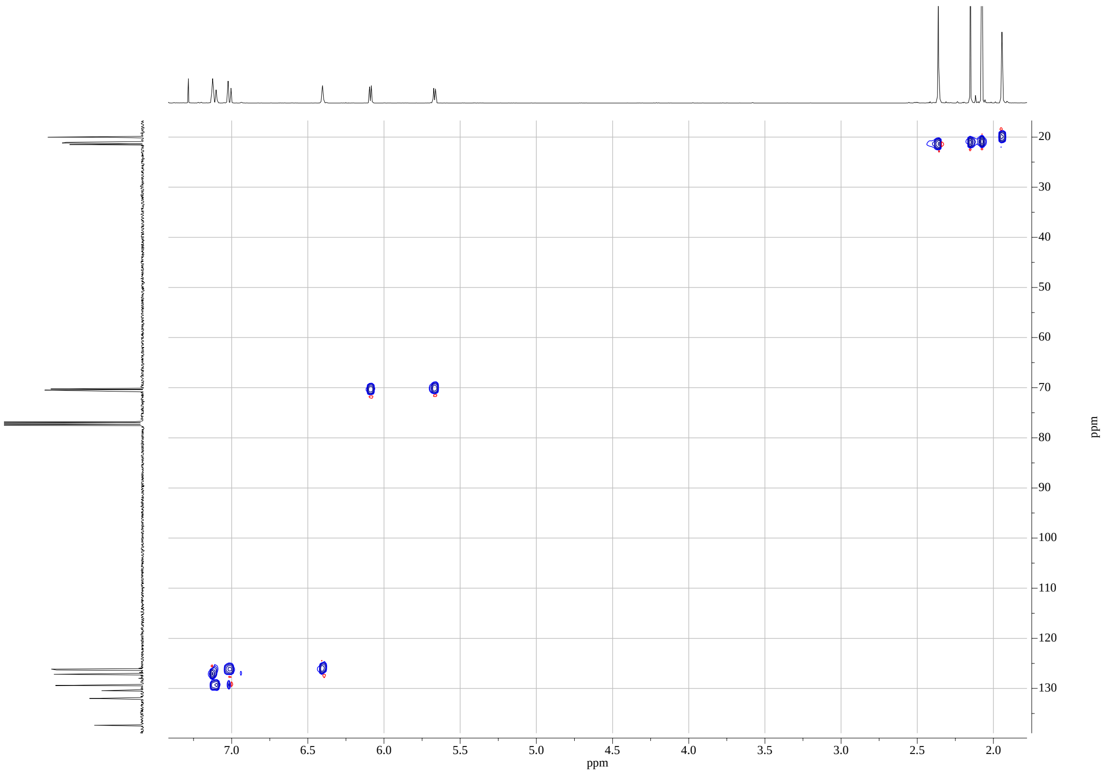

2c - ^{1}H-^{13}C HMBC ($CDCl_3$)

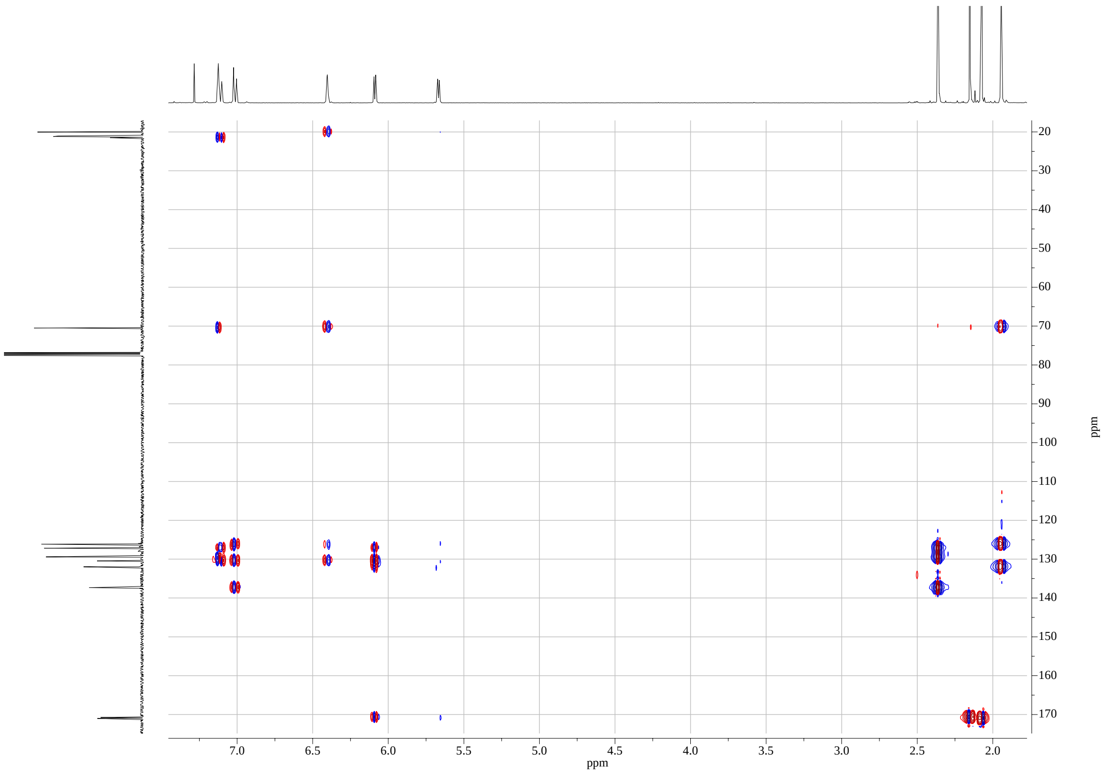

(1α,2α)-1,2-dihydronaphthalene-1,2,6,7-tetrayl tetraacetate (2d)

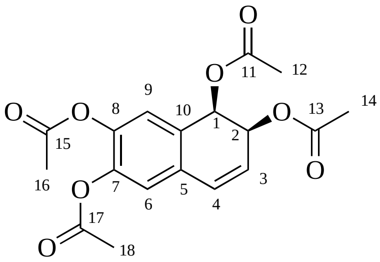

2d - ^{1}H NMR (400 MHz, $CDCl_3$)

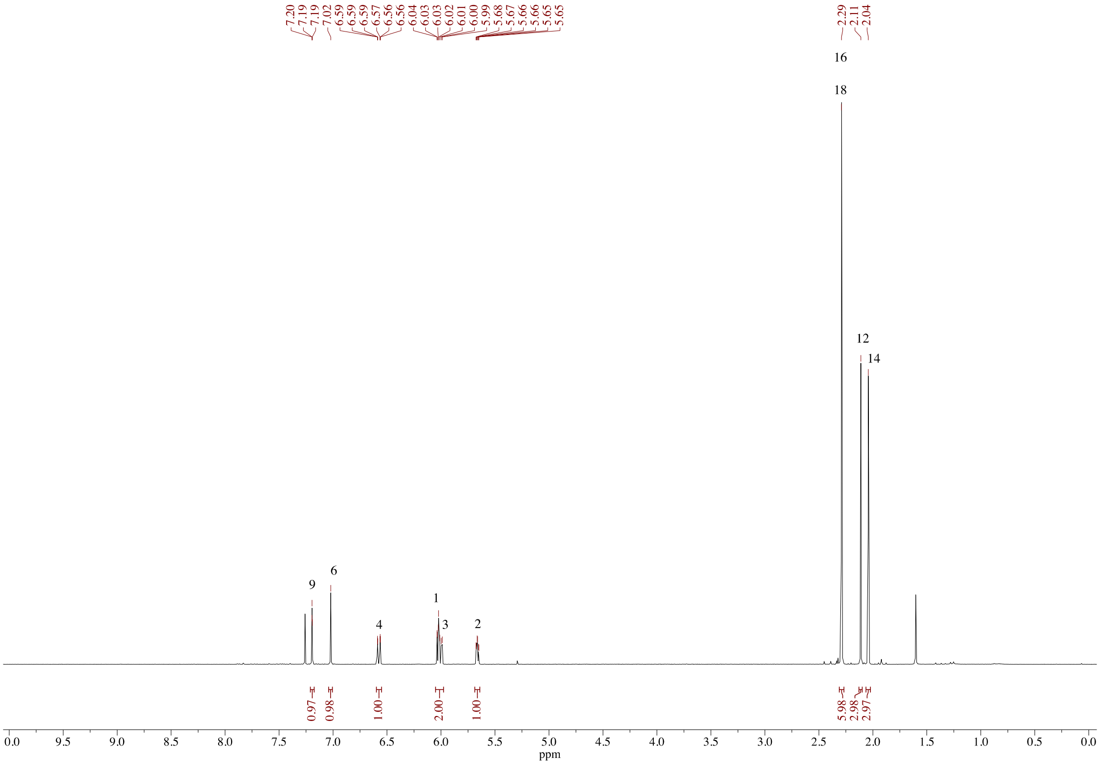

2d - ^{13}C NMR (100 MHz, $CDCl_3$)

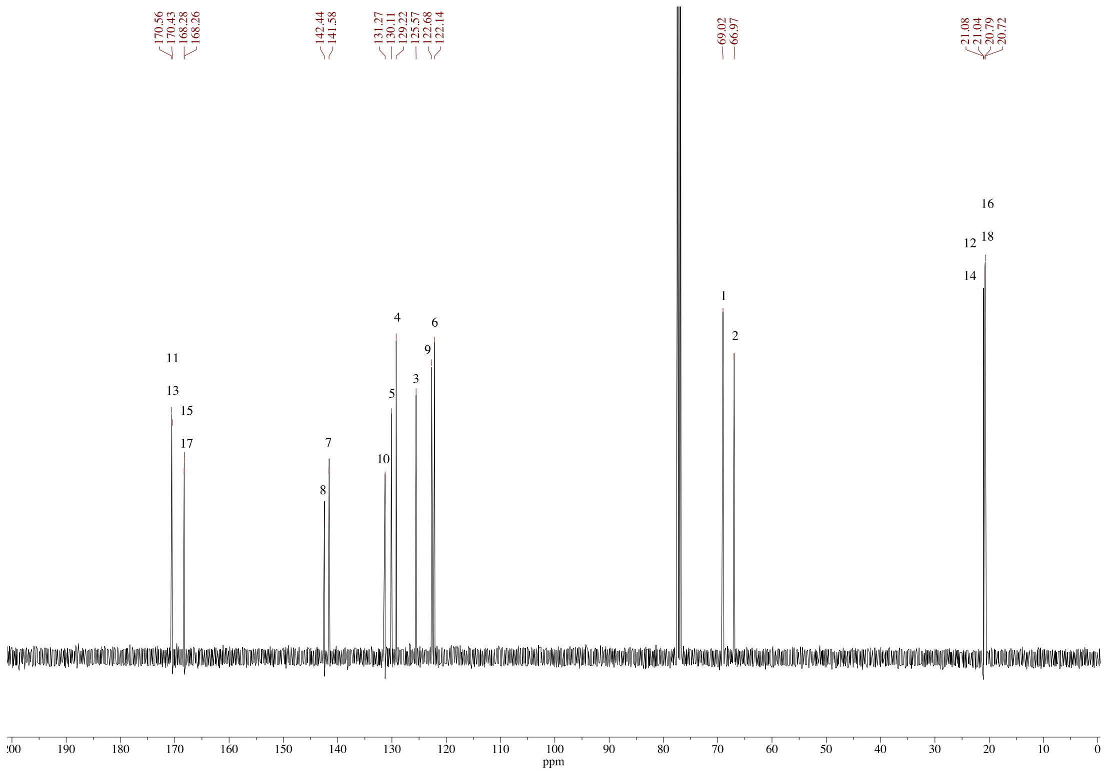

2d - DEPT ($CDCl_3$)

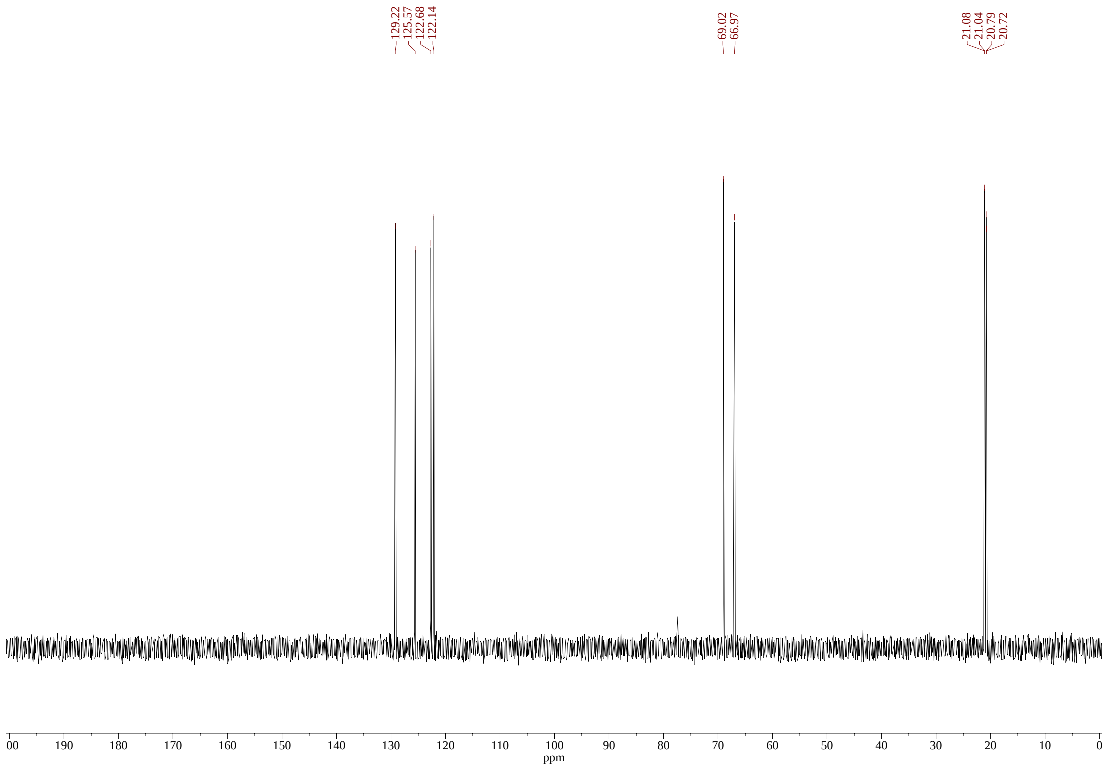

2d - DEPTQ ($CDCl_3$)

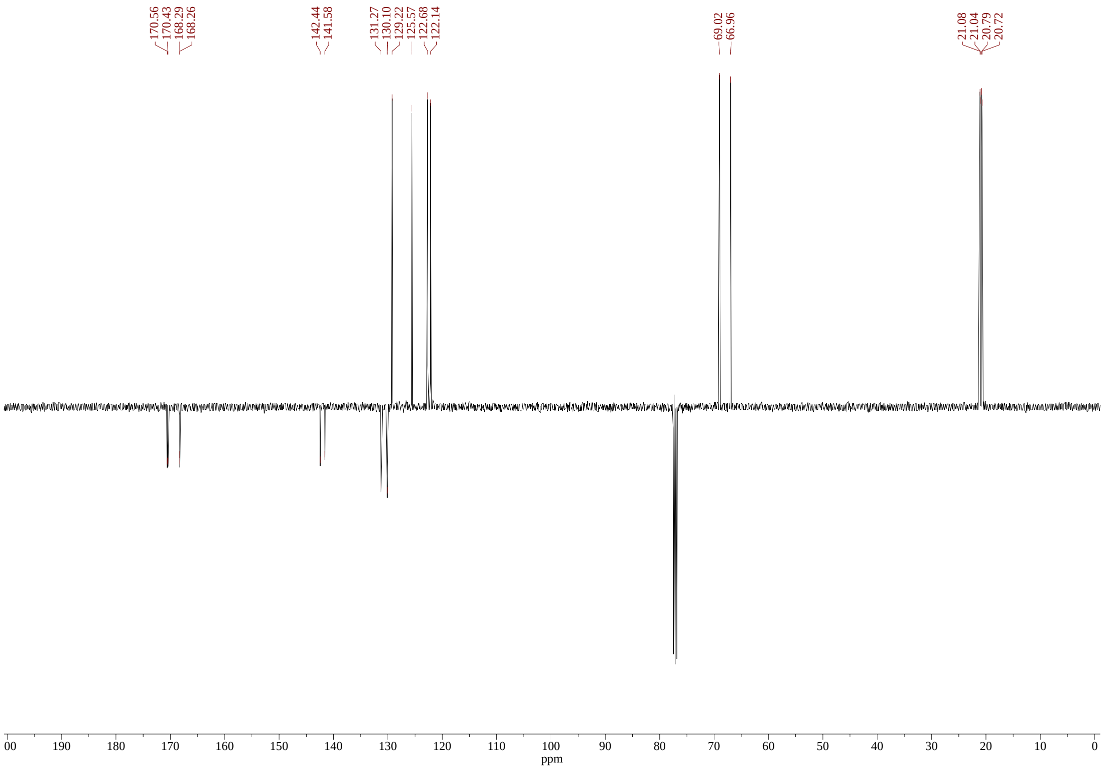

2d - ^1H-^1H COSY ($CDCl_3$)

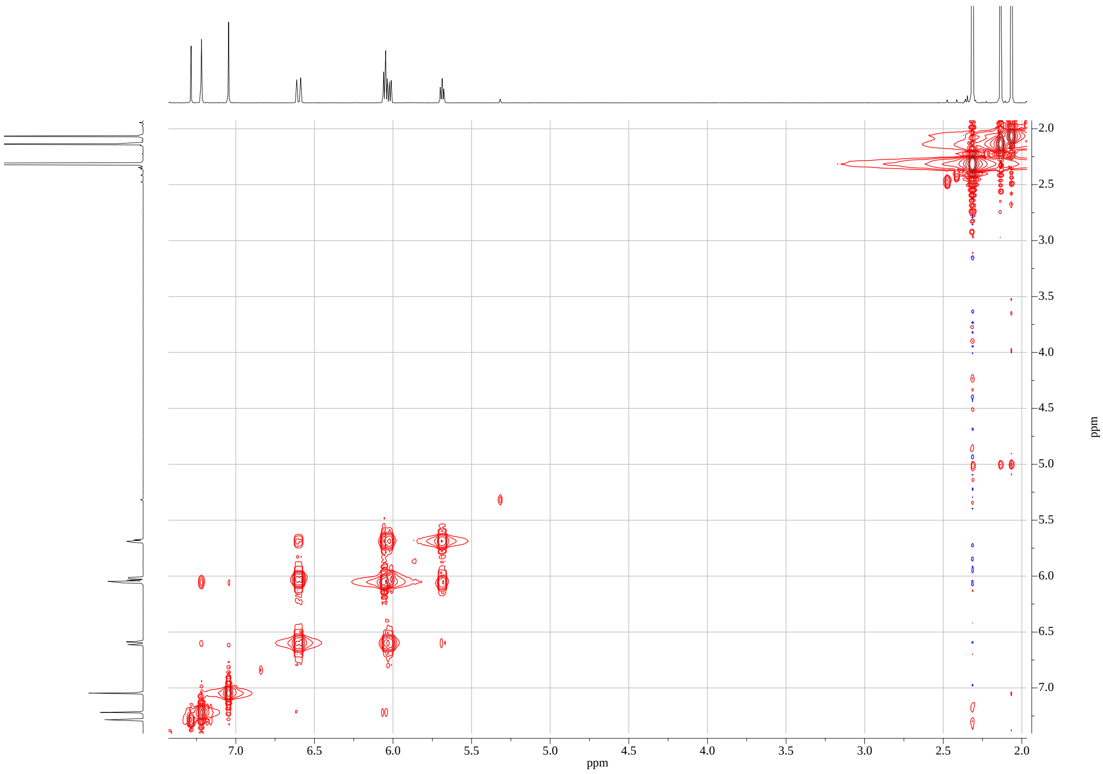

2d - ^{1}H-^{13}C HSQCED ($CDCl_3$)

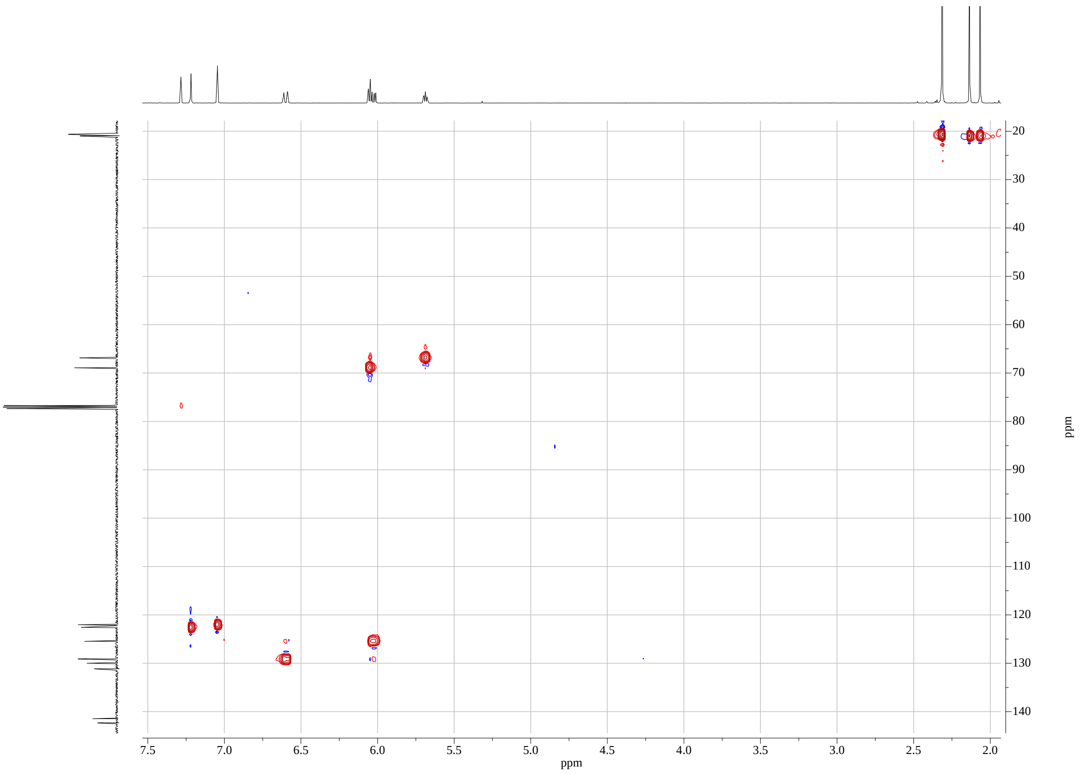

2d - ^{1}H-^{13}C HMBC ($CDCl_3$)

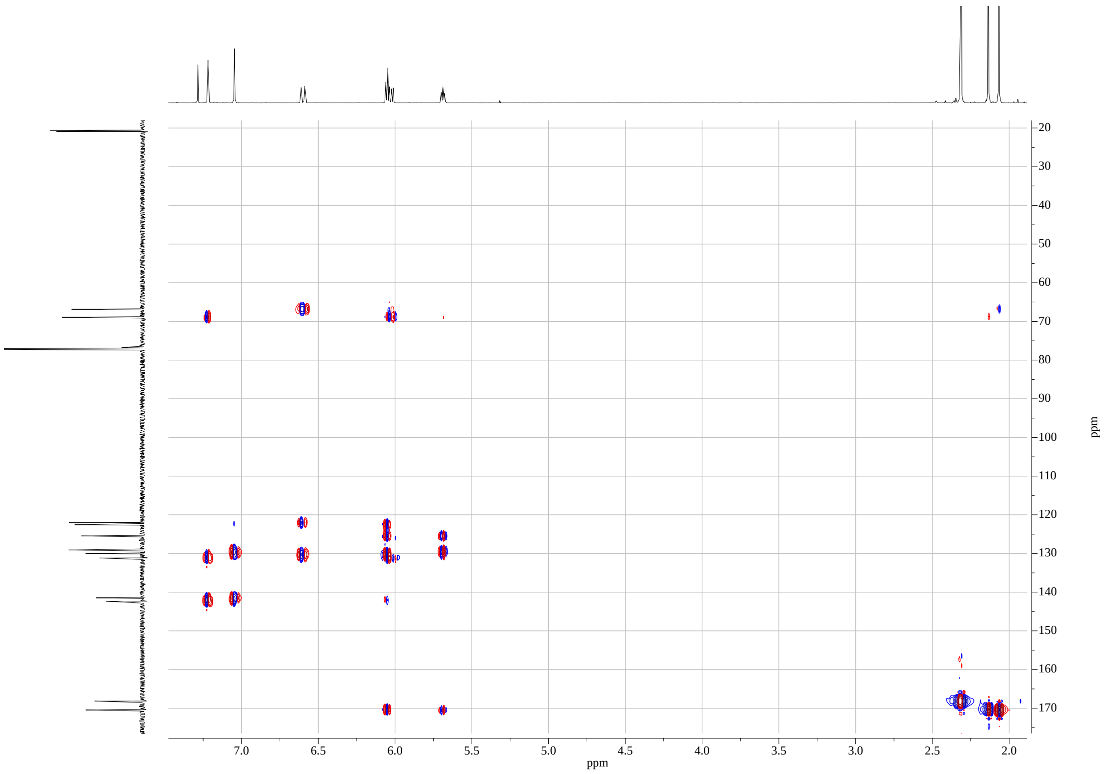

(1α,2α)-7-formyl-1,2-dihydronaphthalene-1,2-diyl diacetate (2s)

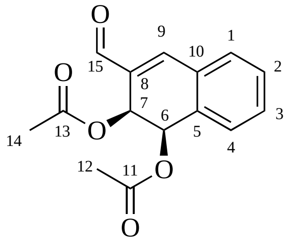

2s - ^{1}H NMR (400 MHz, $CDCl_3$)

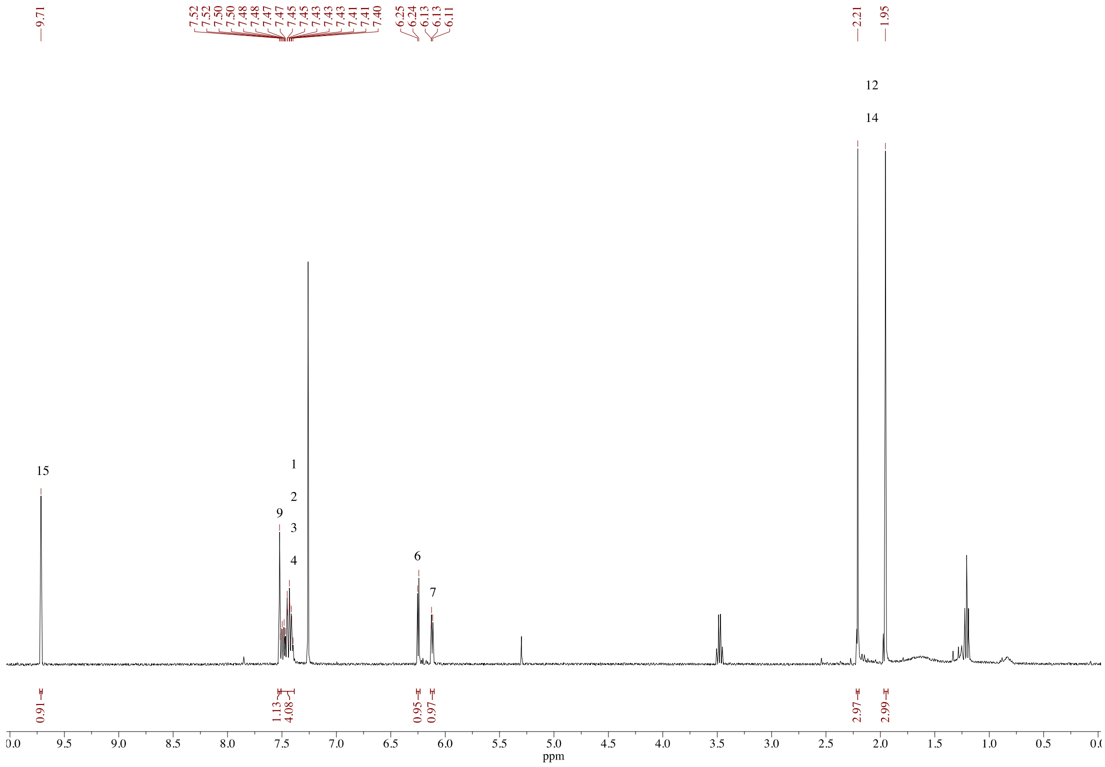

2s - ^{13}C NMR (100 MHz, $CDCl_3$)

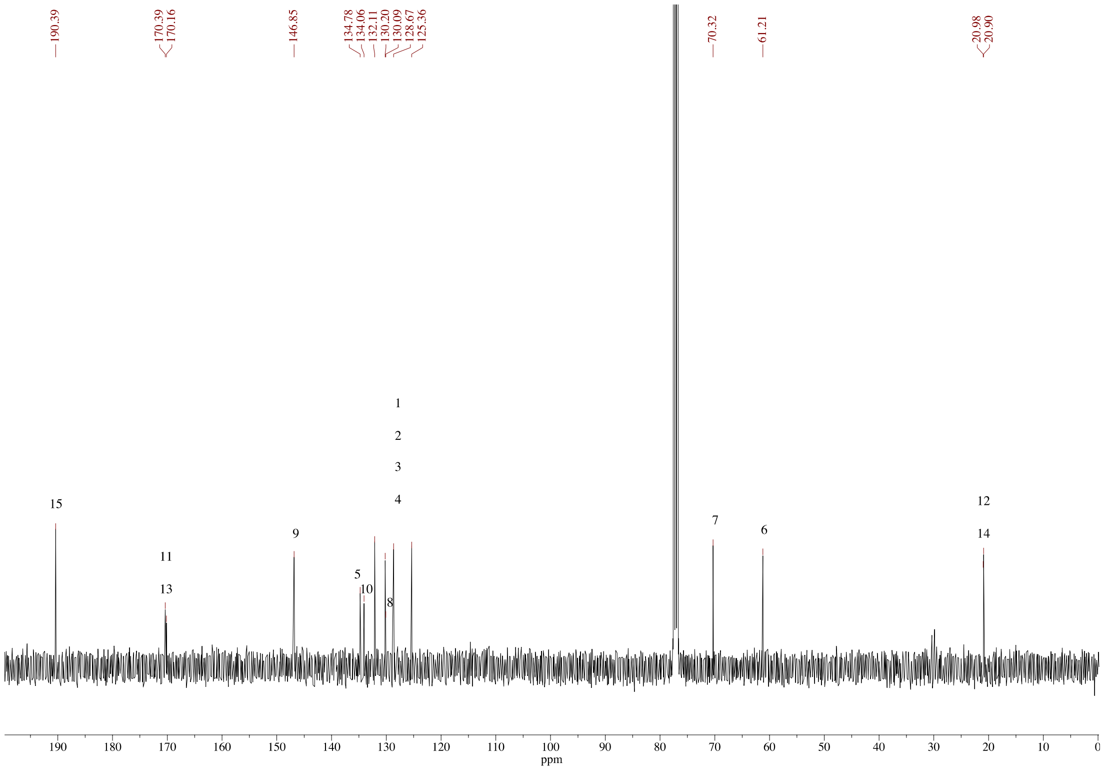

2s - DEPT ($CDCl_3$)

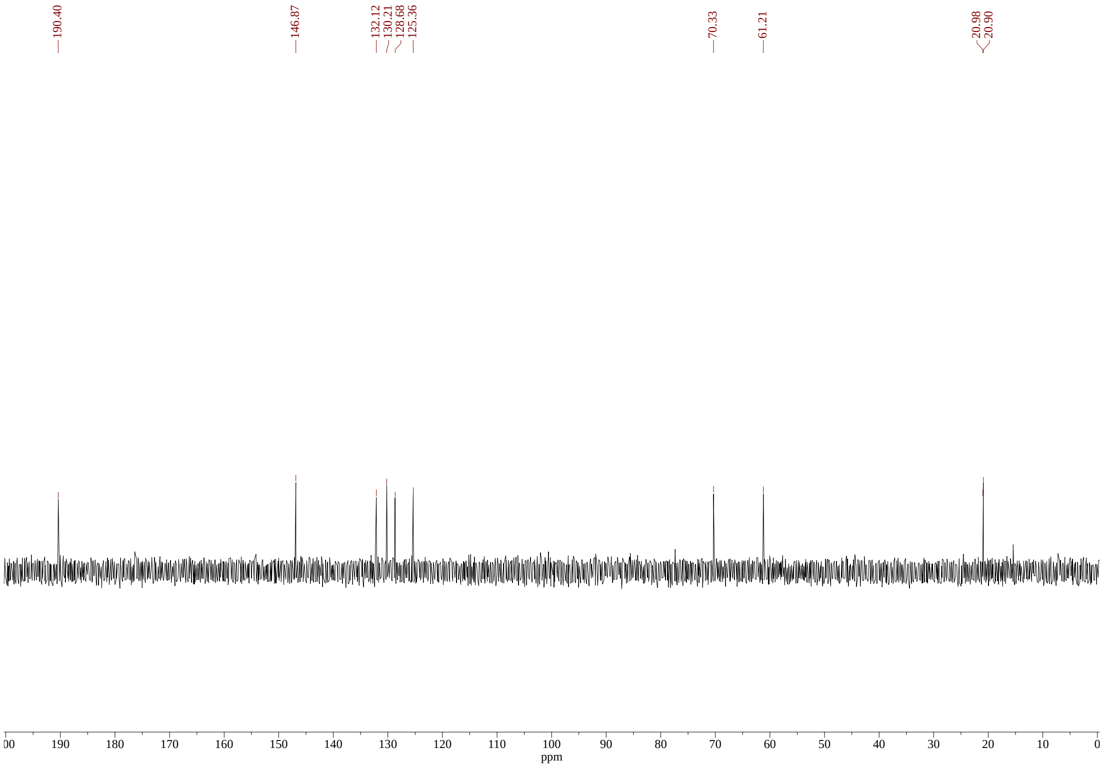

2s - DEPTQ ($CDCl_3$)

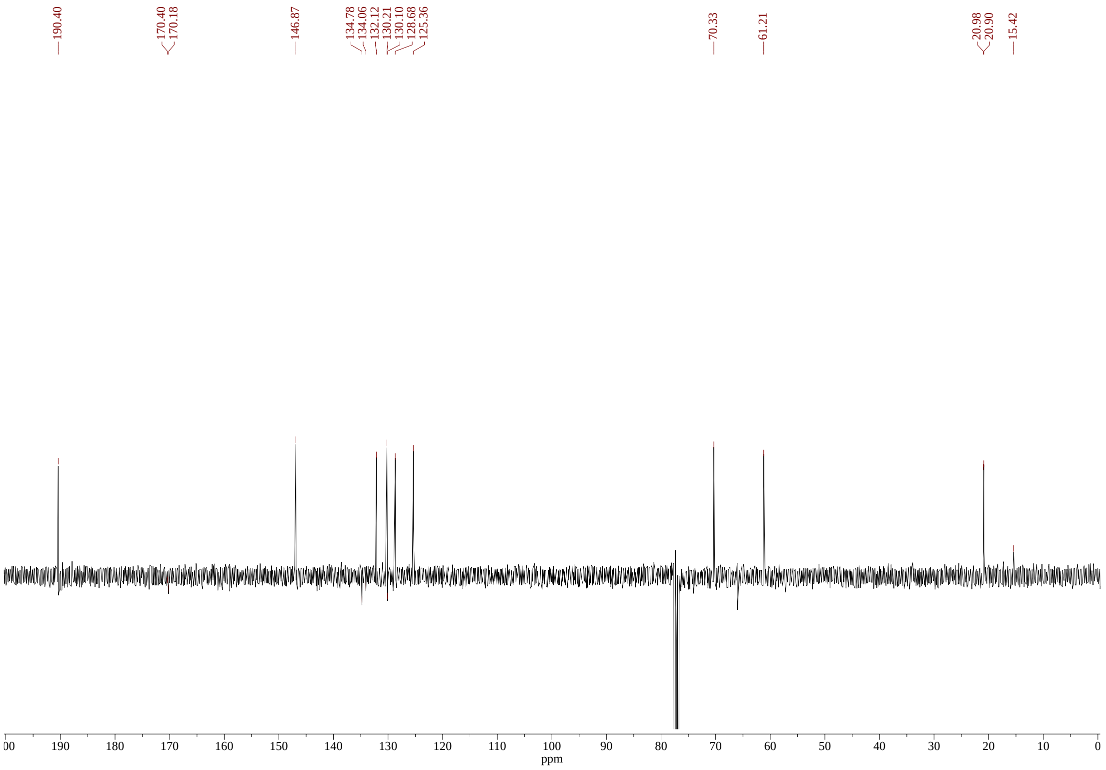

2s - ^{1}H-^{1}H COSY ($CDCl_3$)

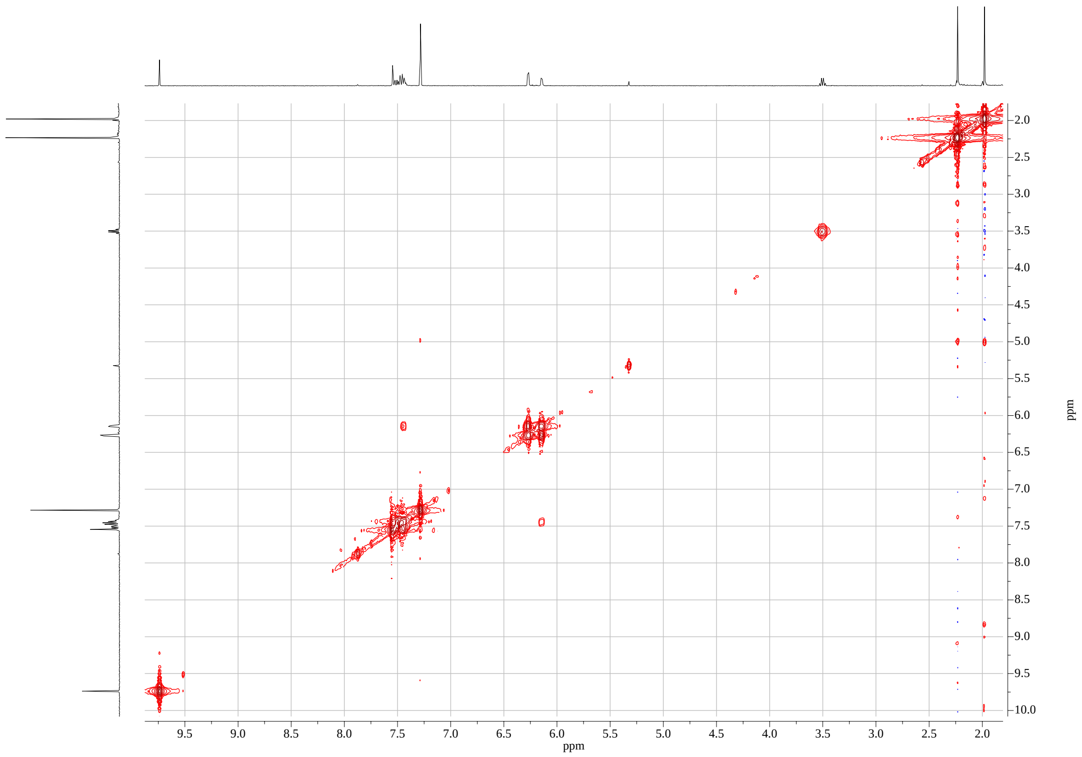

2s - ^{1}H-^{13}C HSQCED ($CDCl_3$)

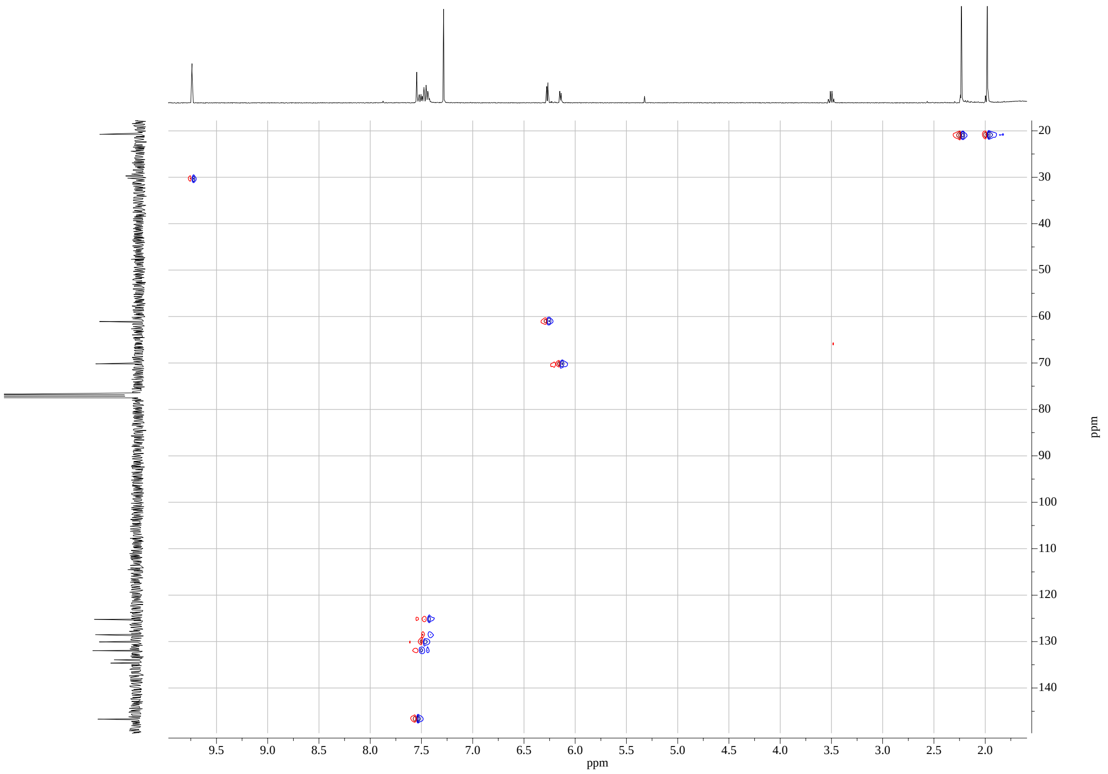

2s - ^{1}H-^{13}C HMBC ($CDCl_3$)

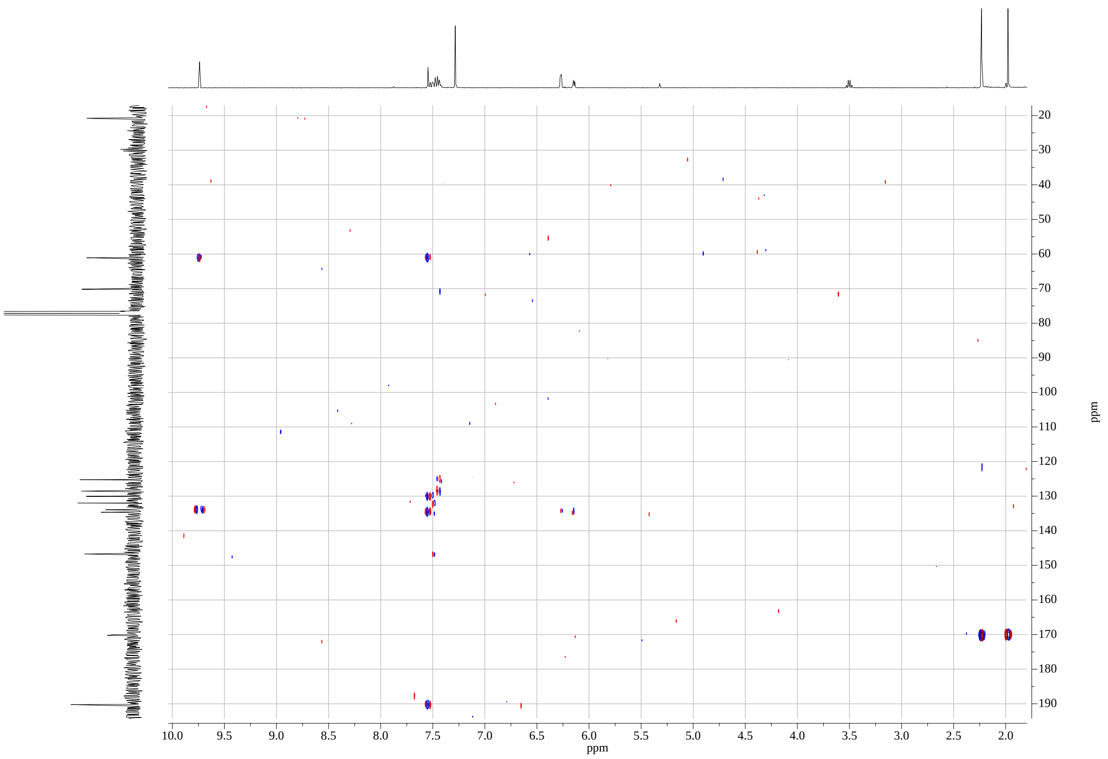

(1α,2α)-3,7-dibromo-1,2-dihydronaphthalene-1,2-diyl diacetate (2e)

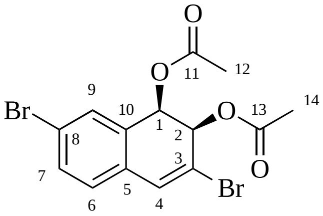

2e - ^{1}H NMR (400 MHz, $CDCl_3$)

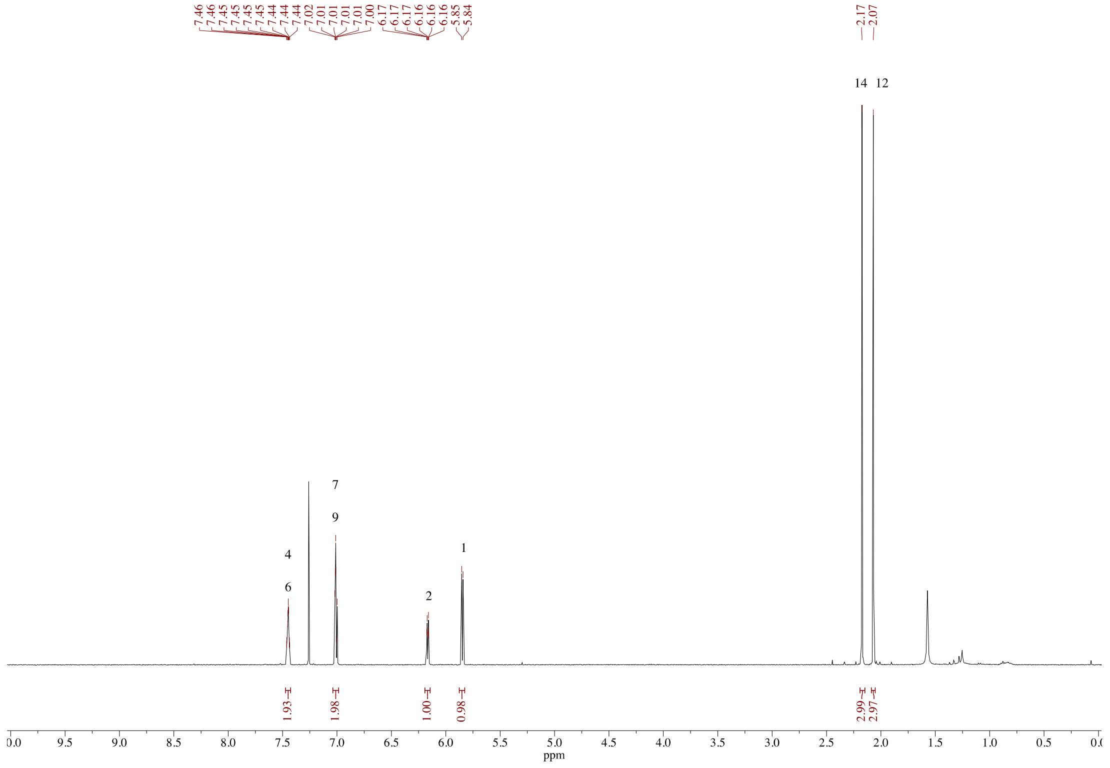

2e - ^{13}C NMR (100 MHz, $CDCl_3$)

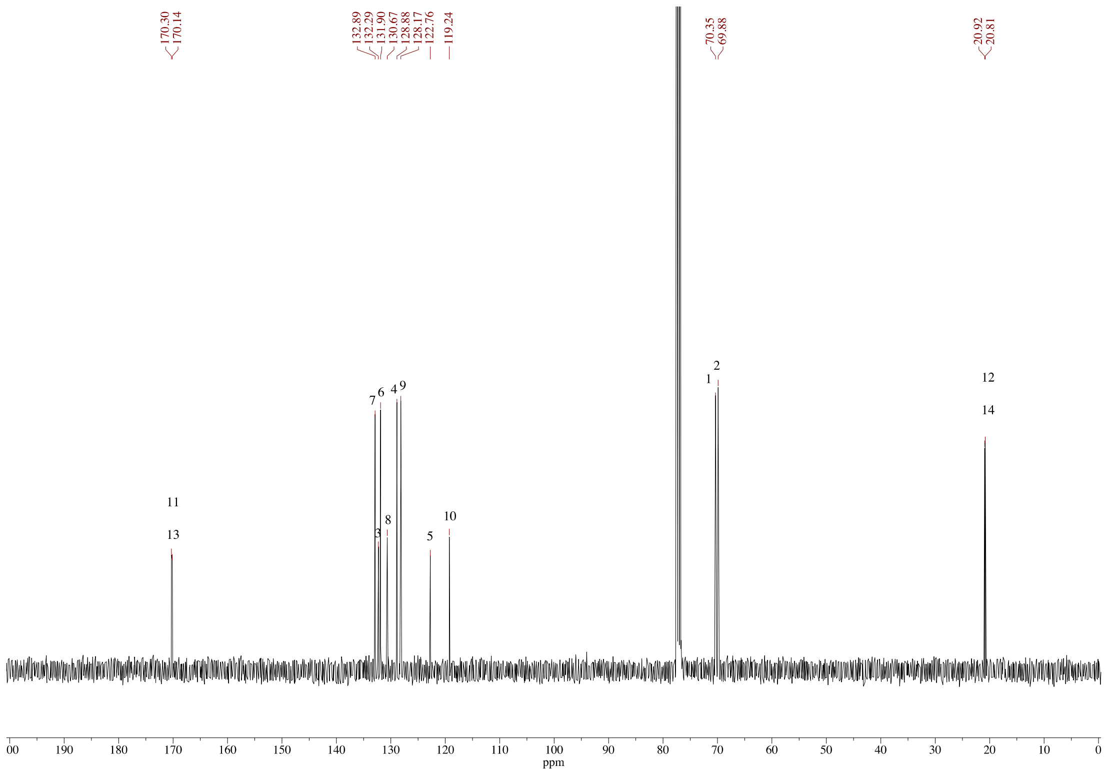

2e - DEPT ($CDCl_3$)

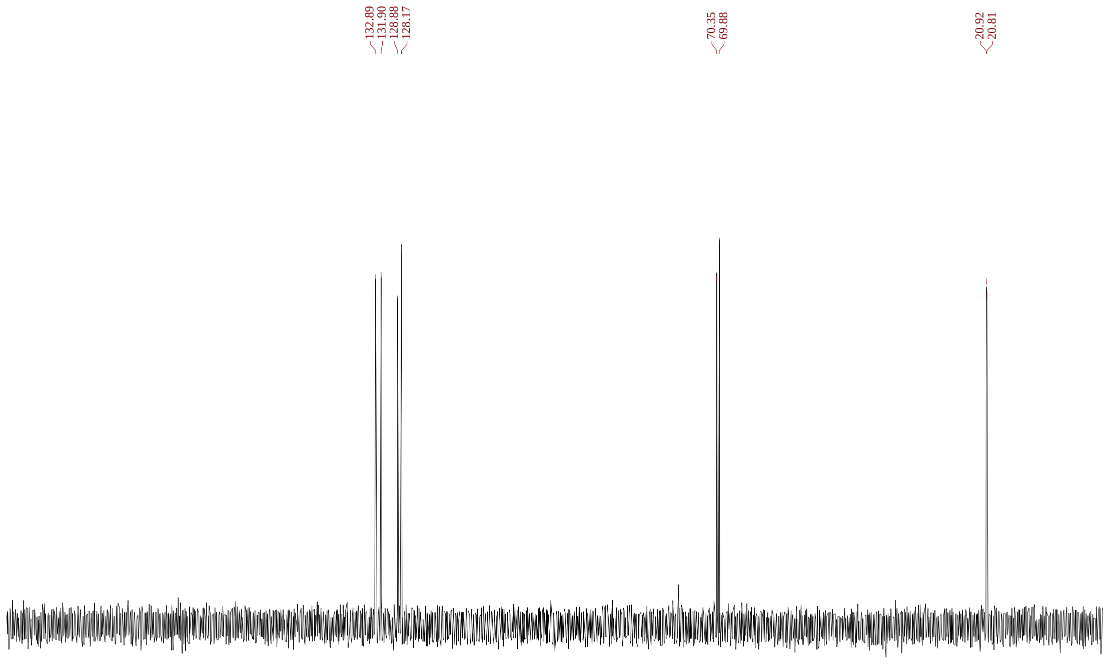

2e - DEPTQ ($CDCl_3$)

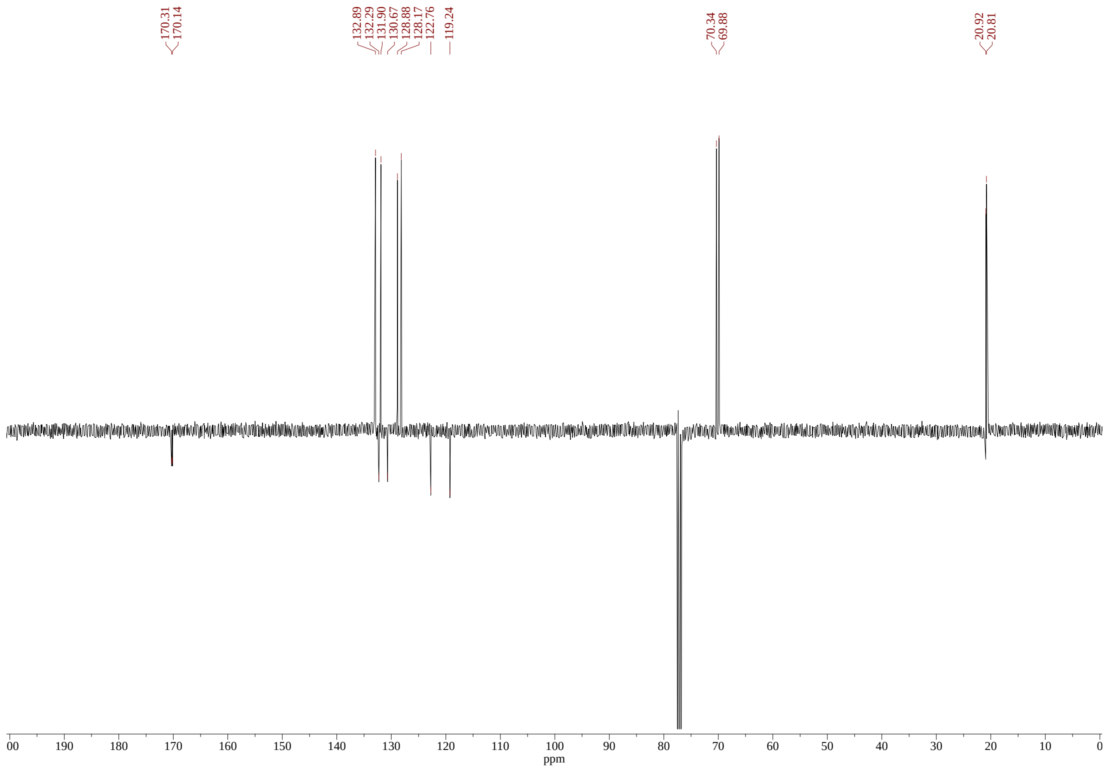

2e - ^1H-^1H COSY ($CDCl_3$)

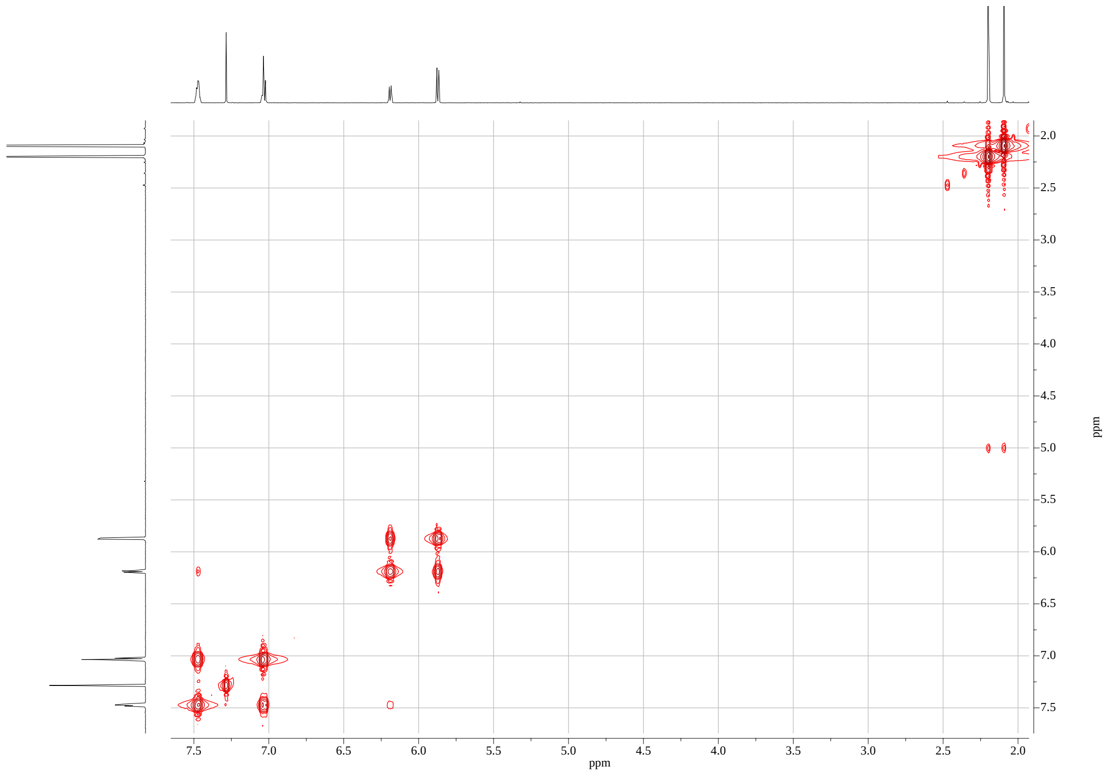

2e - ^{1}H-^{13}C HSQCED ($CDCl_3$)

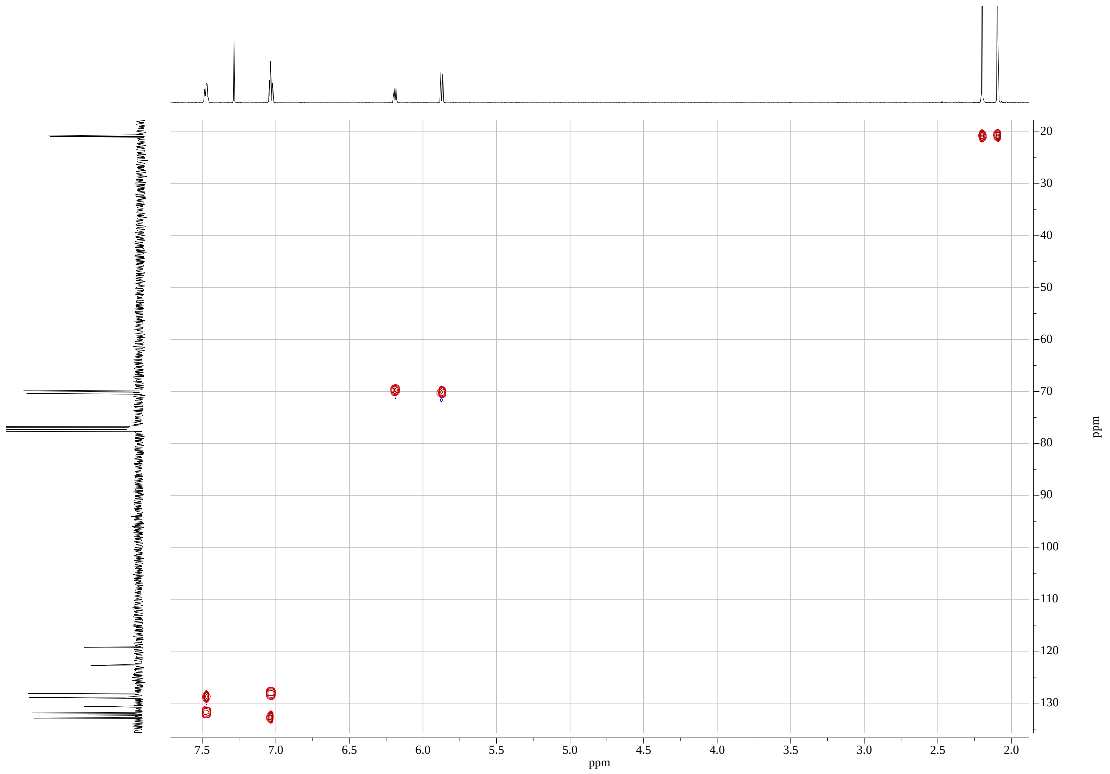

2e - ^{1}H-^{13}C HMBC ($CDCl_3$)

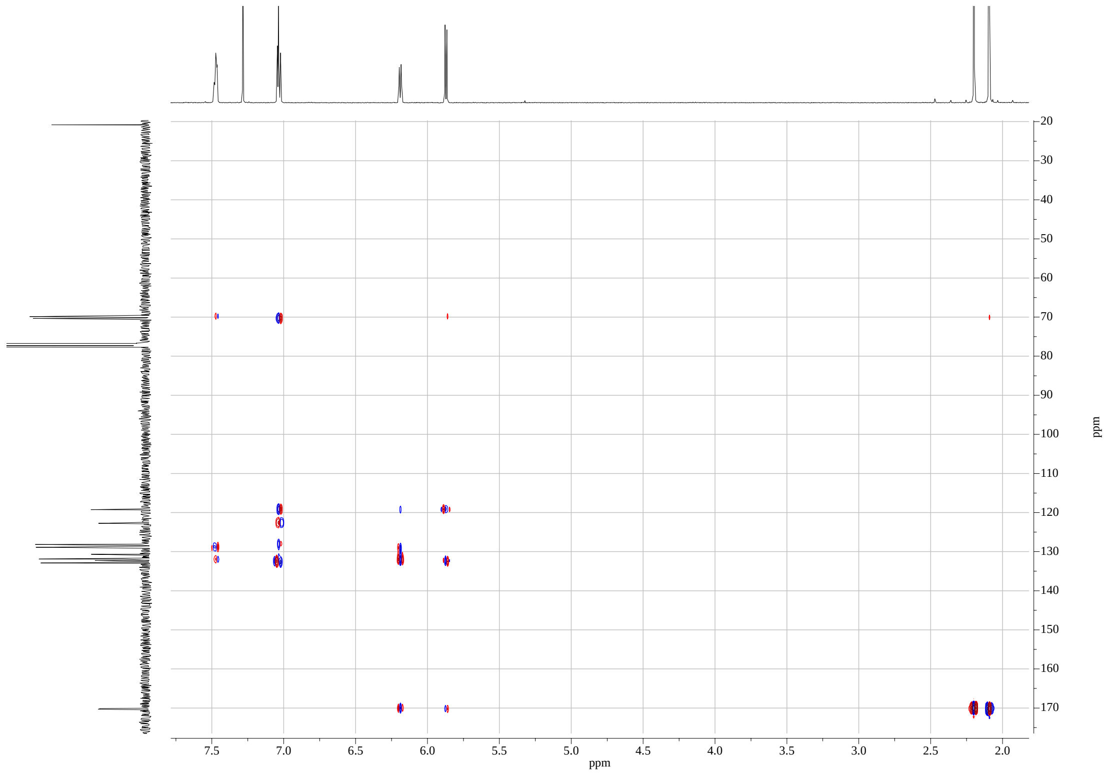

(1α,2α,3α,4α)-1,2,3,4-tetrahydronaphthalene-1,2,3,4-tetraol (3a^{syn})

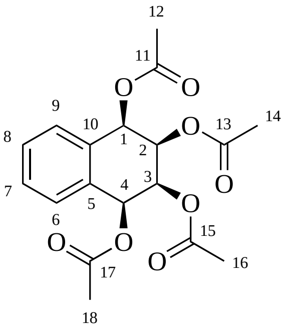

3a^{syn} - ^{1}H NMR (400 MHz, CDCl$_3$)

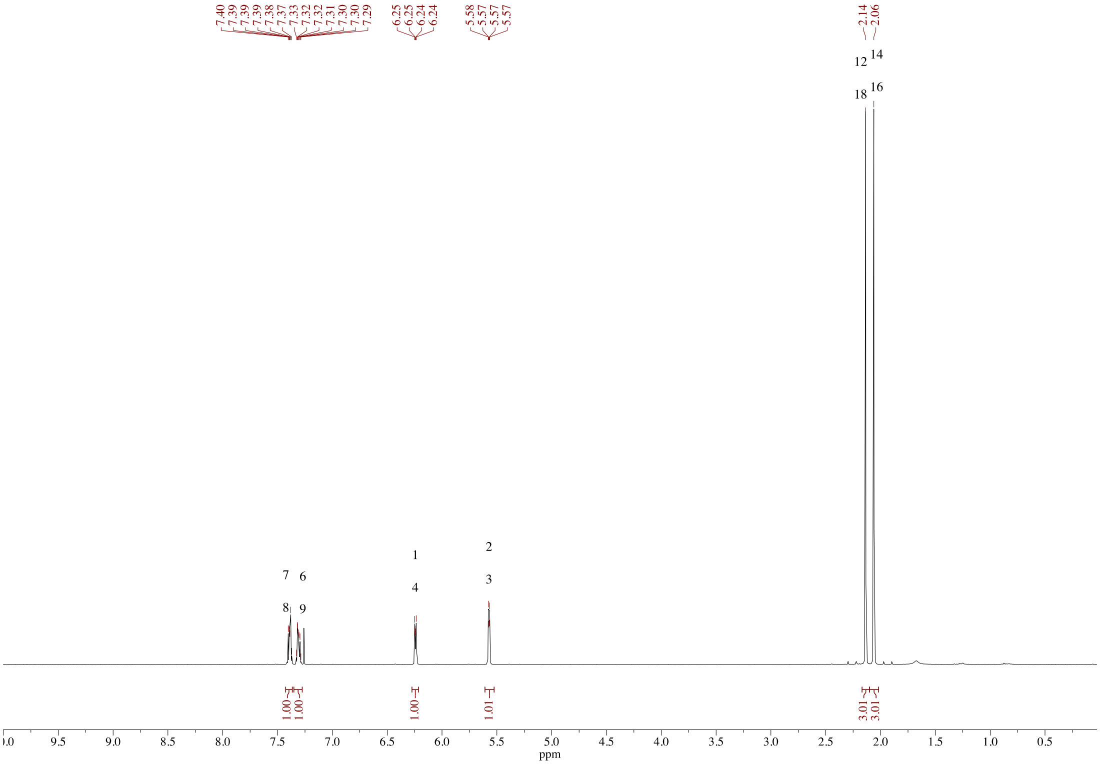

3a^{syn} - ^{13}C NMR (100 MHz, $CDCl_3$)

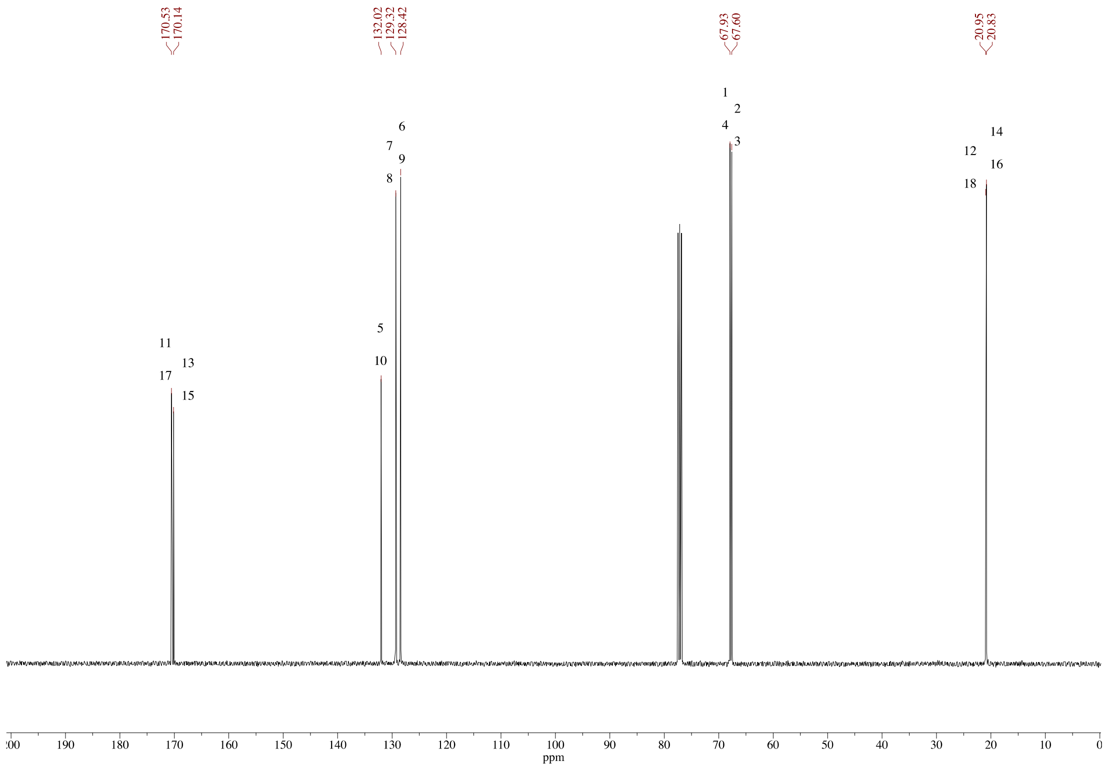

3a^{syn} - DEPT ($CDCl_3$)

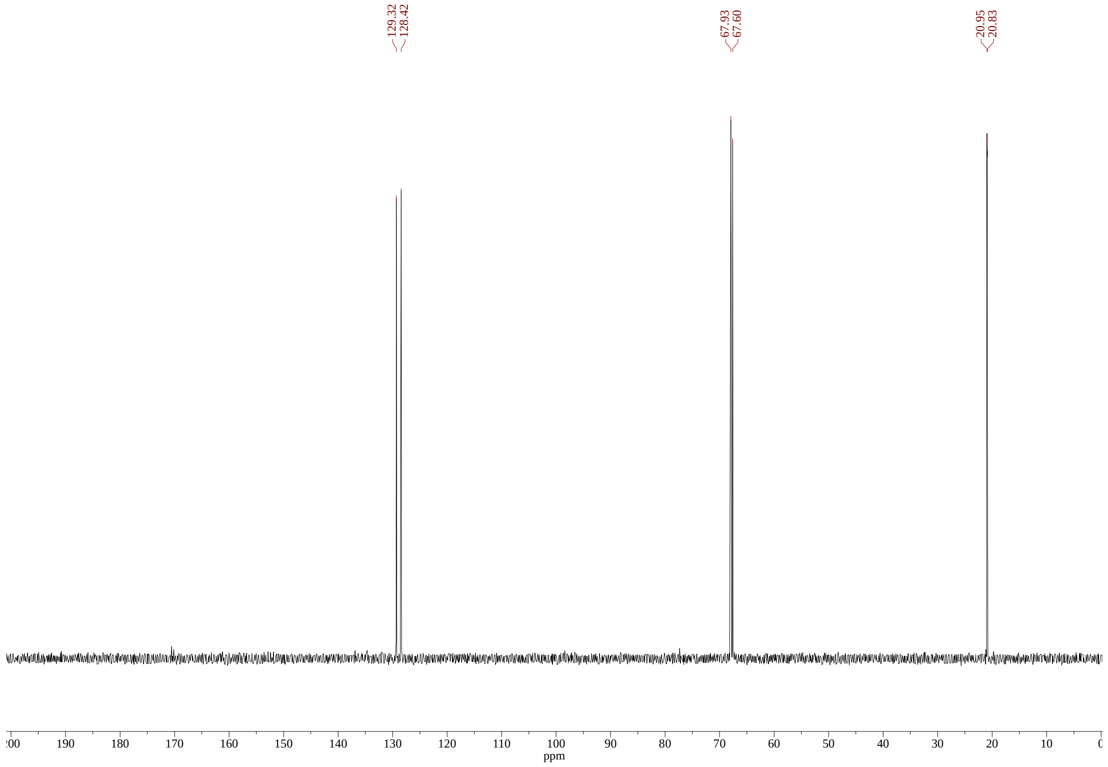

3a^{syn} - DEPTQ ($CDCl_3$)

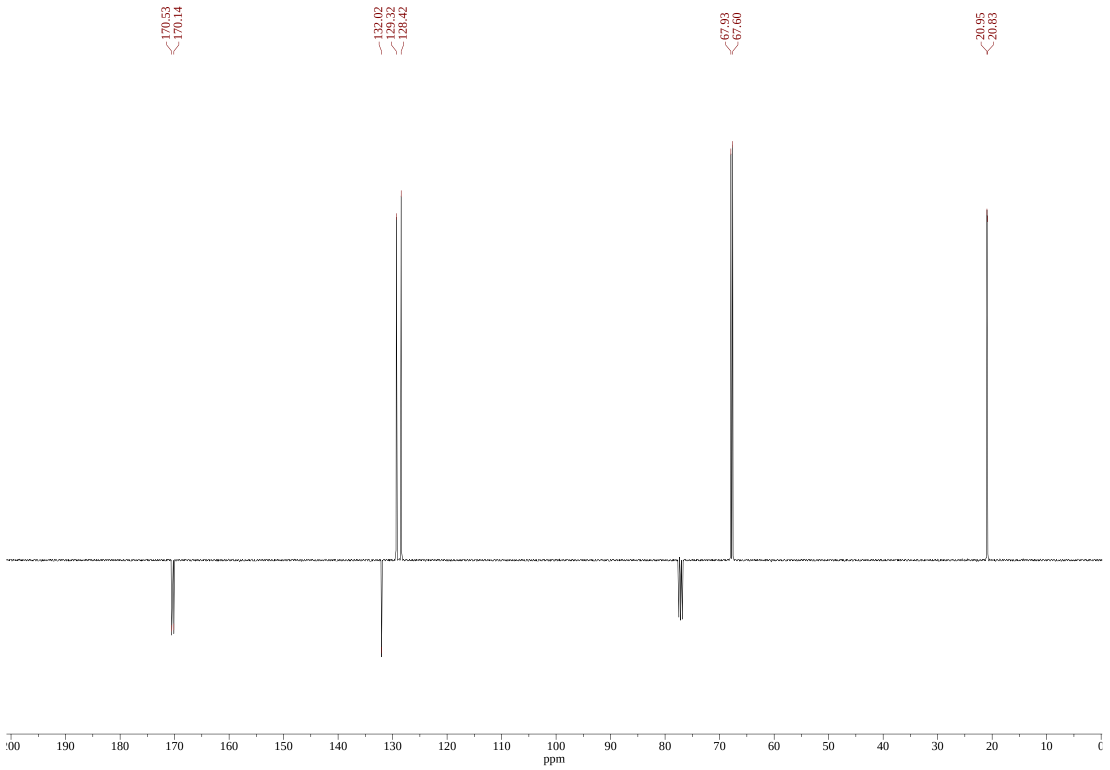

3a^{syn} - ^{1}H-^{1}H COSY ($CDCl_3$)

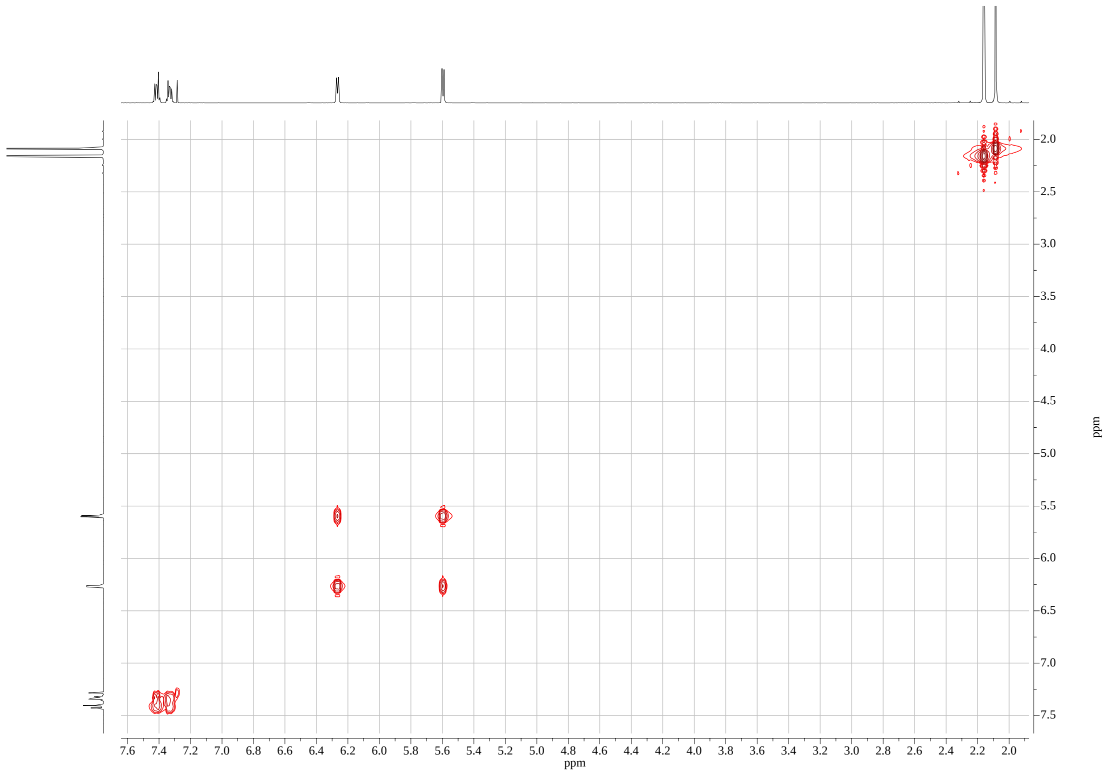

3a^{syn} - ^{1}H-^{13}C HSQCED (CDCl$_3$)

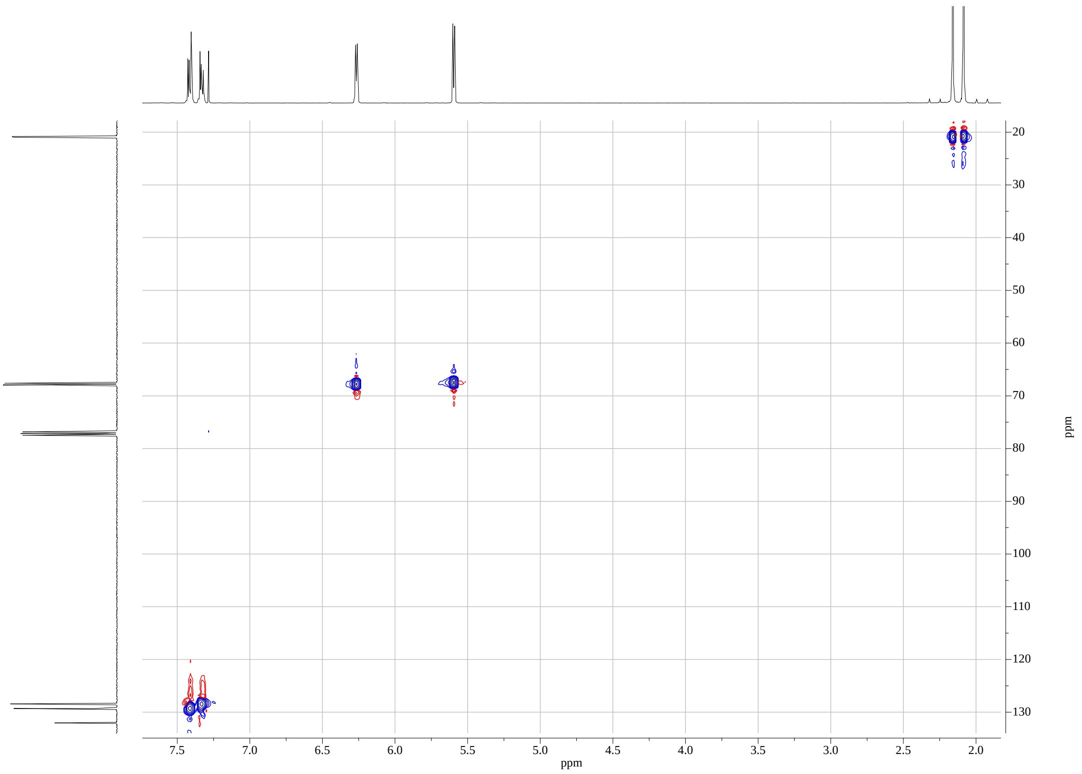

3a^{syn} - ^{1}H-^{13}C HMBC (CDCl$_3$)

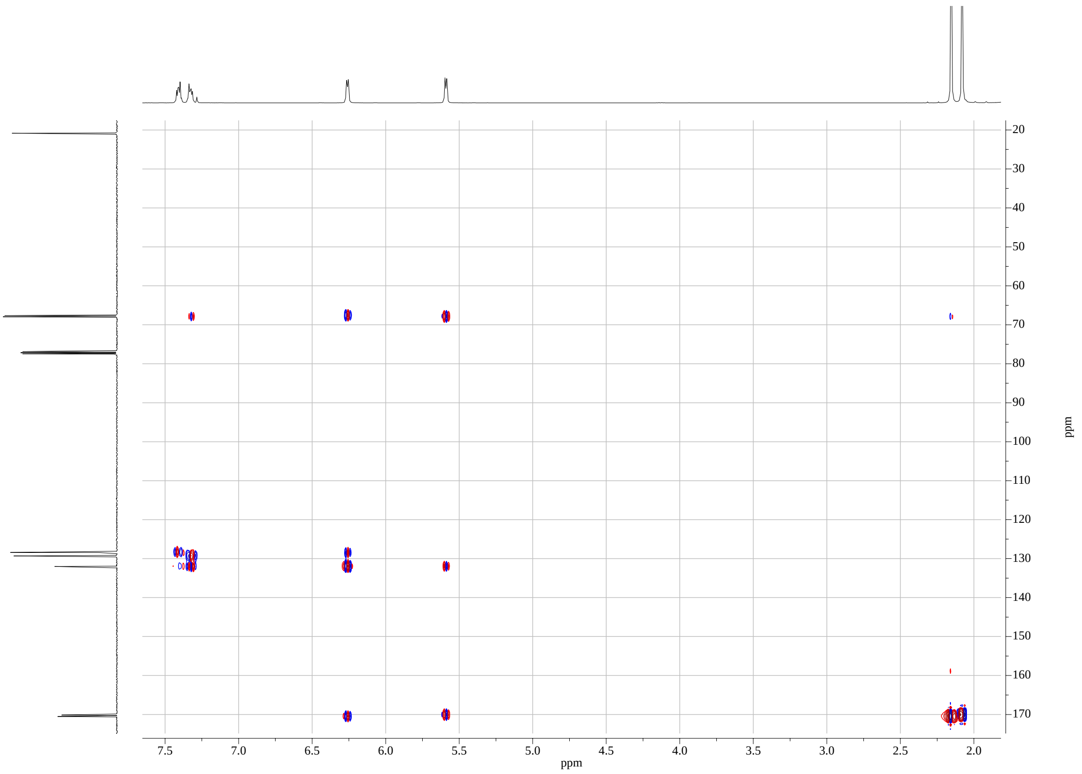

(1α,2α,3β,4β)-1,2,3,4-tetrahydronaphthalene-1,2,3,4-tetraol (3a^anti)

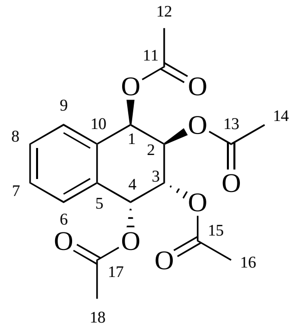

3a^anti - ^{1}H NMR (400 MHz, $CDCl_3$)

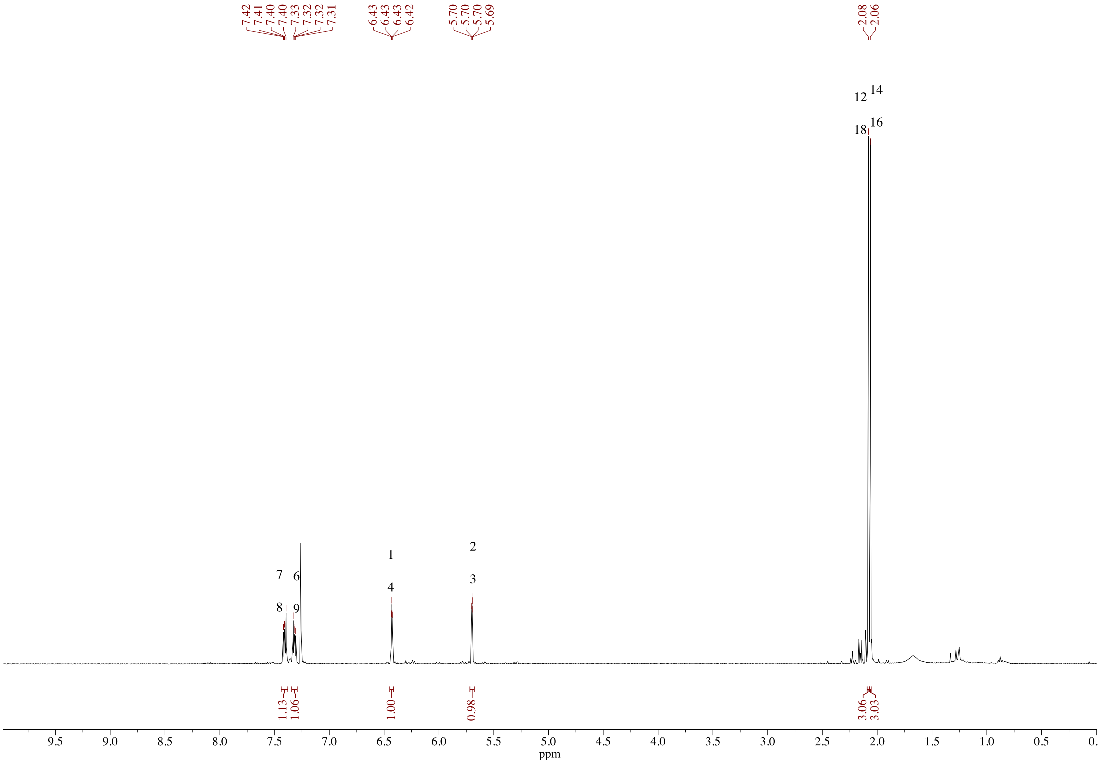

3a[anti] - ^{13}C NMR (100 MHz, $CDCl_3$)

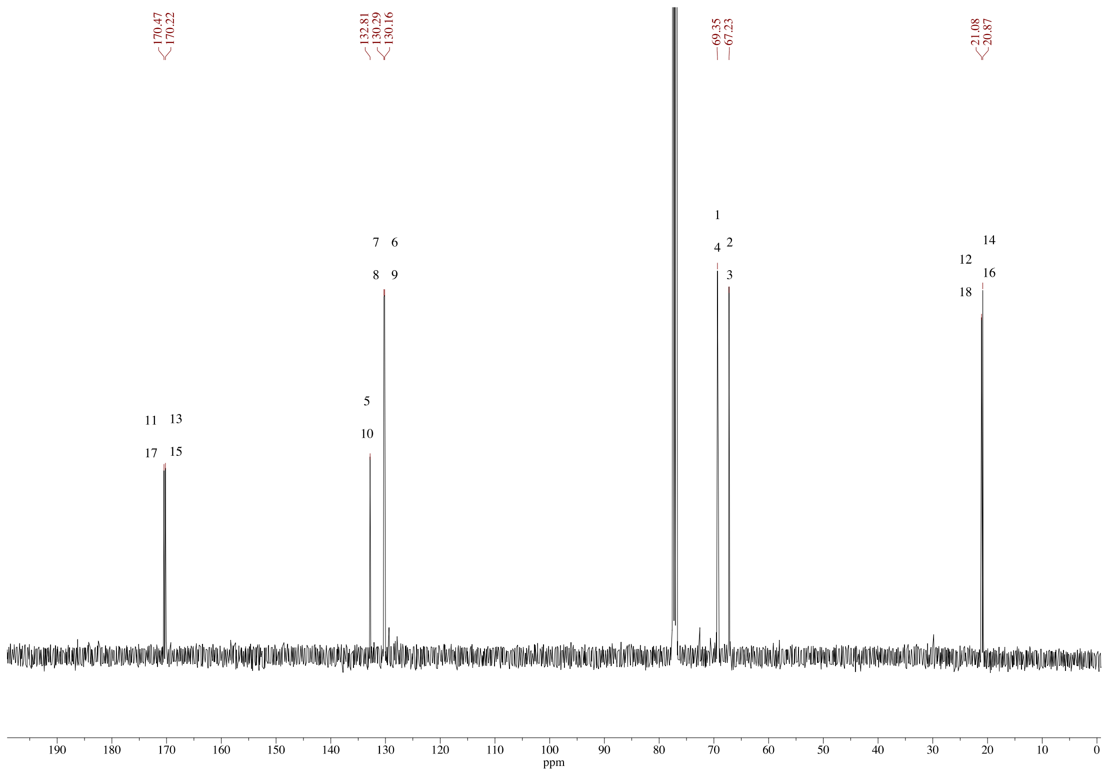

3a[anti] - DEPT ($CDCl_3$)

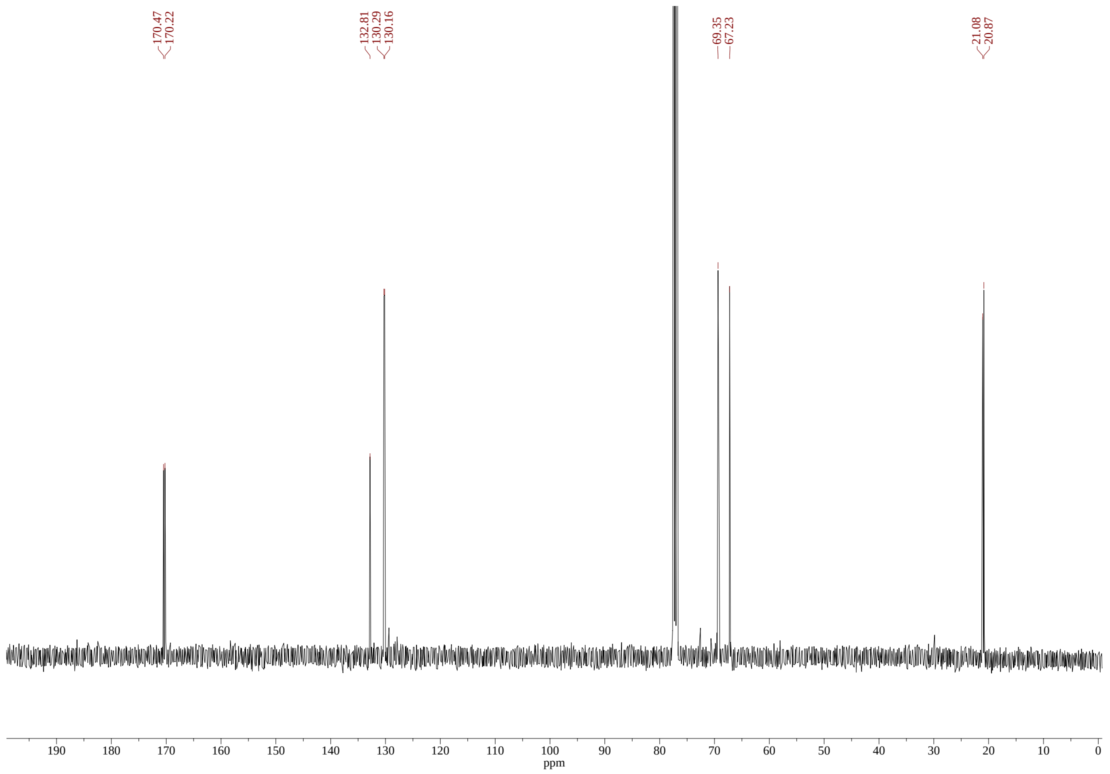

3a[anti] - DEPTQ ($CDCl_3$)

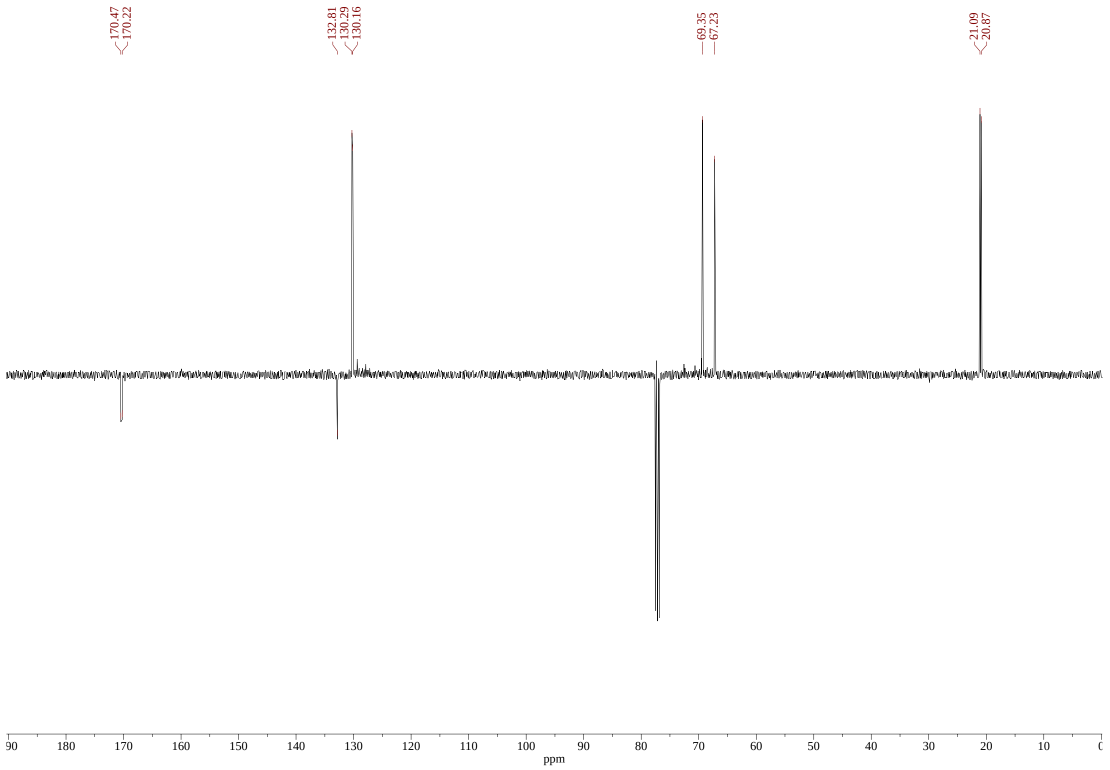

3a[anti] - ^1H-^1H COSY ($CDCl_3$)

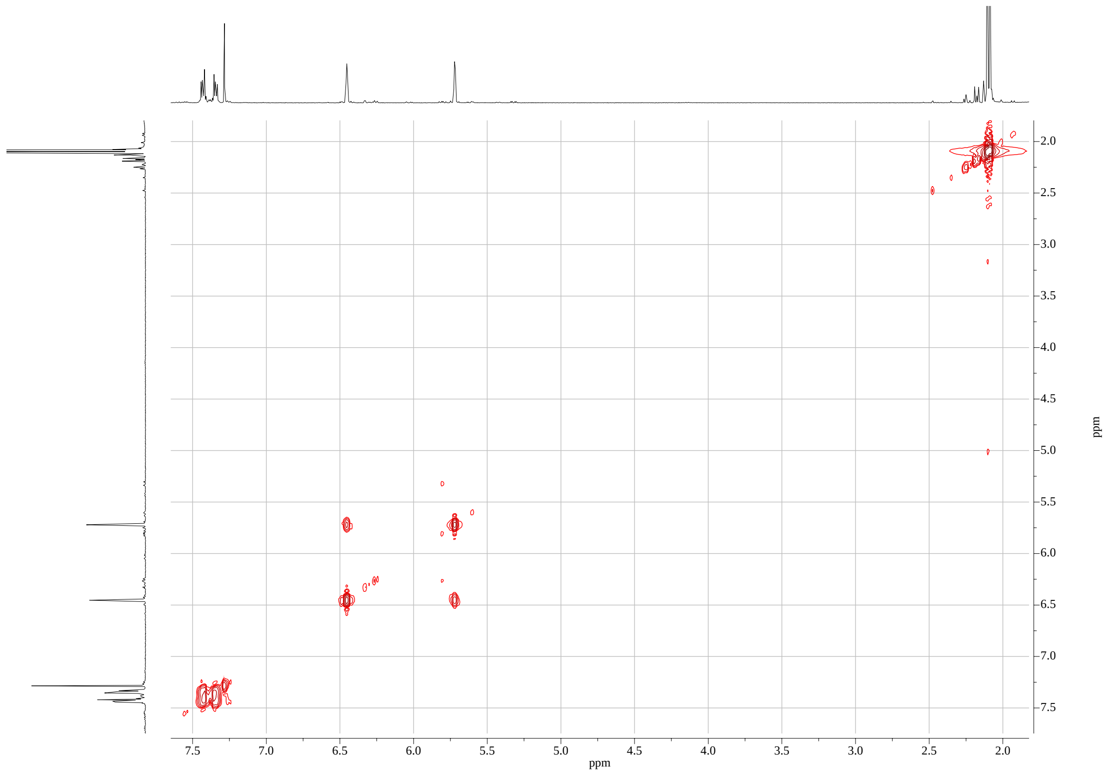

3a^anti^ - ^{1}H-^{13}C HSQCED ($CDCl_3$)

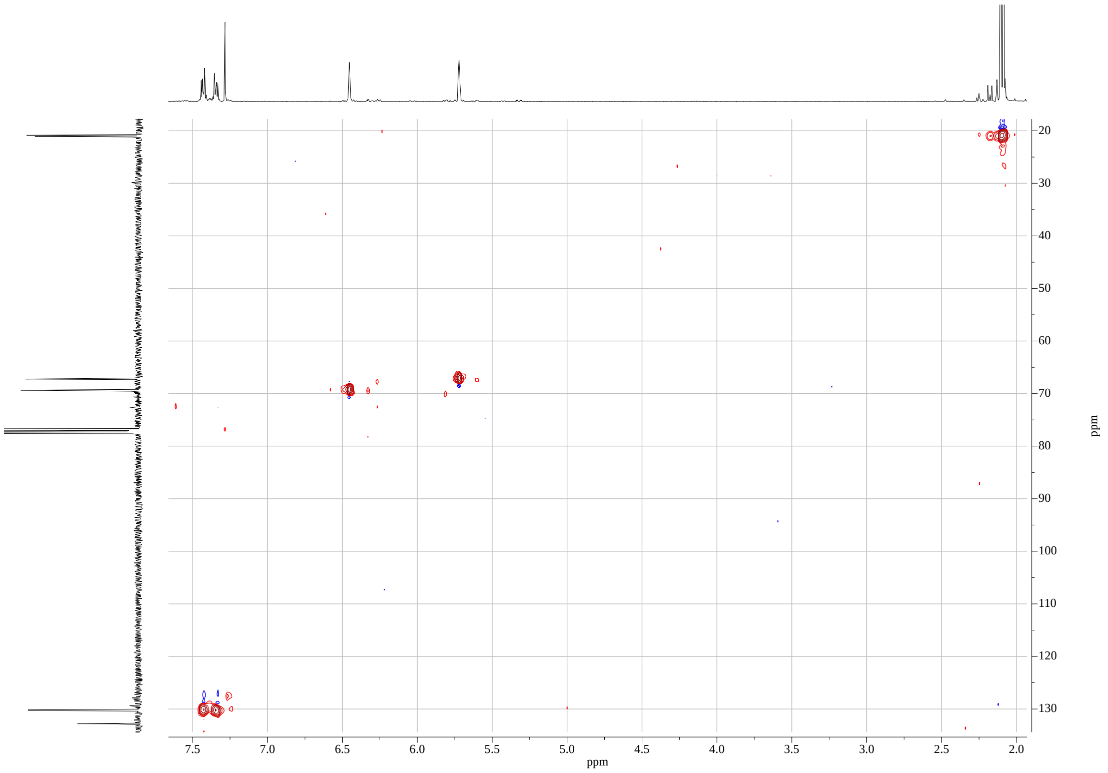

3a^anti^ - ^{1}H-^{13}C HMBC ($CDCl_3$)

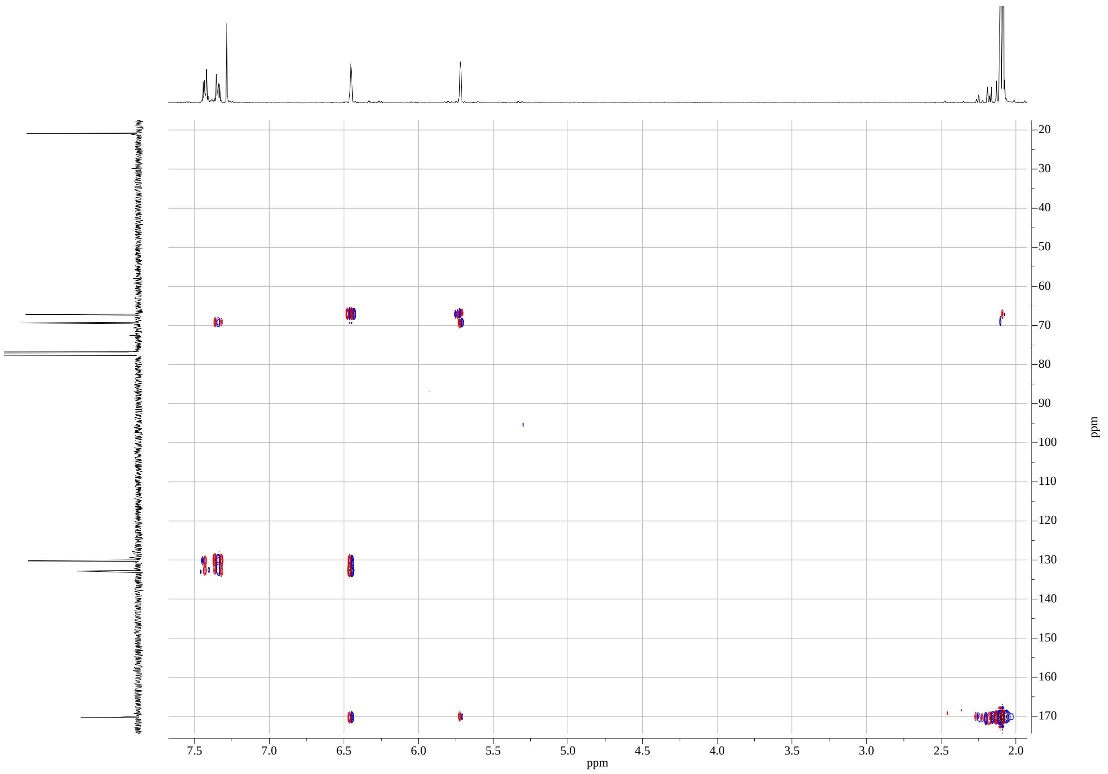

(1α,2α,3α,4α)-6,7-dimethyl-1,2,3,4-tetrahydronaphthalene-1,2,3,4-tetraol (3b^{syn})

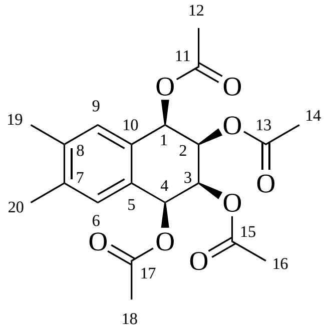

3b^{syn} - ^{1}H NMR (400 MHz, $CDCl_3$)

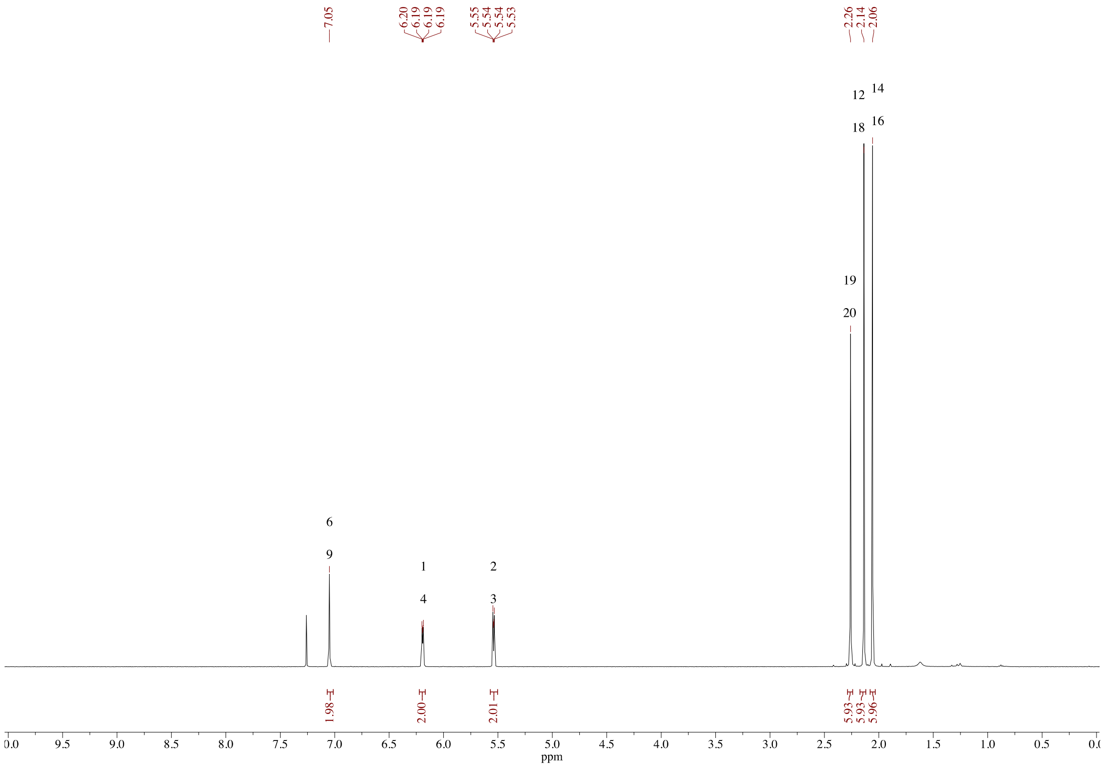

3b^{syn} - ^{13}C NMR (100 MHz, $CDCl_3$)

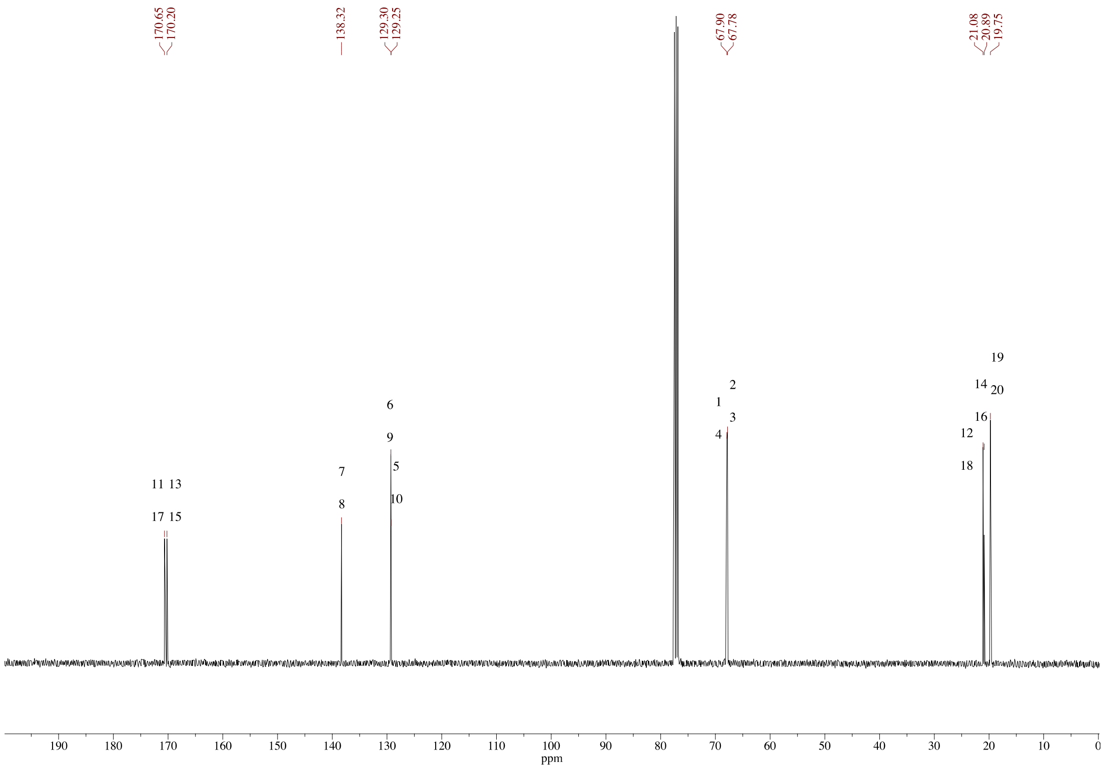

3b^{syn} - DEPT ($CDCl_3$)

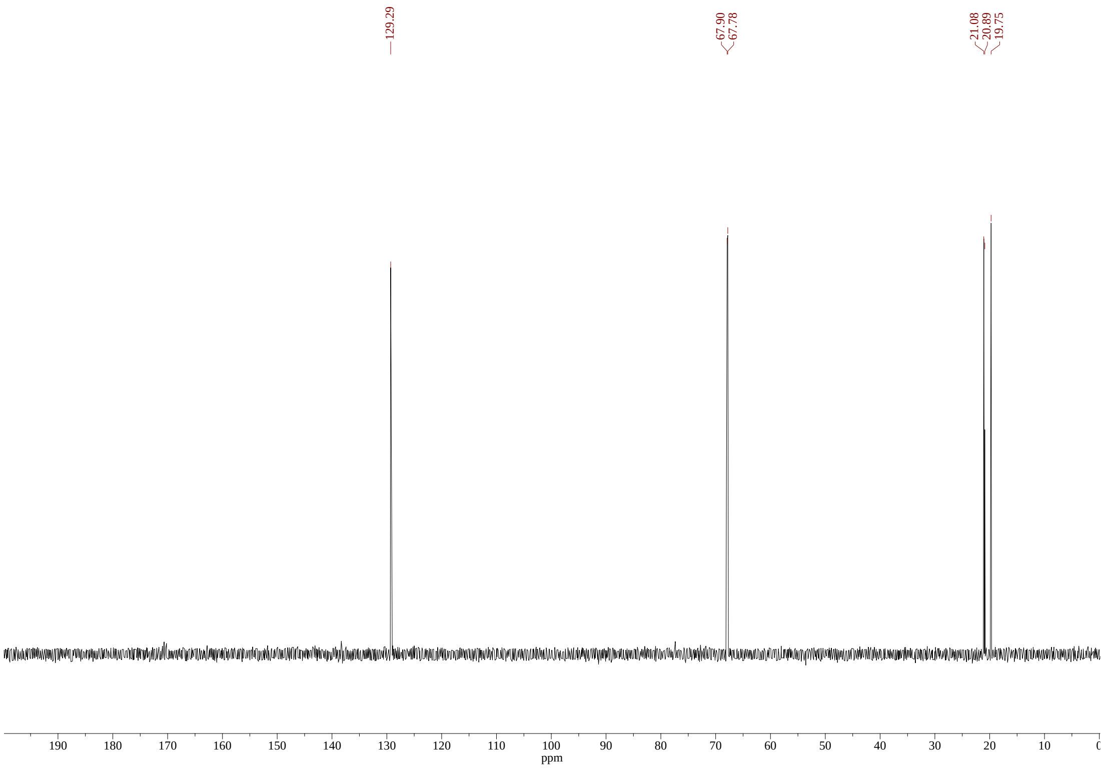

3b^{syn} - DEPTQ ($CDCl_3$)

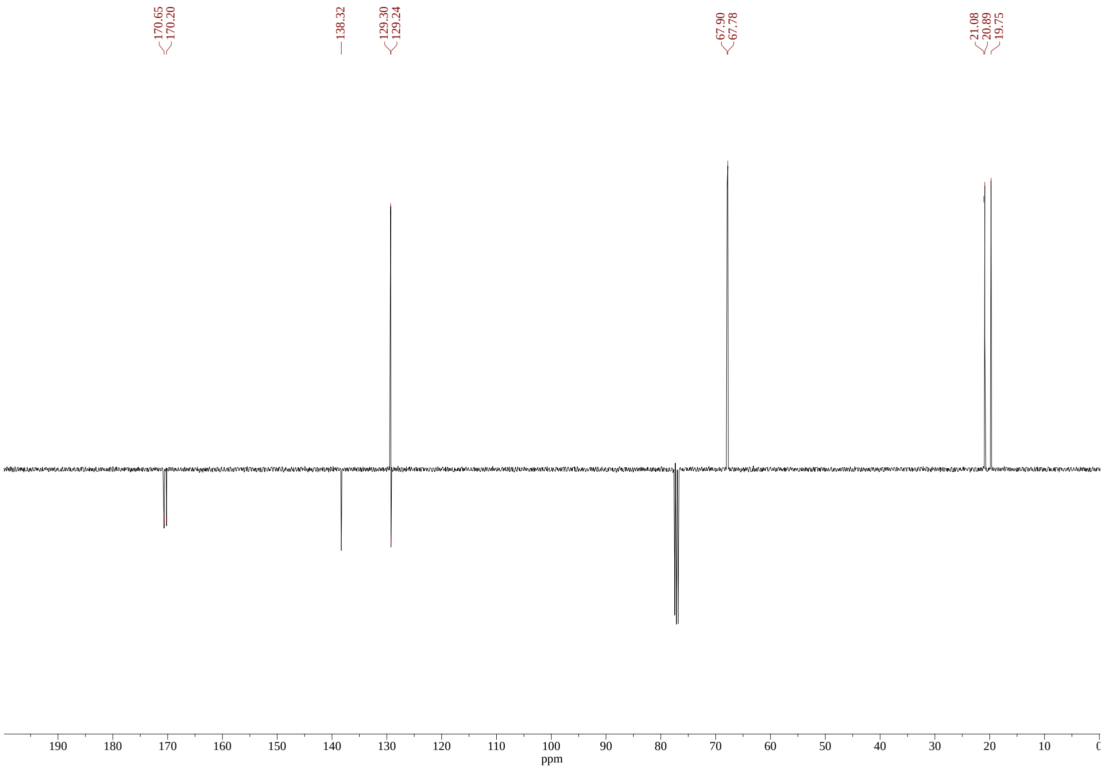

3b^{syn} - ^{1}H-^{1}H COSY ($CDCl_3$)

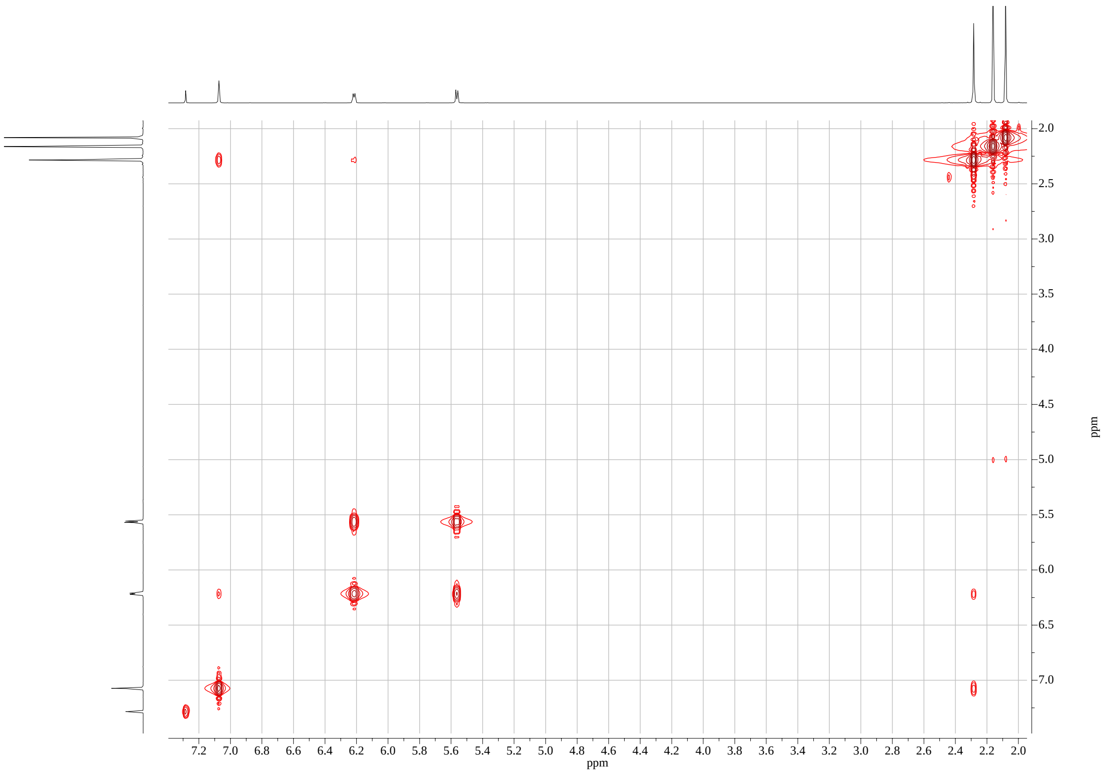

3b^{syn} - ^{1}H-^{13}C HSQCED ($CDCl_3$)

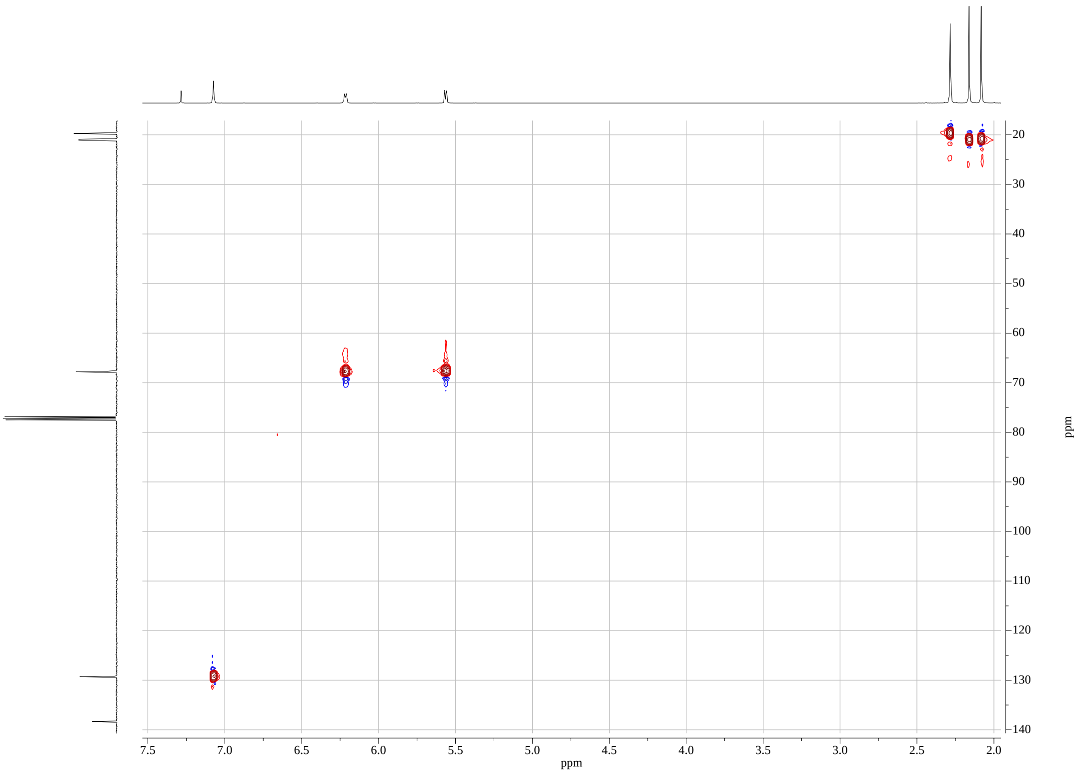

3b^{syn} - ^{1}H-^{13}C HMBC ($CDCl_3$)

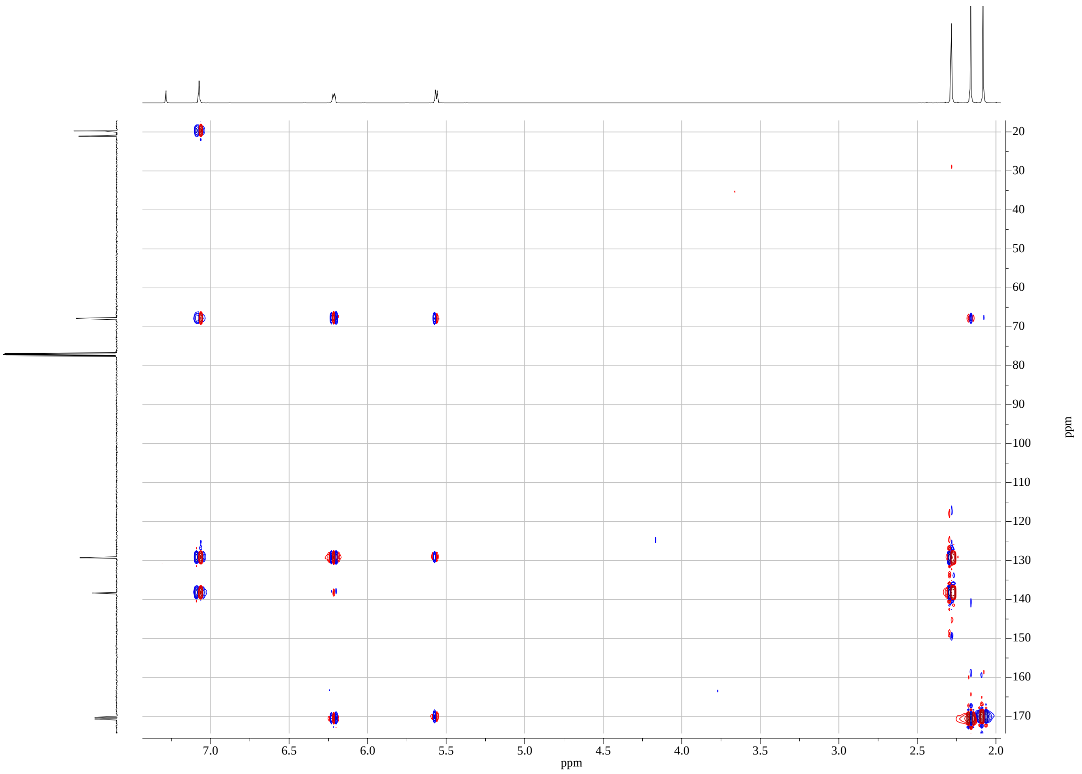

(1α,2α,3β,4β)-6,7-dimethyl-1,2,3,4-tetrahydronaphthalene-1,2,3,4-tetraol (3b^{anti})

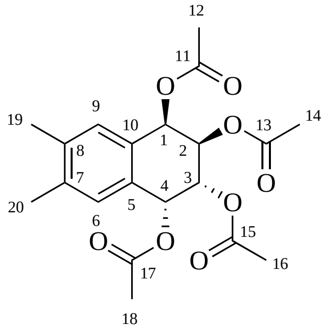

3b^{anti} - ^{1}H NMR (400 MHz, $CDCl_3$)

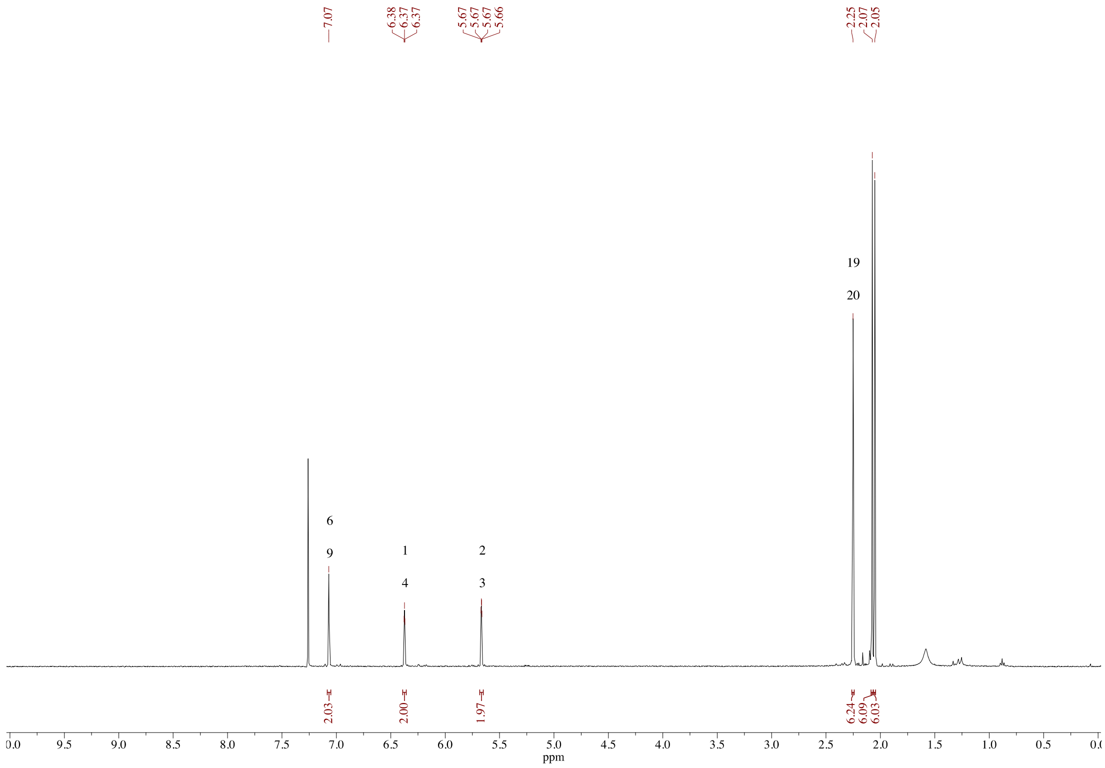

3b^{anti} - ^{13}C NMR (100 MHz, $CDCl_3$)

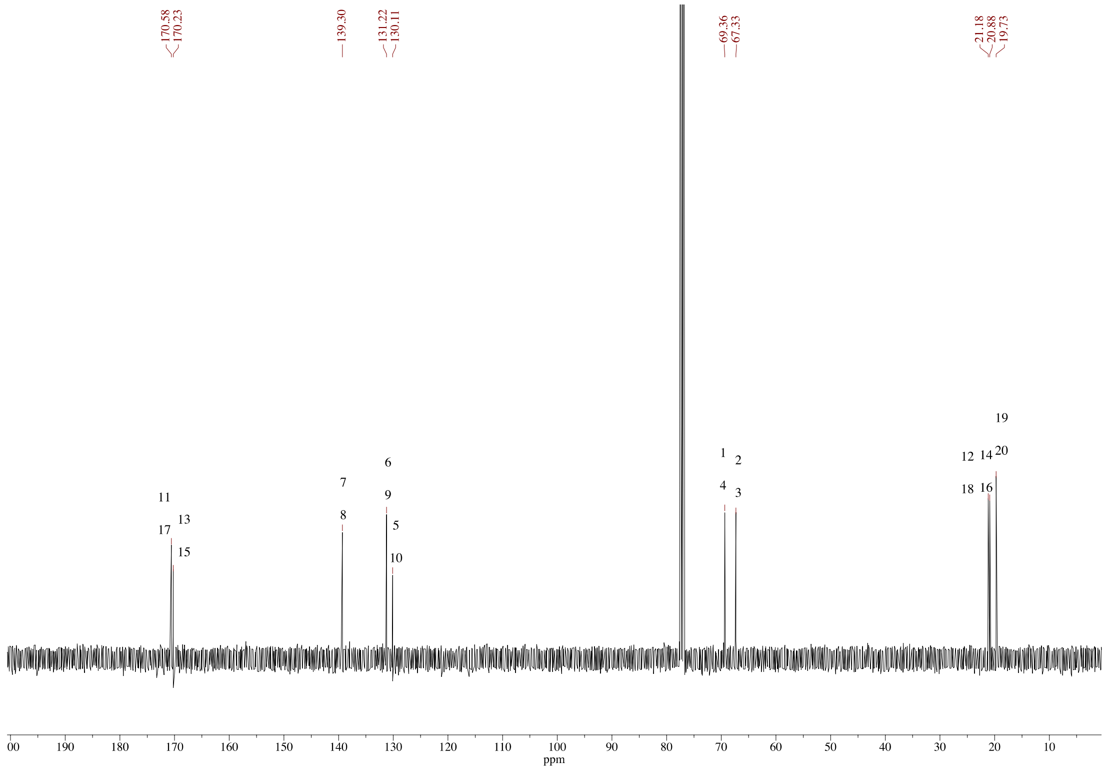

3b^{anti} - DEPT ($CDCl_3$)

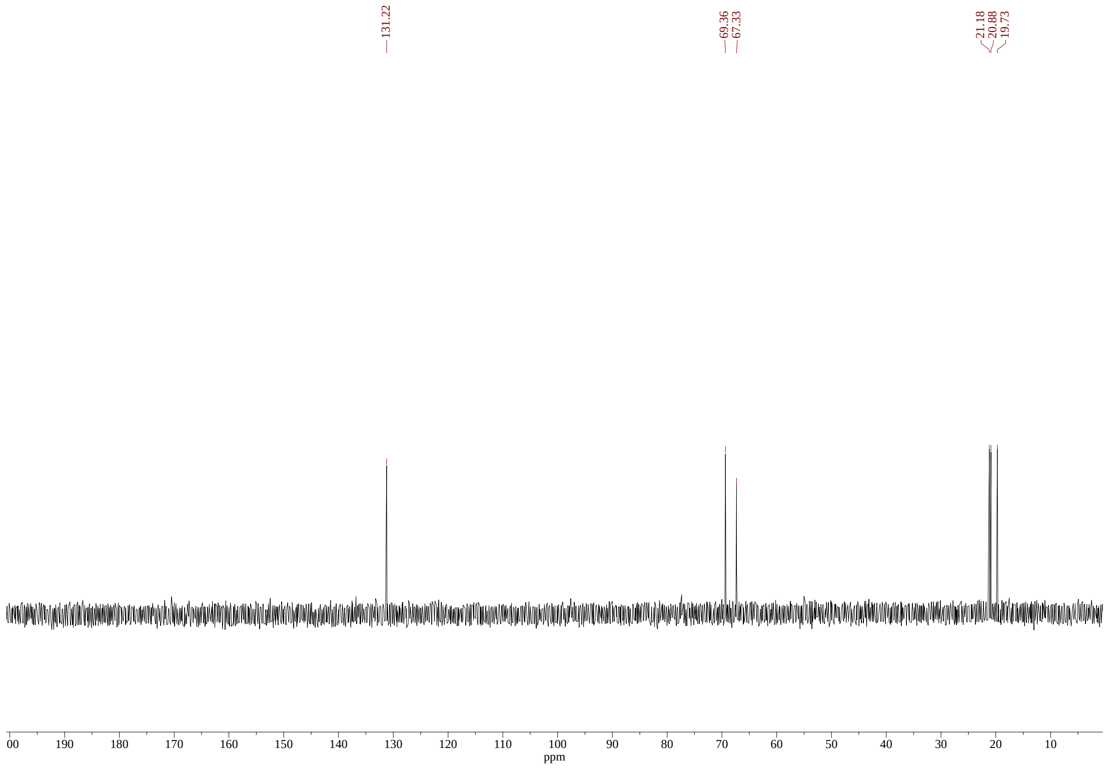

3b[anti] - DEPTQ ($CDCl_3$)

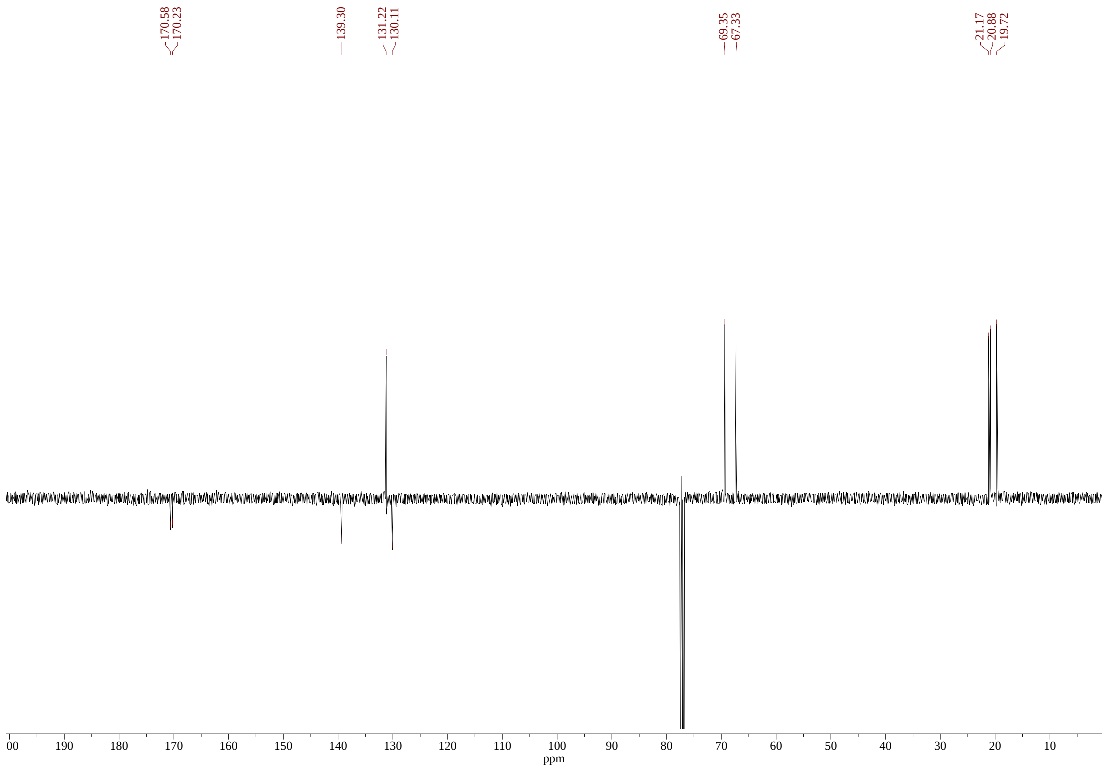

3b[anti] - ^1H-^1H COSY ($CDCl_3$)

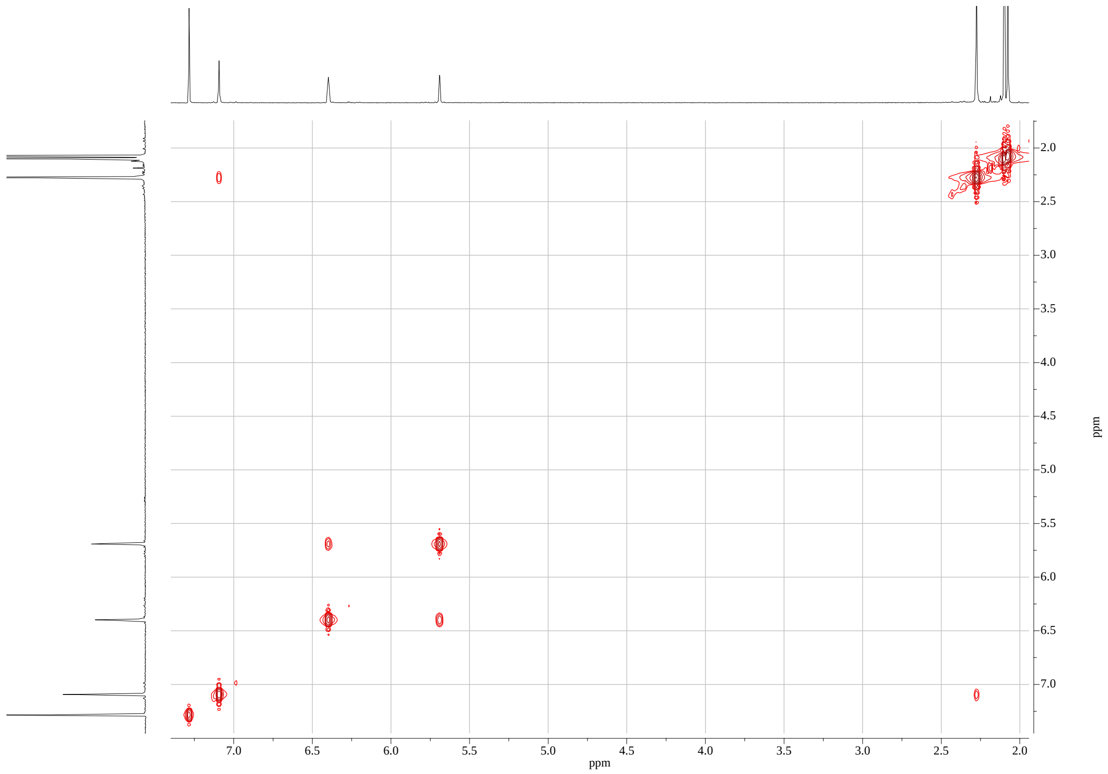

3b^anti^ - ^{1}H-^{13}C HSQCED ($CDCl_3$)

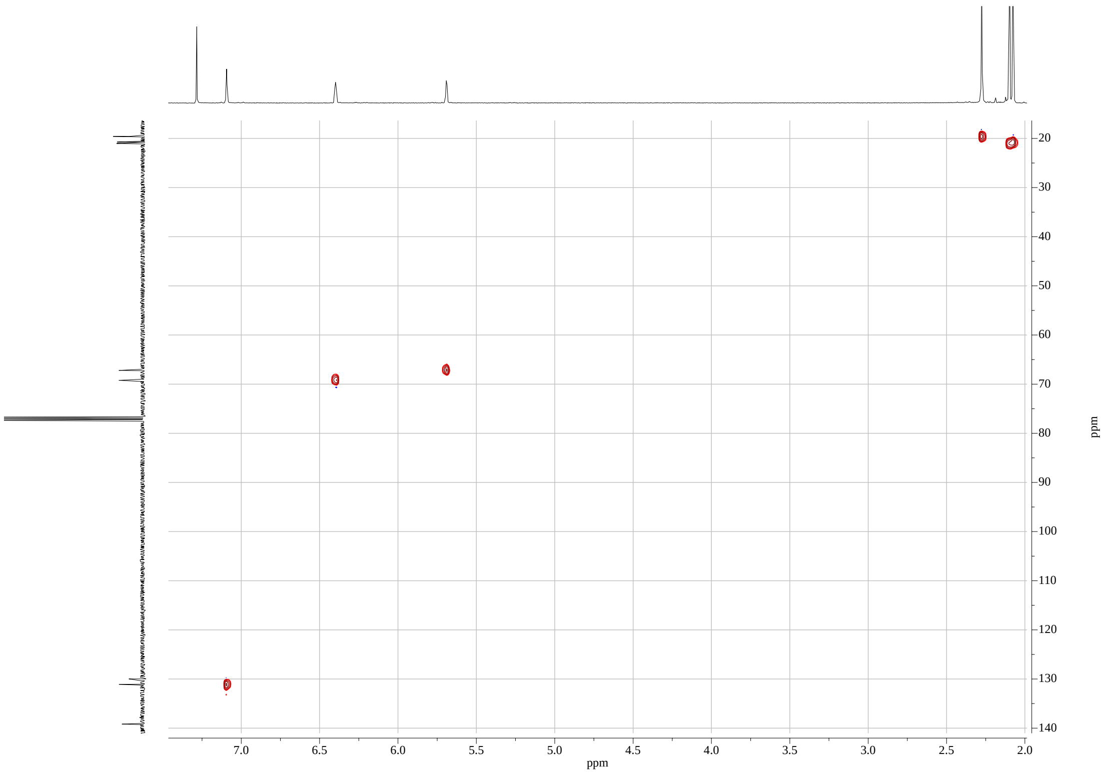

3b^anti^ - ^{1}H-^{13}C HMBC ($CDCl_3$)

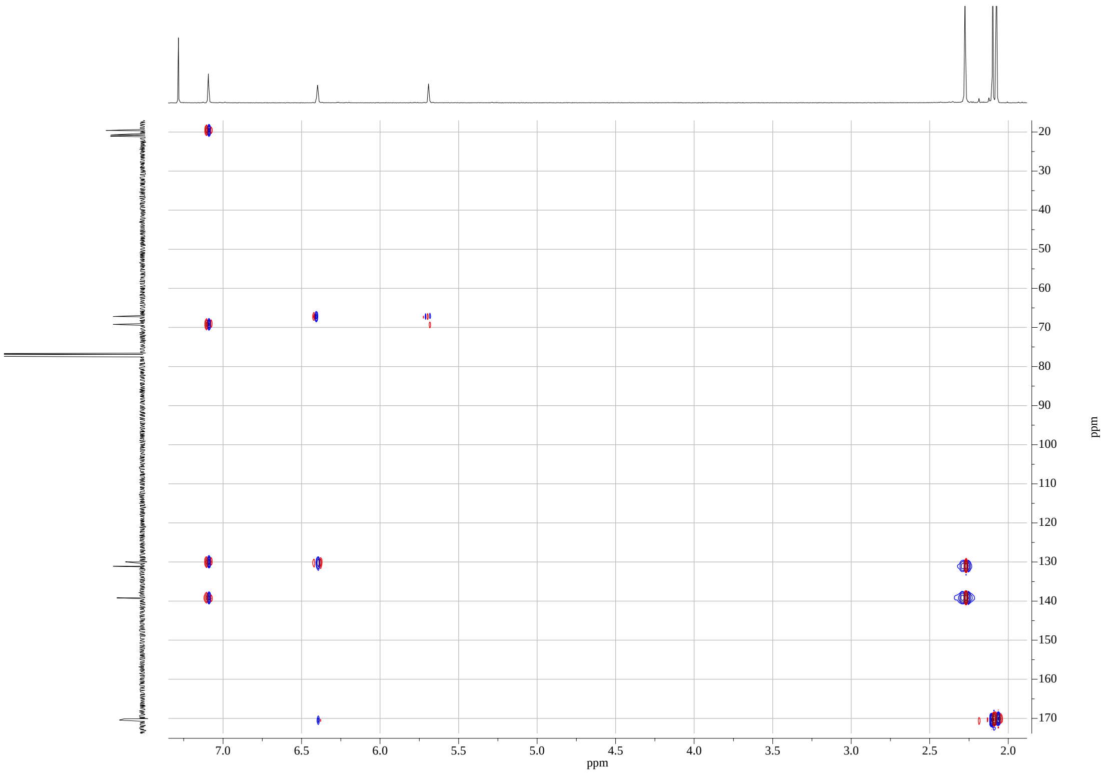

(1α,2α,3α,4α)-3-hydroxy-3,7-dimethyl-1,2,3,4-tetrahydronaphthalene-1,2,4-triyl triacetate (3c^{syn})

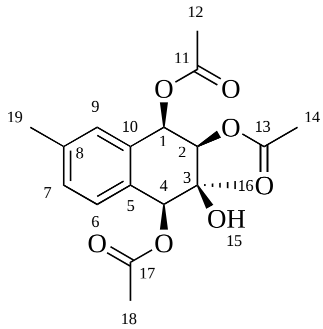

3c^{syn} - ^{1}H NMR (400 MHz, $CDCl_3$)

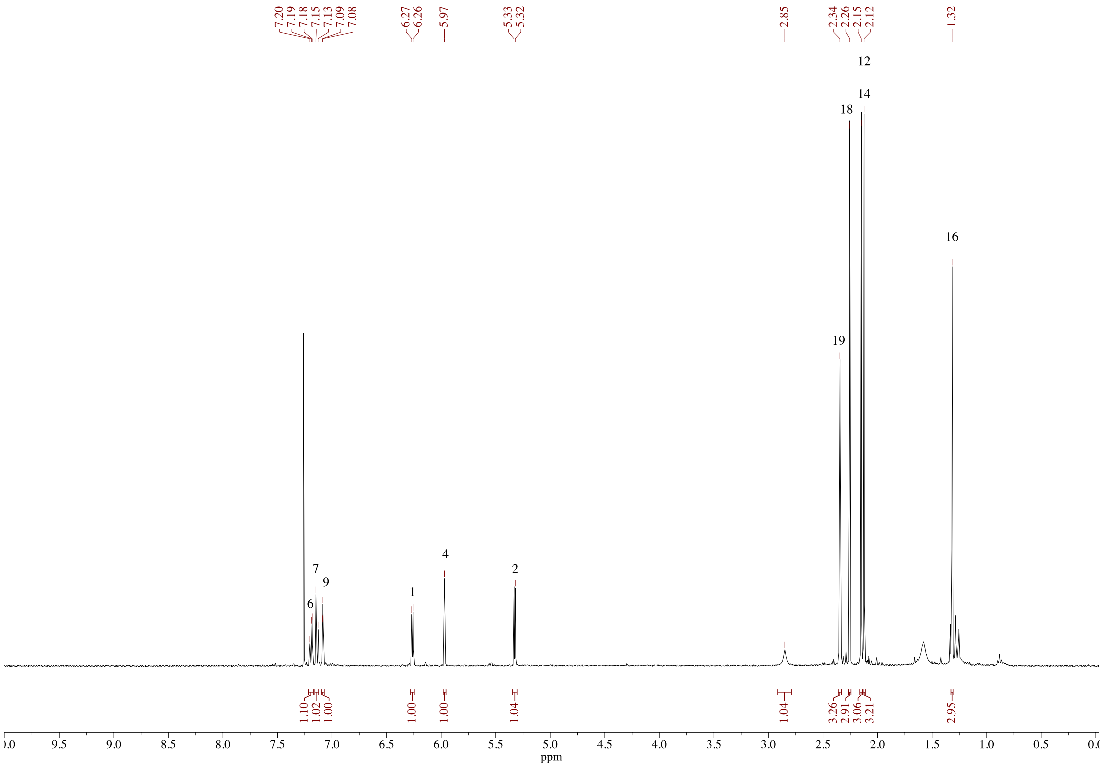

3c^syn^ - ^{13}C NMR (100 MHz, $CDCl_3$)

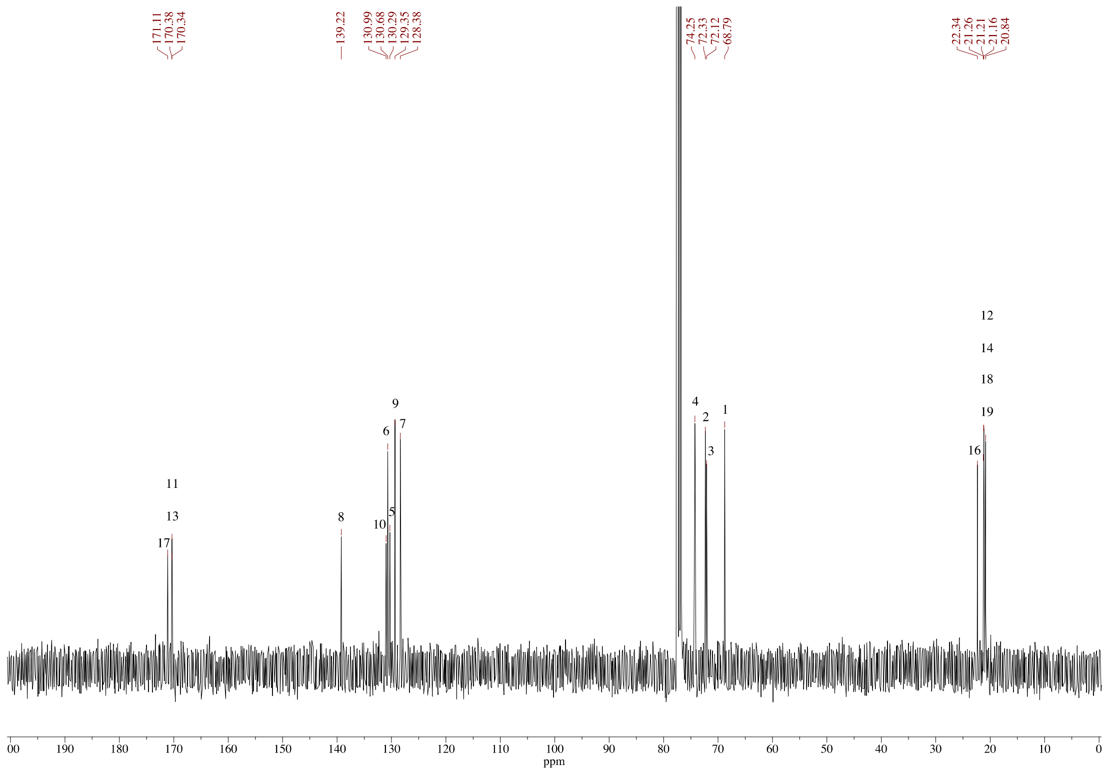

3c^syn^ - DEPT ($CDCl_3$)

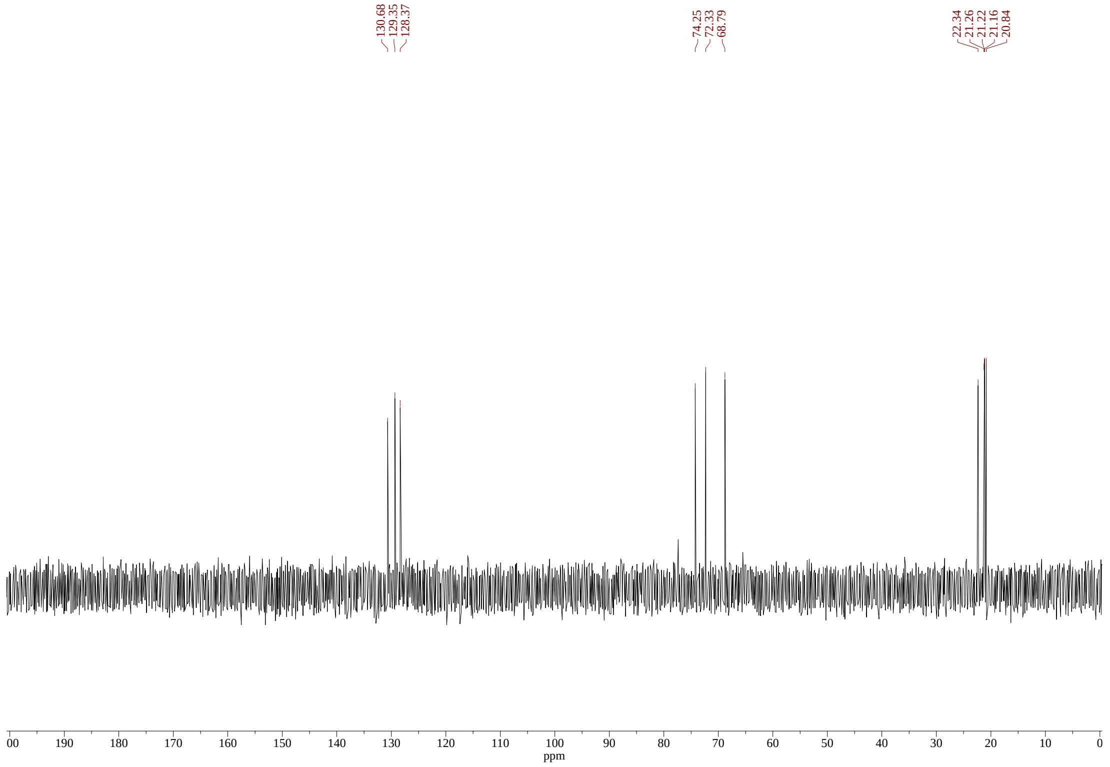

3c^syn - DEPTQ ($CDCl_3$)

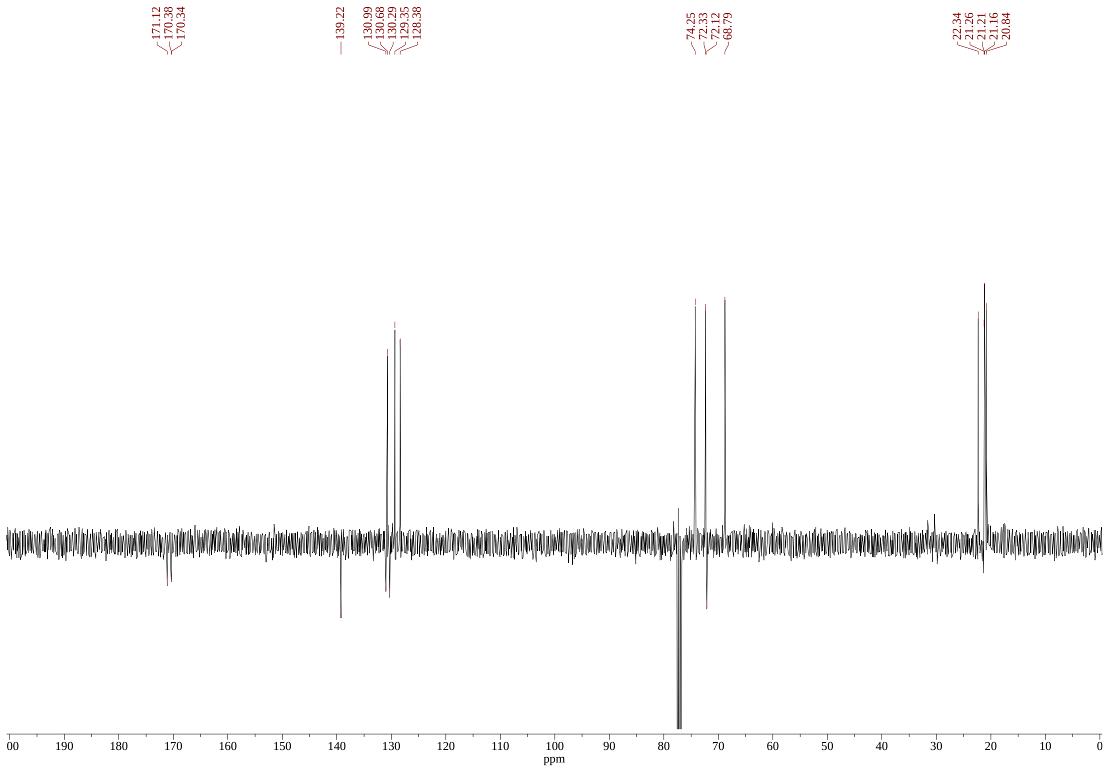

3c^syn - ^1H-^1H COSY ($CDCl_3$)

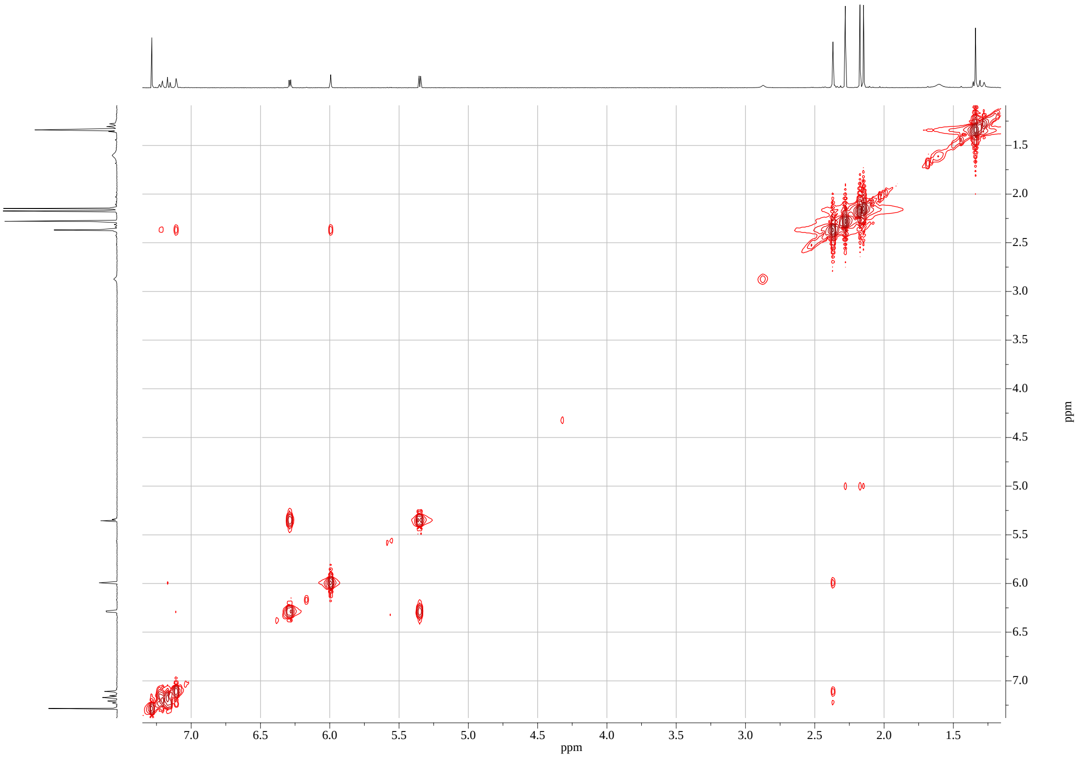

3c^{syn} - ^{1}H-^{13}C HSQCED ($CDCl_3$)

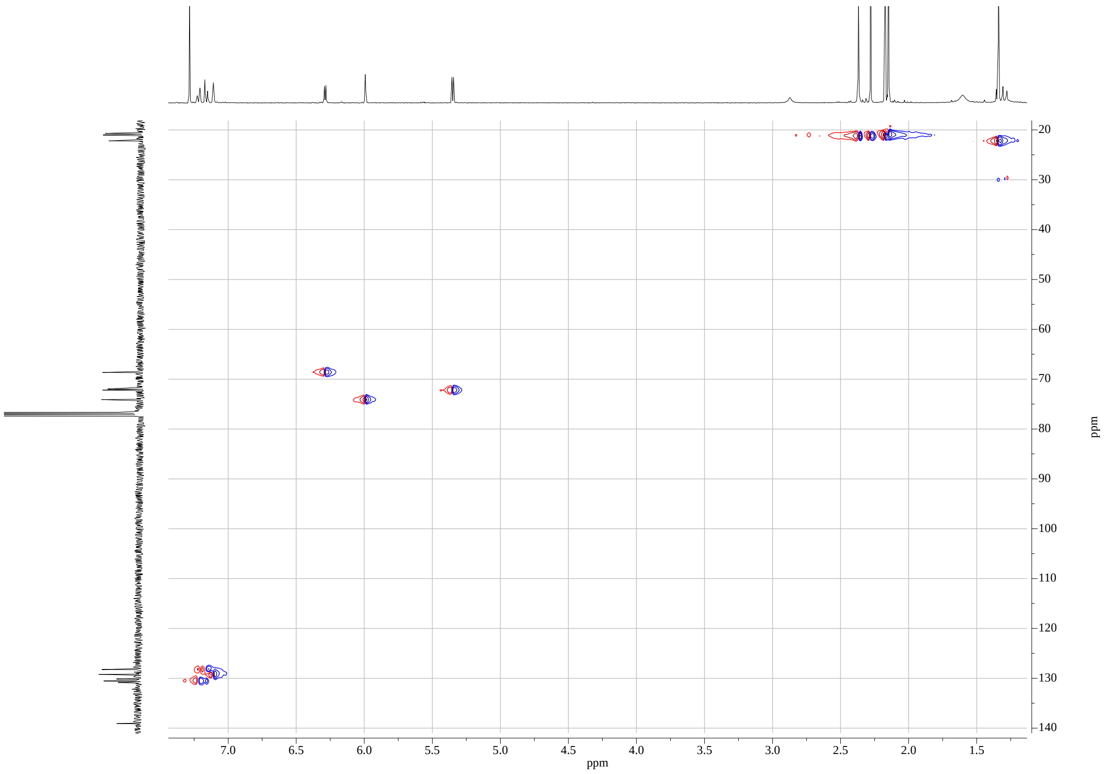

3c^{syn} - ^{1}H-^{13}C HMBC ($CDCl_3$)

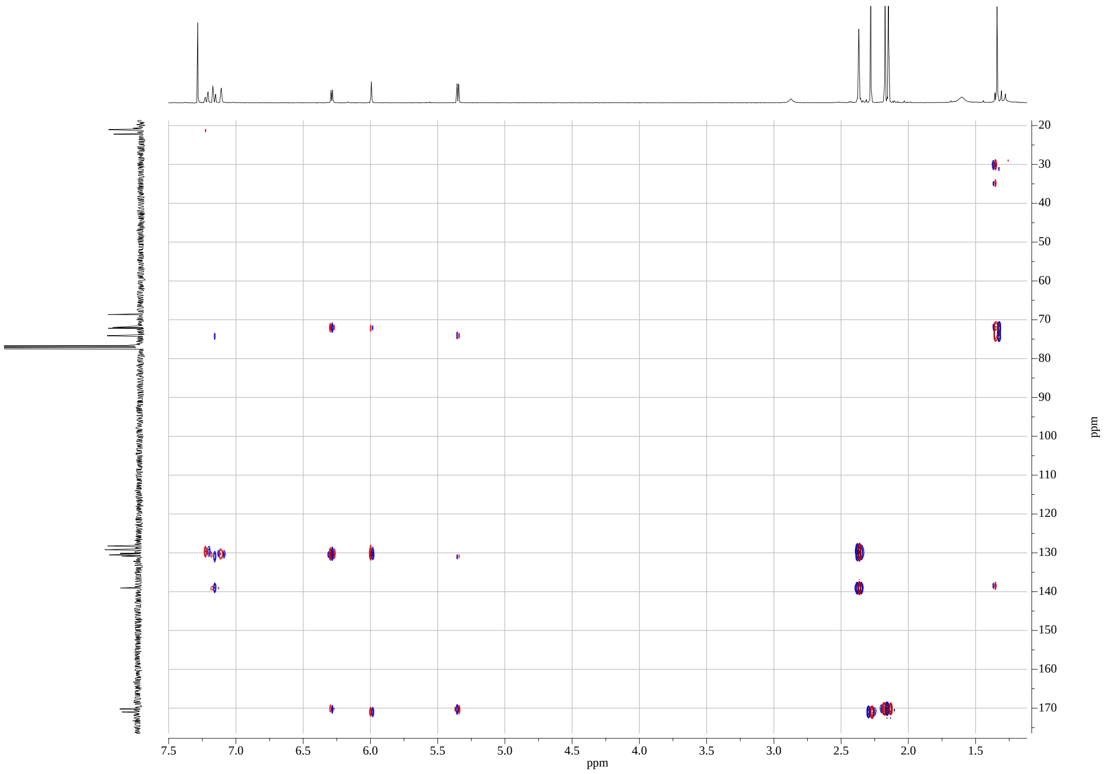

(1α,2α,3β,4β)-3-hydroxy-3,7-dimethyl-1,2,3,4-tetrahydronaphthalene-1,2,4-triyl triacetate (3c[anti])

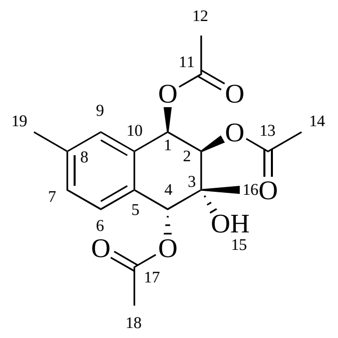

3c[anti] - ^{1}H NMR (400 MHz, $CDCl_3$)

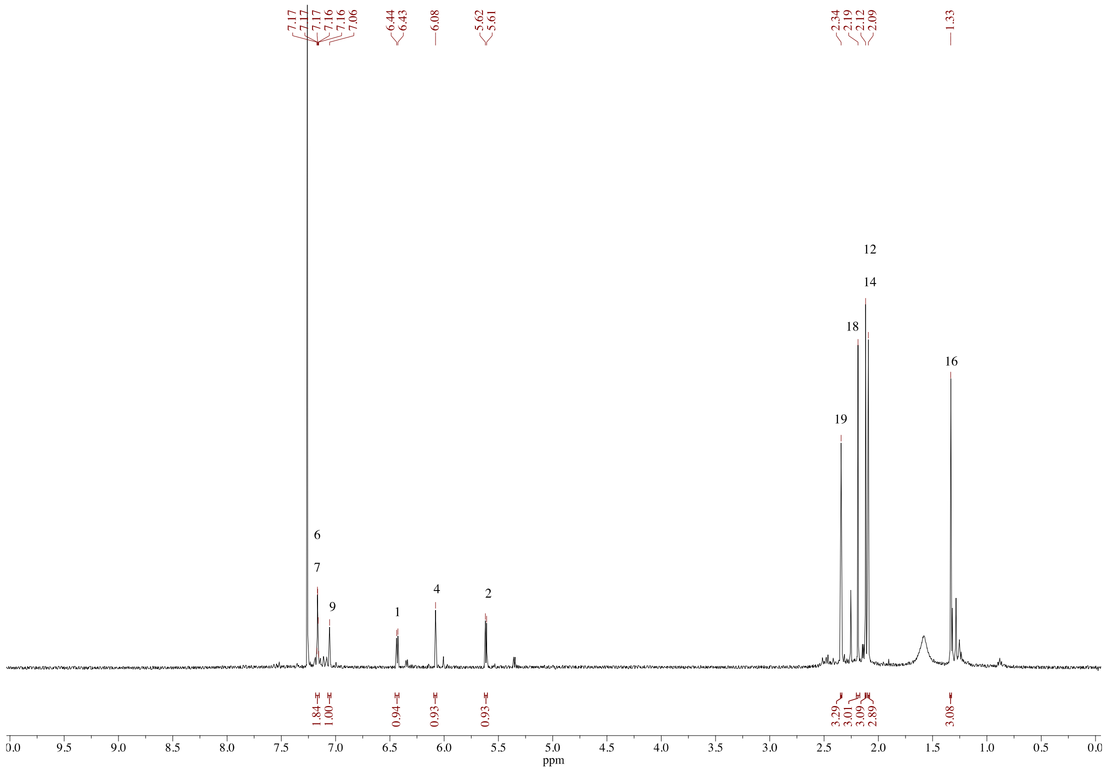

3c[anti] - ^{13}C NMR (100 MHz, $CDCl_3$)

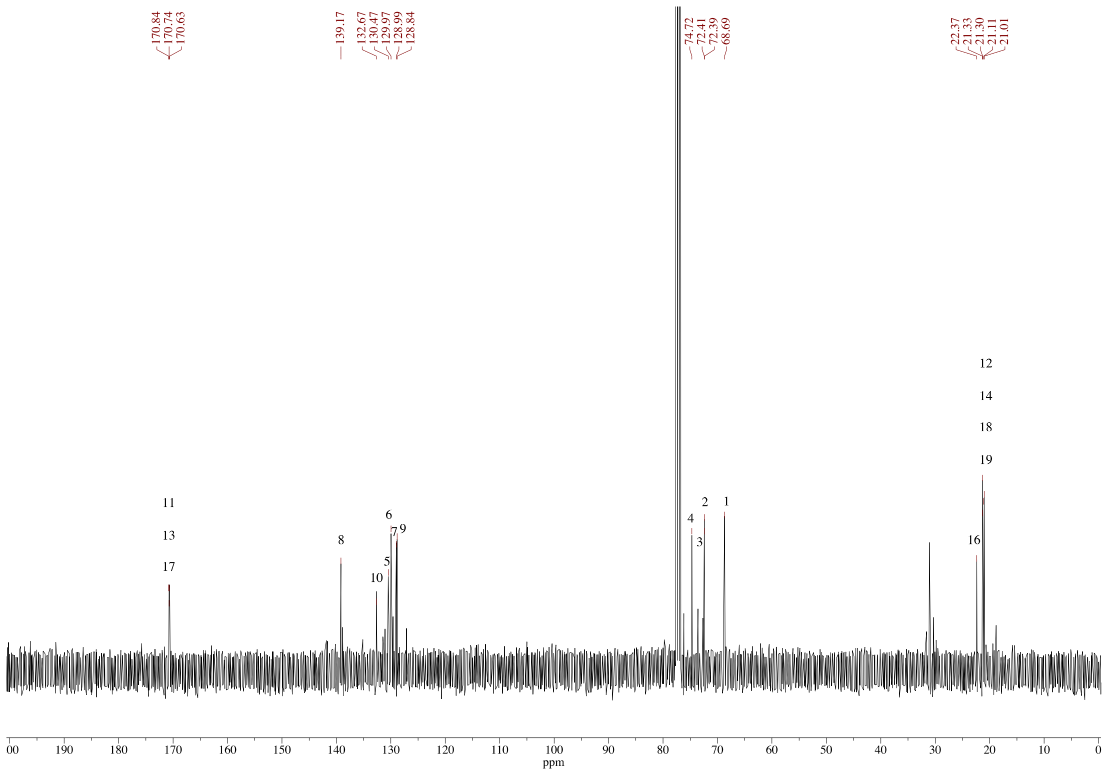

3c[anti] - DEPT ($CDCl_3$)

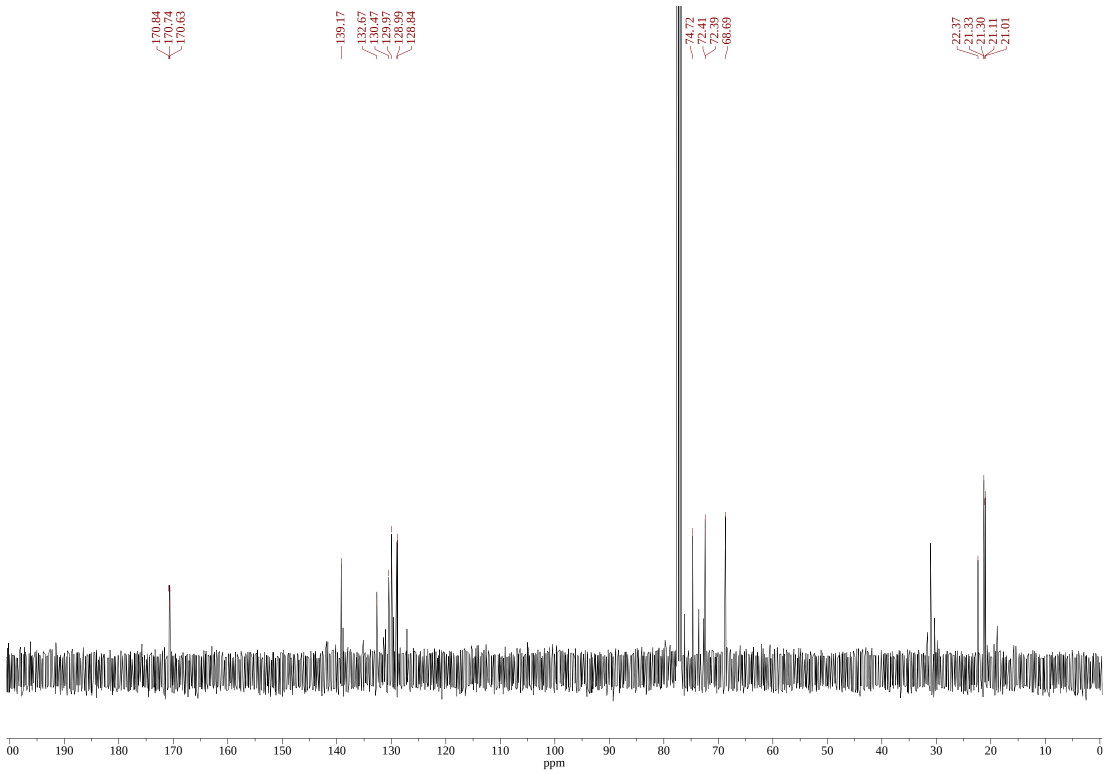

3c^anti^ - DEPTQ ($CDCl_3$)

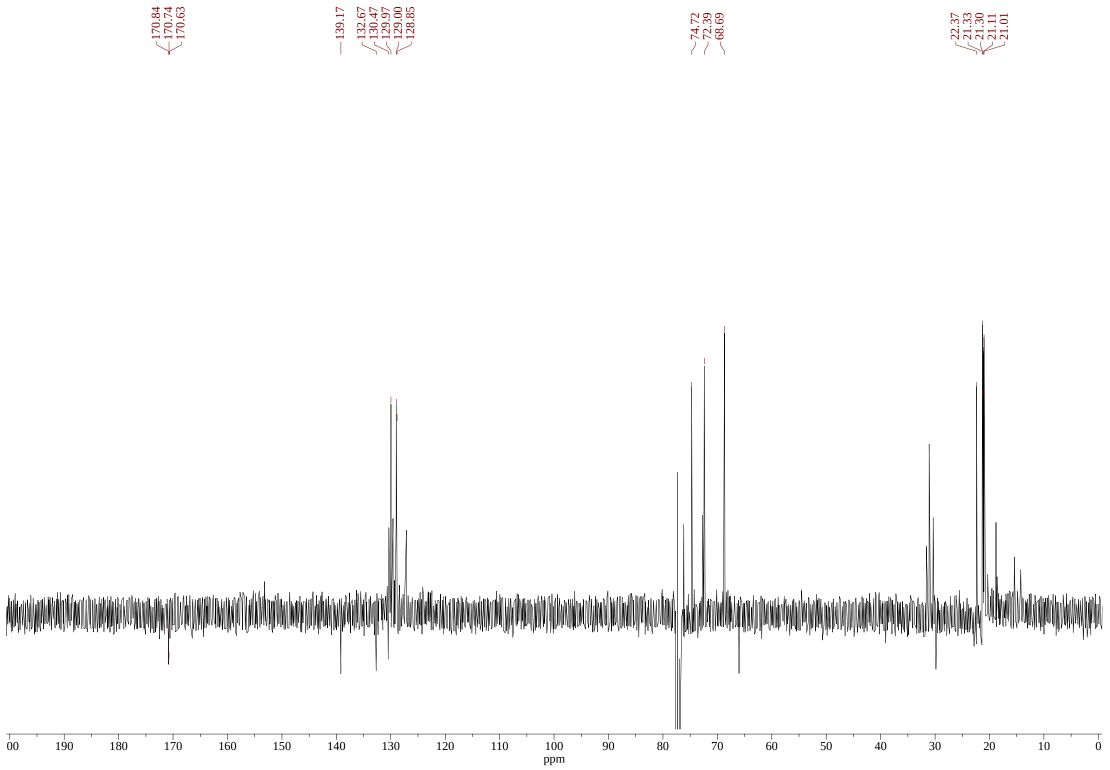

3c^anti^ - ^{1}H-^{1}H COSY ($CDCl_3$)

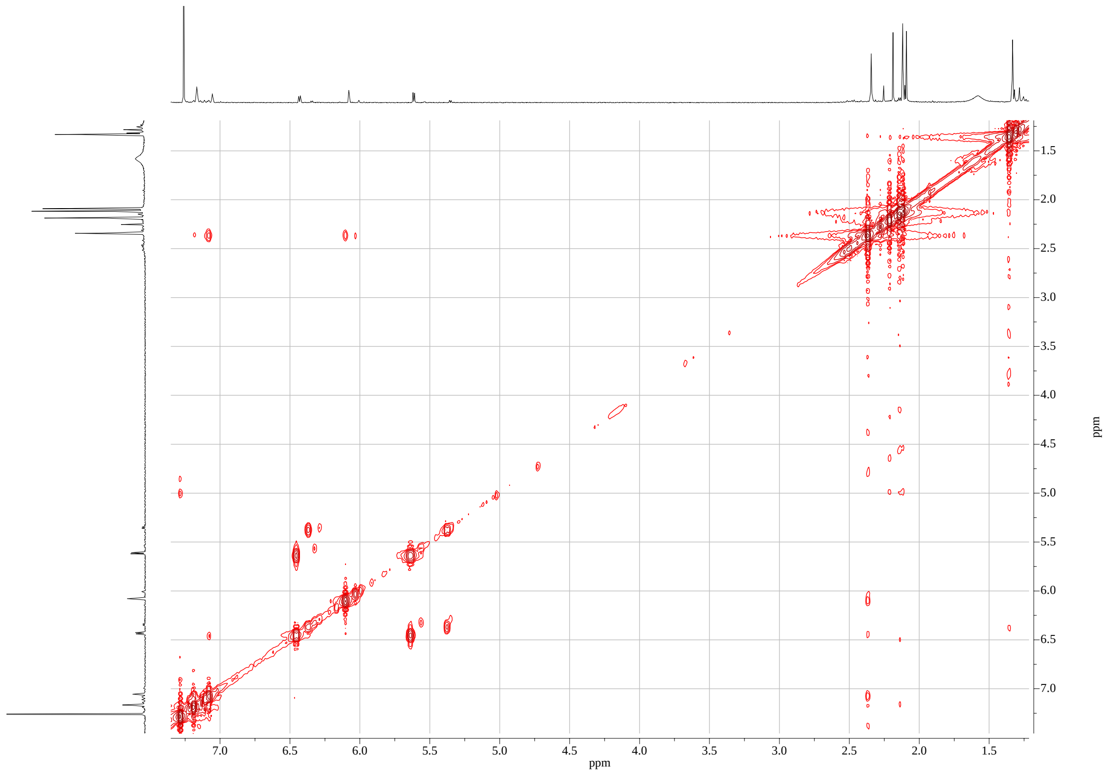

3c^{anti} - ^{1}H-^{13}C HSQCED ($CDCl_3$)

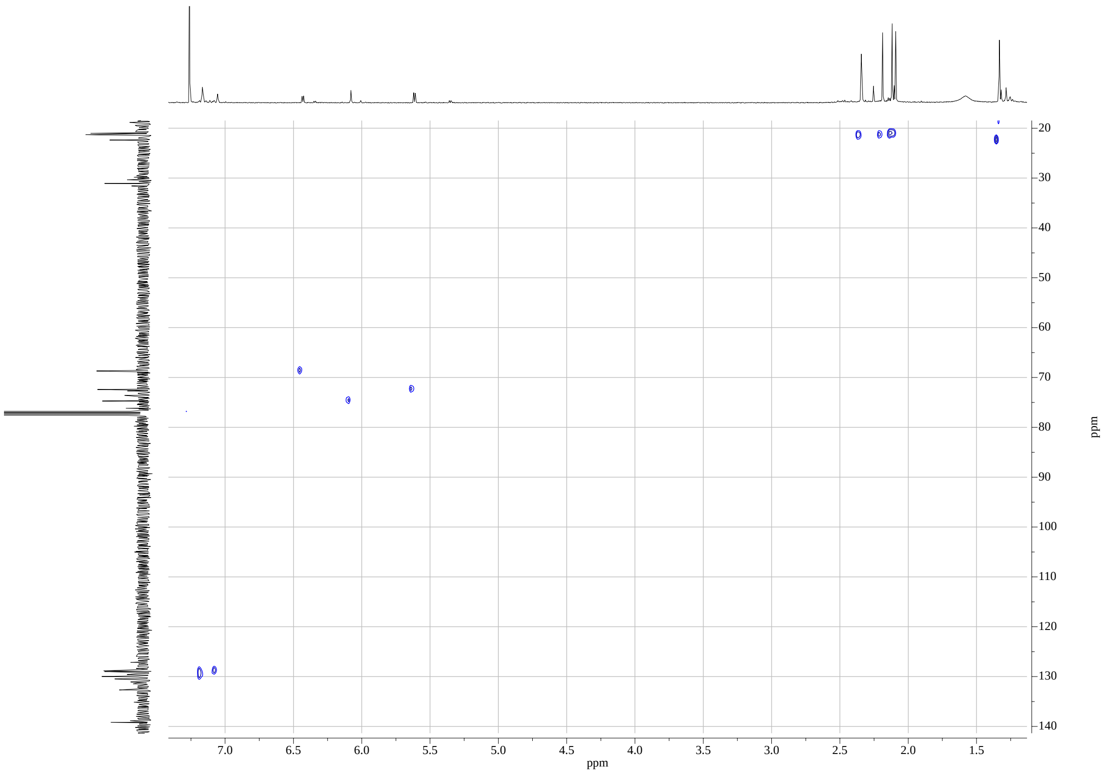

3c^{anti} - ^{1}H-^{13}C HMBC ($CDCl_3$)

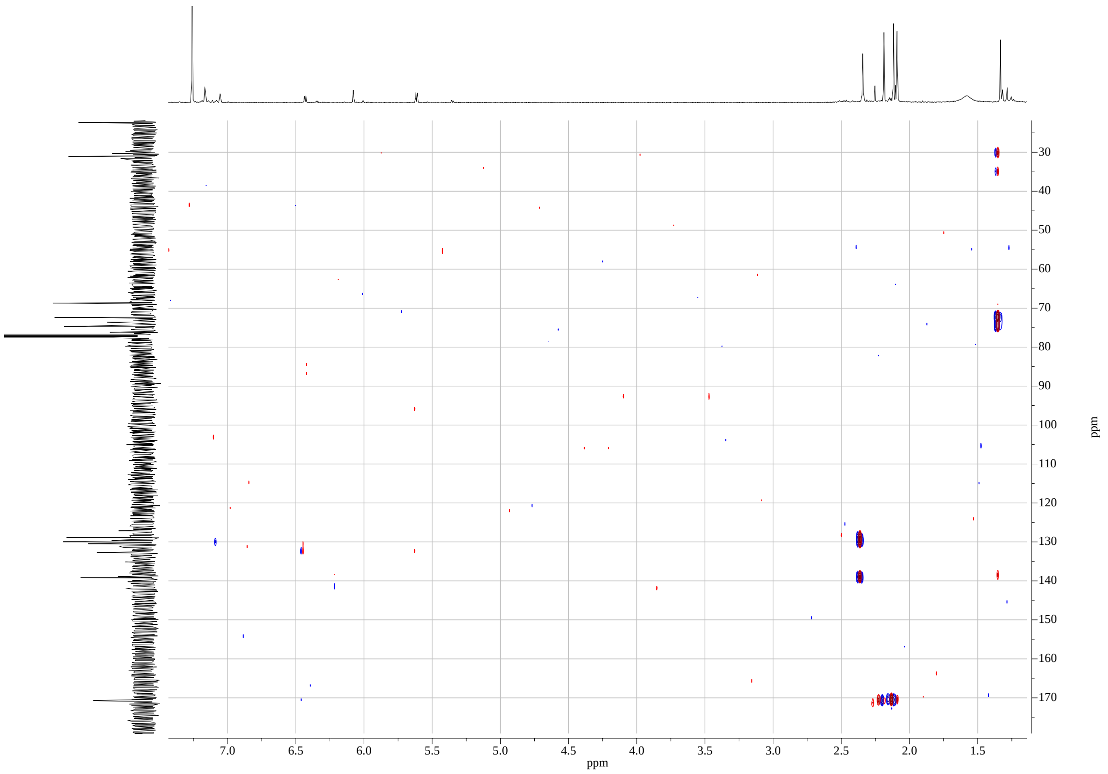

(1α,2α,3α,4α)-6-ethyl-1,2,3,4-tetrahydronaphthalene-1,2,3,4-tetrayl tetraacetate ($3g^{syn}$)

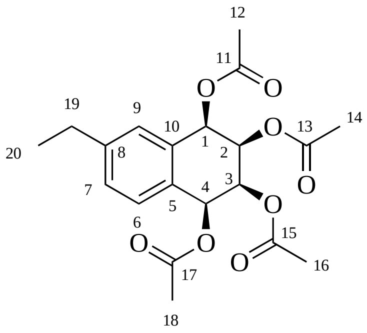

$3g^{syn}$ - ^{1}H NMR (400 MHz, $CDCl_3$)

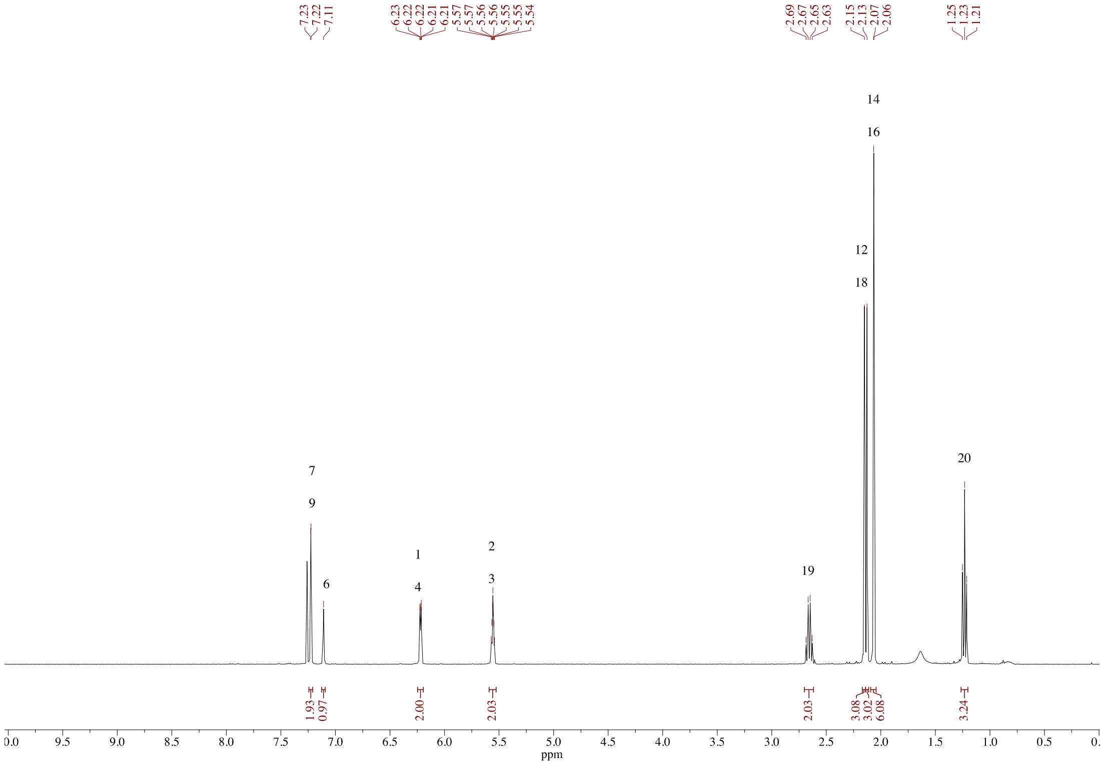

3g[syn] - ^{13}C NMR (100 MHz, $CDCl_3$)

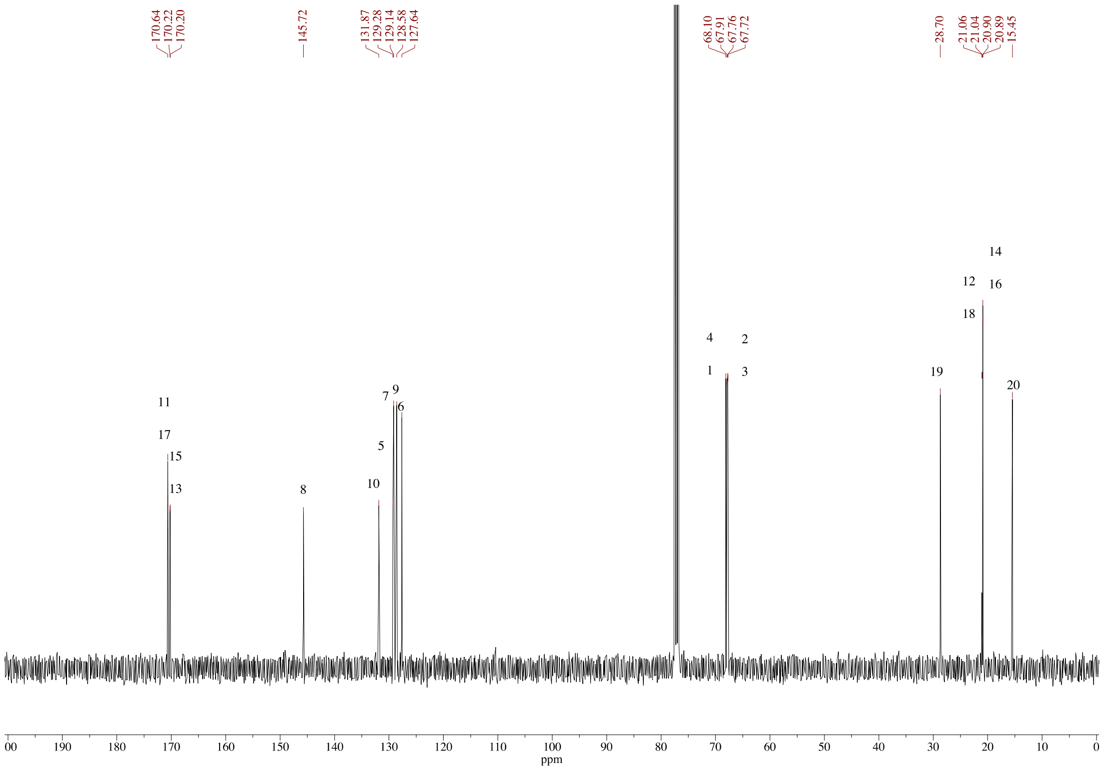

3g[syn] - DEPT ($CDCl_3$)

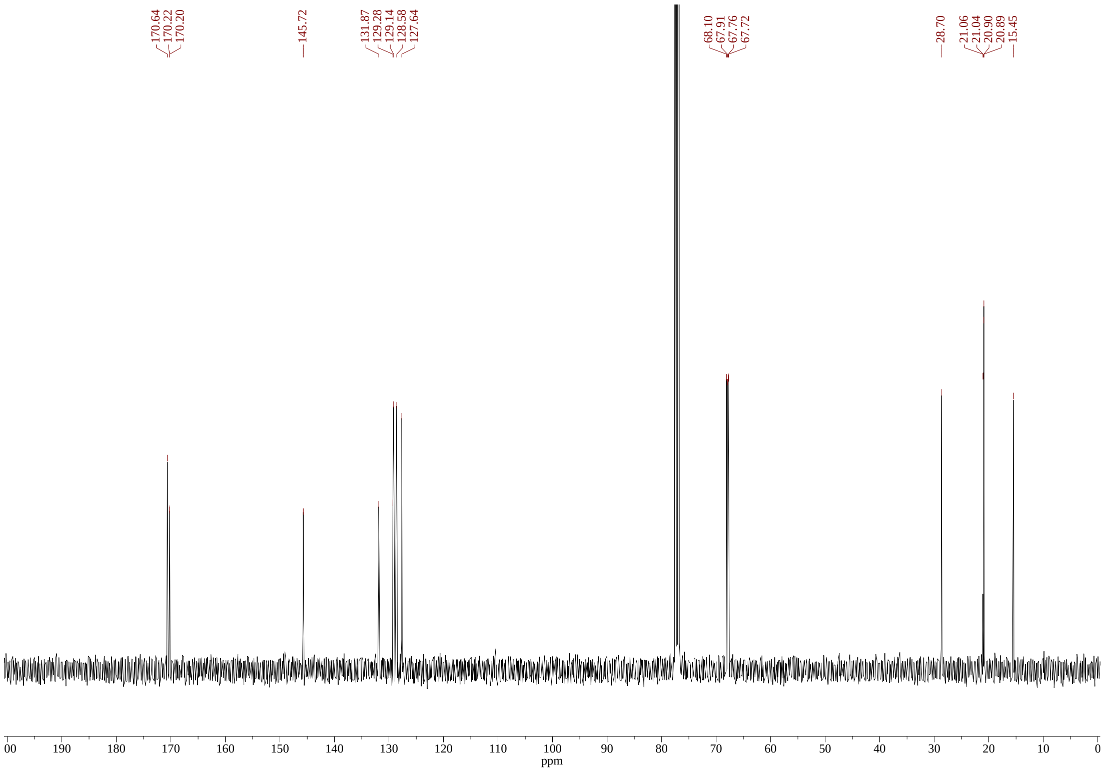

3g^{syn} - DEPTQ ($CDCl_3$)

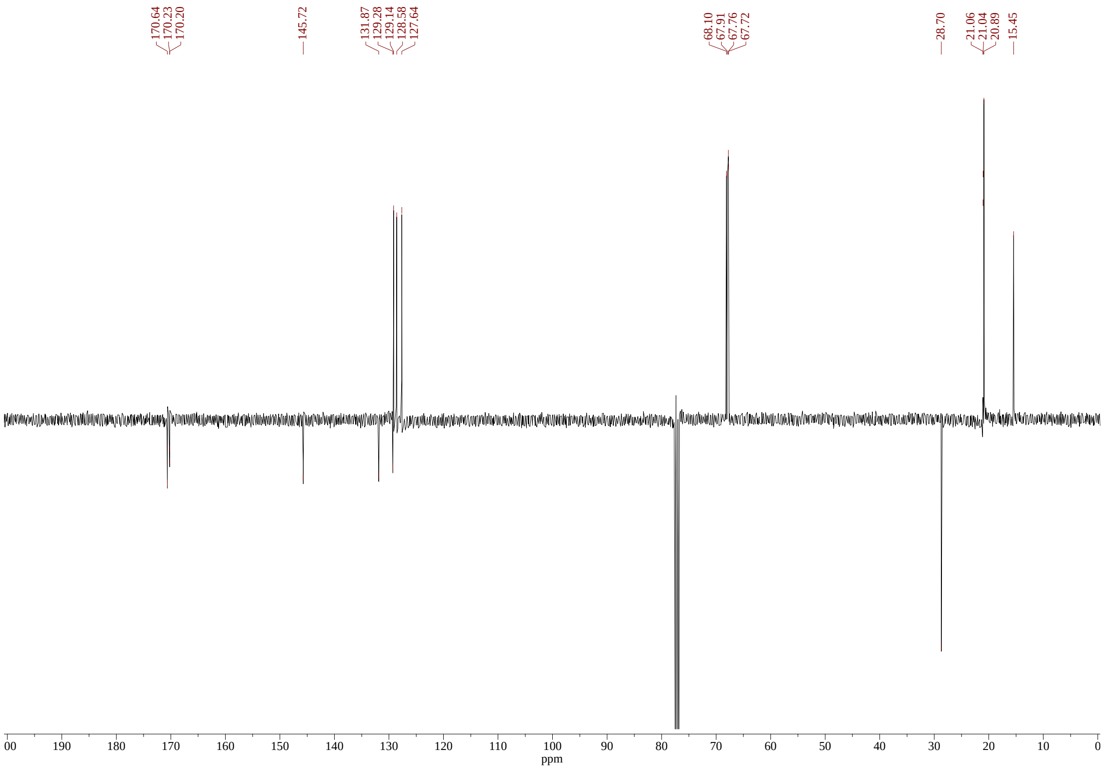

3g^{syn} - ^{1}H-^{1}H COSY ($CDCl_3$)

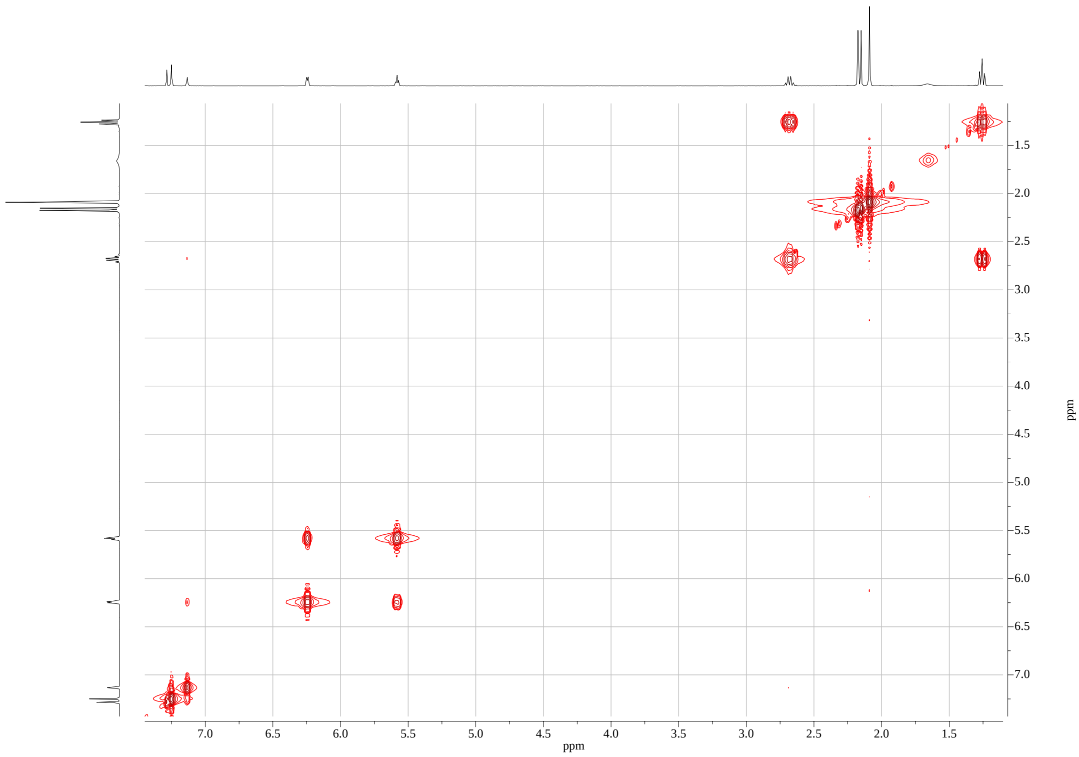

3g^syn^ - ^{1}H-^{13}C HSQCED ($CDCl_3$)

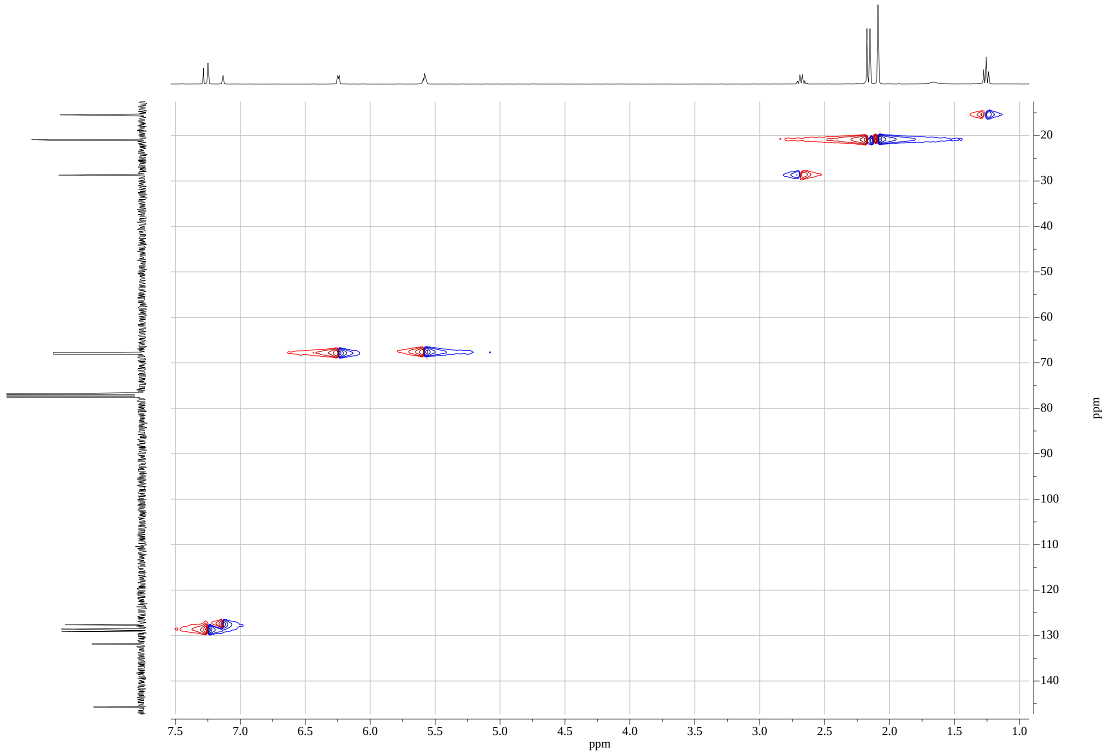

3g^syn^ - ^{1}H-^{13}C HMBC ($CDCl_3$)

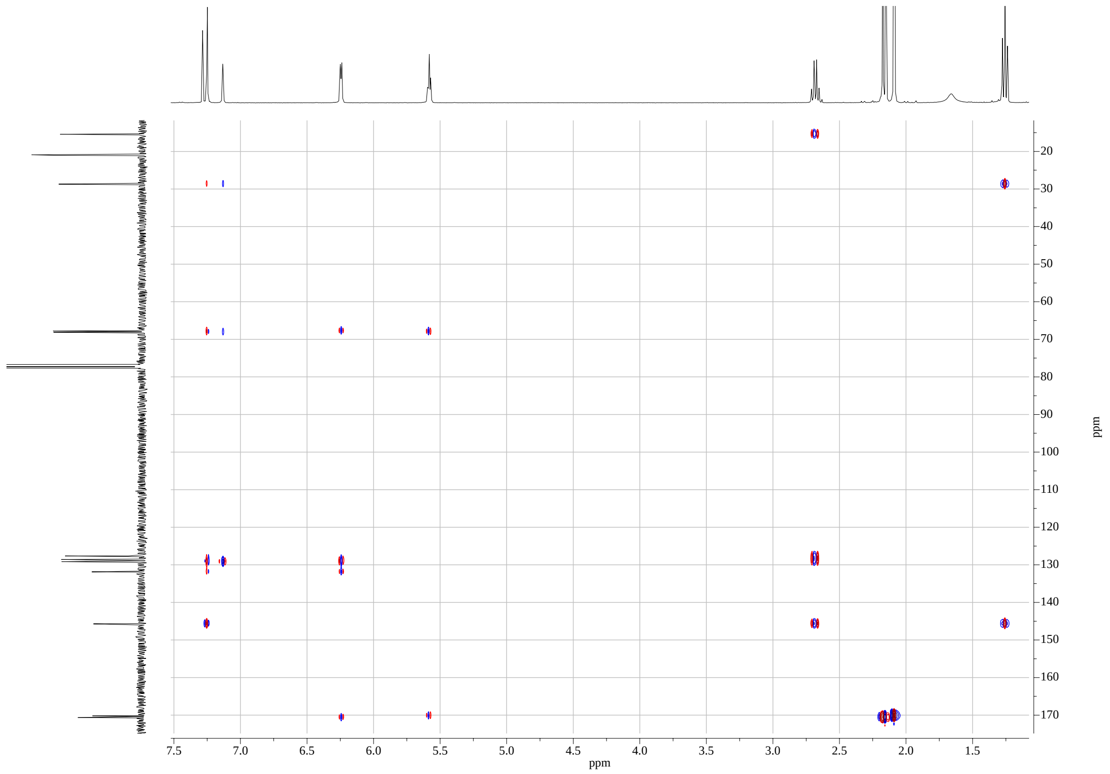

(1α,2α,3β,4β)-6-ethyl-1,2,3,4-tetrahydronaphthalene-1,2,3,4-tetrayl tetraacetate (3g^anti^)

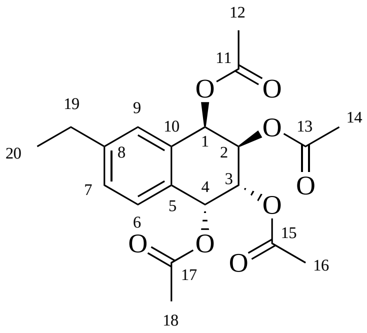

3g^anti^ - ^{1}H NMR (400 MHz, CDCl$_3$)

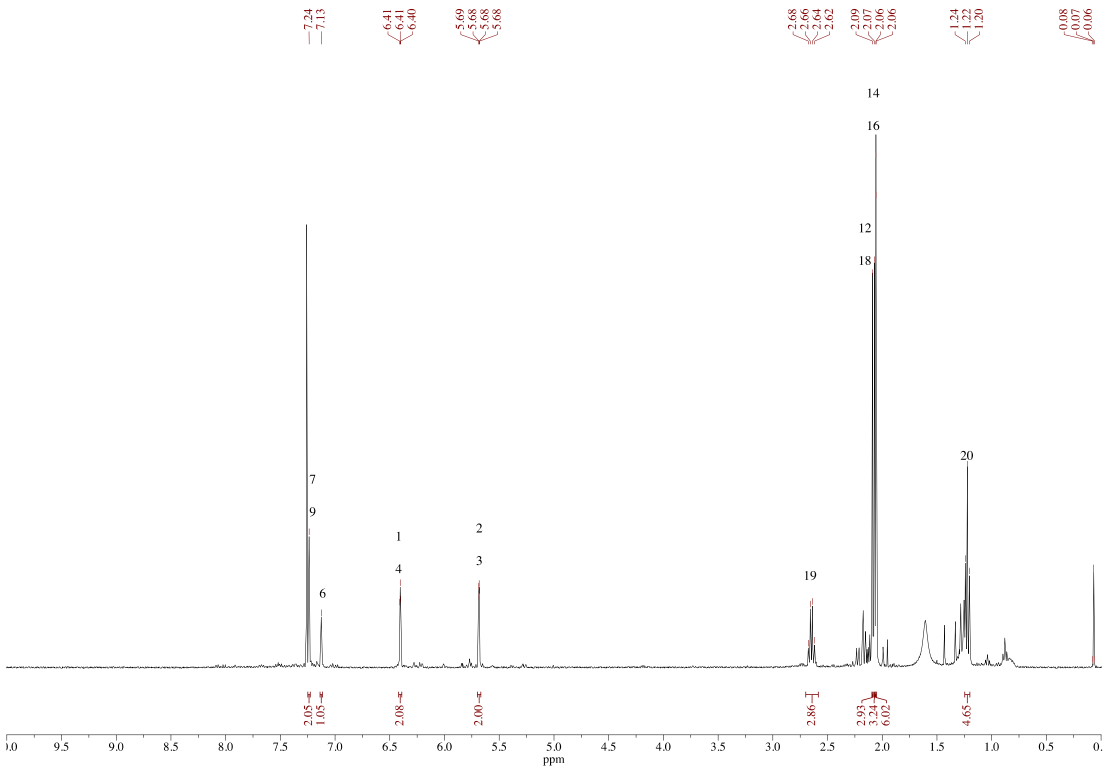

3g[anti] - ^{13}C NMR (100 MHz, $CDCl_3$)

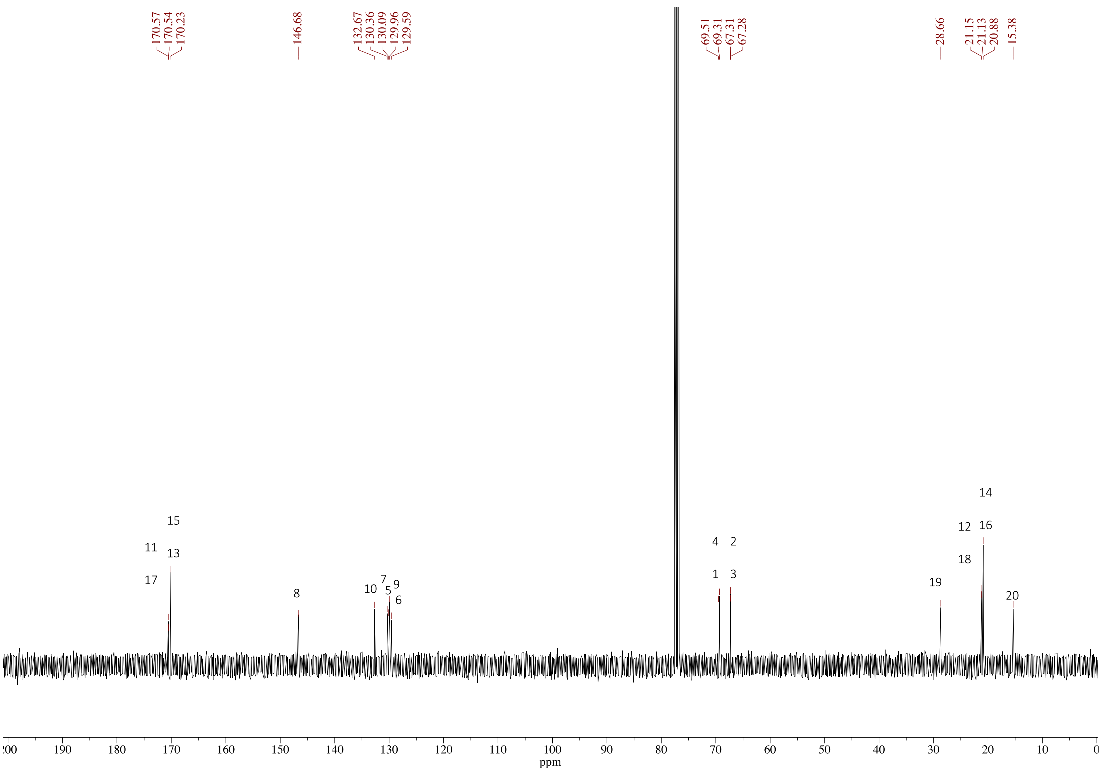

3g[anti] - DEPT ($CDCl_3$)

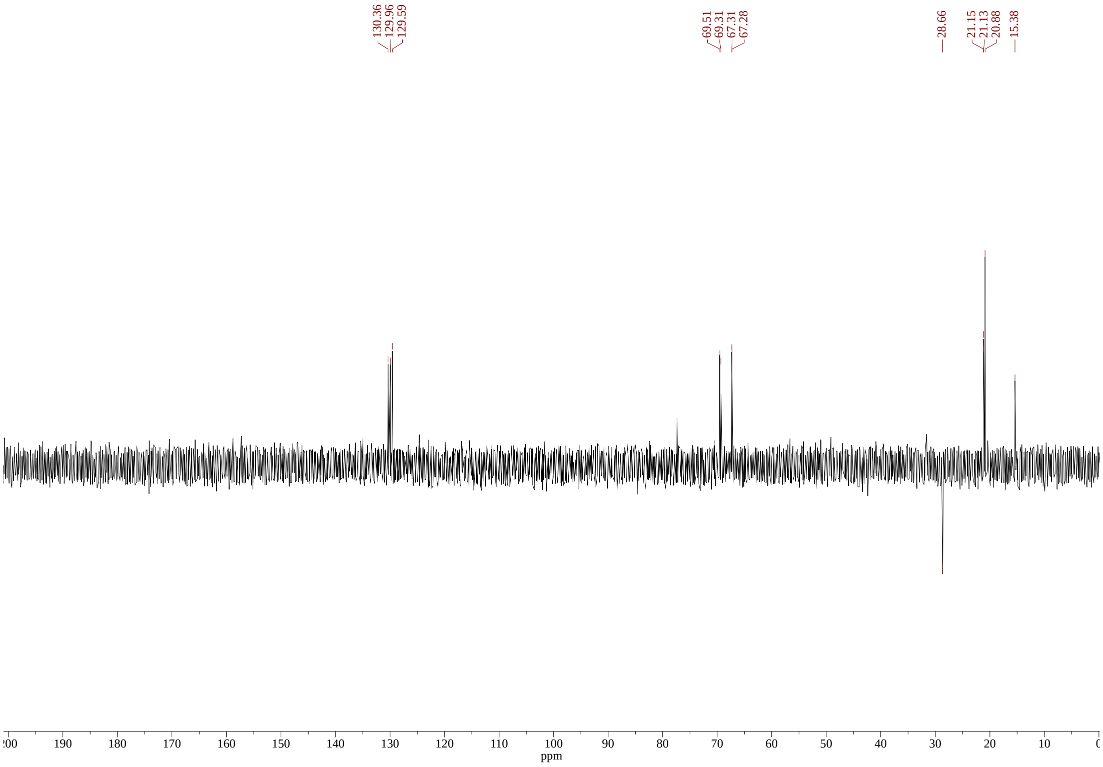

3g^{anti} - DEPTQ ($CDCl_3$)

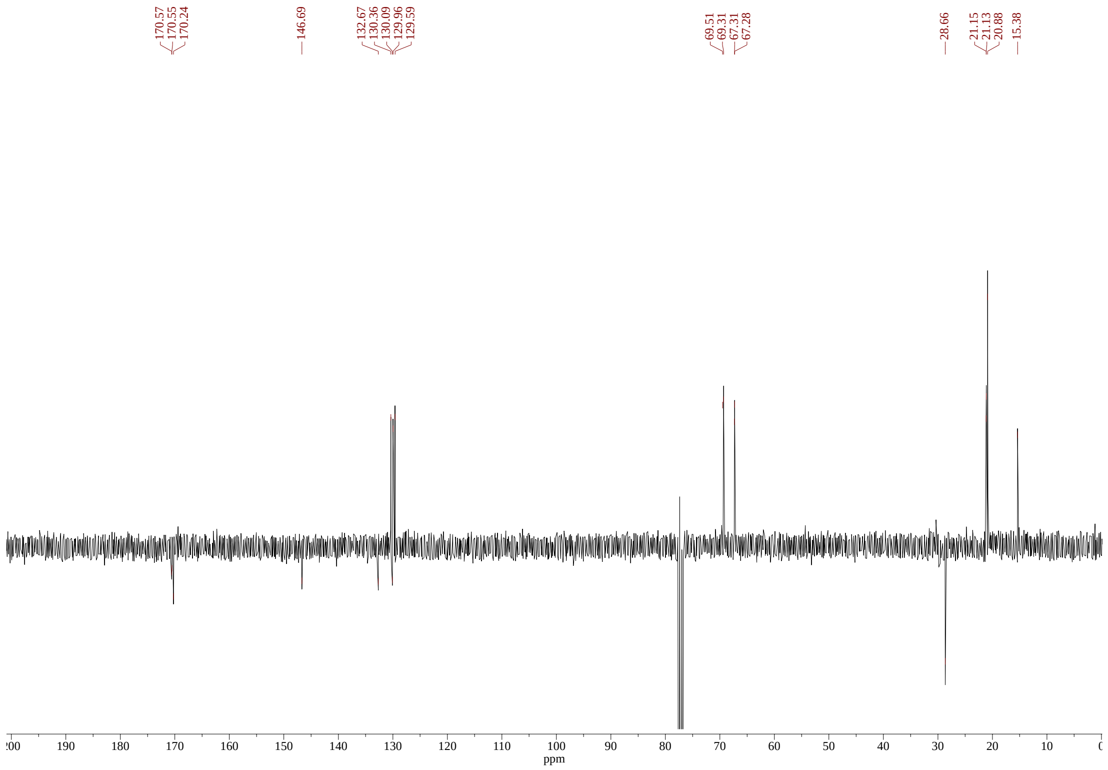

3g^{anti} - ^{1}H-^{1}H COSY ($CDCl_3$)

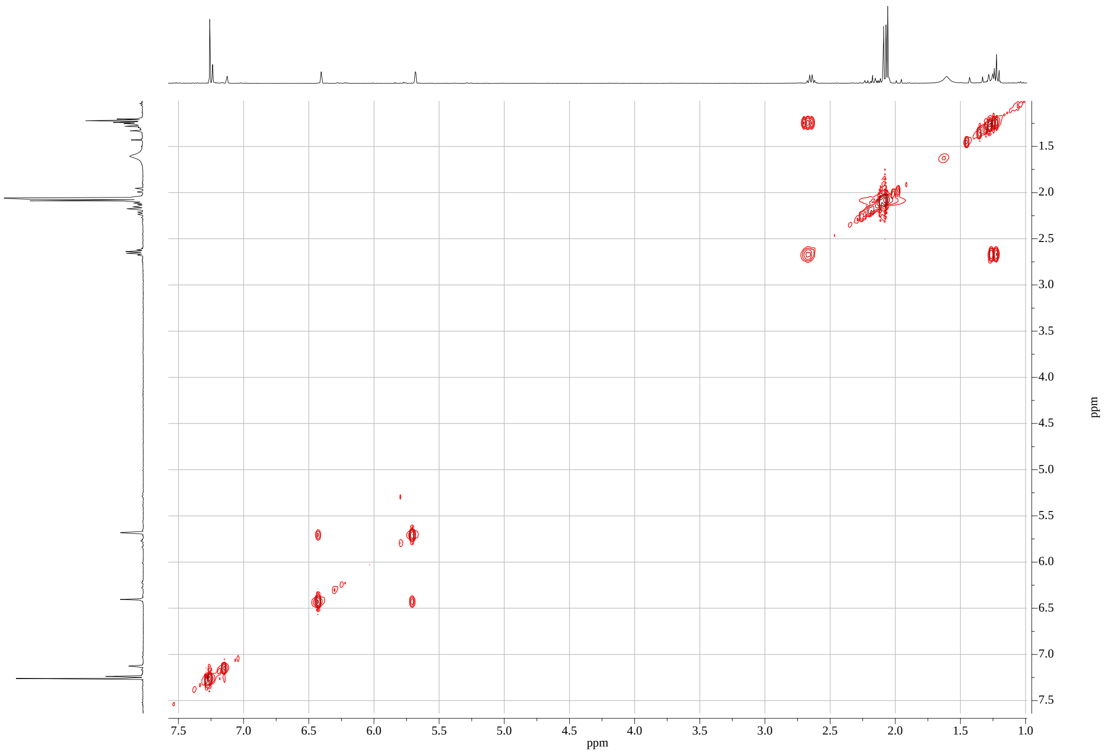

3g^anti^ - ^{1}H-^{13}C HSQCED ($CDCl_3$)

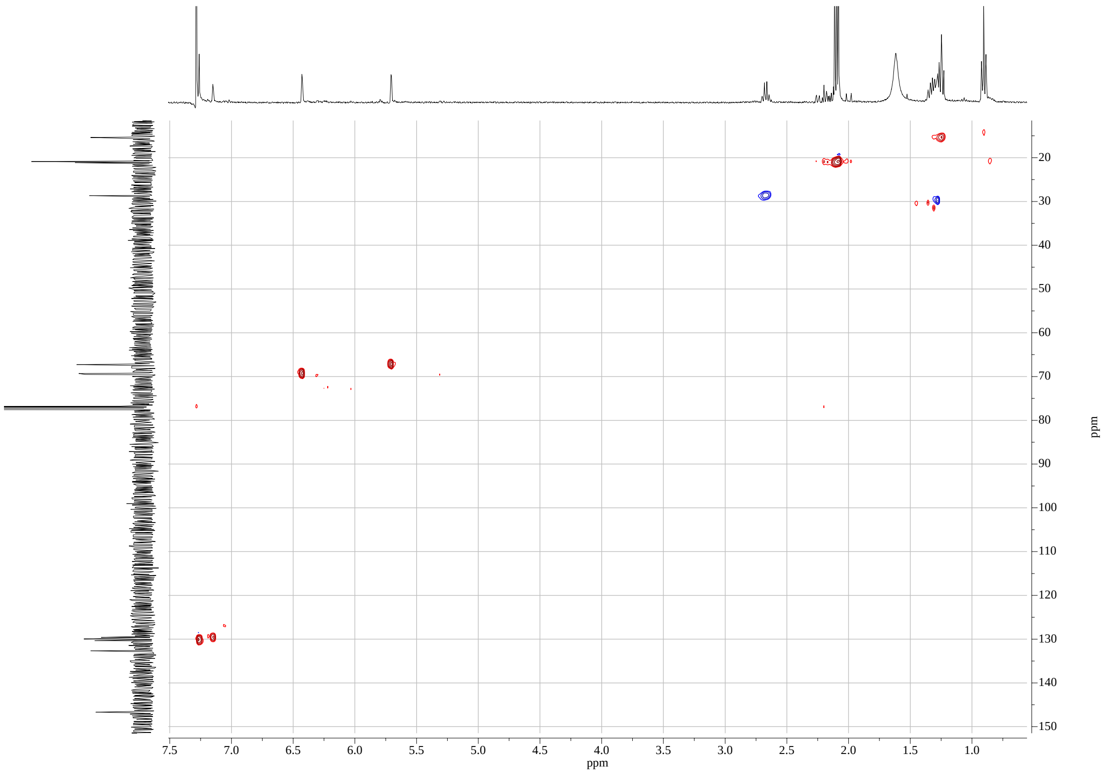

3g^anti^ - ^{1}H-^{13}C HMBC ($CDCl_3$)

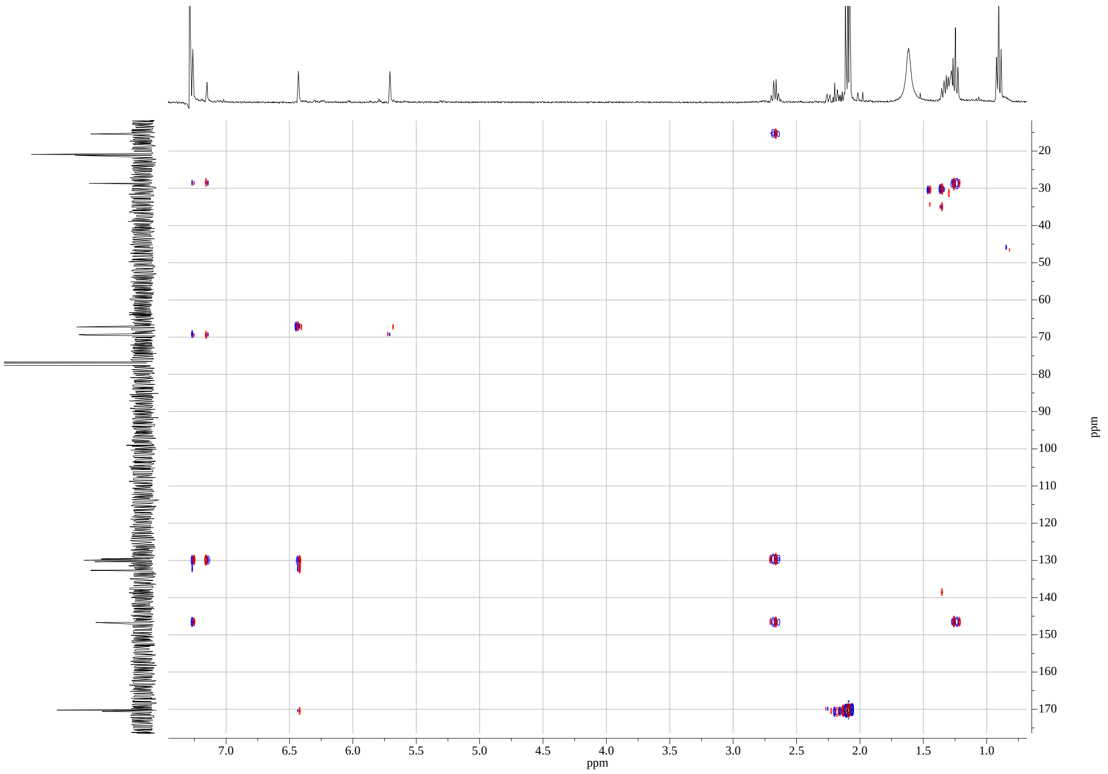

(1α,2α,3α,4α)-6-cyano-1,2,3,4-tetrahydronaphthalene-1,2,3,4-tetrayl tetraacetate (3j^{syn})

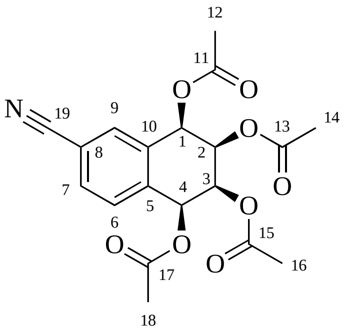

3j^{syn} - ^{1}H NMR (400 MHz, $CDCl_3$)

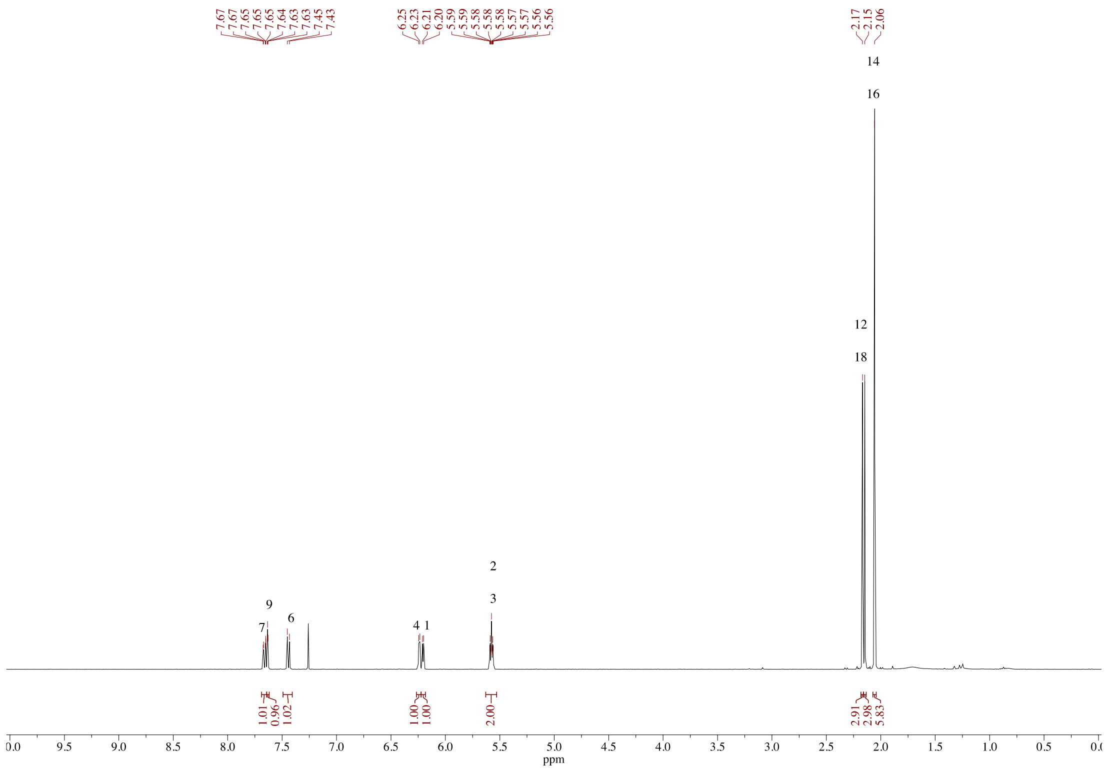

3j^{syn} - ^{13}C NMR (100 MHz, $CDCl_3$)

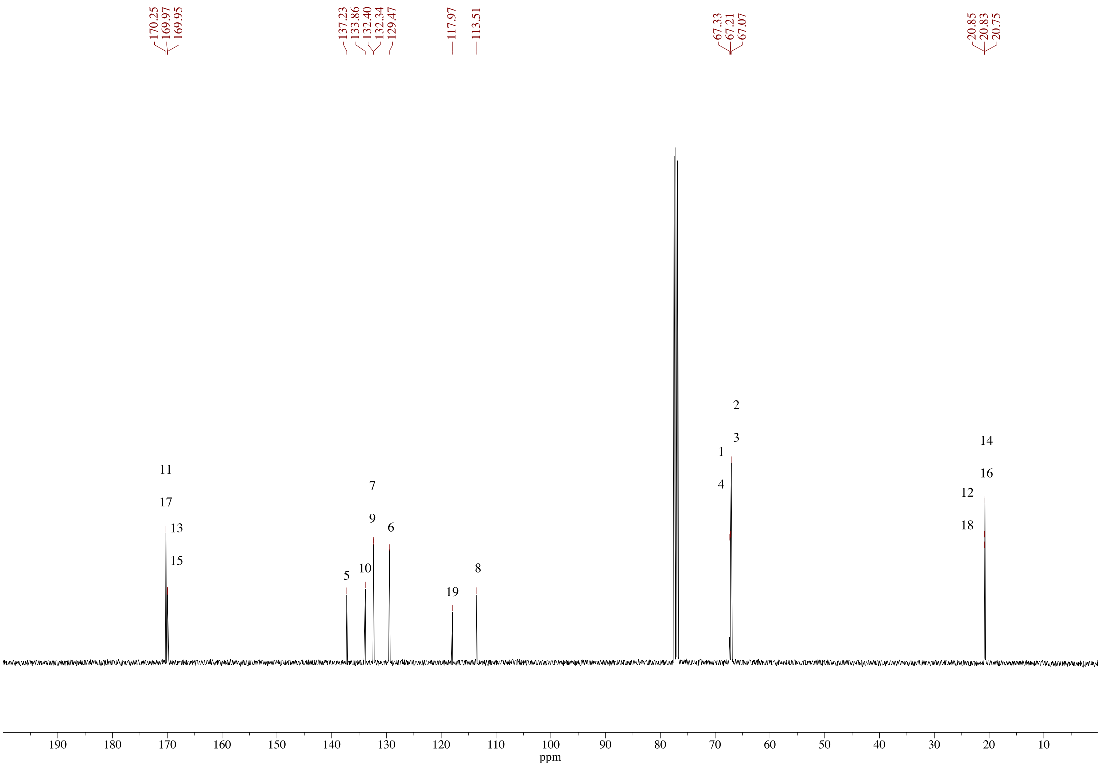

3j^{syn} - DEPT ($CDCl_3$)

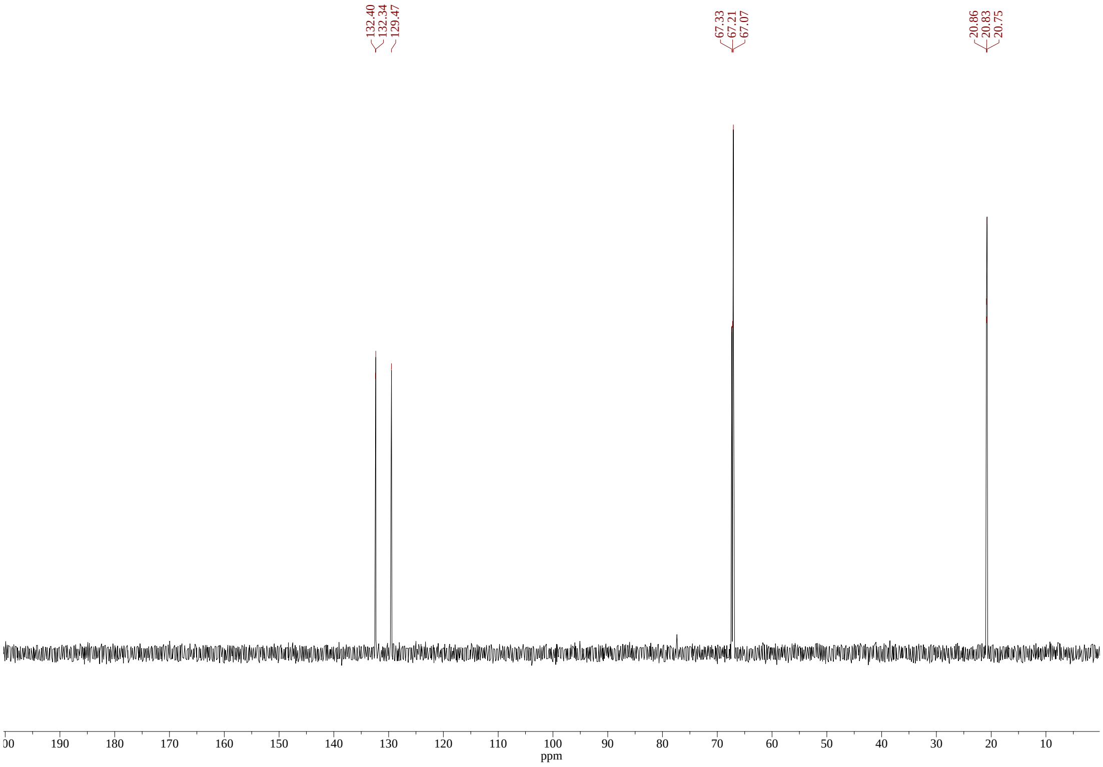

3j^{syn} - DEPTQ ($CDCl_3$)

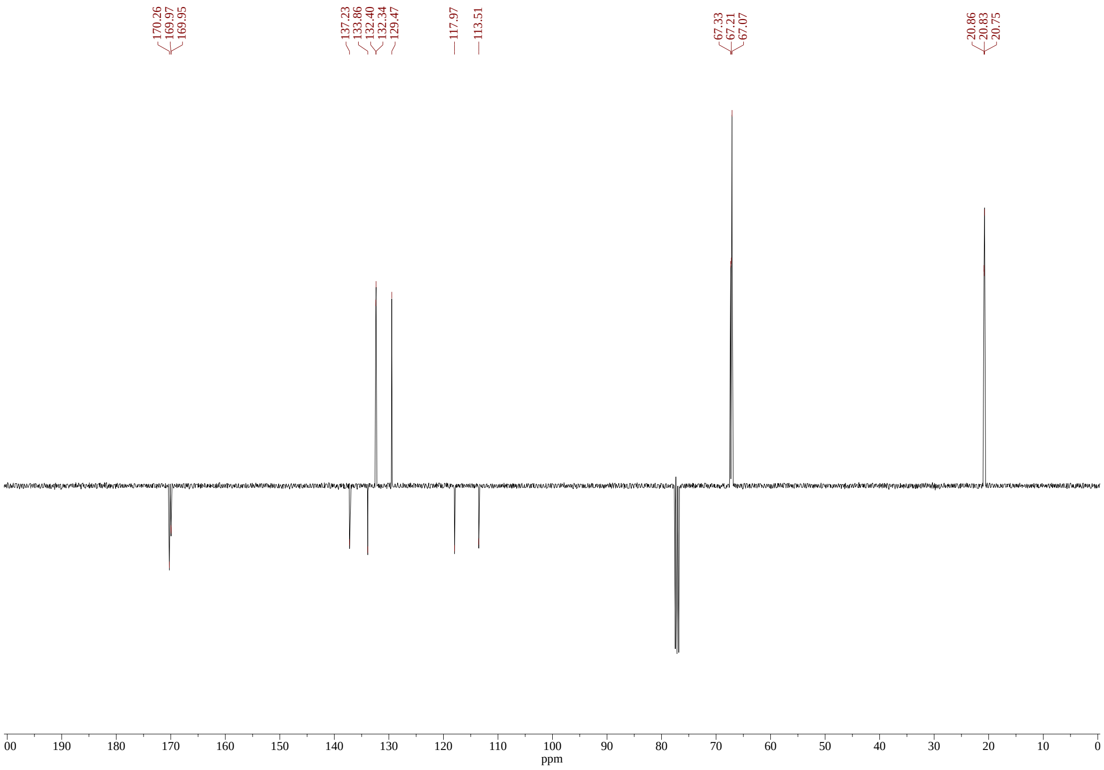

3j^{syn} - ^{1}H-^{1}H COSY ($CDCl_3$)

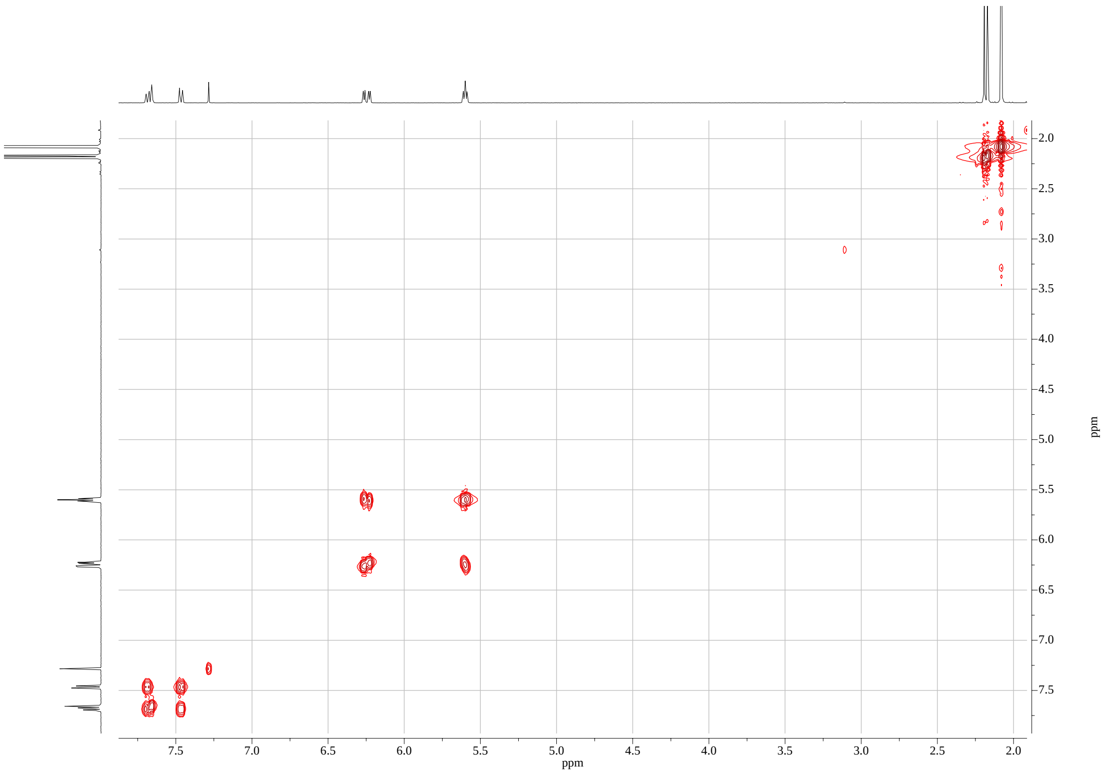

3j^{syn} - ^{1}H-^{13}C HSQCED ($CDCl_3$)

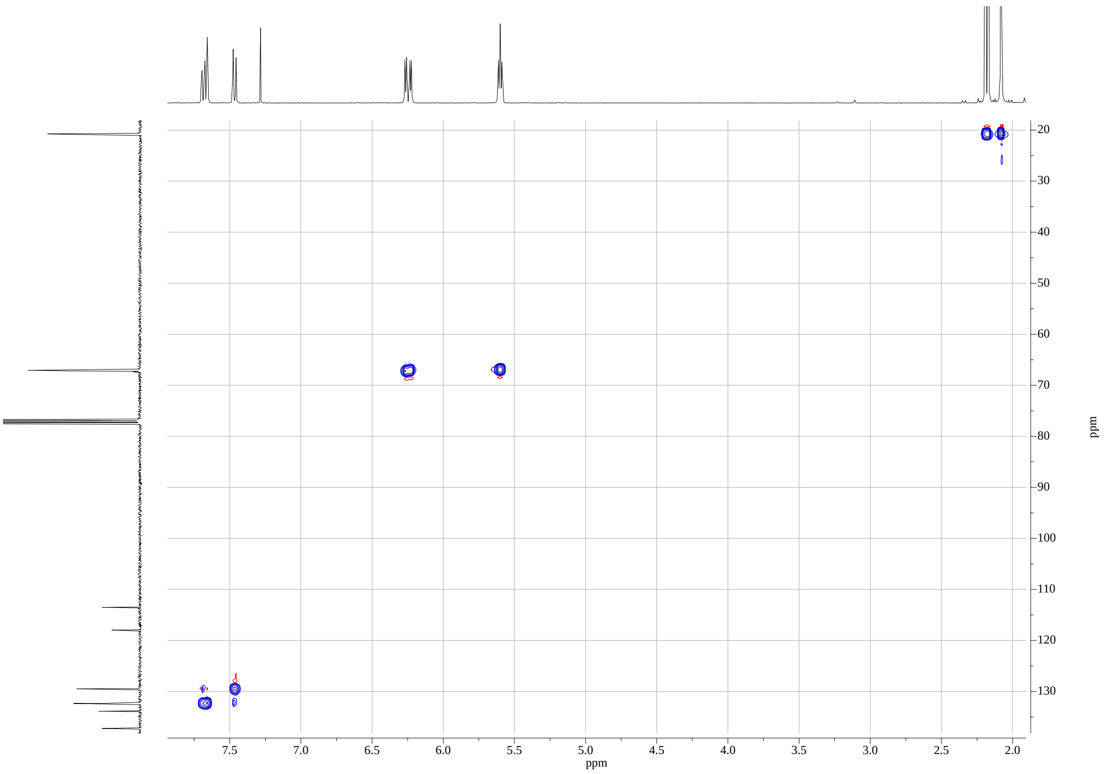

3j^{syn} - ^{1}H-^{13}C HMBC ($CDCl_3$)

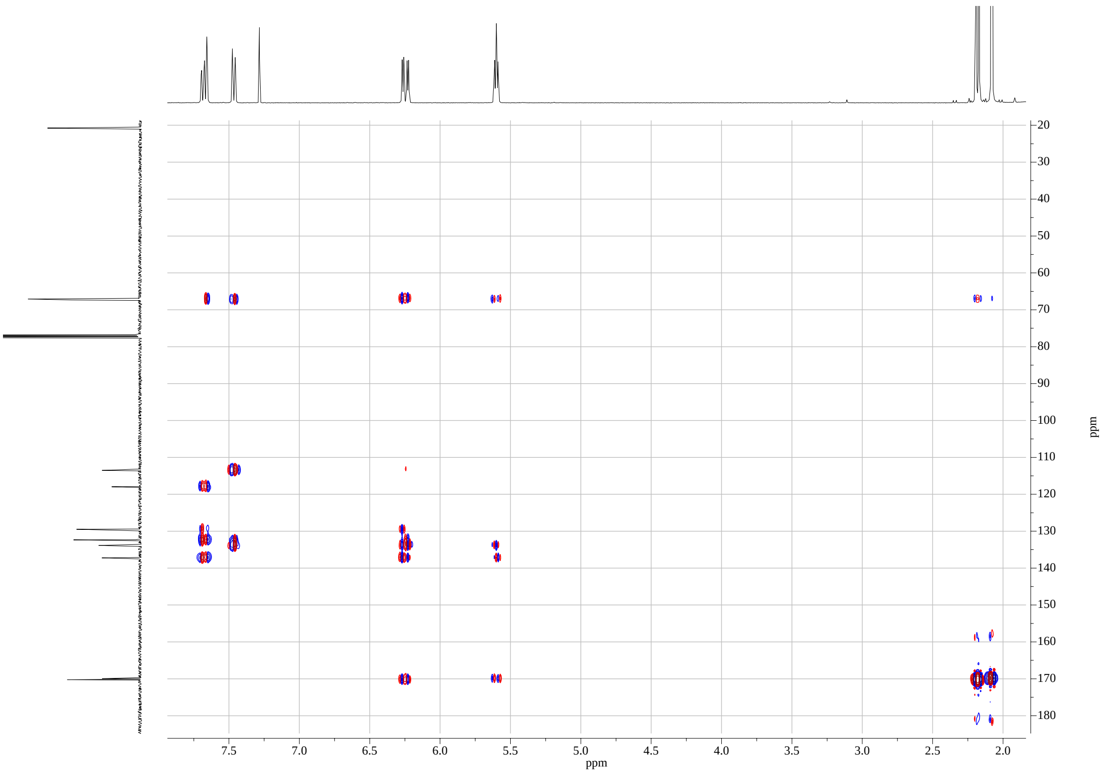

(1α,2α,3β,4β)-6-cyano-1,2,3,4-tetrahydronaphthalene-1,2,3,4-tetrayl tetraacetate (3j[anti])

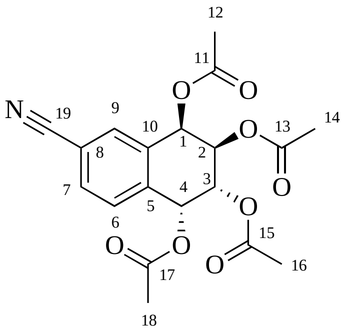

3j[anti] - ^{1}H NMR (400 MHz, $CDCl_3$)

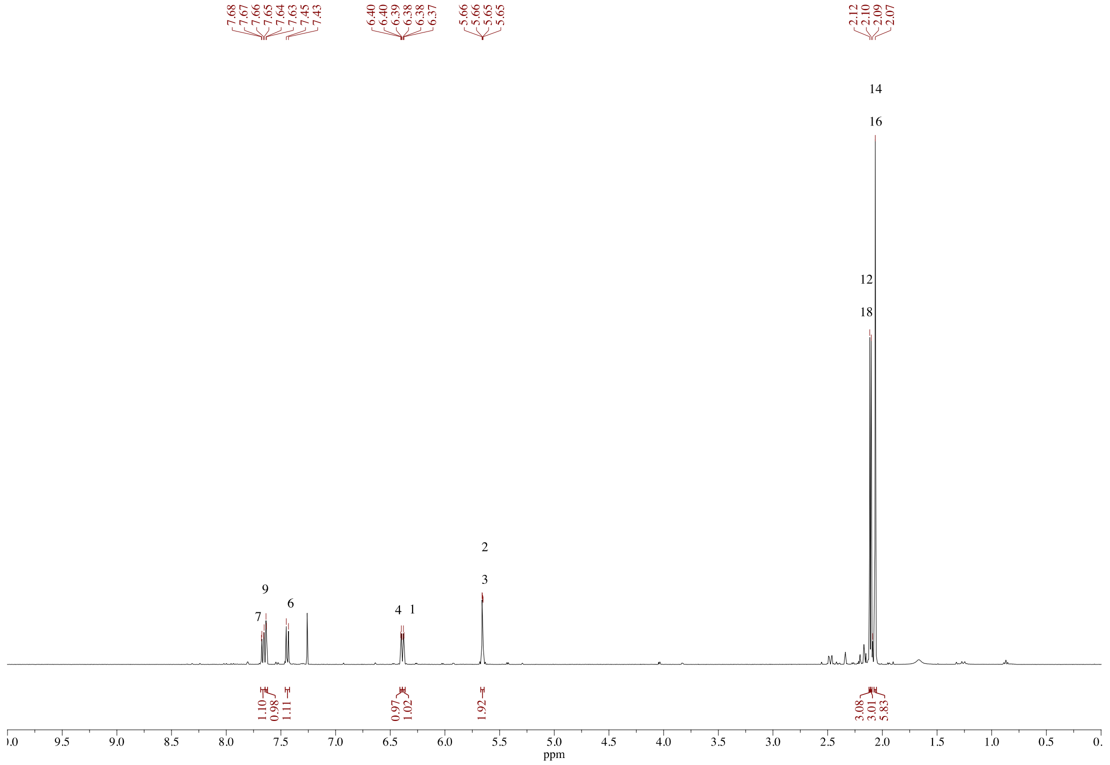

3j^anti^ - ^{13}C NMR (100 MHz, $CDCl_3$)

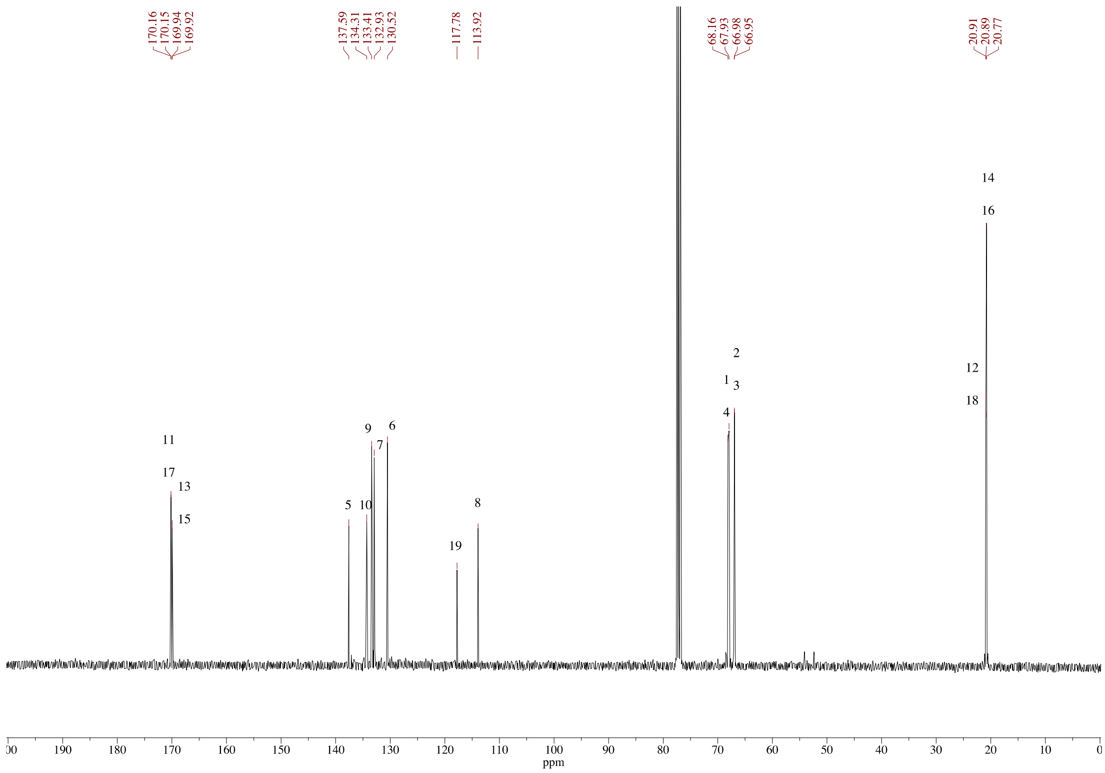

3j^anti^ - DEPT ($CDCl_3$)

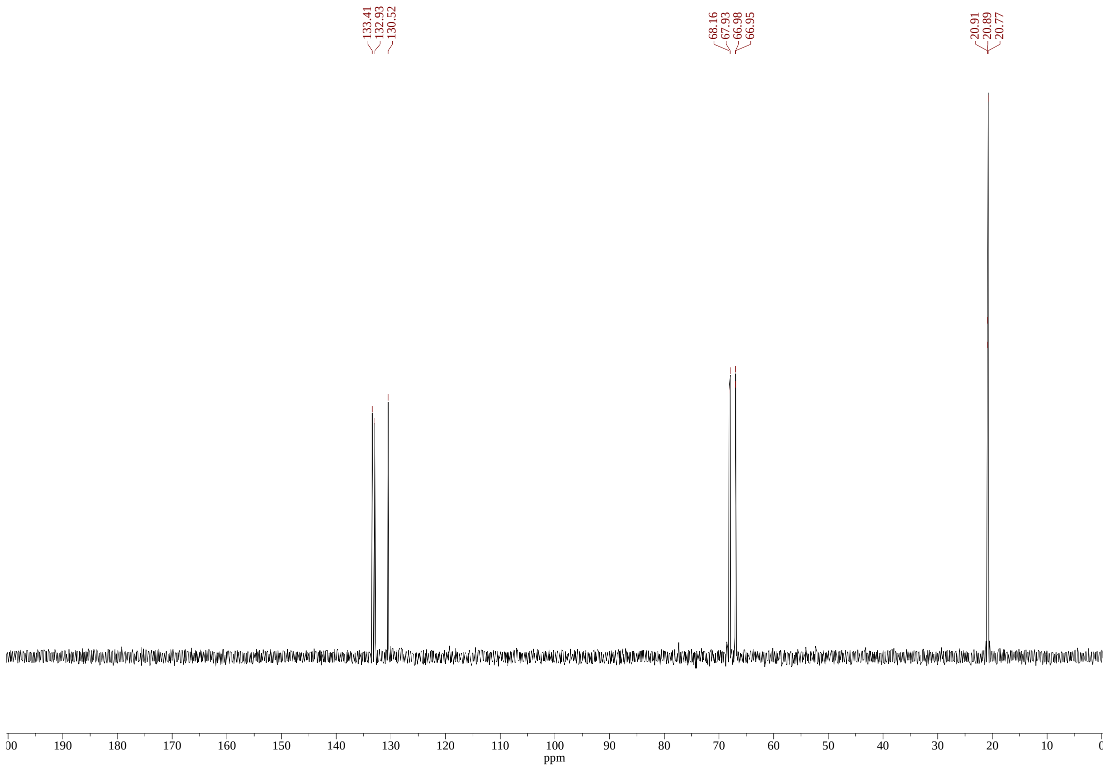

3j[anti] - DEPTQ ($CDCl_3$)

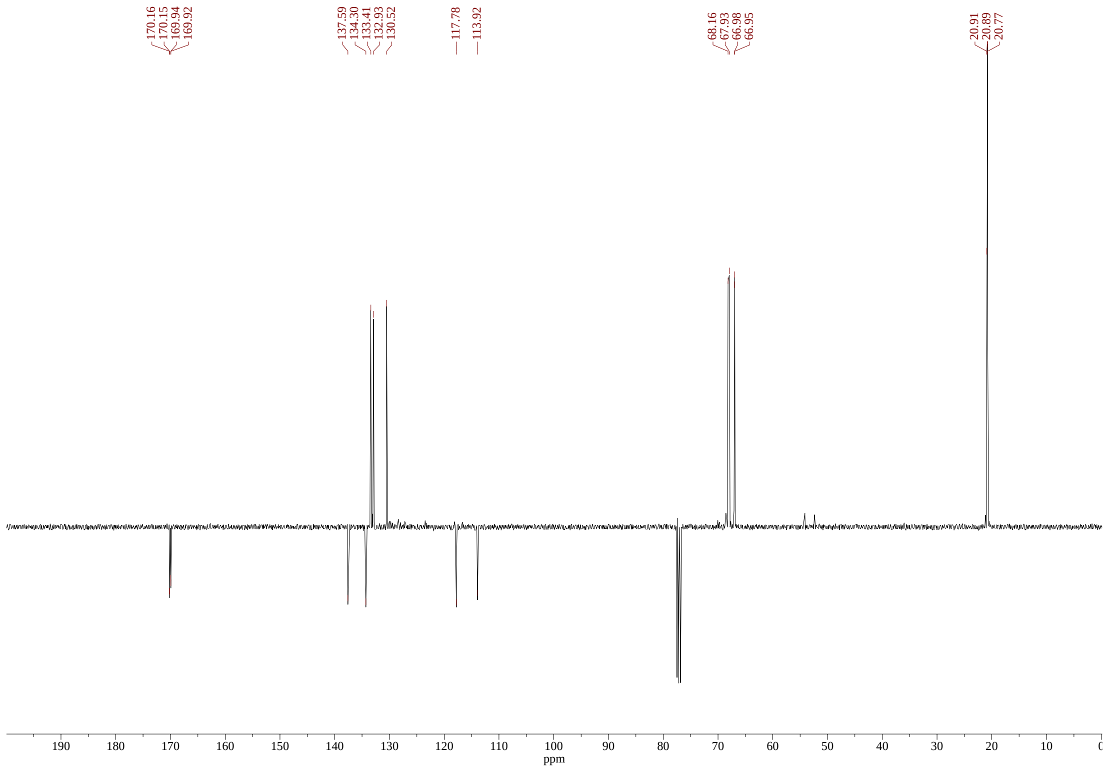

3j[anti] - ^1H-^1H COSY ($CDCl_3$)

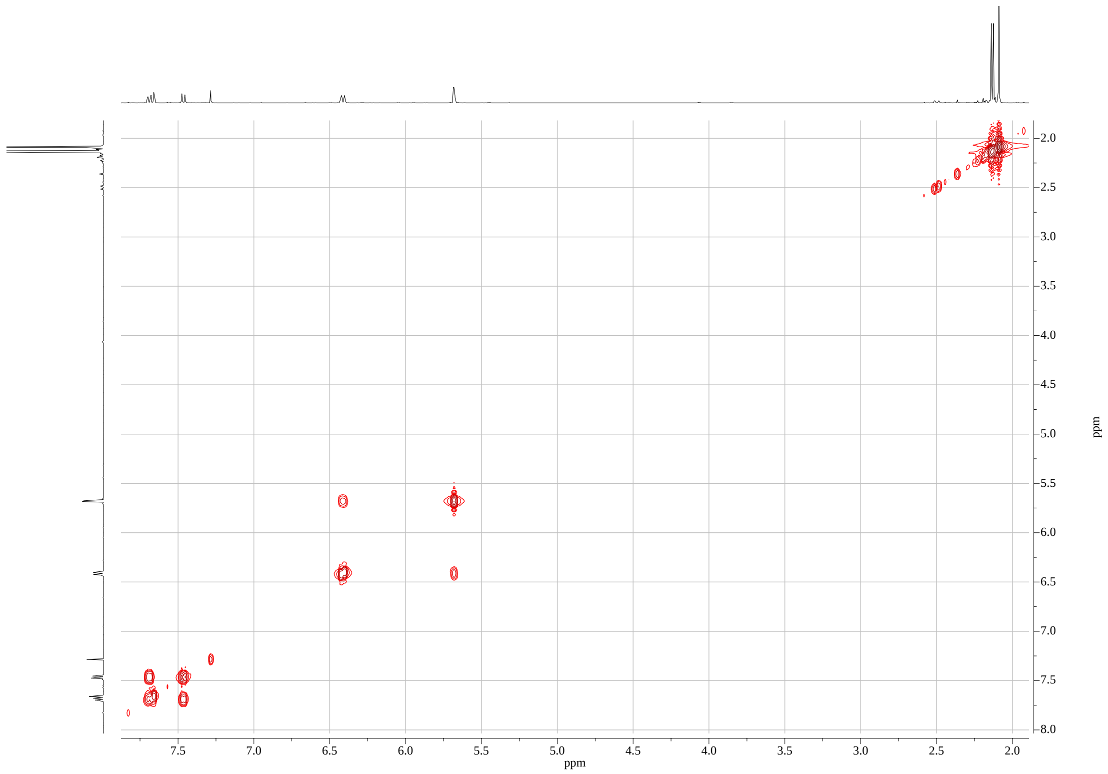

3j^{anti} - ^{1}H-^{13}C HSQCED ($CDCl_3$)

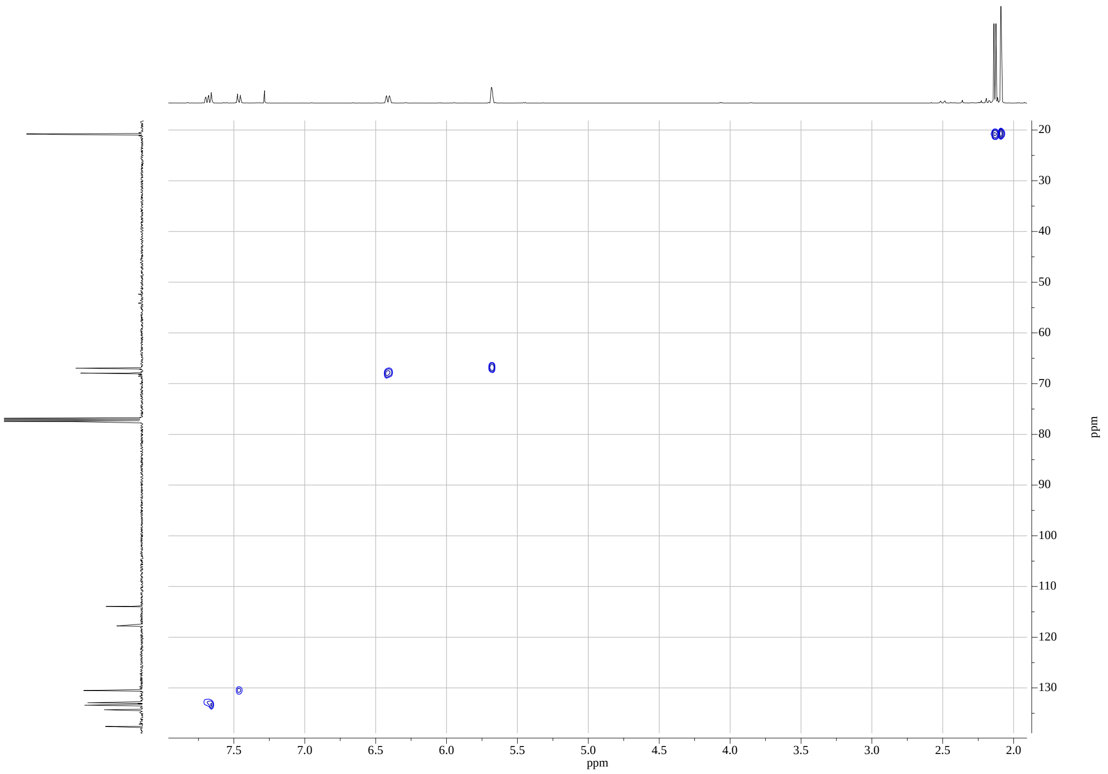

3j^{anti} - ^{1}H-^{13}C HMBC ($CDCl_3$)

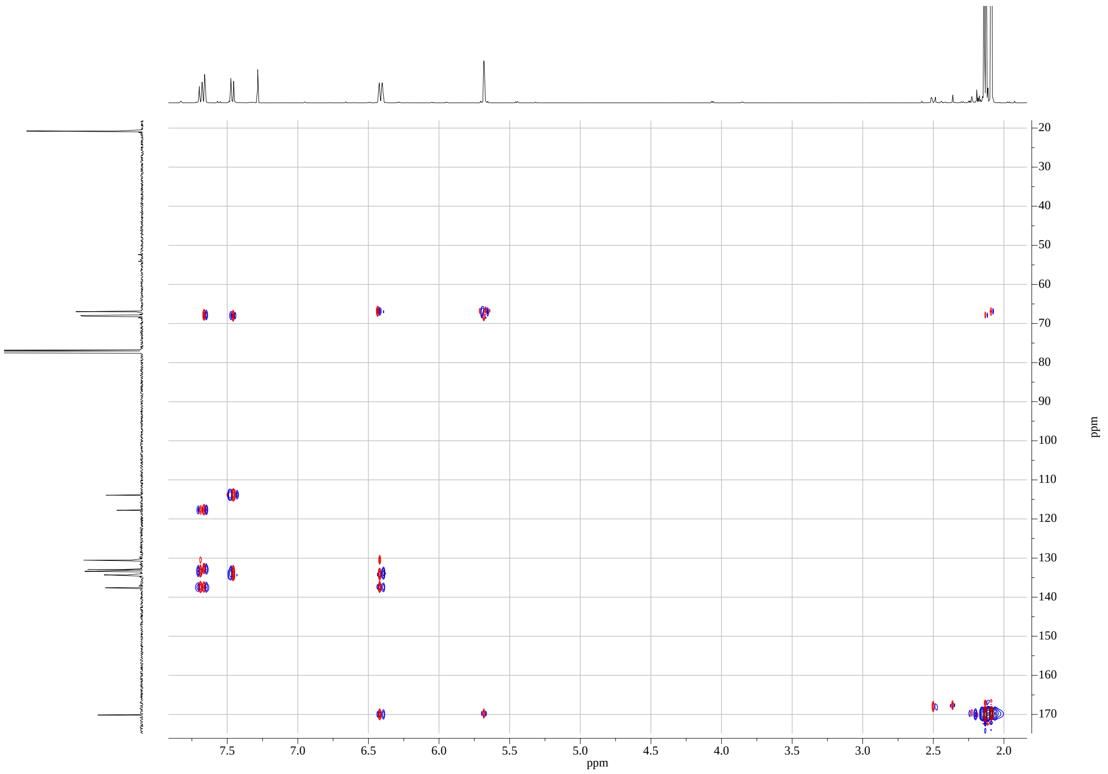

(1α,2α,3α,4α)-5-cyano-1,2,3,4-tetrahydronaphthalene-1,2,3,4-tetrayl tetraacetate (3q^{syn})

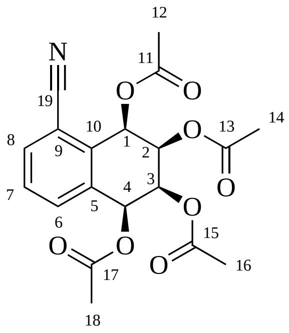

3q^{syn} - ^{1}H NMR (400 MHz, $CDCl_3$)

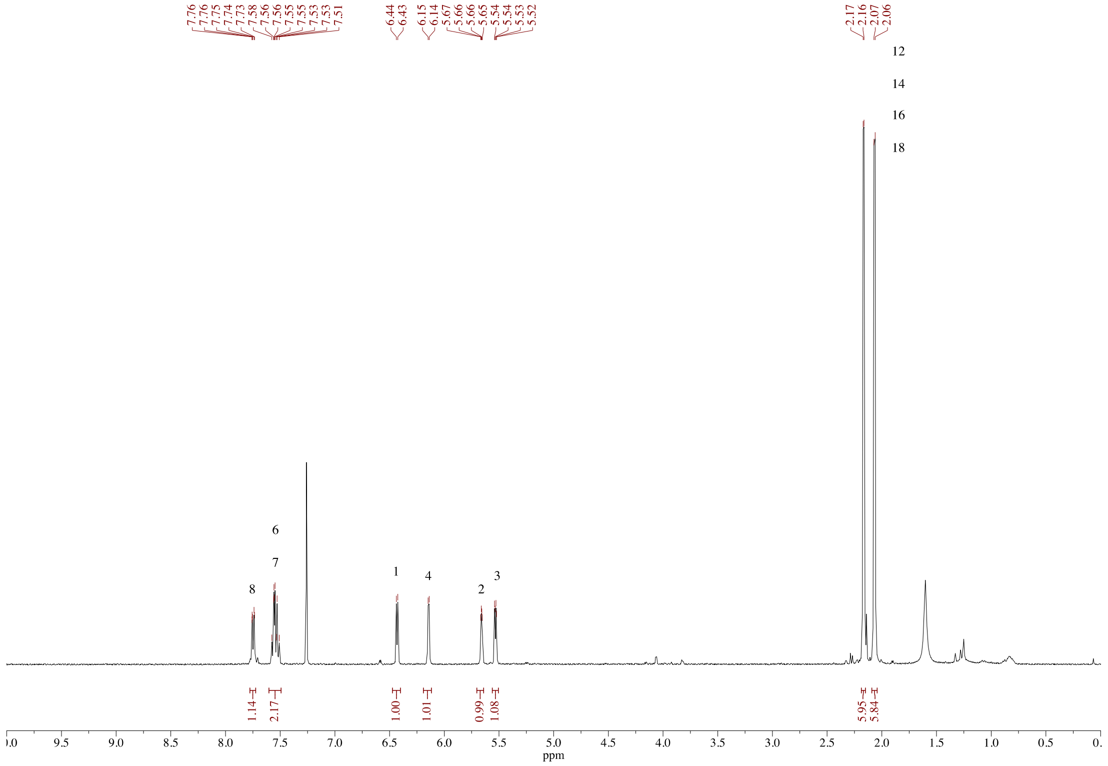

3q^{syn} - ^{13}C NMR (100 MHz, $CDCl_3$)

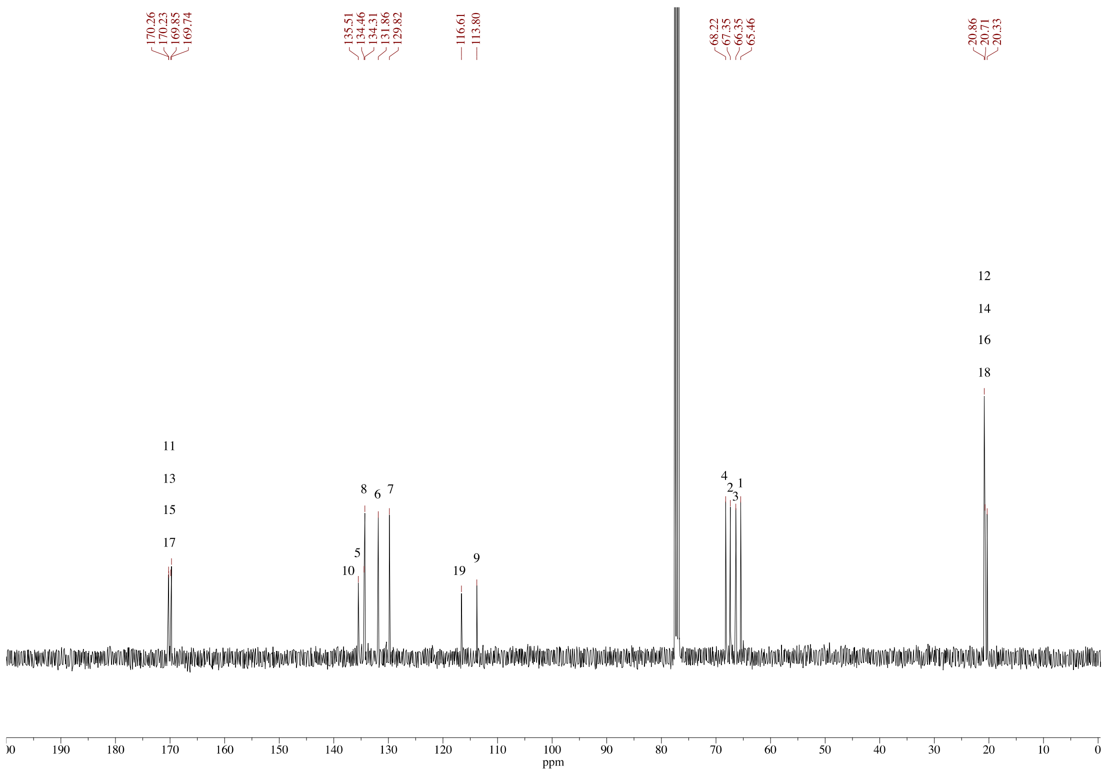

3q^{syn} - DEPT ($CDCl_3$)

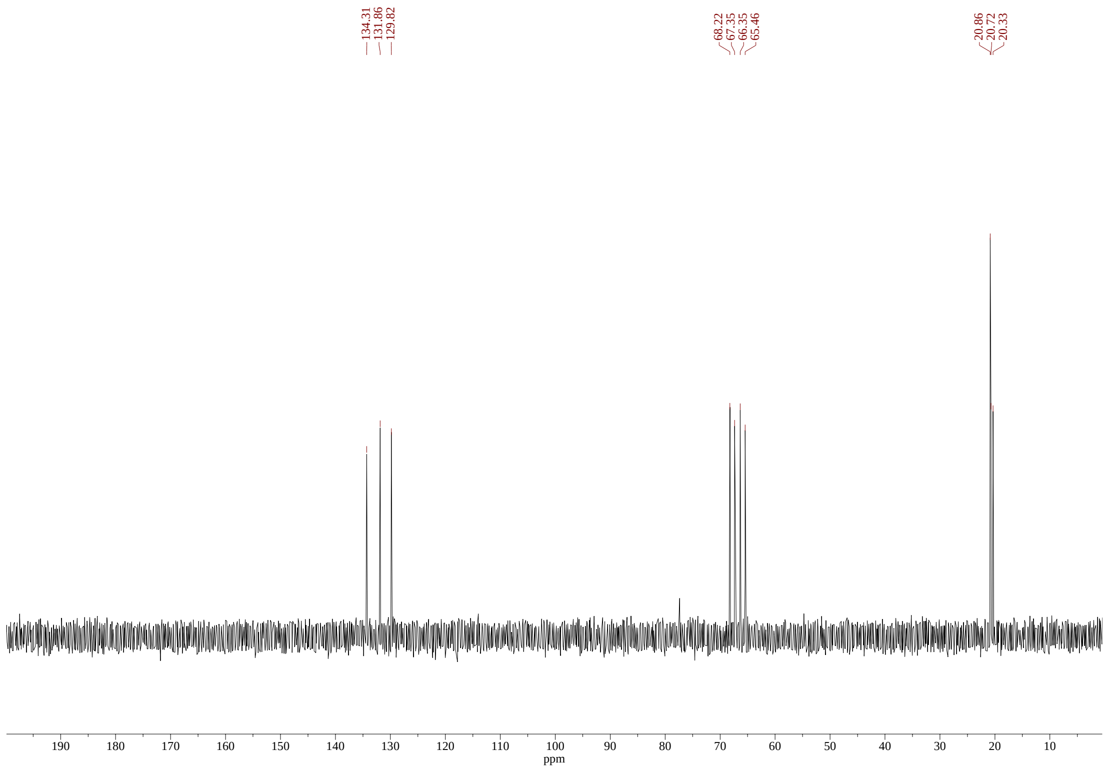

3q^{syn} - DEPTQ ($CDCl_3$)

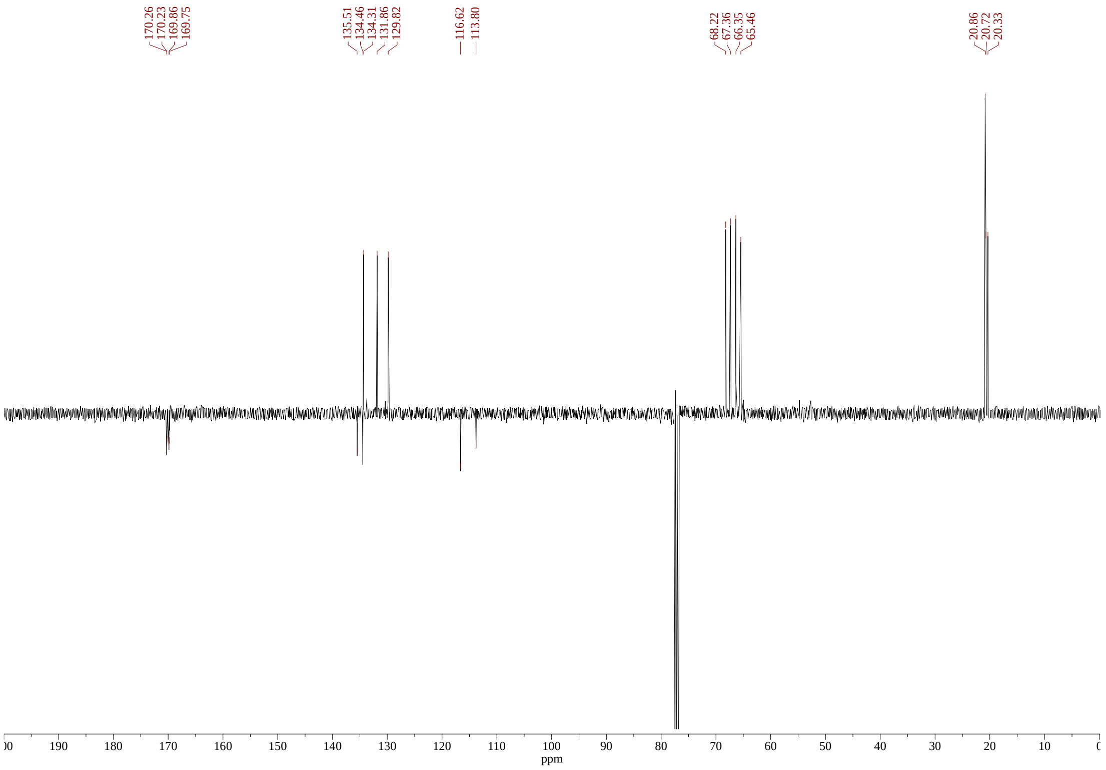

3q^{syn} - ^{1}H-^{1}H COSY ($CDCl_3$)

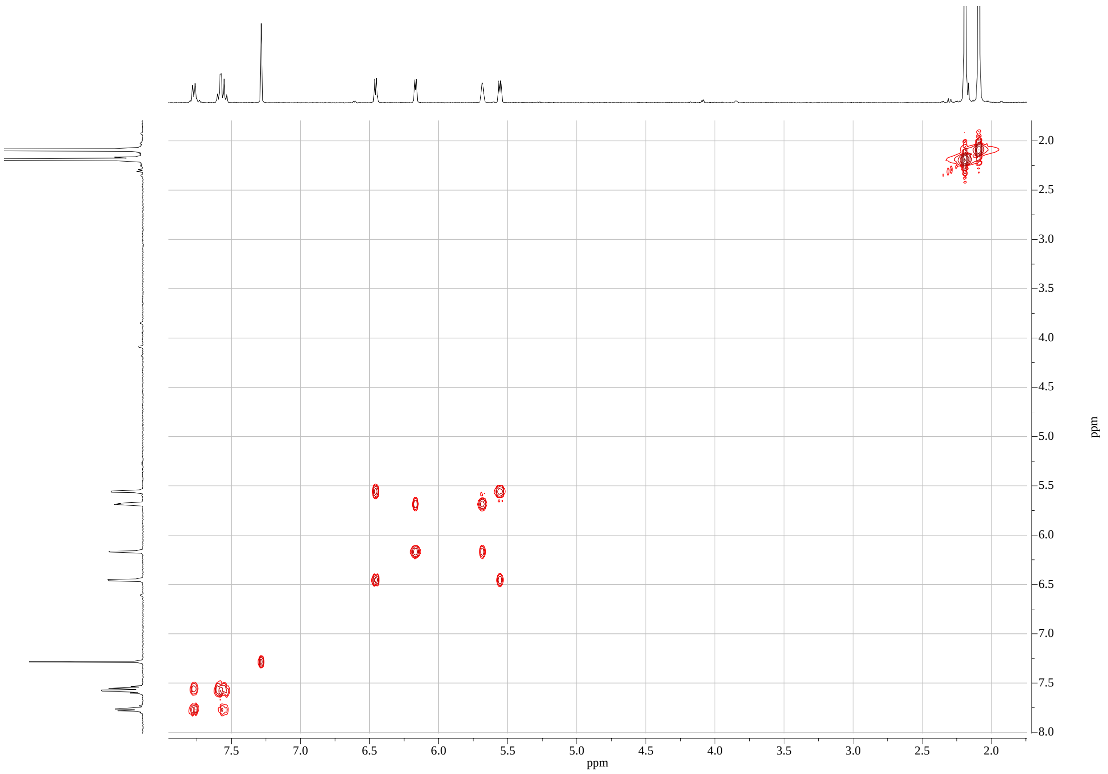

3q^{syn} - ^{1}H-^{13}C HSQCED ($CDCl_3$)

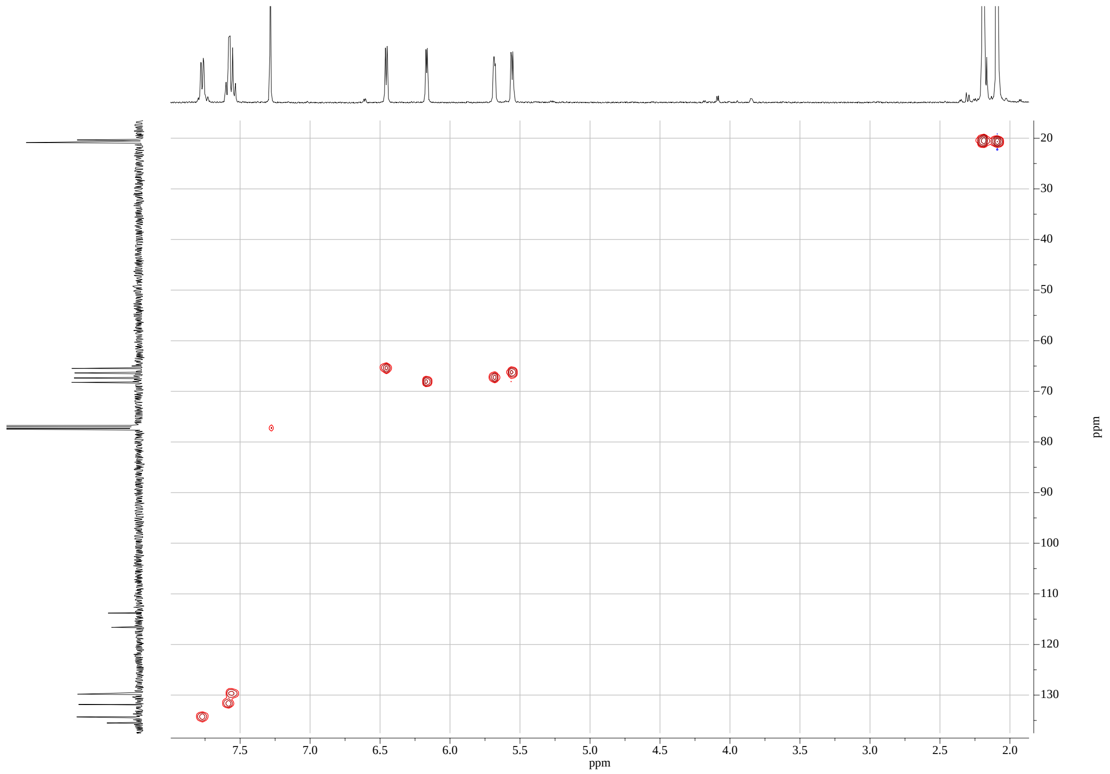

3q^{syn} - ^{1}H-^{13}C HMBC ($CDCl_3$)

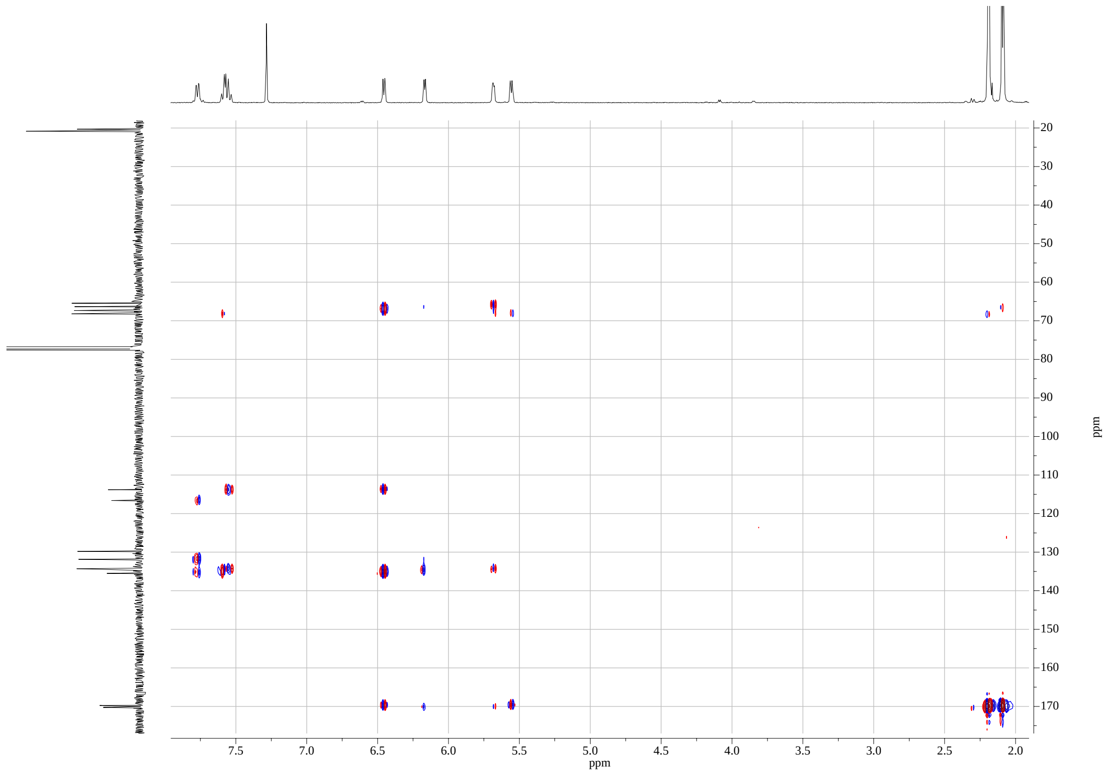

(1α,2α,3β,4β)-5-cyano-1,2,3,4-tetrahydronaphthalene-1,2,3,4-tetrayl tetraacetate (3q^{anti})

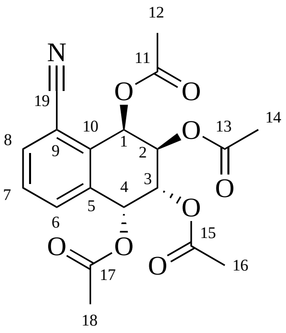

3q^{anti} - ^{1}H NMR (400 MHz, $CDCl_3$)

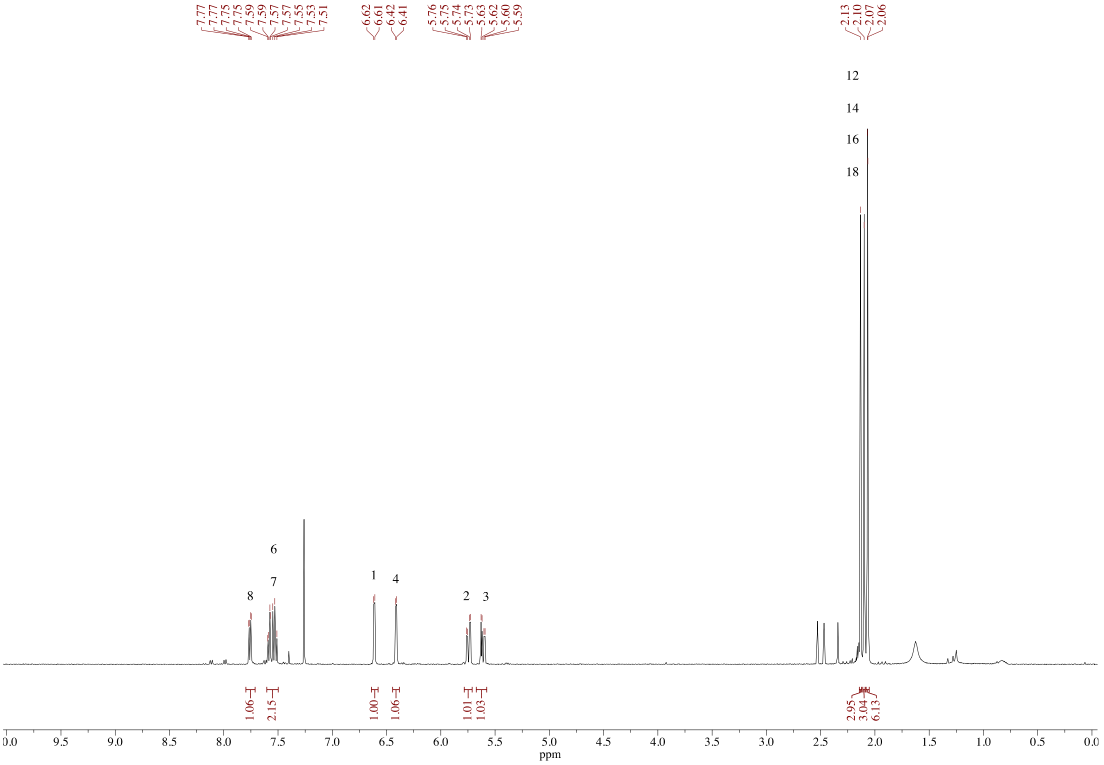

3q^{anti} - ^{13}C NMR (100 MHz, $CDCl_3$)

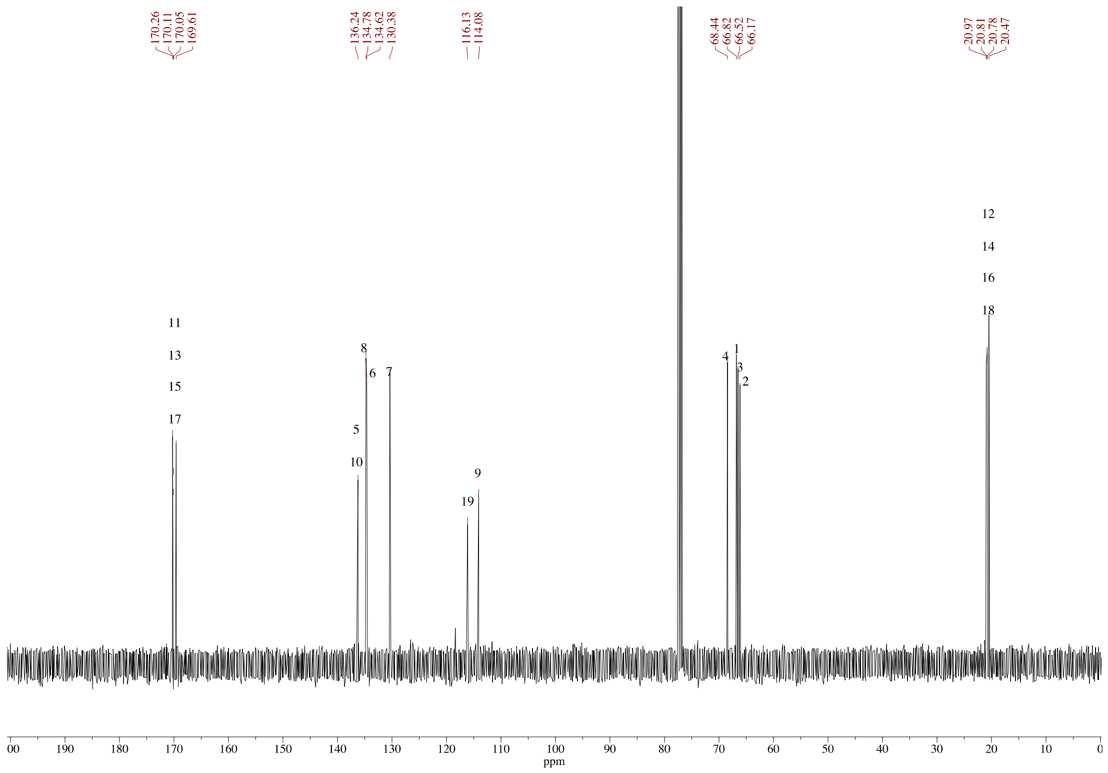

3q^{anti} - DEPT ($CDCl_3$)

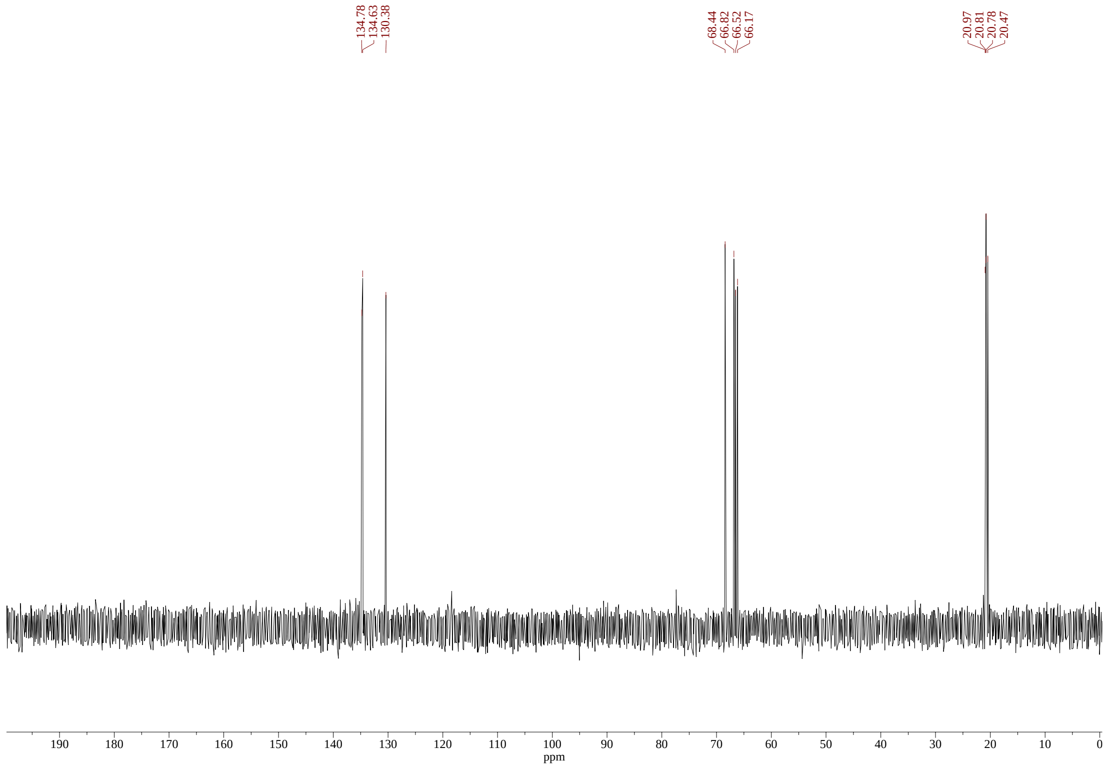

3q[anti] - DEPTQ ($CDCl_3$)

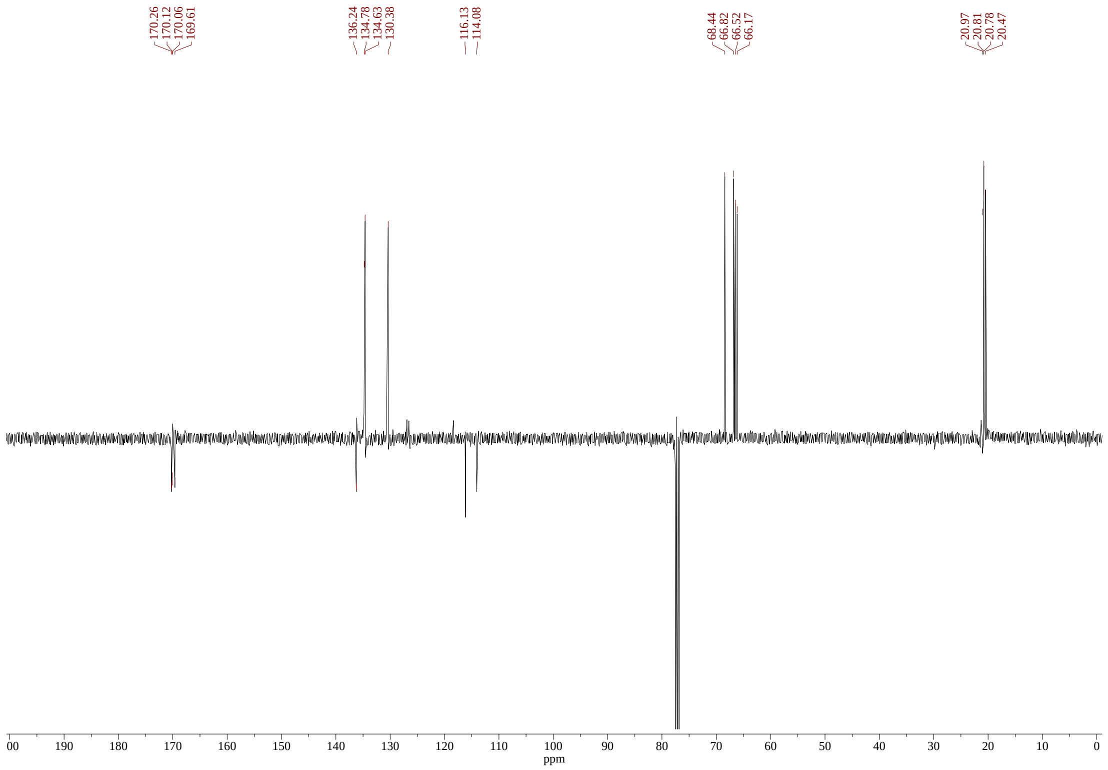

3q[anti] - ^1H-^1H COSY ($CDCl_3$)

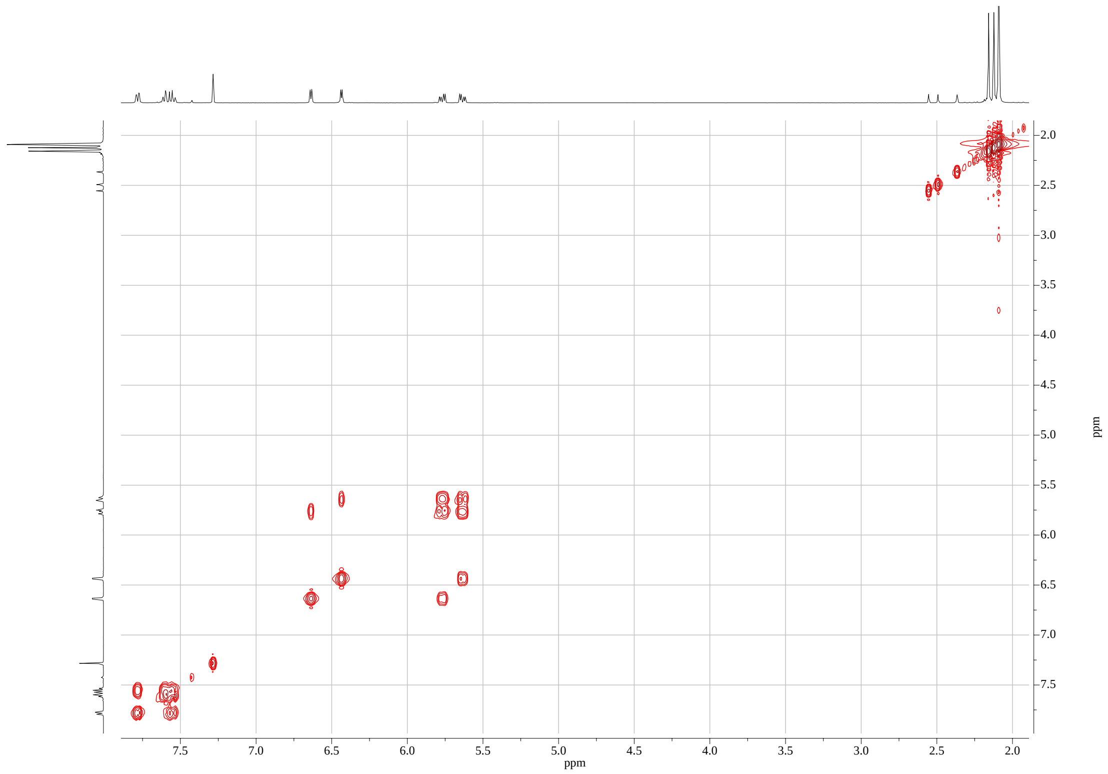

3q^anti - ^{1}H-^{13}C HSQCED ($CDCl_3$)

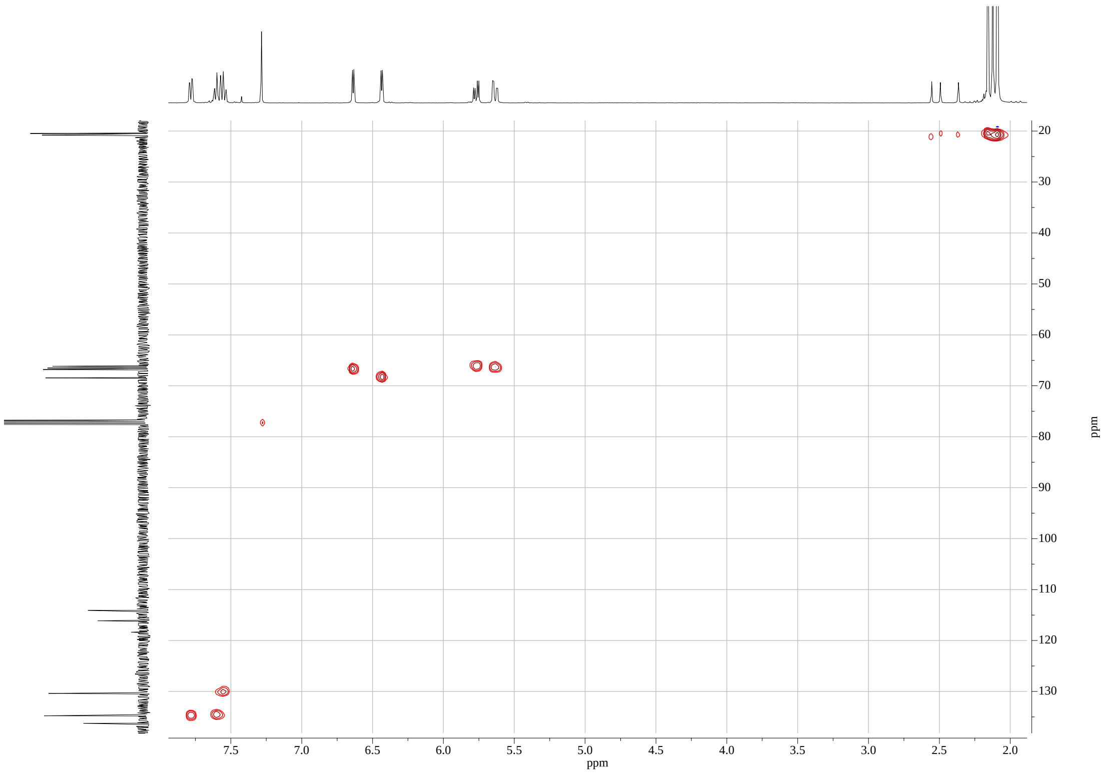

3q^anti - ^{1}H-^{13}C HMBC ($CDCl_3$)

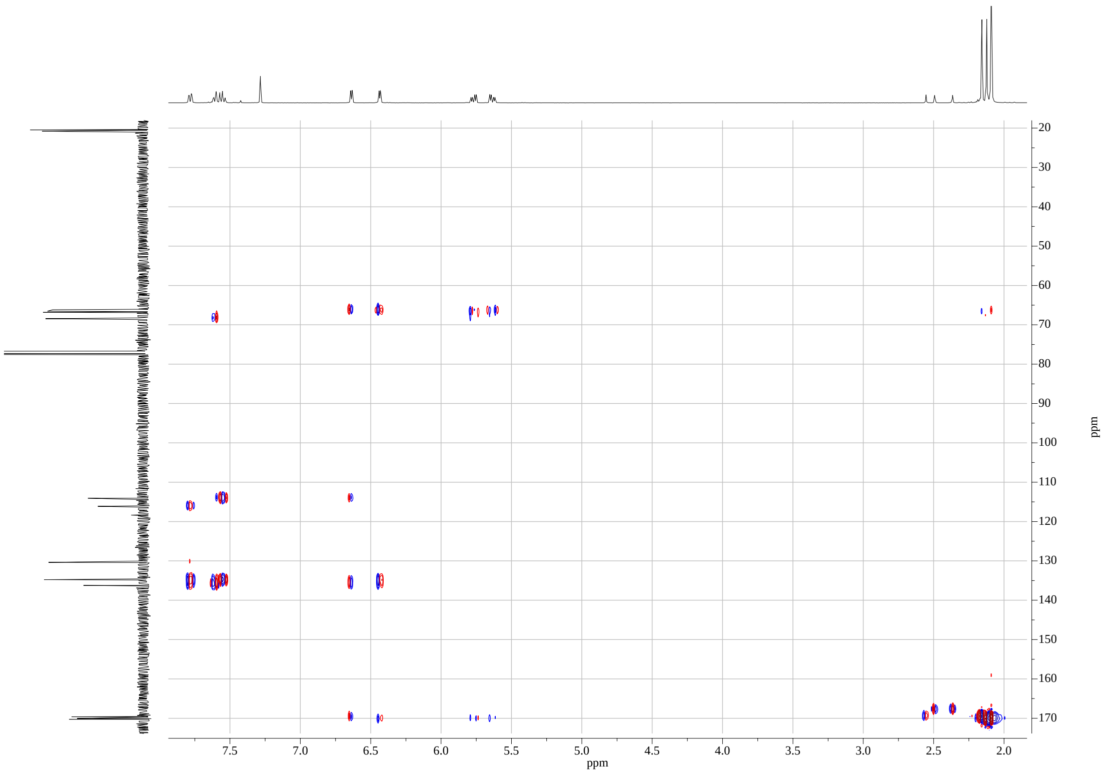

(1α,2α,3α,4α)-6-phenyl-1,2,3,4-tetrahydronaphthalene-1,2,3,4-tetrayl tetraacetate ($3h^{syn}$)

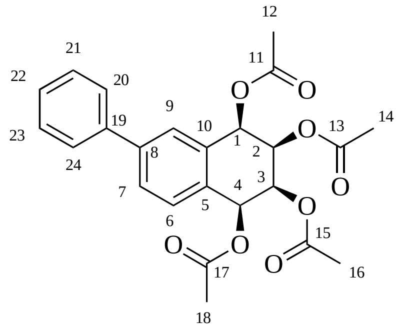

$3h^{syn}$ - ^{1}H NMR (400 MHz, $CDCl_3$)

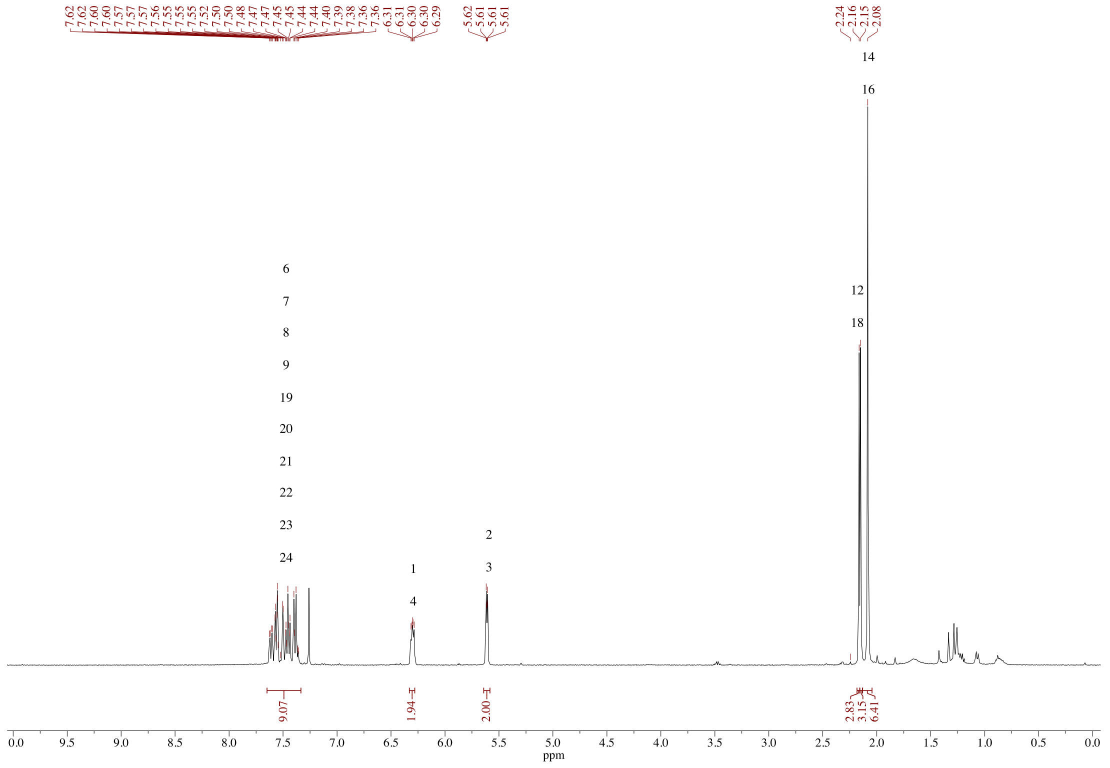

3h^{syn} - ^{13}C NMR (100 MHz, $CDCl_3$)

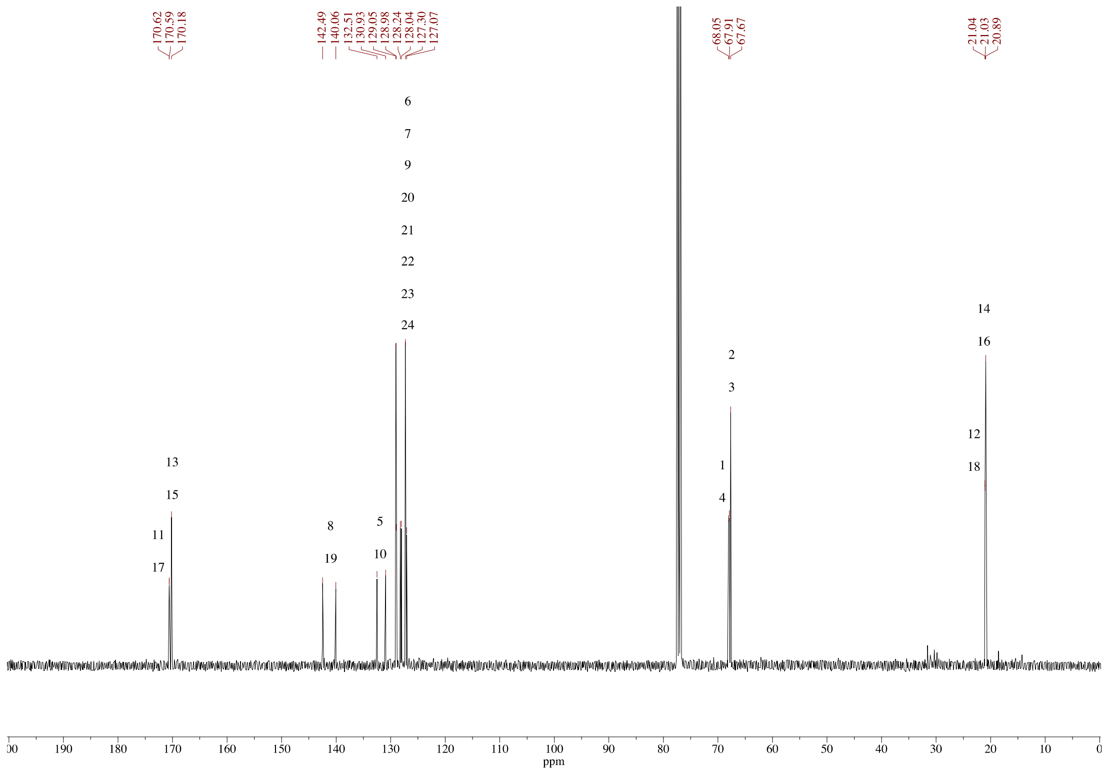

3h^{syn} - DEPT ($CDCl_3$)

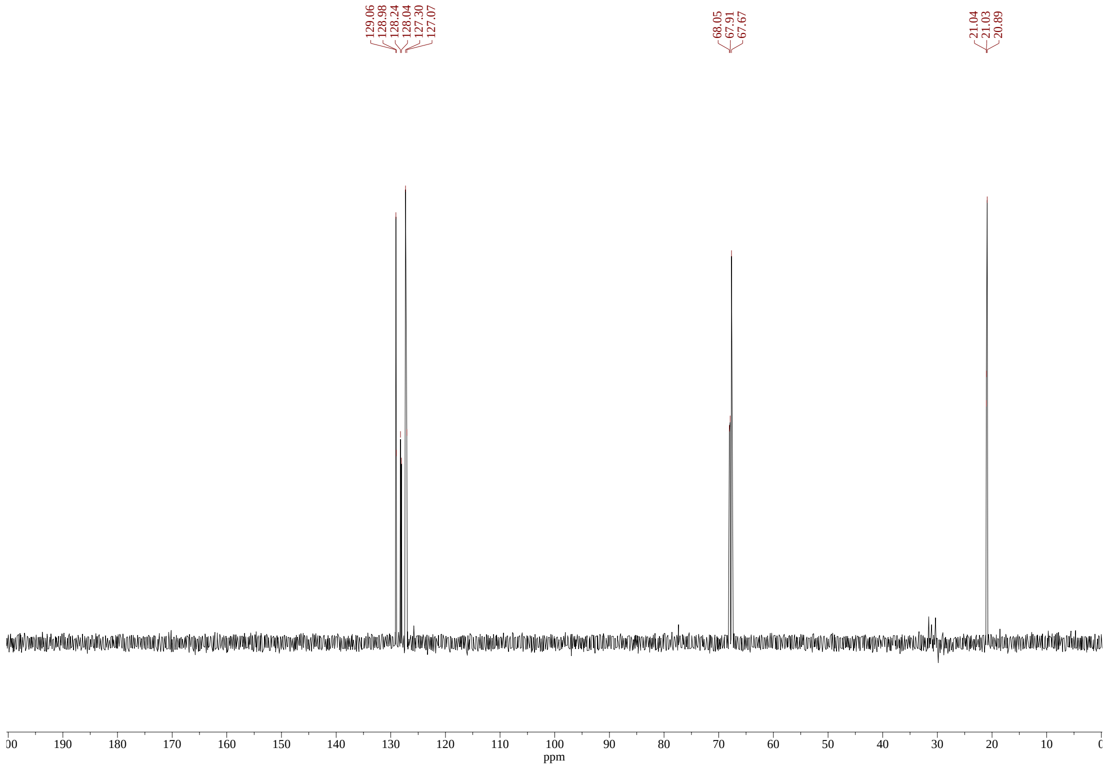

3h^syn - DEPTQ ($CDCl_3$)

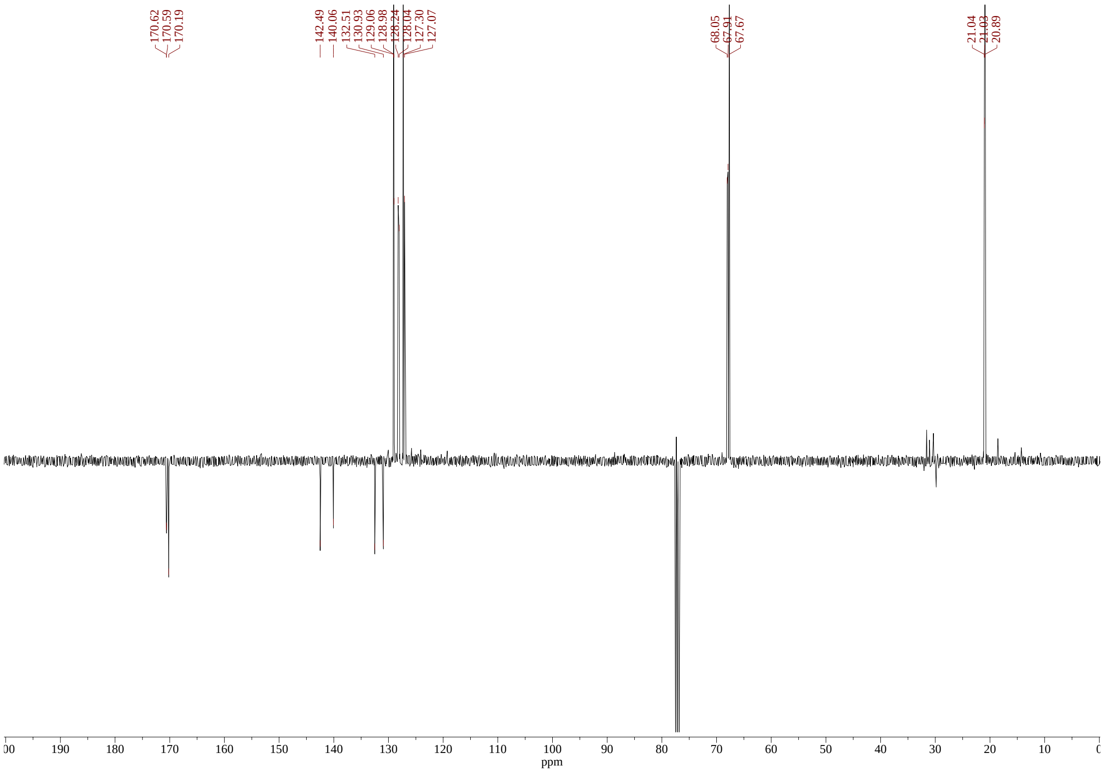

3h^syn - ^1H-^1H COSY ($CDCl_3$)

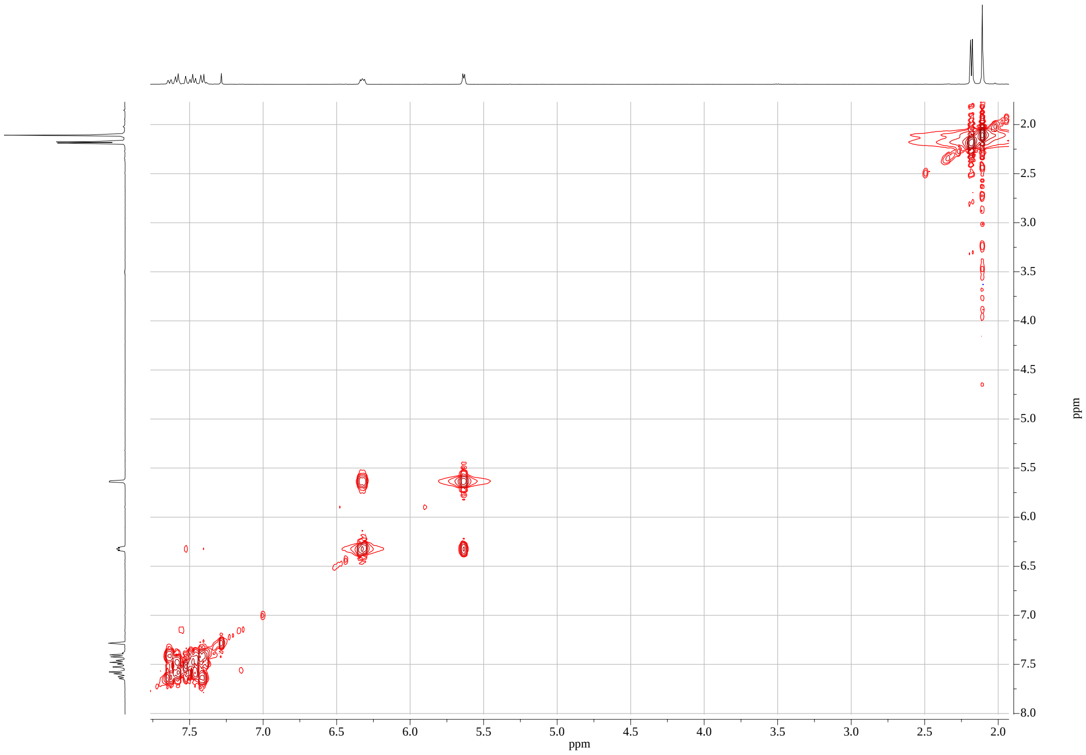

3h^{syn} - ^{1}H-^{13}C HSQCED (CDCl$_3$)

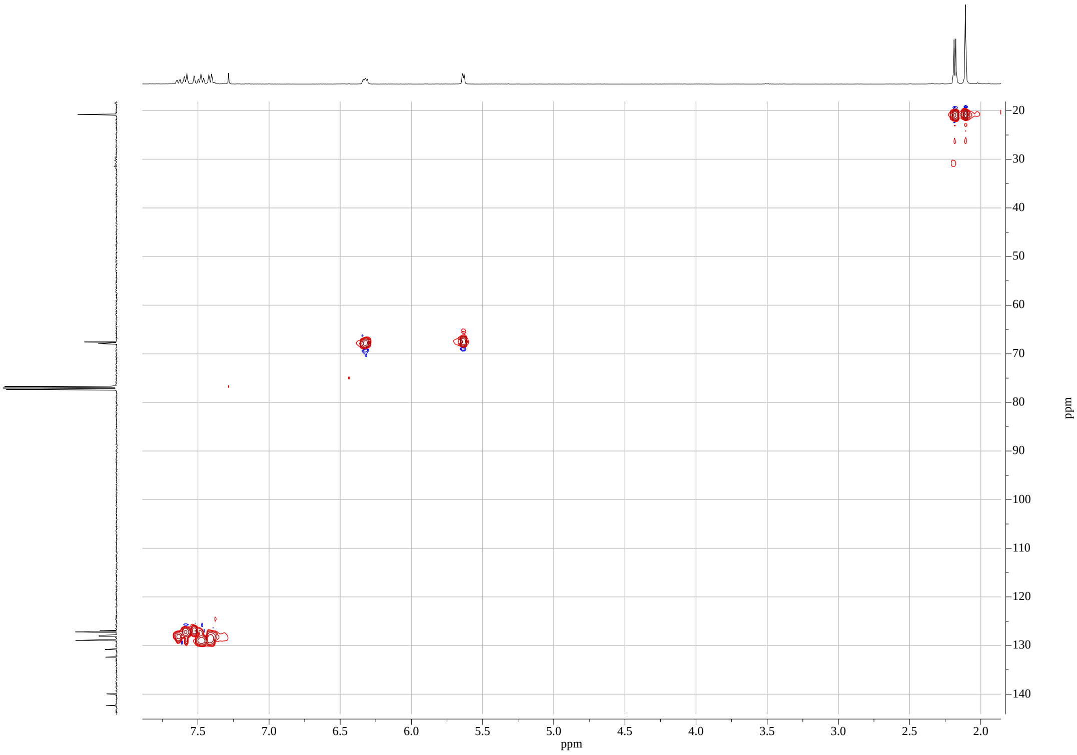

3h^{syn} - ^{1}H-^{13}C HMBC (CDCl$_3$)

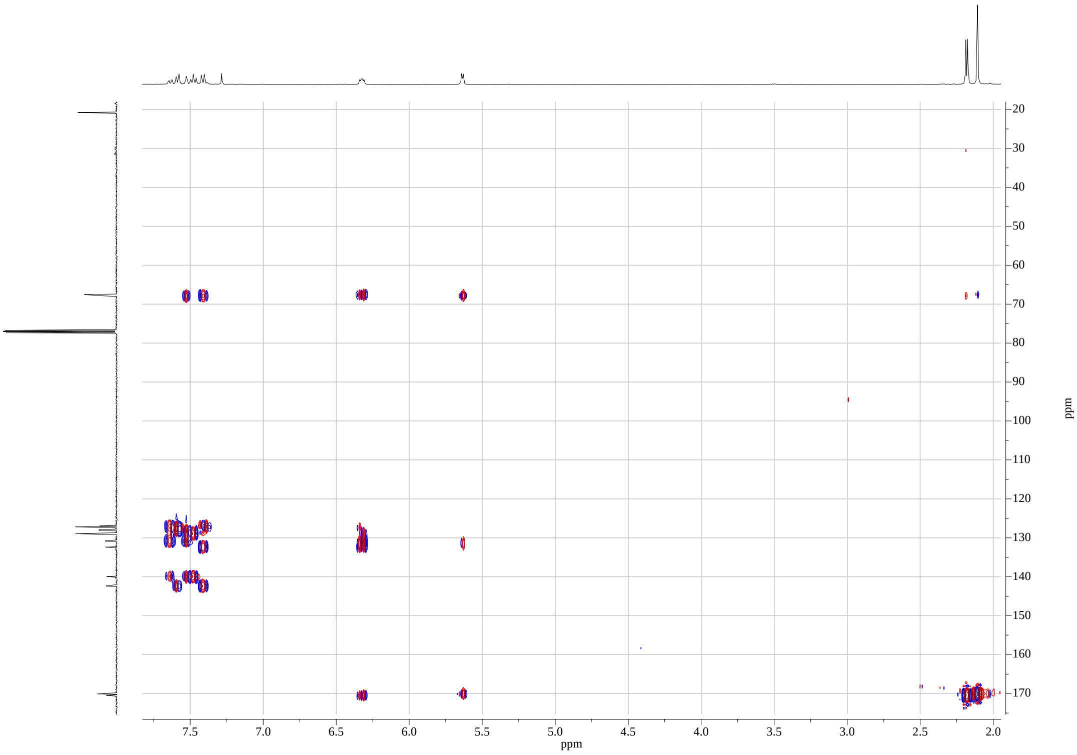

(1α,2α,3β,4β)-6-phenyl-1,2,3,4-tetrahydronaphthalene-1,2,3,4-tetrayl tetraacetate (3h^anti)

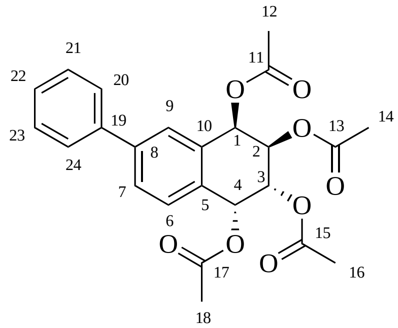

Mixture of cis and trans isomers

3h^anti - ^{1}H NMR (400 MHz, $CDCl_3$)

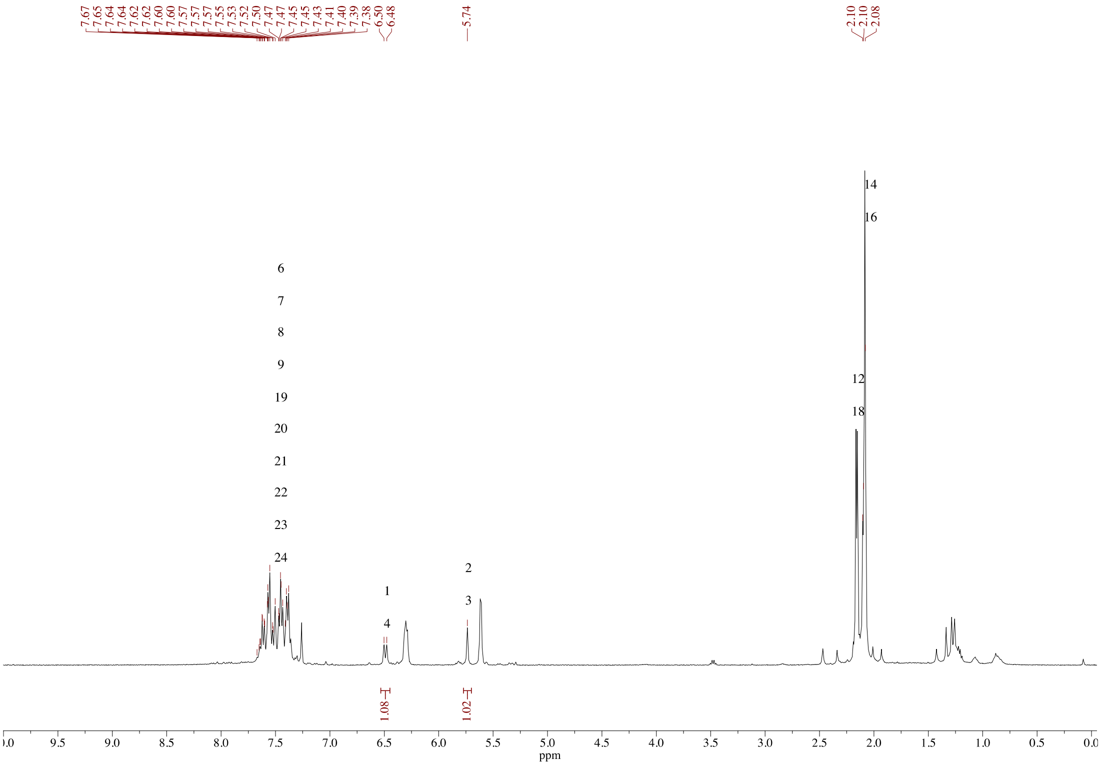

3h[anti] - ^{13}C NMR (100 MHz, $CDCl_3$)

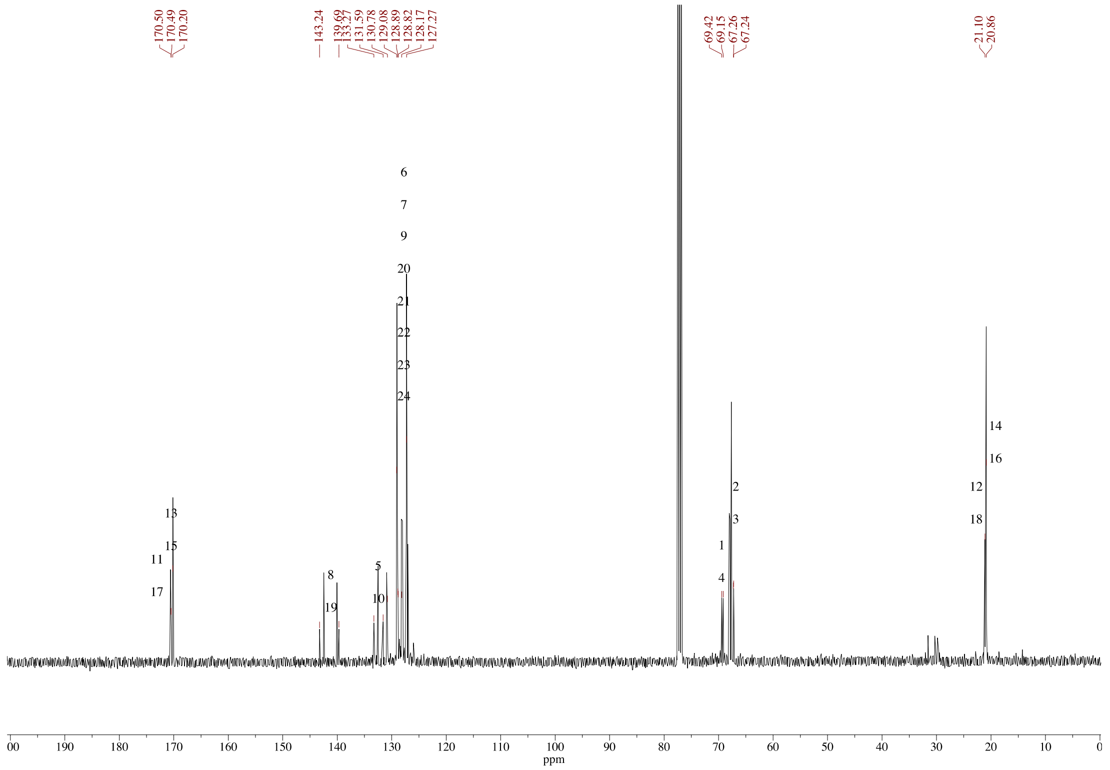

3h[anti] - DEPT ($CDCl_3$)

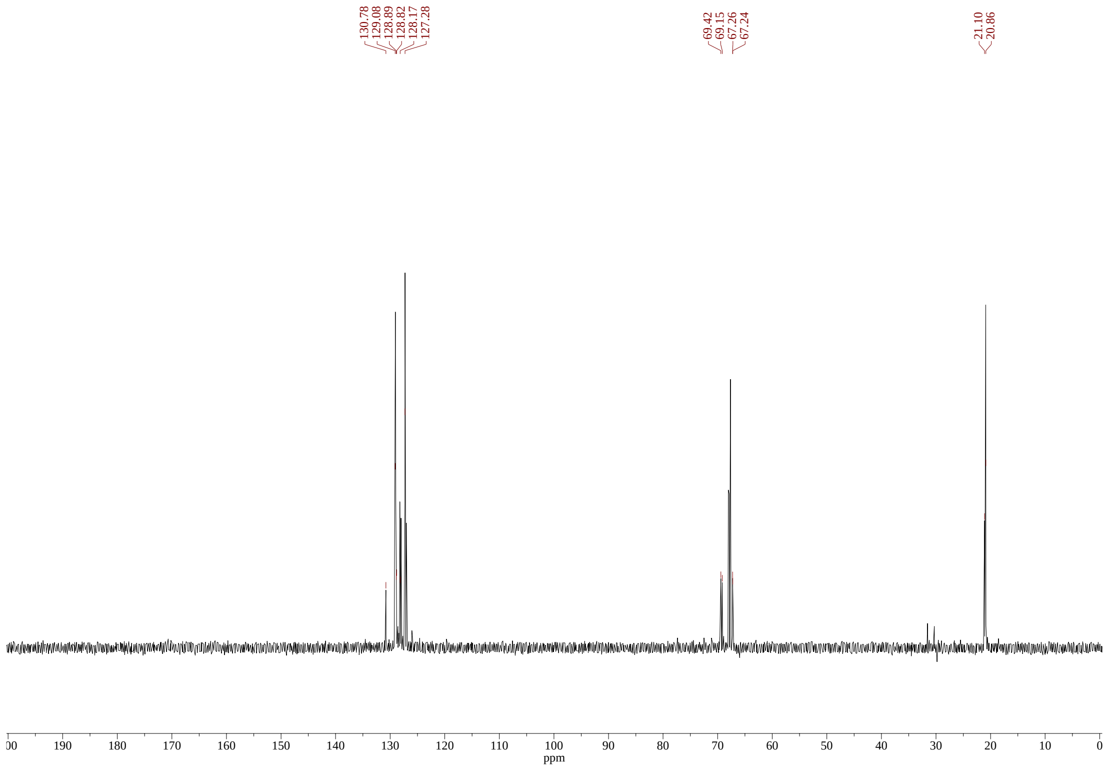

3h^anti - DEPTQ ($CDCl_3$)

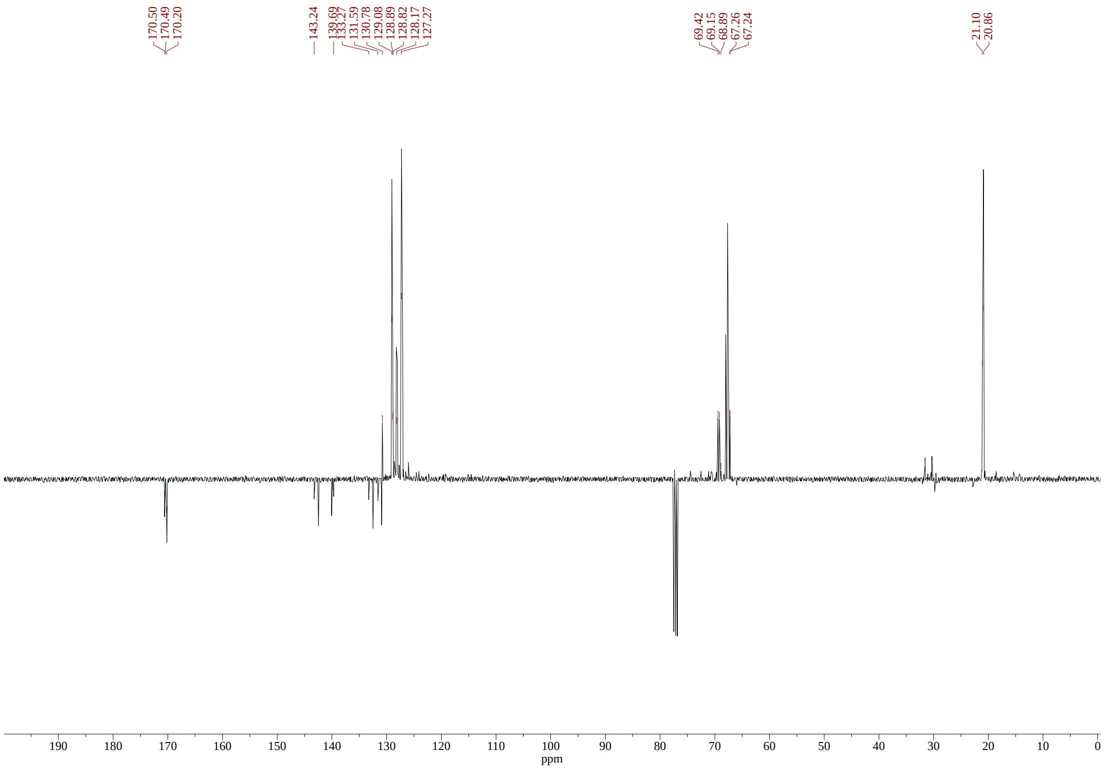

3h^anti - ^1H-^1H COSY ($CDCl_3$)

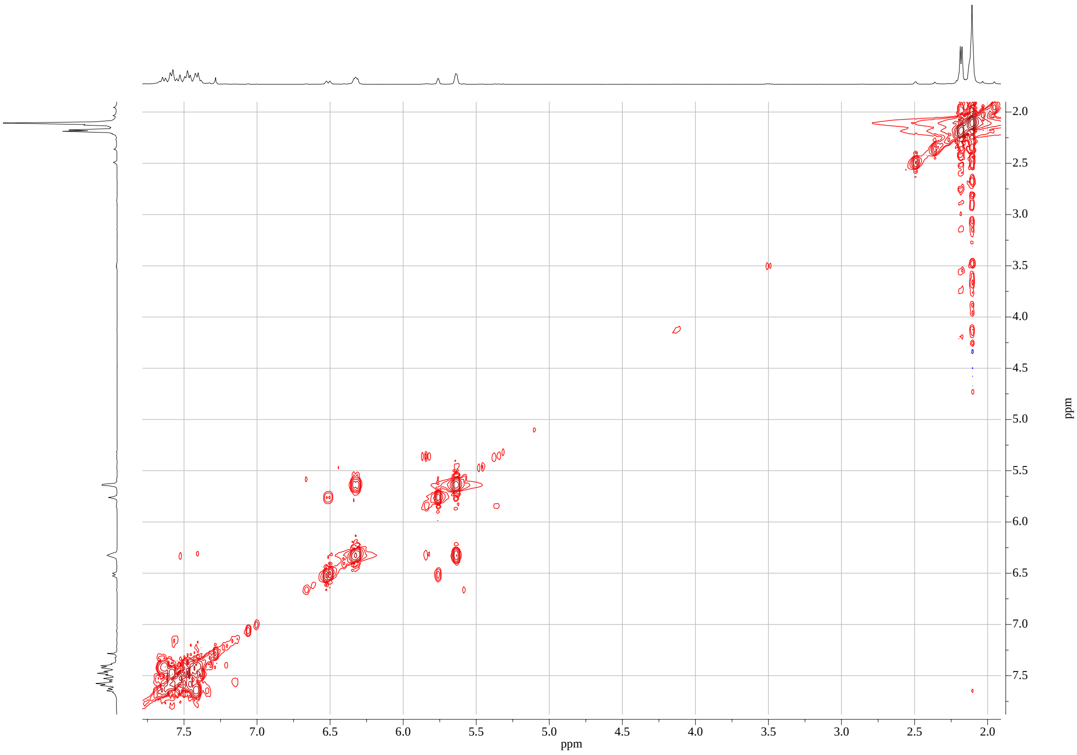

3h^{anti} - ^{1}H-^{13}C HSQCED (CDCl$_3$)

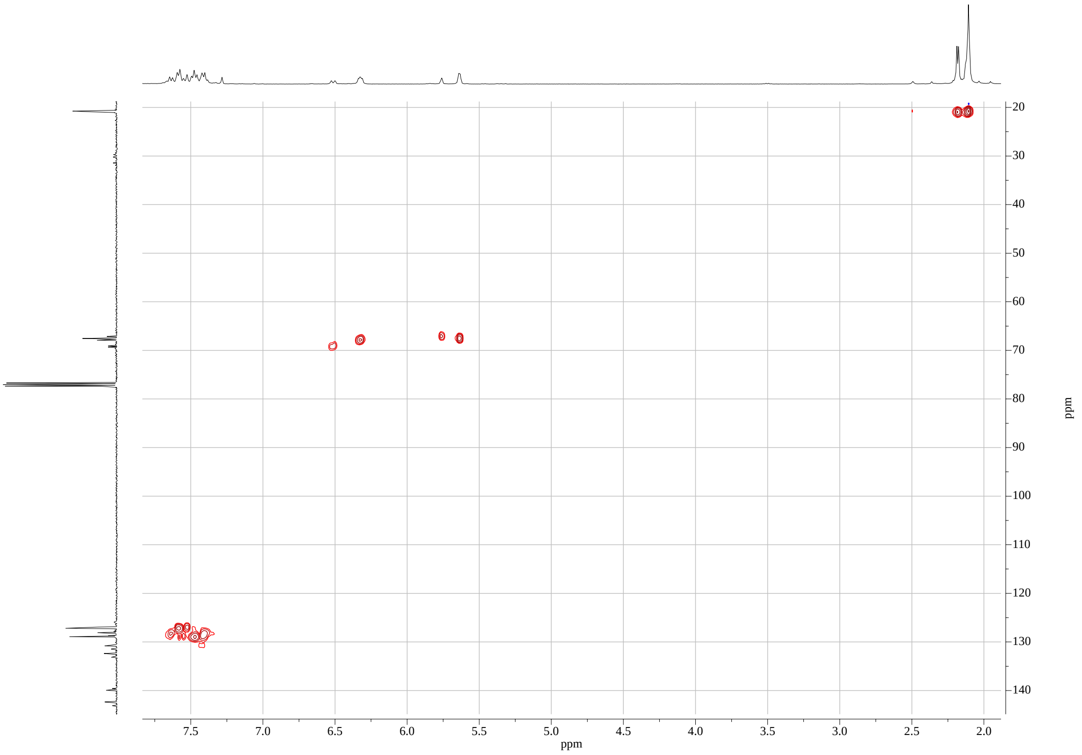

3h^{anti} - ^{1}H-^{13}C HMBC (CDCl$_3$)

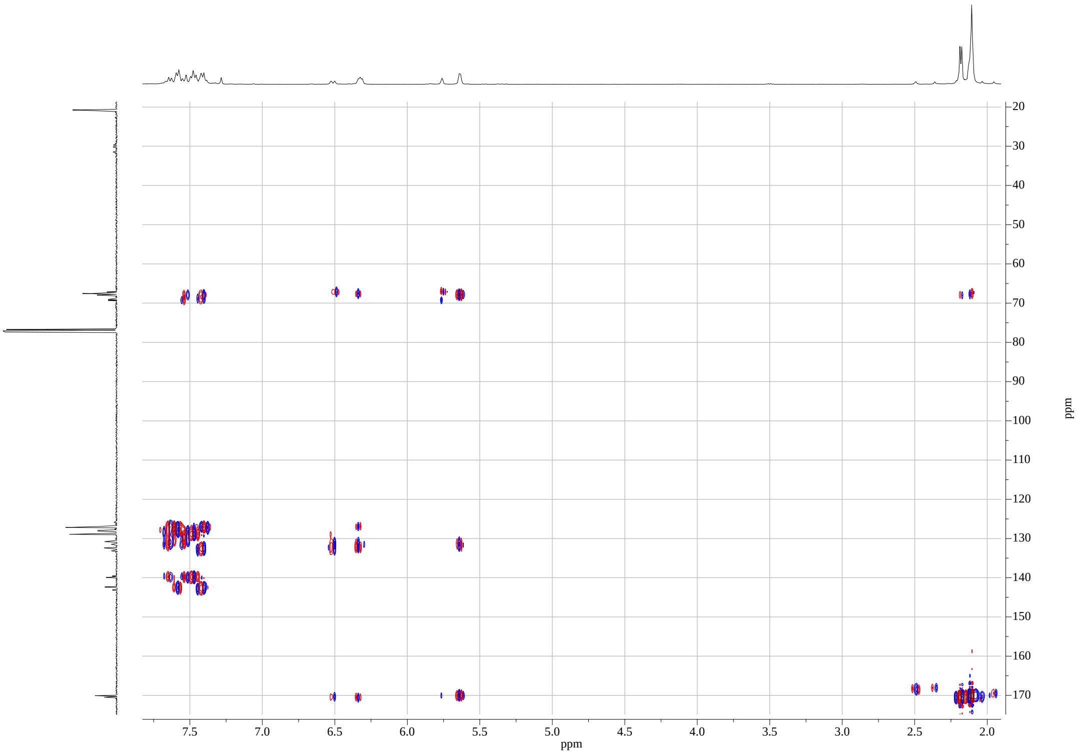

(1α,2α,3α,4α)-5,8-dicyano-1,2,3,4-tetrahydronaphthalene-1,2,3,4-tetrayl tetraacetate (3r^{syn})

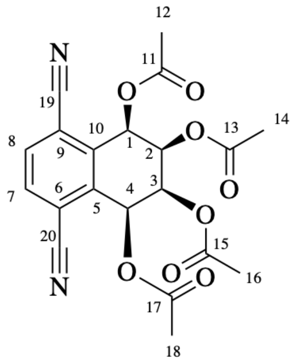

3r^{syn} - ^{1}H NMR (400 MHz, $CDCl_3$)

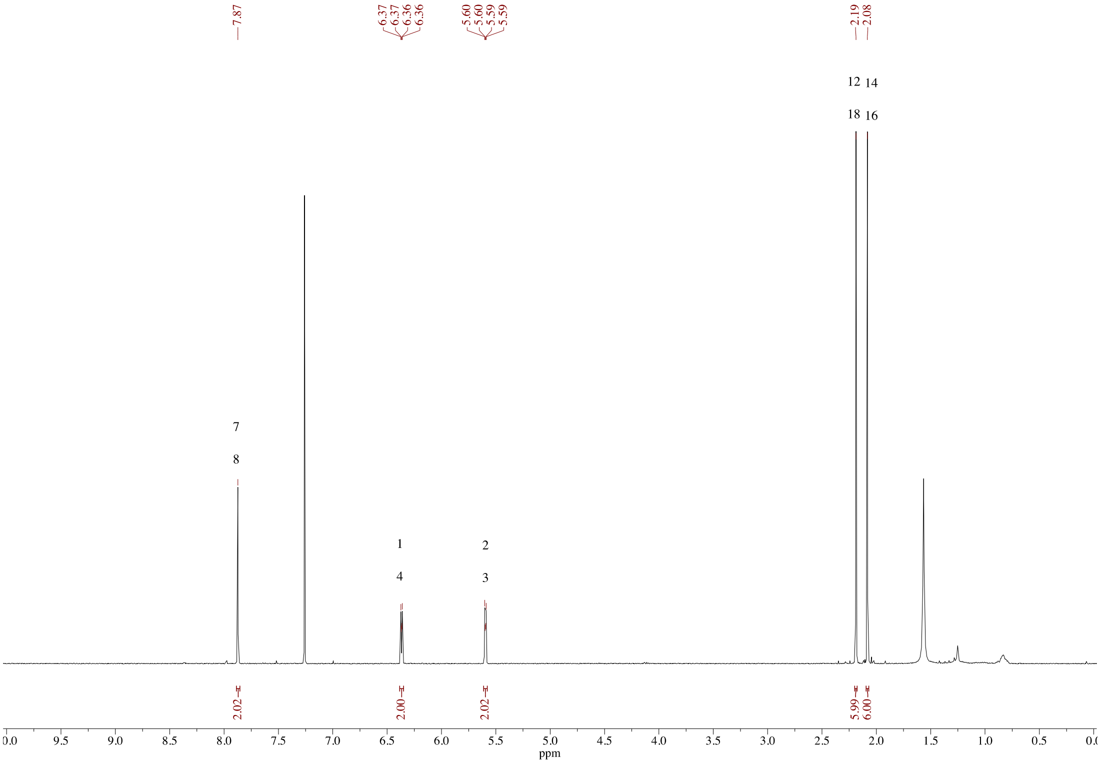

3r^{syn} - ^{13}C NMR (100 MHz, $CDCl_3$)

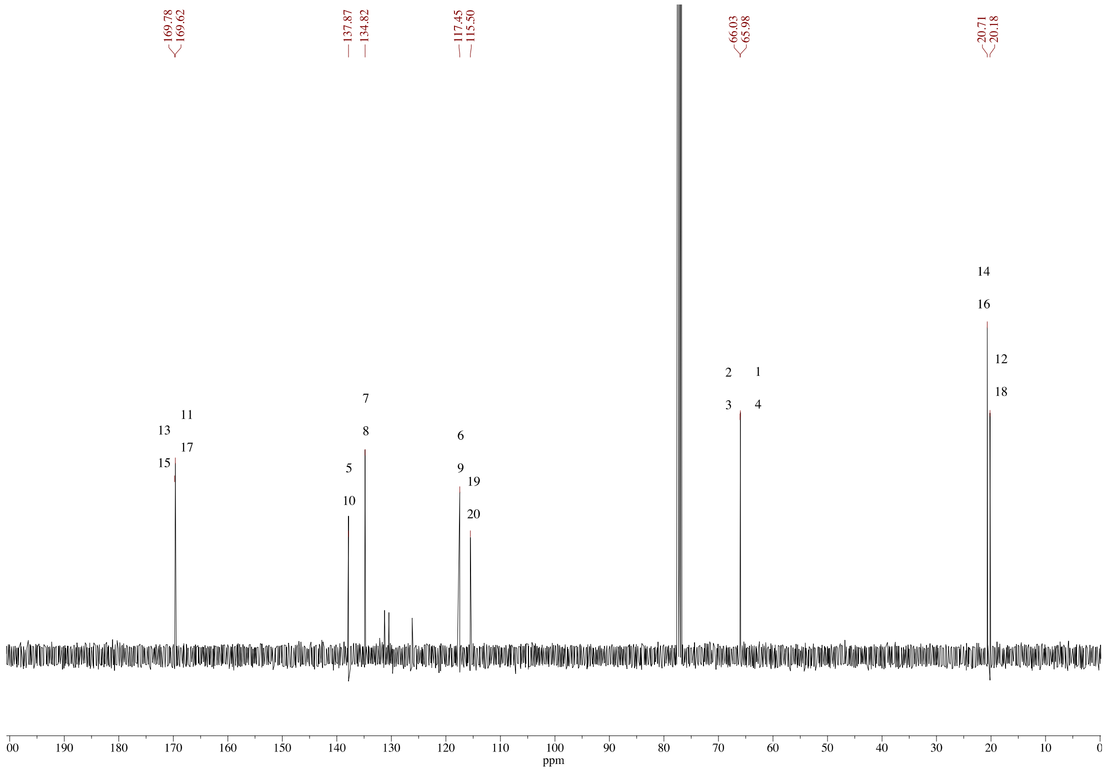

3r^{syn} - DEPT ($CDCl_3$)

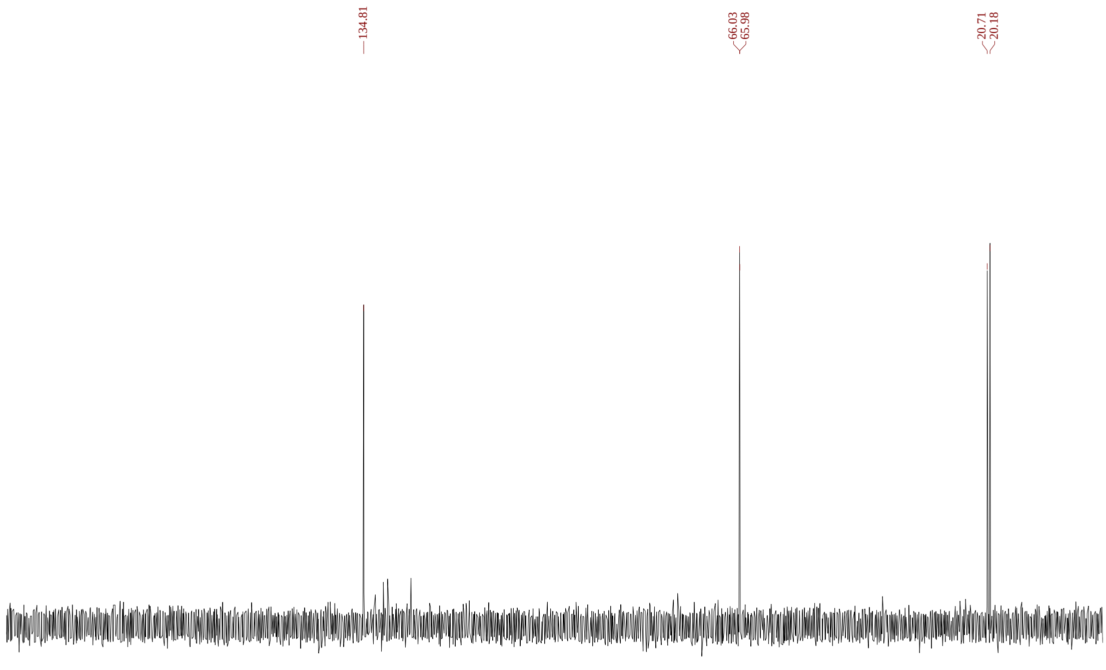

3r^{syn} - DEPTQ ($CDCl_3$)

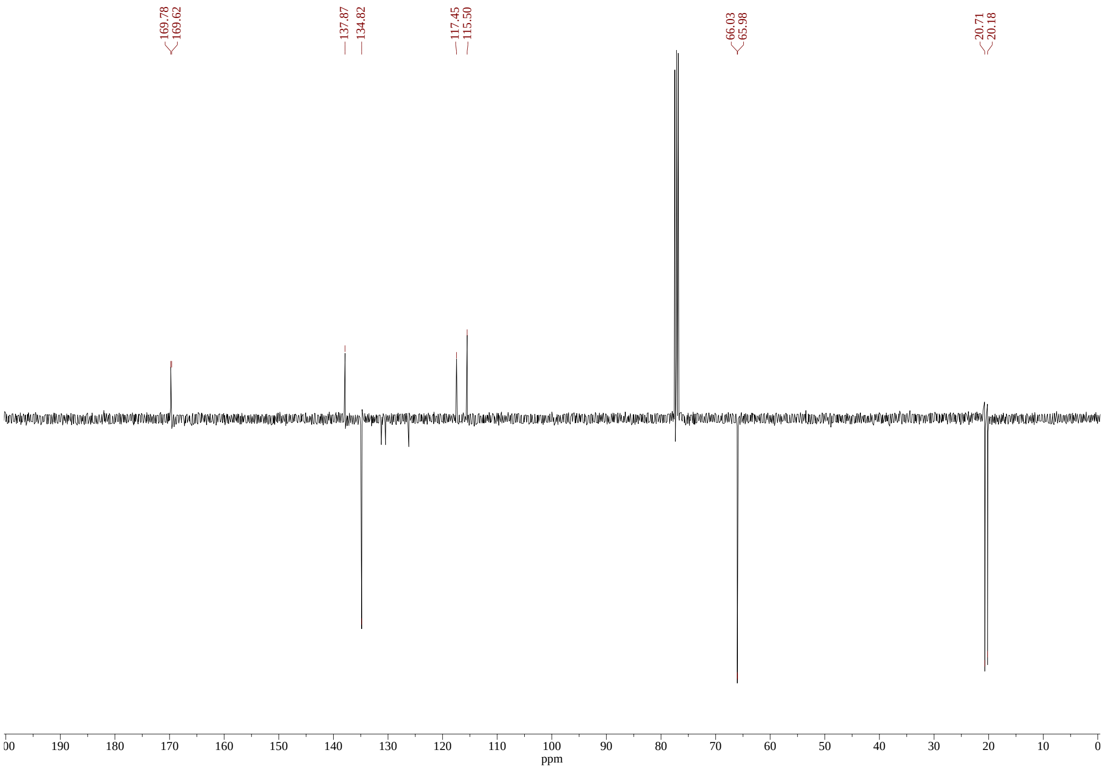

3r^{syn} - ^{1}H-^{1}H COSY ($CDCl_3$)

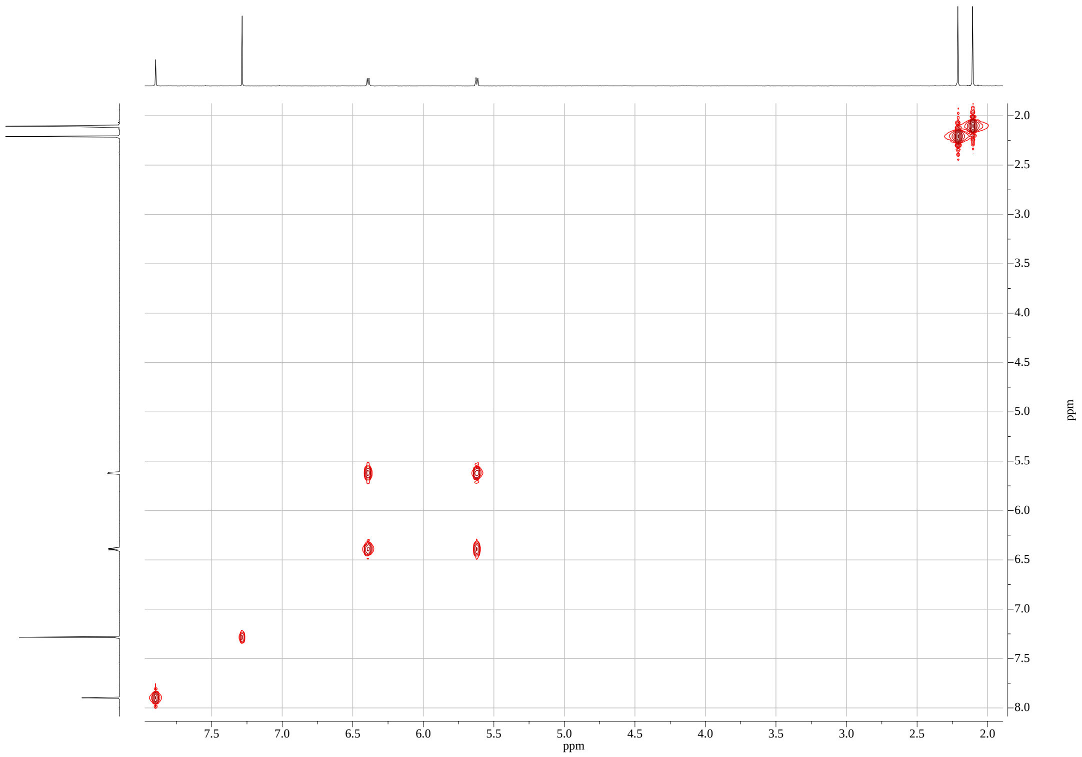

3r^{syn} - ^{1}H-^{13}C HSQCED ($CDCl_3$)

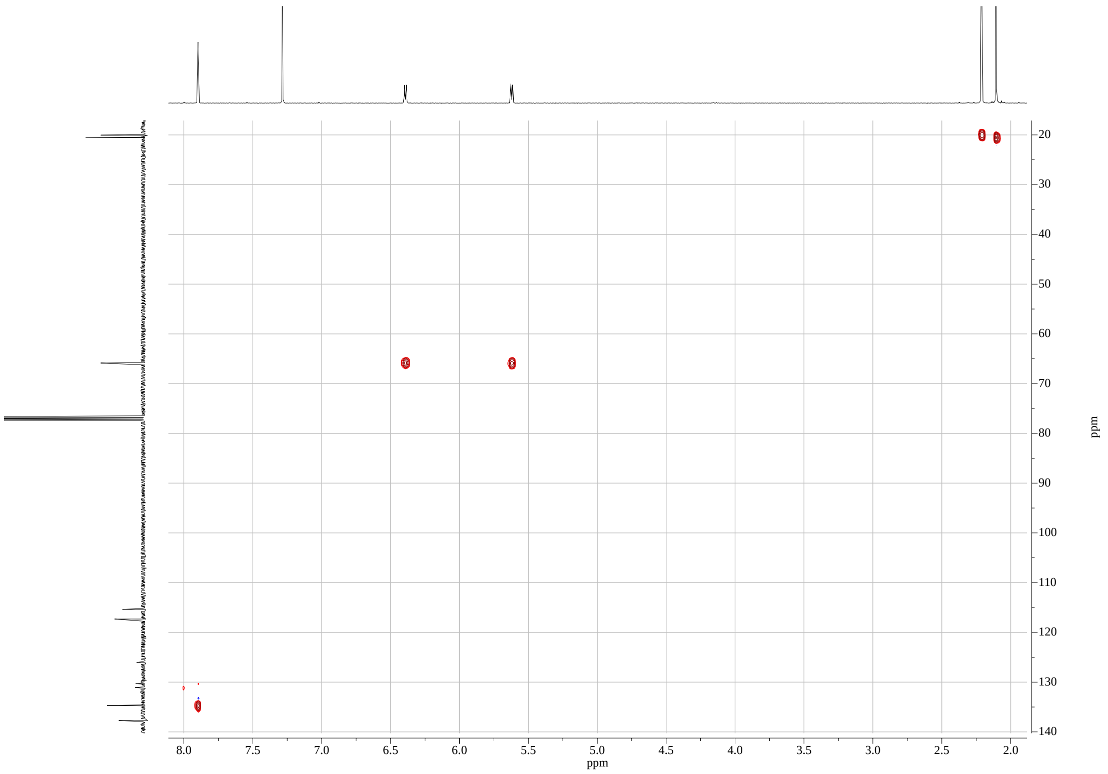

3r^{syn} - ^{1}H-^{13}C HMBC ($CDCl_3$)

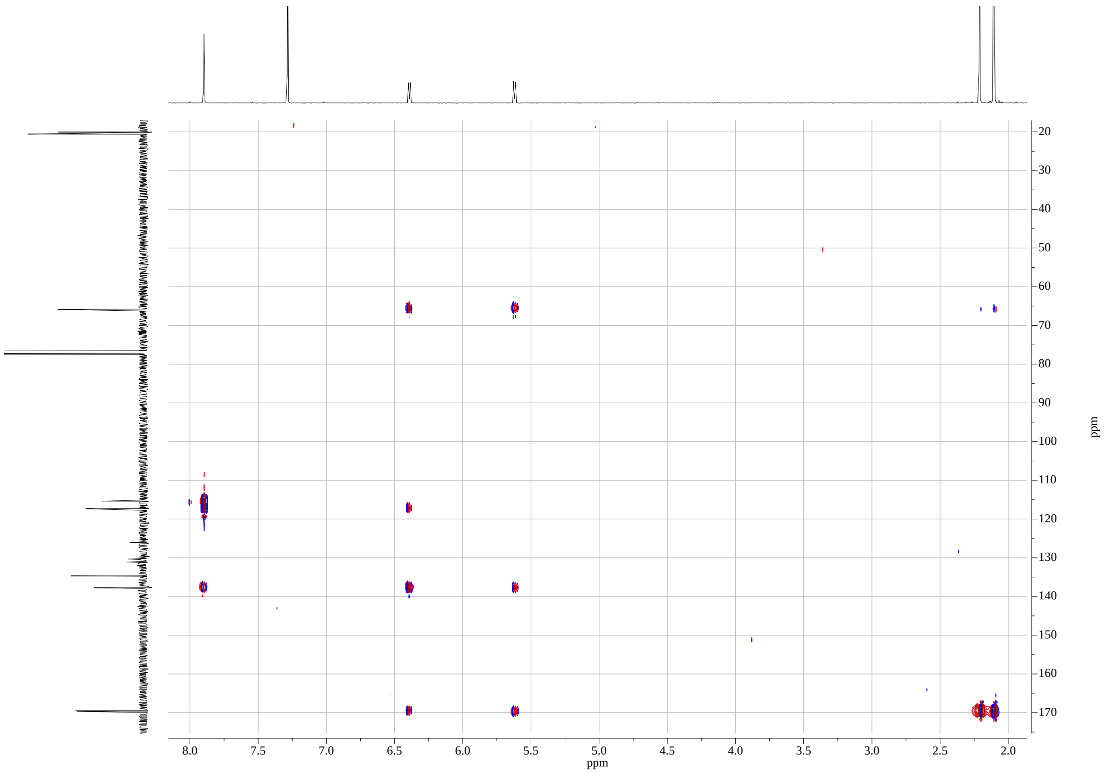

(1α,2α,3α,4α)-6-bromo-1,2,3,4-tetrahydronaphthalene-1,2,3,4-tetrayl tetraacetate ($3l^{syn}$)

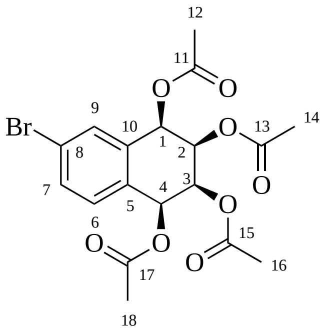

$3l^{syn}$ - ^{1}H NMR (400 MHz, $CDCl_3$)

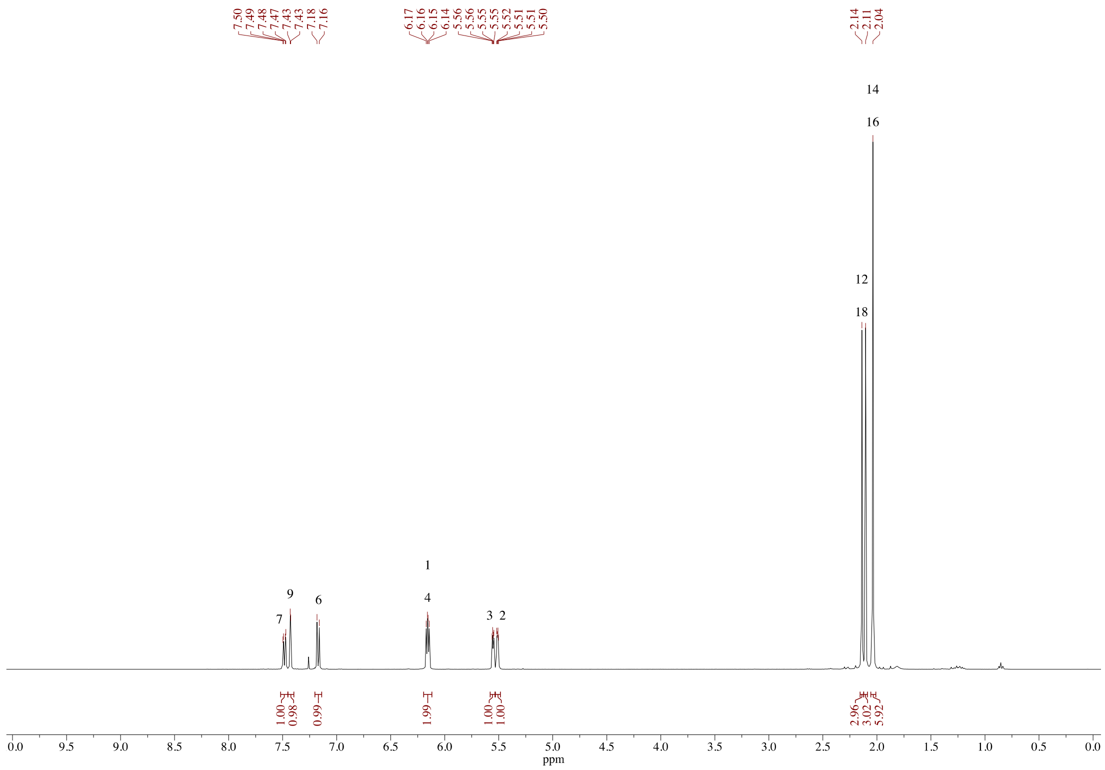

3l^{syn} - ^{13}C NMR (100 MHz, $CDCl_3$)

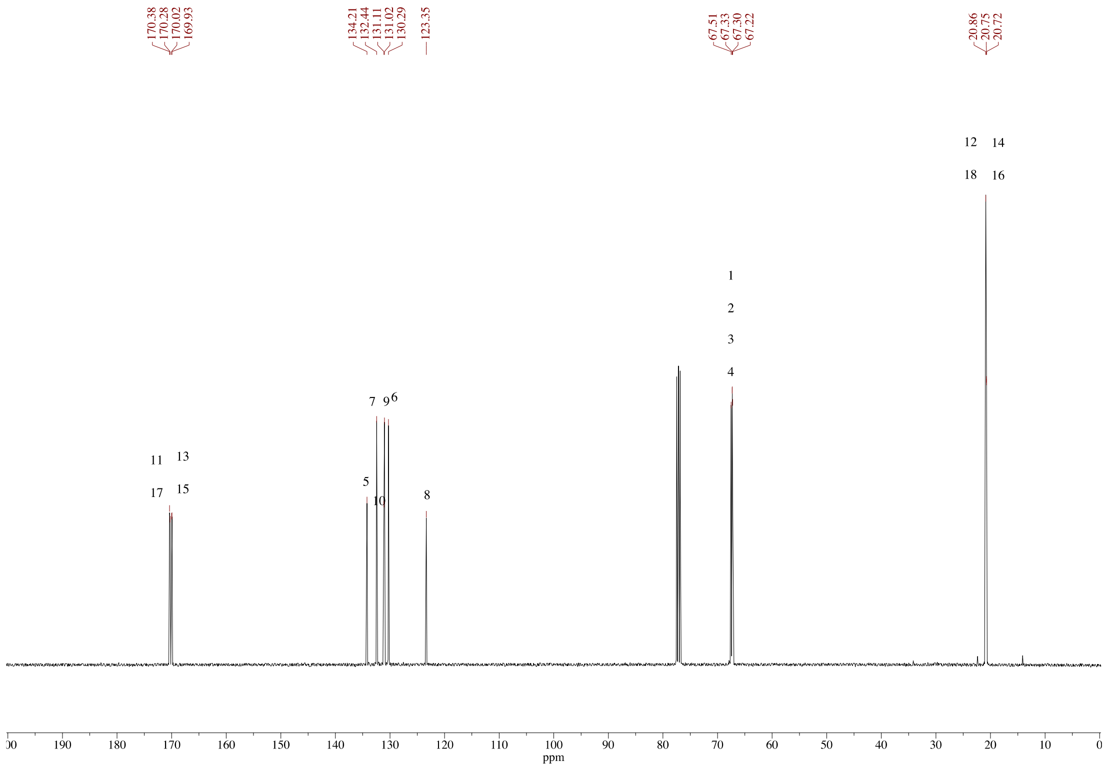

3l^{syn} - DEPT ($CDCl_3$)

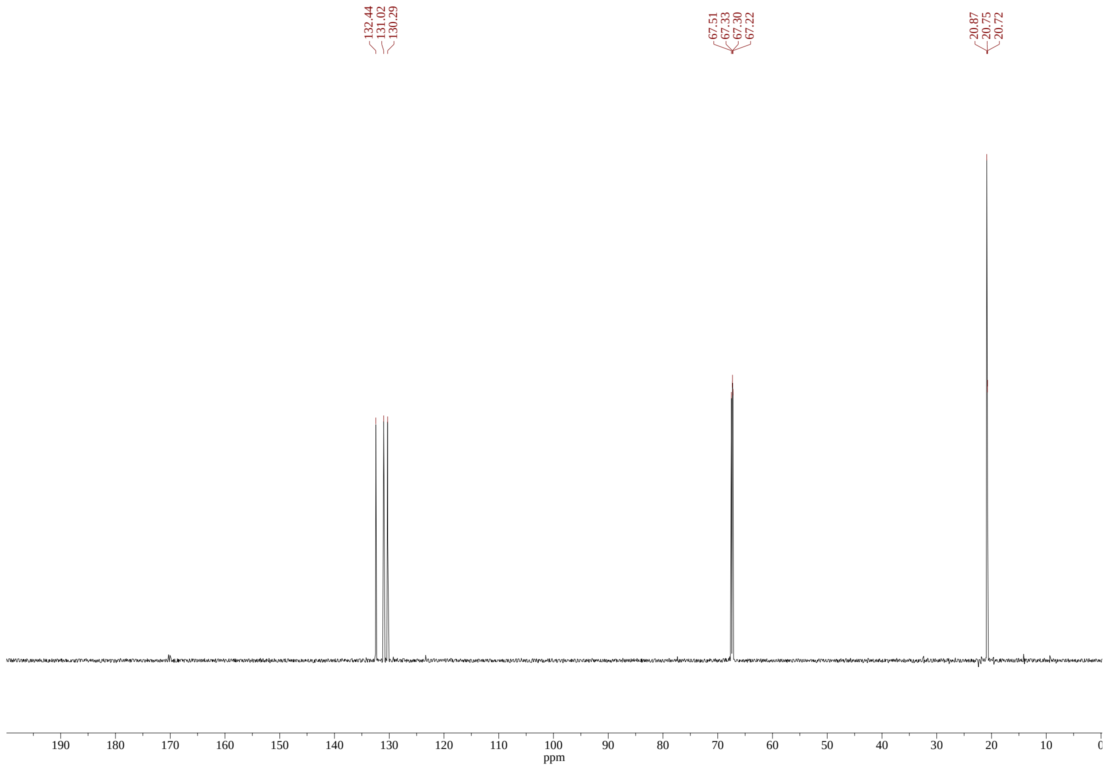

3l^{syn} - DEPTQ ($CDCl_3$)

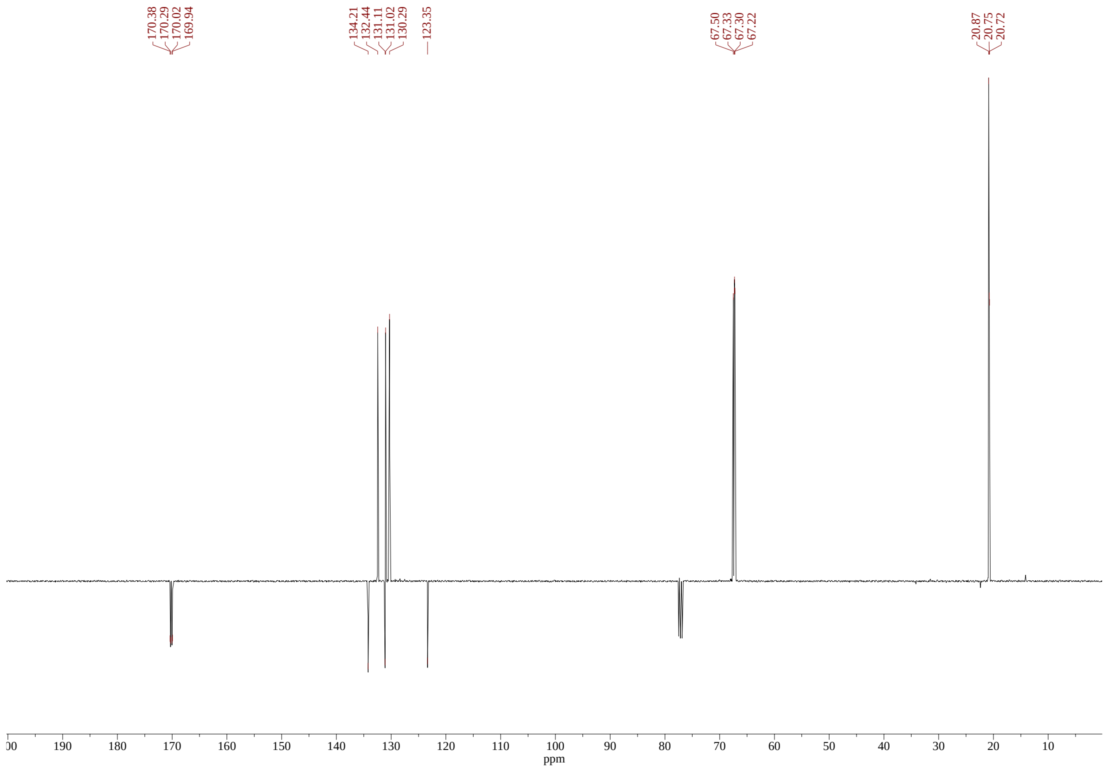

3l^{syn} - ^{1}H-^{1}H COSY ($CDCl_3$)

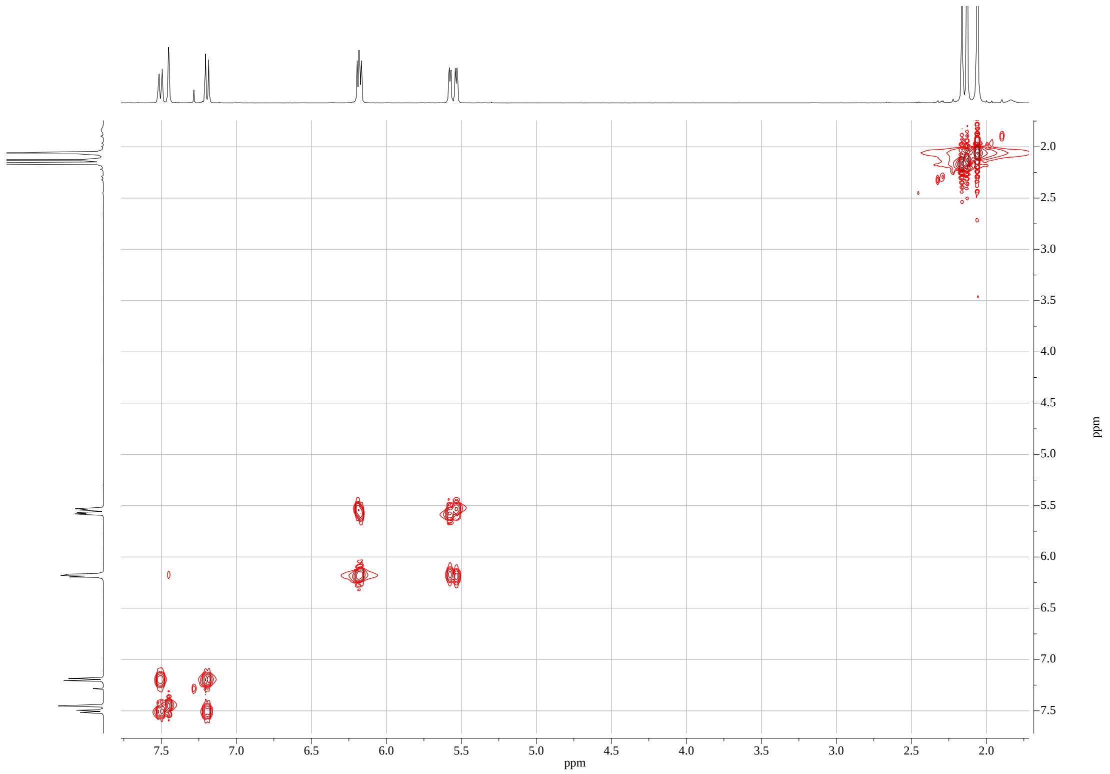

3l^{syn} - ^{1}H-^{13}C HSQCED ($CDCl_3$)

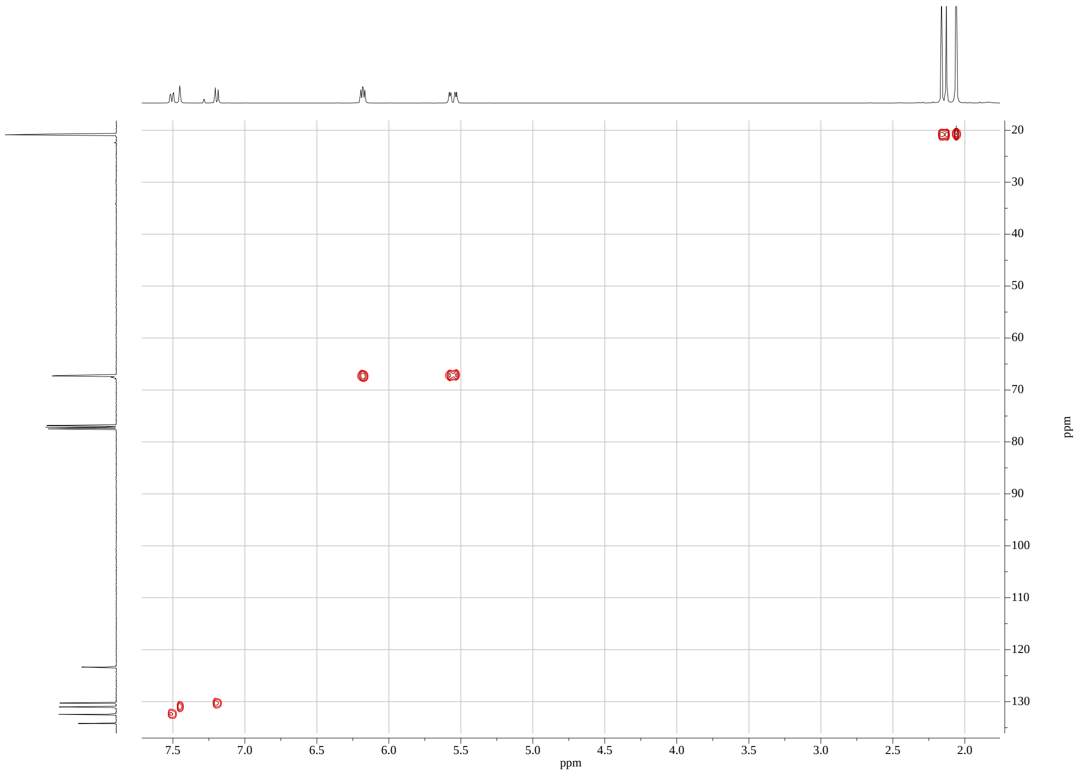

3l^{syn} - ^{1}H-^{13}C HMBC ($CDCl_3$)

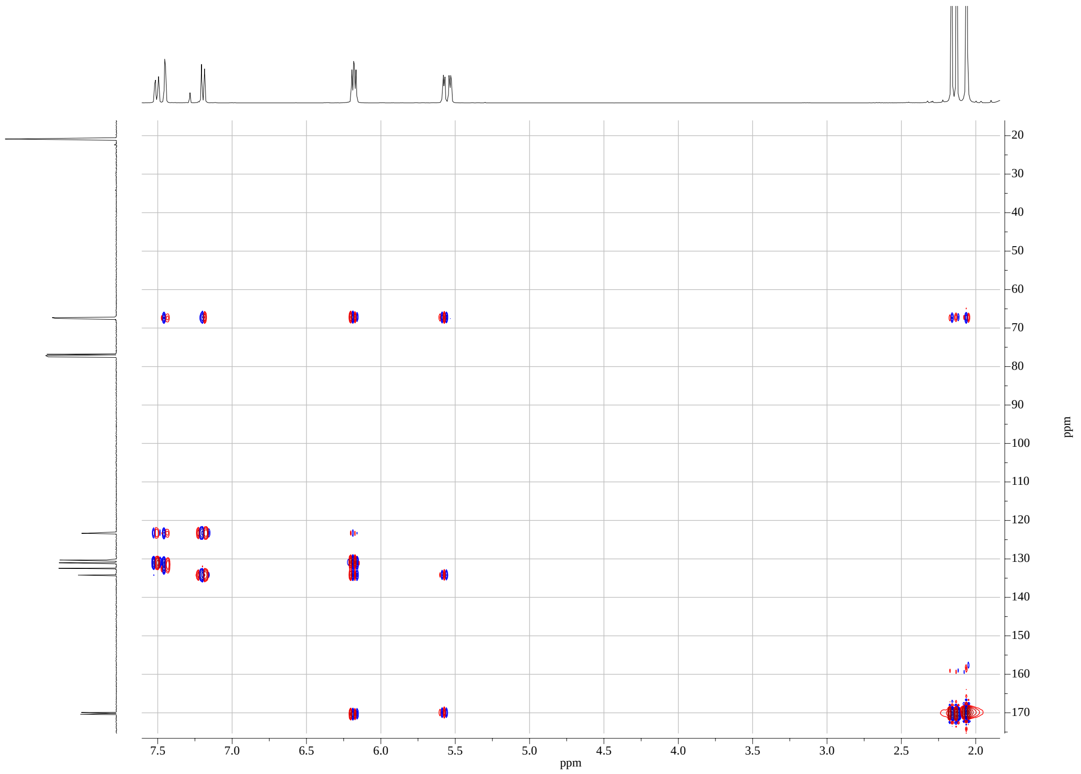

(1α,2α,3β,4β)-6-bromo-1,2,3,4-tetrahydronaphthalene-1,2,3,4-tetrayl tetraacetate (3l^{anti})

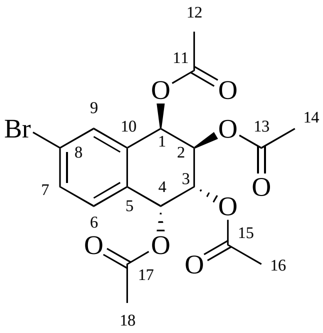

3l^{anti} - ^{1}H NMR (400 MHz, $CDCl_3$)

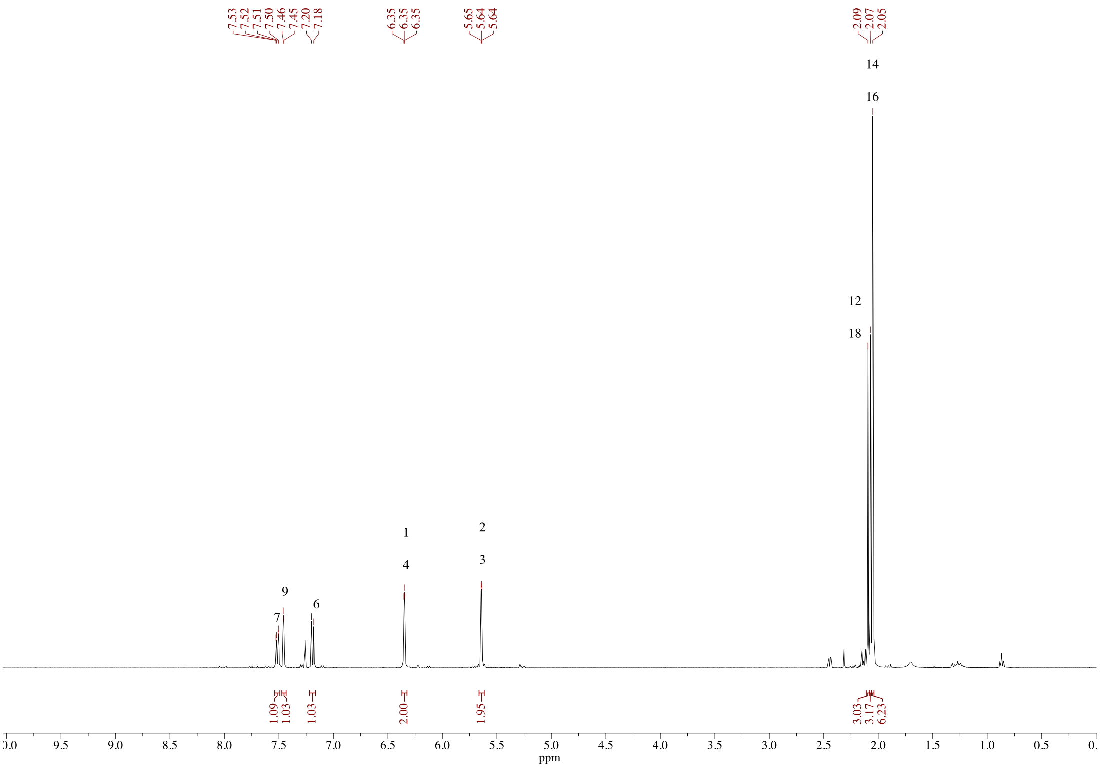

3l[anti] - ^{13}C NMR (100 MHz, $CDCl_3$)

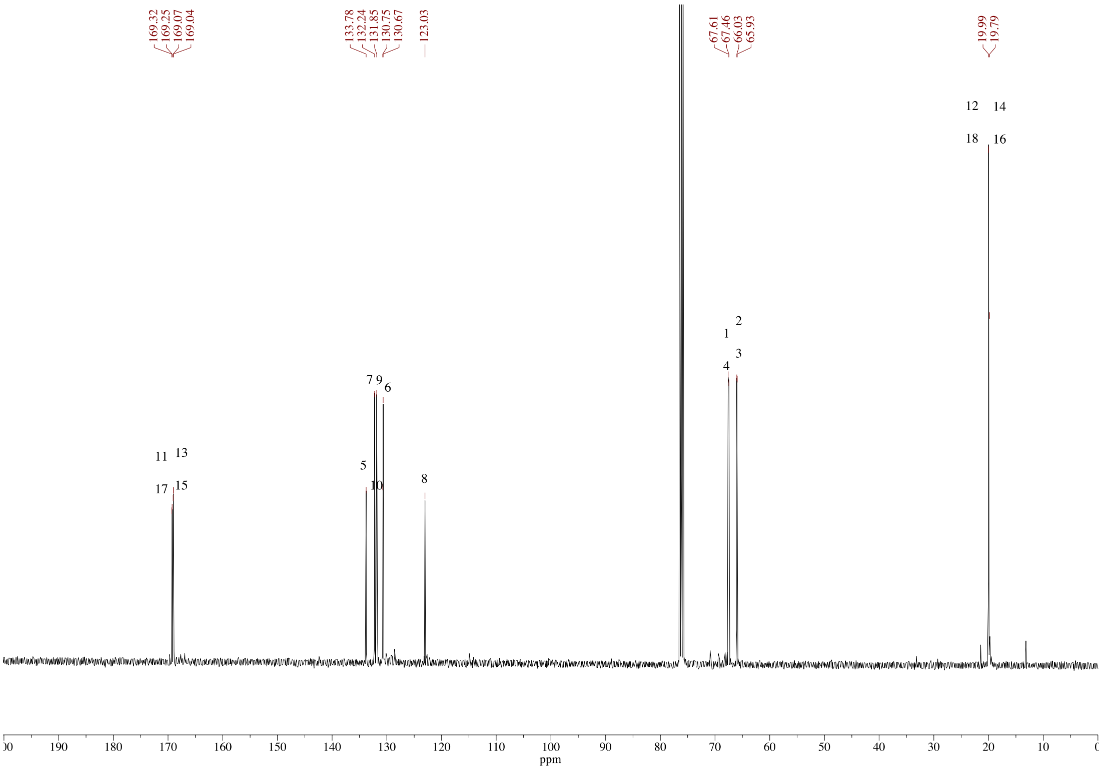

3l[anti] - DEPT ($CDCl_3$)

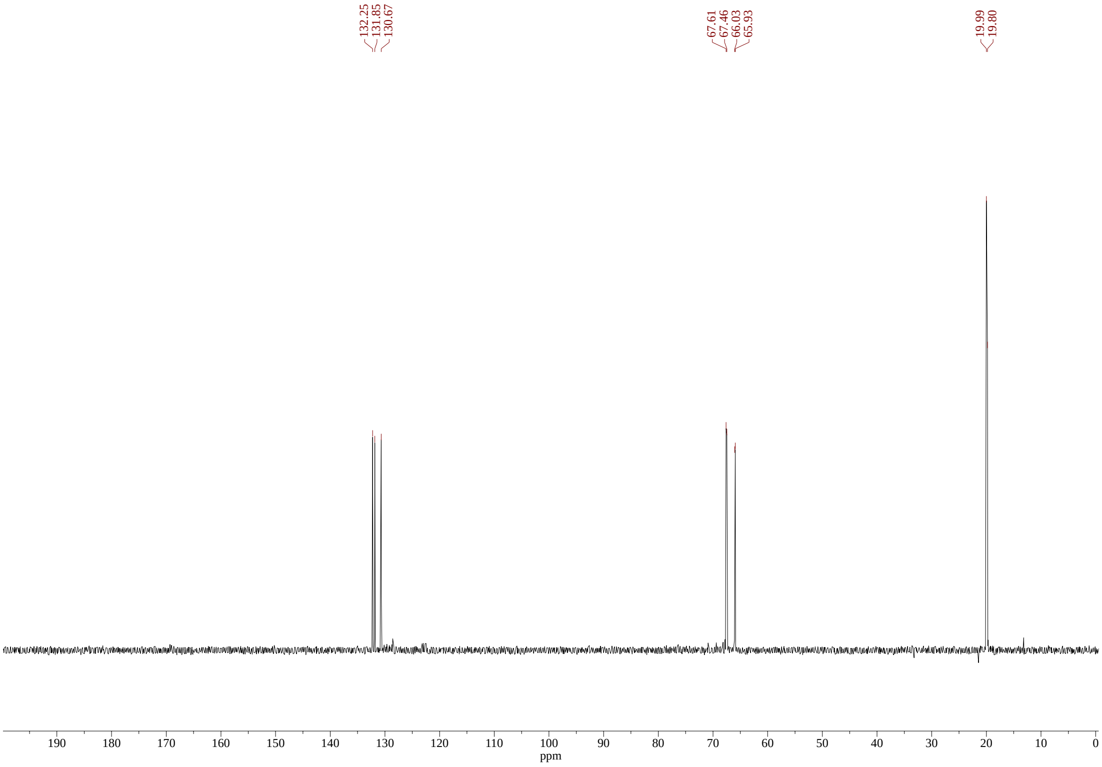

3l^{anti} - DEPTQ (CDCl$_3$)

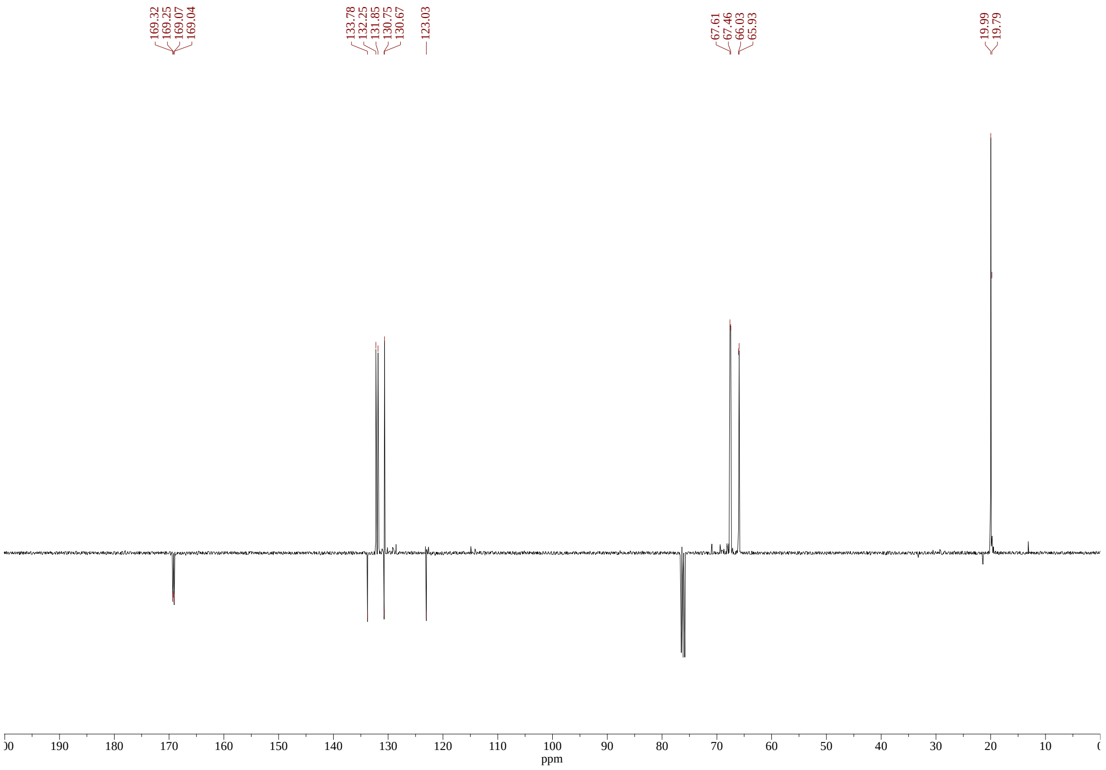

3l^{anti} - ^{1}H-^{1}H COSY (CDCl$_3$)

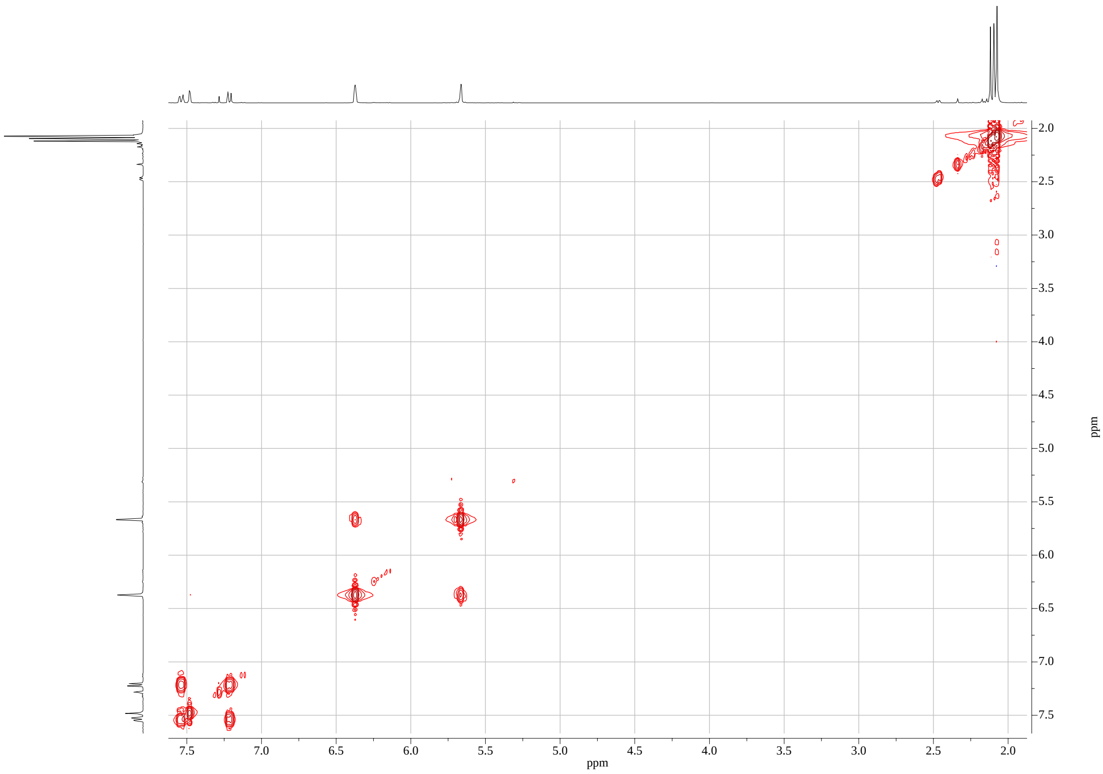

3l^{anti} - ^{1}H-^{13}C HSQCED (CDCl$_3$)

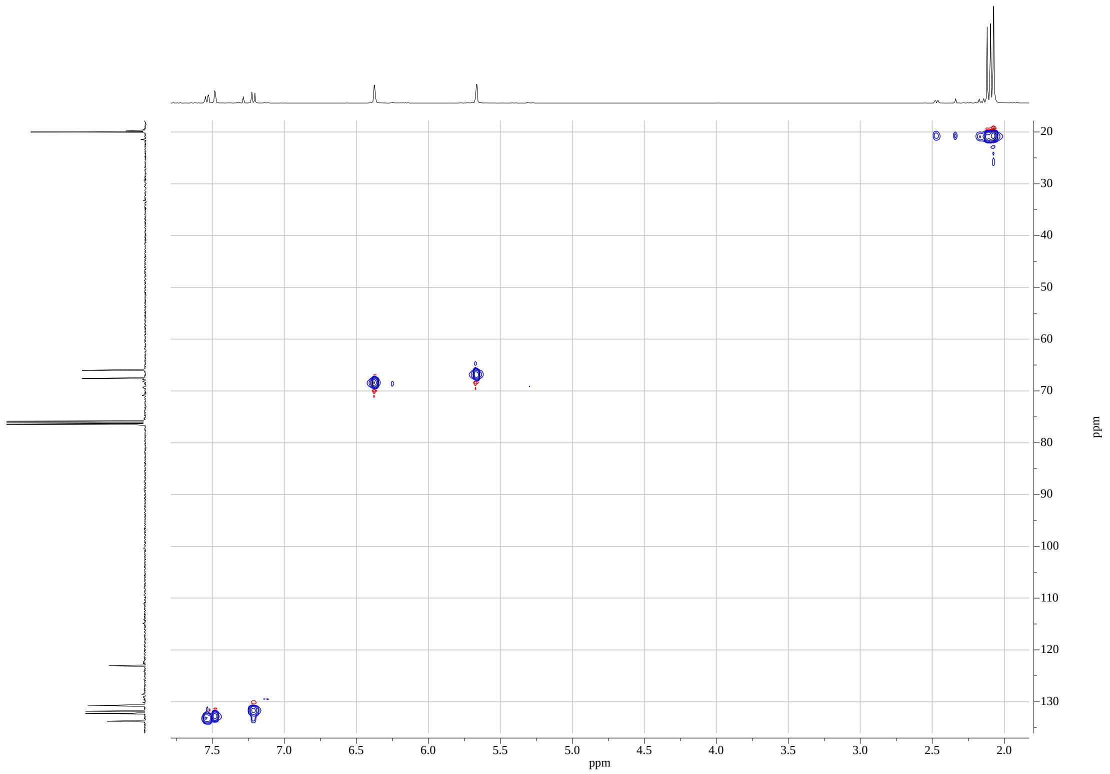

3l^{anti} - ^{1}H-^{13}C HMBC (CDCl$_3$)

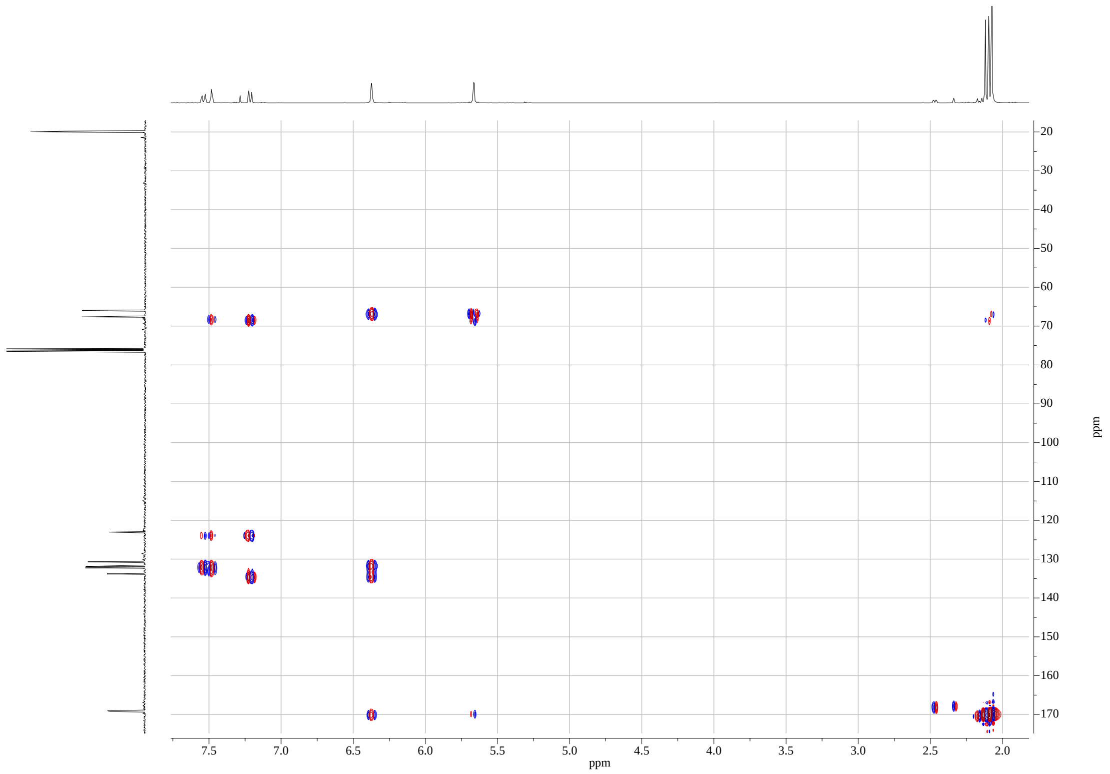

(1α,2α,3α,4α)-6-chloro-1,2,3,4-tetrahydronaphthalene-1,2,3,4-tetrayl tetraacetate (3m^{syn})

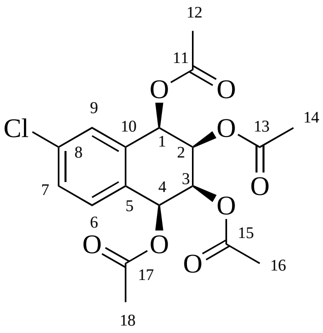

3m^{syn} - ^{1}H NMR (400 MHz, $CDCl_3$)

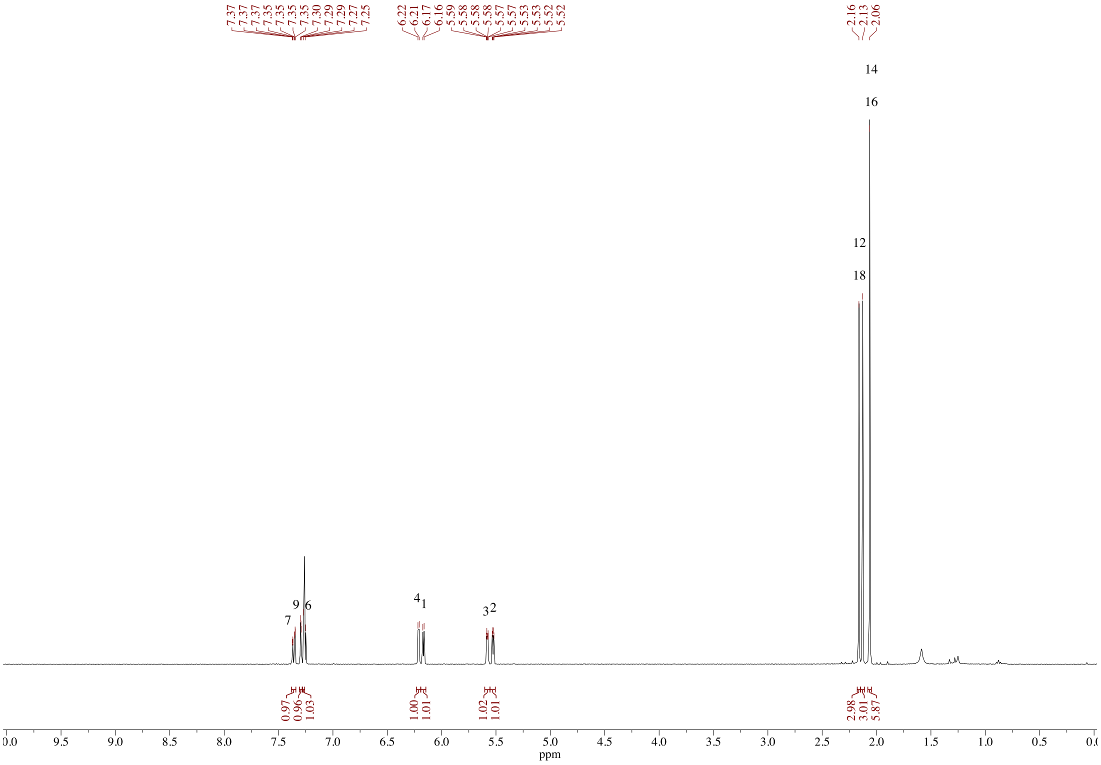

3m^{syn} - ^{13}C NMR (100 MHz, $CDCl_3$)

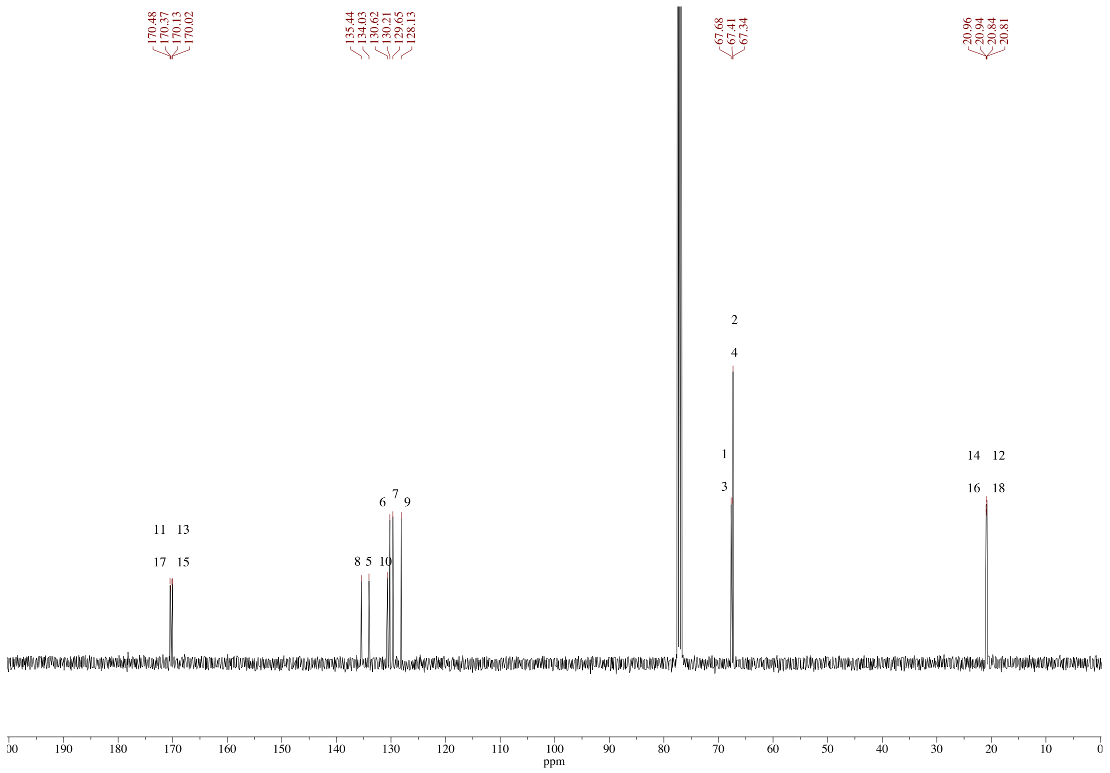

3m^{syn} - DEPT ($CDCl_3$)

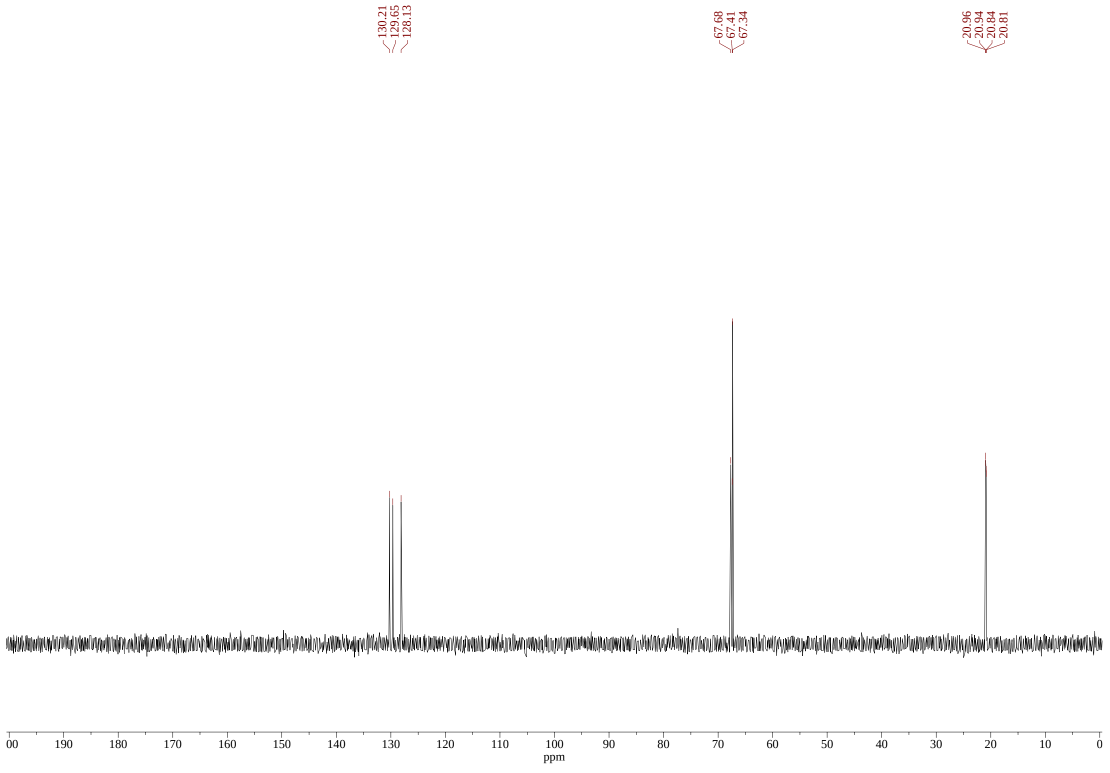

3m^{syn} - DEPTQ ($CDCl_3$)

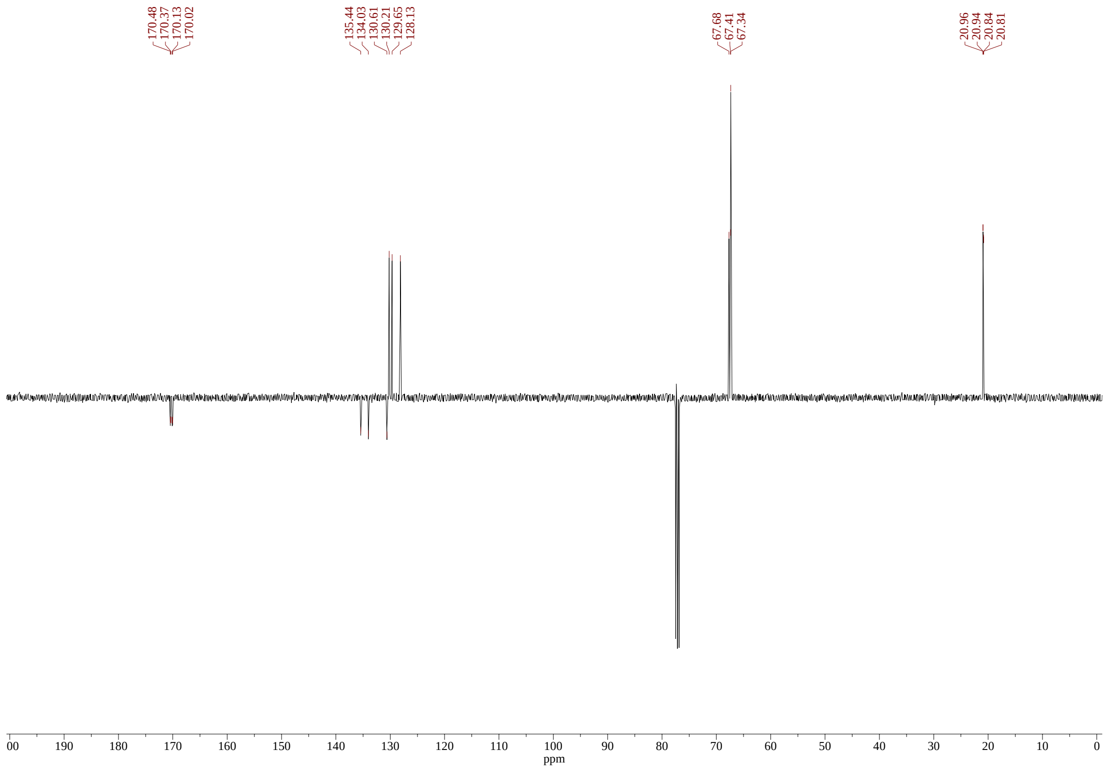

3m^{syn} - ^1H-^1H COSY ($CDCl_3$)

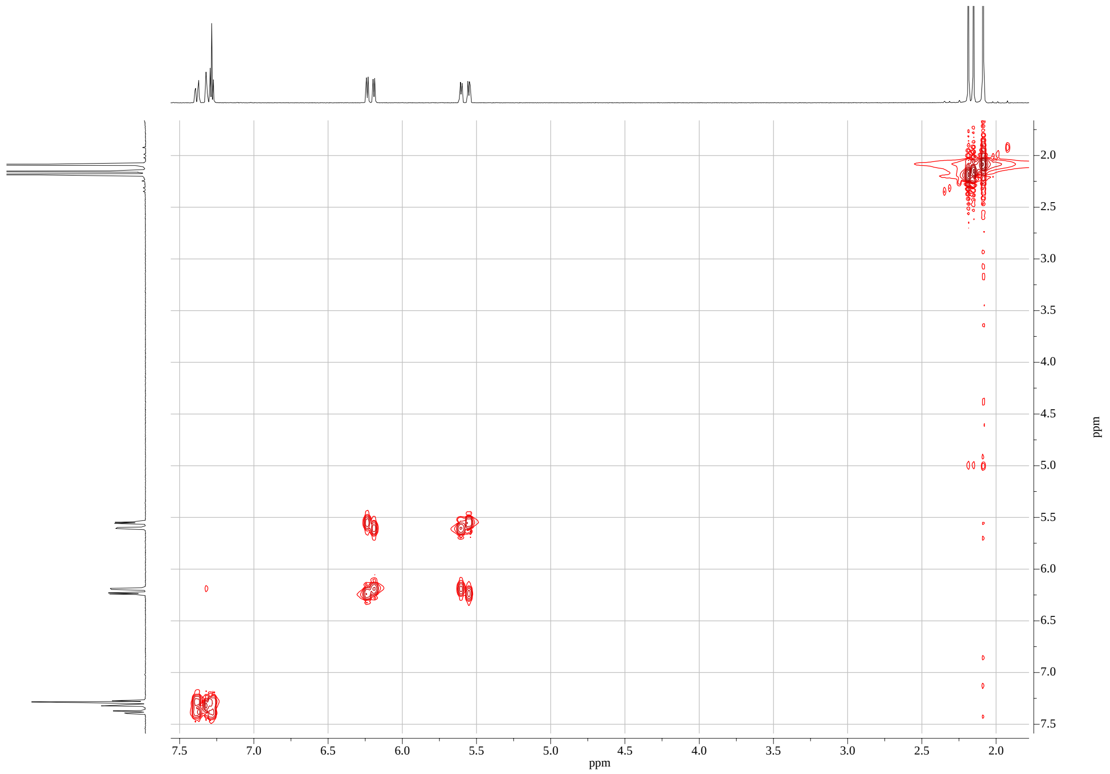

3m^syn^ - ^{1}H-^{13}C HSQCED (CDCl3)

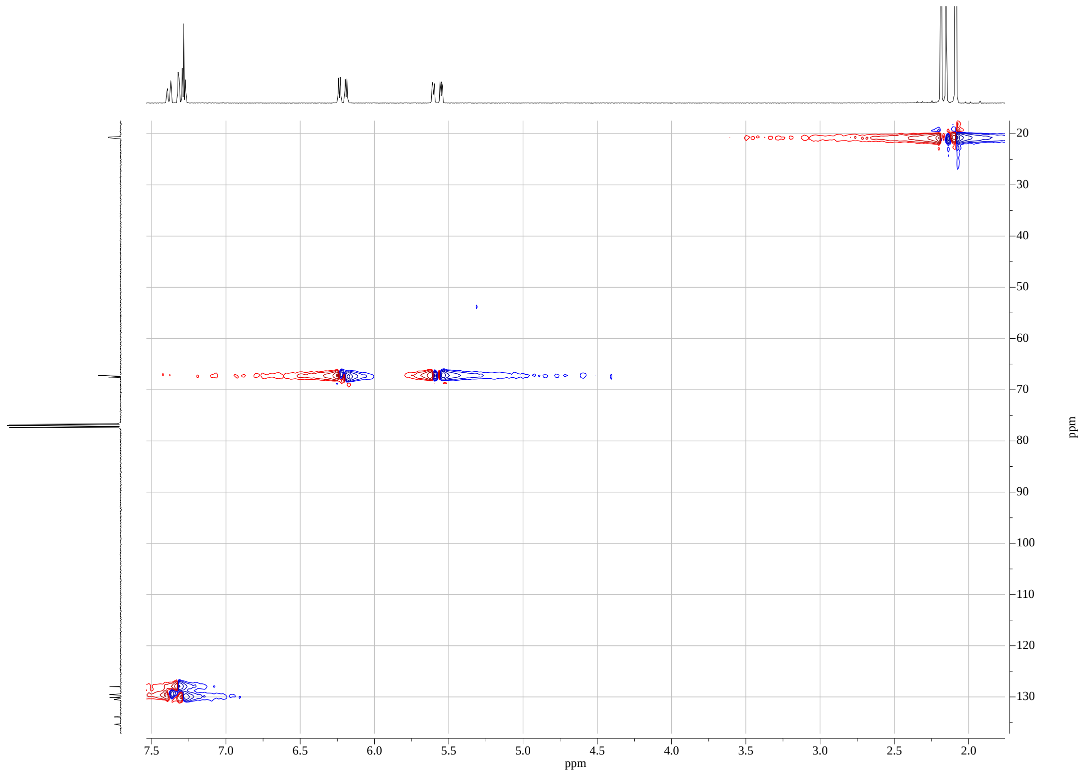

3m^syn^ - ^{1}H-^{13}C HMBC (CDCl3)

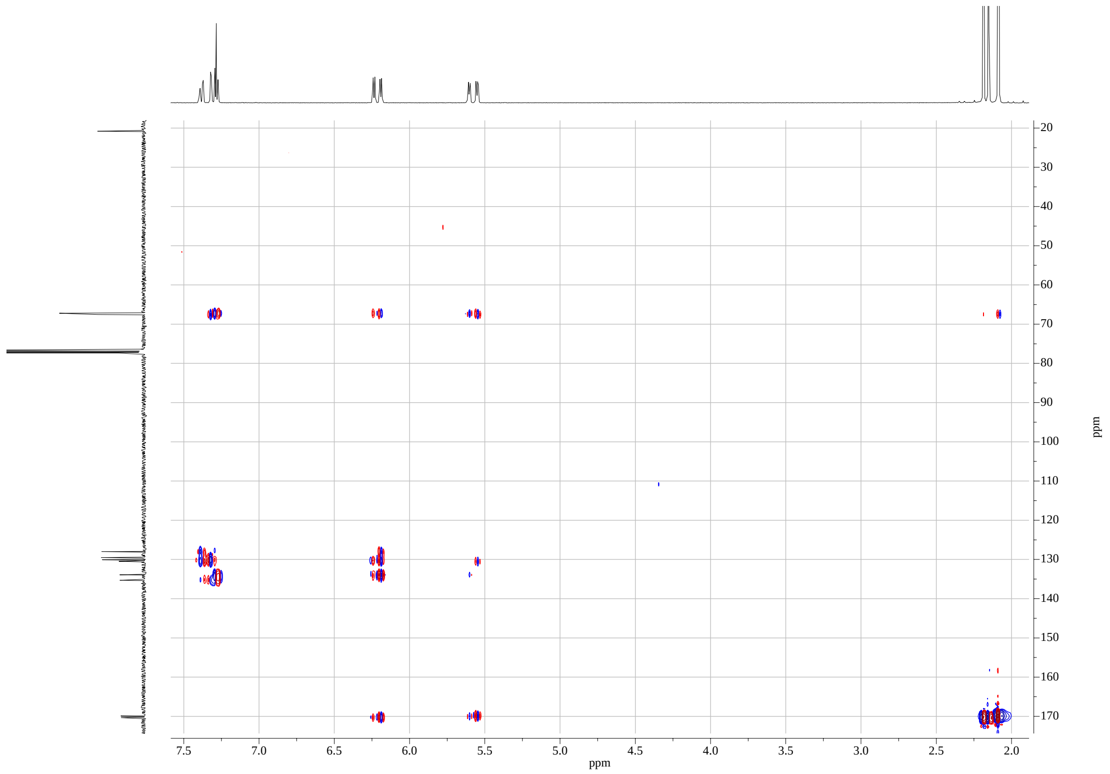

(1α,2α,3β,4β)-6-chloro-1,2,3,4-tetrahydronaphthalene-1,2,3,4-tetrayl tetraacetate (3m^anti^)

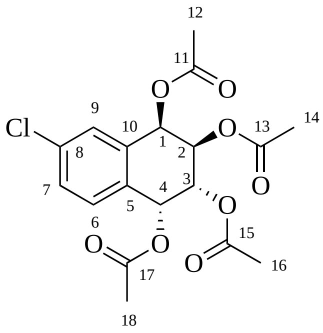

3m^anti^ - ^{1}H NMR (400 MHz, $CDCl_3$)

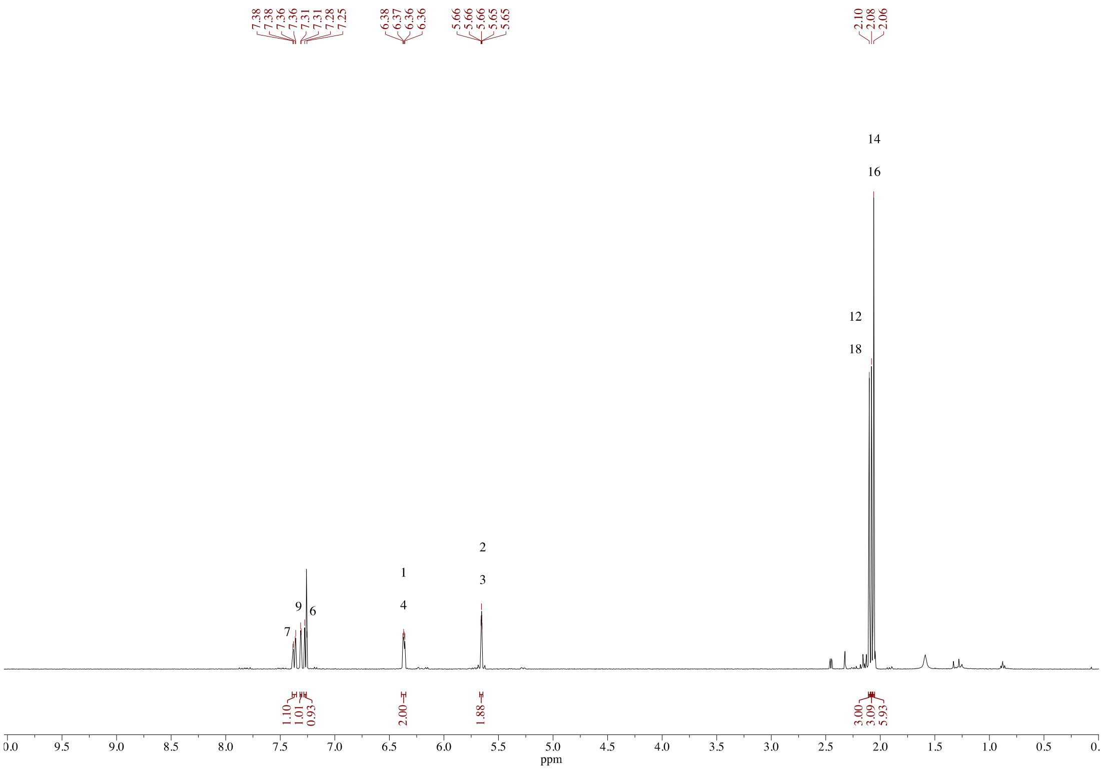

3m^{anti} - ^{13}C NMR (100 MHz, $CDCl_3$)

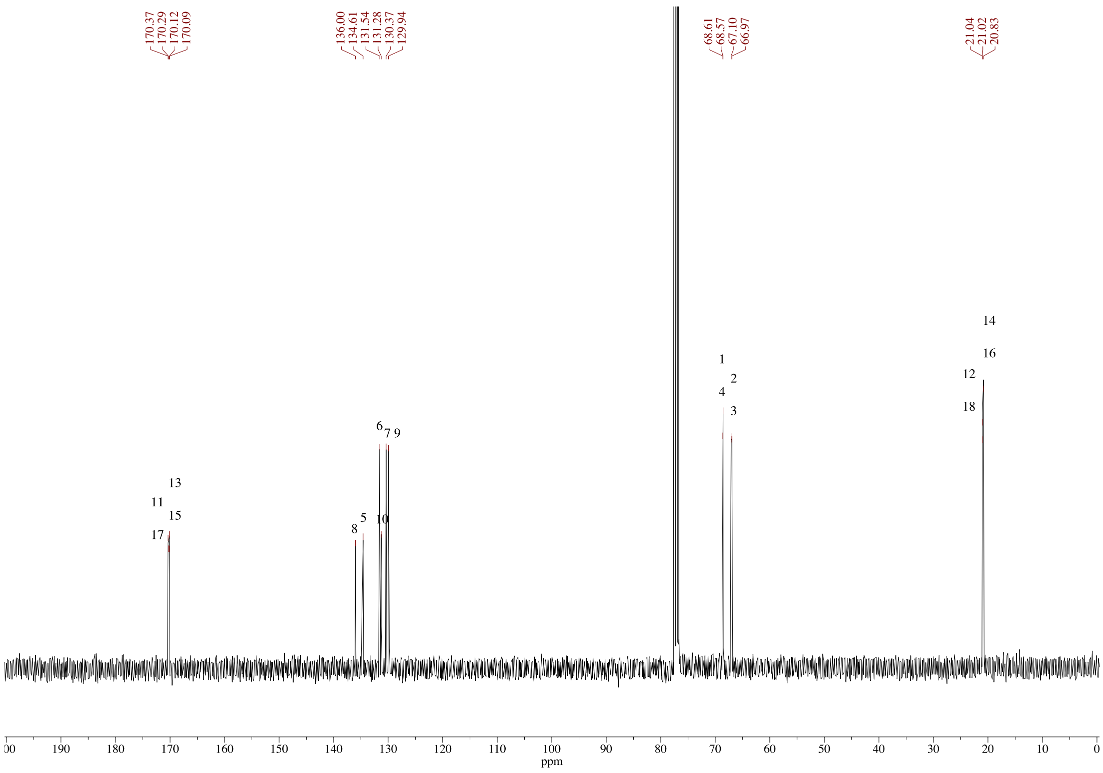

3m^{anti} - DEPT ($CDCl_3$)

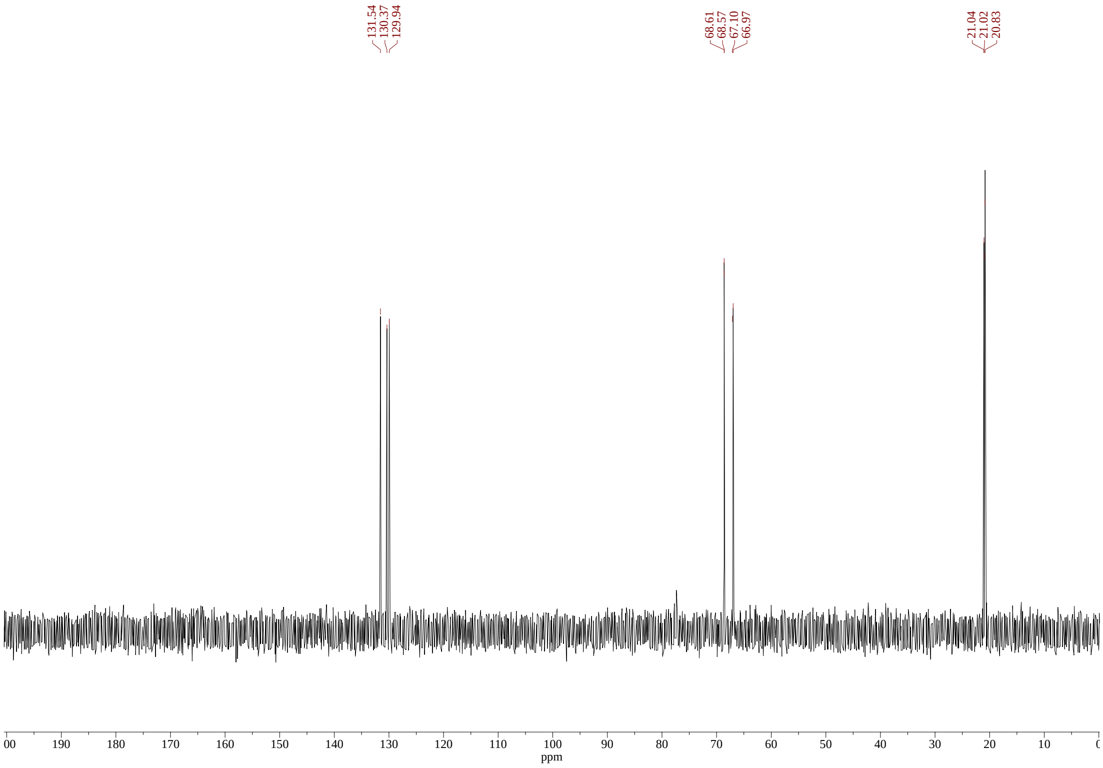

3m[anti] - DEPTQ ($CDCl_3$)

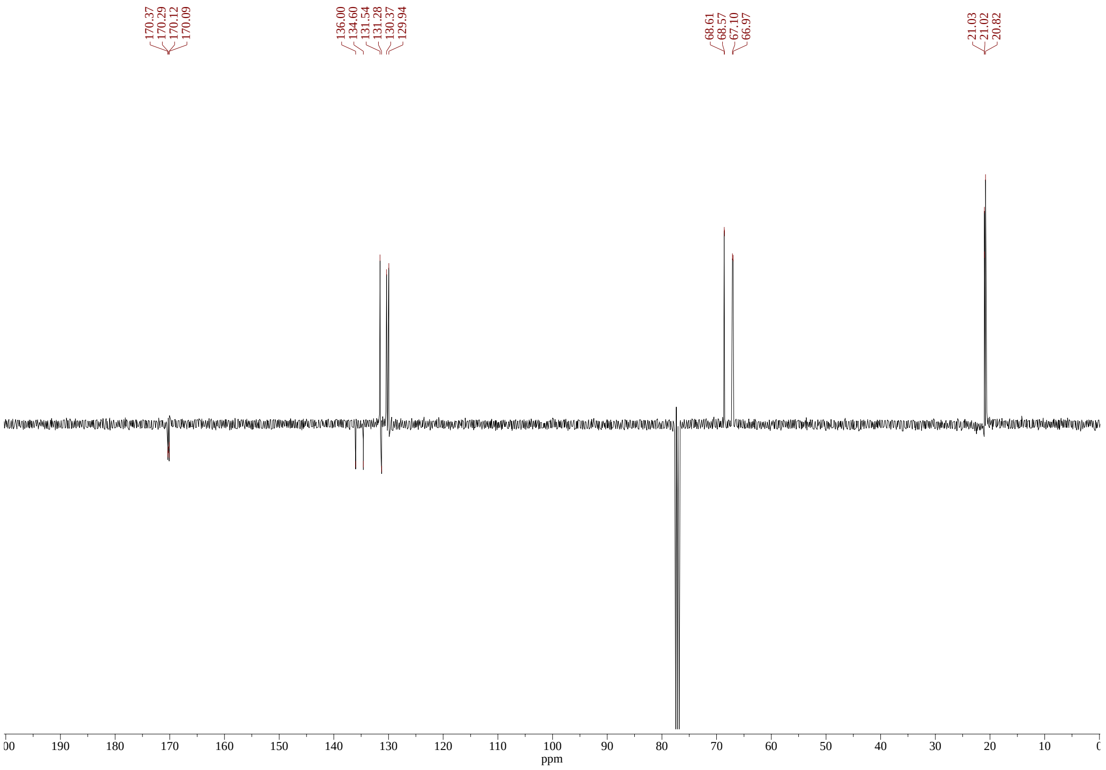

3m[anti] - ¹H-¹H COSY ($CDCl_3$)

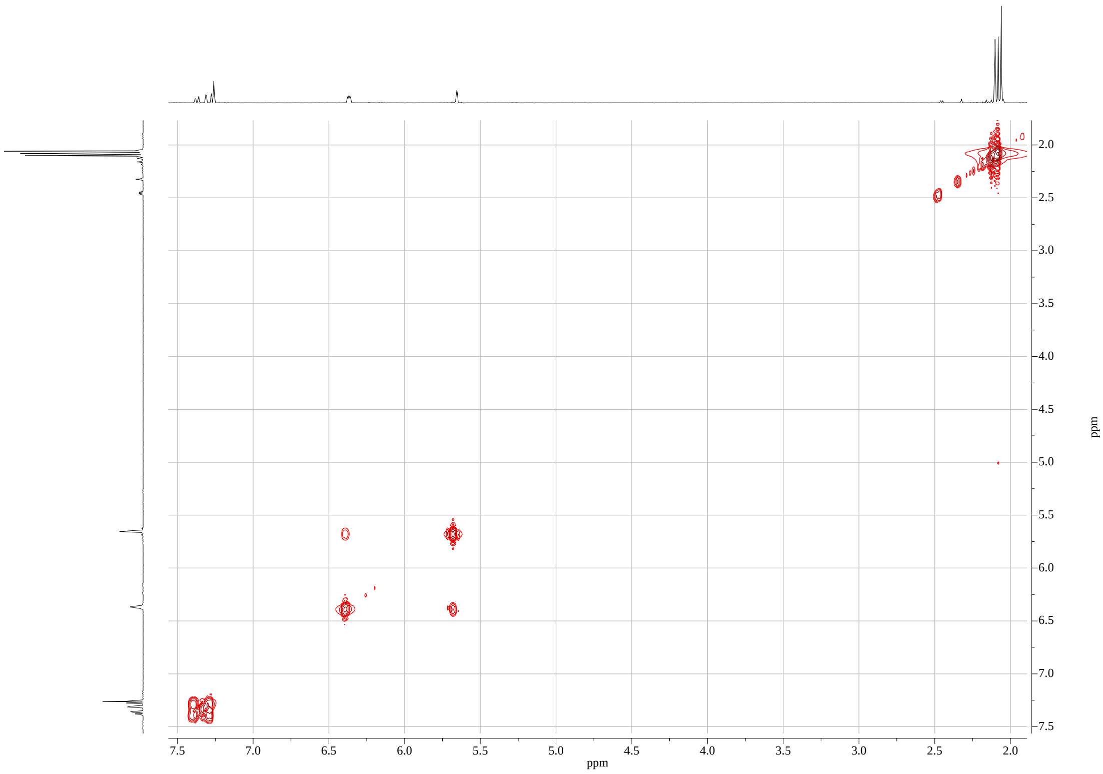

3m[anti] - ^{1}H-^{13}C HSQCED ($CDCl_3$)

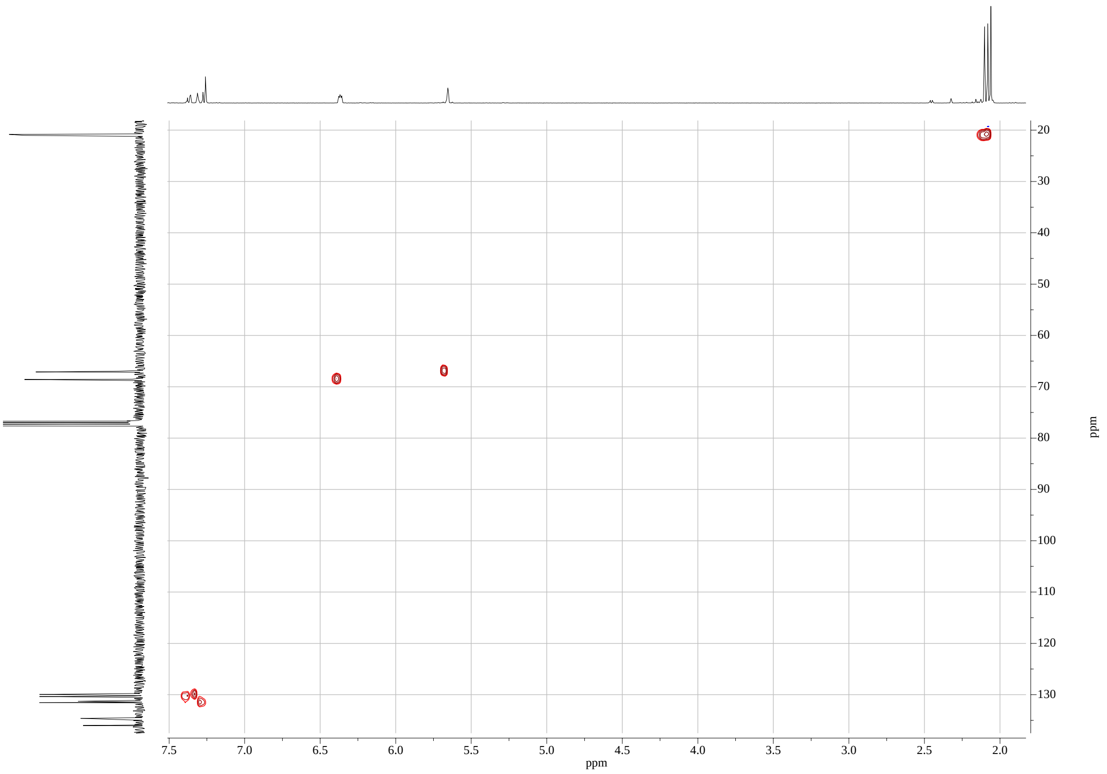

3m[anti] - ^{1}H-^{13}C HMBC ($CDCl_3$)

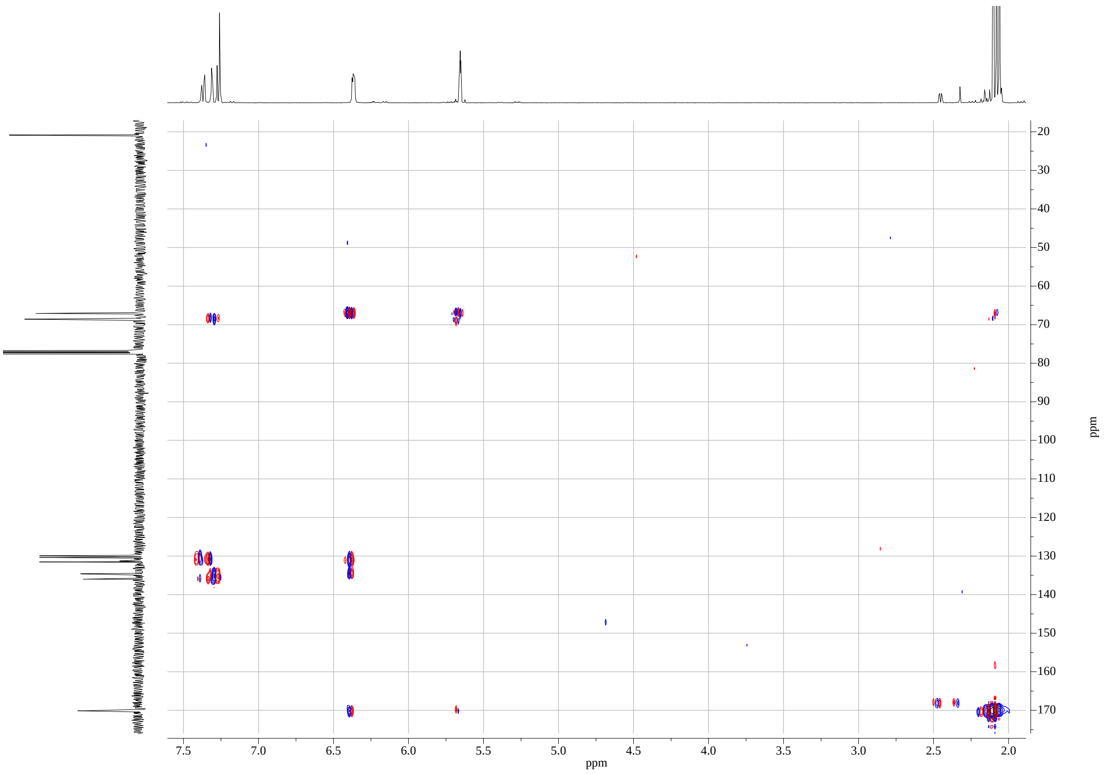

(1α,2α,3α,4α)-1,2,3,4-tetrahydronaphthalene-1,2,3,4,6-pentayl pentaacetate (3f^{syn})

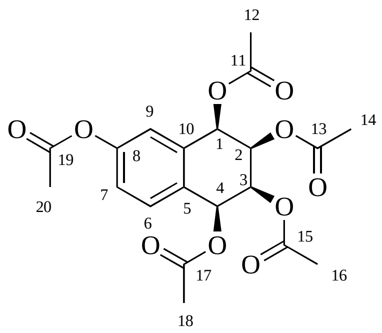

3f^{syn} - ^{1}H NMR (400 MHz, $CDCl_3$)

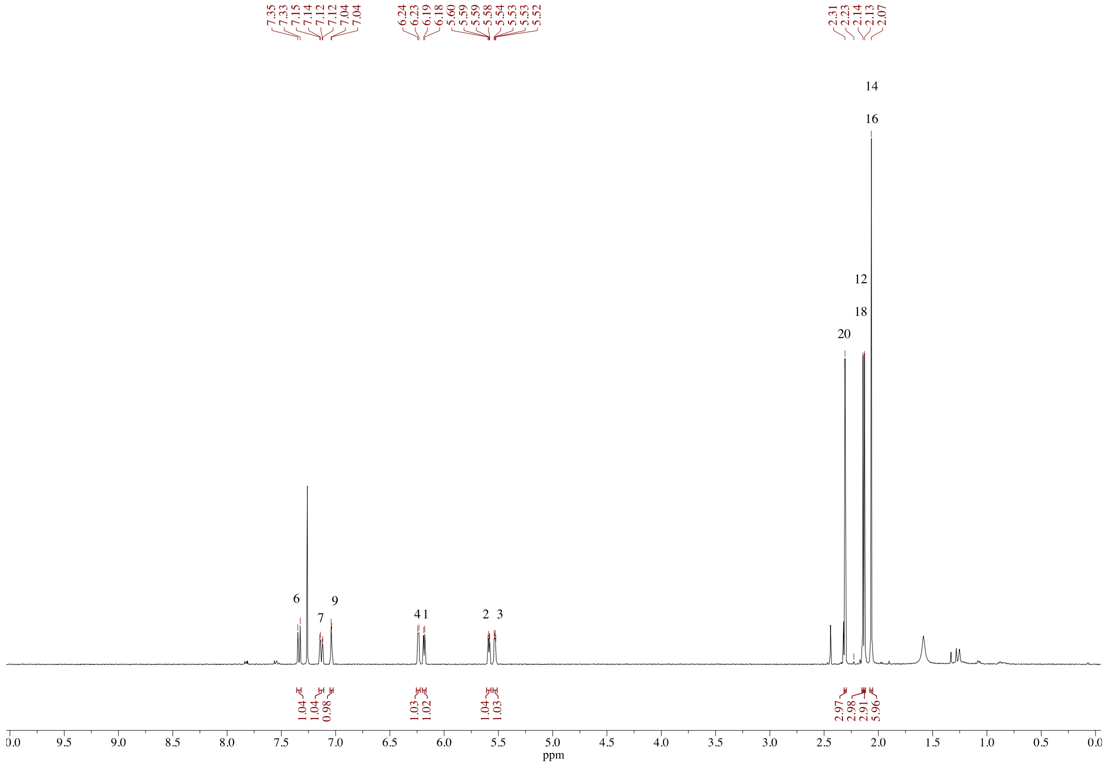

3f^{syn} - ^{13}C NMR (100 MHz, $CDCl_3$)

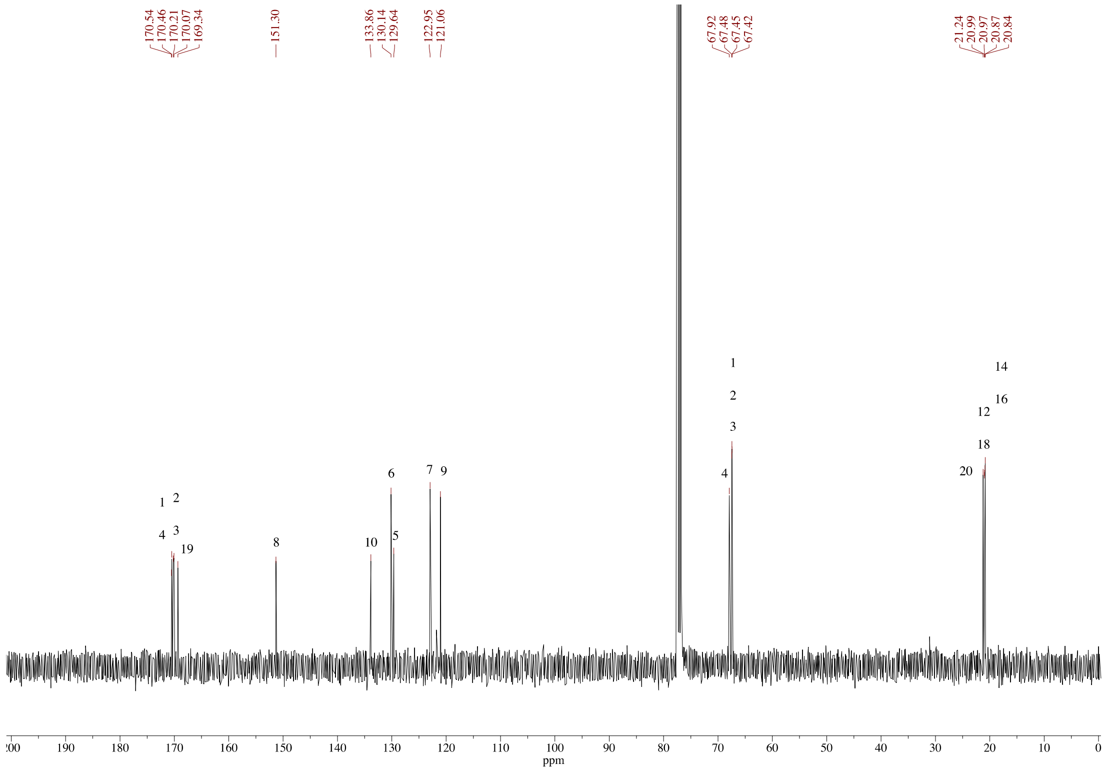

3f^{syn} - DEPT ($CDCl_3$)

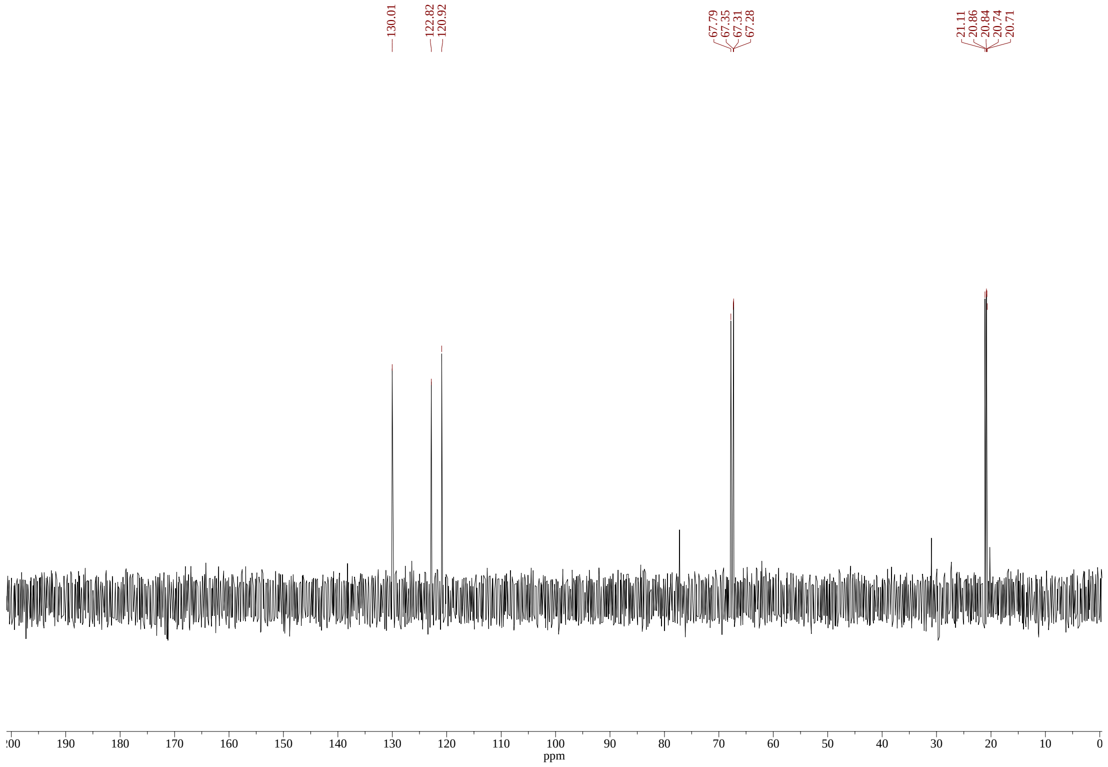

3f[syn] - DEPTQ ($CDCl_3$)

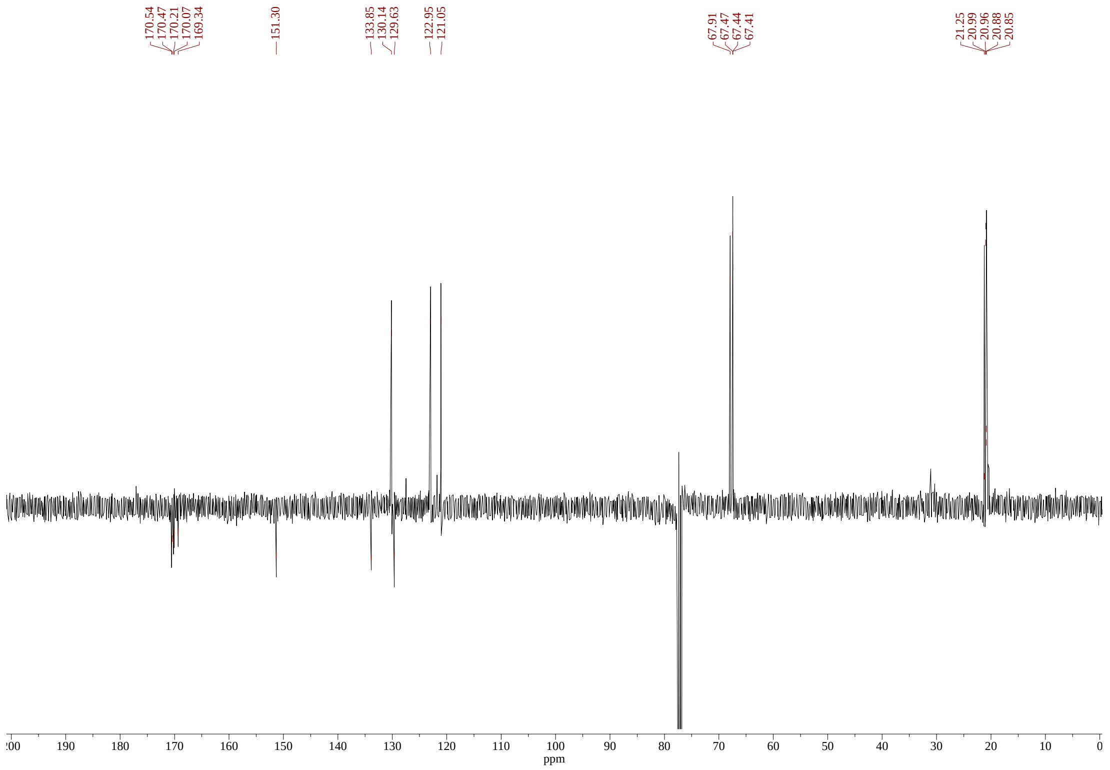

3f[syn] - ^1H-^1H COSY ($CDCl_3$)

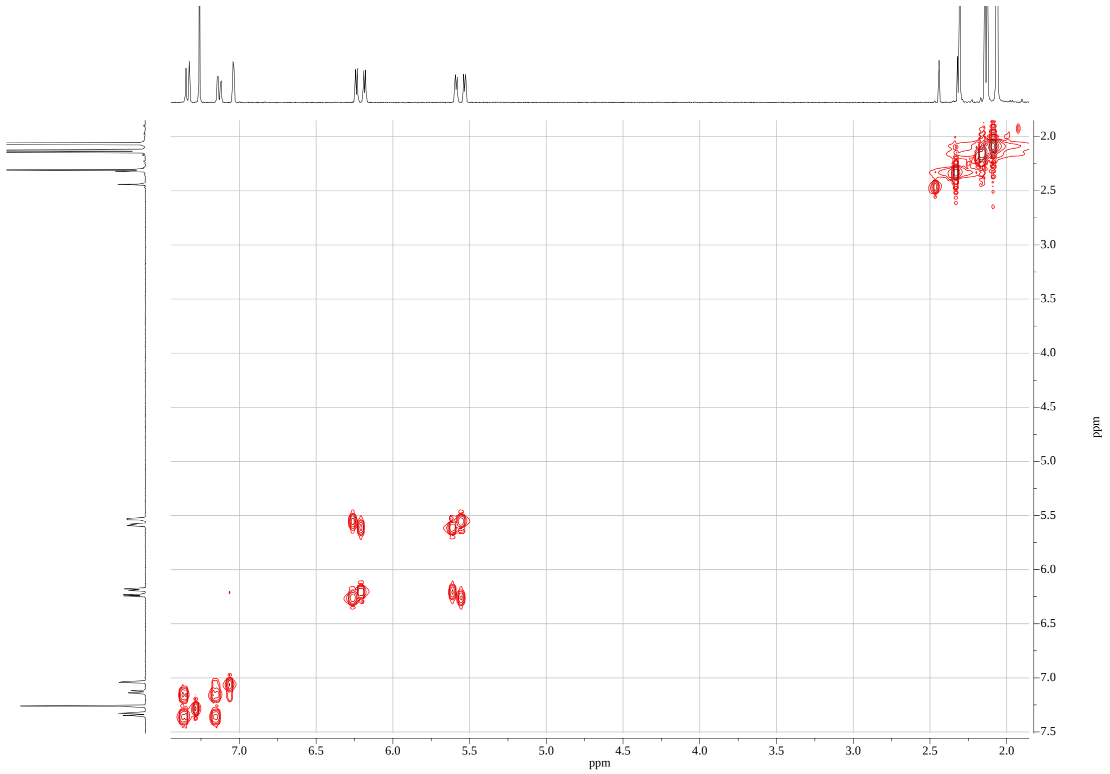

3f^syn - ^{1}H-^{13}C HSQCED ($CDCl_3$)

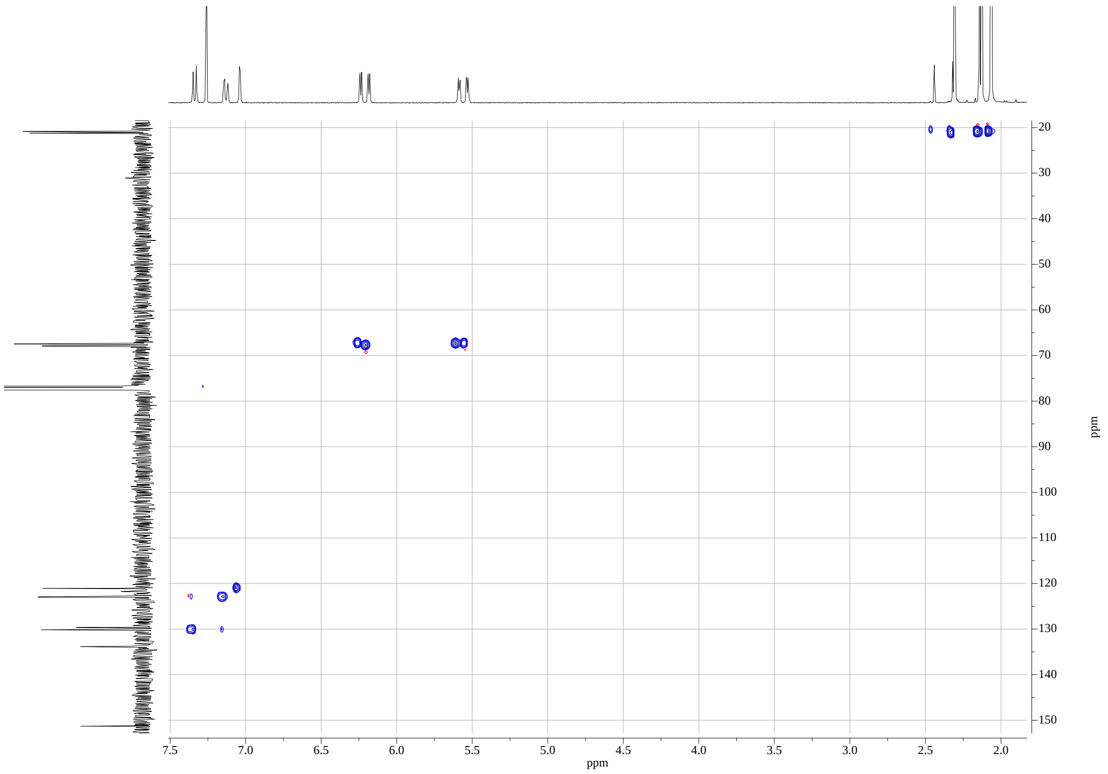

3f^syn - ^{1}H-^{13}C HMBC ($CDCl_3$)

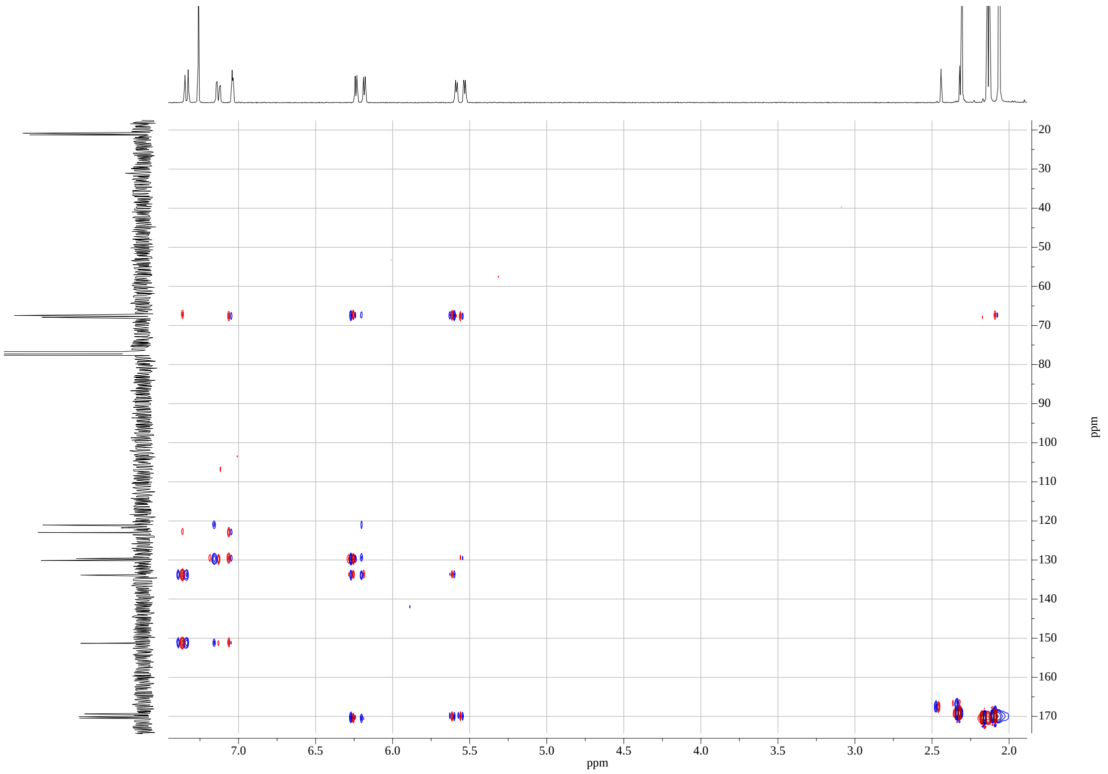

(1α,2α,3β,4β)-1,2,3,4-tetrahydronaphthalene-1,2,3,4,6-pentayl pentaacetate (3f[anti])

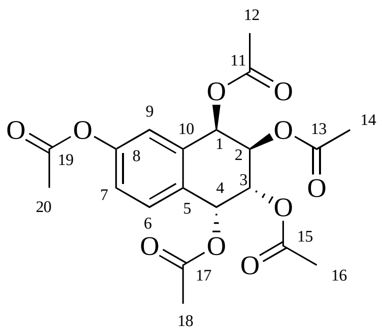

3f[anti] - ^{1}H NMR (400 MHz, $CDCl_3$)

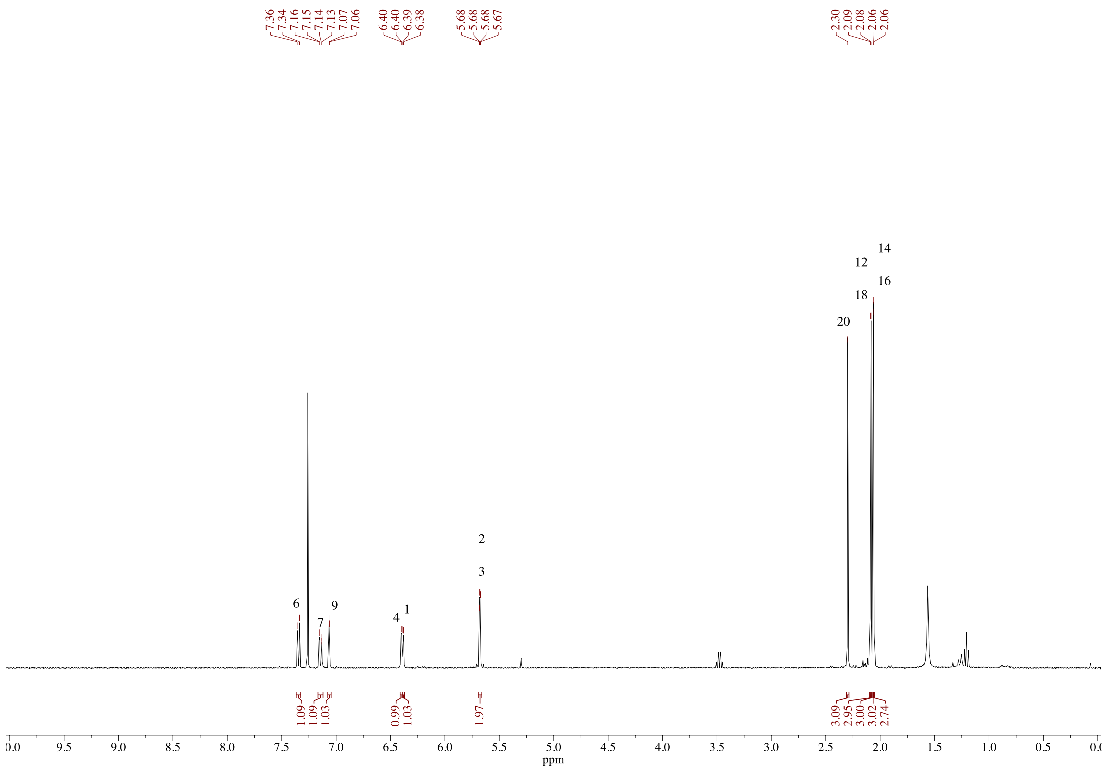

3fanti - ^{13}C NMR (100 MHz, $CDCl_3$)

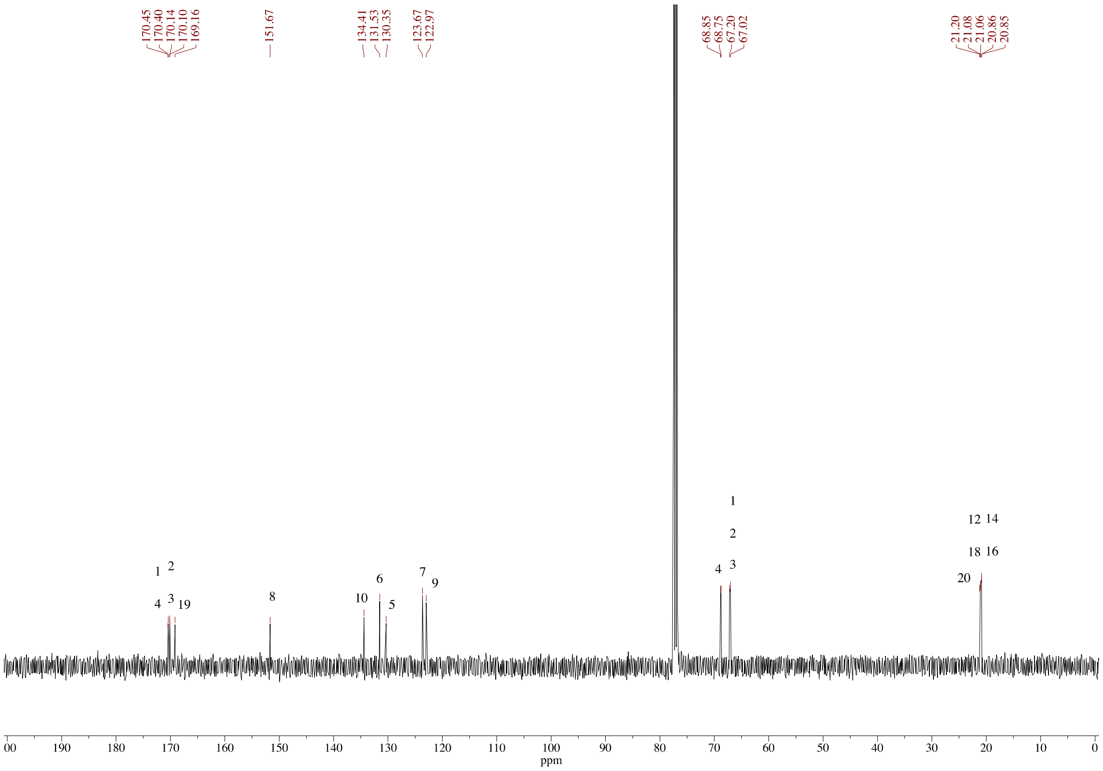

3fanti - DEPT ($CDCl_3$)

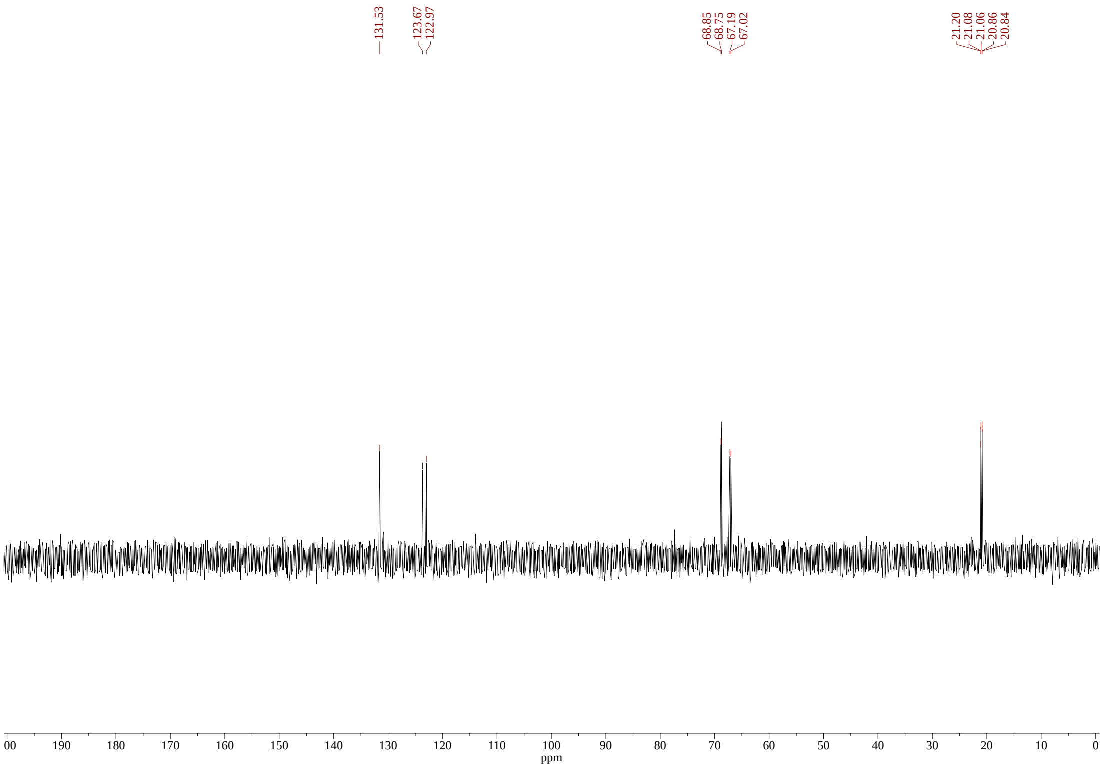

3f[anti] - DEPTQ ($CDCl_3$)

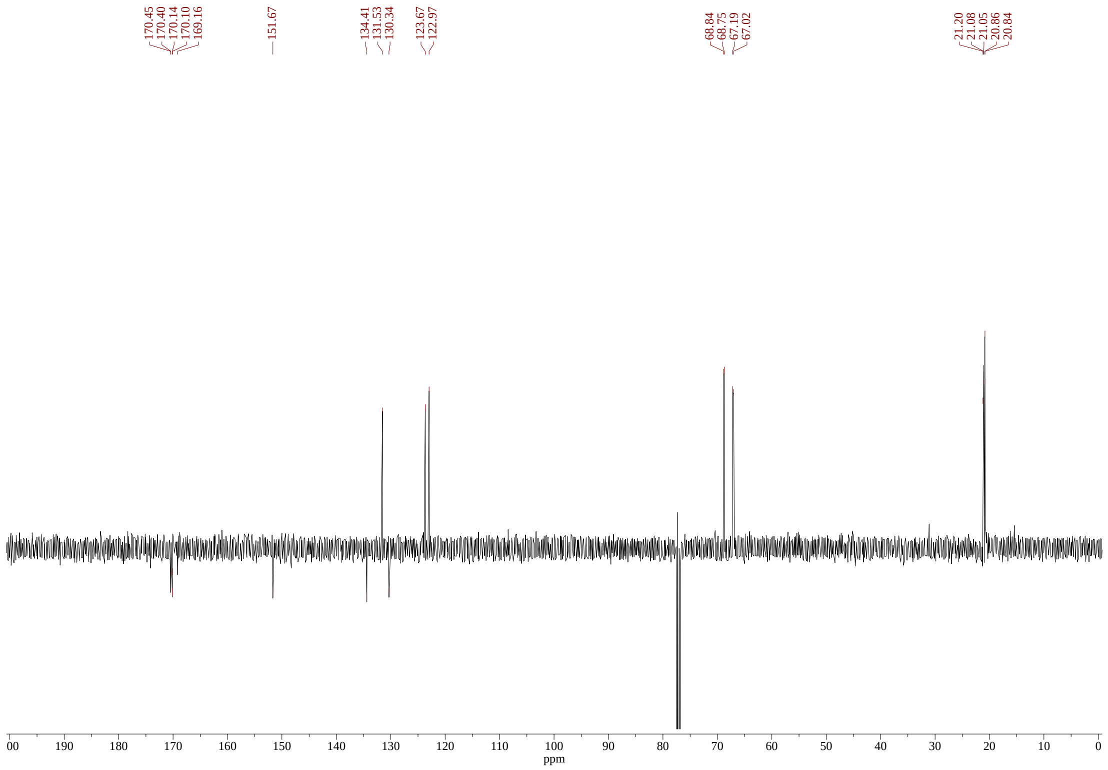

3f[anti] - ^1H-^1H COSY ($CDCl_3$)

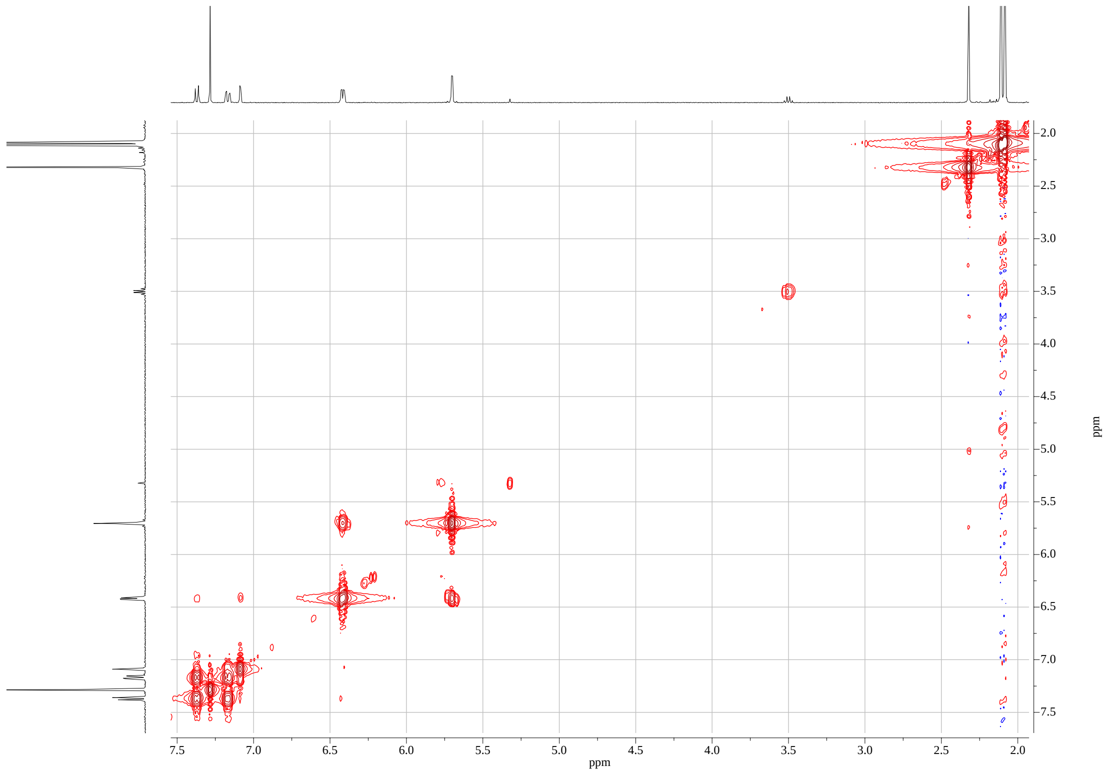

3f[anti] - ^{1}H-^{13}C HSQCED (CDCl$_3$)

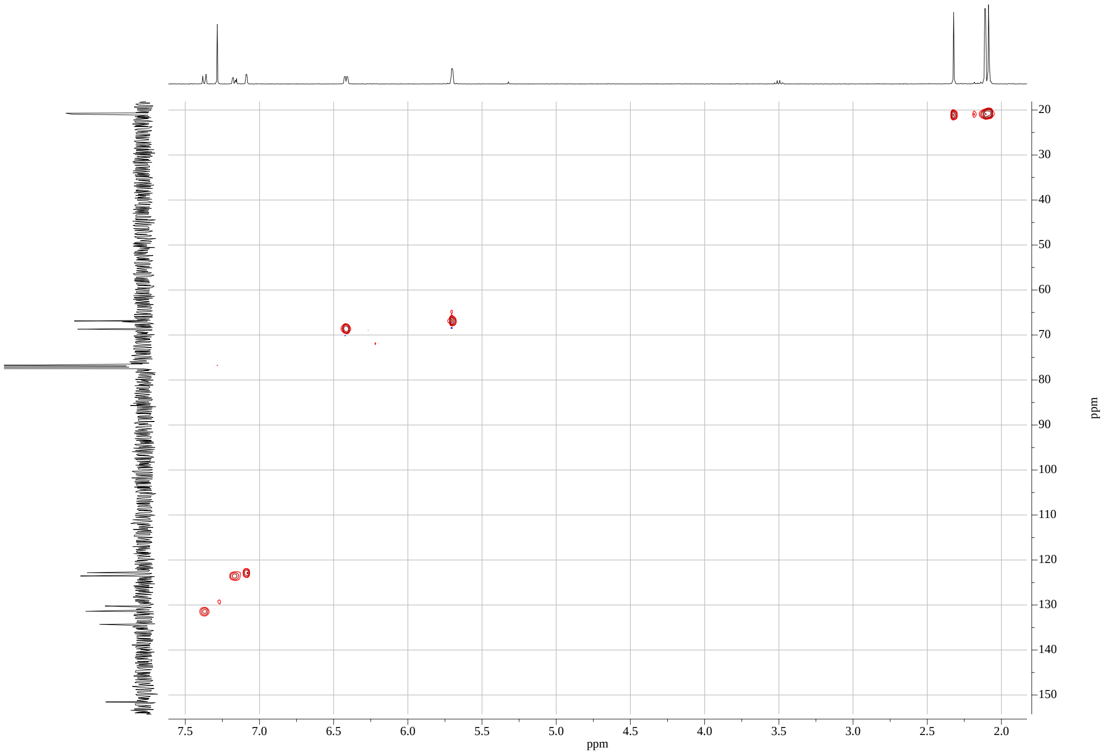

3f[anti] - ^{1}H-^{13}C HMBC (CDCl$_3$)

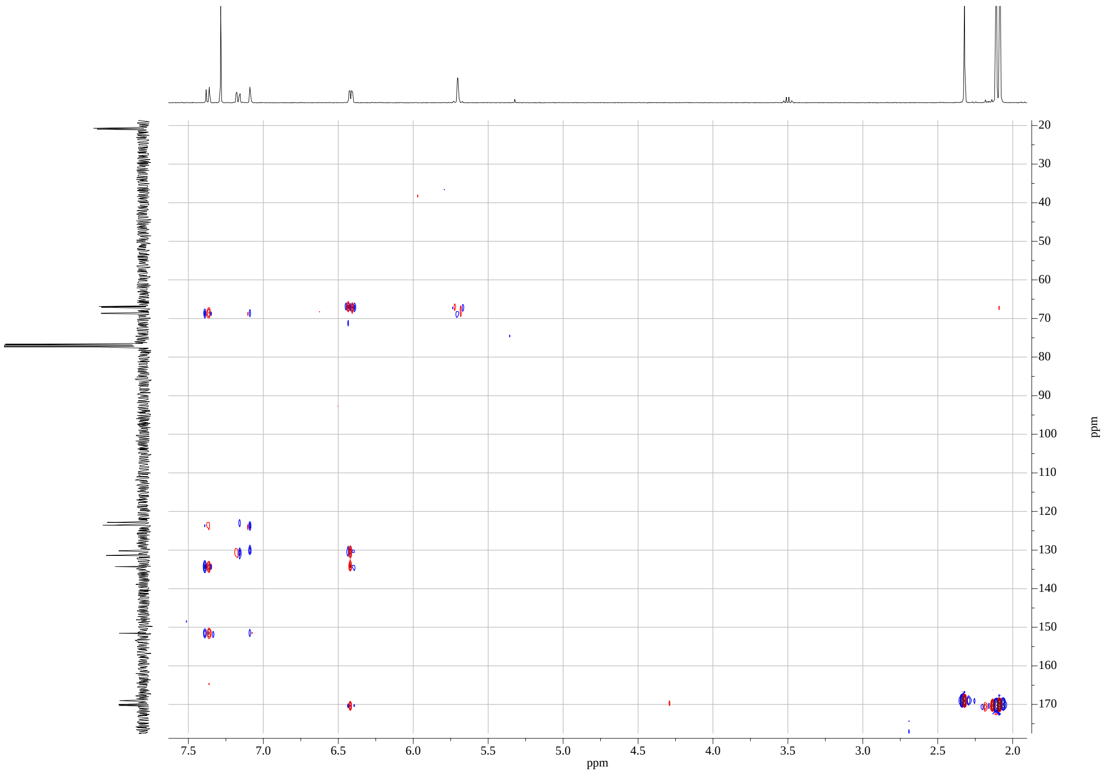

(1α,2α,3α,4α)-1,2,3,4-tetrahydronaphthalene-1,2,3,4,6-pentayl pentaacetate (3d^{syn})

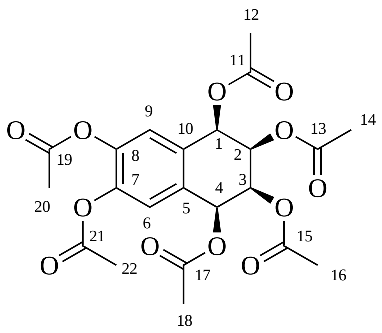

3d^{syn} - ^{1}H NMR (400 MHz, CDCl$_3$)

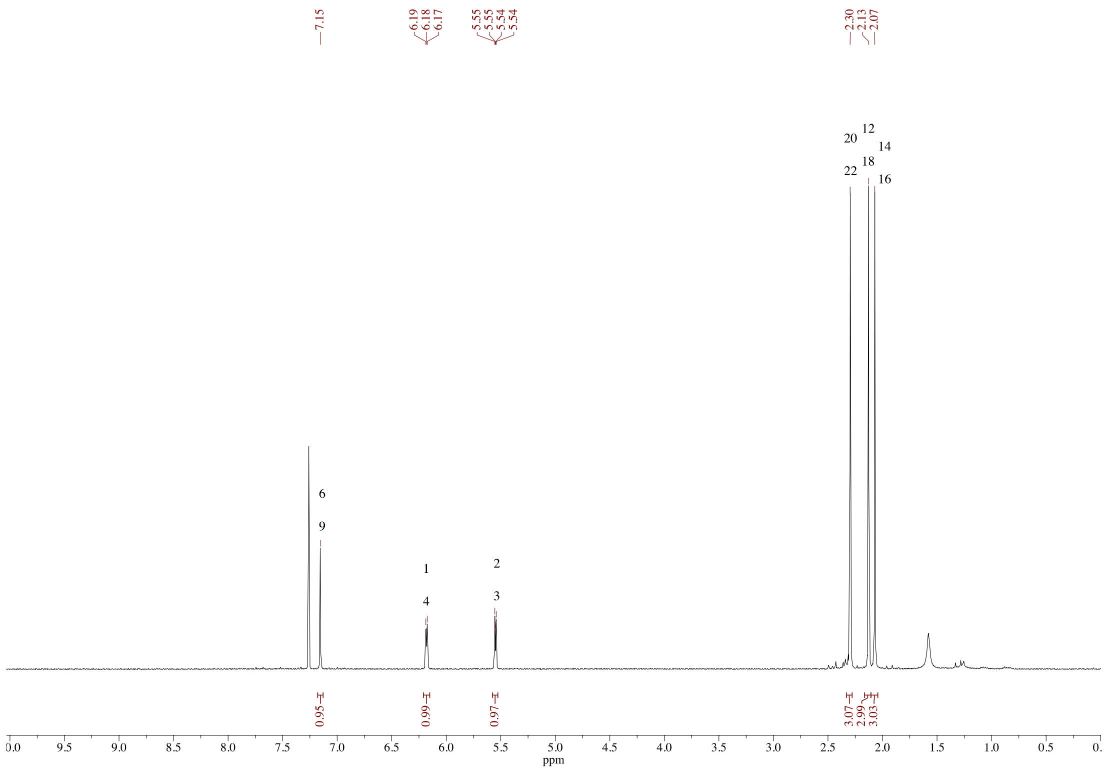

3d[syn] - ^{13}C NMR (100 MHz, $CDCl_3$)

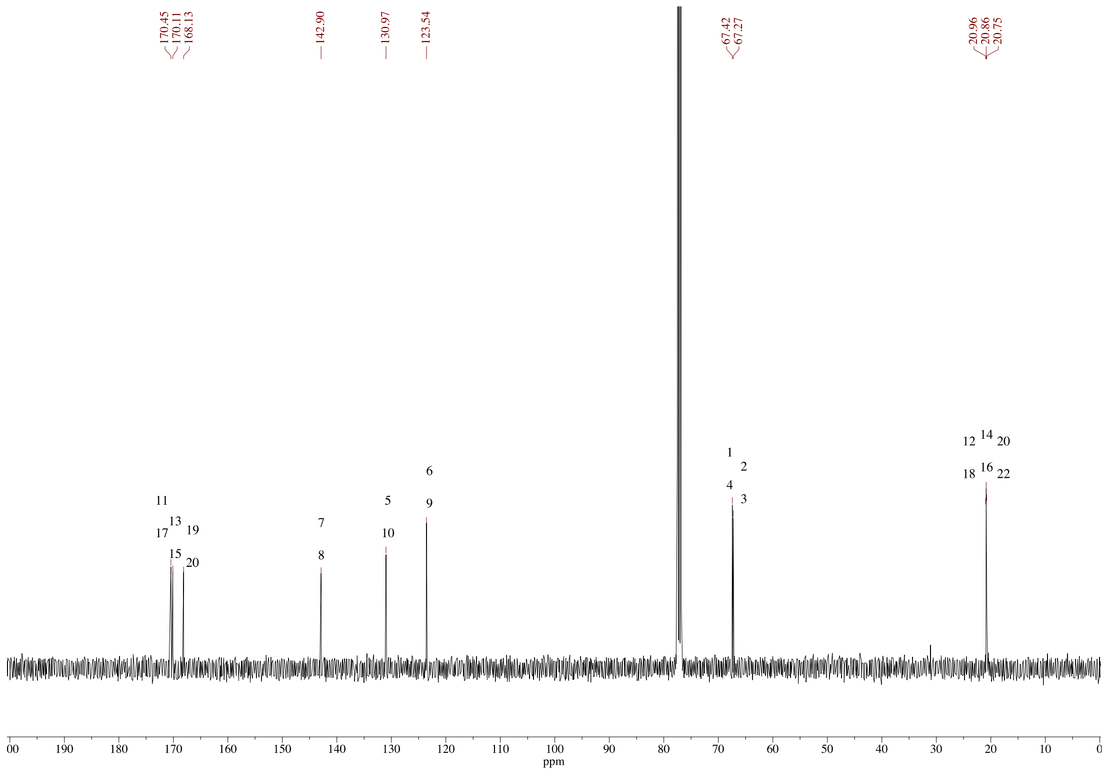

3d[syn] - DEPT ($CDCl_3$)

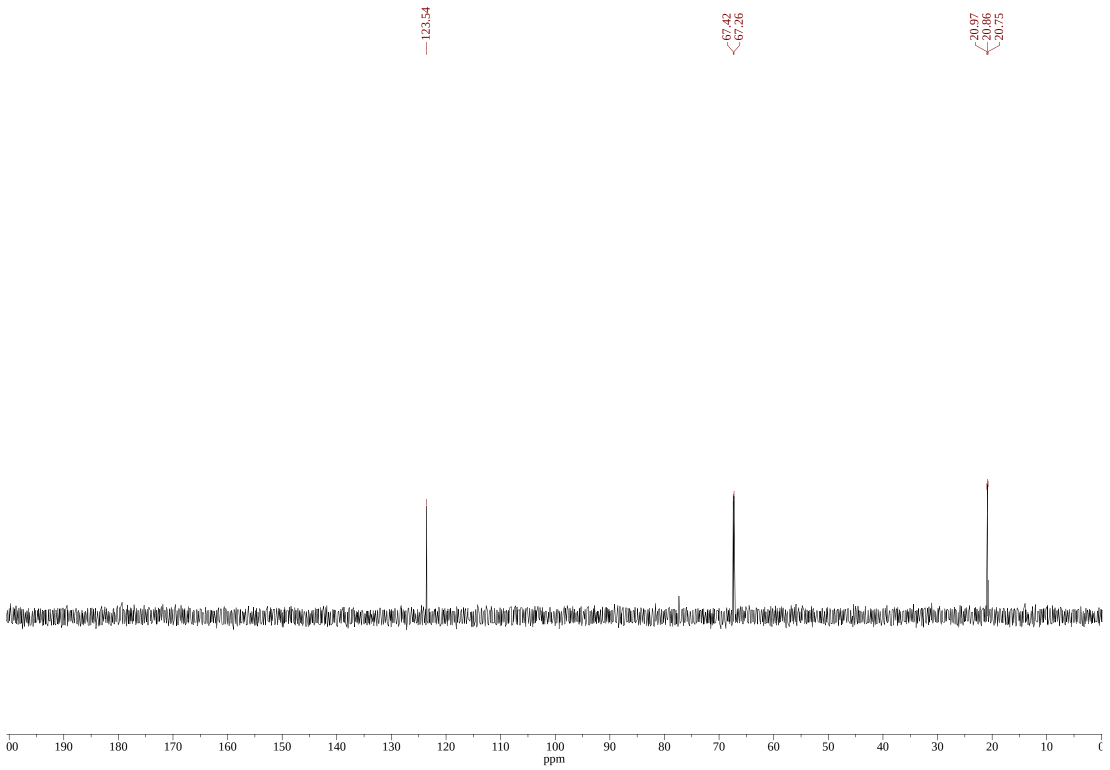

3d^{syn} - DEPTQ ($CDCl_3$)

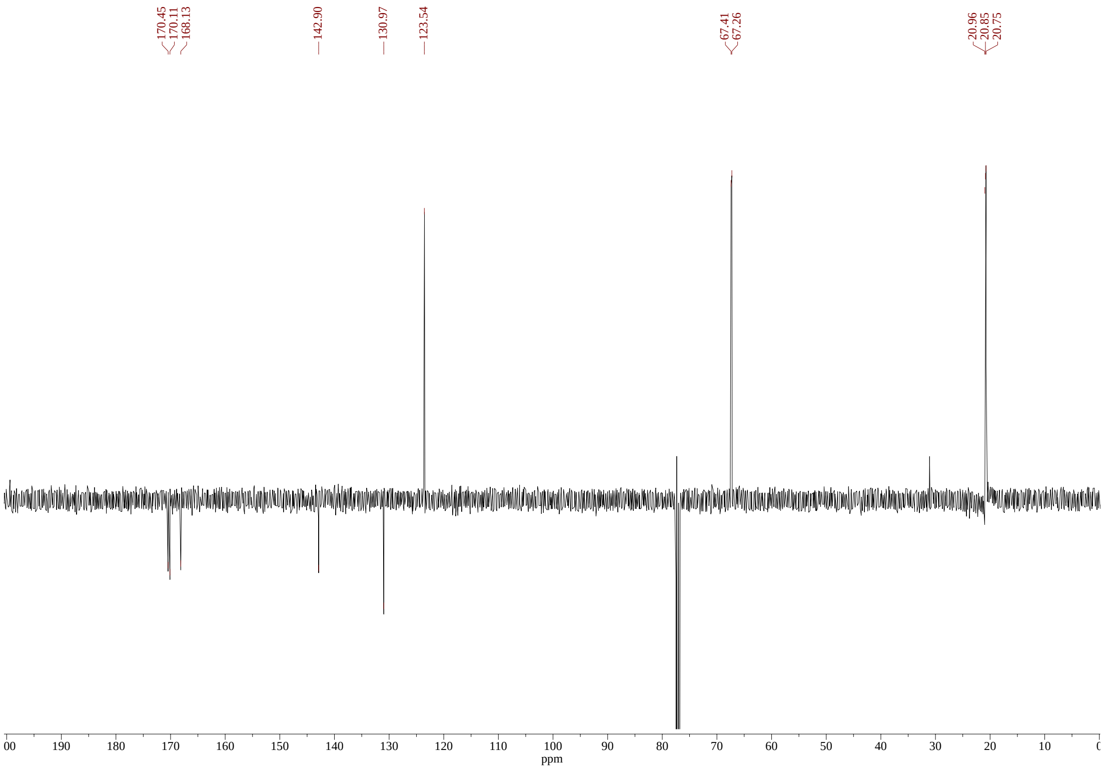

3d^{syn} - ^1H-^1H COSY ($CDCl_3$)

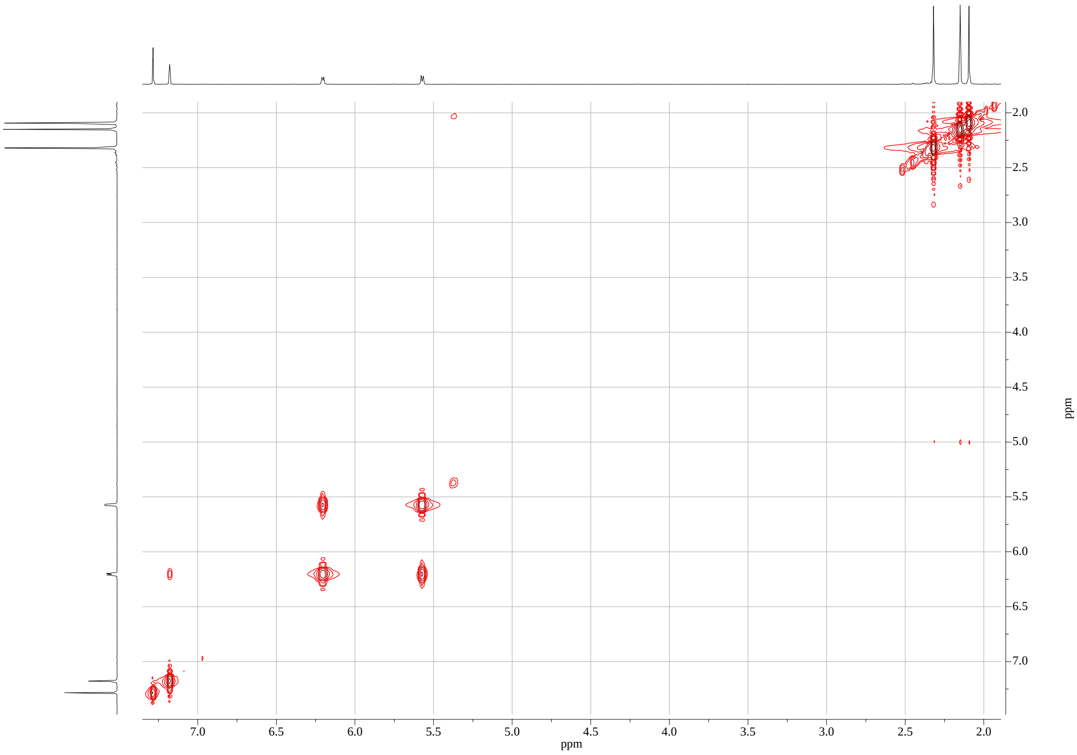

3d^syn^ - ^{1}H-^{13}C HSQCED (CDCl3)

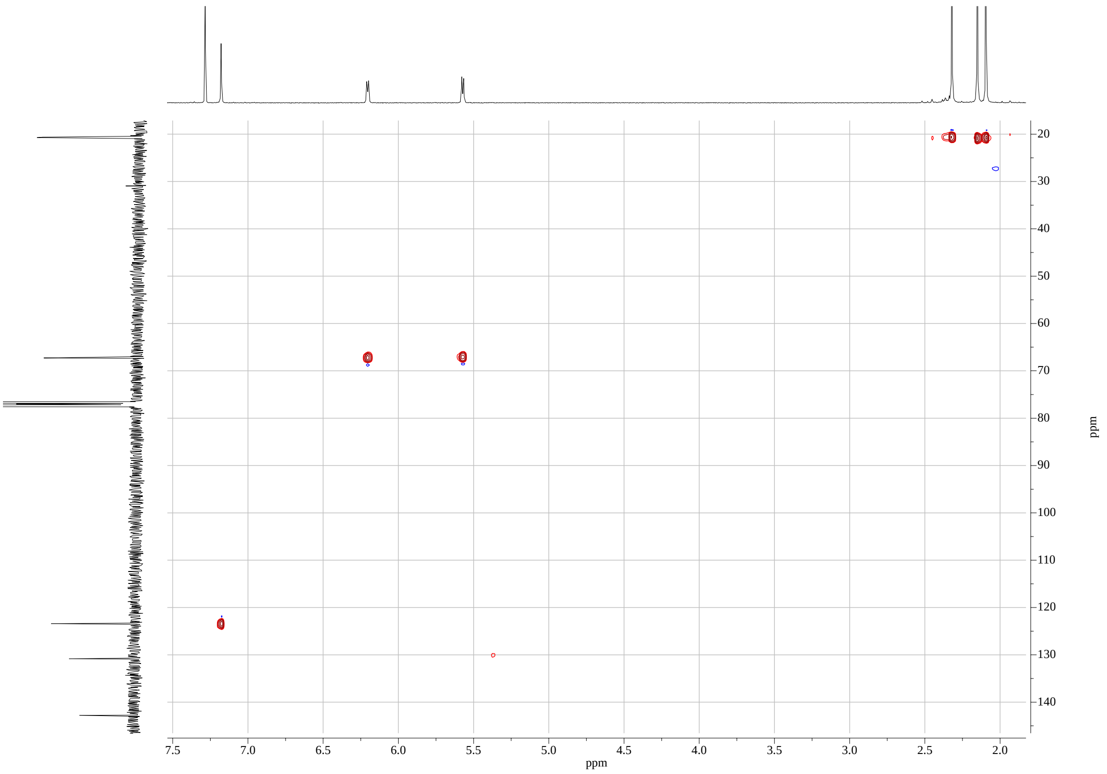

3d^syn^ - ^{1}H-^{13}C HMBC (CDCl3)

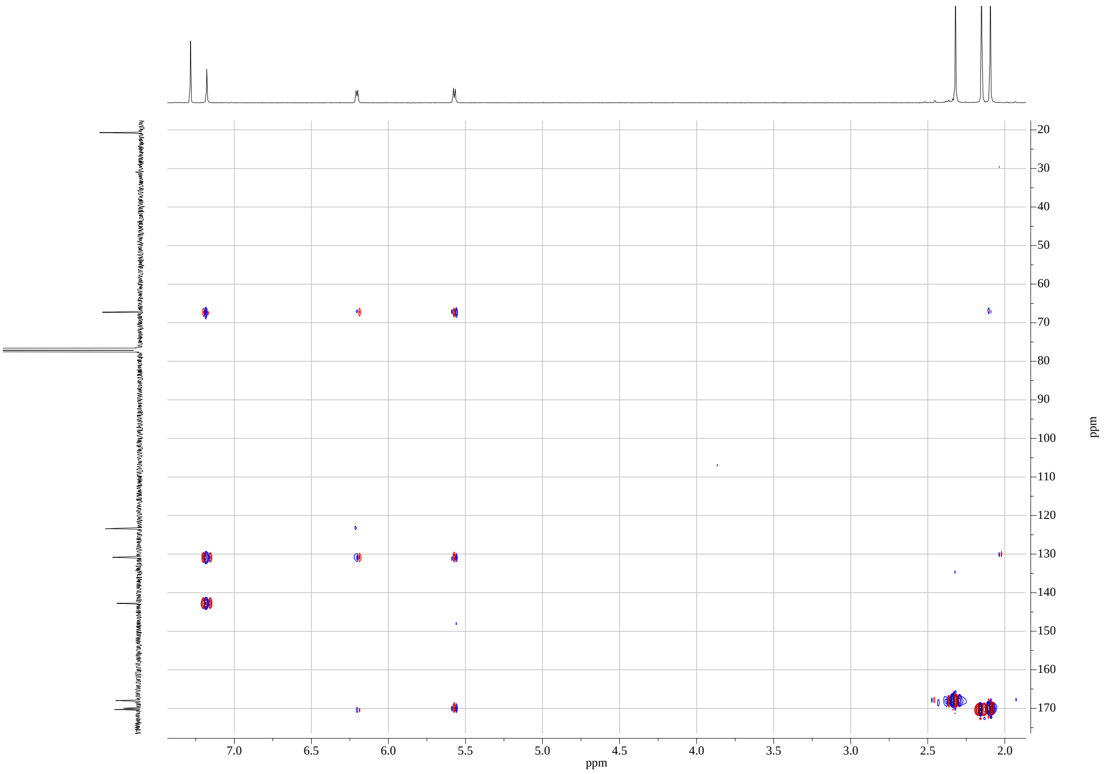

(1α,2α,3β,4β)-1,2,3,4-tetrahydronaphthalene-1,2,3,4,6-pentayl pentaacetate (3d^{anti})

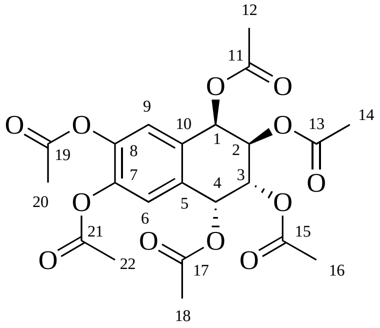

3d^{anti} - ^{1}H NMR (400 MHz, $CDCl_3$)

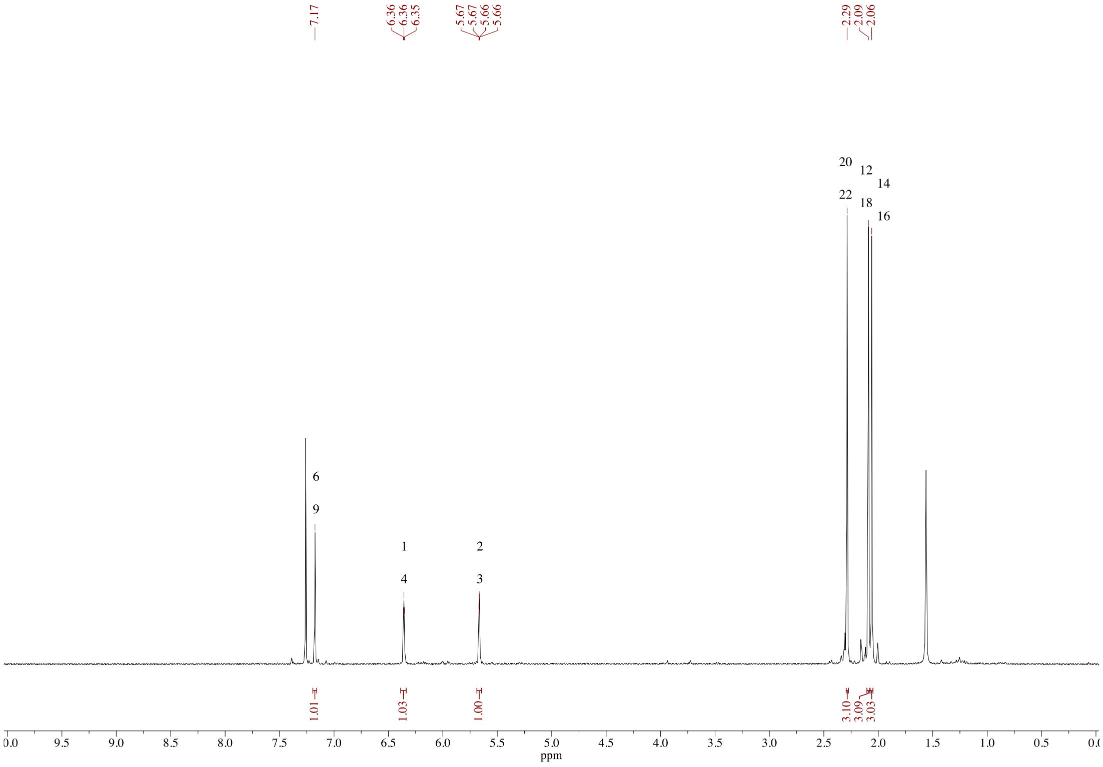

3d^anti^ - ^{13}C NMR (100 MHz, CDCl$_3$)

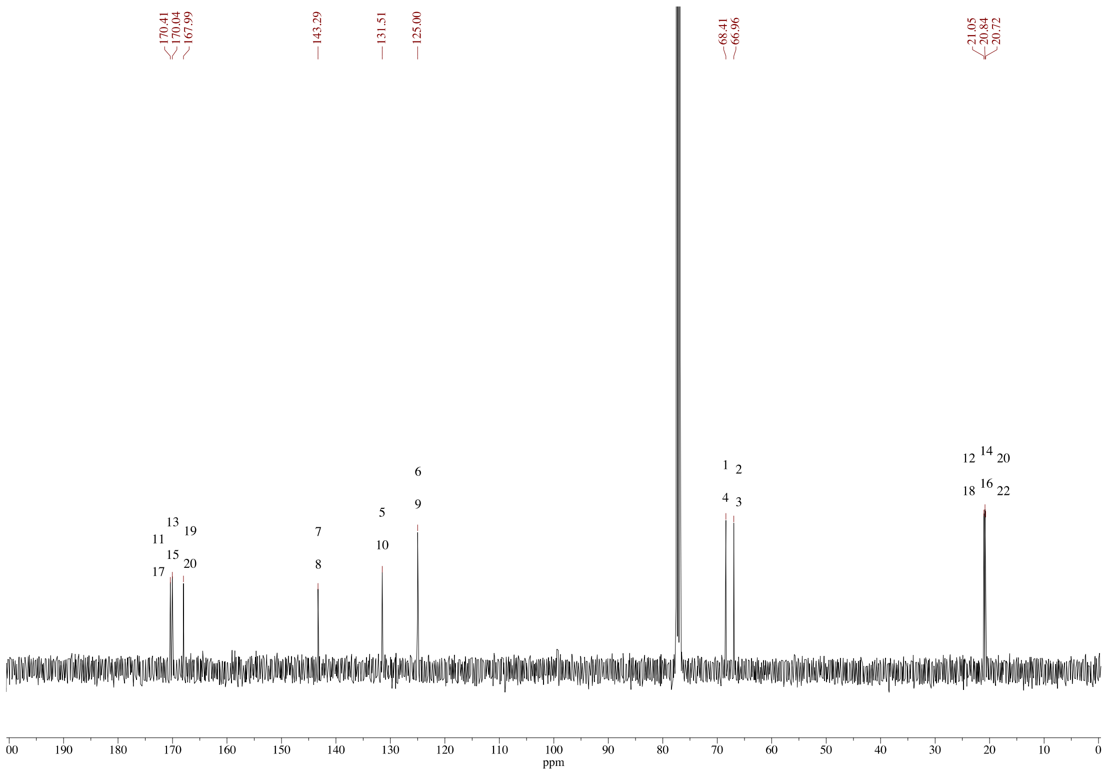

3d^anti^ - DEPT (CDCl$_3$)

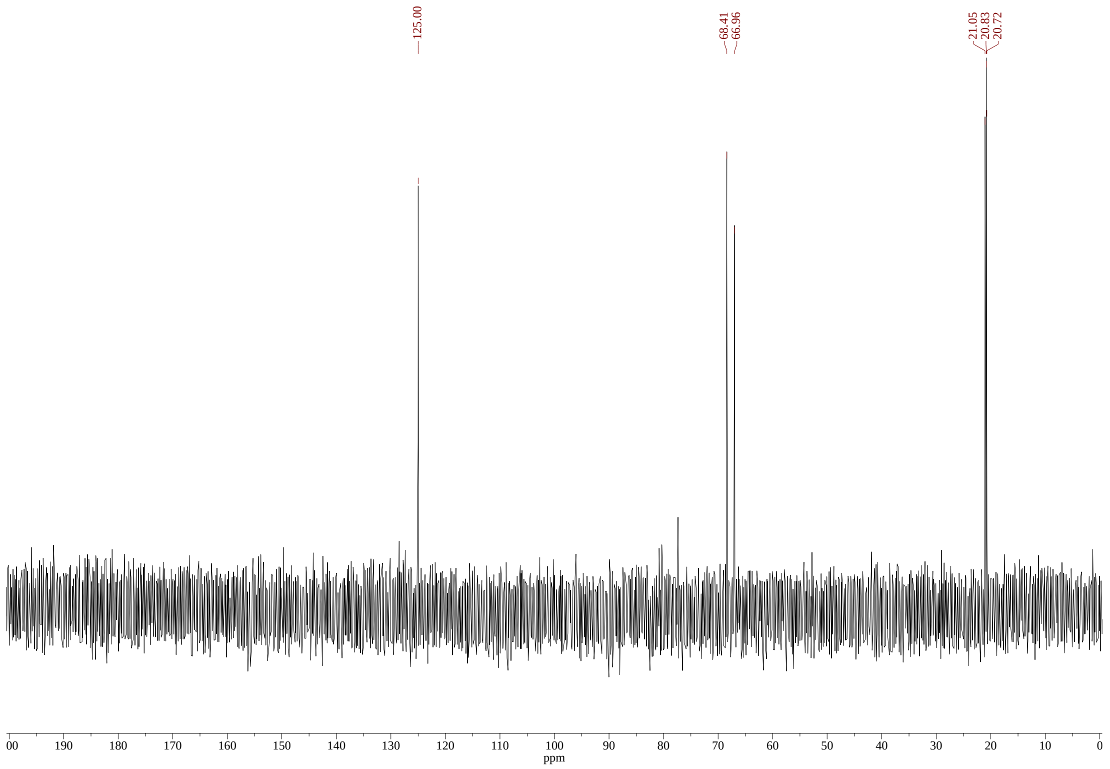

3d^anti^ - DEPTQ ($CDCl_3$)

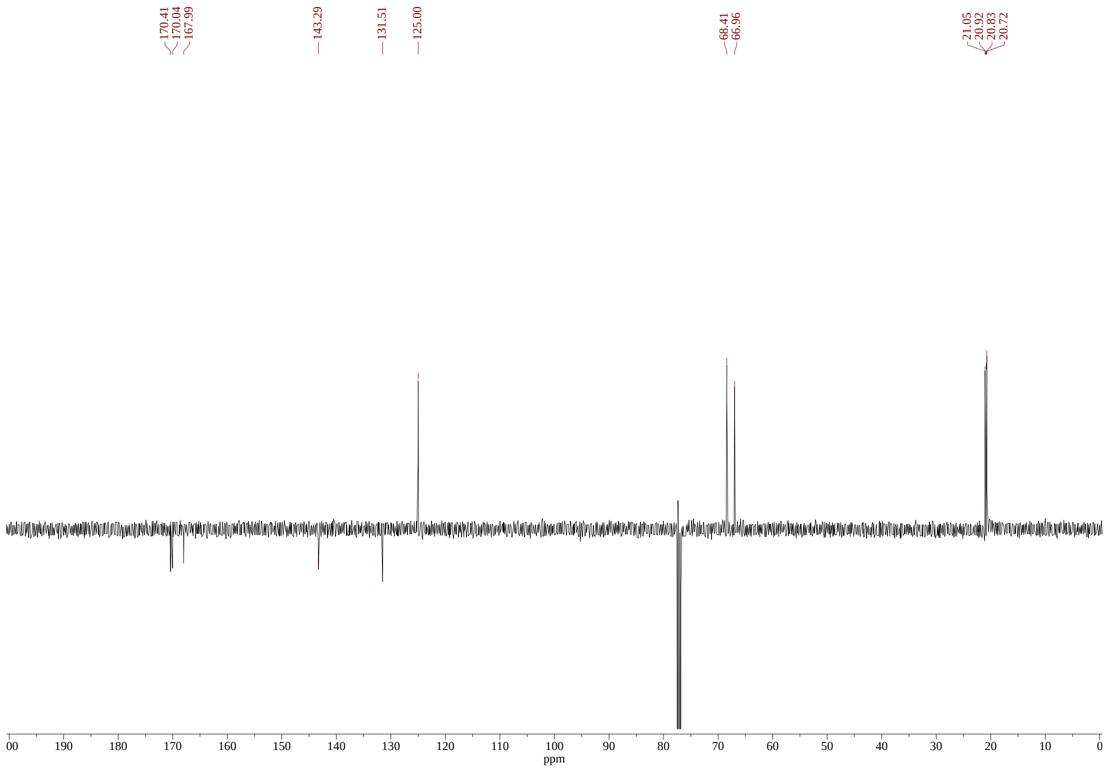

3d^anti^ - ^{1}H-^{1}H COSY ($CDCl_3$)

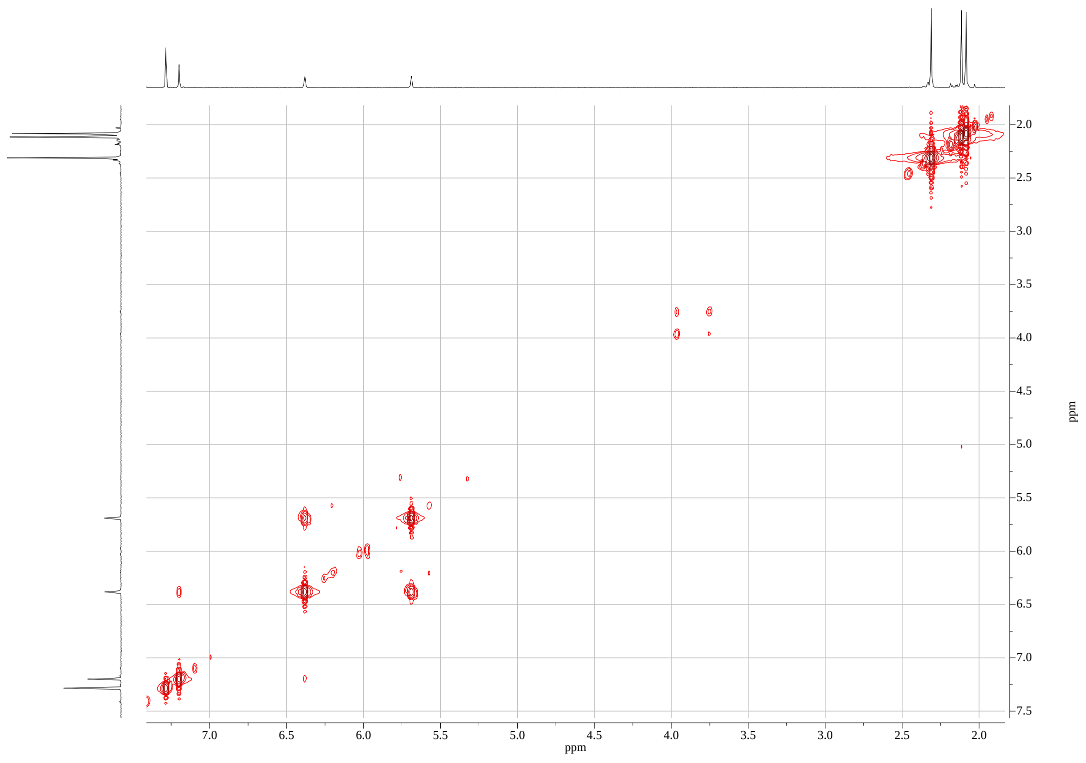

3d[anti] - ^{1}H-^{13}C HSQCED (CDCl$_3$)

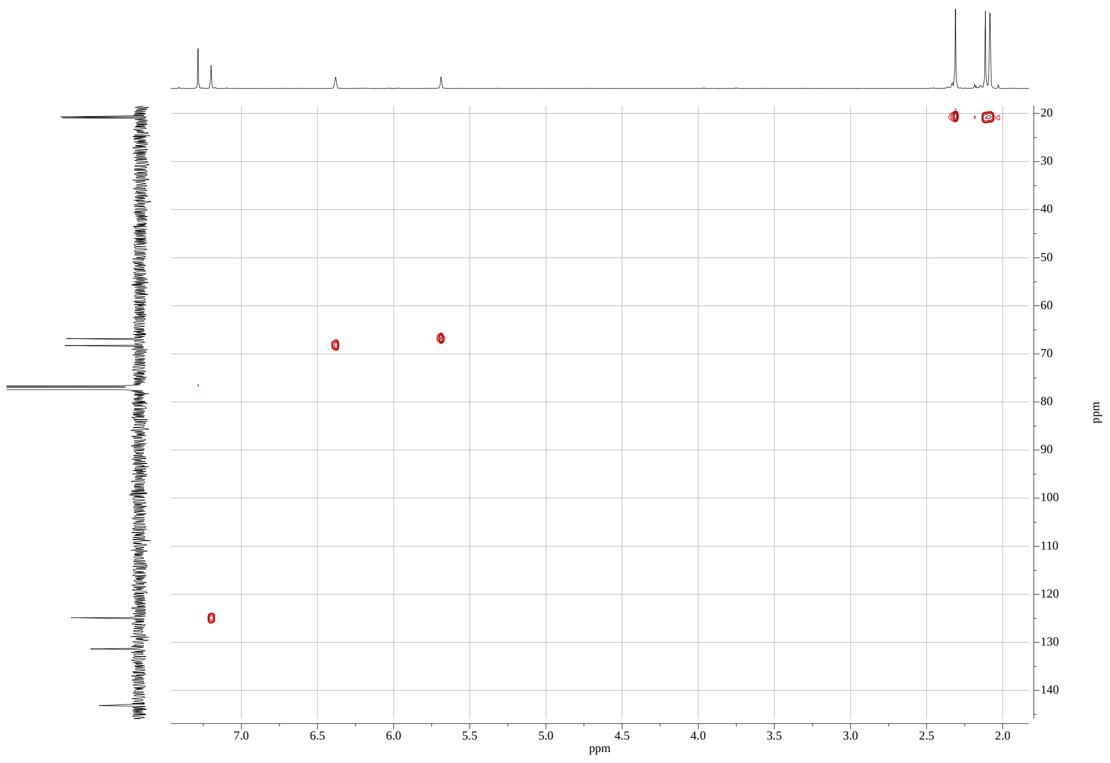

3d[anti] - ^{1}H-^{13}C HMBC (CDCl$_3$)

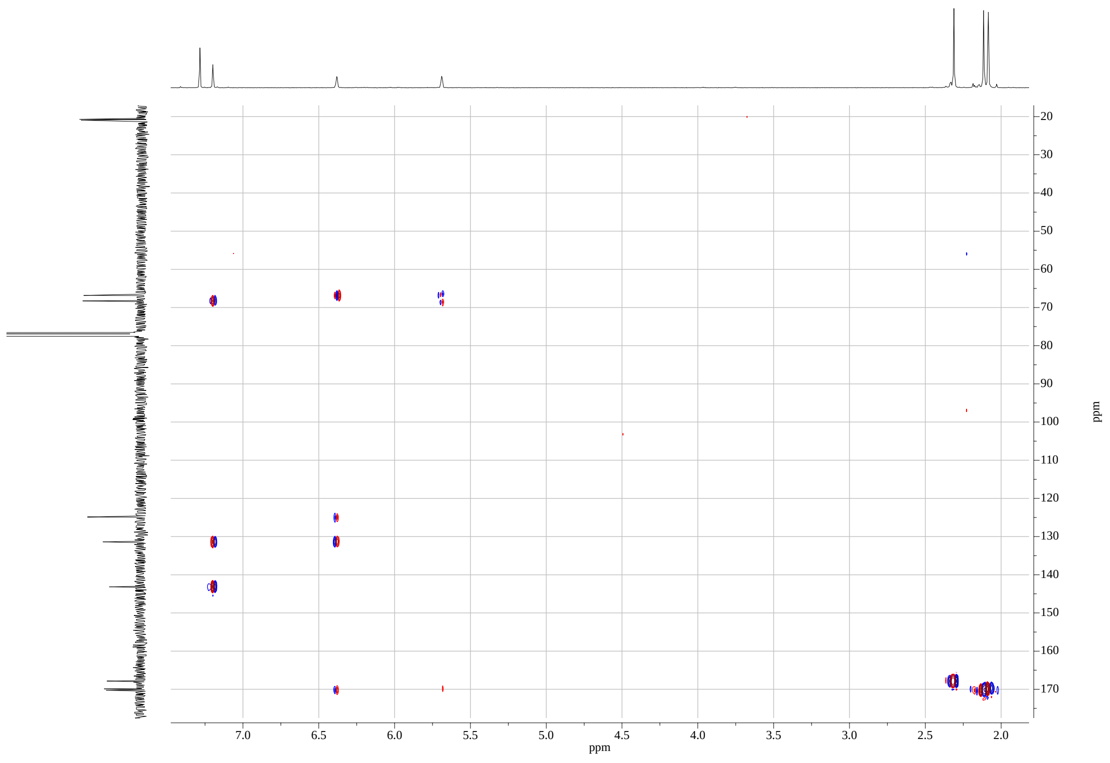

(1α,2α, 3α,4α)-6-nitro-1,2,3,4-tetrahydronaphthalene-1,2,3,4-tetrayl tetraacetate (3i^{syn})

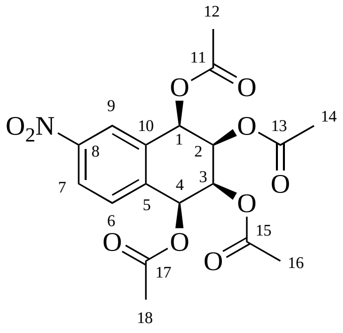

3i^{syn} - ^{1}H NMR (400 MHz, $CDCl_3$)

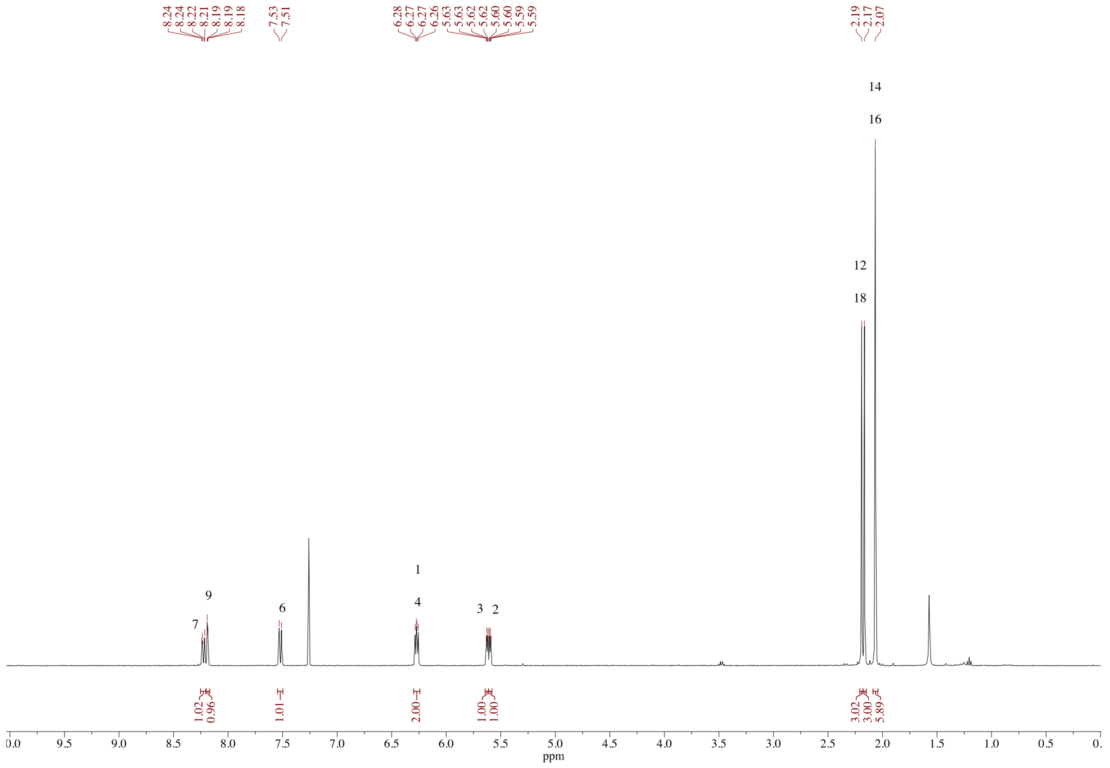

3i^{syn} - ^{13}C NMR (100 MHz, $CDCl_3$)

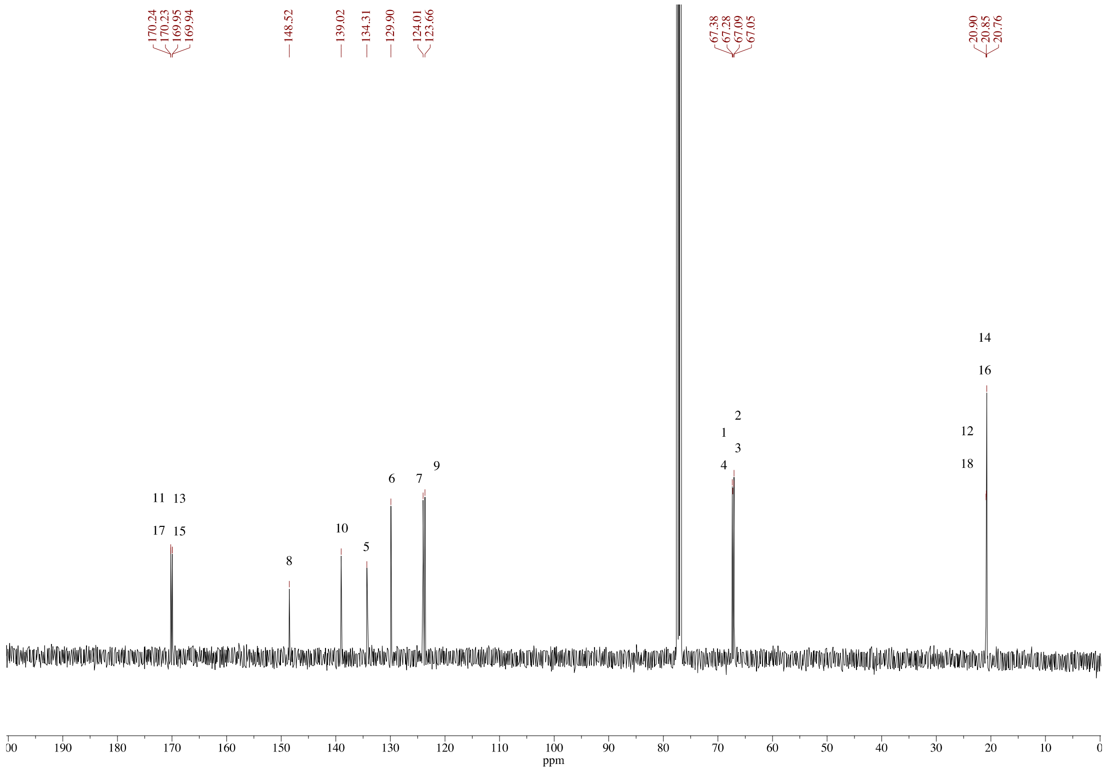

3i^{syn} - DEPT ($CDCl_3$)

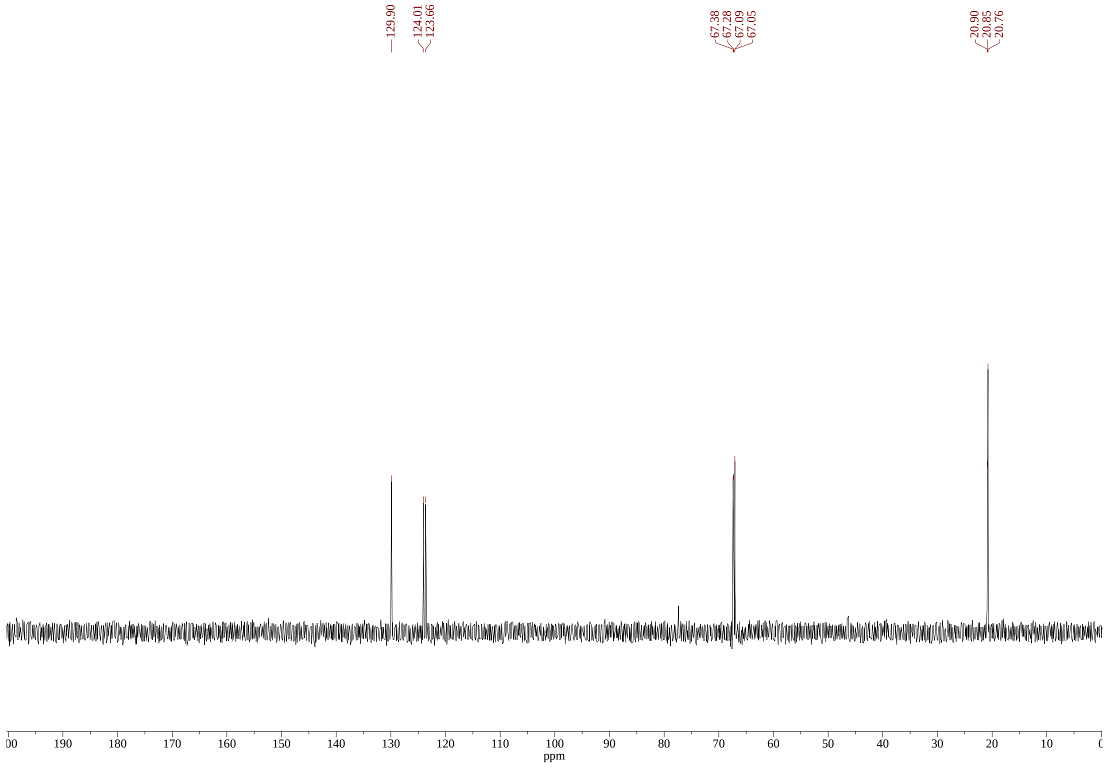

3i^{syn} - DEPTQ ($CDCl_3$)

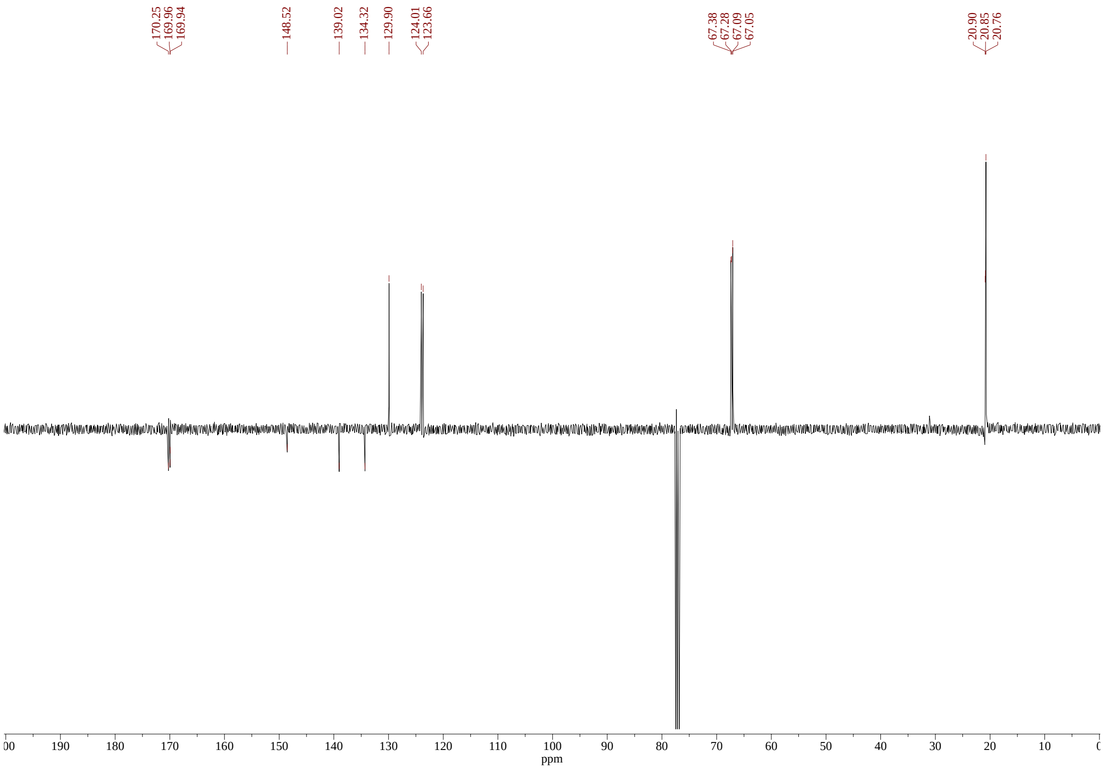

3i^{syn} - ^1H-^1H COSY ($CDCl_3$)

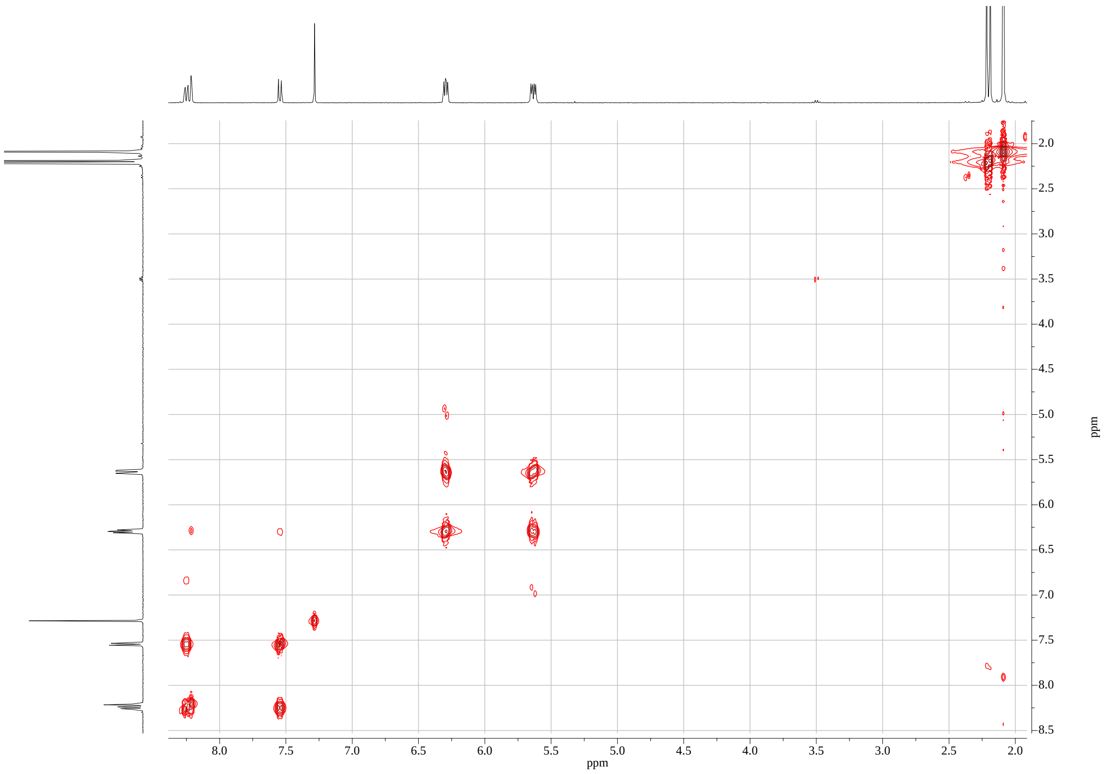

3i^syn^ - ^{1}H-^{13}C HSQCED (CDCl$_3$)

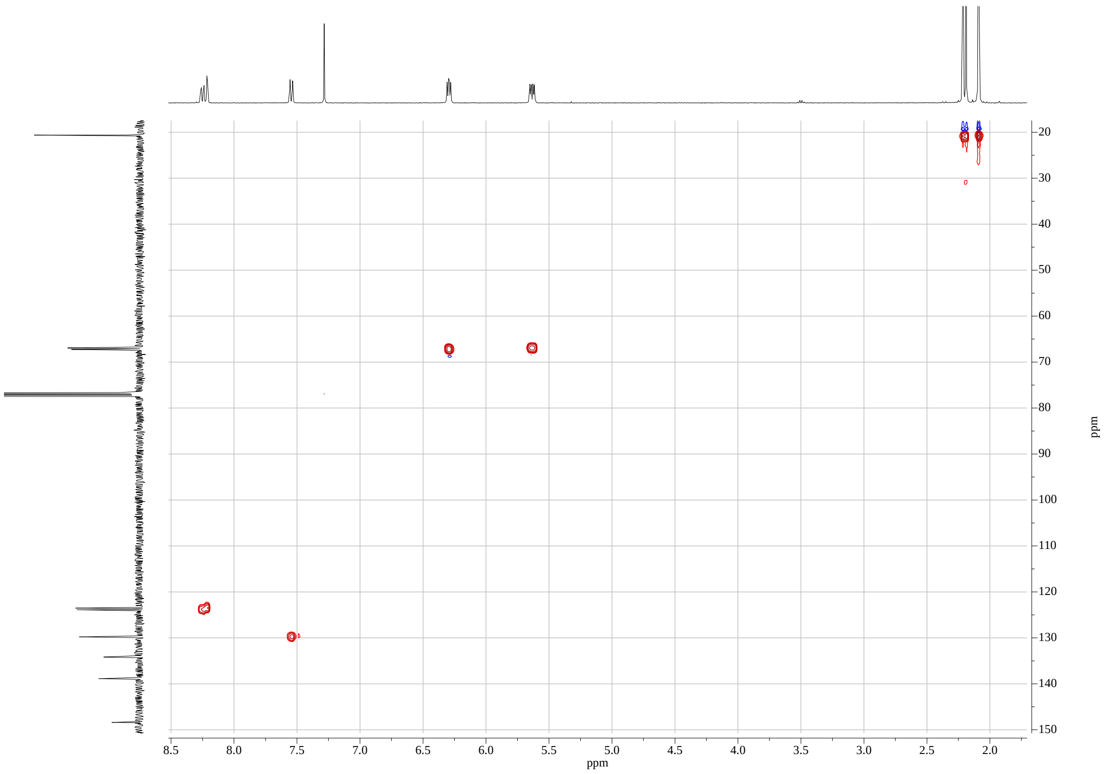

3i^syn^ - ^{1}H-^{13}C HMBC (CDCl$_3$)

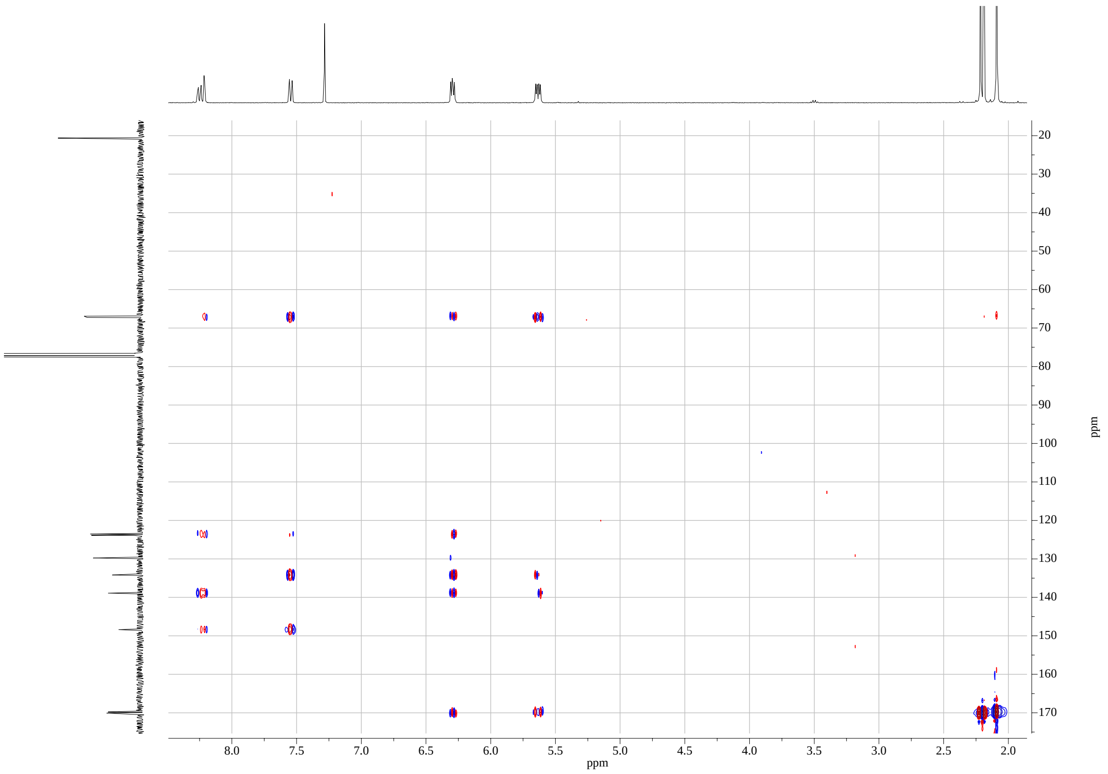

(1α,2α,3β,4β)-6-nitro-1,2,3,4-tetrahydronaphthalene-1,2,3,4-tetrayl tetraacetate (3i^{anti})

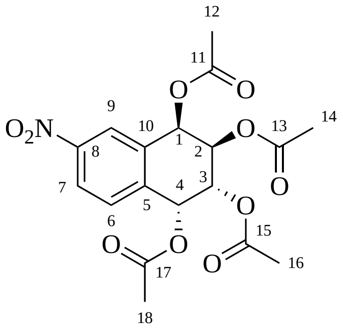

3i^{anti} - ^{1}H NMR (400 MHz, $CDCl_3$)

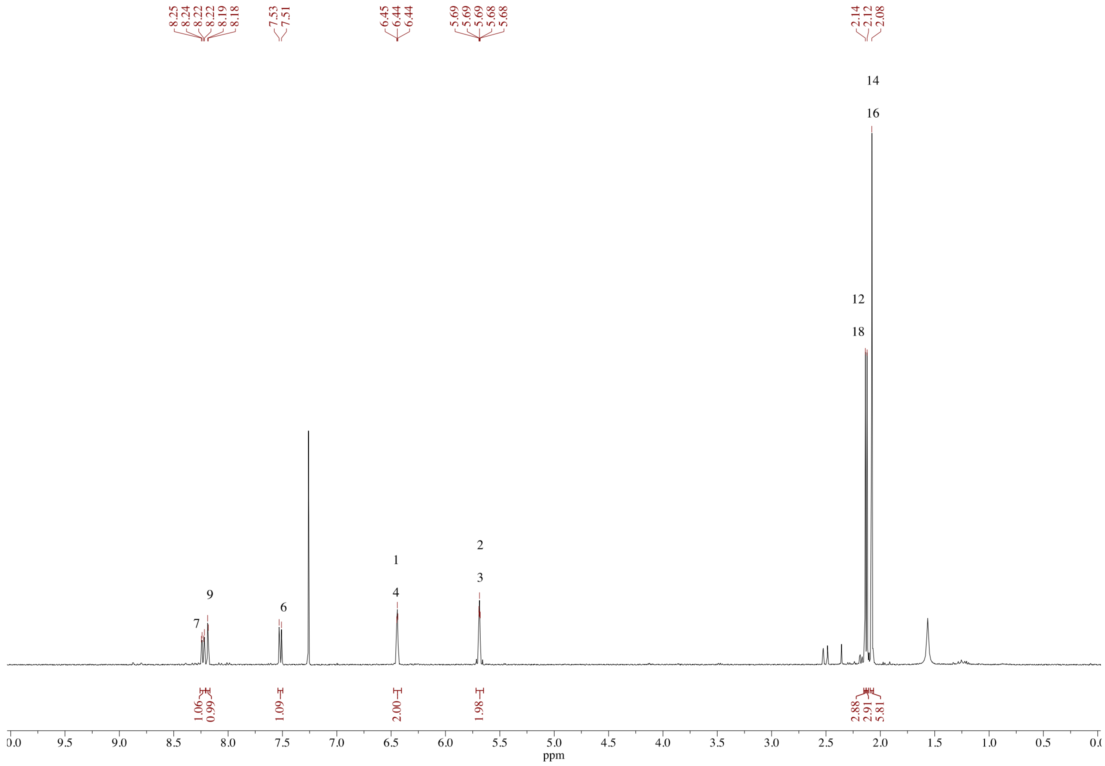

3i[anti] - ^{13}C NMR (100 MHz, $CDCl_3$)

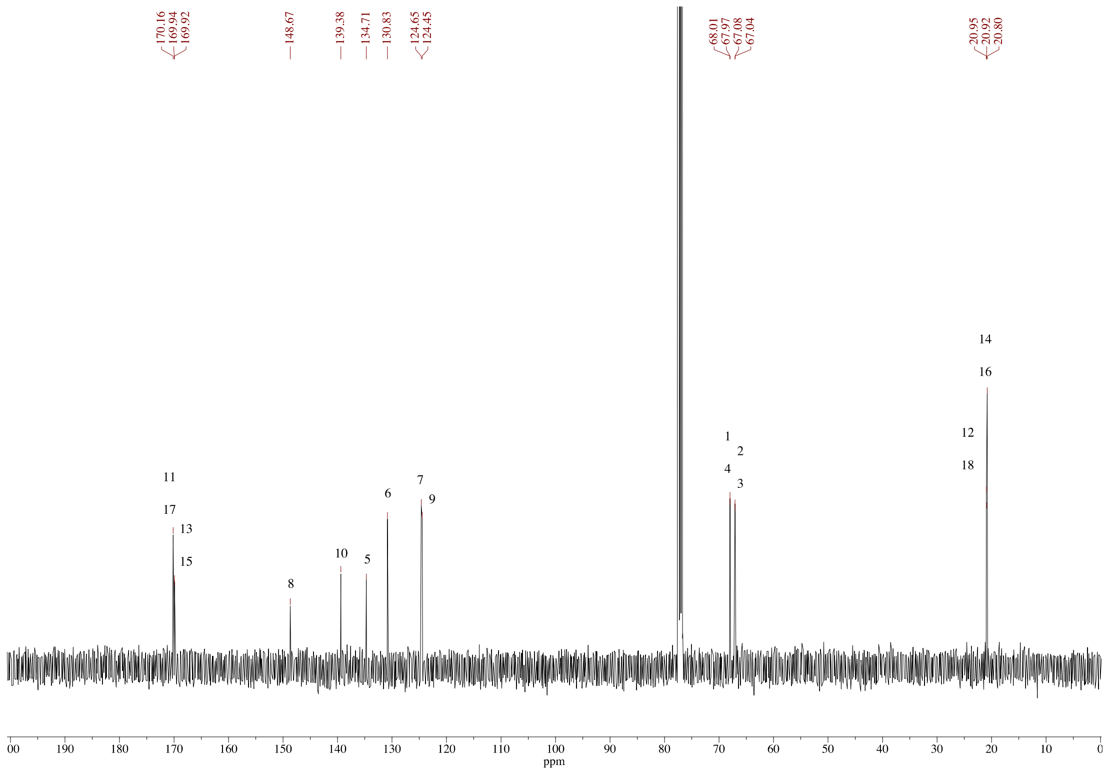

3i[anti] - DEPT ($CDCl_3$)

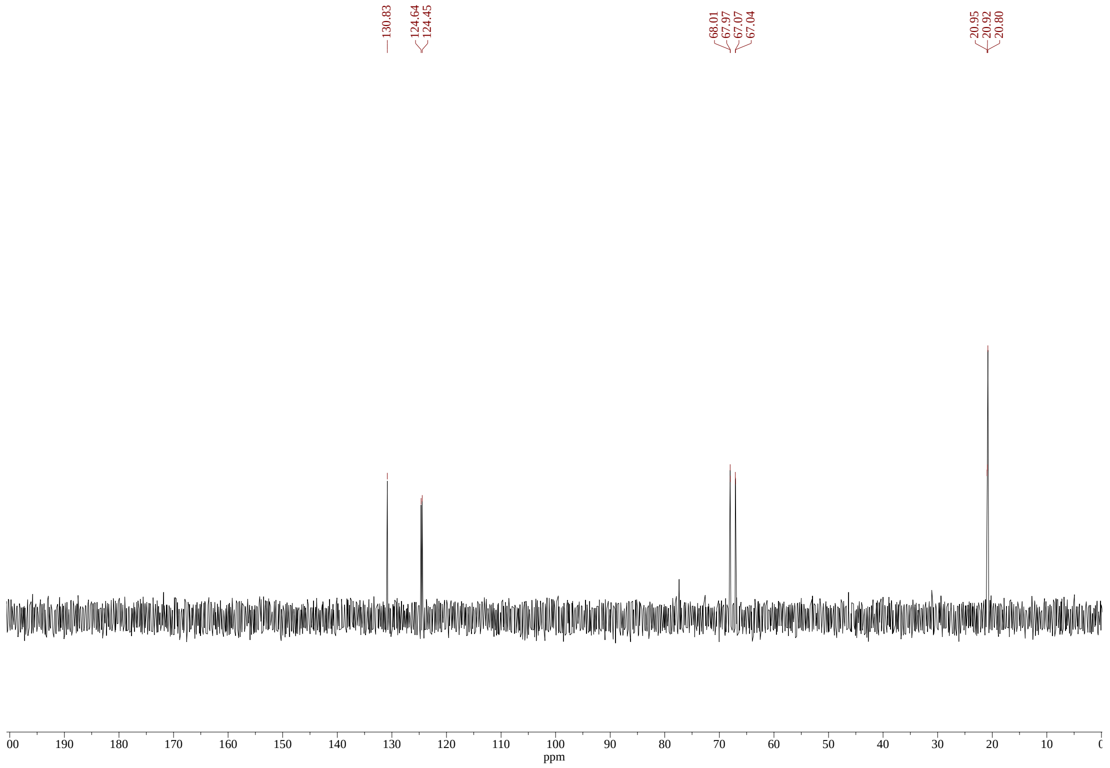

3i^{anti} - DEPTQ ($CDCl_3$)

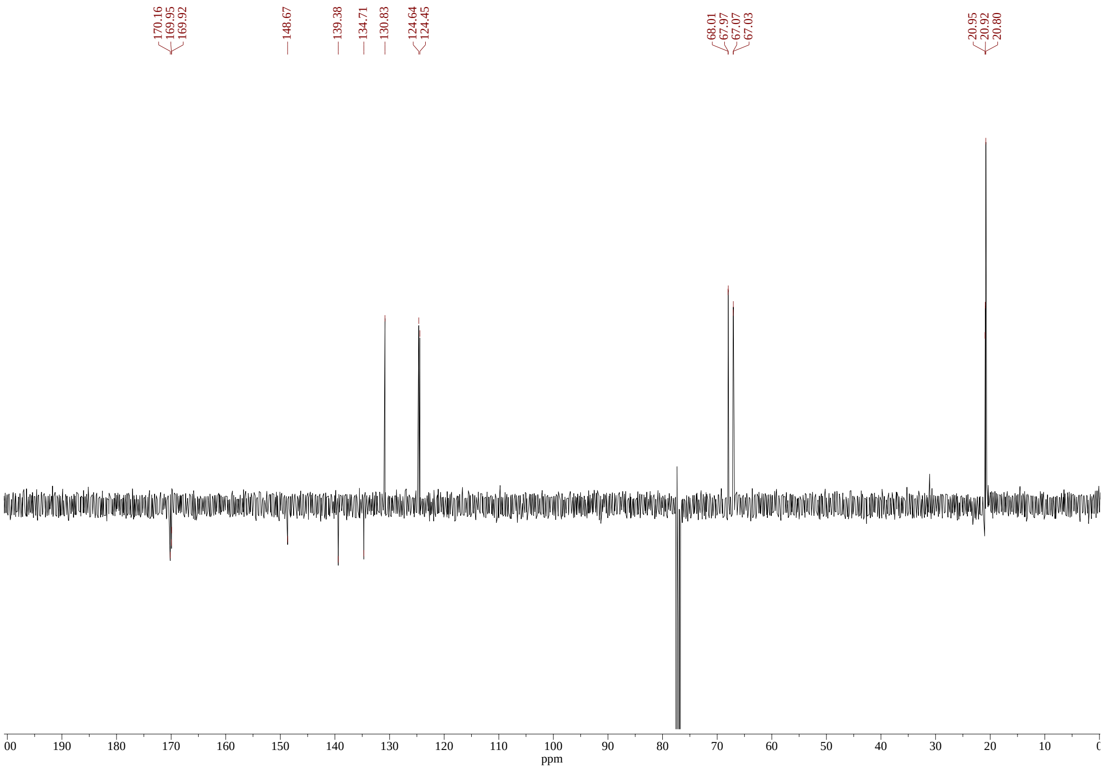

3i^{anti} - ^{1}H-^{1}H COSY ($CDCl_3$)

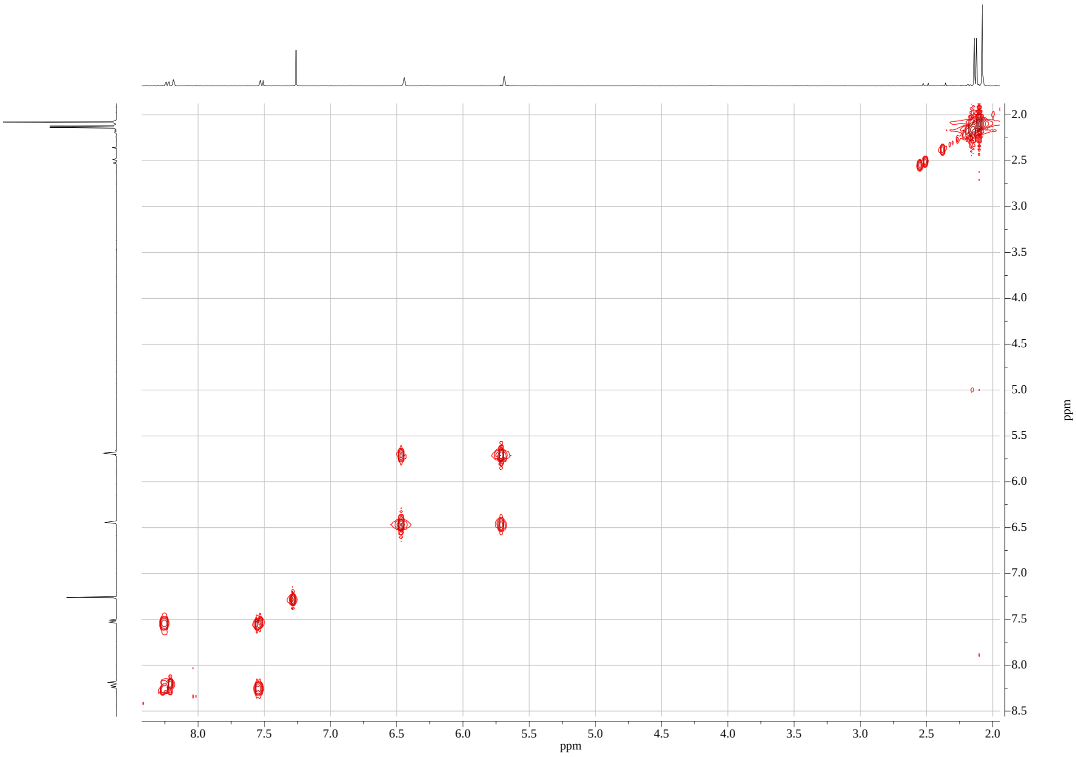

3i[anti] - ^{1}H-^{13}C HSQCED (CDCl$_3$)

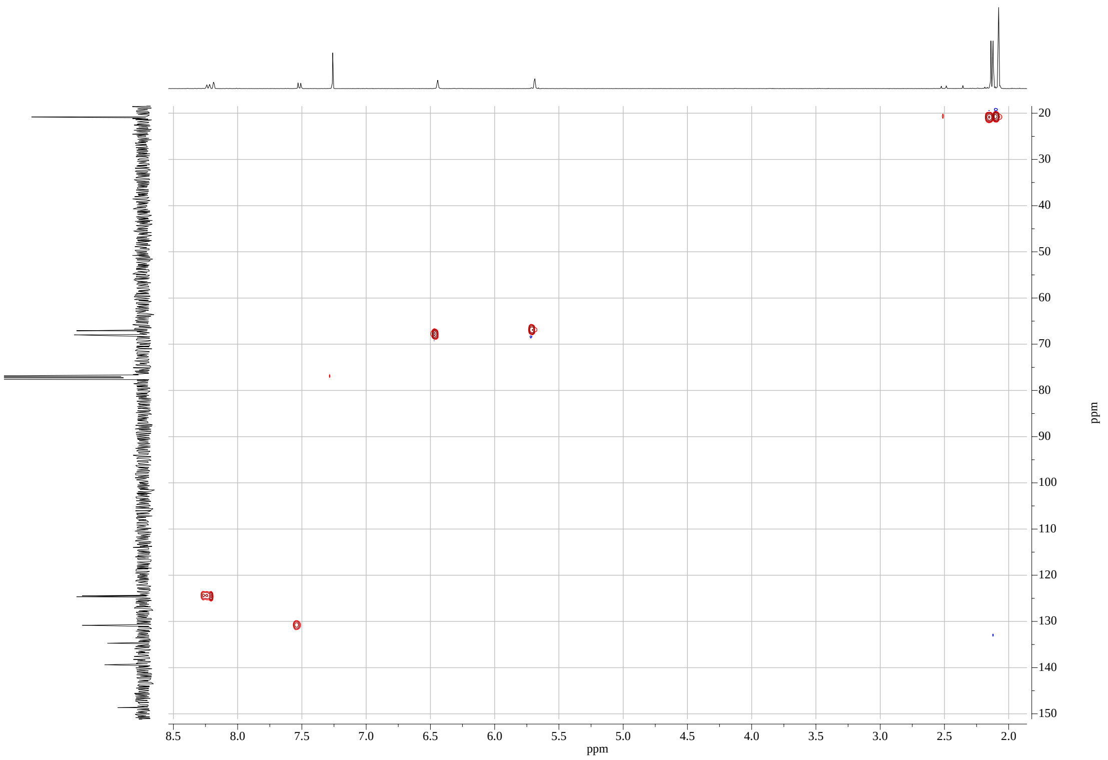

3i[anti] - ^{1}H-^{13}C HMBC (CDCl$_3$)

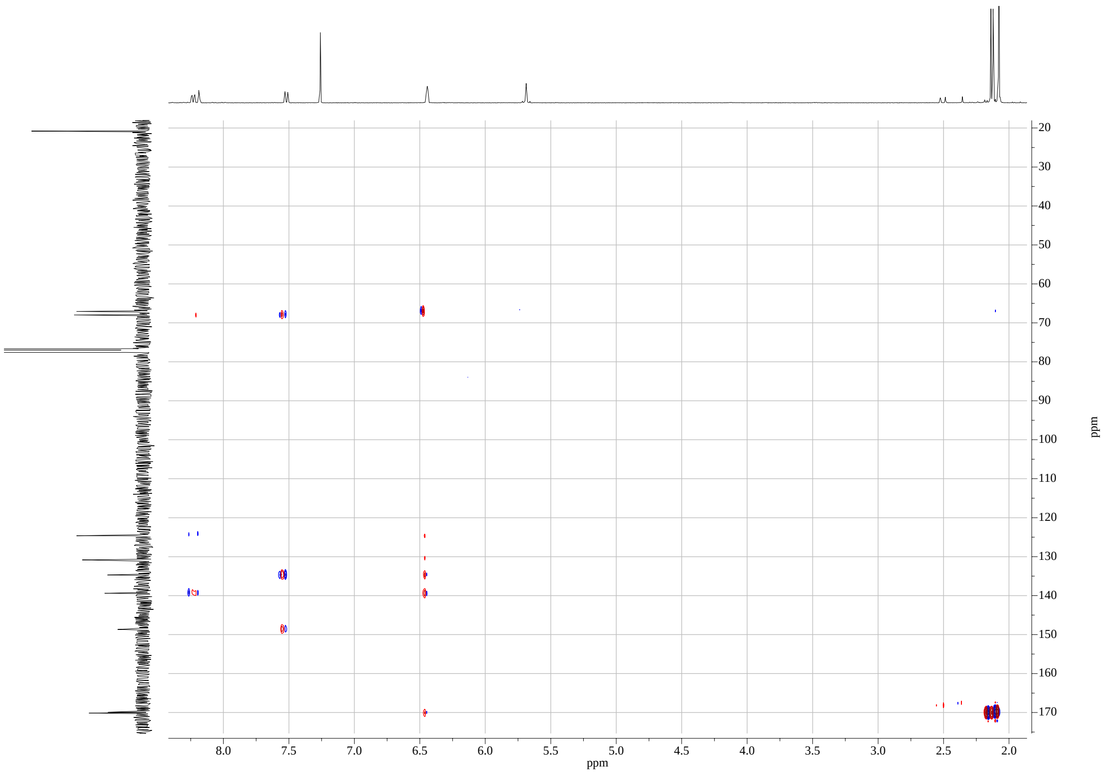

(1α,2α,3α,4α)-6-acetyl-1,2,3,4-tetrahydronaphthalene-1,2,3,4-tetrayl tetraacetate ($3k^{syn}$)

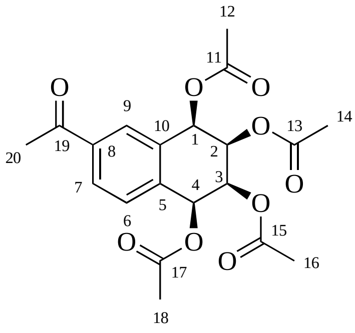

$3k^{syn}$ - ^{1}H NMR (400 MHz, $CDCl_3$)

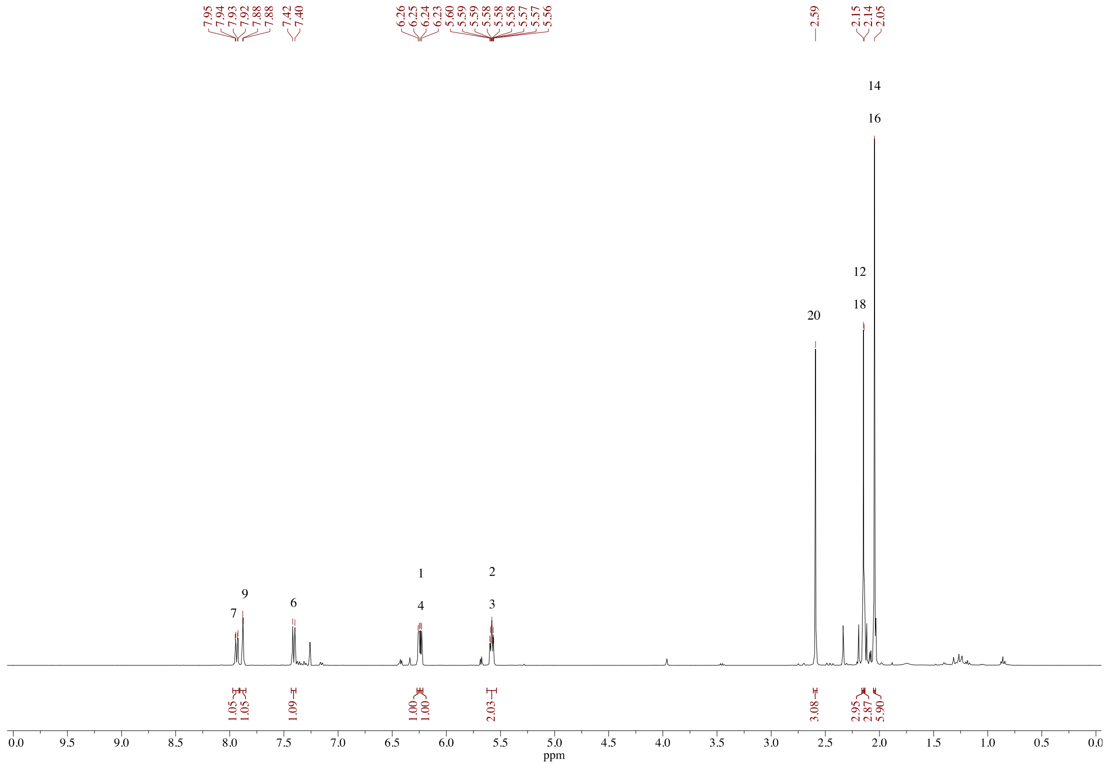

3k^syn - ^{13}C NMR (100 MHz, $CDCl_3$)

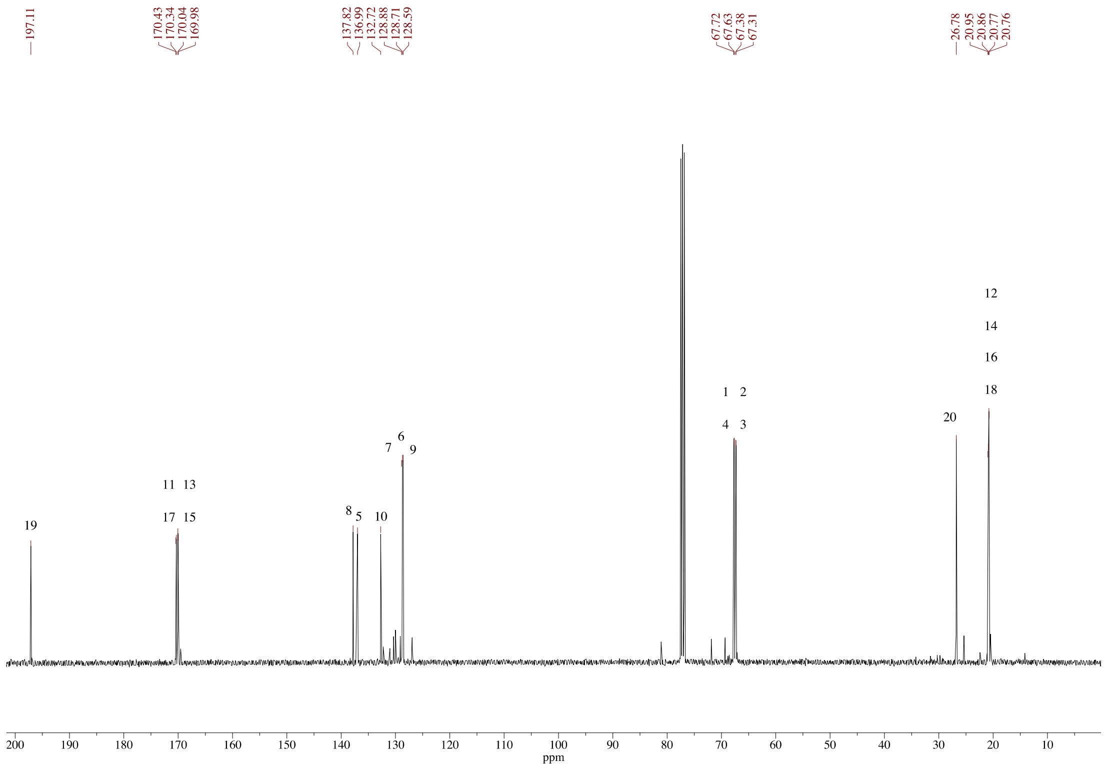

3k^syn - DEPT ($CDCl_3$)

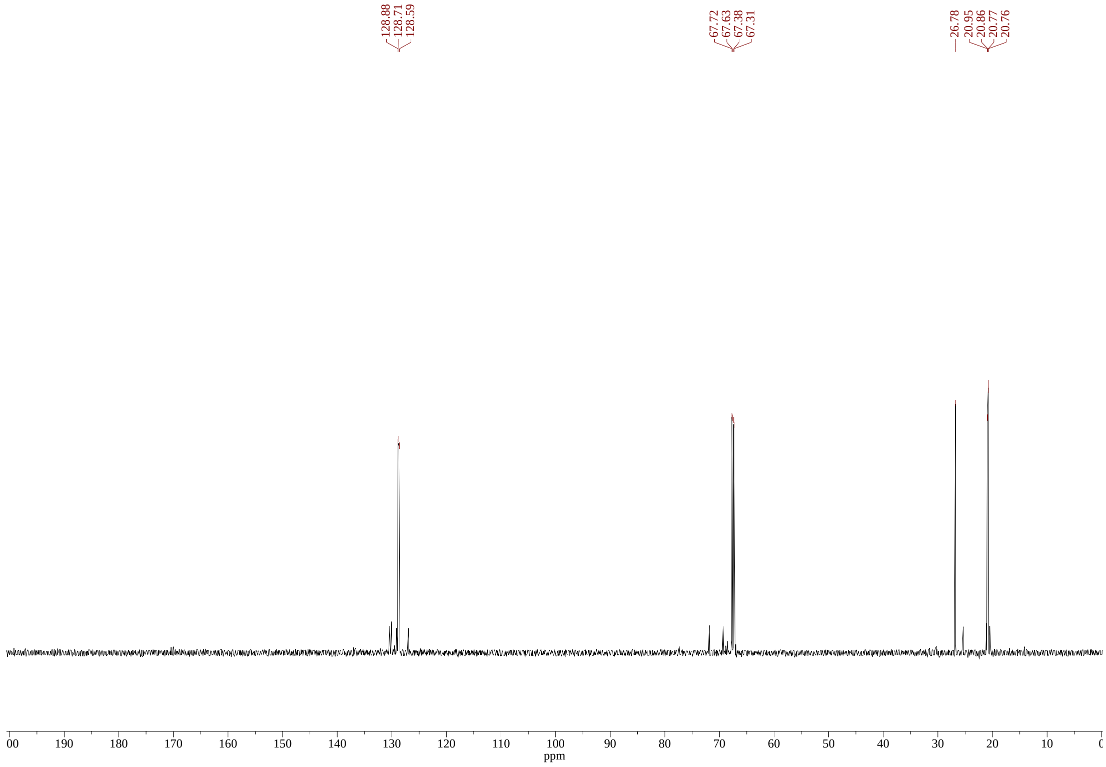

3k^{syn} - DEPTQ ($CDCl_3$)

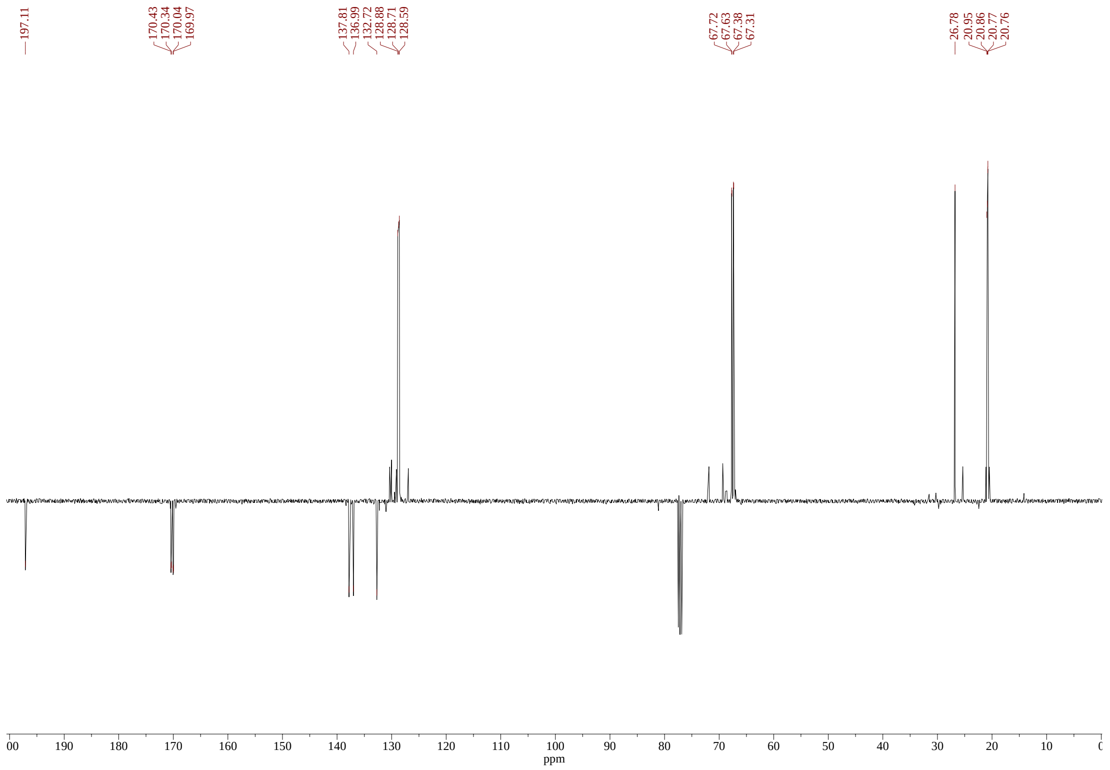

3k^{syn} - ^{1}H-^{1}H COSY ($CDCl_3$)

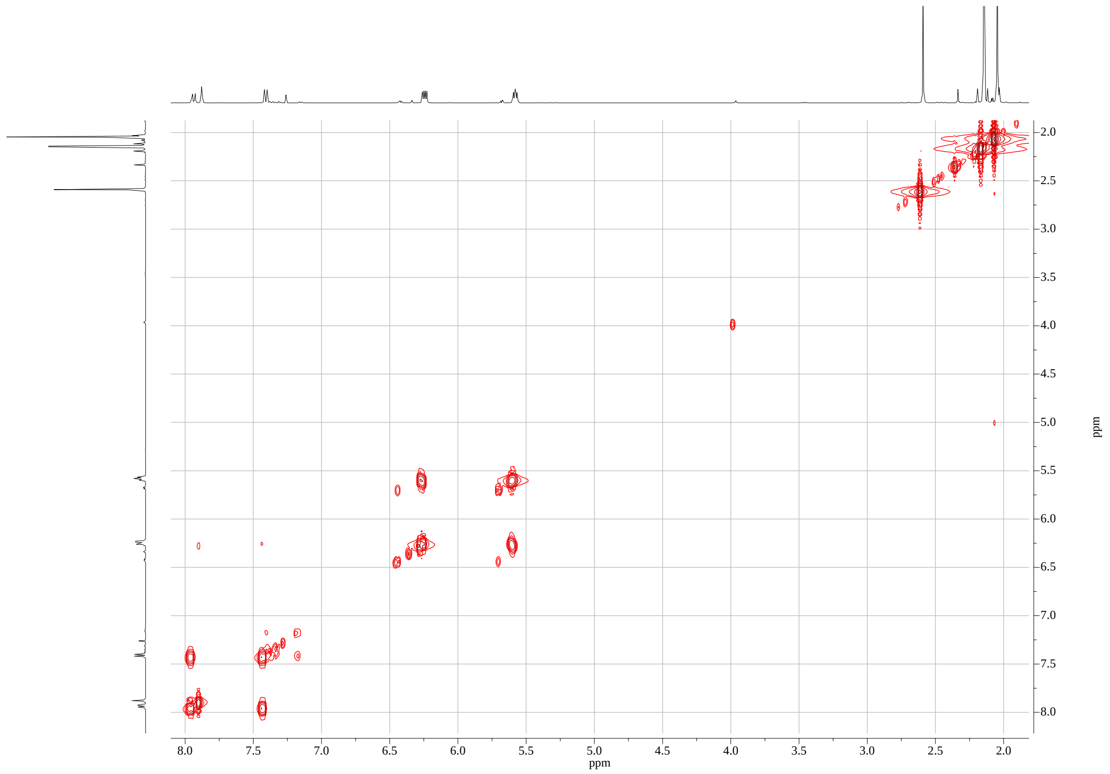

3k^{syn} - ^{1}H-^{13}C HSQCED ($CDCl_3$)

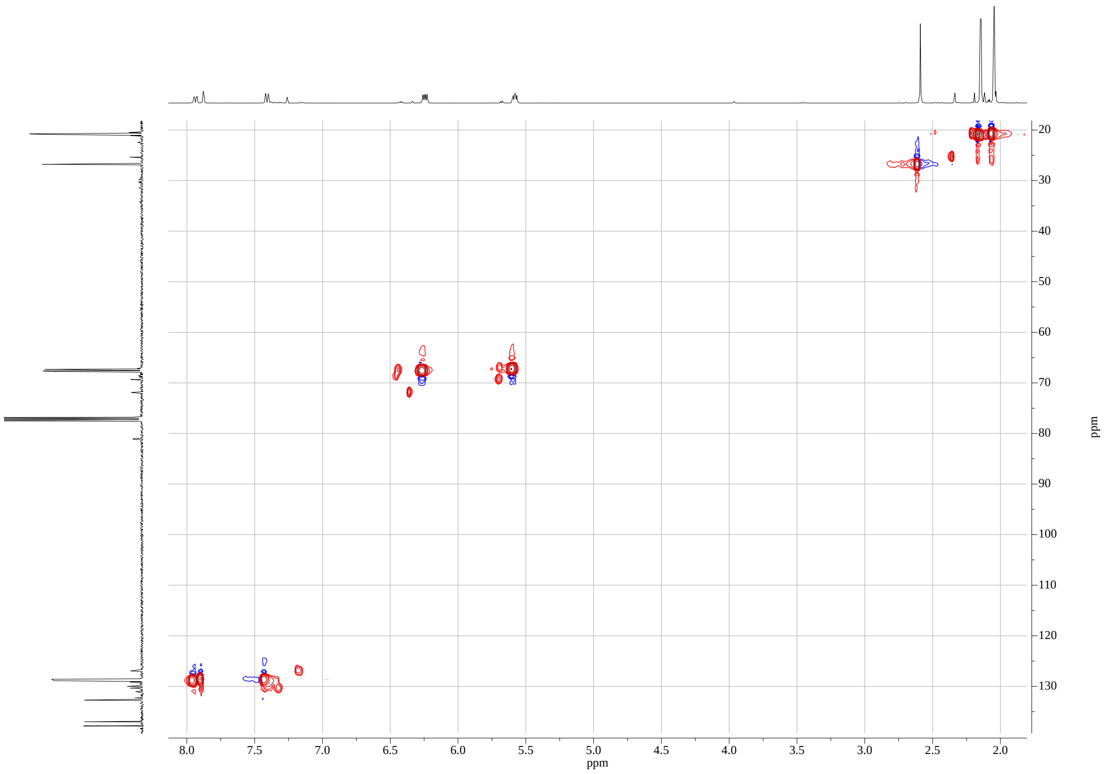

3k^{syn} - ^{1}H-^{13}C HMBC ($CDCl_3$)

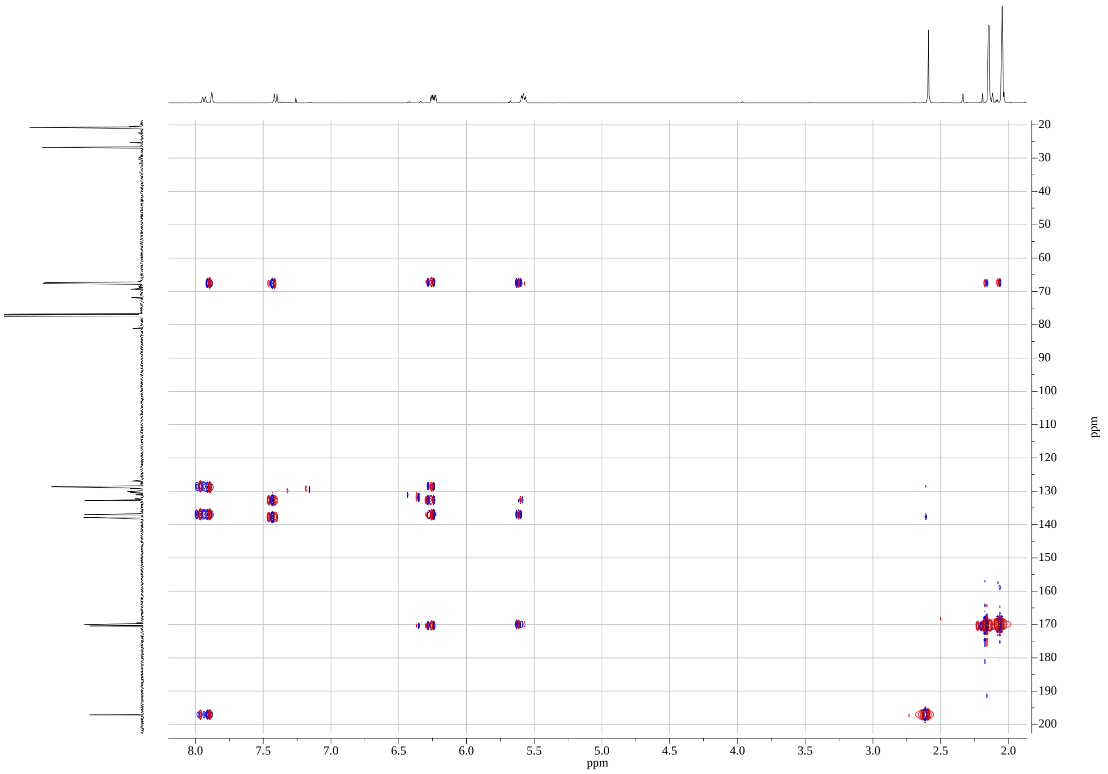

(1α,2α,3β,4β)-6-acetyl-1,2,3,4-tetrahydronaphthalene-1,2,3,4-tetrayl tetraacetate (3k[anti])

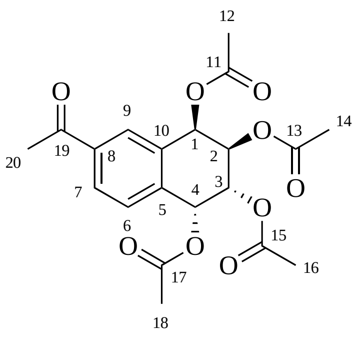

3k[anti] - ^{1}H NMR (400 MHz, $CDCl_3$)

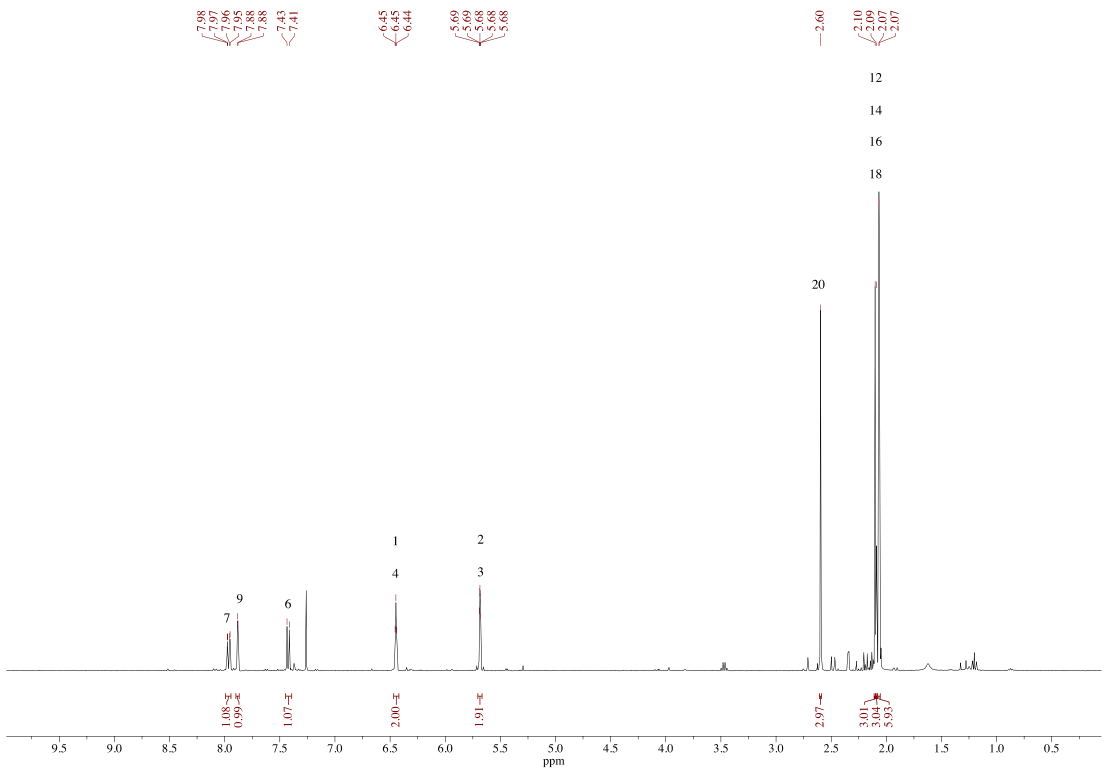

3k[anti] - ^{13}C NMR (100 MHz, $CDCl_3$)

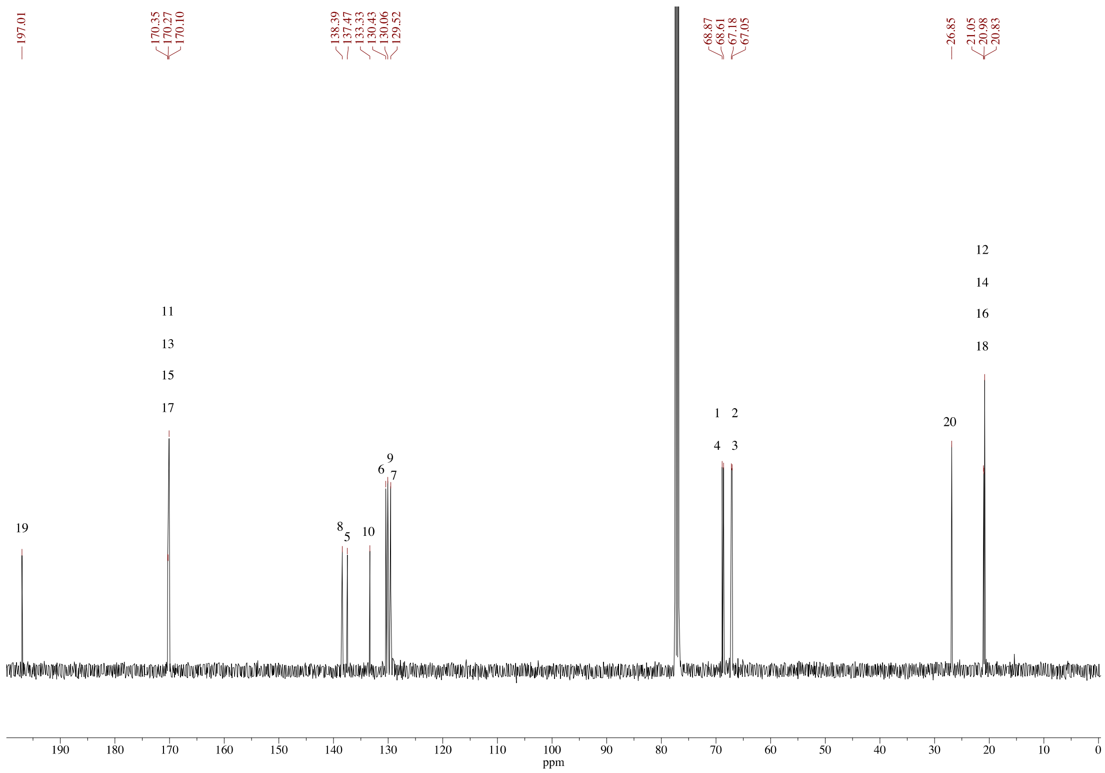

3k[anti] - DEPT ($CDCl_3$)

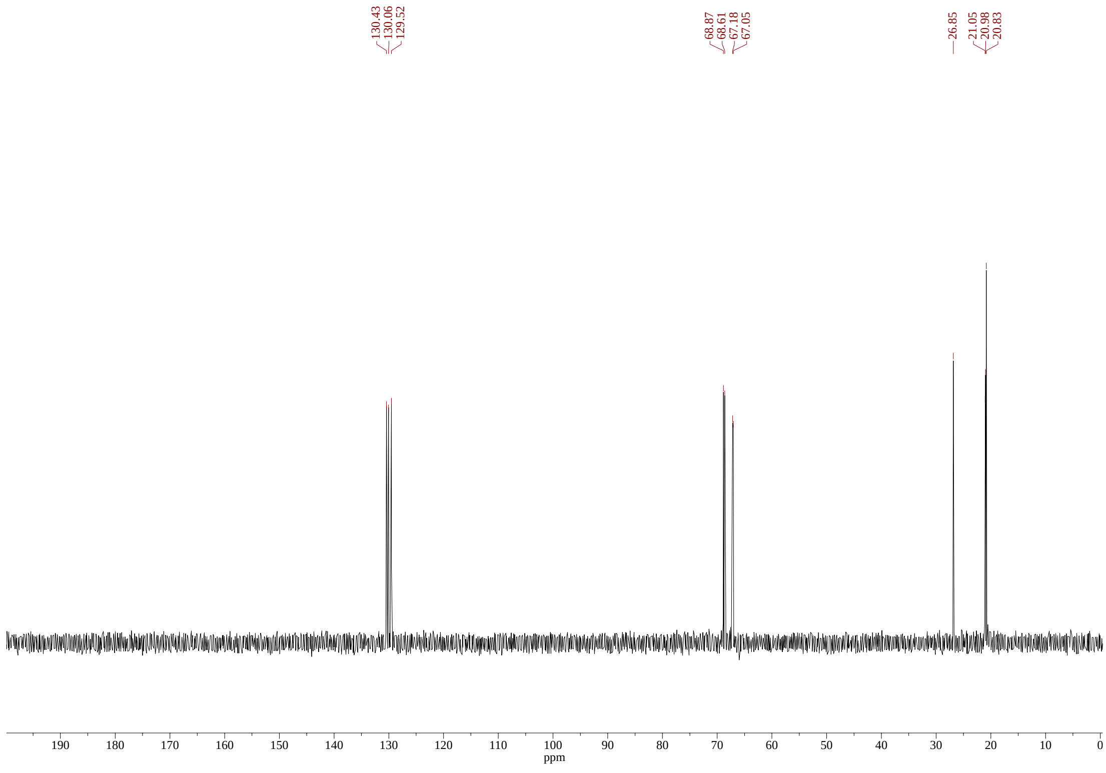

3k^anti - DEPTQ ($CDCl_3$)

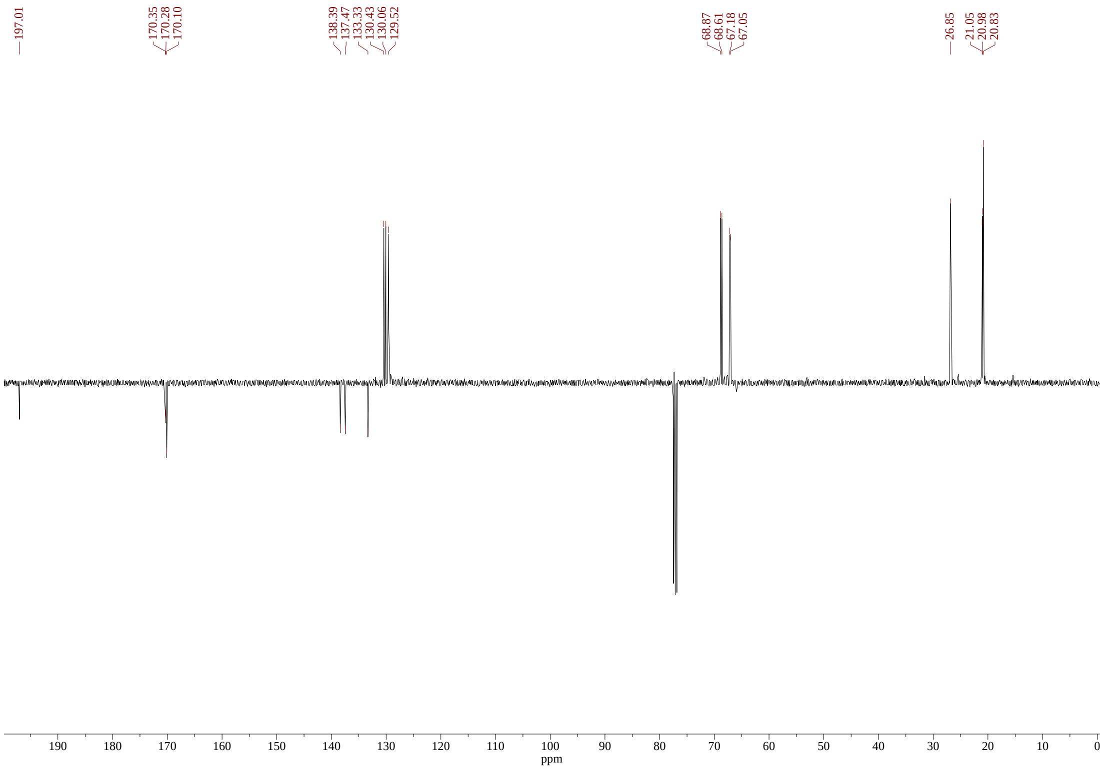

3k^anti - ^1H-^1H COSY ($CDCl_3$)

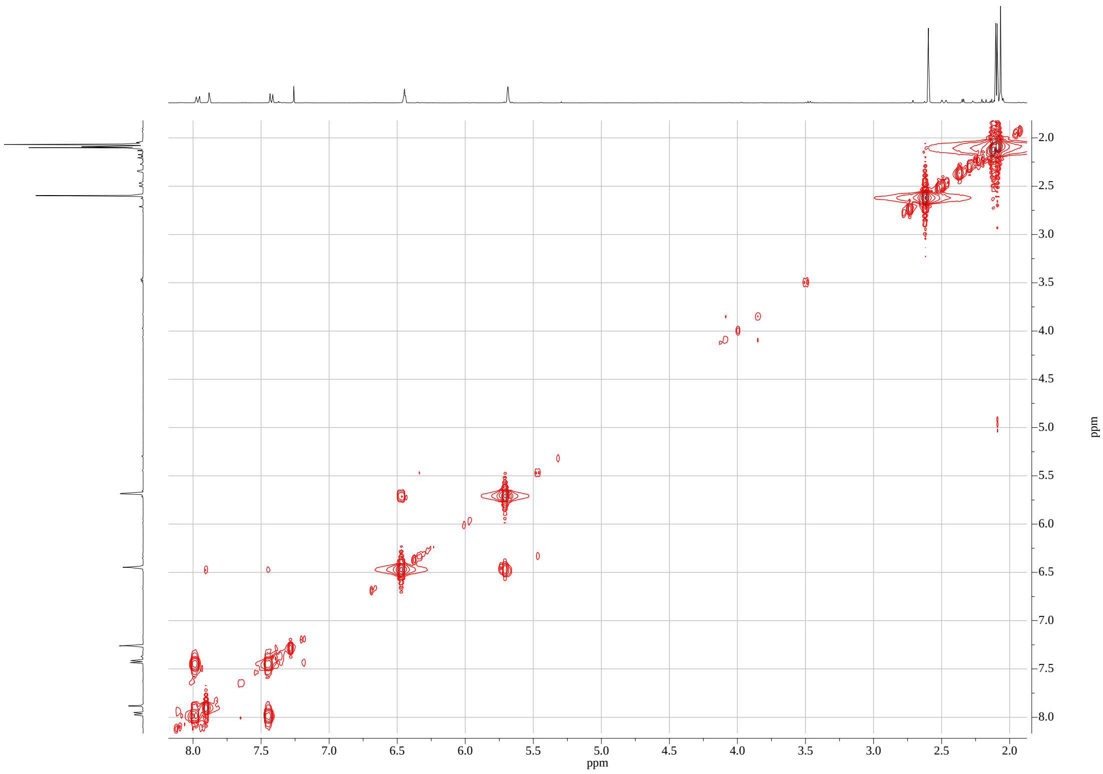

3k^anti - ^{1}H-^{13}C HSQCED ($CDCl_3$)

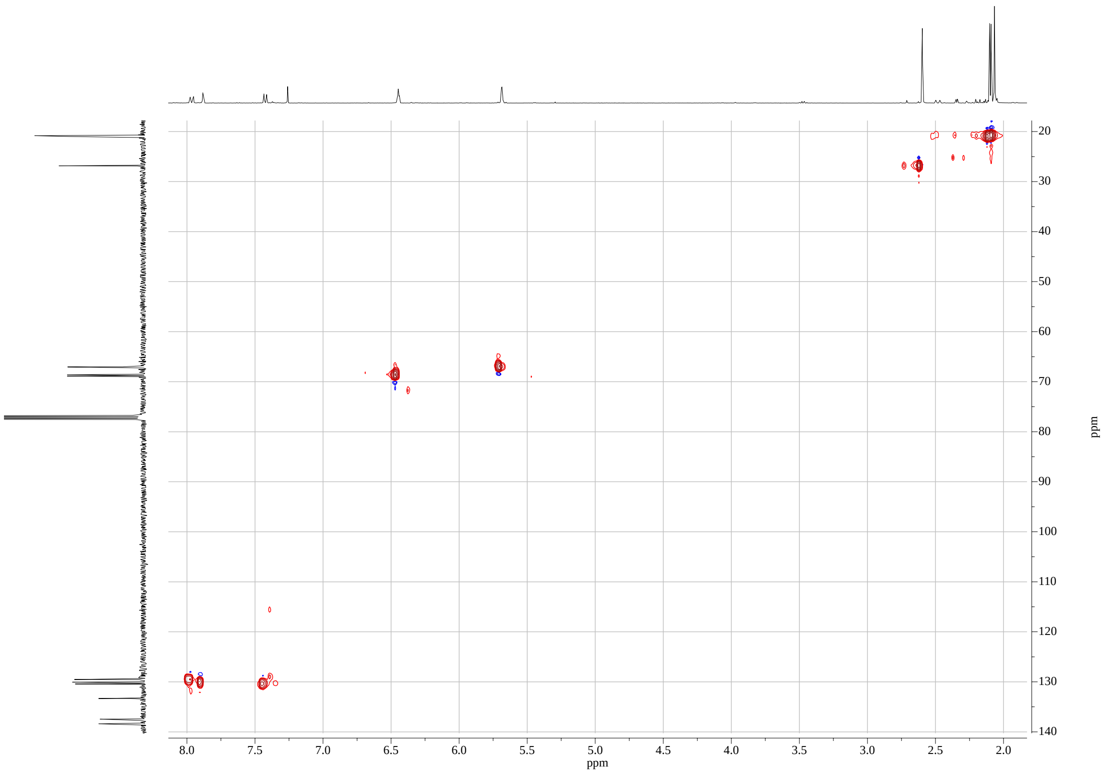

3k^anti - ^{1}H-^{13}C HMBC ($CDCl_3$)

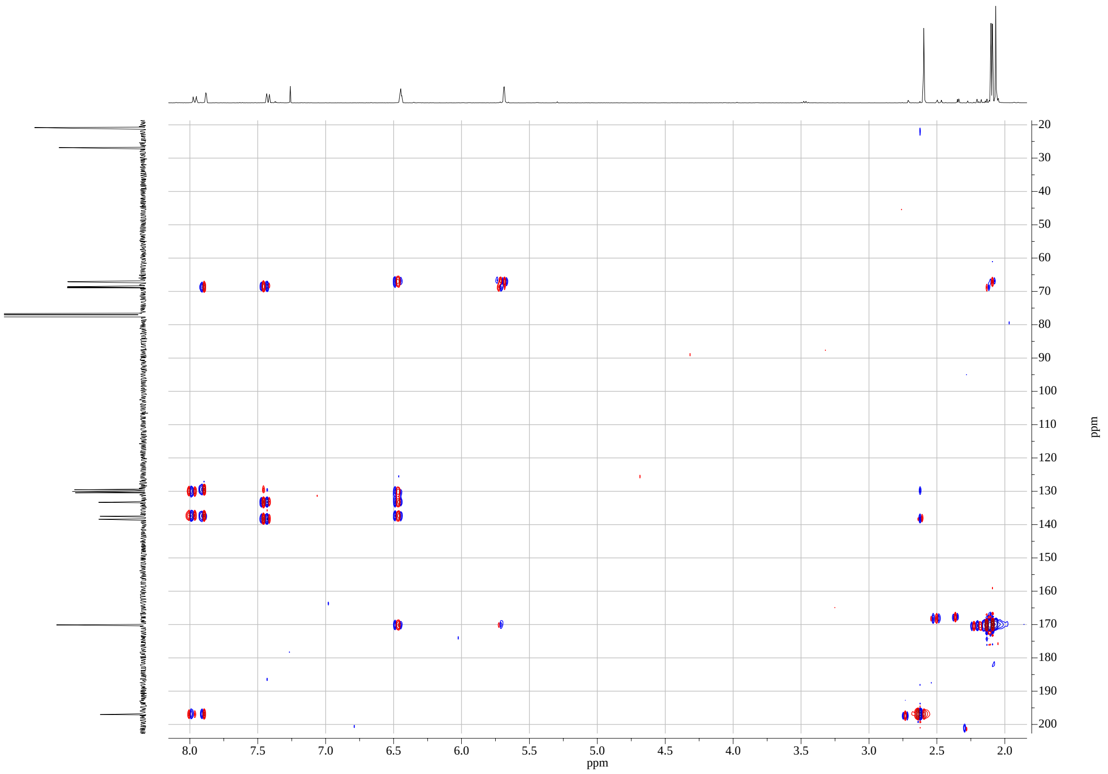

(3α,4α)-3-acetyl-3-hydroxy-1,2,3,4-tetrahydronaphthalene-1,2,4-triyl triacetate (4k)

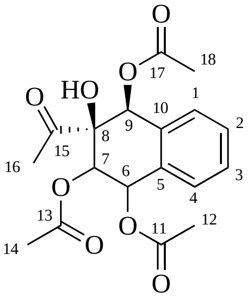

4k - ^{1}H NMR (400 MHz, CDCl$_3$)

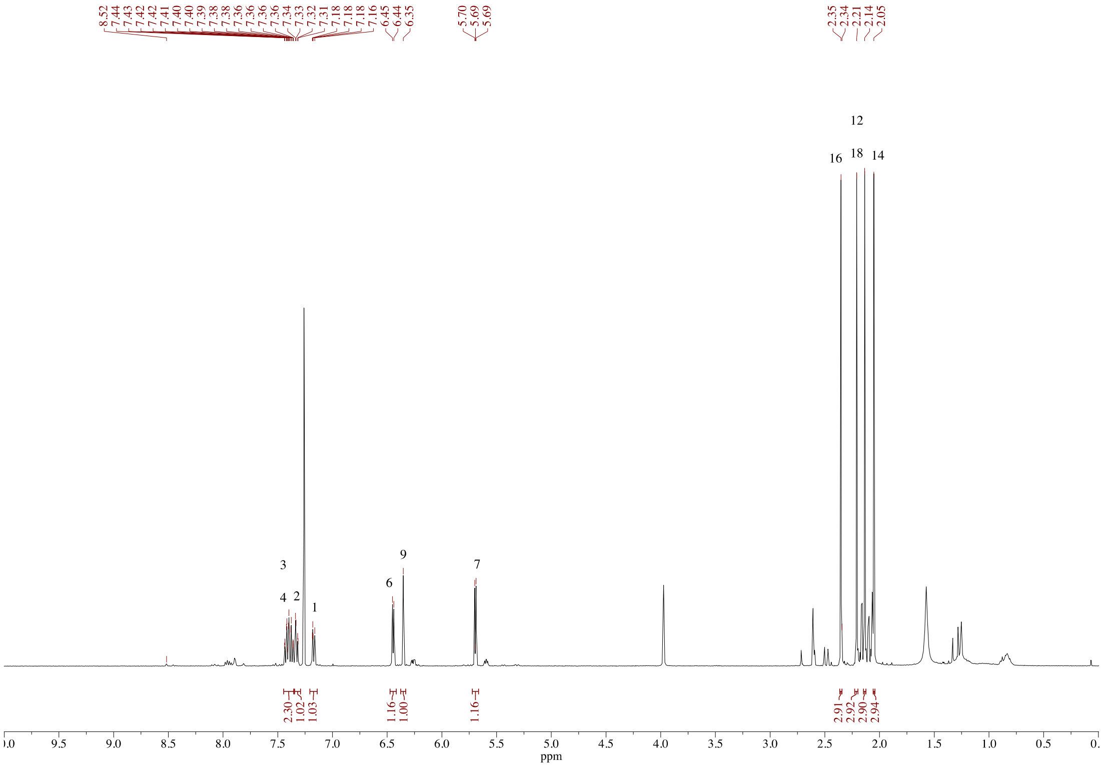

4k - ^{13}C NMR (100 MHz, $CDCl_3$)

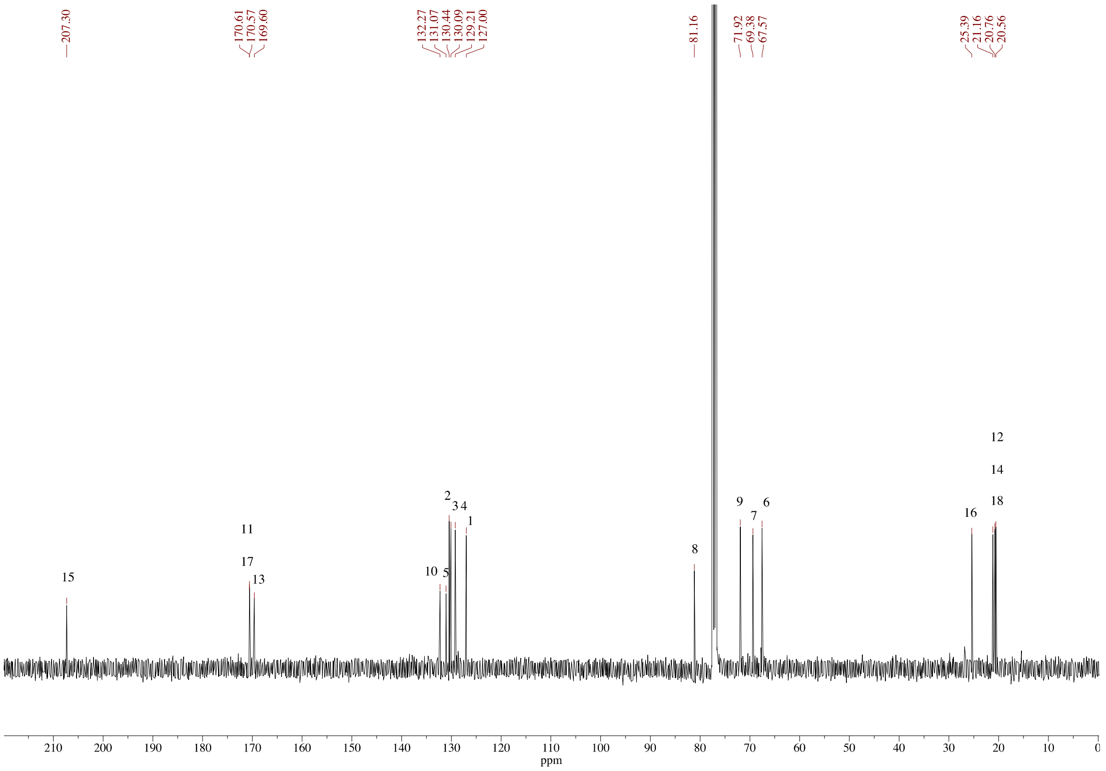

4k - DEPT ($CDCl_3$)

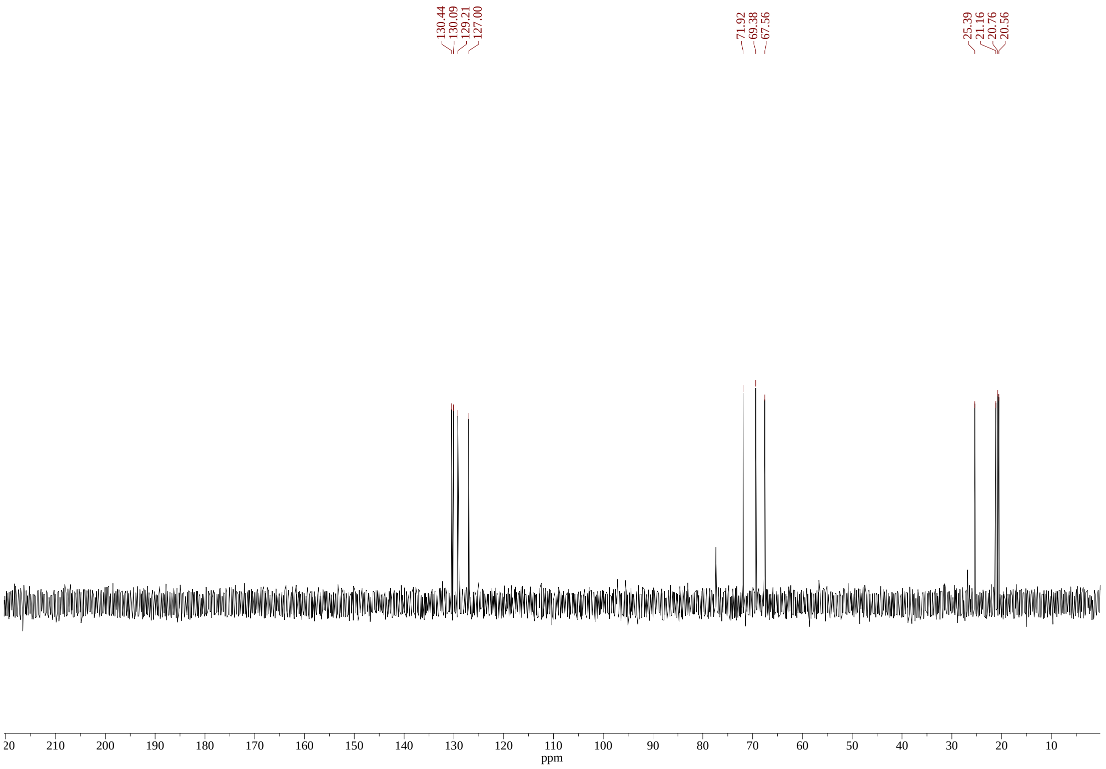

4k - DEPTQ ($CDCl_3$)

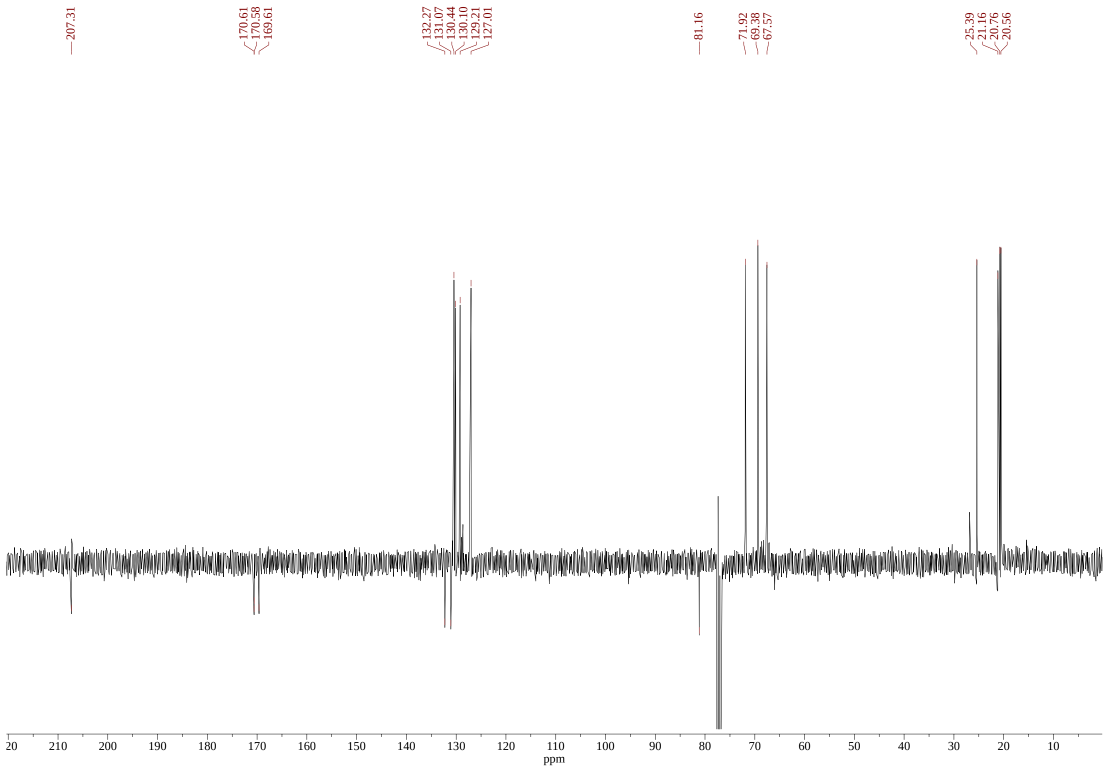

4k - ^1H-^1H COSY ($CDCl_3$)

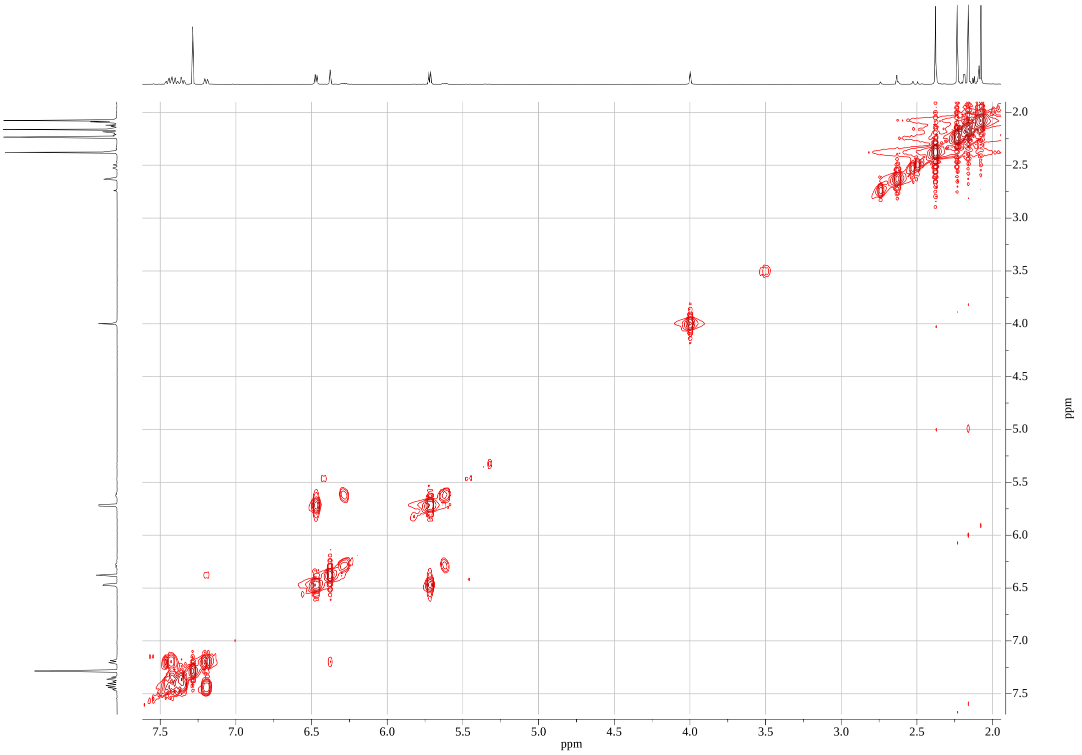

4k - ^{1}H-^{13}C HSQCED ($CDCl_3$)

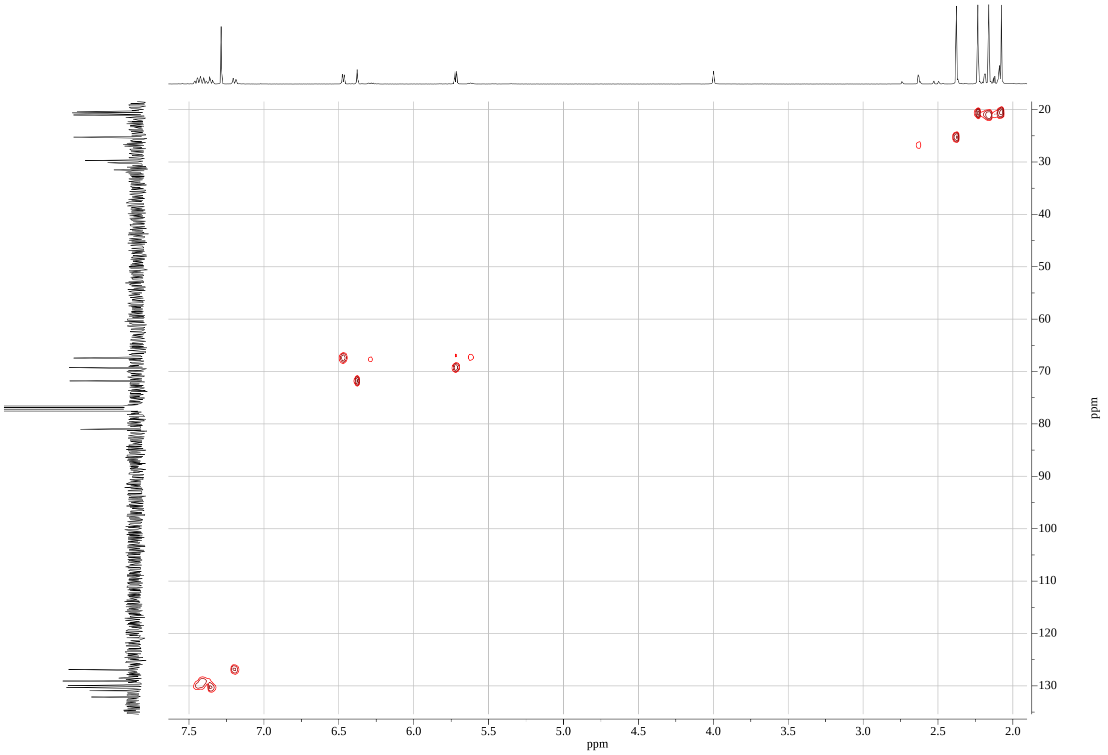

4k - ^{1}H-^{13}C HMBC ($CDCl_3$)

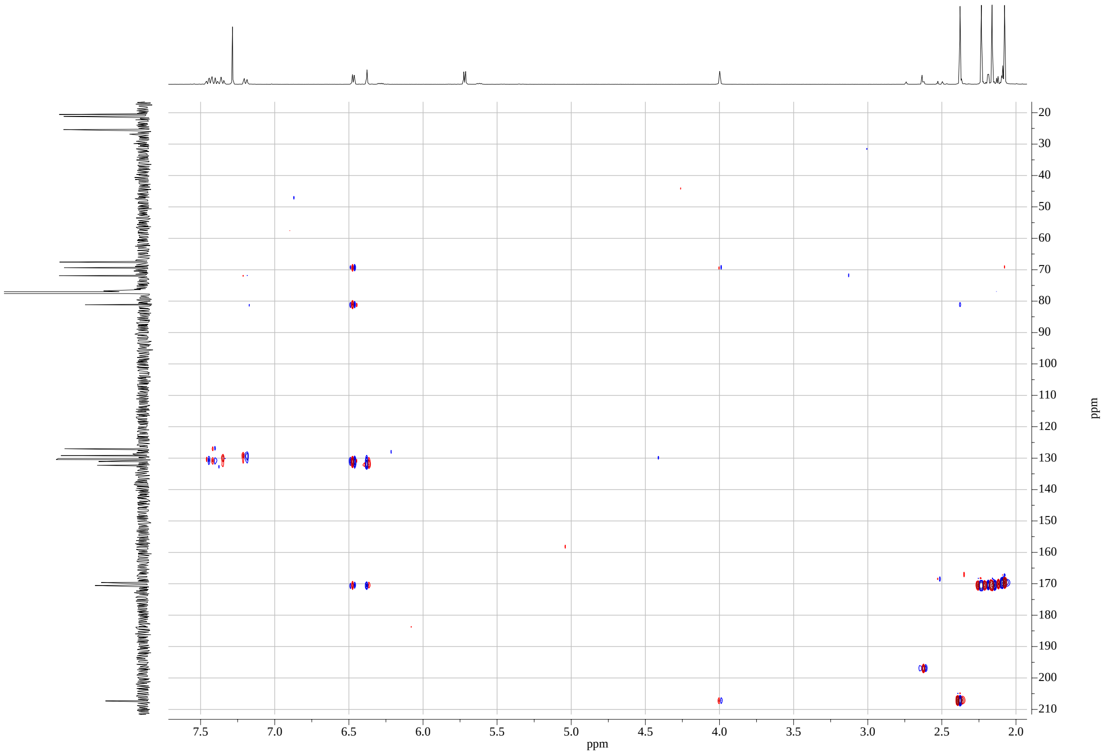

(1α,2α,3α,4α)-5-bromo-1,2,3,4-tetrahydronaphthalene-1,2,3,4-tetrayl tetraacetate ($3o^{syn}$)

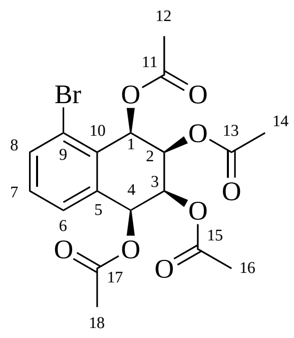

$3o^{syn}$ - ^{1}H NMR (400 MHz, $CDCl_3$)

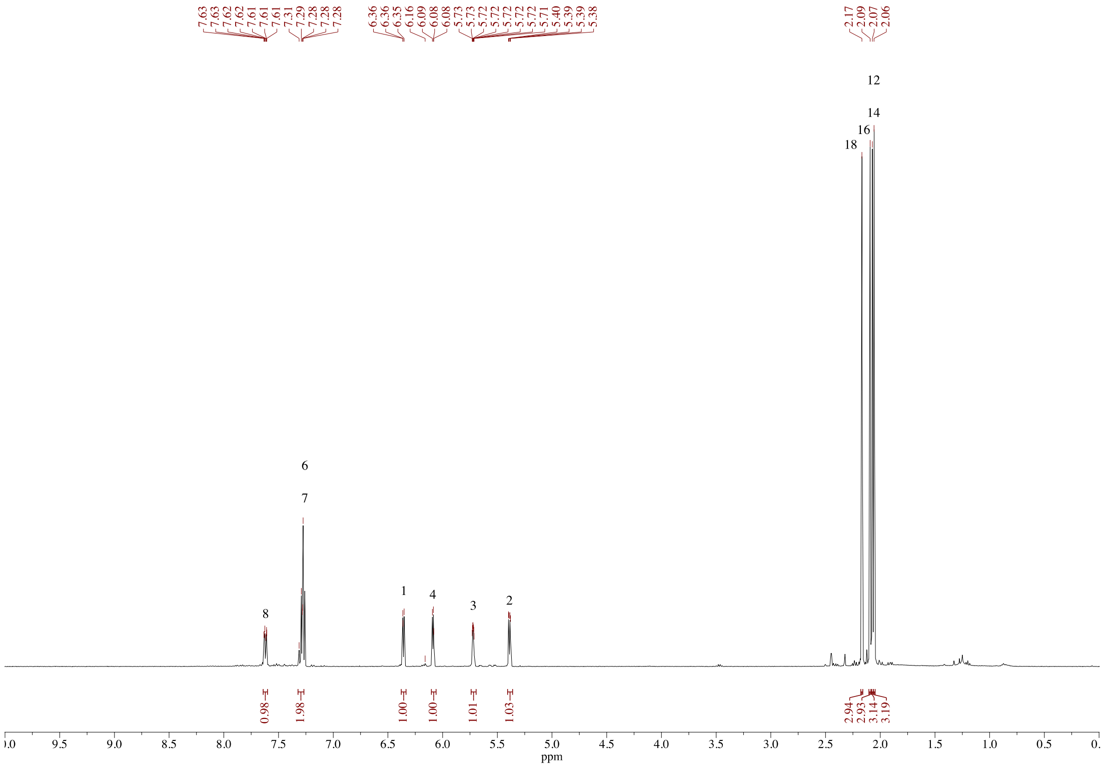

3o[syn] - ^{13}C NMR (100 MHz, $CDCl_3$)

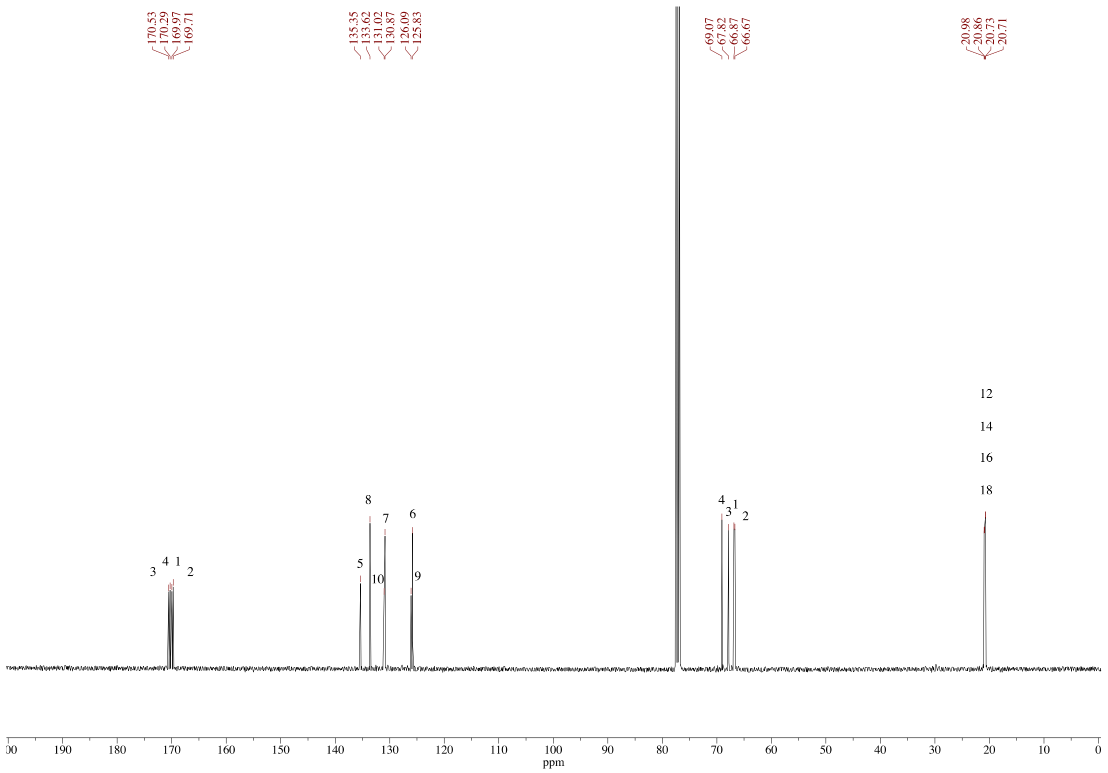

3o[syn] - DEPT ($CDCl_3$)

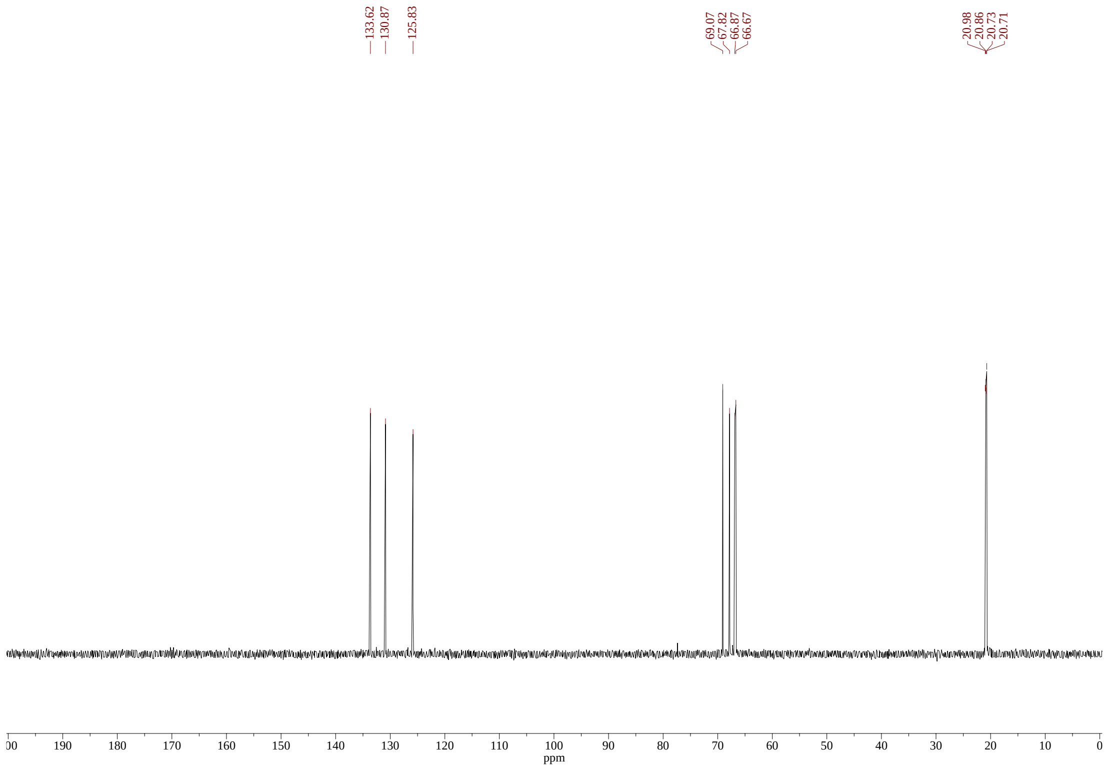

3o^{syn} - DEPTQ ($CDCl_3$)

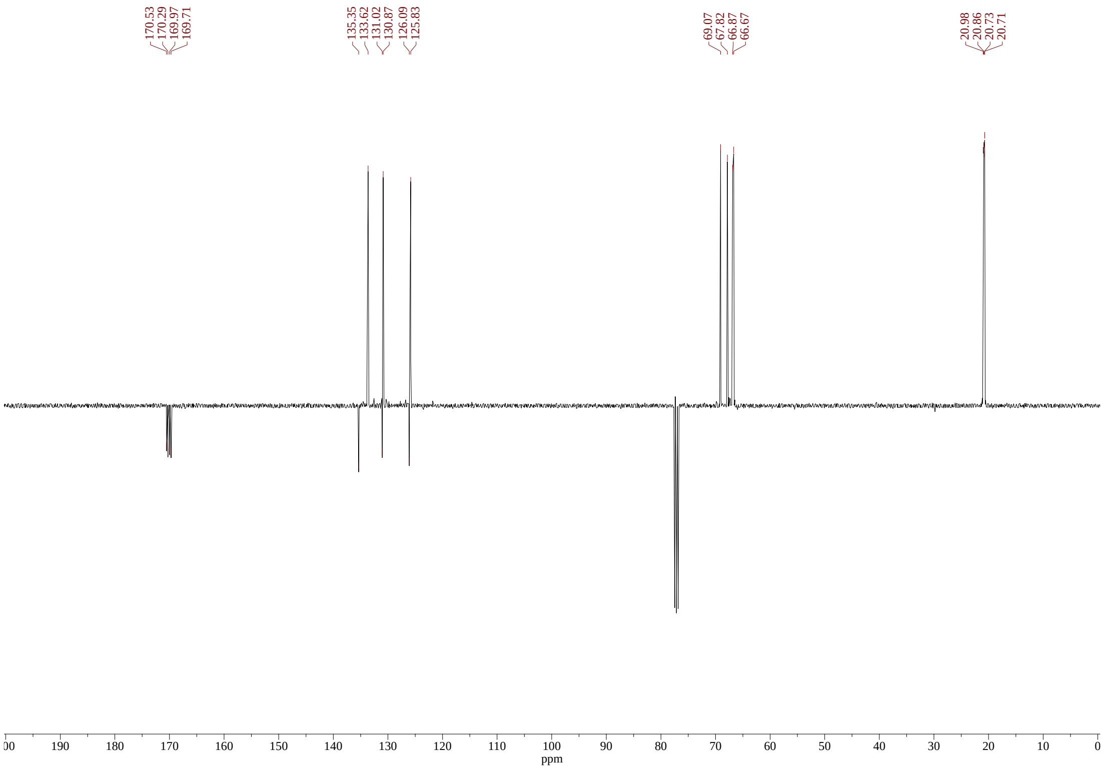

3o^{syn} - ^1H-^1H COSY ($CDCl_3$)

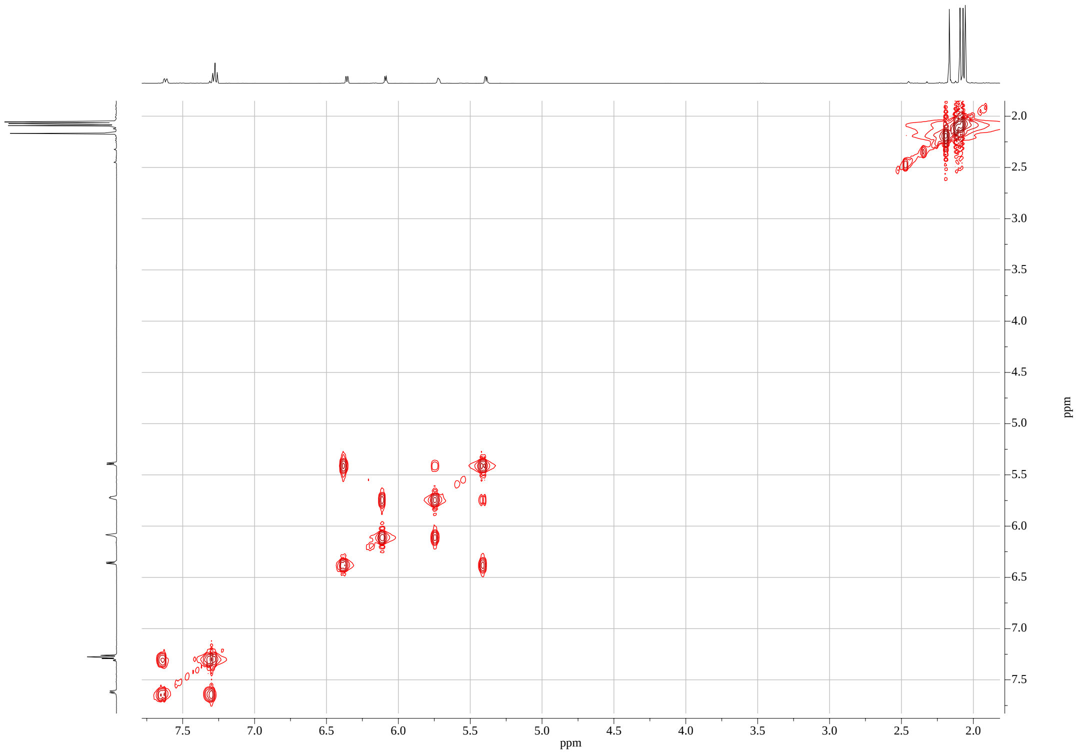

3o^{syn} - ^{1}H-^{13}C HSQCED ($CDCl_3$)

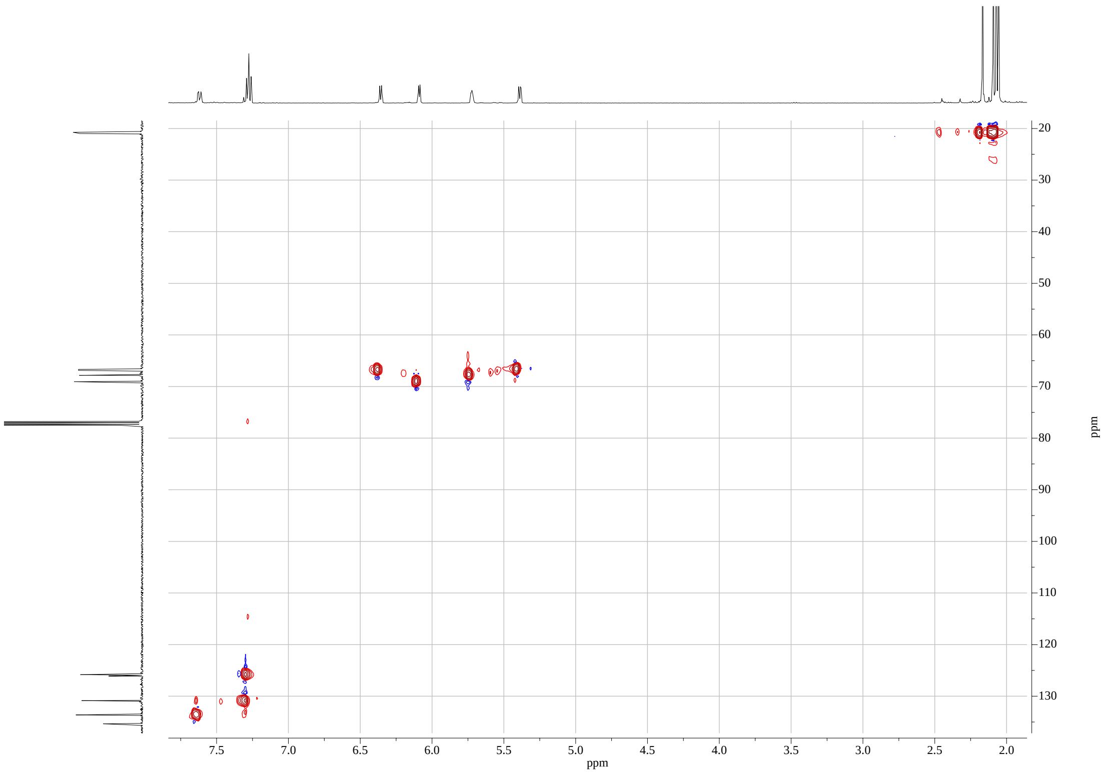

3o^{syn} - ^{1}H-^{13}C HMBC ($CDCl_3$)

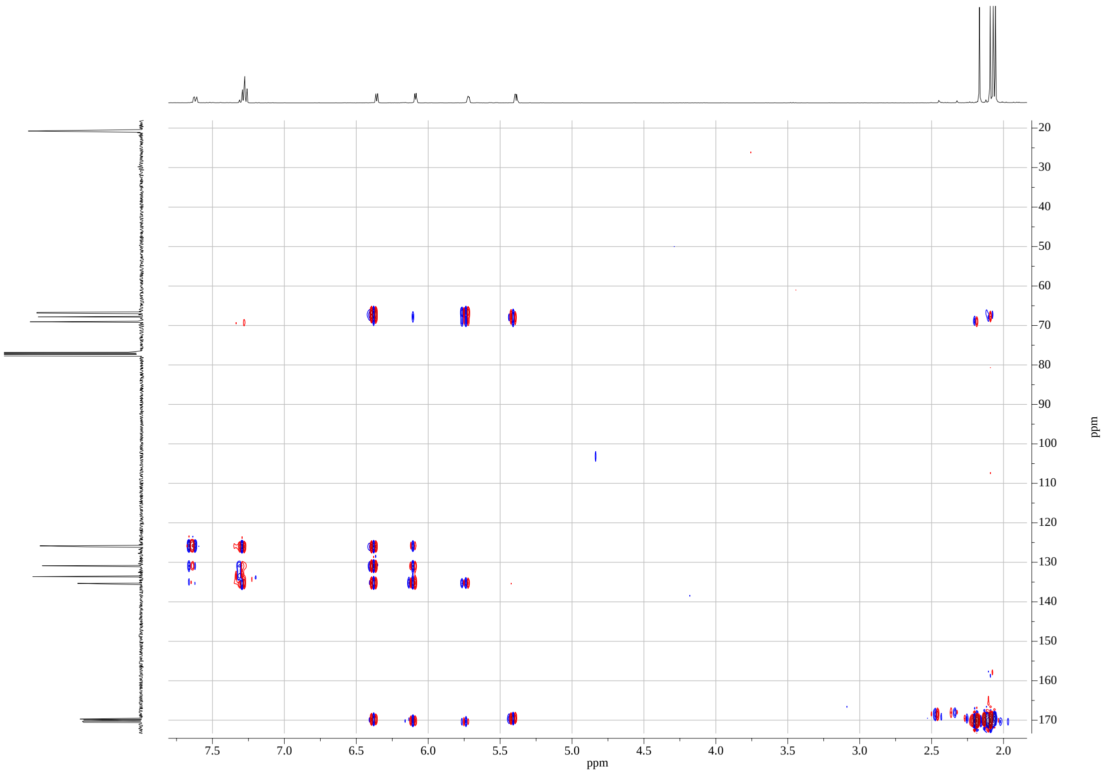

(1α,2α,3β,4β)-5-bromo-1,2,3,4-tetrahydronaphthalene-1,2,3,4-tetrayl tetraacetate (3o^{anti})

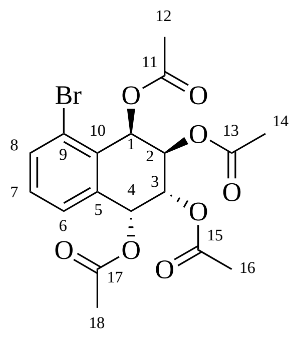

3o^{anti} - ^{1}H N MR (400 MHz, $CDCl_3$)

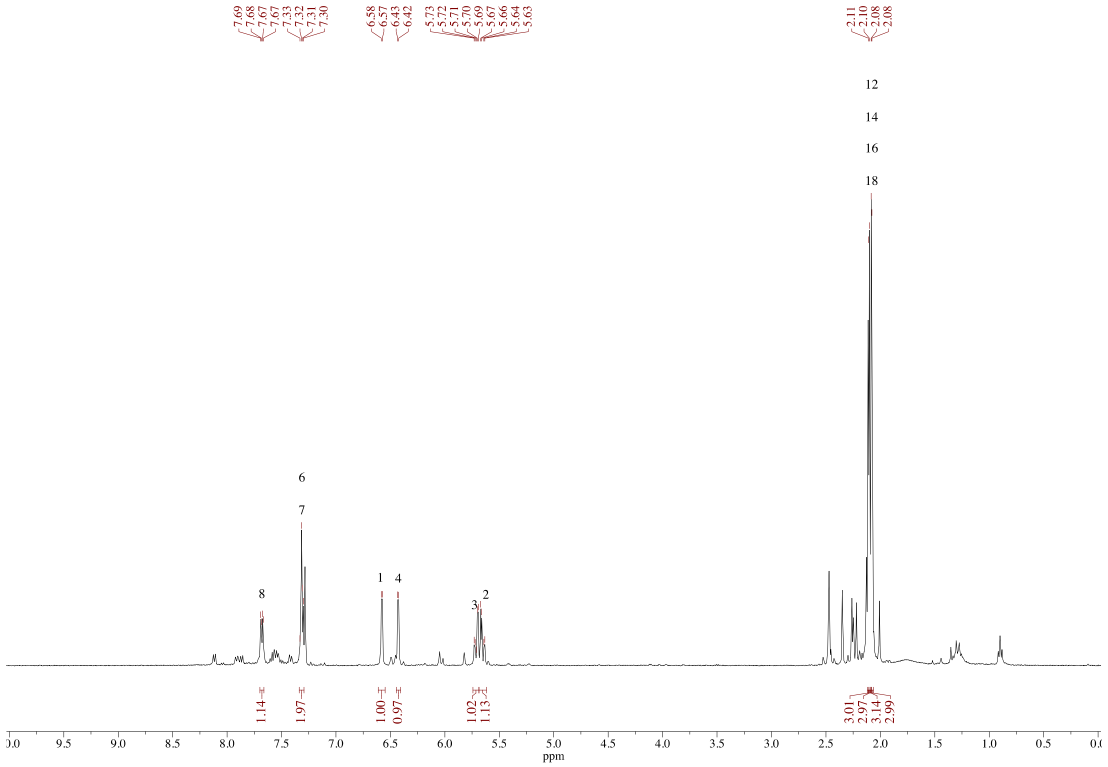

3o^anti - ^{13}C NMR (100 MHz, $CDCl_3$)

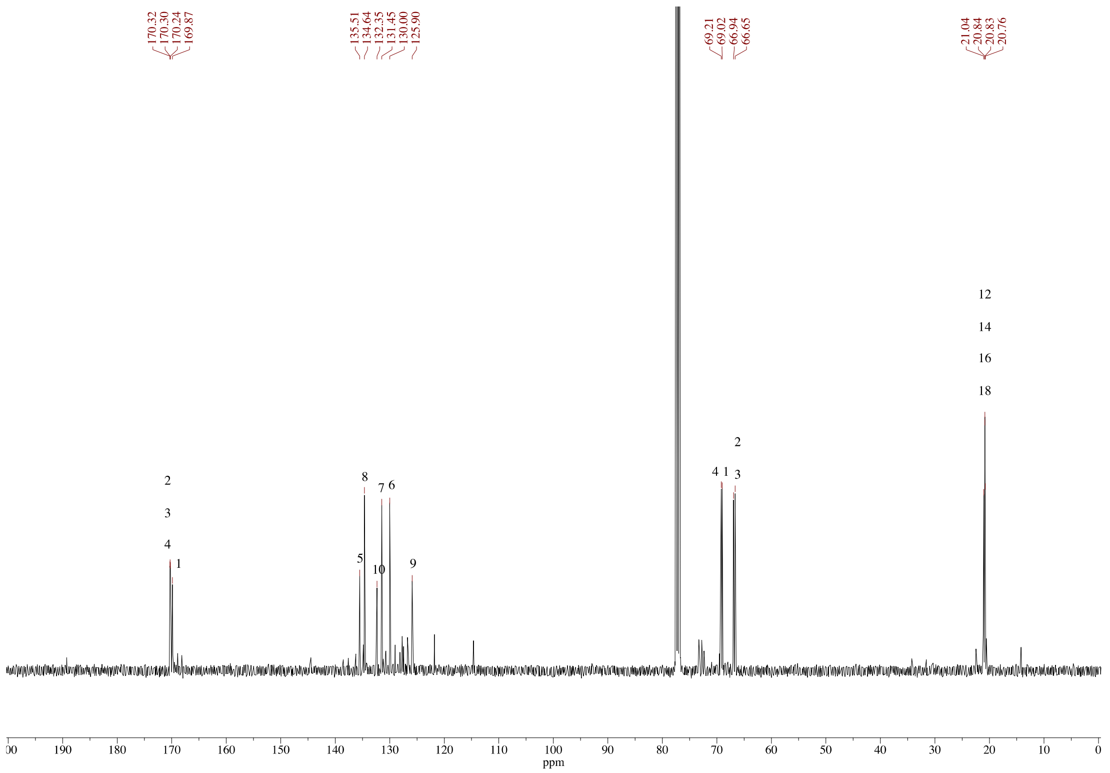

3o^anti - DEPT ($CDCl_3$)

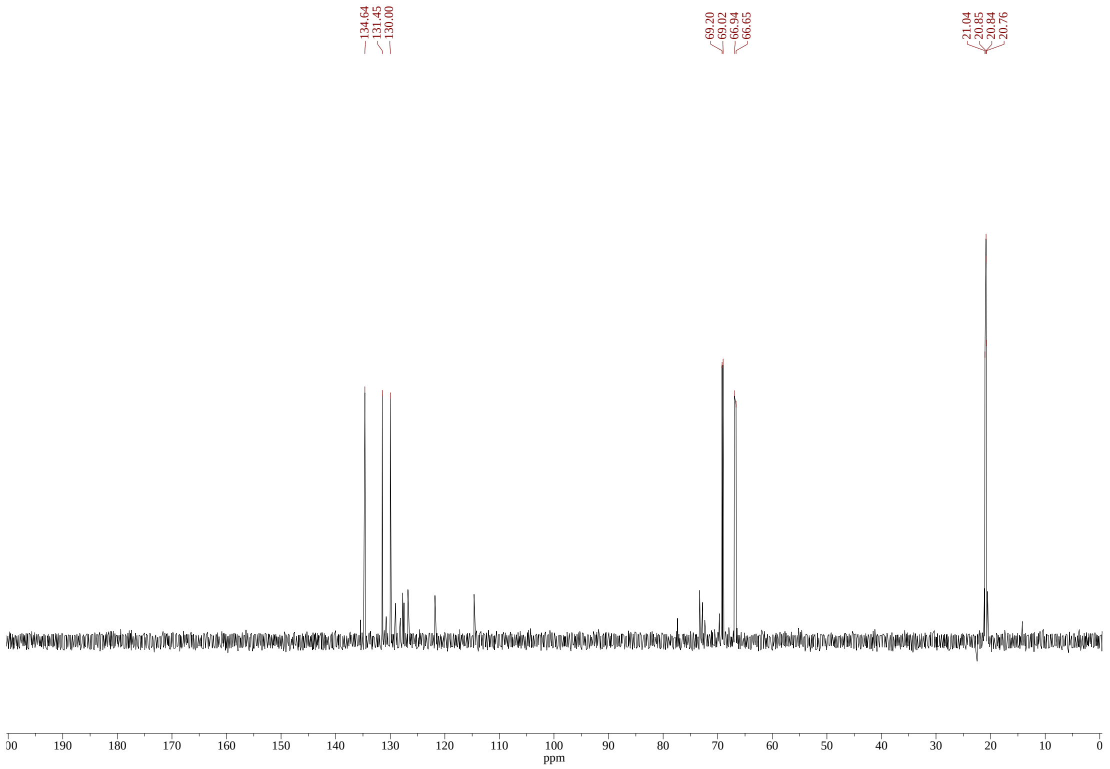

3o^{anti} - DEPTQ (CDCl3)

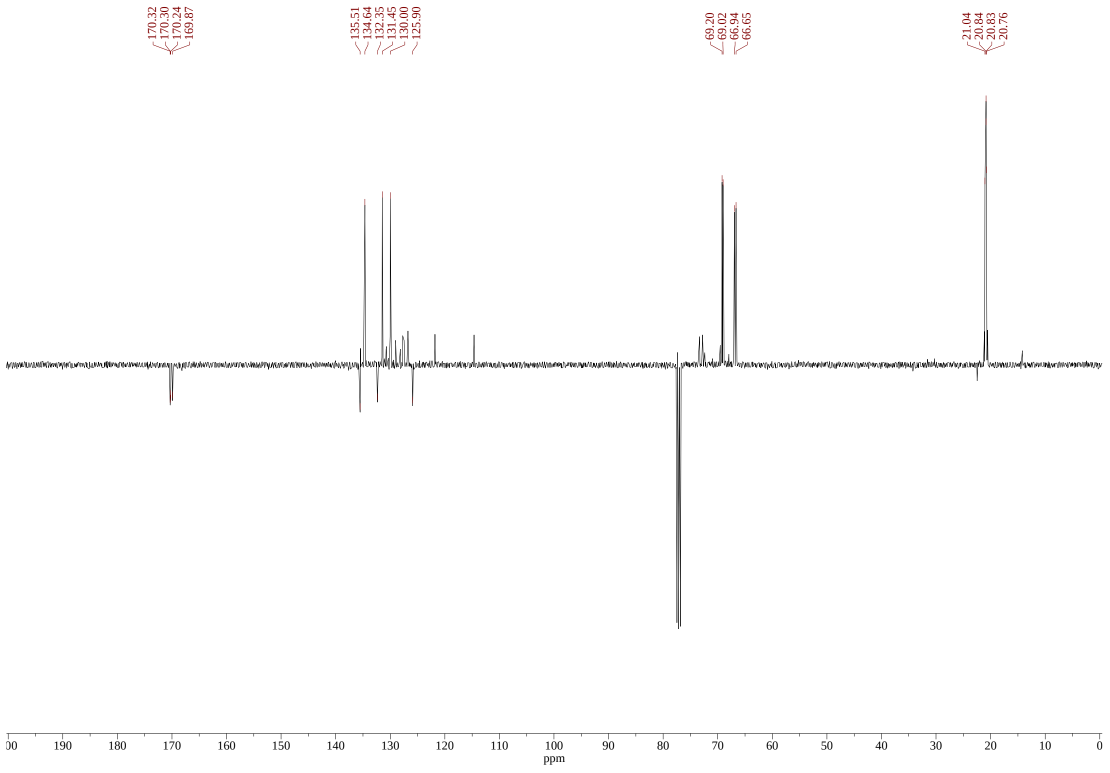

3o^{anti} - ^{1}H-^{1}H COSY (CDCl3)

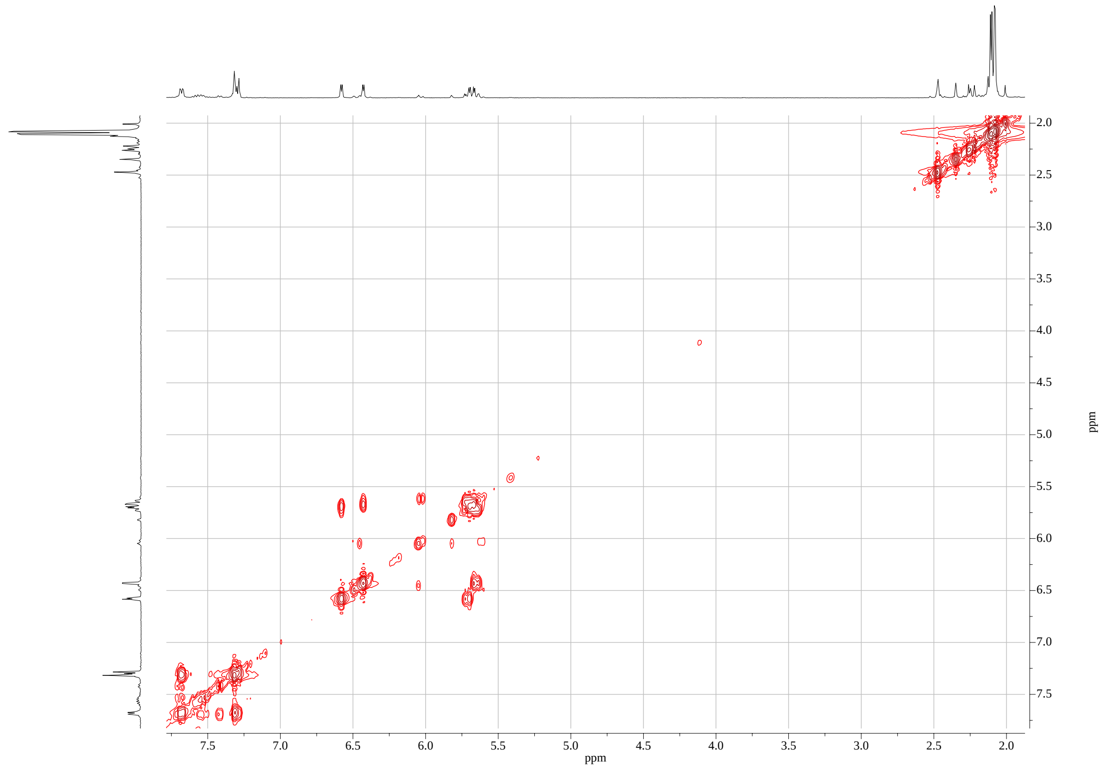

3o^{anti} - ^{1}H-^{13}C HSQCED ($CDCl_3$)

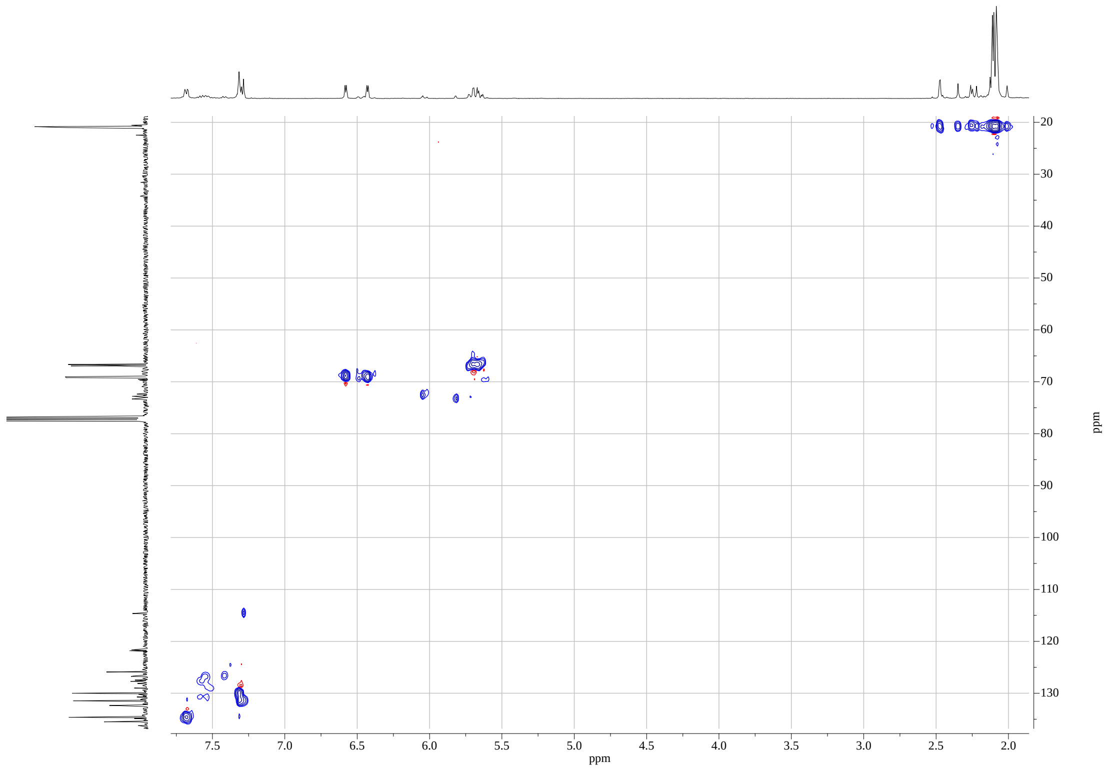

3o^{anti} - ^{1}H-^{13}C HMBC ($CDCl_3$)

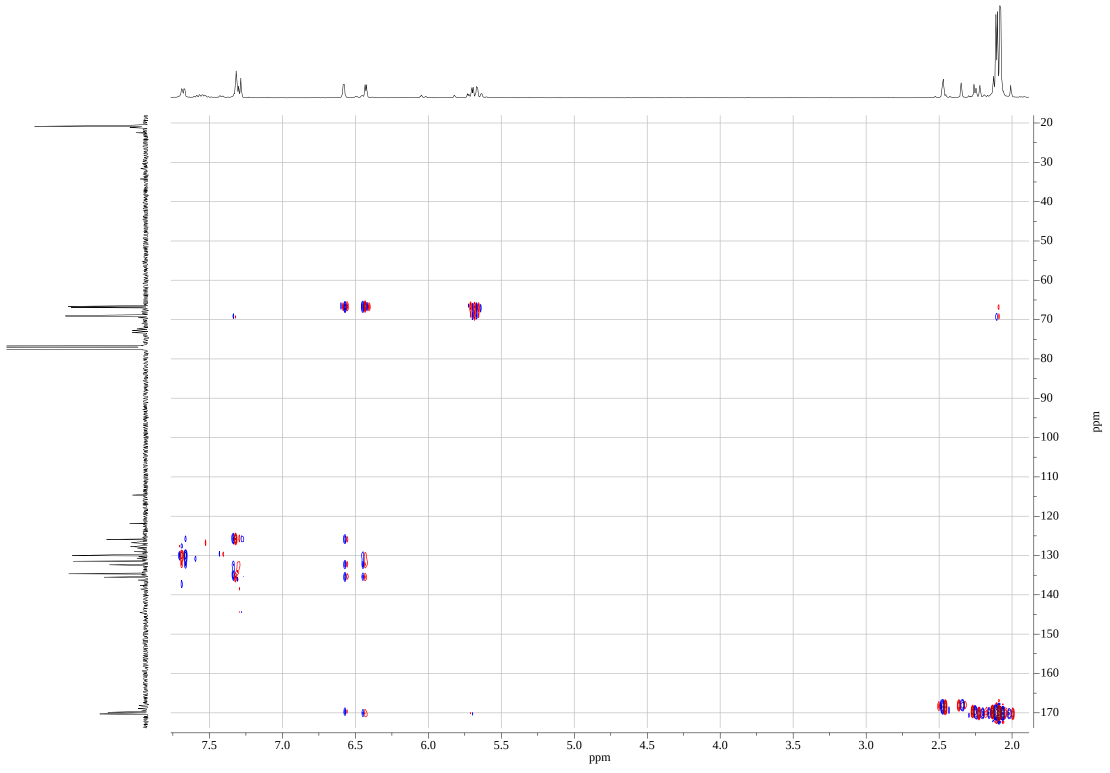

(1α,2α,3α,4α)-4-bromo-4-hydroxy-1,2,3,4-tetrahydronaphthalene-1,2,3-triyl triacetate (4o)

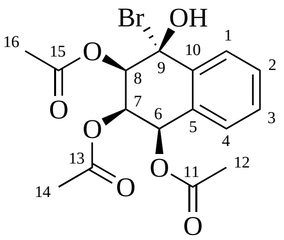

4o - ^{1}H NMR (400 MHz, $CDCl_3$)

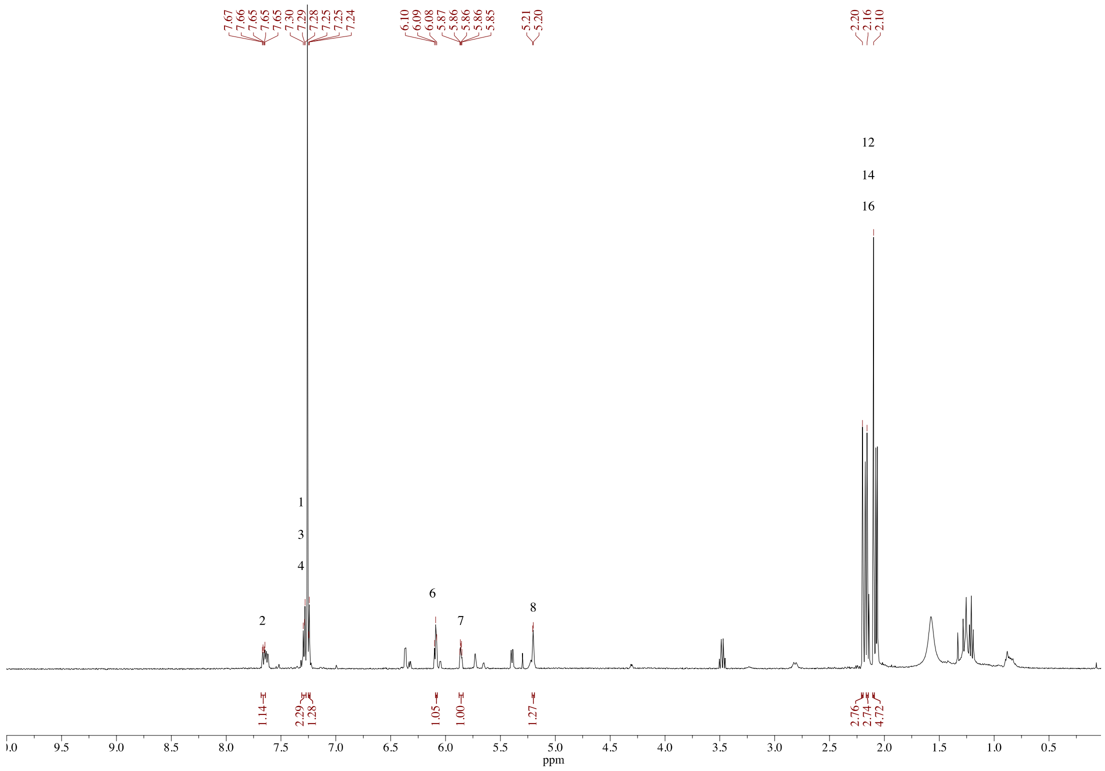

4o - ^{13}C NMR (100 MHz, CDCl$_3$)

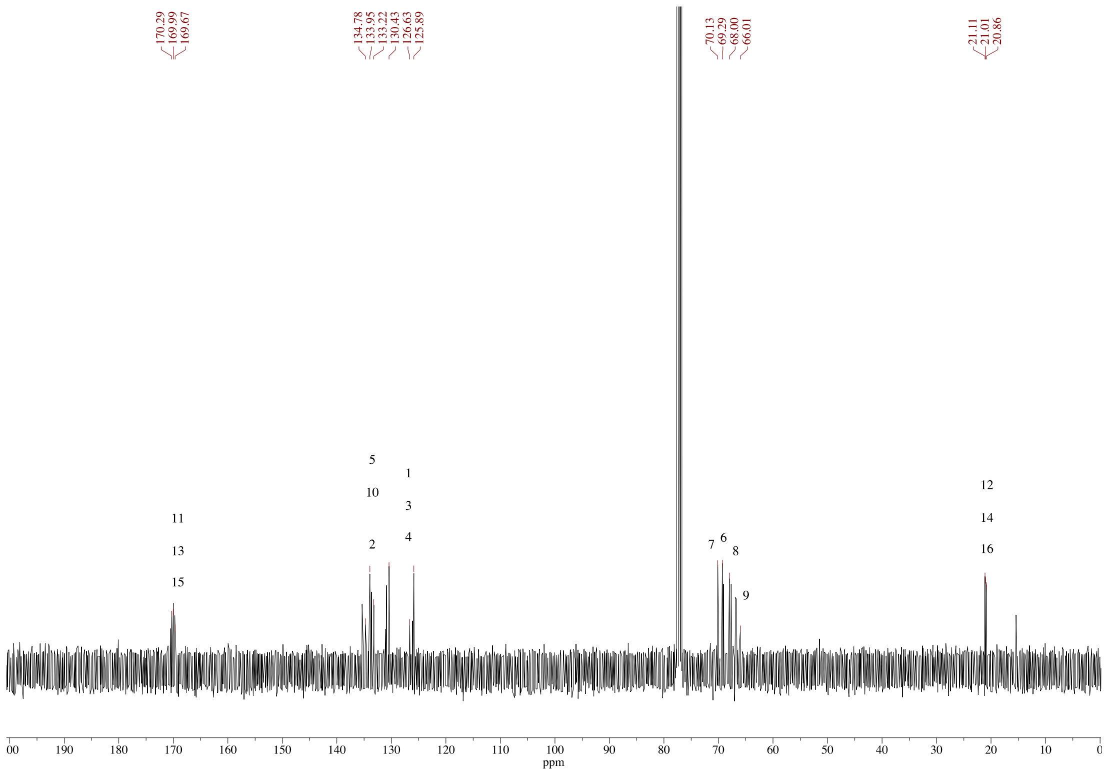

4o - DEPT (CDCl$_3$)

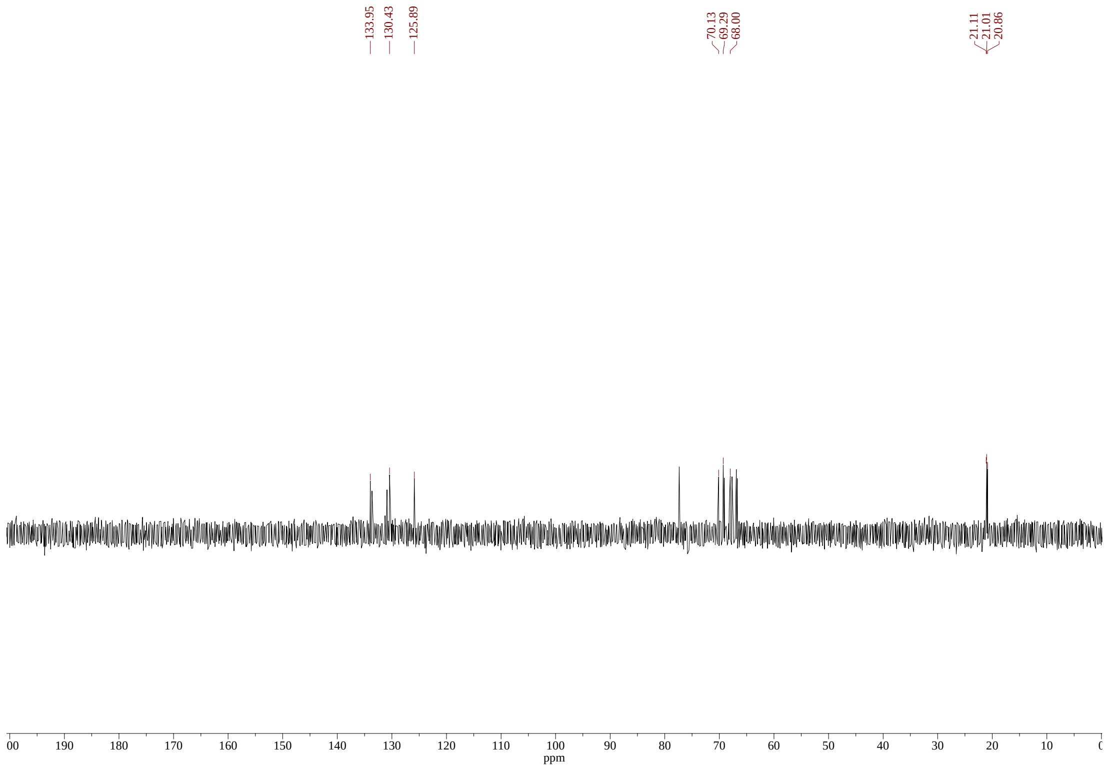

4o - DEPTQ ($CDCl_3$)

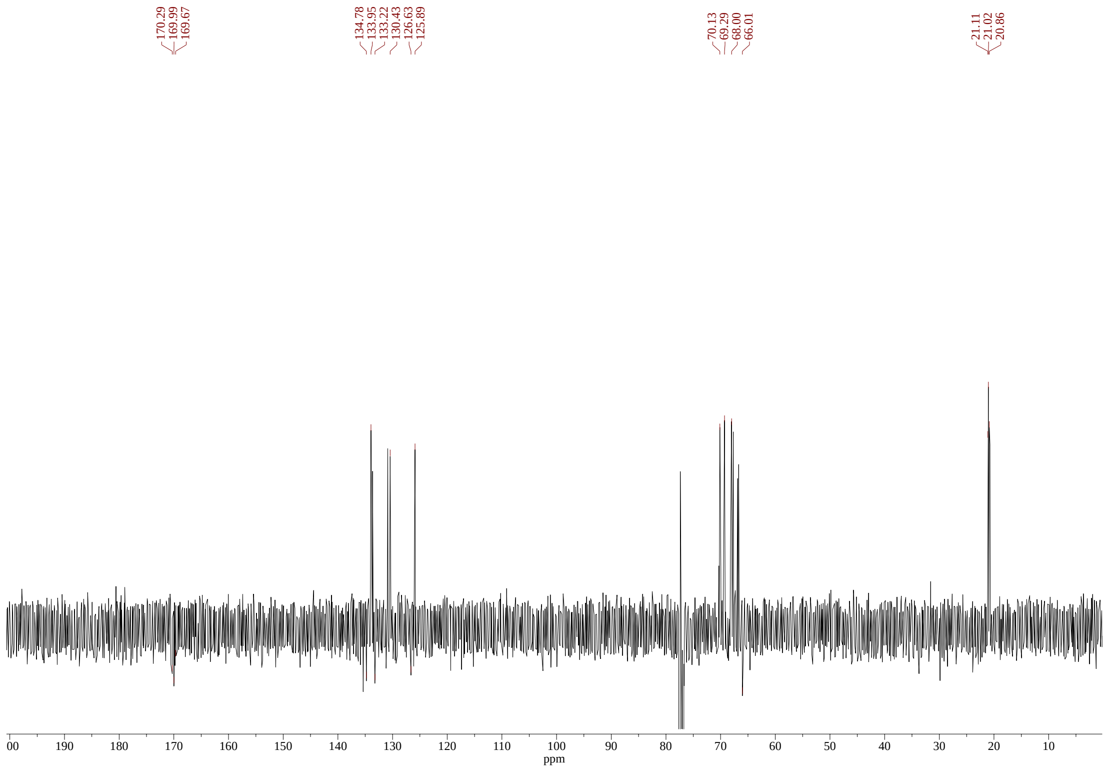

4o - ^1H-^1H COSY ($CDCl_3$)

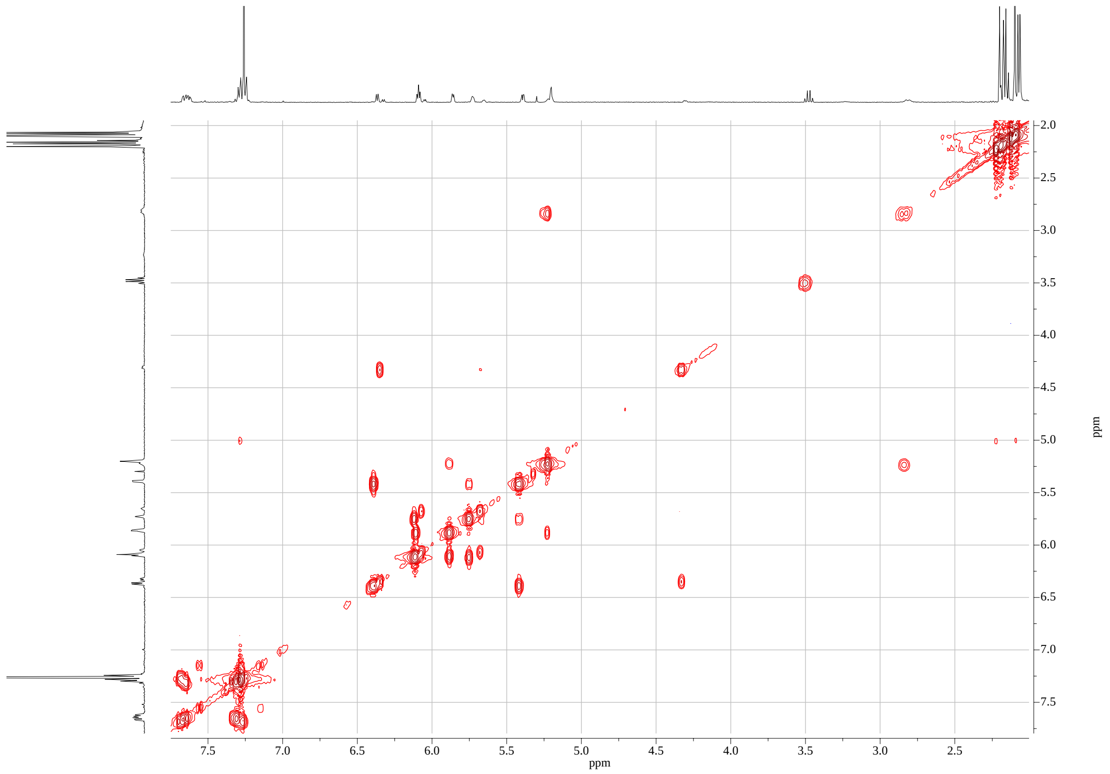

4o - ^{1}H-^{13}C HSQCED ($CDCl_3$)

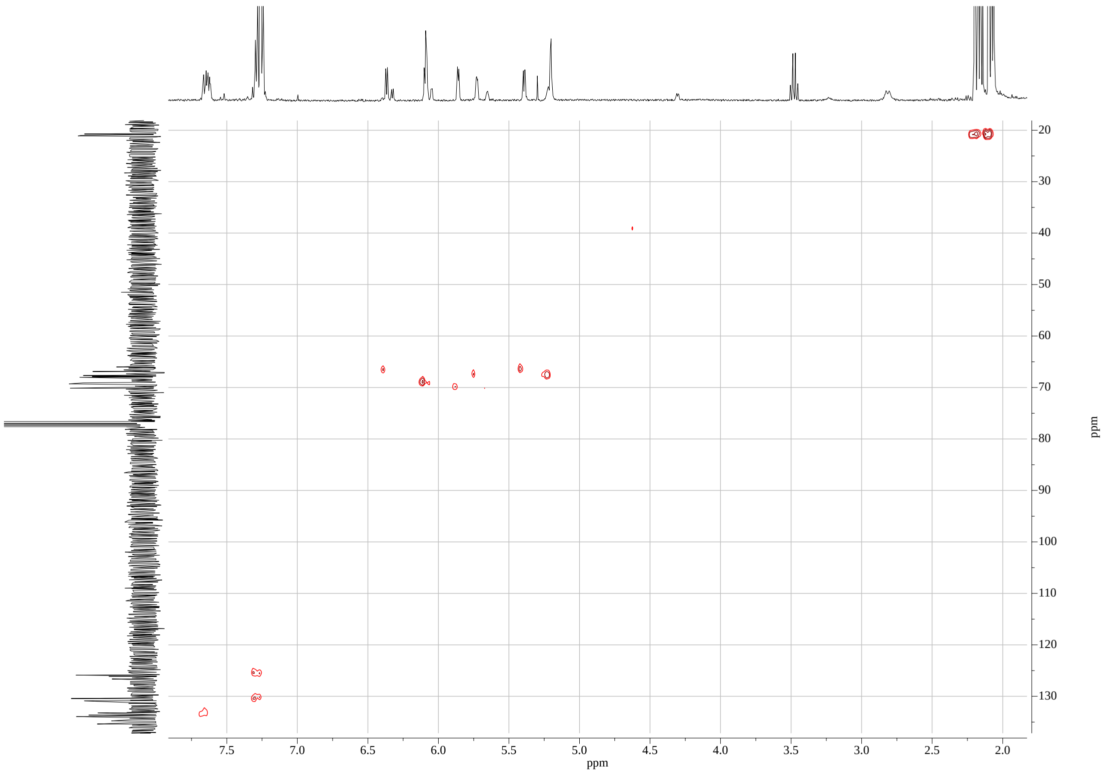

(1α,2α,3α,4α)-5-chloro-1,2,3,4-tetrahydronaphthalene-1,2,3,4-tetrayl tetraacetate (3p^{syn})

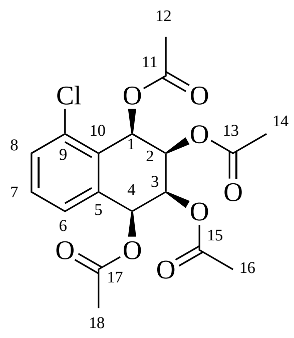

3p^{syn} - ^{1}H NMR (400 MHz, CDCl$_3$)

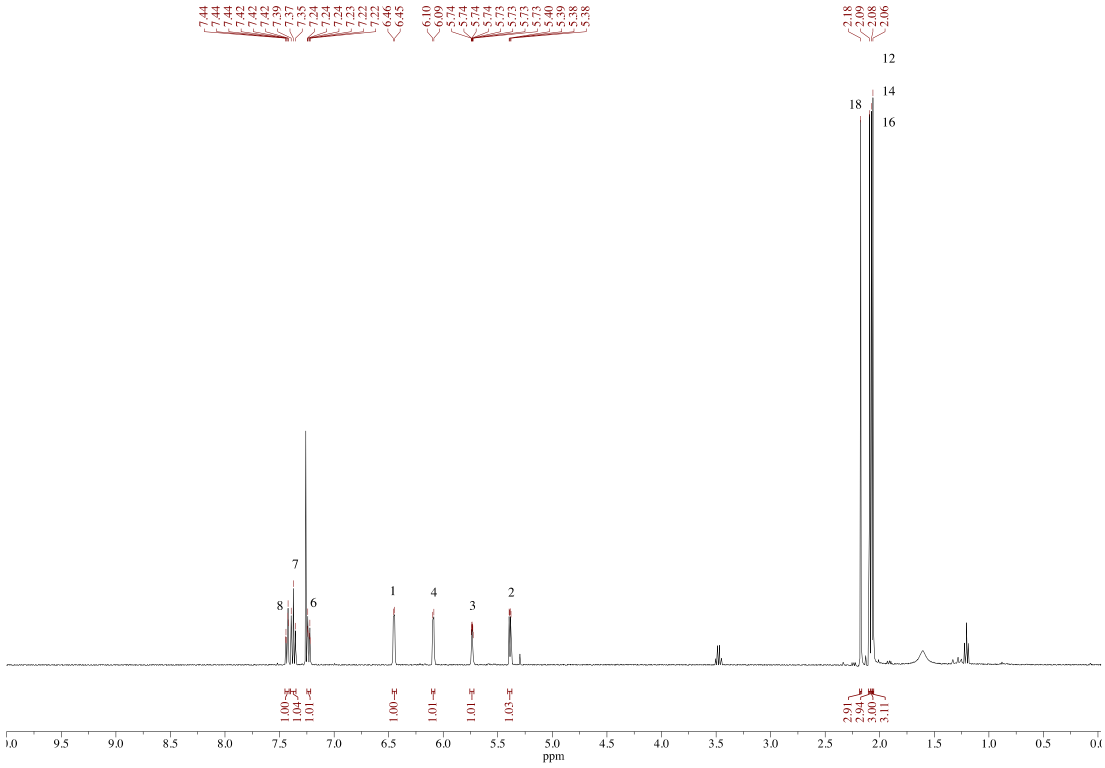

3p^syn^ - ^{13}C NMR (100 MHz, $CDCl_3$)

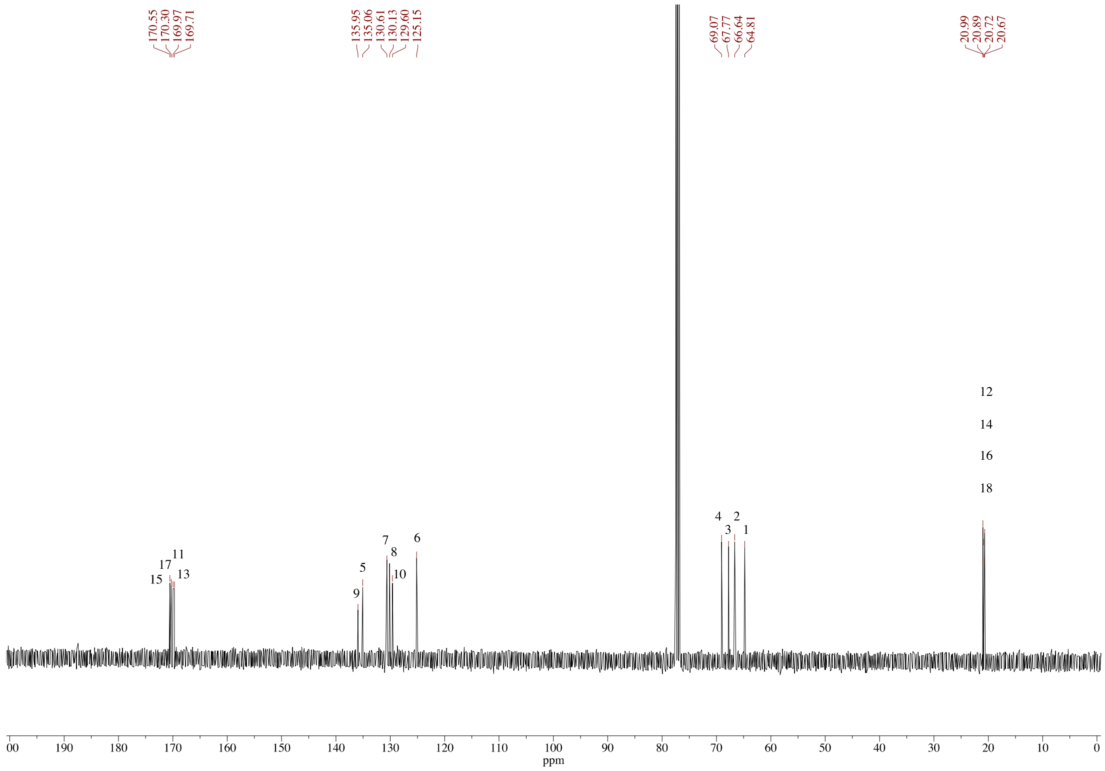

3p^syn^ - DEPT ($CDCl_3$)

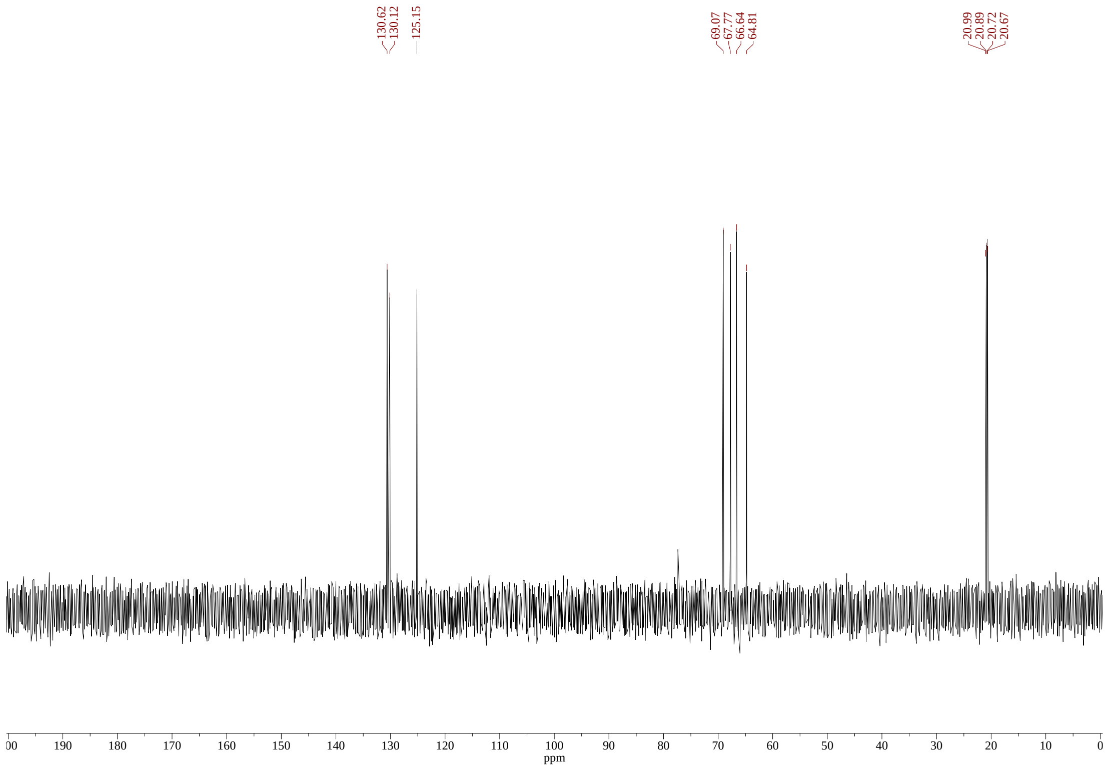

3p^{syn} - DEPTQ ($CDCl_3$)

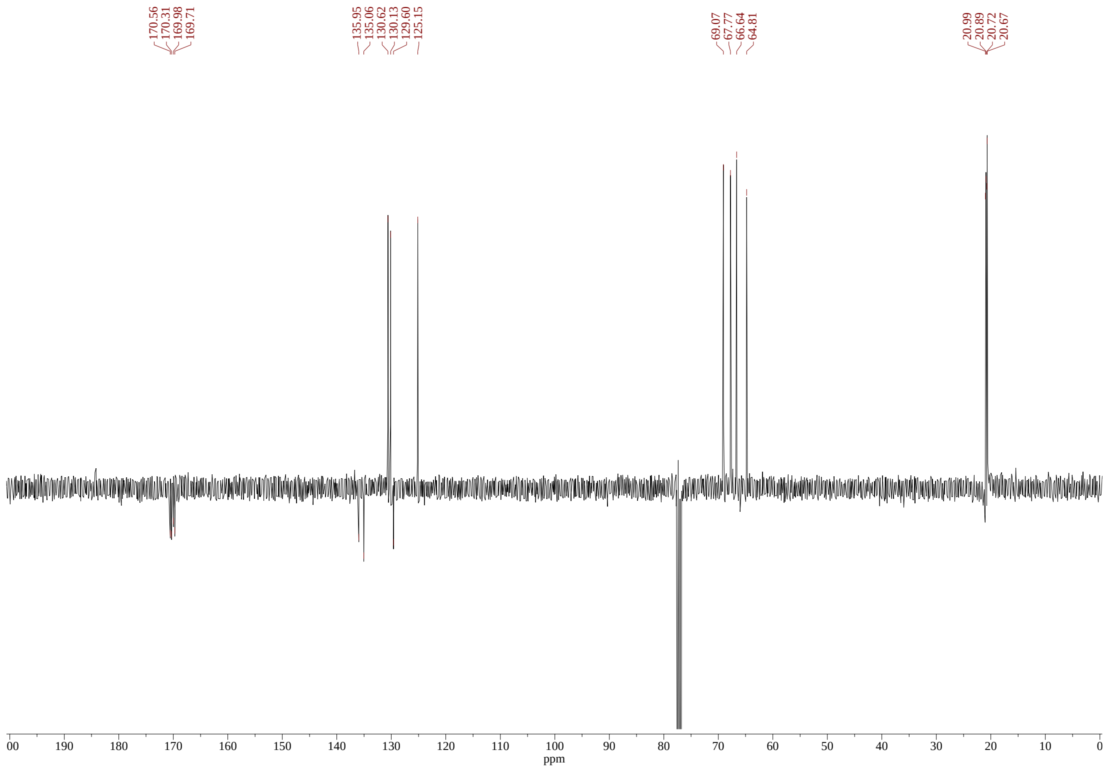

3p^{syn} - ^{1}H-^{1}H COSY ($CDCl_3$)

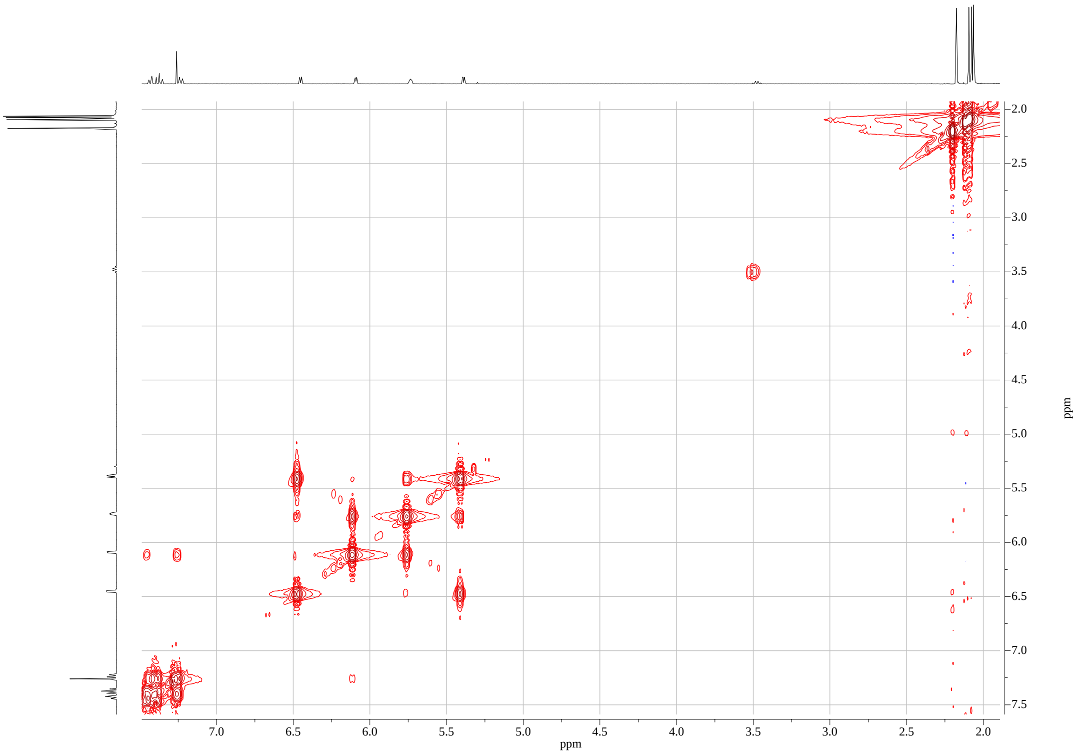

3p[syn] - ^{1}H-^{13}C HSQCED (CDCl$_3$)

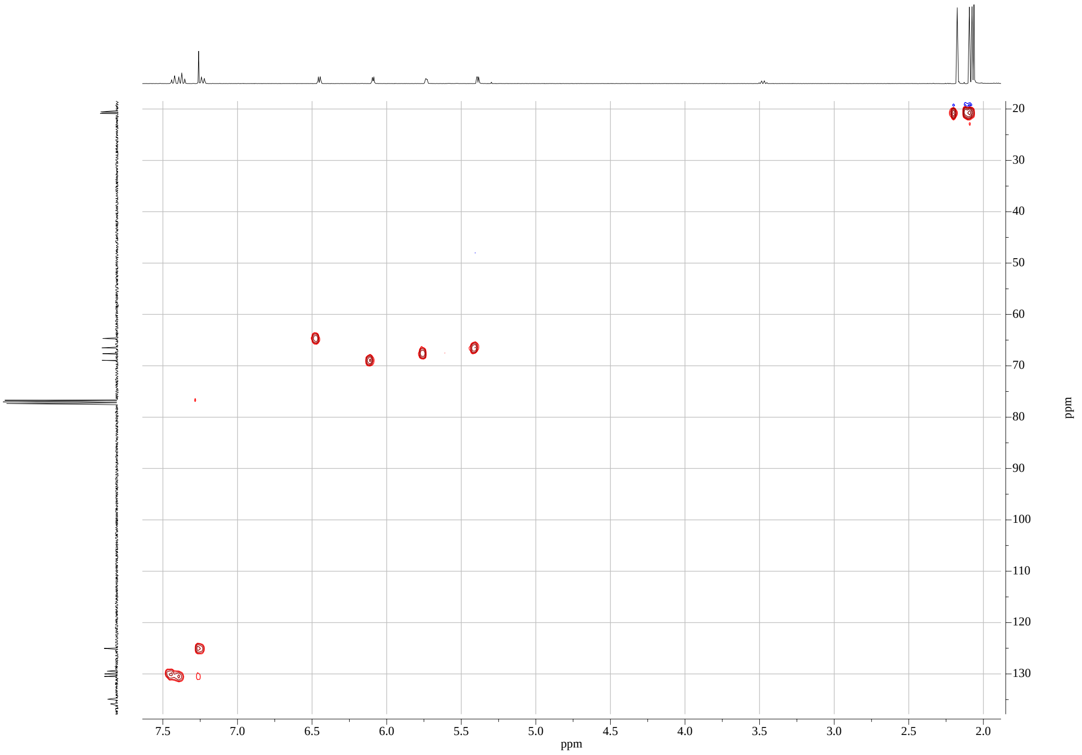

3p[syn] - ^{1}H-^{13}C HMBC (CDCl$_3$)

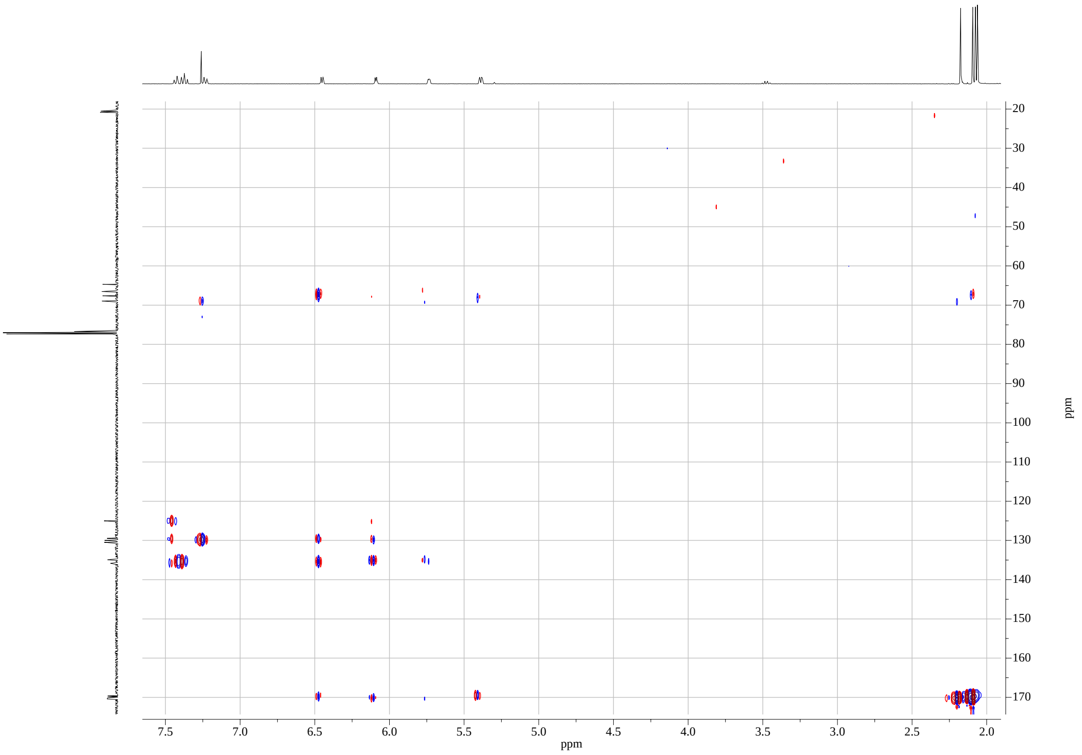

(1α,2α,3β,4β)-5-chloro-1,2,3,4-tetrahydronaphthalene-1,2,3,4-tetrayl tetraacetate (3p^anti^)

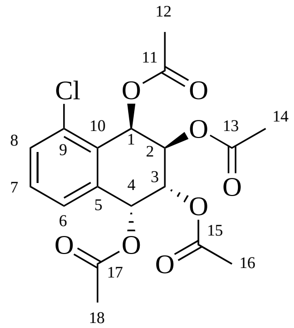

3p^anti^ - ^{1}H NMR (400 MHz, $CDCl_3$)

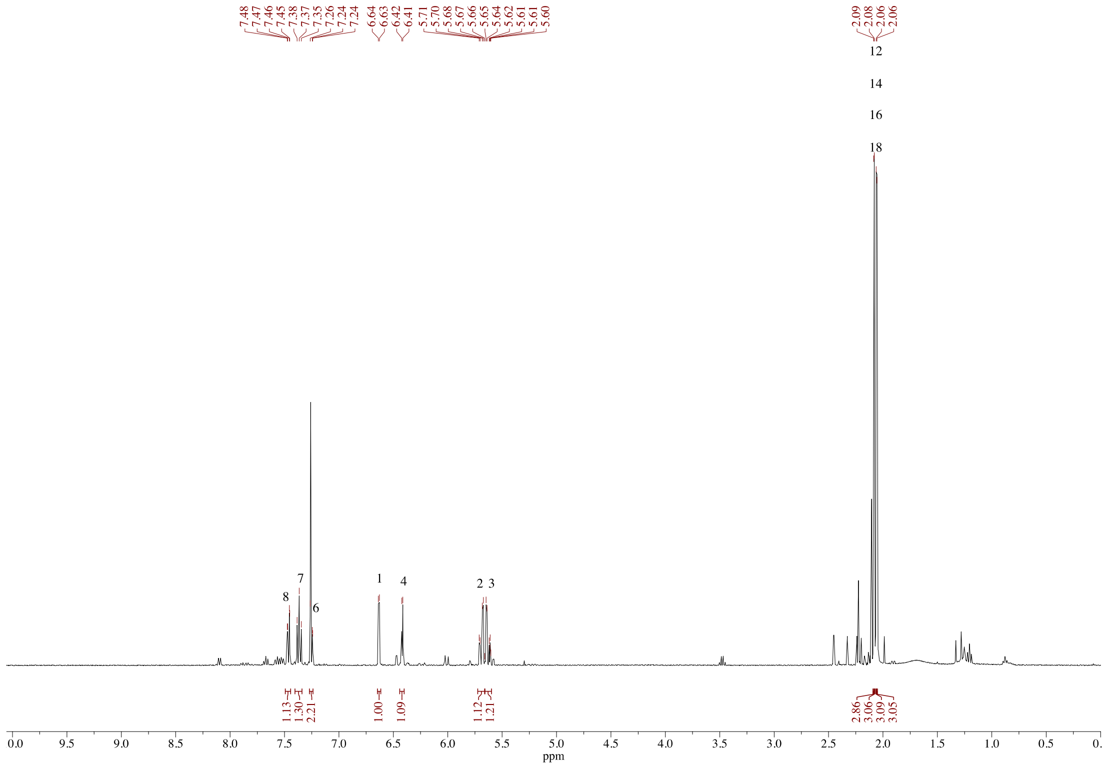

3p[anti] - ^{13}C NMR (100 MHz, $CDCl_3$)

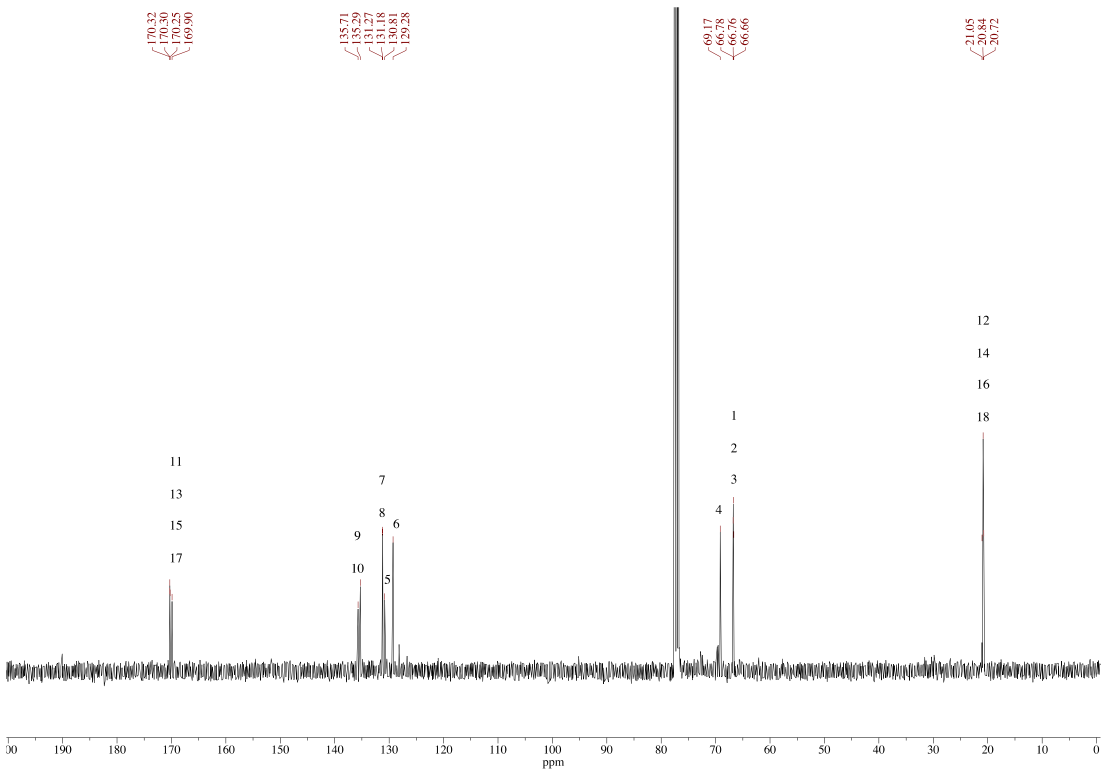

3p[anti] - DEPT ($CDCl_3$)

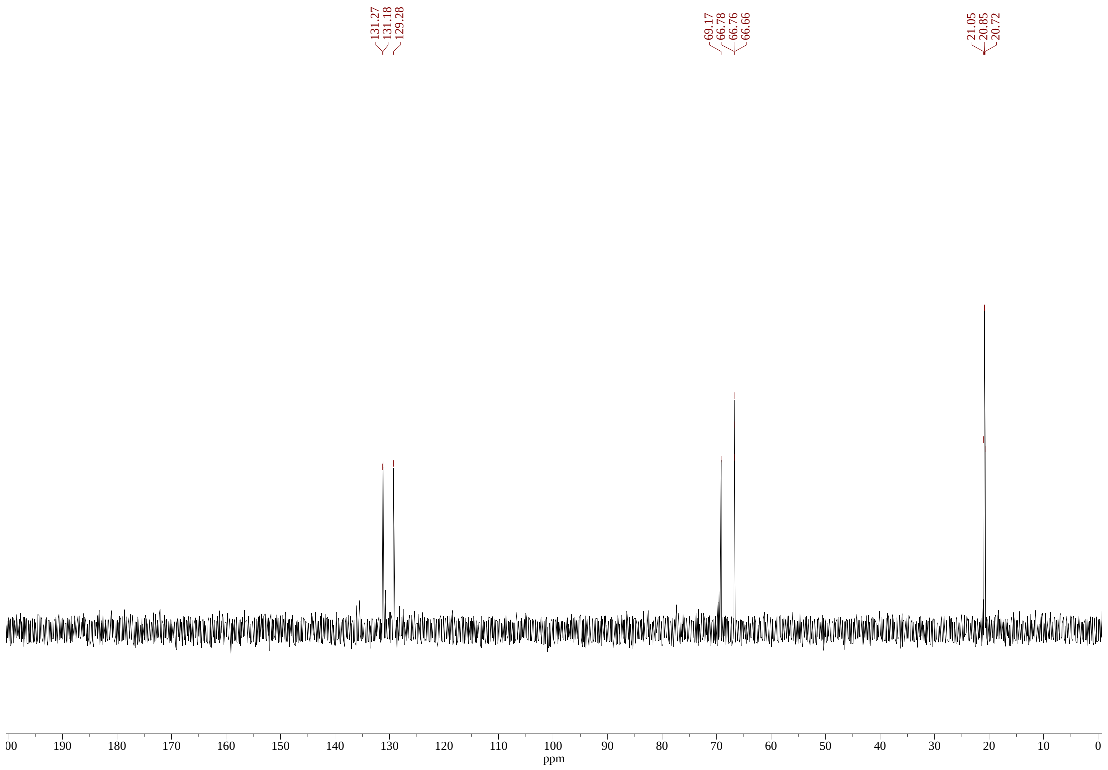

3p^anti^ - DEPTQ ($CDCl_3$)

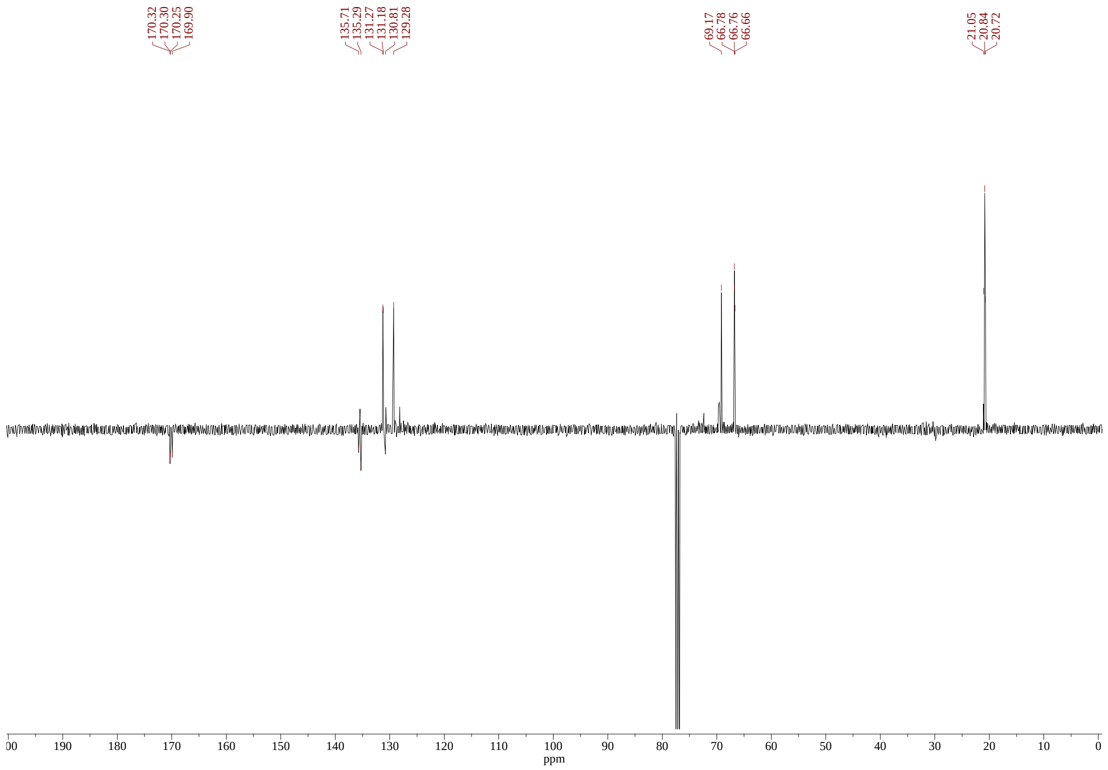

3p^anti^ - ^1H-^1H COSY ($CDCl_3$)

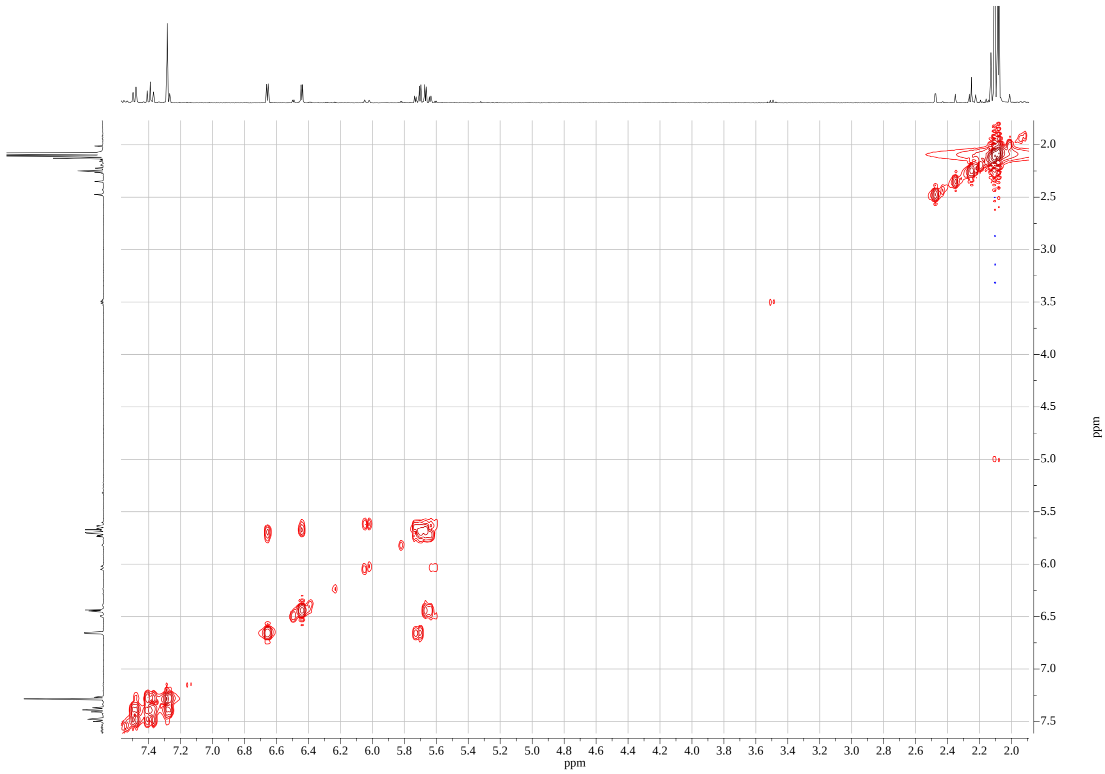

3p[anti] - ^{1}H-^{13}C HSQCED (CDCl$_3$)

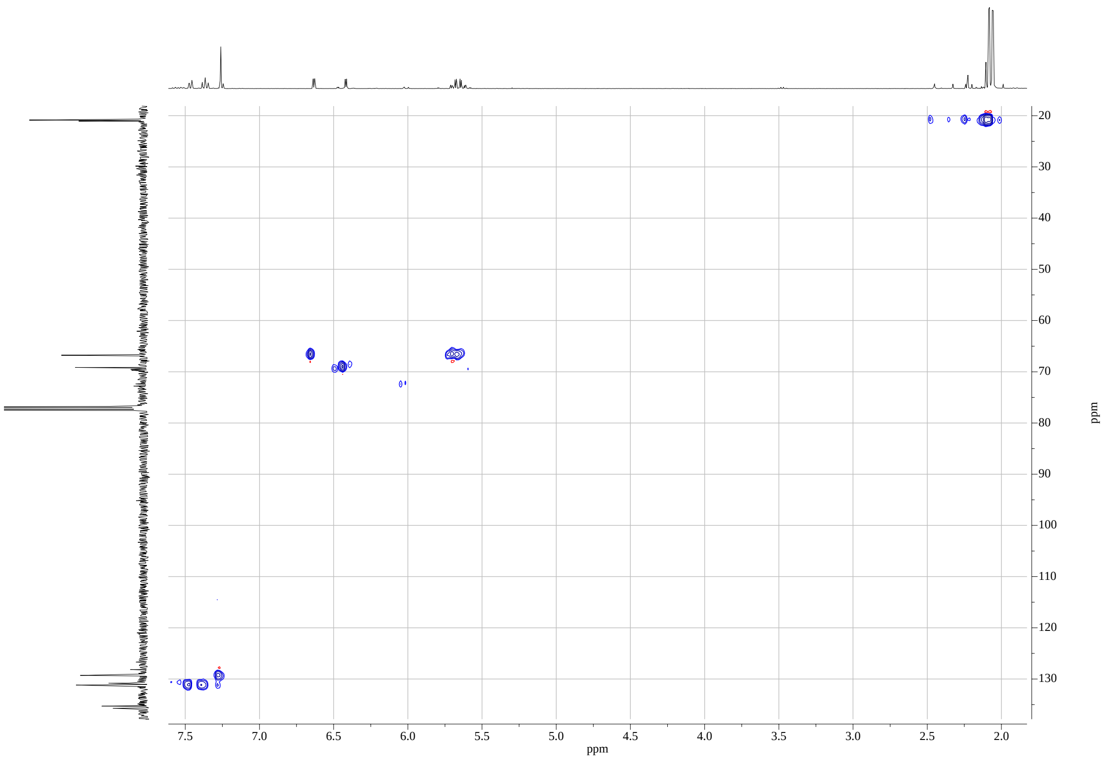

3p[anti] - ^{1}H-^{13}C HMBC (CDCl$_3$)

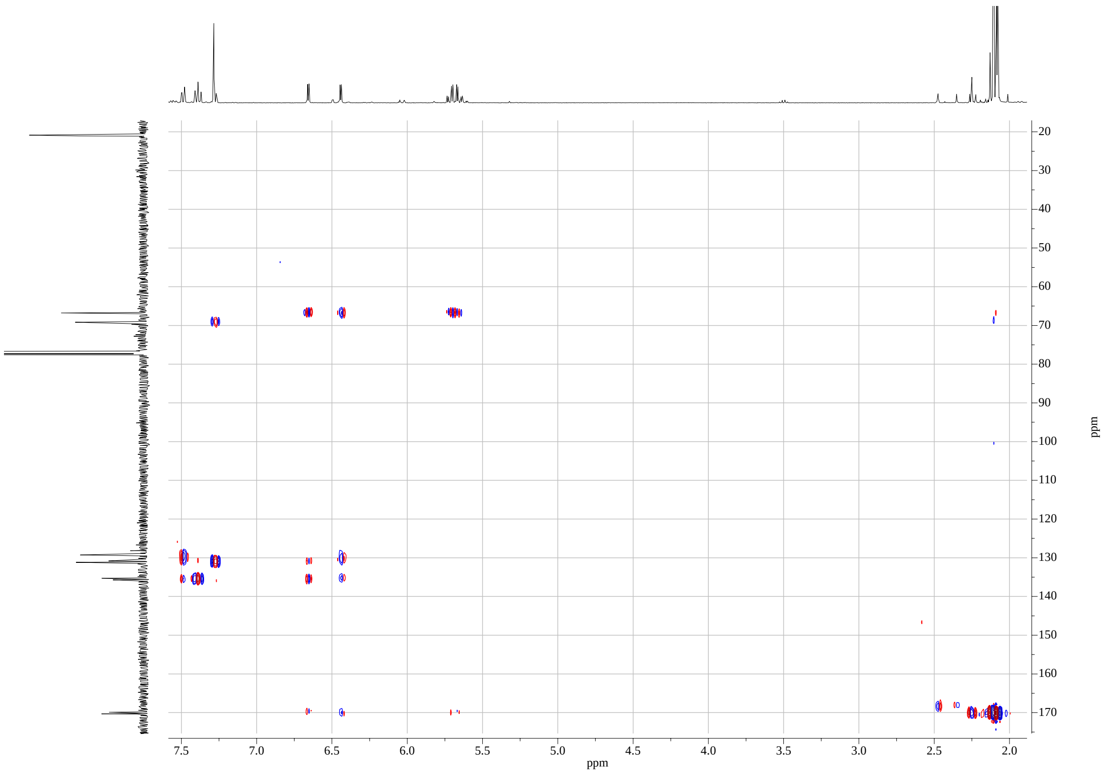

(1α,2α,3α,4α)-5-ethyl-1,2,3,4-tetrahydronaphthalene-1,2,3,4-tetrayl tetraacetate (3n^{syn})

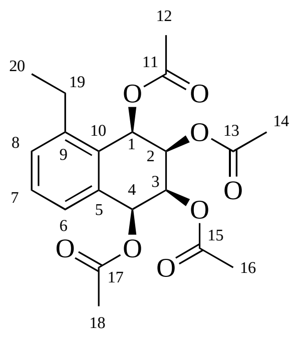

3n^{syn} - ^{1}H NMR (400 MHz, $CDCl_3$)

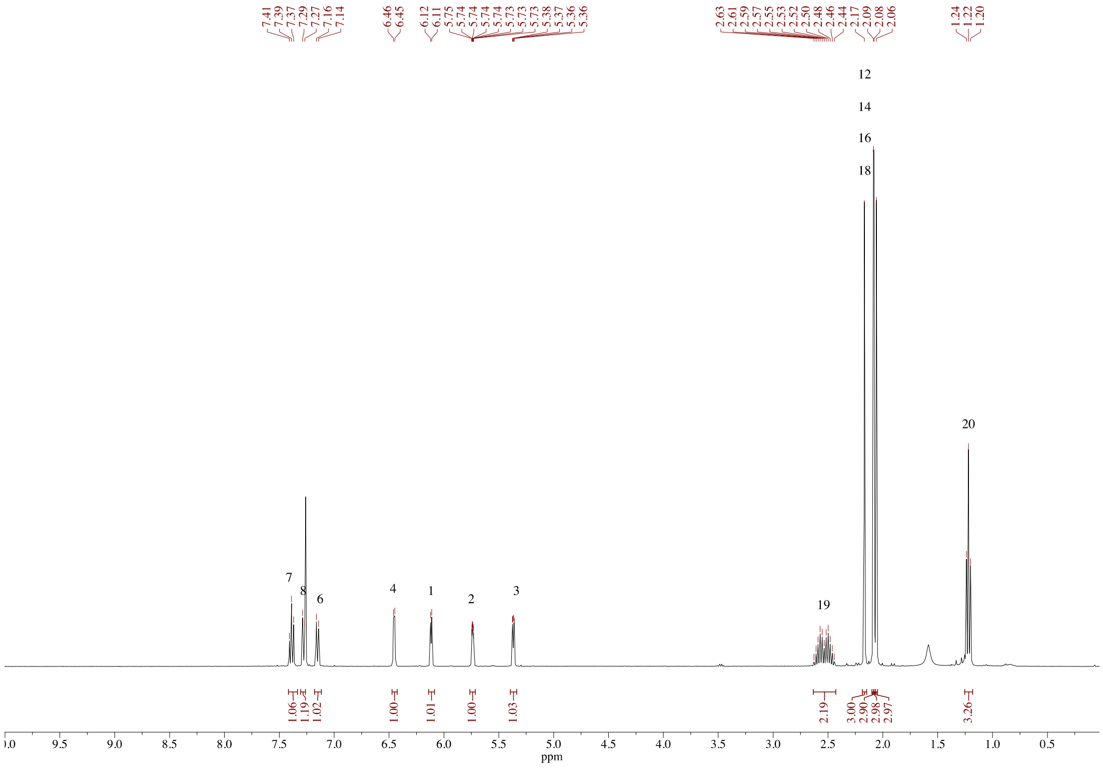

3n^{syn} - ^{13}C NMR (100 MHz, $CDCl_3$)

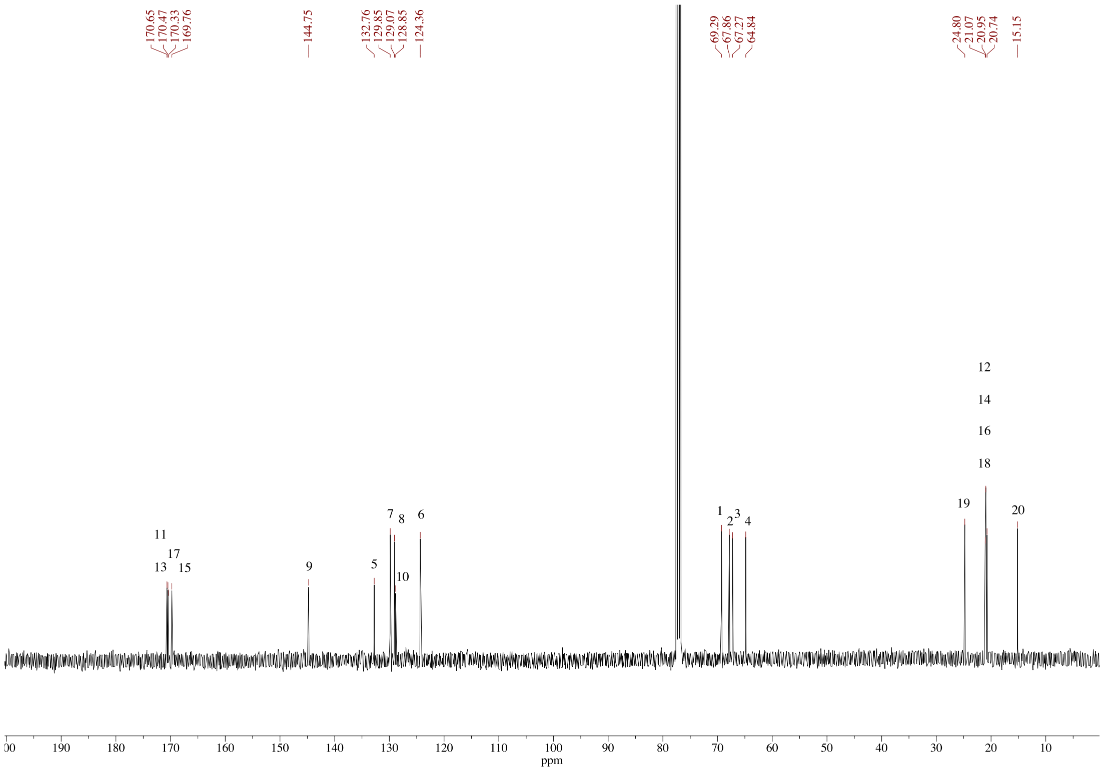

3n^{syn} - DEPT ($CDCl_3$)

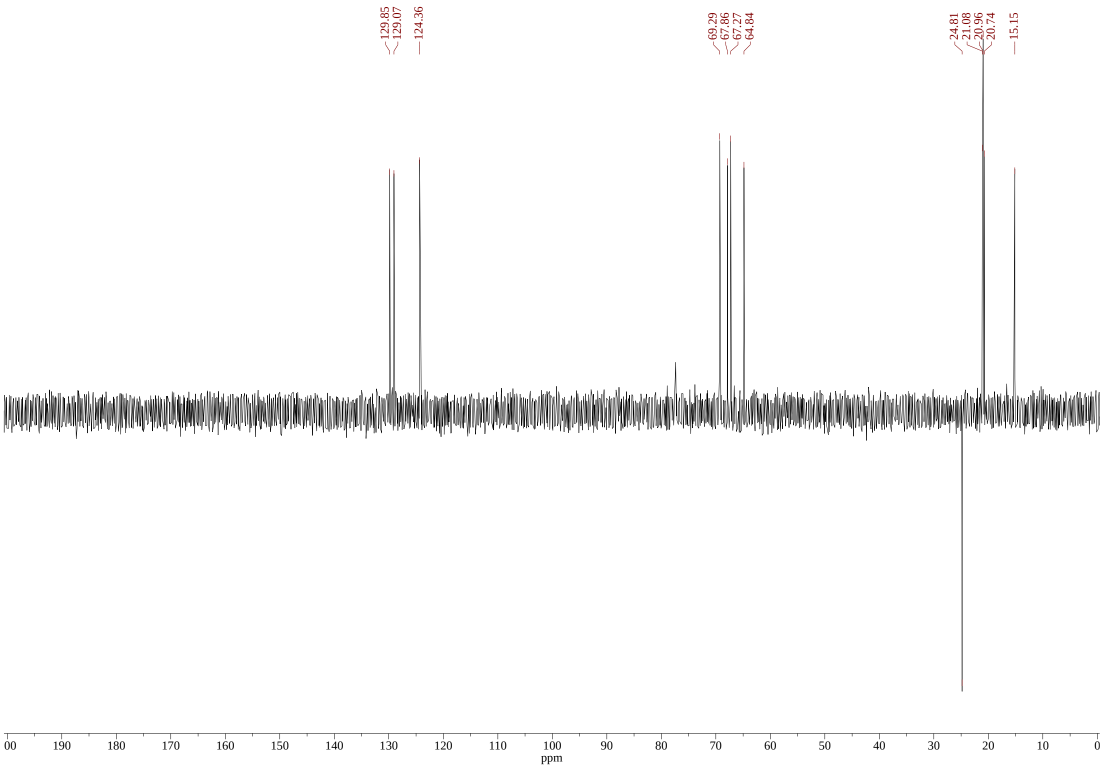

3n^syn - DEPTQ ($CDCl_3$)

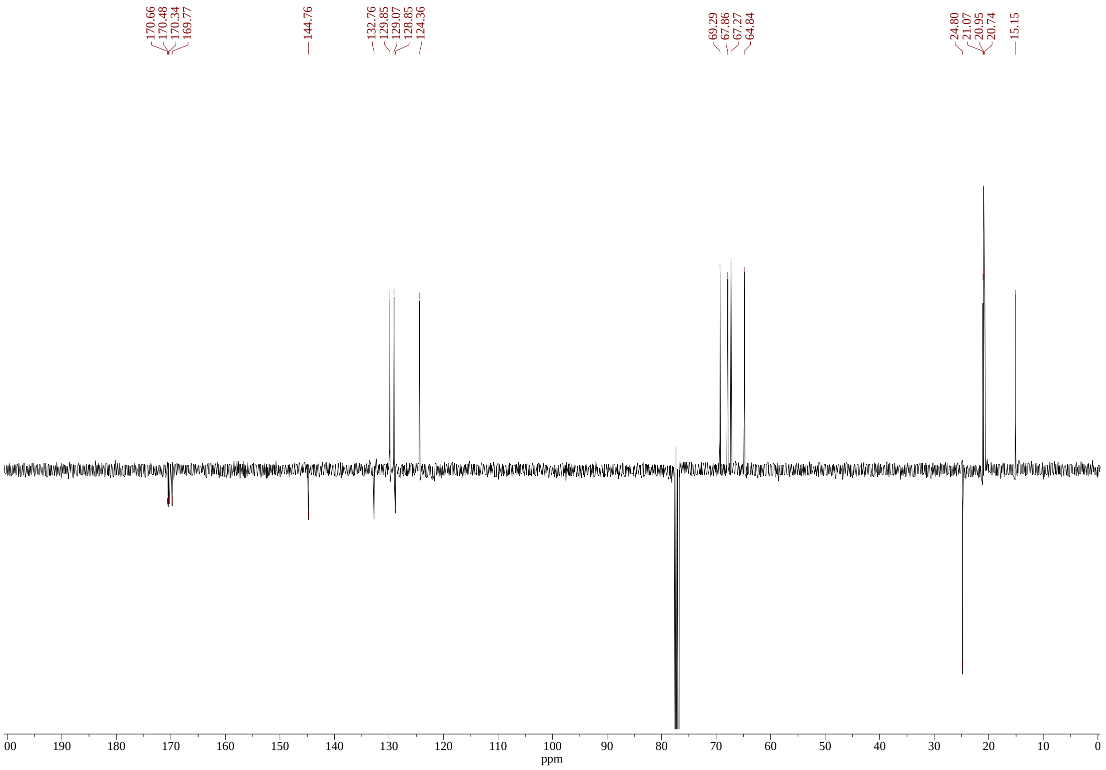

3n^syn - ^{1}H-^{1}H COSY ($CDCl_3$)

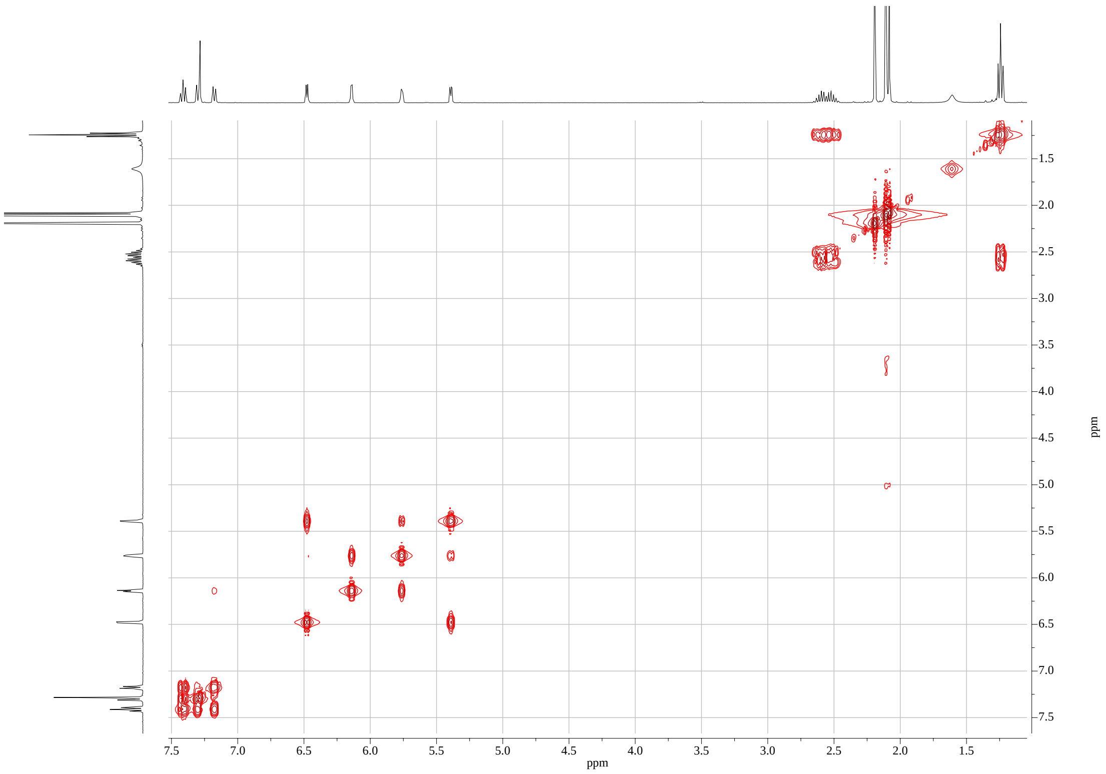

3n^{syn} - ^{1}H-^{13}C HSQCED (CDCl$_3$)

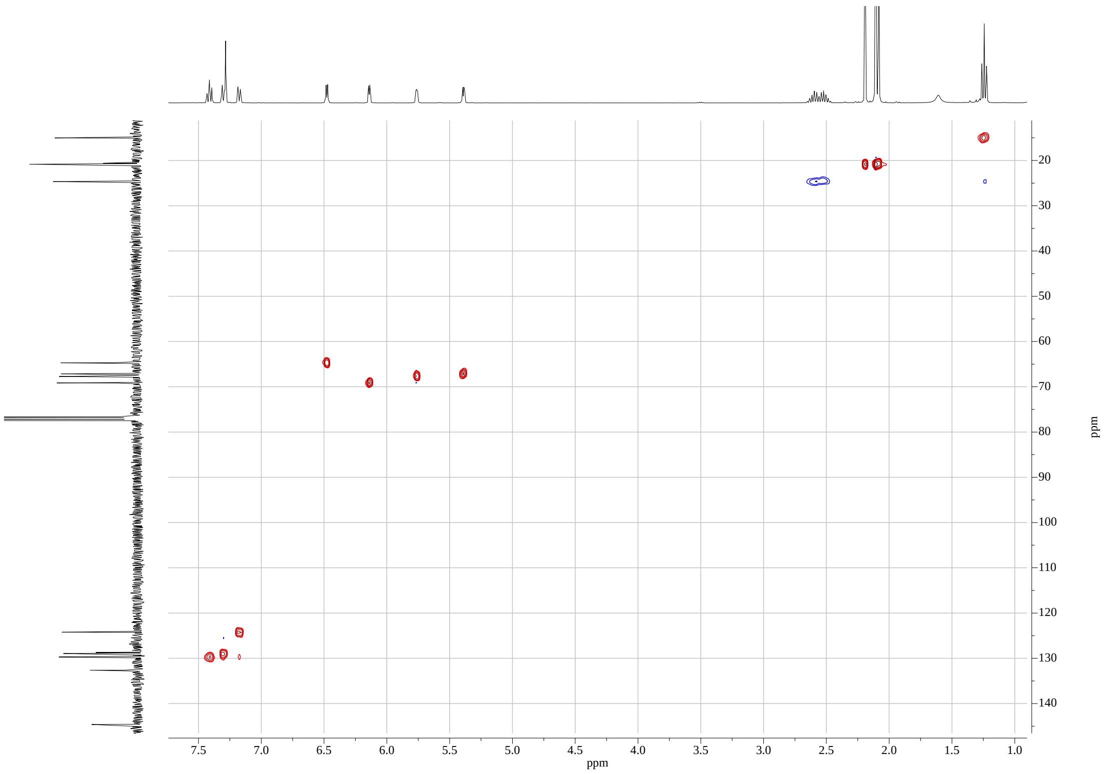

3n^{syn} - ^{1}H-^{13}C HMBC (CDCl$_3$)

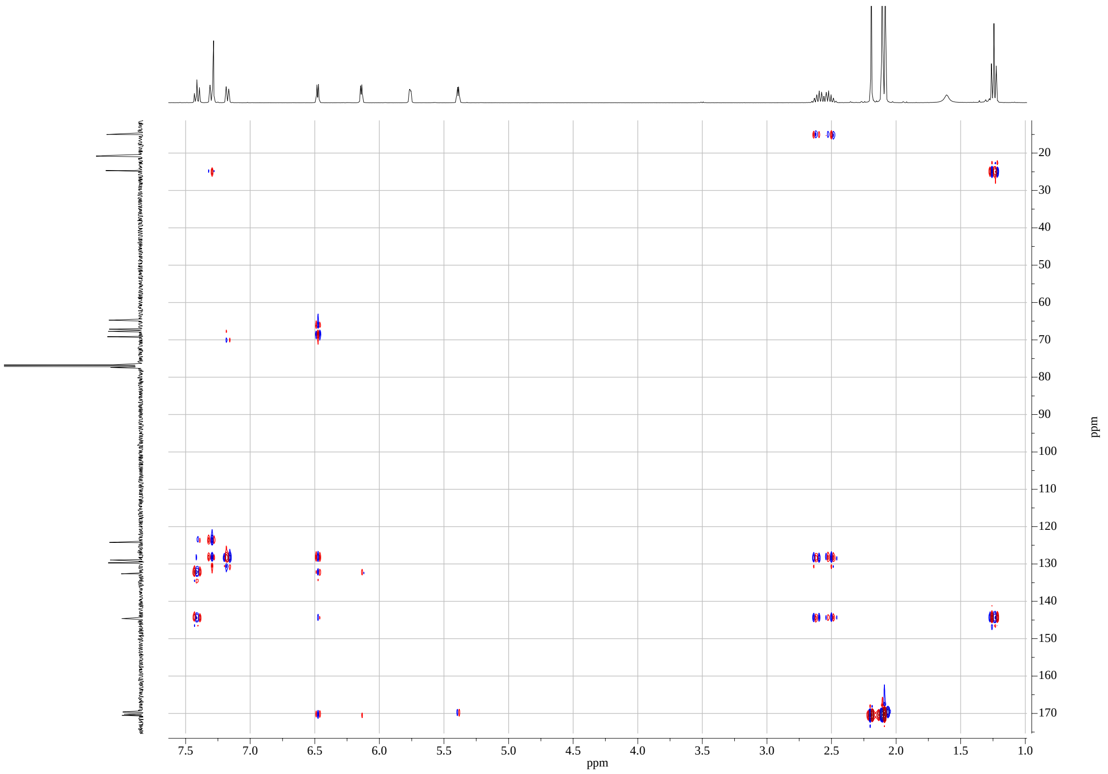

Crude of the reaction with 1-Fluoronaphtalene

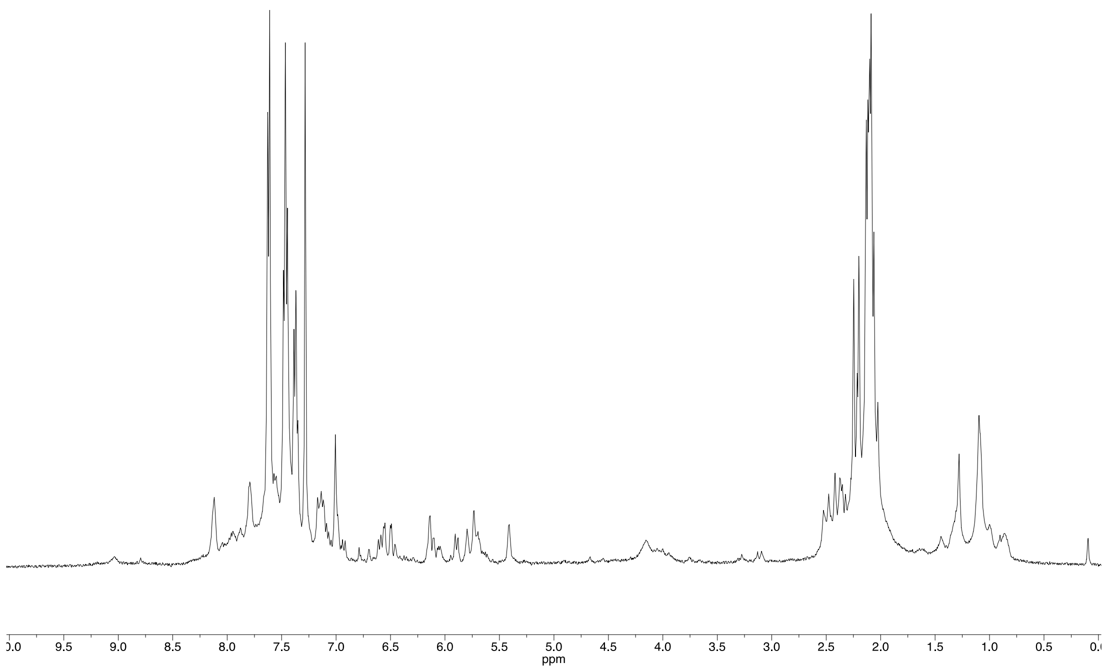

13. References

(1) Yan, G., Zhang, L. and Yu, J. Copper-Catalyzed Nitration of Arylboronic Acids with Nitrite Salts Under Mild Conditions: An Efficient Synthesis of Nitroaromatics. *Letters in Organic Chemistry* **2012**, *9*, 133-137.
(2) Barder, T. E., Walker, S. D., Martinelli, J. R. and Buchwald, S. L. Catalysts for Suzuki−Miyaura Coupling Processes: Scope and Studies of the Effect of Ligand Structure. *J. Am. Chem. Soc.* **2005**, *127*, 4685-4696.
(3) Zhang, C. X., Kaderli, S., Costas, M., Kim, E.-i., Neuhold, Y.-M., Karlin, K. D. and Zuberbühler, A. D. Copper(I)−Dioxygen Reactivity of [(L)CuI]+ (L = Tris(2-pyridylmethyl)amine): Kinetic/Thermodynamic and Spectroscopic Studies Concerning the Formation of Cu−O2 and Cu2−O2 Adducts as a Function of Solvent Medium and 4-Pyridyl Ligand Substituent Variations. *Inorg. Chem.* **2003**, *42*, 1807-1824.
(4) Venkateswarlu, K., Suneel, K., Das, B., Reddy, K. N. and Reddy, T. S. Simple Catalyst-Free Regio- and Chemoselective Monobromination of Aromatics Using NBS in Polyethylene Glycol. *Synthetic Communications* **2008**, *39*, 215-219.
(5) Borrell, M. and Costas, M. Mechanistically Driven Development of an Iron Catalyst for Selective Syn-Dihydroxylation of Alkenes with Aqueous Hydrogen Peroxide. *J. Am. Chem. Soc.* **2017**, *139*, 12821-12829.
(6) Britovsek, G. J. P., England, J. and White, A. J. P. Non-heme Iron(II) Complexes Containing Tripodal Tetradentate Nitrogen Ligands and Their Application in Alkane Oxidation Catalysis. *Inorg. Chem.* **2005**, *44*, 8125-8134.
(7) Feng, Y., Ke, C.-y., Xue, G. and Que, L. Bio-inspired arenecis-dihydroxylation by a non-haem iron catalyst modeling the action of naphthalene dioxygenase. *Chem. Commun.* **2008**, 52.
(8) Prat, I., Company, A., Postils, V., Ribas, X., Que Jr, L., Luis, J. M. and Costas, M. The Mechanism of Stereospecific C-H Oxidation by Fe(Pytacn) Complexes: Bioinspired Non-Heme Iron Catalysts Containing cis-Labile Exchangeable Sites. *Chem. Eur. J.* **2013**, *19*, 6724-6738.
(9) Company, A., Gómez, L., Fontrodona, X., Ribas, X. and Costas, M. A Novel Platform for Modeling Oxidative Catalysis in Non-Heme Iron Oxygenases with Unprecedented Efficiency. *Chem. Eur. J.* **2008**, *14*, 5727-5731.
(10) Eberhardt, M. K. and Colina, R. The reaction of OH radicals with dimethyl sulfoxide. A comparative study of Fenton's reagent and the radiolysis of aqueous dimethyl sulfoxide solutions. *J. Org. Chem.* **1988**, *53*, 1071-1074.
(11) Wang, L., Li, B., Dionysiou, D. D., Chen, B., Yang, J. and Li, J. Overlooked Formation of H2O2 during the Hydroxyl Radical-Scavenging Process When Using Alcohols as Scavengers. *Environmental Science & Technology* **2022**, *56*, 3386-3396.
